# A complex metabolic network and its biomarkers regulate laccase production in white-rot fungus *Cerrena unicolor* 87613

**DOI:** 10.1186/s12934-024-02443-9

**Published:** 2024-06-08

**Authors:** Long-Bin Zhang, Xiu-Gen Qiu, Ting-Ting Qiu, Zhou Cui, Yan Zheng, Chun Meng

**Affiliations:** 1 College of Biological Science and Engineering, Fuzhou University, Fuzhou, Fujian 350108 China; 2 The Key Laboratory of Marine Enzyme Engineering of Fujian Province, Fuzhou University, Fuzhou, Fujian 350108 China

**Keywords:** Laccase production, White rot fungi, *Cerrena unicolor*, Fructose, Metabolic networks, Regulation mechanism

## Abstract

**Background:**

White-rot fungi are known to naturally produce high quantities of laccase, which exhibit commendable stability and catalytic efficiency. However, their laccase production does not meet the demands for industrial-scale applications. To address this limitation, it is crucial to optimize the conditions for laccase production. However, the regulatory mechanisms underlying different conditions remain unclear. This knowledge gap hinders the cost-effective application of laccases.

**Results:**

In this study, we utilized transcriptomic and metabolomic data to investigate a promising laccase producer, *Cerrena unicolor* 87613, cultivated with fructose as the carbon source. Our comprehensive analysis of differentially expressed genes (DEGs) and differentially abundant metabolites (DAMs) aimed to identify changes in cellular processes that could affect laccase production. As a result, we discovered a complex metabolic network primarily involving carbon metabolism and amino acid metabolism, which exhibited contrasting changes between transcription and metabolic patterns. Within this network, we identified five biomarkers, including succinate, serine, methionine, glutamate and reduced glutathione, that played crucial roles in co-determining laccase production levels.

**Conclusions:**

Our study proposed a complex metabolic network and identified key biomarkers that determine the production level of laccase in the commercially promising *Cerrena unicolor* 87613. These findings not only shed light on the regulatory mechanisms of carbon sources in laccase production, but also provide a theoretical foundation for enhancing laccase production through strategic reprogramming of metabolic pathways, especially related to the citrate cycle and specific amino acid metabolism.

**Supplementary Information:**

The online version contains supplementary material available at 10.1186/s12934-024-02443-9.

## Background

White rot fungi (WRF), renowned as natural degraders, possess the capability to degrade both lignin and cellulose biopolymers within lignocellulose biomass [1]. These fungi are noteworthy for their broad substrate non-specificity, which gives them the capacity to alter or break down an extensive spectrum of xenobiotics [2–4]. The efficacy of WRF in degrading various xenobiotics is ascribed to their secretion of an array of bioactive substances, which can be broadly classified as either low-molecular-weight (LMW) compounds or high-molecular-weight (HMW) compounds [5]. The LMW compounds, including phenolic compounds, indoles and other secondary metabolites, exhibit antioxidative, antibacterial, anticancer and other activities [6–8], while the HMW compounds primarily consist of peptides and enzymes [6]. Among these enzymes, laccase stands out as a significant HMW fraction with considerable potential for pharmaceutical and industrial applications [9–11].

Laccases (EC 1.10.3.2) are a multicopper oxidoreductase capable of oxidizing a wide range of substrates with the concomitant reduction of O_2_ to water [12]. Consequently, they are employed as versatile and eco-friendly biocatalysts in various pharmaceutical and industrial sectors [11]. For instance, laccases from *Cerrena unicolor* have been developed for use in antiviral or anticancer treatments [13, 14], as well as for bioremediation in food industries, textile industries, cosmetics, pollutant degradation and numerous other applications [11, 15, 16]. Given the considerable demands for laccase in various pharmaceutical and industrial applications, efforts continue to focus on enhancing laccase production. Although hetero-expression represents an efficient strategy for overproducing industrial enzymes, achieving high expression of laccases in heterologous systems has not been accomplished yet. Antosova summarized the limited production of hetero-expressed laccases within the range of 0.034–380,000 U/L [17]. In comparison, WRF, such as *C. unicolor*, are capable of yielding laccase in amounts ranging from 500–2,800,000 U/L [18]. Therefore, enhancing laccase production from their natural WRF sources remains the preferred choice.

Over the past decades, two primary approaches have been used to enhance WRF laccase production: screening for new WRF species with tremendous capabilities of laccase synthesis and optimizing cultivation conditions [19, 20]. While the screening of new WRF species is serendipitous and uncertain, intentional optimization of various cultivation parameters offers a more purposeful approach to enhancing WRF laccase productivity [21]. Among these parameters, the selection of a carbon source plays a pivotal role in mediating laccase production. For instance, *Pleurotus sajor*-*caju* cultivated with glucose or fructose exhibited higher levels of laccase production compared to those cultivated with lactose [22]. Similarly, in *Phellinus noxius* hpF17, laccase production increased by 1.4-fold only when glucose was used as the carbon source, whereas sucrose or cellobiose did not yield such enhancements [23]. However, glucose repressed the transcription of the laccase isoenzyme *lap2* in *Trametes pubescens* [24]. Fructose was found to be the most efficient carbon source for *Pycnoporus sanguineus* laccase production, followed by sucrose, glucose, and maltose successively [25]. Conversely, laccase production of *Ganoderma* sp. WR-1 was four times higher when starch was the sole carbon source compared to when fructose was utilized [26]. Additionally, co-cultivating different WRF species has been identified as a useful strategy for enhancing laccase production. Notably, a study of *Coprinopsis cinerea* demonstrated that the enhancement of its laccase productivity relied on the metabolization of sucrose into fructose by the co-cultivated strain *Gongronella* sp. w5 [27]. Collectively, these studies underscore the significant influence of carbon sources on WRF laccase productivity. Despite ongoing efforts by scientists to optimize carbon sources to enhance WRF laccase productivity as a standardized process [28–30], the regulatory mechanism underlying these phenomena has received insufficient attention and remains unclear in the long term. In addition to conventional strategies, technological innovations have spurred advancements in genetic and protein engineering [20]. However, the lack of a clear understanding of regulatory mechanisms governing laccase production hampers the implementation of genetic engineering strategies as well. Therefore, investigating the intrinsic mechanisms of carbon sources in regulating WRF laccase productivity is crucial for devising new approaches to improve laccase production.

Here, we assessed the impact of various tested carbon sources on laccase activity of *C. unicolor* 87613, a promising WRF cell factory for laccase production. Our findings revealed the most significant alteration in laccase activity in *C. unicolor* 87613 from *f*ructose-*c*ultivation *d*ay 6 (FCd-6) to FCd-10. Therefore, we subsequently conducted an integrated analysis combining transcriptomics and metabolomics of fructose-cultivated *C. unicolor* 87613, using high-throughput RNA sequencing and LC-MS/MS technologies. Our study provides a comprehensive dataset of changes in both transcriptomic and metabolomic profiles in *C. unicolor* 87613. Based on this analysis, we have elucidated the putative mechanisms underlying the modulation of laccase production by fructose. Our findings contribute to a better and more comprehensive understanding of *C. unicolor*, and provide a theoretical foundation for augmenting laccase production by reprograming the intracellular metabolic networks.

## Methods

### Microbial strains and culture conditions

A strain of *Cerrena unicolor*, cataloged as numeral 87613, was acquired from the China Forestry Culture Collection Center (CFCC) and preserved under optimal conditions at the Key Laboratory of Marine Enzyme Engineering of Fujian Province, Fuzhou University. The strain was revived on Potato Dextrose Agar media (PDA solid media) under stationary conditions at 30 ℃ for 4–5 days. Culture plugs measuring 5 mm in diameter were excised with a sterile cork borer and transferred to PDA liquid media supplemented with a variety of carbon sources. These samples were then incubated at 200 rpm and 30 ℃. Specifically, each carbon source (glucose, fructose, sucrose, lactose, starch, and dextrin) was provided at a concentration of 20 g/L in the liquid media.

### Determination of laccase activities

Laccase activities were assayed over a period of 2 to 12 days in cultures supplemented with various carbon sources. Utilizing 2,2’-azino-bis-(3-ethylbenzothiazoline-6-sulfonic acid) (ABTS, Sigma-Aldrich, St. Louis, MO, USA) as a chromogenic substrate, we quantified the oxidation rate catalyzed by the extracellular laccase in the supernatant, and spectrophotometric readings were taken at an absorbance of 420 nm. The reaction system consisting of cultivation supernatant (enzyme solution, 25 µL), sodium acetate solution (0.1 M, pH 3.0, 975 µL), ABTS (0.5 mM, 1000 µL) were incubated at 45 ℃ for 5 min [31]. One unit of laccase activity (U) was defined as the quantity of laccase required to convert 1 µmol ABTS per minute. The assays were performed in triplicate.

### RNA sequencing and transcriptomic analysis at two cultivation periods

The assays that revealed peak and minimal laccase activity on days 6 and 10 of cultivation with fructose, resulted in the harvesting of samples on fructose-cultivation day 6 and day 10 (FCd-6 and FCd-10), respectively. Total RNA was extracted from three replicates using RNAiso™ Reagent (TaKaRa, Dalian, China), and subsequently sequenced on an Illumina NovaSeq 6000 platform at Novogene Company (Beijing, China). Raw data (NCBI GEO: GSE236542) in fastq format were initially processed using an in-house Perl script to obtain clean data. Subsequently, the data were mapped to the *C. unicolor* 87613 genome assembled from next-generation sequencing data (NCBI SRA: SRR23097119) [32] and normalized as fragments per kilobase of exon per million fragments mapped (FPKM) using Hisat2 v2.0.5 and featureCounts v1.5.0-p3 with the order of “featureCounts -T 4 -F GTF -t exon -g gene_id -s 0 -Q 10 -C -B -p”, respectively. The differently expressed genes (DEGs) were accepted at significant levels of |log2 (FCd-6/FCd-10 ratio)| ≥ 1 and of *P*-value < 0.05. The DAVID tool (https://david.ncifcrf.gov/) was used for Gene Ontology (GO) analysis. All DEGs were categorized based on their annotated functions [33]. To proceed Kyoto Encyclopedia of Genes and Genomes (KEGG) analysis, an online database (https://www.kegg.jp/kegg/) was used to enrich the DEGs in various pathways at a significant level of *P*-value < 0.05 [34].

### Assay and analysis of metabolomic profiles at two cultivation periods

Parallel to transcriptomic investigations, metabolites were extracted from FCd-6 and FCd-10 cultures grounded with liquid nitrogen, and profiled via LC-MS/MS by Novogene Company (Beijing, China). The analysis was performed using a Vanquish UHPLC system (Thermo Fisher) coupled with an Orbitrap Q Exactive HF-X mass spectrometer (Thermo Fisher). Raw data were calculated and analyzed following the methodology of previous studies [35, 36]. The chemistry compounds were defined by mapping them to three chemical-compound banks (ChemSpider, mzCloud and/or mzVault). The parameters of |log2 (FCd-6/FCd-10 ratio)| ≥ 1 and of *P*-value < 0.05 were used to identify the differentially abundant metabolites (DAMs). The online server (Metaboanalyst 6.0, https://www.metaboanalyst.ca/) was used to categorize each metabolite and enrich metabolic pathways with their impact values [37].

### Assaying for intracellular ROS levels

Aliquots of 0.1 g of hyphal cells from FCd-6 or FCd-10 cultures were collected. Subsequently, the fungal cells were ground into powder using liquid nitrogen, and then suspended in 1 mL of PBS buffer (0.1 M, pH 7.4). After centrifugation at 12,000 × g and 4 ℃, the supernatants were obtained for ROS measurement using the ROS Assays Kit (mlbio, Shanghai, China). Briefly, 50 µL of blank solution, gradient-dilution standard solutions, and dilution supernatants were added to each well of Elisa plate following the user’s guide. Thereafter, each sample was incubated with 100 µL of working solution containing horseradish peroxidase (HRP) at 37 ℃ for 60 min. Following the removal of the reaction mixtures, all wells were washed with washing buffer five times, and then successively added with 50 µL of substances A and B. After 15-minute incubation at 37 ℃ in the dark, 50-µL of terminal solution was added to all wells and absorbance was measured at 450 nm. The ROS contents in each sample were calculated based on the ROS standard curve using the sample readings.

### Assaying for intracellular contents of reduced and oxidized glutathione

The tested samples were prepared as mentioned above with the substitution of PBS buffer with buffer 1 in the Reduced or Oxidized Glutathione Assay Kits (Solarbio, Beijing, China). A 1 mL reaction system containing a sample solution (100 µL), buffer 2 (700 µL), and buffer 3 (200 µL) was prepared using a GSH Assay Kits. The GSH contents in each system were quantified at OD412 after 2 min of standing at room temperature. For GSSG quantification, 100-µL samples were pre-treated with 2-µL of buffer 2 from a GSSG Assay Kit to exclude GSH. After a 150-sec mixed reaction of pre-treated sample (102 µL), buffer 3 (700 µL), buffer 4 (100 µL), buffer 5 (100 µL) and buffer 6 (10 µL), the GSSG contents were measured at OD412. The concentration of GSH or GSSG in each reaction was calculated using the GSH or GSS standard curve with sample reading at OD_412_. The final contents of GSH and GSSG were estimated as follows:

$${\rm{GSH or GSSG }}\left( {{\rm{\mu g/g culture}}} \right) = {{\rm{C}}_{\rm{S}}} \times {{\rm{V}}_{\rm{S}}} \times {\rm{N / }}{{\rm{W}}_{\rm{S}}}$$

(C_S_ indicates the concentration of GSH or GSSG in each sample solution; V_S_ refers to the total volume of collected supernatant (1 mL); W_S_ stands for the weight of extracted cultures (0.1 g); N represents the dilution rate.)

### Biomass estimation

The growth of *C. unicolor* 87613 was quantified by measuring the dry weight of the culture biomass obtained at two-day intervals from twelve groups of identically fructose-supplemented cultures. Measurements were replicated to ensure reliability.

### Assaying for *C. Unicolor* 87613 laccase production in response to different exogenous supplies

To demonstrate the potential effect of targeted metabolites on laccase production in *C. unicolor* 87613, the strains were cultured for a 4-day submerged cultivation under optimal conditions of 30 ℃ and 200 rpm, followed by an additional 2 days of cultivation alone (control), or with varied concentrations of succinate (1, 7.5 or 15 mM), serine (0.0125, 0.05, 0.1 M), methionine (7.5, 15, 30 mM), glutamate (0.02, 0.04, 0.08 mM), or reduced glutathione (GSH, 10, 20, 30 mM) (treatment). At FCd-6 period, the supernatant of each experimental group was collected for a laccase activity assay.

In addition, total RNA were extracted from the cultures of control or treatment groups on the sixth day as mentioned above, using RNAisoTM Plus Reagent (TaKaRa, Dalian, Chine). After being reverse transcribed into cNDAs using a PrimeSript® RT reagent kit (TaKaRa), three cDNAs samples (ten-fold dilution) of each culture were used as templates to quantify the transcript levels of laccase gene family via qRT-PCR with paired primers (Table S1). Fungal 18 S rRNA was used as an internal standard. The relative transcription level of each gene was calculated as the ratio of transcripts in each treatment group over that in control group using the 2^−ΔΔCt^ method [38].

### Statistical analysis

All phenotypic parameters were quantified from the experiments with at least three replicates and were subjected to one-factor analysis of variance, followed by Tukey’s honestly significant difference (HSD) test to determine the differences in each phenotype between the control and treatment samples.

## Results and discussion

### Varied laccase production in *C. Unicolor* 87613 supplemented with different carbon sources

**Fig. 1:**
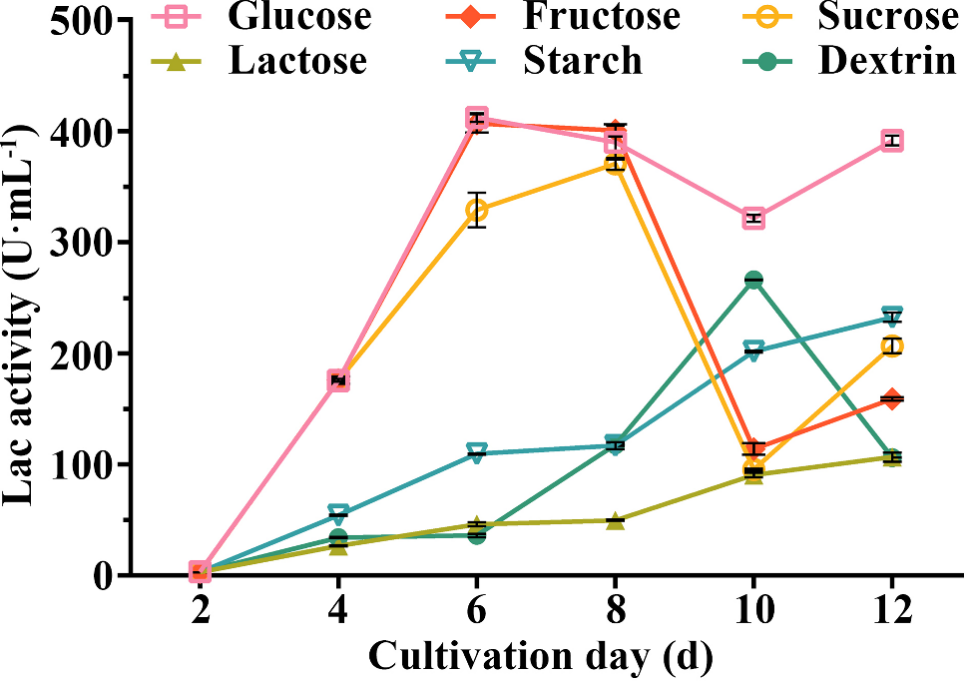
Laccase production of *C. unicolor* 87613. To assess the effect of different carbon sources, strains were incubated in PDA media supplied with 20 g/L of glucose, fructose, sucrose, lactose, starch, or dextrin as extra carbon source, respectively. Then, the laccase activity was quantified using ABTS as the substrate

*C. unicolor* 87613 has been previously identified as a superior cell factory for laccase production [32]. To further investigate its laccase-producing capabilities under varied cultivation conditions, this study examined the role of the carbon source, a critical nutrient that impacts WRF laccase productivity [19]. We assessed the influences of six prevalent carbon sources on *C. unicolor* 87613 laccase production. As shown in Fig. 1, strains cultivated with sucrose, lactose, starch and dextrin displayed laccase activities, peaking at 329.0 U/mL, 46.2 U/mL, 109.5 U/mL and 36.1 U/mL at later cultivation periods of day 8, 12, 12, and 10, respectively. In contrast, glucose and fructose resulted in the highest laccase-producing levels (412 U/mL and 407 U/mL, respectively) in the earlier growth cycle (both at cultivation day 6), suggesting their potential as advantageous carbon sources for laccase production in *C. unicolor* 87613. The efficiency of fructose utilization in laccase production by *C. unicolor* 87613 in laccase production is similar to that of *Pycnoporus sanguineus* [25], but opposite to that of *Ganoderma* sp. WR-1 [26]. More interestingly, *C. unicolor* 87613 maintained laccase activity above 321.6 U/mL during later cultivation when supplemented with glucose, while the laccase activity dropped to 107.20 U/mL by day 10 in fructose-containing media (Fig. 1). These results suggest the significant impacts of different carbon sources on the timing, level, and persistence of laccase production in *C. unicolor* 87613.

### Changes of transcription profiles in *C. Unicolor* 87613 supplemented with fructose at FCd-6 and FCd-10

**Fig. 2:**
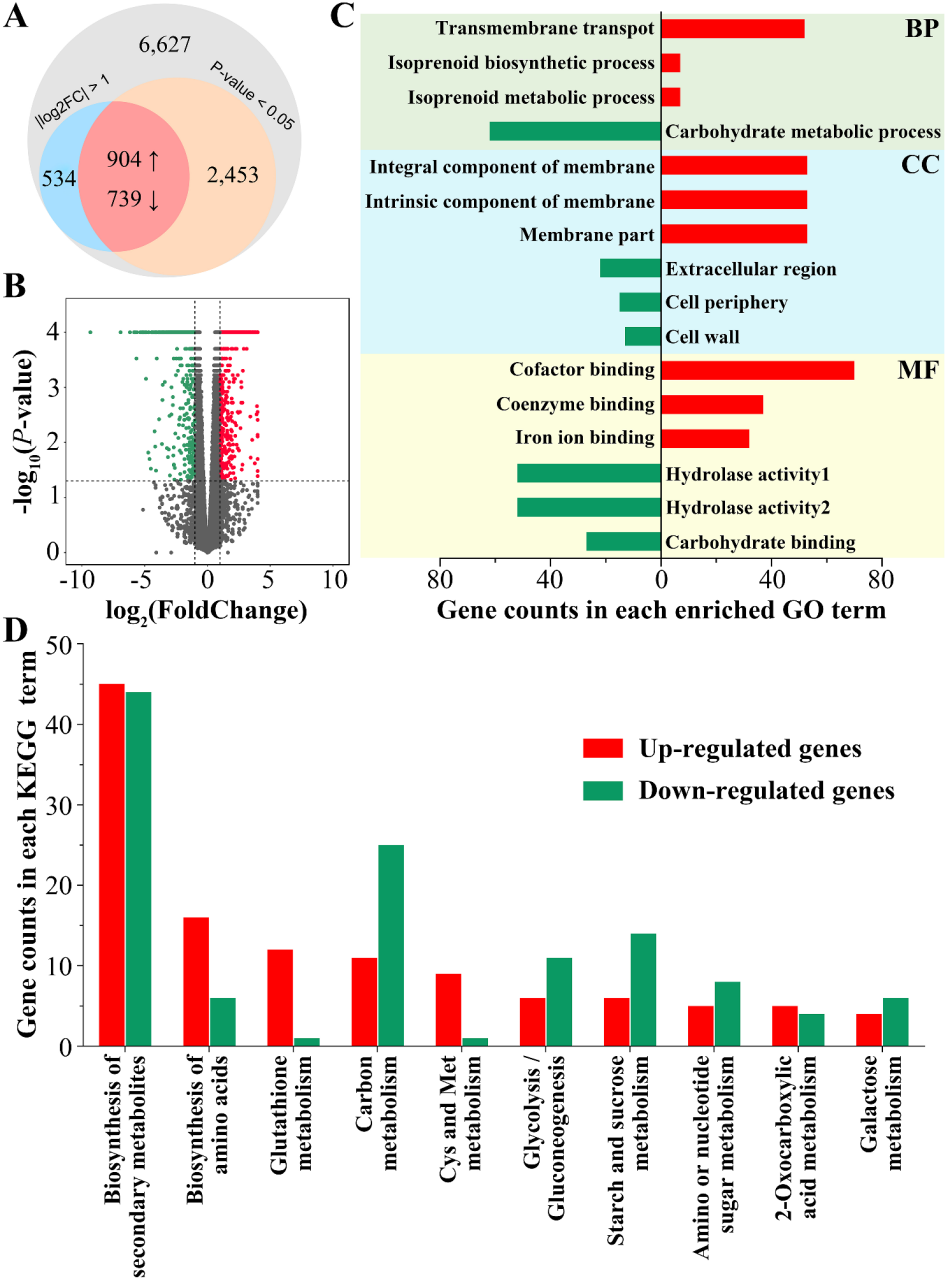
The transcription pattern of FCd-6 compared to that of FCd-10. **A** Venn diagram showing the numbers and relationship of all detected transcripts according to setting threshold limit as log2 (fold-change) > 1 or log2(fold-change) < − 1, and *P*-value < 0.05. **B** The volcano map of transcription profile of all RNAs in FCd-6 compared to those in FCd-10. The red dots indicate significantly up-regulated genes, whereas the green dots indicate down-regulated genes. The grey dots indicate that genes were not significantly changed. **C** Gene Ontology (GO) analysis of up-regulated genes (red box) and down-regulated genes (green box) in categories of Biological Process (BP), Cellular Component (CC), and Molecular Function (MF). **D** KEGG analysis of up-regulated genes (red box) and down-regulated genes (green box) in ten enriched terms

Cultivation with fructose resulted in a significantly high level of laccase production, but there was a noticeable decline from FCd-6 to FCd-10 period. It attracted us to investigate the potential mechanism underlying these phenomena. RNA sequencing is a powerful technology for uncovering intracellular transcriptional changes potentially associated with various cell processes, e.g. laccase production. Therefore, three replicated cultures from FCd-6 and FCd-10 were collected and used for RNA sequencing. The outcome data were reliable with the values of Q20 (> 97%), Q30 (> 93%) and the percentage of mapped reads to total reads (> 93%) (Table S2). Subsequently, a total of 11,257 transcripts were obtained from these data. Among those, 1,643 genes with |log2 (FCd-6/FCd-10 ratio)| ≥ 1 and of *P*-value < 0.05 were considered as differentially expressed genes (DEGs), including 904 up-regulated genes and 739 down-regulated genes in FCd-6 cultures compared to those in FCd-10 (Fig. 2A, B and Table S3). The DEGs were analyzed using the Gene Ontology (GO) database, resulting in three main categories: Biological Process (BP), Cellular Component (CC) and Molecular Function (MF) (Table S4). As illustrated in Fig. 2C, top three significantly enriched GO terms of each category were presented. Regarding up-regulated DEGs (UDEGs) enrichment, the term of “transmembrane transport” in BP harbored the highest number of UDEGs (up to 52 genes), while the remaining eleven BP terms were mainly involved in various metabolic processes, each enriched by 5–7 UDEGs, respectively (Fig. 2C and Table S4). On the other hand, down-regulated DEGs (DDEGs) only enriched into one BP term, namely “carbohydrate metabolic process”. Accordingly, we also found that DDEGs-enriched MF terms were associated with the hydrolyzation of carbohydrate (Fig. 2C), suggesting a suppression of carbohydrate metabolism during the peak phase of laccase production (FCd-6). Moreover, all three UDGEs-enriched terms in the CC category were associated with the fungal membrane, whereas components of the cell periphery and cell wall were enriched by DDGEs (Fig. 2C and Table S4). These results suggest that *C. unicolor* 87613 was undergoing more active membrane construction but weaker cell wall maintenance at FCd-6. Interestingly, fungal laccases are generally secreted enzymes [12]. Activation of the cell membrane and weakness of the cell wall might promote the secretion of fungal laccase, resulting in increased laccase production. In addition, laccase activity also depends on the binding of multiple factors, such as Cu^2+^, Fe^2+^ and Mn^2+^ [39, 40]. In this case, UDGEs-enriched MF terms were found to be involved in the binding of cofactor, coenzymes or ions (Fig. 2C and Table S4), which might contribute to the high level of laccase activity at FCd-6.

Furthermore, all DEGs were analyzed by KEGG and sorted into sixteen pathways (Table S5). Ten of the most significantly enriched terms were associated with the biosynthesis of secondary metabolites and amino acids, or the metabolism of glutathione, carbon, and other carbohydrates (Fig. 2D and Table S5). Notably, the coexistence of up- and down-regulated genes within these pathways complicates the assessment of their exact contributions to laccase production.

### Variations in metabolomic profiles between FCd-6 and FCd-10 periods

**Fig. 3:**
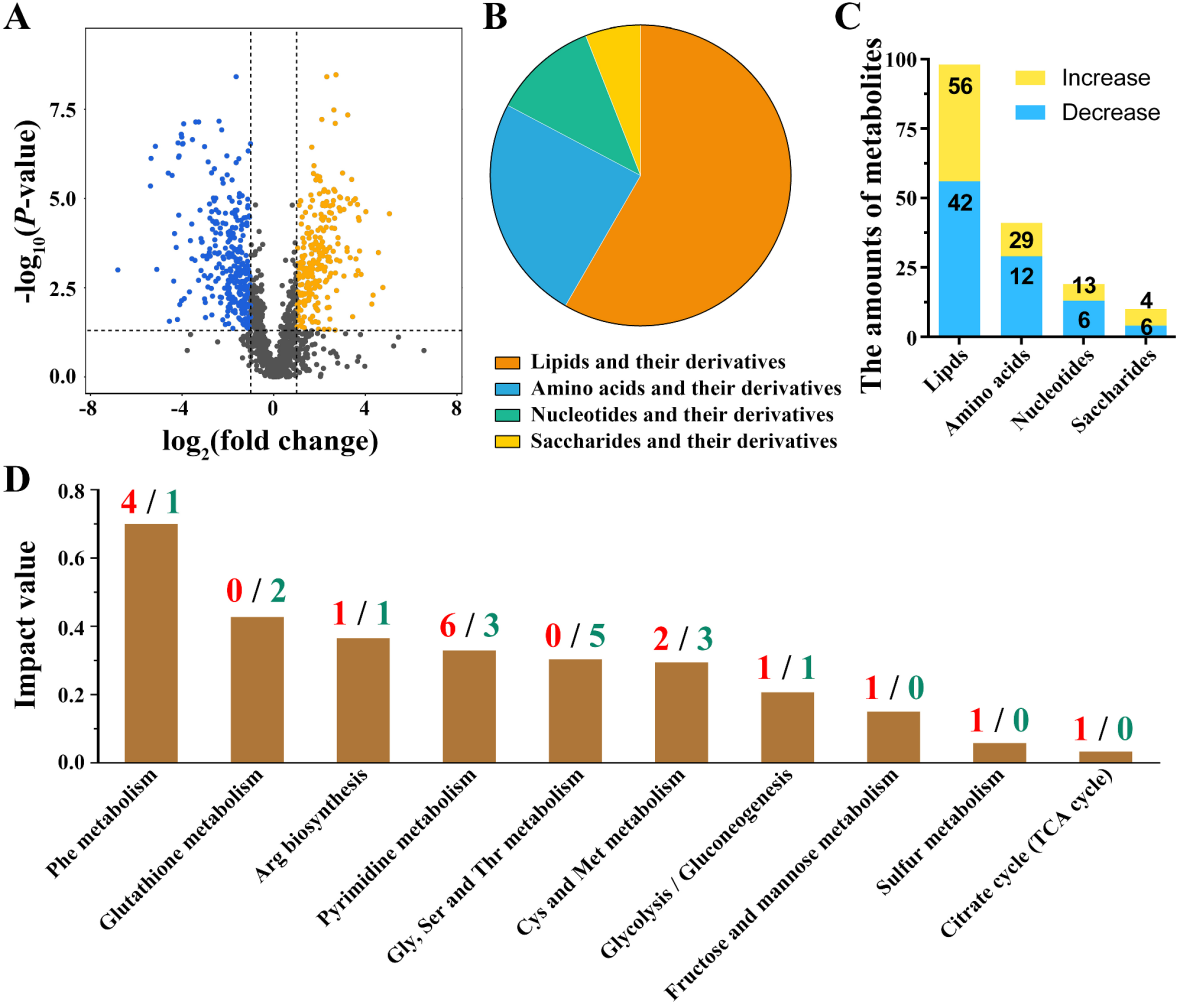
The metabolic pattern of FCd-6 compared to that of FCd-10. **A** The volcano map of metabolites profile in FCd-6 compared to those in FCd-10. The increased metabolites were marked in orange dots, whereas the decreased metabolites were marked in blue dots. **B** Categories of metabolites exhibiting differential abundances. **C** Number of metabolites with differential abundance. **D** Metabolic pathways enriched by differentially abundant metabolites (DAMs) using MetaboAnalyst 6.0 online server. The red and green number indicate the increased or decreased DAMs in each pathway, respectively

Complementing our transcriptomic detection, LC-MS/MS was conducted to measure the metabolomic changes between FCd-6 and FCd-10. Principal components analysis (PCA) with PC1 value of 46.99% and PC2 value of 18.98% confirmed a clear distinction between the two growth stages (Fig. S1A). Of the total 1,347 metabolites, 257 were differentially abundant (DAMS) with |log2 (FCd-/FCd-10 ratio)| ≥ 1, of *P*-value < 0.05 and of VIP (variable importance in the projection) score ≥ 1 (Fig. S1B, Fig. 3A and Table S6). According to the analysis of the online platform (MetaboAnalyst 6.0) [37], 91 of these DAMs were classified as lipids and their derivatives, 41 were sorted into amino acids, 19 were defined as nucleotides and their derivatives, and 10 were classified as saccharides and their derivatives (Fig. 3B). During the FCd-6 period, the amounts of increased DAMs in lipids, amino acids and nucleotides categories were higher than those in the FCd-10 period (Fig. 3C).

In addition, the enrichment of all DAMs identified twenty-nine pathways related to the metabolism of amino acids, saccharides and functional factors (including glutathione, riboflavin and NAD^+^) (Table S7). Top ten pathways with the highest impact value were presented according to the evaluation of Metaboanalyst 6.0 online server (Fig. 3D, Table S7), partially sharing the enrichments with those from the glucose-stimulated metabolome [41]. Notably, the abundance of targeted metabolites within these pathways was differentially altered from FCd-6 to FCd-10 periods (Fig. 3D), which might contribute to the decline of laccase production in *C. unicolor* 87613.

### Comprehensive illustration of pathways by combination analysis of two omics

**Fig. 4:**
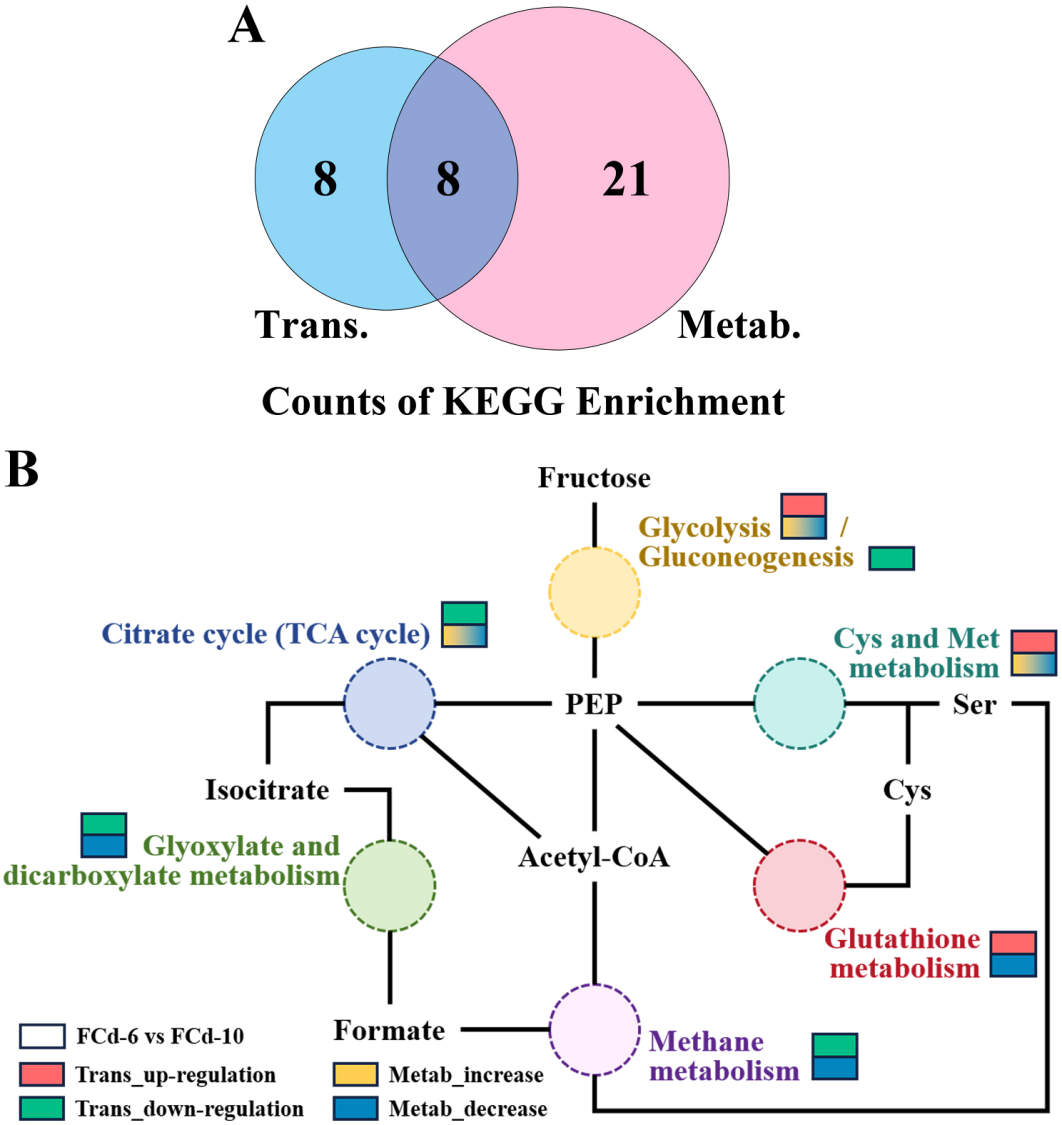
Combination analysis of transcriptome and metabolome resulting **A** eight co-enriched pathways. **B** six of them constituted a complicated metabolic network with different changes of transcription and metabolic profiles. The left and right box indicated FCd-6 and FCd-10 samples, respectively. The red box indicated significantly up-regulated pattern of transcription, whereas the green box indicated down-regulated pattern of transcription. The yellow box indicated significantly increased pattern of metabolome, whereas the blue box indicated significantly decreased pattern of metabolome

Transcriptomic and metabolomic analysis uncovered various pathways potentially regulating laccase production in *C. unicolor* 87613. To further specify the regulatory pathways, a combined analysis using both omics approaches was conducted. As a result, eight pathways were shared by these two omics, predominantly concerning carbon-related metabolism (including galactose metabolism, glycolysis/gluconeogenesis, citrate cycle, glyoxylate and dicarboxylate metabolism, and methane metabolism) and amino acid-related metabolism (including amino sugar and nucleotide sugar metabolism, cysteine and methionine metabolism, and glutathione metabolism) (Fig. 4A and Table S8). Based on the DAMs involved in each pathway, we surprisingly discovered that six of these co-enriched pathways could be interconnected as a complex metabolic network (Fig. 4B and detailed in Fig. S2). Briefly, *C. unicolor* 87613 can absorb and utilize fructose through the glycolysis pathway to produce phosphoenolpyruvate (PEP), which then diverges into two separate metabolic streams: one leading to various carbon-related metabolic processes from the citrate cycle (TCA cycle) to glyoxylate and dicarboxylate metabolism, eventually to methane metabolism; the other pathway is involved in amino acid-related metabolism, particularly in cysteine and methionine metabolism and glutathione metabolism.

### TCA cycle and its biomarker associated with laccase production

**Fig. 5:**
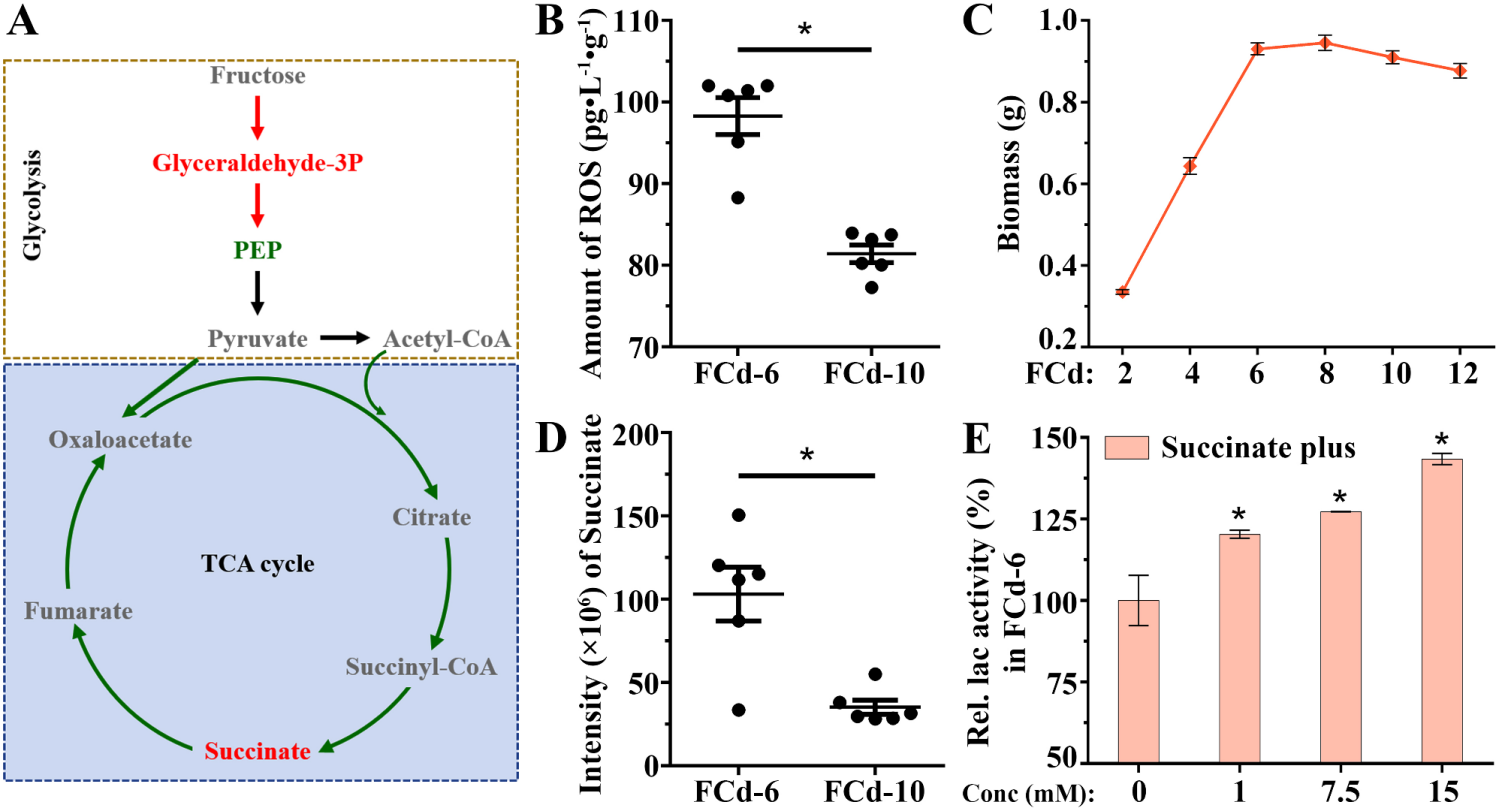
The alteration of **A** citrate cycle (TCA) cycle in FCd-6 compared to those FCd-10 as cultivated with fructose. **B** the ROS levels at FCd-6 and FCd-10 periods. **C** the biomass of fungal hyphae growing from FCd-2 to FCd-12. **D** the intensity of succinate measured by LC-MS.MS at FCd-6 and FCd-10 periods. **E** the dose-dependent induction of laccase production by additive succinate during *C. unicolor* 87613 cultivation

To gain a profound understanding of how these pathways regulate laccase production in *C. unicolor* 87613, we meticulously analyzed their intricate changes. In light of a transcriptomic study of *T. gibbose* that suggested a correlation between the TCA cycle and laccase production [42], we initially focused on the enriched TCA cycle. As shown in Fig. 5A and Fig. S2, we surprisingly found a notable down-regulation of the entire TCA cycle genes during the FCd-6 period compared to FCd-10. Given that reactive oxygen species (ROS) act as oxidative-stress inducer for laccase production [43, 44], as well as byproducts of the TCA cycle [45], we examined intracellular ROS levels during the FCd-6 and FCd-10 periods. Intriguingly, the ROS concentration was found to be 19.85% higher during FCd-6 than FCd-10, which corresponded with the respective levels of laccase production but was opposite to the TCA cycle activity (Figs. 1 and 5B). Subsequently, we evaluated the growth of the strains by measuring the dried biomass on each cultivation day. As a result, we observed rapid growth from FCd-2 (0.34 g) to FCd-6 (0.93 g), followed by a plateau phase where the biomass ranged from 0.88 g to 0.95 g (Fig. 5C). Based on these findings, we inferred that the rapid growth of fungal hyphae in the early phase resulted in the copious generation of ROS, which in turn led to laccase overproduction but concurrently suppressed the expression of TCA cycle genes at FCd-6 [46]. The repression of the TCA cycle genes was then alleviated during the subsequent growth phases. Meanwhile, the repression of the TCA cycle led to a 2.93-fold increase in the accumulation of the biomarker – succinate at FCd-6 compared to that at FCd-10 (Fig. 5A, D). Previous omics studies of *T. gibbose* have reported the importance of succinate dehydrogenase in laccase production, and identified fluctuations in the abundance of succinate under lignin stress, a known inducer of laccase production [42, 47]. These studies suggested a potential relationship between succinate and laccase, but the specific role of succinate in laccase production had not been explored yet. In this study, we, for the first time, examined the effect of succinate by adding different concentrations of this biomarker to the cultivation media. As shown in Fig. 5E, an increase in laccase production was observed in line with the rising concentration of added succinate, indicating a dose-dependent positive impact of succinate on laccase production. In the yeast *Xanthophyllomyces dendrorhous*, succinate was verified to increase the production of ROS through oxidative respiration [48]. Meanwhile, increased levels of ROS could act as signaling molecules to stimulate the overexpression of laccase [49]. Accordingly, supplemented succinate globally increased the transcript level of the laccase gene family in *C. unicolor* 87613 (Fig. S3). More interestingly, the overexpression level of *lac1*, *2*, *5*, *9*, *17/18* were strictly and positively correlated with the increasing concentration of added succinate (Fig. S3), suggesting a potential mechanism by which succinate acted dose-dependently on laccase production.

In summary, *C. unicolor* 87613 metabolizes fructose to undergo rapid mycelial growth, leading to the accumulation of ROS; these ROS feedback suppresses the transcription activity of the TCA cycle, thereby causing the increase of succinate abundance; together with the accumulated ROS, the increased succinate further stimulates the overexpression of laccase in *C. unicolor* 87613.

### Cysteine and methionine metabolism and its biomarkers involved in laccase production

**Fig. 6:**
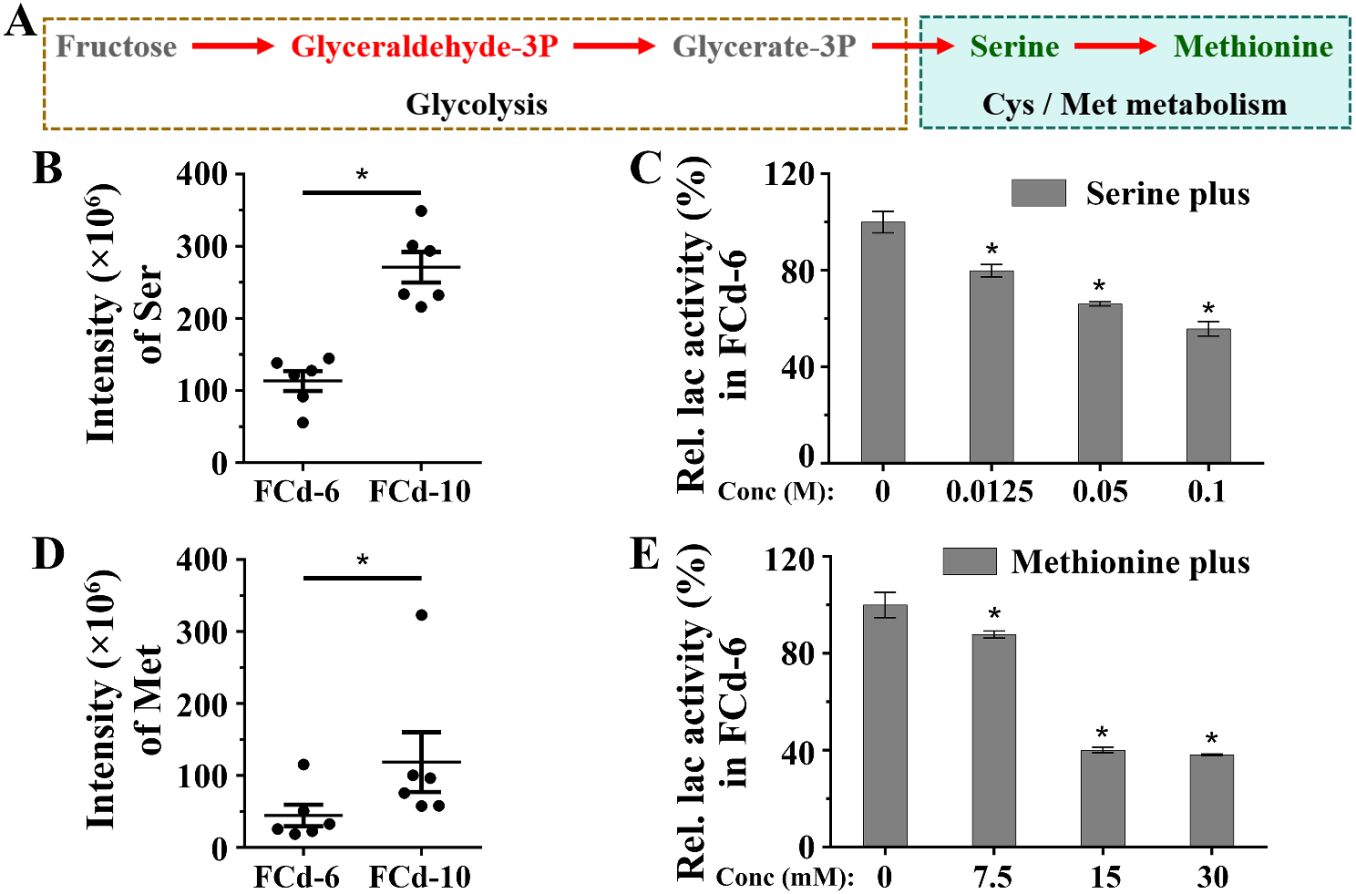
The alteration of **A** cysteine and methionine metabolism pathway in Cd-6 compared to those FCd-10 as cultivated with fructose. **B** the intensity of serine (Ser) measured by LC-MS.MS at FCd-6 and FCd-10 periods. **C** the dose-dependent repression of laccase production by additive serine during *C. unicolor* 87613 cultivation. **D** the intensity of methionine (Met) measured by LC-MS.MS at FCd-6 and FCd-10 periods. **E** the dose-dependent repression of laccase production by additive methionine during *C. unicolor* 87613 cultivation

In addition to carbon metabolism, amino acid metabolism emerged as a significant metabolic network, simultaneously enriched by both DEGs and DAMs. Within this network, the cysteine and methionine metabolism, downstream of glycolysis, exhibited a pronounced up-regulation in its transcription profile (Fig. 6A and Fig. S2). Notably, the serine biomarker was reduced by 58% in FCd-6 compared to FCd-10 (Fig. 6B). Cultures supplemented with additional serine exhibited a dose-dependent decrease in laccase production (Fig. 6C). Although there is still no evidence to explain how serine impacts fungal laccase production, previous studies might provide some clues. For instance, a metabolite – β-carotene was demonstrated to stimulate laccase production by modulating the transcript factors TFb, TFc and TFd in *Pleurotus eryngii* var. *ferulae* [50]. Moreover, Gan and collaborators uncovered a novel role for seryl-tRNA synthetase (TherRS) as a TF in the repression of laccase production through its interaction with the promoter region of the laccase gene in *T. hisuta* AH28-2 [51]. These findings suggest a potential mechanism by which serine might regulate laccase production through interaction with TFs, such as TherRS. Thereafter, we were inspired to investigate the expression of TherRS in *C. unicolor* 87613. Unfortunately, the coding genes for ThserRS (A01192 and A07904) did not show significant variation between FCd-6 and FCd-10 (Fig. S4). Despite the lack of change in ThserRS expression levels, serine in *C. unicolor* 87613 did show the same negative impact on laccase production as TherRS reported in *T. hisuta* AH28-2 [51]. Hence, it remains a possible way to negatively influence fungal laccase production through the interaction between serine and TherRS (or other TFs). This hypothesis, however, requires further experimental validation. Nevertheless, we still found that additional serine could globally repressed the transcript levels of laccase gene family (Fig. S5). The transcriptional repression levels of each laccase gene were not negatively correlated with the increasing concentration of supplemented serine, except for *lac2*, *5*, *14*, *16*, *17/18* (Fig. S5), suggesting that serine might impact on laccase production via transcriptional regulation and other potential mechanisms. For example, both earlier studies and our metabolic-network analysis (Fig S2) revealed the precursor role of serine in synthesis of reduced glutathione (GSH) [52, 53], which was previously demonstrated to dose-dependently repress the laccase production in *C. unicolor* 87613 [41].

Moreover, we observed a 54% reduction in methionine in FCd-6 compared to FCd-10 (Fig. 6D). The negative impact of methionine on laccase production was confirmed by its supplementation, which resulted in a 22.2% decrease in laccase activity at 7.5 mM methionine, worsening to a 40.1% reduction at 15 mM methionine (Fig. 6E). Although previous studies identified the methionine residue as crucial for the redox potential of the laccase T1 copper [32], an important indicator of the catalytic properties of laccase [54, 55], our research revealed the negative effect of free methionine on laccase production.

In summary, the metabolism of cysteine and methionine played a critical role in regulating laccase production in *C. unicolor* 87613; the transcriptional up-regulation of this pathway potentially served as a response to the reduction of serine and methionine; and the intracellular contents of both serine and methionine negatively regulated laccase production in *C. unicolor* 87613. To the best of our knowledge, this is the first demonstration of the dose-dependently inhibitory impact of serine and methionine on fungal laccase production. However, their regulatory mechanisms require further investigation.

### Glutathione metabolism and its biomarkers in regulating laccase production

**Fig. 7:**
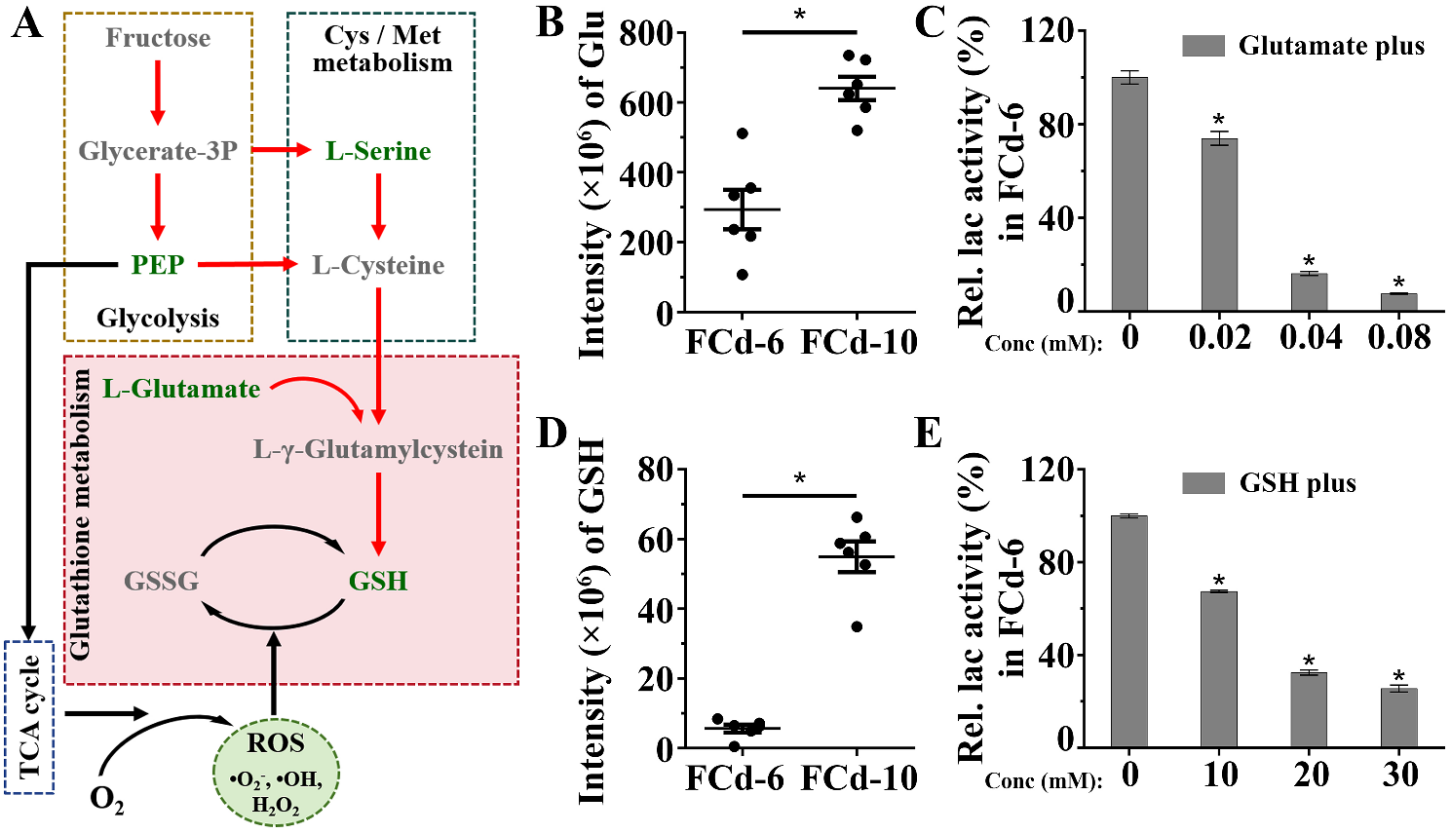
The alteration of **A** glutathione metabolism pathway in Cd-6 compared to those FCd-10 as cultivated with fructose. **B** the intensity of glutamate (Glu) measured by LC-MS.MS at FCd-6 and FCd-10 periods. **C** the dose-dependent repression of laccase production by additive glutamate during *C. unicolor* 87613 cultivation. **D** the intensity of reduced glutathione (GSH) measured by LC-MS.MS at FCd-6 and FCd-10 periods. **E** the dose-dependent repression of laccase production by additive GSH during *C. unicolor* 87613 cultivation

Analysis of pathways under both glucose- [41] and fructose-cultivated conditions pinpointed the importance of glutathione metabolism (Table S8). Moreover, glutathione metabolism acts as the downstream of cysteine and methionine metabolism and the TCA cycle (Fig. 7A). Thus, glutathione metabolism might be pivotal for regulating laccase production in *C. unicolor* 87613. Being similar to cysteine and methionine metabolism, glutathione metabolism showed contrasting changes between transcriptional and metabolic levels in FCd-6 compared to FCd-10. As indicated by metabolomic analysis, the level of glutamate within the glutathione metabolism pathway was reduced by 54% in FCd-6 compared to FCd-10 (Fig. 7B). Experimental evidence confirmed the negative dose-dependent effects of glutamate on *C. unicolor* 87613 laccase production, with only 7.7% retention of laccase activity in the presence of 0.08 mM glutamate (Fig. 7C). Controversially, a previous study demonstrated that glutamate was the best-defined nitrogen source for laccase production by *T. trogii* and *T. villosa* [56]. We speculated that the effect of glutamate on laccase production varies depending on the species.

In addition to glutamate, GSH, the core biomarker within glutathione metabolism, show differential abundance between FCd-6 and FCd-10. A 54% reduction in GSH was observed in FCd-6 compared to FCd-10 (Fig. 7D). Similarly, GSH exhibited a dose-dependent repressive impact on laccase production (Fig. 7E). The reduction in laccase production was about 74.5% in cultures supplemented with 30 mM GSH. Importantly, compelling evidence shows a counterbalancing role of GSH against ROS in maintaining fungal redox homeostasis [57, 58]. Therefore, we measured the intracellular levels of both reduced and oxidized glutathione (GSH and GSSG). As a result, the GSH levels were 28.9% lower in FCd-6 compared to FCd-10, while the GSSG amounts were 1.6 times higher in FCd-6 (Fig. S6). Given the indicative role of the GSH/GSSG balance in intracellular redox states [59], we further calculated their ratio and found a significant shift from a value of 1.1 in FCd-6 to 2.6 in FCd-10 (Fig. S6), suggesting an increase in oxidative stress in FCd-6 hyphal cells. The observation aligns with our findings on ROS levels above (Fig. 5B). Accordingly, we proposed that the reduction of GSH in FCd-6 contributed to higher oxidative stress, which facilitated laccase production [43, 44]. Notably, glutathione is composed of glutamate, cysteine, and glycine [60]. Increasing metabolic substrates, such as glutamate, in the glutathione metabolic pathway might promote the generation of GSH, which negatively affects laccase production. This deduction could explain the way that glutamate negatively impacted laccase production in *C. unicolor* 87613.

Collectively, these findings unveil the significance of glutathione metabolism as a critical downstream pathway of the complex metabolic network; the core biomarker – GSH serves as a balancing agent against ROS by-produced by the TCA cycle, thereby influencing laccase production through alterations of intracellular redox homeostasis.

### Comparison of abundant changes of metabolic biomarkers by glucose- and fructose-induction

**Fig. 8:**
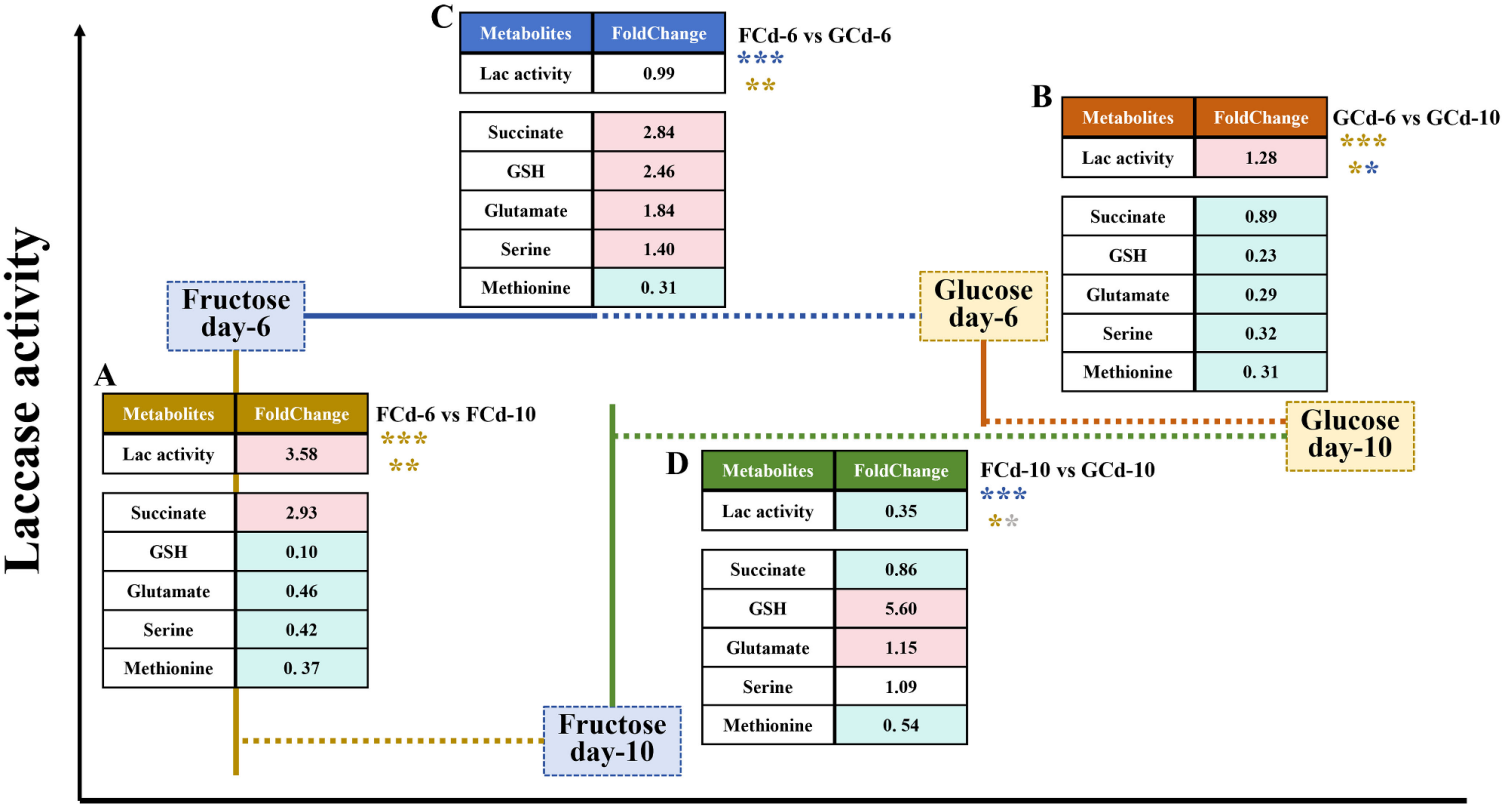
The illustration of altered laccase production and corresponding intracellular abundance of five metabolic biomarkers (succinate, GSH, glutamate, serine, and methionine) in **A** FCd-6 versus FCd-10, **B** GCd-6 versus GCd-10, **C** FCd-6 versus GCd-6, and **D** FCd-10 versus GCd-10, respectively. The yellow asterisk indicates a promoting effect on laccase activity, the blue asterisk suggests an inhibitory effect on laccase activity, and the gray asterisk indicates on effect on laccase activity

Transcriptomic and metabolomic analysis revealed the positive influence of succinate and negative impacts of three amino acids (serine, methionine, glutamate) and one derivative (GSH) on laccase production in *C. unicolor* 87613, when cultivated with fructose. It is curious to analyze the antagonistic and/or synergistic effects of these metabolites. Therefore, their patterns were estimated in both fructose- and glucose-cultivated samples (referred to as FCd and GCd). Consequently, the changes in all five metabolic biomarkers positively affected laccase production, resulting in a 3.58-fold increase in FCd-6 compared to FCd-10 (Fig. 8A). Interestingly, the changes in these metabolites under glucose conditions resulted in four positive effects and one negative impact on laccase production, which led to a less significant difference in laccase activity (1.28 times in GCd-6 versus those in GCd-10) (Fig. 8B). The fold-change in laccase production decreased further (0.99 times) when comparing FCd-6 to GCd-6, as metabolic biomarkers showed mutual cancellation effects with two positive and three negative shifts (Fig. 8C). More importantly, the comparison between FCd-10 and GCd-10 revealed only one positive and three negative shifts in the levels of five metabolites, leading to a reverse increase in laccase production difference (Fig. 8D). The comparative analysis suggests that an increased proportion of positive changes in these five metabolites might boost the production level of laccase. A greater disparity between their positive and negative changes might cause larger fluctuations in laccase production. In addition, the specific abundance levels of these metabolites might also play a role in influencing laccase production.

## Conclusion

The production of laccase by most white-rot fungi (WRF) has not yet met industrial-scale demands, with laccase production in some WRF occurring during secondary metabolism and might exhibiting limited sustainability [18, 28, 61]. This study utilized both transcriptomic and metabolomic approaches to identify key pathways that determine the production of laccase in significant quantities. Our combined analysis highlighted the positive contributions of succinate within carbon metabolism, and the negative roles of amino acid metabolism (primarily involving serine, methionine, glutamate and GSH) in regulating *C. unicolor* 87613 laccase productivity. More importantly, these metabolites could interact with each other within the complex metabolic network. These findings not only offer a fresh perspective on the regulatory mechanisms of laccase production influenced by varying carbon sources, but also provide a promising framework for enhancing fungal laccase production by modulating the related metabolic network.

### Electronic supplementary material

Below is the link to the electronic supplementary material.

Supplementary Material 1

Supplementary Material 2

Supplementary Material 3

Supplementary Material 4

Supplementary Material 5

Supplementary Material 6

Supplementary Material 7

Supplementary Material 8

Supplementary Material 9

## Data Availability

All data used in this study are available upon personal request to the authors. Among those, the raw high throughput sequencing data of RNA sequencing have been deposited in the NCBI Sequence Read Archive (SRA) under the GEO accession number GSE236542.
