# Correction: A complex metabolic network and its biomarkers regulate laccase production in white-rot fungus *Cerrena unicolor* 87613

**DOI:** 10.1186/s12934-024-02458-2

**Published:** 2024-07-15

**Authors:** Long-Bin Zhang, Xiu-Gen Qiu, Ting-Ting Qiu, Zhou Cui, Yan Zheng, Chun Meng

**Affiliations:** 1 College of Biological Science and Engineering, Fuzhou University, Fuzhou, Fujian 350108 China; 2 The Key Laboratory of Marine Enzyme Engineering of Fujian Province, Fuzhou University, Fuzhou, Fujian 350108 China

**Correction: Microbial Cell Factories 2024: (23)167**

10.1186/s12934-024-02443-9.

Following publication of the original article, the authors identified an error in the author name of Xiu-Gen Qiu.

The incorrect author name is: Xiu-Gen Xiu

The correct author name is: Xiu-Gen Qiu

The author group has been updated above and the original article has been corrected.

