# Supplementary material for: A complex metabolic network and its biomarkers regulate laccase production in white-rot fungus Cerrena unicolor 87613
Source: Microb Cell Fact. 2024 Jun 8;23:167. doi: 10.1186/s12934-024-02443-9 (PMC11162070; doi:10.1186/s12934-024-02443-9)
Supplement: Supplementary file 1 — Supplementary Material 1 [file 12934_2024_2443_MOESM1_ESM.docx]

**Table S3 Enrichment analysis of Gene Ontology (GO) assignments for the differentially expressed genes (DEGs) of FCd-6 samples versus FCd-10 samples.**

| **Change** | **Category*** | **GO ID** | **Terms** | ***P*-value** | **Gene Counts** | **Gene IDs** |
| --- | --- | --- | --- | --- | --- | --- |
| **Up** | BP | GO:0055085 | transmembrane transport | 0.001 | 52 | A07141.gene/A03912.gene/A00814.gene/A01531.gene/A03189.gene/A02617.gene/A08685.gene/A09012.gene/A10149.gene/A00699.gene/A09118.gene/A07547.gene/A04492.gene/A04383.gene/A01790.gene/A03958.gene/A03752.gene/A00613.gene/A10157.gene/A06525.gene/A07048.gene/A06872.gene/A00508.gene/A02961.gene/A09747.gene/A10196.gene/A02588.gene/A03902.gene/A04250.gene/A00583.gene/A02778.gene/A08994.gene/A05048.gene/A10150.gene/A09843.gene/A01699.gene/A08566.gene/A02037.gene/A04421.gene/A06162.gene/A07357.gene/A03937.gene/A04441.gene/A01869.gene/A07030.gene/A03929.gene/A05357.gene/A01416.gene/A08565.gene/A07719.gene/A00019.gene/A02170.gene |
|  | BP | GO:0006720 | isoprenoid metabolic process | 0.000 | 7 | A08991.gene/A08993.gene/A07924.gene/A09077.gene/A07925.gene/A09002.gene/A10171.gene |
|  | BP | GO:0008299 | isoprenoid biosynthetic process | 0.000 | 7 | A08991.gene/A08993.gene/A07924.gene/A09077.gene/A07925.gene/A09002.gene/A10171.gene |
|  | BP | GO:0016052 | carbohydrate catabolic process | 0.000 | 7 | A09661.gene/A06836.gene/A06115.gene/A03279.gene/A06837.gene/A06380.gene/A09662.gene |
|  | BP | GO:0046700 | heterocycle catabolic process | 0.003 | 7 | A09251.gene/A03451.gene/A03279.gene/A03209.gene/A06380.gene/A02397.gene/A00416.gene |
|  | BP | GO:0034655 | nucleobase-containing compound catabolic process | 0.003 | 6 | A03451.gene/A03279.gene/A03209.gene/A06380.gene/A02397.gene/A00416.gene |
|  | BP | GO:0006714 | sesquiterpenoid metabolic process | 0.001 | 5 | A08991.gene/A08993.gene/A07924.gene/A07925.gene/A09002.gene |
|  | BP | GO:0006721 | terpenoid metabolic process | 0.001 | 5 | A08991.gene/A08993.gene/A07924.gene/A07925.gene/A09002.gene |
|  | BP | GO:0009166 | nucleotide catabolic process | 0.001 | 5 | A03451.gene/A03279.gene/A03209.gene/A06380.gene/A00416.gene |
|  | BP | GO:0016106 | sesquiterpenoid biosynthetic process | 0.001 | 5 | A08991.gene/A08993.gene/A07924.gene/A07925.gene/A09002.gene |
|  | BP | GO:0016114 | terpenoid biosynthetic process | 0.001 | 5 | A08991.gene/A08993.gene/A07924.gene/A07925.gene/A09002.gene |
|  | BP | GO:1901292 | nucleoside phosphate catabolic process | 0.001 | 5 | A03451.gene/A03279.gene/A03209.gene/A06380.gene/A00416.gene |
|  | CC | GO:0016021 | integral component of membrane | 0.000 | 53 | A07141.gene/A08248.gene/A03912.gene/A00814.gene/A01531.gene/A03189.gene/A01068.gene/A07967.gene/A02617.gene/A08685.gene/A06402.gene/A09012.gene/A10149.gene/A09880.gene/A00662.gene/A02764.gene/A00699.gene/A09118.gene/A07547.gene/A04383.gene/A03958.gene/A03752.gene/A00613.gene/A10157.gene/A06525.gene/A07048.gene/A06872.gene/A00508.gene/A02961.gene/A02588.gene/A03902.gene/A04250.gene/A02778.gene/A08994.gene/A05048.gene/A10150.gene/A01699.gene/A08566.gene/A03941.gene/A04421.gene/A06162.gene/A03202.gene/A03937.gene/A04441.gene/A01869.gene/A07030.gene/A03929.gene/A05357.gene/A01416.gene/A07719.gene/A00664.gene/A00019.gene/A02170.gene |
|  | CC | GO:0031224 | intrinsic component of membrane | 0.000 | 53 | A07141.gene/A08248.gene/A03912.gene/A00814.gene/A01531.gene/A03189.gene/A01068.gene/A07967.gene/A02617.gene/A08685.gene/A06402.gene/A09012.gene/A10149.gene/A09880.gene/A00662.gene/A02764.gene/A00699.gene/A09118.gene/A07547.gene/A04383.gene/A03958.gene/A03752.gene/A00613.gene/A10157.gene/A06525.gene/A07048.gene/A06872.gene/A00508.gene/A02961.gene/A02588.gene/A03902.gene/A04250.gene/A02778.gene/A08994.gene/A05048.gene/A10150.gene/A01699.gene/A08566.gene/A03941.gene/A04421.gene/A06162.gene/A03202.gene/A03937.gene/A04441.gene/A01869.gene/A07030.gene/A03929.gene/A05357.gene/A01416.gene/A07719.gene/A00664.gene/A00019.gene/A02170.gene |
|  | CC | GO:0044425 | membrane part | 0.000 | 53 | A07141.gene/A08248.gene/A03912.gene/A00814.gene/A01531.gene/A03189.gene/A01068.gene/A07967.gene/A02617.gene/A08685.gene/A06402.gene/A09012.gene/A10149.gene/A09880.gene/A00662.gene/A02764.gene/A00699.gene/A09118.gene/A07547.gene/A04383.gene/A03958.gene/A03752.gene/A00613.gene/A10157.gene/A06525.gene/A07048.gene/A06872.gene/A00508.gene/A02961.gene/A02588.gene/A03902.gene/A04250.gene/A02778.gene/A08994.gene/A05048.gene/A10150.gene/A01699.gene/A08566.gene/A03941.gene/A04421.gene/A06162.gene/A03202.gene/A03937.gene/A04441.gene/A01869.gene/A07030.gene/A03929.gene/A05357.gene/A01416.gene/A07719.gene/A00664.gene/A00019.gene/A02170.gene |
|  | MF | GO:0048037 | cofactor binding | 0.000 | 70 | A08062.gene/A07571.gene/A04402.gene/A03210.gene/A03665.gene/A00661.gene/A09941.gene/A02613.gene/A06660.gene/A00692.gene/A07547.gene/A07224.gene/A03947.gene/A06229.gene/A07254.gene/A00222.gene/A02505.gene/A07354.gene/novel.1493/A00555.gene/A09856.gene/A06840.gene/A04617.gene/A09665.gene/A07914.gene/A04426.gene/A09141.gene/A06088.gene/A07284.gene/A07369.gene/A04901.gene/A08032.gene/A06760.gene/A07322.gene/A00923.gene/A04499.gene/A09846.gene/A01137.gene/A06350.gene/A03646.gene/A08064.gene/A08574.gene/A01256.gene/A02289.gene/A09087.gene/A04442.gene/A07010.gene/A04500.gene/A08670.gene/A00187.gene/A07917.gene/A07919.gene/A00229.gene/A00420.gene/A02719.gene/novel.1433/A00740.gene/A01230.gene/A02624.gene/A04210.gene/A09177.gene/A06552.gene/A10153.gene/A03309.gene/A08240.gene/A07605.gene/A03458.gene/A03012.gene/A06553.gene/A06295.gene |
|  | MF | GO:0050662 | coenzyme binding | 0.000 | 37 | A08062.gene/A07571.gene/A04402.gene/A03210.gene/A00661.gene/A02613.gene/A06660.gene/A00692.gene/A03947.gene/A06229.gene/A02505.gene/novel.1493/A00555.gene/A09856.gene/A06840.gene/A04617.gene/A09665.gene/A07914.gene/A04426.gene/A09141.gene/A07369.gene/A04901.gene/A06760.gene/A04499.gene/A06350.gene/A08064.gene/A08574.gene/A01256.gene/A04442.gene/A00187.gene/A07917.gene/A00229.gene/A02719.gene/A02624.gene/A09177.gene/A03309.gene/A03012.gene |
|  | MF | GO:0005506 | iron ion binding | 0.000 | 32 | A03665.gene/A09941.gene/A07547.gene/A07224.gene/A07254.gene/A00222.gene/A07354.gene/A06757.gene/A02450.gene/A07284.gene/A08032.gene/A07322.gene/A00923.gene/A09846.gene/A01137.gene/A09087.gene/A07557.gene/A07010.gene/A04500.gene/A08670.gene/A07919.gene/A00420.gene/A00740.gene/A01230.gene/A04210.gene/A06552.gene/A10153.gene/A08240.gene/A07605.gene/A03458.gene/A06553.gene/A06295.gene |
|  | MF | GO:0016705 | oxidoreductase activity, acting on paired donors, with incorporation or reduction of molecular oxygen | 0.000 | 30 | A03665.gene/A09941.gene/A07547.gene/A07224.gene/A07254.gene/A00222.gene/A07354.gene/A09251.gene/A07284.gene/A08032.gene/A07322.gene/A00923.gene/A09846.gene/A01137.gene/A09087.gene/A07010.gene/A04500.gene/A08670.gene/A07919.gene/A00420.gene/A00740.gene/A01230.gene/A04210.gene/A06552.gene/A10153.gene/A08240.gene/A07605.gene/A03458.gene/A06553.gene/A06295.gene |
|  | MF | GO:0020037 | heme binding | 0.002 | 30 | A03665.gene/A09941.gene/A07547.gene/A07224.gene/A07254.gene/A00222.gene/A07354.gene/A07284.gene/A08032.gene/A07322.gene/A00923.gene/A09846.gene/A01137.gene/A03646.gene/A09087.gene/A07010.gene/A04500.gene/A08670.gene/A07919.gene/A00420.gene/A00740.gene/A01230.gene/A04210.gene/A06552.gene/A10153.gene/A08240.gene/A07605.gene/A03458.gene/A06553.gene/A06295.gene |
|  | MF | GO:0046906 | tetrapyrrole binding | 0.002 | 30 | A03665.gene/A09941.gene/A07547.gene/A07224.gene/A07254.gene/A00222.gene/A07354.gene/A07284.gene/A08032.gene/A07322.gene/A00923.gene/A09846.gene/A01137.gene/A03646.gene/A09087.gene/A07010.gene/A04500.gene/A08670.gene/A07919.gene/A00420.gene/A00740.gene/A01230.gene/A04210.gene/A06552.gene/A10153.gene/A08240.gene/A07605.gene/A03458.gene/A06553.gene/A06295.gene |
|  | MF | GO:0016614 | oxidoreductase activity, acting on CH-OH group of donors | 0.000 | 19 | A03210.gene/A07430.gene/A02613.gene/A00692.gene/A04730.gene/novel.1493/A00555.gene/A06840.gene/A04617.gene/A09665.gene/A07914.gene/A09141.gene/A07369.gene/A04901.gene/A04499.gene/A06979.gene/novel.610/A01256.gene/A02502.gene |
|  | MF | GO:0050660 | flavin adenine dinucleotide binding | 0.007 | 14 | A04402.gene/A03210.gene/A02613.gene/A00692.gene/A00555.gene/A06840.gene/A09665.gene/A07914.gene/A07369.gene/A04901.gene/A04499.gene/A01256.gene/A02719.gene/A09177.gene |
|  | MF | GO:0010181 | FMN binding | 0.000 | 10 | A00661.gene/A04426.gene/A06350.gene/A08574.gene/A04442.gene/A00187.gene/A07917.gene/A02624.gene/A03309.gene/A03012.gene |
|  | MF | GO:0016835 | carbon-oxygen lyase activity | 0.001 | 10 | A08991.gene/A08993.gene/A07924.gene/A01532.gene/A07925.gene/A09002.gene/A06762.gene/A07913.gene/A01522.gene/A08695.gene |
|  | MF | GO:0005507 | copper ion binding | 0.000 | 9 | A08410.gene/A08247.gene/A07756.gene/A00709.gene/A07743.gene/A00710.gene/A06088.gene/A08365.gene/novel.1433 |
|  | MF | GO:0010333 | terpene synthase activity | 0.000 | 9 | A08991.gene/A08993.gene/A07924.gene/A01532.gene/A07925.gene/A09002.gene/A06762.gene/A07913.gene/A01522.gene |
|  | MF | GO:0016838 | carbon-oxygen lyase activity, acting on phosphates | 0.000 | 9 | A08991.gene/A08993.gene/A07924.gene/A01532.gene/A07925.gene/A09002.gene/A06762.gene/A07913.gene/A01522.gene |
|  | MF | GO:0005509 | calcium ion binding | 0.002 | 7 | A09661.gene/A00942.gene/A00951.gene/A06836.gene/A06115.gene/A06837.gene/A09662.gene |
|  | MF | GO:0010334 | sesquiterpene synthase activity | 0.001 | 5 | A08991.gene/A08993.gene/A07924.gene/A07925.gene/A09002.gene |
|  | MF | GO:0045482 | trichodiene synthase activity | 0.001 | 5 | A08991.gene/A08993.gene/A07924.gene/A07925.gene/A09002.gene |
|  | MF | GO:0004866 | endopeptidase inhibitor activity | 0.002 | 5 | A00789.gene/A03837.gene/A01764.gene/A00801.gene/A00795.gene |
|  | MF | GO:0030414 | peptidase inhibitor activity | 0.002 | 5 | A00789.gene/A03837.gene/A01764.gene/A00801.gene/A00795.gene |
|  | MF | GO:0061134 | peptidase regulator activity | 0.002 | 5 | A00789.gene/A03837.gene/A01764.gene/A00801.gene/A00795.gene |
|  | MF | GO:0061135 | endopeptidase regulator activity | 0.002 | 5 | A00789.gene/A03837.gene/A01764.gene/A00801.gene/A00795.gene |
|  | MF | GO:0004185 | serine-type carboxypeptidase activity | 0.005 | 5 | A10216.gene/A08286.gene/A09278.gene/A10229.gene/A09280.gene |
|  | MF | GO:0004180 | carboxypeptidase activity | 0.007 | 5 | A10216.gene/A08286.gene/A09278.gene/A10229.gene/A09280.gene |
|  | MF | GO:0004857 | enzyme inhibitor activity | 0.007 | 5 | A00789.gene/A03837.gene/A01764.gene/A00801.gene/A00795.gene |
|  | MF | GO:0070008 | serine-type exopeptidase activity | 0.007 | 5 | A10216.gene/A08286.gene/A09278.gene/A10229.gene/A09280.gene |
|  | MF | GO:0004867 | serine-type endopeptidase inhibitor activity | 0.006 | 4 | A00789.gene/A01764.gene/A00801.gene/A00795.gene |
| **Down** | BP | GO:0005975 | carbohydrate metabolic process | 0.000 | 62 | A08202.gene/A03096.gene/A02384.gene/A07290.gene/A03683.gene/A02402.gene/A07830.gene/A00532.gene/A10087.gene/A07831.gene/-/A07741.gene/A02821.gene/A01247.gene/A09461.gene/A01954.gene/A08879.gene/A04824.gene/A07949.gene/A00342.gene/A03971.gene/A09771.gene/A01792.gene/A04336.gene/A00713.gene/A01650.gene/A00537.gene/A00980.gene/A06361.gene/A03487.gene/A00706.gene/A06807.gene/A02124.gene/A04878.gene/A05680.gene/A03885.gene/A01658.gene/A07715.gene/A09587.gene/A09462.gene/A01293.gene/A02839.gene/A07231.gene/A04030.gene/A04855.gene/A06736.gene/A04655.gene/A03535.gene/A00979.gene/A06517.gene/A07278.gene/A02849.gene/A02001.gene/A08188.gene/A04856.gene/A00542.gene/A01315.gene/A09048.gene/A06079.gene/A05970.gene/A01858.gene/A02750.gene |
|  | CC | GO:0005576 | extracellular region | 0.000 | 22 | A02745.gene/A02021.gene/A07830.gene/A07831.gene/A01954.gene/A04824.gene/A01792.gene/A00713.gene/A01650.gene/A00537.gene/A01467.gene/A01466.gene/A03885.gene/A01293.gene/A04855.gene/A06736.gene/A03535.gene/A02001.gene/A04856.gene/A00755.gene/A05970.gene/A02750.gene |
|  | CC | GO:0071944 | cell periphery | 0.000 | 15 | A01089.gene/A10056.gene/A06328.gene/A03602.gene/A03604.gene/A00365.gene/A01088.gene/A01090.gene/A01091.gene/A06327.gene/A03607.gene/A04541.gene/A04482.gene/A07536.gene/A10121.gene |
|  | CC | GO:0005618 | cell wall | 0.000 | 13 | A01089.gene/A10056.gene/A06328.gene/A03602.gene/A03604.gene/A01088.gene/A01090.gene/A01091.gene/A06327.gene/A03607.gene/A04541.gene/A04482.gene/A07536.gene |
|  | CC | GO:0030312 | external encapsulating structure | 0.000 | 13 | A01089.gene/A10056.gene/A06328.gene/A03602.gene/A03604.gene/A01088.gene/A01090.gene/A01091.gene/A06327.gene/A03607.gene/A04541.gene/A04482.gene/A07536.gene |
|  | CC | GO:0009277 | fungal-type cell wall | 0.000 | 12 | A01089.gene/A06328.gene/A03602.gene/A03604.gene/A01088.gene/A01090.gene/A01091.gene/A06327.gene/A03607.gene/A04541.gene/A04482.gene/A07536.gene |
|  | MF | GO:0004553 | hydrolase activity, hydrolyzing O-glycosyl compounds | 0.000 | 52 | A08202.gene/A03096.gene/A02384.gene/A07290.gene/A02402.gene/A07830.gene/A05393.gene/A09857.gene/A10087.gene/A07831.gene/-/A07741.gene/A02821.gene/A09461.gene/A01954.gene/A08879.gene/A04824.gene/A07949.gene/A03971.gene/A01792.gene/A04336.gene/A00713.gene/A03917.gene/A01650.gene/A00537.gene/A00980.gene/A06361.gene/A10000.gene/A03487.gene/A00706.gene/A06807.gene/A05680.gene/A03885.gene/A09587.gene/A09462.gene/A01293.gene/A07231.gene/A09971.gene/A04030.gene/A06736.gene/A04655.gene/A03535.gene/A00979.gene/A07278.gene/A02849.gene/A02001.gene/A08188.gene/A01315.gene/A09048.gene/A06079.gene/A05970.gene/A02750.gene |
|  | MF | GO:0016798 | hydrolase activity, acting on glycosyl bonds | 0.000 | 52 | A08202.gene/A03096.gene/A02384.gene/A07290.gene/A02402.gene/A07830.gene/A05393.gene/A09857.gene/A10087.gene/A07831.gene/-/A07741.gene/A02821.gene/A09461.gene/A01954.gene/A08879.gene/A04824.gene/A07949.gene/A03971.gene/A01792.gene/A04336.gene/A00713.gene/A03917.gene/A01650.gene/A00537.gene/A00980.gene/A06361.gene/A10000.gene/A03487.gene/A00706.gene/A06807.gene/A05680.gene/A03885.gene/A09587.gene/A09462.gene/A01293.gene/A07231.gene/A09971.gene/A04030.gene/A06736.gene/A04655.gene/A03535.gene/A00979.gene/A07278.gene/A02849.gene/A02001.gene/A08188.gene/A01315.gene/A09048.gene/A06079.gene/A05970.gene/A02750.gene |
|  | MF | GO:0030246 | carbohydrate binding | 0.000 | 27 | A03712.gene/A07830.gene/A00532.gene/A07831.gene/A07741.gene/A01954.gene/A08879.gene/A04824.gene/A01792.gene/A04336.gene/A00713.gene/A01650.gene/A00537.gene/A02124.gene/A04878.gene/A03885.gene/A01293.gene/A04855.gene/A06736.gene/A03535.gene/A06517.gene/A02001.gene/A04856.gene/A00542.gene/A05970.gene/A01858.gene/A02750.gene |
|  | MF | GO:0001871 | pattern binding | 0.000 | 16 | A03712.gene/A07830.gene/A00532.gene/A07831.gene/A01954.gene/A04824.gene/A01792.gene/A00713.gene/A03885.gene/A01293.gene/A06736.gene/A03535.gene/A02001.gene/A00542.gene/A05970.gene/A02750.gene |
|  | MF | GO:0030247 | polysaccharide binding | 0.000 | 16 | A03712.gene/A07830.gene/A00532.gene/A07831.gene/A01954.gene/A04824.gene/A01792.gene/A00713.gene/A03885.gene/A01293.gene/A06736.gene/A03535.gene/A02001.gene/A00542.gene/A05970.gene/A02750.gene |
|  | MF | GO:0030248 | cellulose binding | 0.000 | 13 | A07830.gene/A07831.gene/A01954.gene/A04824.gene/A01792.gene/A00713.gene/A03885.gene/A01293.gene/A06736.gene/A03535.gene/A02001.gene/A05970.gene/A02750.gene |
|  | MF | GO:0005199 | structural constituent of cell wall | 0.000 | 12 | A01089.gene/A06328.gene/A03602.gene/A03604.gene/A01088.gene/A01090.gene/A01091.gene/A06327.gene/A03607.gene/A04541.gene/A04482.gene/A07536.gene |

***BP, CC and MF are shorted for Biological Process, Cellular Component, and Molecular Function, respectively.**
