# Supplementary material for: A complex metabolic network and its biomarkers regulate laccase production in white-rot fungus Cerrena unicolor 87613
Source: Microb Cell Fact. 2024 Jun 8;23:167. doi: 10.1186/s12934-024-02443-9 (PMC11162070; doi:10.1186/s12934-024-02443-9)
Supplement: Supplementary file 2 — Supplementary Material 2 [file 12934_2024_2443_MOESM2_ESM.docx]

**Table S1 Paired primers used for transcriptional profiling of laccase gene family (Cu*Lac*) in *C.unicolor* 87613.**

| **Primers** | **Sequences (5’ → 3’)** |
| --- | --- |
| Cu*Lac1-*F / R | TTACCTTCTCCATTGATG / GTCCAGTGTTGTTGTATT |
| Cu*Lac2-*F / R | CCGTTGATGCTGCGAATG / TTGTAAGTAAGGAGTGTGAGGTAG |
| Cu*Lac3-*F / R | CATCTCTTGGTCTTGTCT / TACTCTGTAGGCATTGTG |
| Cu*Lac4-*F / R | AGGTCAACGATATTCATT / GTAATATAGCGGAGTTCA |
| Cu*Lac5-*F / R | CCTGGAGCACTTATCACT / ACATACTGGCATCGGTAA |
| Cu*Lac6-*F / R | TAGGATGGATAGATGTGAAC / CAATATGGCGAGACTGTA |
| Cu*Lac7-*F / R | GGTCCTCTTGTTGTGTAT / GGTGATAACGGTAGTCTC |
| Cu*Lac8-*F / R | TATGACCATTATTGAAGTTG / ACGAATCCAGTAGTTATC |
| Cu*Lac9-*F / R | TTGAAGTGGATAGTGTTA / TTGATACCATTGTTGAAG |
| Cu*Lac10-*F / R | CTCTGTCATTAGCGTTCA / TCATATTATGGTTGTCAATC |
| Cu*Lac11-*F / R | TCGTGCTCTTAGTGCTTA / TCCGTGGAGATATTGGTATT |
| Cu*Lac12-*F / R | GAATCCACTGTTATCACCTT / TTGCGACCTAATCCATTG |
| Cu*Lac13-*F / R | CGAAGTTGATGGTGTTAA / ACGAATCCAGTAATTGTC |
| Cu*Lac14-*F / R | TTGCTGGTGGTATCAATTC / TGGTCTGCGAAGTAGTAG |
| Cu*Lac15-*F / R | CATTGAAGTTGATGGTGTT / ACGAATCCAGTAGTTGTC |
| Cu*Lac16-*F / R | AACACCAATATGACGAAGAAT / GGCAACACTGATGAACTG |
| Cu*Lac17*/18*-*F / R | GTCCTCGTCCATACCAAT / ATAAGTGAACATATCCGTCTG |
| *18S*-F / R | AGACGGAAGTTTGAGGCA / CTTCCGGCCAAGGTGAA |
