# Supplementary material for: A complex metabolic network and its biomarkers regulate laccase production in white-rot fungus Cerrena unicolor 87613
Source: Microb Cell Fact. 2024 Jun 8;23:167. doi: 10.1186/s12934-024-02443-9 (PMC11162070; doi:10.1186/s12934-024-02443-9)
Supplement: Supplementary file 3 — Supplementary Material 3 [file 12934_2024_2443_MOESM3_ESM.docx]

**Table S1 Quality control and characterization of transcription sequencing data of *Cerrena unicolor* 87613 extracts from fructose-cultivation day 6 (FCd-6) and day 10 (FCd-10), respectively.**

| **Sample** | **Total reads** | **Clean bases**  **（G）** | **Total mapped** | **Multiple mapped** | **Unique mapped** | **Error rate（%）** | **Q20（%）** | **Q30（%）** | **GC content（%）** |
| --- | --- | --- | --- | --- | --- | --- | --- | --- | --- |
| FCd-6-1 | 28,674,552 | 4.30 | 26,787,257  (93.42%) | 1,465,357  (5.11%) | 25,321,900  (88.31%) | 0.03 | 97.58 | 93.38 | 51.21 |
| FCd-6-2 | 25,415,046 | 3.81 | 24,193,855  (95.2%) | 919,078  (3.62%) | 23,274,777  (91.58%) | 0.03 | 97.68 | 93.52 | 51.15 |
| FCd-6-3 | 29,852,030 | 4.48 | 28,498,203  (95.46%) | 1,176,382  (3.94%) | 27,321,821  (91.52%) | 0.03 | 97.78 | 93.68 | 50.96 |
| FCd-10-1 | 29,195,484 | 4.38 | 27,861,667  (95.43%) | 936,212  (3.21%) | 26,925,455  (92.22%) | 0.03 | 97.73 | 93.59 | 51.43 |
| FCd-10-2 | 27,204,294 | 4.08 | 25,898,878  (95.2%) | 1,214,419  (4.46%) | 24,684,459  (90.74%) | 0.03 | 97.61 | 93.39 | 51.00 |
| FCd-10-3 | 26,132,722 | 3.92 | 24,994,781  (95.65%) | 1,159,450  (4.44%) | 23,835,331  (91.21%) | 0.03 | 97.83 | 93.85 | 50.96 |

Total reads: the number of clean reads after quality control of sequencing data;

Clean bases: The number of bases after filtering the raw data (clean base = clean reads × 150 bp);

Total mapped: The number of reads mapped to the genome and its percentage;

Multiple mapped: The number of reads mapped to multiple locations in the reference genome and its percentage;

Unique mapped: The number of reads mapped to a unique position in the reference genome and its percentage (These data were used for subsequent quantitative data analysis of reads);

Error rate: Overall sequencing error rate;

Q20 and Q30: The percentage of bases with a Phred score greater than 20 or 30 out of the total bases, respectively;

GC content: The percentage of G and C bases in the four types of bases in clean reads.
