# Supplementary material for: A complex metabolic network and its biomarkers regulate laccase production in white-rot fungus Cerrena unicolor 87613
Source: Microb Cell Fact. 2024 Jun 8;23:167. doi: 10.1186/s12934-024-02443-9 (PMC11162070; doi:10.1186/s12934-024-02443-9)
Supplement: Supplementary file 4 — Supplementary Material 4 [file 12934_2024_2443_MOESM4_ESM.docx]

**Table S4 Enrichment analysis of the KEGG pathway for the differentially expressed genes (DEGs) in FCd-6 samples versus FCd-10 samples.**

| **ID** | **Terms** | ***P*-value** | **Counts of UR genes** | **UR* genes ID** | **Counts of DR genes** | **DR* genes ID** |
| --- | --- | --- | --- | --- | --- | --- |
| tvs01110 | Biosynthesis of secondary metabolites | 0.000 | **45** | A07571.gene/A06418.gene/A09661.gene/A07430.gene/A06660.gene/A01055.gene/A03069.gene/A09077.gene/A04730.gene/A02716.gene/A06836.gene/A09251.gene/novel.1493/A09117.gene/A00473.gene/A09856.gene/A07433.gene/A03726.gene/A06757.gene/A09141.gene/A06088.gene/A05473.gene/A03023.gene/A07316.gene/A08399.gene/A06115.gene/A07832.gene/A06132.gene/A06979.gene/A09113.gene/A08064.gene/A06837.gene/A03191.gene/A07557.gene/A10114.gene/A03209.gene/A07073.gene/A05471.gene/A06380.gene/A07913.gene/novel.1433/A07533.gene/A10171.gene/A01861.gene/A09662.gene | **44** | A00533.gene/A02610.gene/A03683.gene/A07667.gene/A02126.gene/A00376.gene/A08519.gene/A01247.gene/A02991.gene/A08879.gene/A05165.gene/A08521.gene/A00342.gene/A04336.gene/A05242.gene/A03087.gene/A00980.gene/A00541.gene/A00497.gene/A06807.gene/A04878.gene/A02865.gene/A07087.gene/A04759.gene/A01658.gene/A07715.gene/A06983.gene/A01721.gene/A08480.gene/A03397.gene/A02839.gene/A08306.gene/A04580.gene/A04030.gene/A03251.gene/A00979.gene/A02116.gene/A06517.gene/A03801.gene/A06878.gene/A07278.gene/A04255.gene/A08370.gene/A08285.gene |
| tvs01230 | Biosynthesis of amino acids | 0.004 | **16** | A07571.gene/A06418.gene/A07430.gene/A06660.gene/A03069.gene/A02716.gene/A09856.gene/A03726.gene/A03023.gene/A08399.gene/A06979.gene/A09113.gene/A08064.gene/A03191.gene/A07073.gene/A06380.gene | **6** | A03087.gene/A02865.gene/A01721.gene/A08480.gene/A06878.gene/A04255.gene |
| tvs00480 | Glutathione metabolism | 0.000 | **12** | A07600.gene/A03489.gene/A03741.gene/A04730.gene/novel.1493/A07601.gene/A09881.gene/A04379.gene/A04373.gene/A00358.gene/A07225.gene/A09094.gene | **1** | A03323.gene |
| tvs01200 | Carbon metabolism | 0.000 | **11** | A07571.gene/A01055.gene/A04730.gene/A02716.gene/novel.1493/A04617.gene/A03726.gene/A03023.gene/A08399.gene/A03191.gene/A06380.gene | **25** | A07185.gene/A00533.gene/A02610.gene/A03683.gene/A07667.gene/A02126.gene/A09800.gene/A01247.gene/A00342.gene/A05242.gene/A03087.gene/A00541.gene/A00497.gene/A08417.gene/A04759.gene/A07715.gene/A01721.gene/A00276.gene/A03397.gene/A02839.gene/A08306.gene/A06878.gene/A04255.gene/A08370.gene/A01756.gene |
| tvs00270 | Cysteine and methionine metabolism | 0.006 | **9** | A07571.gene/A06418.gene/A06660.gene/A03741.gene/A09856.gene/A03726.gene/A03023.gene/A09094.gene/A08064.gene | **1** | A07130.gene |
| tvs00010 | Glycolysis / Gluconeogenesis | 0.000 | **6** | A02716.gene/A08399.gene/A06132.gene/A06380.gene/A07533.gene/A01861.gene | **11** | A07667.gene/A01247.gene/A05165.gene/A00342.gene/A04878.gene/A07715.gene/A02839.gene/A04580.gene/A06517.gene/A08370.gene/A08285.gene |
| tvs00500 | Starch and sucrose metabolism | 0.000 | **6** | A09661.gene/A06836.gene/A06115.gene/A06837.gene/A02010.gene/A09662.gene | **14** | A01410.gene/A03712.gene/A08879.gene/A01792.gene/A04336.gene/A00980.gene/A06807.gene/A01658.gene/A04030.gene/A06736.gene/A00979.gene/A07278.gene/A05970.gene/A02750.gene |
| tvs00520 | Amino sugar and nucleotide sugar metabolism | 0.010 | **5** | A06399.gene/A06397.gene/A00535.gene/A00194.gene/A06133.gene | **8** | A08202.gene/A07290.gene/A01650.gene/A00537.gene/A06361.gene/A04655.gene/A08188.gene/A09048.gene |
| tvs01210 | 2-Oxocarboxylic acid metabolism | 0.010 | **5** | A06418.gene/A07430.gene/A06660.gene/A06979.gene/A07073.gene | **4** | A03087.gene/A01721.gene/A06878.gene/A04255.gene |
| tvs00052 | Galactose metabolism | 0.000 | **4** | A06132.gene/A06380.gene/A01861.gene/A06133.gene | **6** | A08879.gene/A04336.gene/A09791.gene/A05680.gene/A06517.gene/A02849.gene |
| tvs00350 | Tyrosine metabolism | 0.001 | **4** | A06660.gene/A06088.gene/novel.1433/A07533.gene | **4** | A01591.gene/A07667.gene/A05165.gene/A03801.gene |
| tvs00620 | Pyruvate metabolism | 0.000 | **3** | A03210.gene/A04617.gene/A07369.gene | **13** | A00421.gene/A03683.gene/A03460.gene/A00422.gene/A09800.gene/A01247.gene/A00342.gene/A03461.gene/A04759.gene/A07715.gene/A04580.gene/A08370.gene/A08285.gene |
| tvs00680 | Methane metabolism | 0.000 | **3** | A02716.gene/A03023.gene/A06380.gene | **6** | A07185.gene/A07667.gene/A08417.gene/A00276.gene/A02839.gene/A08370.gene |
| tvs00910 | Nitrogen metabolism | 0.000 | **1** | A10255.gene | **6** | A01584.gene/A04382.gene/A02865.gene/A00196.gene/A08419.gene/A01756.gene |
| tvs00630 | Glyoxylate and dicarboxylate metabolism | 0.001 | **1** | A01055.gene | **11** | A00533.gene/A03683.gene/A02126.gene/A03087.gene/A01721.gene/A00276.gene/A08306.gene/A06878.gene/A04255.gene/A08370.gene/A01756.gene |
| tvs00020 | Citrate cycle (TCA cycle) | 0.000 | **0** | - | **12** | A01247.gene/A00342.gene/A05242.gene/A03087.gene/A00541.gene/A00497.gene/A04759.gene/A07715.gene/A01721.gene/A03397.gene/A06878.gene/A04255.gene |

***UR and DR genes stands for the genes with up-regulated (UR) or down-regulated (DR) expression level, respectively.**
