# Supplementary material for: A complex metabolic network and its biomarkers regulate laccase production in white-rot fungus Cerrena unicolor 87613
Source: Microb Cell Fact. 2024 Jun 8;23:167. doi: 10.1186/s12934-024-02443-9 (PMC11162070; doi:10.1186/s12934-024-02443-9)
Supplement: Supplementary file 5 — Supplementary Material 5 [file 12934_2024_2443_MOESM5_ESM.docx]

**Table S6 Enrichment analysis of the KEGG pathway for the differentially abundant metabolites (DAMs) in FCd-6 samples versus FCd-10 samples.**

| **Terms** | **Impact** | ***P*-value** | **IM* counts** | **Increased metabolites** | **DM* counts** | **Decreased metabolites** |
| --- | --- | --- | --- | --- | --- | --- |
| Phenylalanine metabolism | 0.700 | 0.013 | **4** | L-Phenylalanine; Phenethylamine; Phenylacetaldehyde; Succinic acid | **1** | Phenylpyruvic acid |
| Glutathione metabolism | 0.427 | 0.626 | **0** | -- | **2** | L-Glutamate; Glutathione |
| Tryptophan metabolism | 0.376 | 0.082 | **0** | -- | **5** | L-Tryptophan; L-5-Hydroxytryptophan; L-Kynurenine; 6-Hydroxymelatonin; Oxoadipic acid |
| Arginine biosynthesis | 0.365 | 0.048 | **1** | N-Acetylornithine | **1** | L-Argininosuccinate |
| Alanine, aspartate and glutamate metabolism | 0.342 | 0.251 | **1** | Succinic acid | **1** | L-Argininosuccinate |
| Pyrimidine metabolism | 0.329 | 0.001 | **6** | Cytidine; Cytosine; UMP; 5-Phosphoribosyl 1-pyrophosphate; Thymine; dTMP | **3** | UDP; Deoxycytidine; 2-Deoxyuridine |
| Nicotinate and nicotinamide metabolism | 0.319 | 0.063 | **1** | Succinic acid | **2** | NAD^+^; β-Nicotinamide mononucleotide |
| Glycine, serine and threonine metabolism | 0.303 | 0.102 | **0** | -- | **5** | L-Threonine; L-Serine; Cystathionine; DL-Serine; Creatine |
| Cysteine and methionine metabolism | 0.294 | 0.220 | **2** | S-Adenosylhomocysteine; 5'-S-Methyl-5'-thioadenosine | **3** | L-Serine; Cystathionine; L-Methionine |
| Methane metabolism | 0.261 | 0.273 | **1** | Glyceraldehyde 3-phosphate | **2** | Phosphoenolpyruvic acid; L-Serine |
| Pantothenate and CoA biosynthesis | 0.207 | 0.067 | **2** | Valine; 3-Methyl-2-oxobutanoic acid | **2** | Pantetheine; Pantothenic acid |
| Glycolysis / Gluconeogenesis | 0.207 | 0.582 | **1** | Glyceraldehyde 3-phosphate | **1** | Phosphoenolpyruvic acid |
| Purine metabolism | 0.188 | 0.002 | **5** | 5-Phosphoribosyl 1-pyrophosphate; Guanosine; Guanine; dAMP; Adenine | **7** | Allantoic acid; AMP; ADP; Deoxyadenosine; Deoxyinosine; Xanthine; Xanthosine |
| Riboflavin metabolism | 0.152 | 0.215 | **1** | -- | **2** | FMN; FAD |
| Fructose and mannose metabolism | 0.150 | 0.307 | **1** | D-(-)-Mannitol | **0** | -- |
| Lysine biosynthesis | 0.140 | 0.737 | **0** | -- | **1** | Oxoadipic Acid |
| Amino sugar and nucleotide sugar metabolism | 0.124 | 0.582 | **0** | -- | **2** | UDP-N-acetylglucosamine; UDP-galactose |
| Pentose phosphate pathway | 0.121 | 0.048 | **2** | 5-Phosphoribosyl 1-pyrophosphate;  Gluconic acid | **2** | 6-phospho-D-glucono-1,5-lactone; Gluconolactone |
| Terpenoid backbone biosynthesis | 0.115 | 0.737 | **0** | -- | **1** | Mevalonic acid |
| Arginine and proline metabolism | 0.112 | 0.318 | **1** | 3-Hydroxyproline | **2** | Creatine; 4-Guanidinobutanoic acid |
| Valine, leucine and isoleucine biosynthesis | 0.101 | 0.207 | **2** | 3-Methyl-2-oxobutanoic acid; Valine | **1** | L-Threonine |
| Glycerophospholipid metabolism | 0.080 | 0.739 | **2** | CDP-choline; Glycerol-3-phosphate | **0** | -- |
| Phenylalanine, tyrosine and tryptophan biosynthesis | 0.062 | 0.004 | **1** | L-Phenylalanine | **2** | Phenylpyruvic acid; L-Tyrosine |
| Glycerolipid metabolism | 0.061 | 0.689 | **1** | Glycerol-3-phosphate | **0** | -- |
| Sulfur metabolism | 0.058 | 0.661 | **1** | Succinic acid | **0** | -- |
| Citrate cycle (TCA cycle) | 0.033 | 0.481 | **1** | Succinic acid | **1** | Phosphoenolpyruvic acid |
| Vitamin B6 metabolism | 0.032 | 0.215 | **0** | -- | **1** | Pyridoxamine |
| Galactose metabolism | 0.026 | 0.758 | **0** | -- | **1** | UDP-galactose |
| Glyoxylate and dicarboxylate metabolism | 0.024 | 0.341 | **0** | -- | **1** | L-Serine |

***IM and DM stands for the increased metabolites or decreased metabolites, respectively.**
