# Supplementary material for: A complex metabolic network and its biomarkers regulate laccase production in white-rot fungus Cerrena unicolor 87613
Source: Microb Cell Fact. 2024 Jun 8;23:167. doi: 10.1186/s12934-024-02443-9 (PMC11162070; doi:10.1186/s12934-024-02443-9)
Supplement: Supplementary file 6 — Supplementary Material 6 [file 12934_2024_2443_MOESM6_ESM.docx]

**Table S7 The KEGG pathways shared by both transcriptomic and metabolomic analysis (FCd-6 samples versus FCd-10 sample).**

| **KEGG terms** | **Involved genes** | **Involved metabolites** |
| --- | --- | --- |
| Galactose metabolism | **Up-regulated:**  Galactose mutarotase; ATP-dependent 6-phosphofructokinase; Galactose kinase  **Down-regulated:**  Alpha-glucosidase; D-galactonate dehydratase; Galactose mutarotase; | **Increased:**  None  **Decreased:**  UDP-galactose |
| Glycolysis / Gluconeogenesis | **Up-regulated:**  Broad-specificity phosphatase; Aldose 1-epimerase; Glyceraldehyde-3-phosphate dehydrogenase; ATP-dependent 6-phosphofructokinase; Alcohol dehydrogenase  **Down-regulated:**  Glucose-6-phosphate 1-epimerase; Glutathione-dependent formaldehyde dehydrogenase; Phosphoenolpyruvate carboxykinase; NAD-dependent alcohol dehydrogenase; Fructose-1,6-bisphosphatase; Aldehyde dehydrogenase; Aldose 1-epimerase; Acetate--CoA ligase | **Increased:**  Glyceraldehyde 3-phosphate  **Decreased:**  Phosphoenolpyruvic acid |
| Citrate cycle (TCA cycle) | **Up-regulated:**  None  **Down-regulated:**  Phosphoenolpyruvate carboxykinase; Succinyl-CoA synthetase; 2-methylcitrate synthase, mitochondrial; Succinate dehydrogenase; Fumarate hydratase, mitochondrial; Citrate synthase, mitochondrial; Aconitate hydratase, mitochondrial; | **Increased:**  Succinic acid  **Decreased:**  Phosphoenolpyruvic acid |
| Glyoxylate and dicarboxylate metabolism | **Up-regulated:**  2-methylisocitrate lyase, mitochondrial  **Down-regulated:**  Catalase; Malate synthase; Isocitrate lyase; 2-methylcitrate synthase, mitochondrial; Citrate synthase, mitochondrial; Formate dehydrogenase; Aconitate hydratase, mitochondrial; Acetyl-coenzyme A synthetase; Formamidase | **Increased:**  None  **Decreased:**  L-Serine |
| Methane metabolism | **Up-regulated:**  Broad-specificity phosphatase; Phosphoserine aminotransferase; ATP-dependent 6-phosphofructokinase  **Down-regulated:**  Alcohol oxidase 1; Glutathione-dependent formaldehyde dehydrogenase; NAD-dependent formate dehydrogenase; Fructose-1,6-bisphosphatase, cytosolic; Acetyl-coenzyme A synthetase | **Increased:**  None  **Decreased:**  Phosphoenolpyruvic acid; L-Serine |
| Amino sugar and nucleotide sugar metabolism | **Up-regulated:**  Chitin deacetylase; Chitinase 1; Endochitinase B; Galactokinase  **Down-regulated:**  Alpha-L-arabinofuranosidase; Exo-1,4-beta-xylosidase bxlB; Chitinase 1; Endochitinase B1; Beta-N-acetylhexosaminidase; | **Increased:**  None  **Decreased:**  UDP-N-acetylglucosamine; UDP-galactose |
| Cysteine and methionine metabolism | **Up-regulated:**  Homocysteine synthase; Branched-chain-amino-acid aminotransferase, mitochondrial; Aromatic amino acid aminotransferase; Gamma-glutamylcysteine synthetase; Homoserine dehydrogenase; Cysteine synthase 1; Phosphoserine aminotransferase; Glutathione synthetase; Cystathionine gamma-lyase  **Down-regulated:**  S-methyl-5'-thioadenosine phosphorylase | **Increased:**  S-Adenosylhomocysteine; 5'-S-Methyl-5'-thioadenosine  **Decreased:**  L-Serine; Cystathionine; L-Methionine |
| Glutathione metabolism | **Up-regulated:**  Ribonucleoside-diphosphate reductase; Glutathione S-transferase; Glutathione S-transferase P; Gamma-glutamylcysteine synthetase; 6-phosphogluconate dehydrogenase; Glucose-6-phosphate dehydrogenase; oxoprolinase; Glutathione synthetase  **Down-regulated:**  Glutathione S-transferase 1 | **Increased:**  None  **Decreased:**  L-Glutamate; Glutathione |
