# Supplementary material for: A complex metabolic network and its biomarkers regulate laccase production in white-rot fungus Cerrena unicolor 87613
Source: Microb Cell Fact. 2024 Jun 8;23:167. doi: 10.1186/s12934-024-02443-9 (PMC11162070; doi:10.1186/s12934-024-02443-9)
Supplement: Supplementary file 7 — Supplementary Material 7 [file 12934_2024_2443_MOESM7_ESM.docx]

**Table S5 Collection of differentially abundant metabolites (DAMs) in the cultures from fructose-cultivated day 6 (FCd-6) versus those from FCd-10.**

| **Charge** | **ID** | **Name** | **Formula** | **MW*** | **RT**  **[min]** | **m/z** | **FCd-6_1** | **FCd-6_2** | **FCd-6_3** | **FCd-6_4** | **FCd-6_5** | **FCd-6_6** | **FCd-10_1** | **FCd-10_2** | **FCd-10_3** | **FCd-10_4** | **FCd-10_5** | **FCd-10_6** | **Log2FC*** | ***P*-value** | **VIP*** |
| --- | --- | --- | --- | --- | --- | --- | --- | --- | --- | --- | --- | --- | --- | --- | --- | --- | --- | --- | --- | --- | --- |
| pos | Com_6 | 3-methyl-5-oxo-5-(4-toluidino)pentanoic acid | C13 H17 N O3 | 257.10 | 1.25 | 258.11 | 4807431834.10 | 6381503212.15 | 8990466355.90 | 6872294336.78 | 7595059737.15 | 5424347117.39 | 19000251704.27 | 20776977742.02 | 20767895833.32 | 20776763748.62 | 19572862709.09 | 18484728764.12 | -1.57 | 0.000 | 1.664 |
| pos | Com_10 | cis-4-Hydroxy-D-proline | C5 H9 N O3 | 131.06 | 1.24 | 132.07 | 6457968197.75 | 9933447595.45 | 1783695261.10 | 7575488682.23 | 6220460960.68 | 10631412148.44 | 819692984.76 | 772652169.03 | 586450080.34 | 877772866.65 | 941345054.53 | 623783408.00 | 3.20 | 0.000 | 1.569 |
| pos | Com_13 | 9-Oxo-10(E),12(E)-octadecadienoic acid | C18 H30 O3 | 294.22 | 13.90 | 295.23 | 4149105423.80 | 4216850500.46 | 5931760336.29 | 5059422298.52 | 4570945850.06 | 4750905704.57 | 2335896238.24 | 1001832644.57 | 5277133751.25 | 1270707617.61 | 2134048657.04 | 1649093858.06 | 1.07 | 0.012 | 1.315 |
| pos | Com_37 | Indole-3-acrylic acid | C11 H9 N O2 | 187.06 | 6.69 | 188.07 | 1141931827.72 | 1073753548.58 | 1063520086.36 | 1277108376.30 | 2122947905.28 | 1705382808.84 | 3862554707.95 | 5262215834.41 | 3512507576.72 | 5211816173.67 | 3173424698.65 | 4571651336.89 | -1.61 | 0.000 | 1.040 |
| pos | Com_39 | DL-Tryptophan | C11 H12 N2 O2 | 204.09 | 6.68 | 205.10 | 1129643563.03 | 1063197907.45 | 1053317630.47 | 1261763774.97 | 2099125383.59 | 1687072554.95 | 3823013295.04 | 5213896829.87 | 3474744046.07 | 5167579578.83 | 3145889949.57 | 4537359057.48 | -1.61 | 0.000 | 1.041 |
| pos | Com_82 | Diaminopimelic acid | C7 H14 N2 O4 | 190.10 | 1.30 | 191.10 | 528738365.18 | 1517802664.08 | 2326925893.05 | 2269430175.03 | 1526532374.37 | 1132174994.44 | 282839458.92 | 271454509.75 | 238979482.72 | 274665459.80 | 348512429.33 | 257579457.75 | 2.47 | 0.001 | 1.577 |
| pos | Com_89 | 3-amino-4-(propylamino)cyclobut-3-ene-1,2-dione | C7 H10 N2 O2 | 154.07 | 3.77 | 155.08 | 948678889.60 | 985520616.42 | 1161019665.24 | 1092997855.01 | 1481270637.14 | 1382408328.77 | 2714248324.70 | 2219014439.95 | 2483957346.25 | 3067587999.65 | 2696243942.31 | 2303760927.65 | -1.13 | 0.000 | 1.367 |
| pos | Com_94 | Oxaceprol | C7 H11 N O4 | 173.07 | 1.30 | 174.08 | 523987220.37 | 1422660755.31 | 2203490454.73 | 2136649171.98 | 1440593745.28 | 1103849563.26 | 241253986.57 | 226692676.94 | 200421281.34 | 272687276.88 | 302730655.89 | 215165759.96 | 2.60 | 0.000 | 1.302 |
| pos | Com_110 | Styrene | C8 H8 | 104.06 | 5.68 | 105.07 | 200824676.74 | 1355458314.64 | 524307361.07 | 1909656130.45 | 230220434.37 | 958627014.78 | 16416398.29 | 24683109.45 | 54075131.55 | 7919907.25 | 261732701.92 | 64753808.00 | 3.59 | 0.001 | 1.221 |
| pos | Com_136 | L(-)-Carnitine | C7 H15 N O3 | 161.11 | 1.17 | 162.11 | 92555059.06 | 374774124.76 | 350048294.77 | 319707189.63 | 449773126.18 | 328004379.65 | 1052338660.99 | 1563948709.28 | 1291425625.37 | 1201500103.19 | 1974143827.74 | 1784198381.11 | -2.21 | 0.000 | 1.478 |
| pos | Com_151 | pentane-1,2,3,4,5-pentol | C5 H12 O5 | 152.07 | 1.28 | 153.08 | 764189734.52 | 1059100753.19 | 926603807.60 | 989409432.40 | 1188938286.24 | 1300358869.21 | 368677131.23 | 416697689.32 | 443483461.35 | 467240575.12 | 551096747.62 | 499212501.80 | 1.18 | 0.000 | 1.385 |
| pos | Com_152 | Valine | C5 H11 N O2 | 117.08 | 1.27 | 118.09 | 865146722.14 | 1358195276.50 | 735147911.36 | 1074757393.70 | 1373218748.41 | 1480791238.88 | 217663690.69 | 367999224.50 | 242758308.41 | 273428756.59 | 749214856.97 | 593423503.34 | 1.49 | 0.002 | 1.523 |
| pos | Com_153 | MAG (18:3) | C21 H36 O4 | 352.26 | 13.55 | 353.27 | 298481392.27 | 605190442.53 | 857867933.92 | 564158918.04 | 405440214.59 | 478324648.21 | 45957779.94 | 39622446.80 | 104885053.69 | 36040382.08 | 21862980.40 | 22749432.58 | 3.57 | 0.000 | 1.504 |
| pos | Com_190 | Cystathionine | C7 H14 N2 O4 S | 222.07 | 1.17 | 223.07 | 50790226.82 | 195343580.06 | 56092802.09 | 110305954.93 | 197496498.93 | 280860570.01 | 1157030043.87 | 1218700487.05 | 1429331640.68 | 1396625307.97 | 1048423155.98 | 1318165009.96 | -3.09 | 0.000 | 1.496 |
| pos | Com_231 | Targinine | C7 H16 N4 O2 | 188.13 | 1.16 | 189.13 | 147295150.96 | 369144168.95 | 446748582.71 | 364667802.40 | 363547335.98 | 339256610.52 | 1212590032.84 | 1153916550.06 | 1184607689.33 | 1131877328.30 | 1064641624.21 | 989406542.67 | -1.73 | 0.000 | 1.597 |
| pos | Com_315 | Piperine | C17 H19 N O3 | 285.13 | 1.37 | 286.14 | 15953067.71 | 174715459.92 | 283792366.18 | 197843570.72 | 72750642.34 | 150888280.32 | 767024759.11 | 644227268.22 | 525345413.14 | 954365228.30 | 265335440.30 | 399128619.57 | -1.99 | 0.011 | 1.296 |
| pos | Com_363 | Carbaprostacyclin | C21 H34 O4 | 332.23 | 14.35 | 333.24 | 293412183.80 | 661798837.62 | 598319356.45 | 356295325.53 | 656397275.86 | 519984544.50 | 206975985.87 | 138390948.87 | 438477204.07 | 207575693.72 | 18325396.87 | 216394198.85 | 1.33 | 0.042 | 1.080 |
| pos | Com_365 | L-Threonine | C4 H9 N O3 | 119.06 | 1.23 | 120.07 | 159994759.07 | 255171810.54 | 527953874.78 | 292063964.86 | 336316495.50 | 278287706.37 | 757595104.89 | 766369478.64 | 649166778.99 | 781533963.70 | 668088505.70 | 550857215.93 | -1.17 | 0.002 | 1.353 |
| pos | Com_367 | 3-Methyl-2-oxobutanoic acid | C5 H8 O3 | 116.05 | 1.28 | 117.05 | 355338876.02 | 555358882.79 | 509760358.74 | 523186443.73 | 628198196.92 | 667451464.01 | 180704760.92 | 213570487.94 | 226711061.14 | 227227675.33 | 277484078.06 | 240889160.86 | 1.25 | 0.000 | 1.491 |
| pos | Com_370 | Phenethylamine | C8 H11 N | 121.09 | 5.74 | 122.10 | 60701971.64 | 430571604.54 | 157722302.92 | 588147314.35 | 68053282.09 | 288760549.17 | 4971255.49 | 6495679.36 | 14949733.06 | 2923163.62 | 76857653.21 | 17976466.49 | 3.68 | 0.001 | 1.211 |
| pos | Com_393 | Testosterone undecanoate | C30 H48 O3 | 456.36 | 14.31 | 457.37 | 274525553.92 | 226685800.49 | 123425591.15 | 301851878.89 | 88229404.92 | 74571855.89 | 43462552.15 | 26918807.73 | 18311842.01 | 29505150.24 | 19657155.69 | 18199171.62 | 2.80 | 0.000 | 1.335 |
| pos | Com_439 | L-Iditol | C6 H14 O6 | 182.08 | 1.29 | 183.09 | 73130178.38 | 382269176.22 | 413456790.52 | 395124912.23 | 540372097.02 | 553602829.63 | 10300889.31 | 10166518.26 | 11406300.66 | 9371009.28 | 21106477.93 | 8669280.52 | 5.05 | 0.000 | 1.772 |
| pos | Com_560 | L-5-Hydroxytryptophan | C11 H12 N2 O3 | 220.08 | 5.78 | 221.09 | 56415591.38 | 83781128.20 | 65920453.77 | 91584903.70 | 64546080.06 | 78285499.65 | 236980225.20 | 259316505.99 | 493432279.42 | 127146928.98 | 128609093.86 | 194391279.06 | -1.71 | 0.002 | 1.304 |
| pos | Com_572 | (+/-)-CP 47,497-C7-Hydroxy metabolite | C21 H34 O3 | 334.25 | 14.20 | 335.26 | 141060559.17 | 140476184.88 | 80225787.65 | 159019015.38 | 83671994.73 | 91659975.29 | 29671647.36 | 23753686.94 | 12085749.35 | 18907951.01 | 14458128.72 | 10387206.66 | 2.67 | 0.000 | 1.445 |
| pos | Com_576 | L-Histidine | C6 H9 N3 O2 | 155.07 | 1.08 | 156.08 | 69781499.42 | 80582297.78 | 175616969.03 | 74181065.26 | 90449361.33 | 95379568.93 | 440040397.50 | 407516837.89 | 188200455.97 | 308801989.61 | 243542380.62 | 194258894.69 | -1.60 | 0.000 | 1.291 |
| pos | Com_591 | Indole | C8 H7 N | 117.06 | 6.68 | 118.07 | 96540740.55 | 81992797.62 | 86717375.04 | 113457052.00 | 192620694.32 | 152159525.46 | 344628843.18 | 457967881.77 | 316933776.18 | 488849453.15 | 277176255.13 | 399380118.28 | -1.66 | 0.000 | 1.047 |
| pos | Com_611 | 16-Heptadecyne-1,2,4-triol | C17 H32 O3 | 306.22 | 13.71 | 307.23 | 218838387.59 | 180713335.24 | 258959070.79 | 345891437.38 | 154819812.76 | 187885560.93 | 78668162.58 | 41036345.99 | 131151113.75 | 58958440.61 | 53608544.24 | 55027103.68 | 1.69 | 0.000 | 1.441 |
| pos | Com_708 | (11E,15Z)-9,10,13-trihydroxyoctadeca-11,15-dienoic acid | C18 H32 O5 | 350.21 | 11.91 | 351.21 | 17411541.95 | 15557726.61 | 23923811.63 | 24507246.83 | 12450190.43 | 10134606.83 | 84703856.35 | 127321434.76 | 37472814.75 | 103776931.49 | 257110478.94 | 109026235.95 | -2.79 | 0.000 | 1.365 |
| pos | Com_712 | U-47700-d6 | C16 H16 [2]H6 Cl2 N2 O | 334.15 | 1.24 | 335.16 | 121531916.36 | 151663578.65 | 79742944.73 | 121123718.27 | 157599617.47 | 195692568.08 | 266493891.74 | 313752491.90 | 317987196.33 | 264793235.59 | 287142136.78 | 323654959.07 | -1.10 | 0.001 | 1.073 |
| pos | Com_718 | Guanosine | C10 H13 N5 O5 | 283.09 | 3.30 | 284.10 | 187054897.78 | 231266538.86 | 235234419.36 | 282937769.22 | 186200534.80 | 200545593.45 | 32683730.57 | 42814127.00 | 81555479.78 | 34883899.61 | 46415147.61 | 32618393.49 | 2.29 | 0.000 | 1.640 |
| pos | Com_753 | N-cyclohexyl-N-methyl-6-quinoxalinecarboxamide | C16 H19 N3 O | 291.13 | 6.04 | 292.14 | 12508669.89 | 53001765.33 | 9649429.35 | 35815011.62 | 12463306.85 | 40224406.92 | 284376147.07 | 370579389.08 | 297000460.88 | 159877224.25 | 149035682.12 | 183731349.46 | -3.14 | 0.000 | 1.115 |
| pos | Com_787 | Pyridoxamine | C8 H12 N2 O2 | 168.09 | 5.73 | 169.10 | 114832493.27 | 128218961.79 | 158757131.28 | 154974968.87 | 181755944.73 | 176869554.57 | 353253885.55 | 305057419.65 | 305520379.05 | 361344965.54 | 296850711.52 | 297340547.23 | -1.07 | 0.000 | 1.330 |
| pos | Com_794 | Oleanolic acid | C30 H48 O3 | 438.35 | 14.49 | 439.36 | 195957869.68 | 149048167.66 | 93496356.53 | 135402890.69 | 77924496.49 | 85269631.03 | 63417408.43 | 46077684.87 | 30200037.34 | 33016890.78 | 24655850.92 | 26839964.15 | 1.72 | 0.000 | 1.189 |
| pos | Com_878 | DL-Serine | C3 H7 N O3 | 105.04 | 1.19 | 106.05 | 57552283.53 | 71535370.51 | 76089665.52 | 56411497.69 | 67066563.49 | 84346826.69 | 201287880.64 | 196441011.93 | 159610047.72 | 215282007.26 | 291754960.60 | 222317285.56 | -1.64 | 0.000 | 1.645 |
| pos | Com_936 | Guanine | C5 H5 N5 O | 151.05 | 3.05 | 152.06 | 142339944.86 | 183500526.41 | 184498653.12 | 198381897.92 | 144151268.68 | 173070308.04 | 30868372.92 | 40616146.67 | 74395409.45 | 24861227.09 | 3391110.03 | 27286471.95 | 2.35 | 0.006 | 1.513 |
| pos | Com_948 | 1-(3-phenylpropanoyl)-4-piperidinecarboxylic acid | C15 H19 N O3 | 261.13 | 1.22 | 262.14 | 65710617.22 | 142790536.01 | 55774462.32 | 83302783.31 | 123995437.63 | 175298212.13 | 158408543.93 | 191946108.45 | 281010554.27 | 282958853.89 | 272638899.82 | 232910204.27 | -1.13 | 0.004 | 1.199 |
| pos | Com_998 | 1,4-dihydroxyheptadec-16-en-2-yl acetate | C19 H36 O4 | 350.24 | 14.55 | 351.25 | 112660020.65 | 61177513.89 | 137482843.94 | 200314802.31 | 83840475.32 | 96803782.74 | 19482427.92 | 30027769.32 | 74734735.15 | 25350334.69 | 26132175.87 | 34231541.38 | 1.72 | 0.001 | 1.300 |
| pos | Com_999 | Glycerol-3-phosphate | C3 H9 O6 P | 172.01 | 1.47 | 173.02 | 150694525.44 | 127366047.41 | 181001689.63 | 162437604.93 | 93887907.06 | 126490082.78 | 27650978.45 | 26011971.15 | 20262830.68 | 17802608.89 | 59113102.20 | 49537603.94 | 2.07 | 0.000 | 1.745 |
| pos | Com_1046 | delta9-THC-d3 | C21 H27 [2]H3 O2 | 317.24 | 14.50 | 318.25 | 25506886.65 | 42095323.06 | 28196007.10 | 24584886.33 | 38713824.86 | 45935296.61 | 126226023.27 | 103932447.52 | 113755563.40 | 104916700.99 | 91389431.36 | 121695859.41 | -1.69 | 0.000 | 1.364 |
| pos | Com_1077 | N-Oleoyl Glycine | C20 H37 N O3 | 339.28 | 14.32 | 340.28 | 120543795.30 | 103274999.96 | 148221966.17 | 143598647.97 | 79836399.54 | 91116167.58 | 7668889.25 | 5104802.72 | 41376555.50 | 7392231.46 | 6445004.19 | 6696246.55 | 3.20 | 0.000 | 1.425 |
| pos | Com_1094 | N-Acetylornithine | C7 H14 N2 O3 | 174.10 | 1.37 | 175.11 | 134565533.60 | 149334700.56 | 104857065.38 | 130154033.24 | 89484373.66 | 221566545.10 | 40343062.69 | 47697705.95 | 55408305.50 | 58797956.19 | 36516170.36 | 44014705.92 | 1.55 | 0.000 | 1.064 |
| pos | Com_1109 | N-Tetradecanamide | C14 H29 N O | 227.22 | 14.41 | 228.23 | 42497420.08 | 124702266.72 | 62418668.48 | 52035417.89 | 143084135.48 | 122771799.46 | 171322317.77 | 191200783.45 | 146985277.48 | 188536535.98 | 218189847.12 | 208612595.43 | -1.04 | 0.011 | 1.171 |
| pos | Com_1143 | Cytosine | C4 H5 N3 O | 111.04 | 1.37 | 112.05 | 51156432.42 | 37084893.50 | 70700294.82 | 68117398.96 | 34834534.79 | 30098115.08 | 9087288.09 | 7042158.43 | 13300139.17 | 6231737.06 | 27752801.10 | 8087148.18 | 2.03 | 0.000 | 1.125 |
| pos | Com_1157 | 13,14-Dihydro prostaglandin E1 | C20 H36 O5 | 338.25 | 14.19 | 339.25 | 36415617.36 | 64363778.61 | 60948647.50 | 55945388.37 | 48337646.06 | 57270254.96 | 229481375.33 | 184156567.11 | 137286782.98 | 184628113.67 | 104358226.35 | 200538363.07 | -1.69 | 0.000 | 1.593 |
| pos | Com_1278 | 2-Methylpentanedioic acid | C6 H10 O4 | 146.06 | 1.32 | 147.07 | 36696804.78 | 148036657.33 | 124304295.41 | 136326905.47 | 163060577.96 | 186640918.68 | 20955894.82 | 19252100.53 | 16800826.10 | 20126467.07 | 23570653.42 | 17477936.30 | 2.75 | 0.001 | 1.600 |
| pos | Com_1326 | Cytidine | C9 H13 N3 O5 | 243.09 | 1.27 | 244.09 | 35155227.68 | 106764592.59 | 66064819.46 | 76986215.32 | 136261958.07 | 148320977.70 | 27317688.22 | 47203851.55 | 19921667.25 | 51988362.85 | 36135093.01 | 36142643.70 | 1.38 | 0.008 | 1.209 |
| pos | Com_1331 | Gly-Phe | C11 H14 N2 O3 | 222.10 | 6.58 | 223.11 | 52143519.27 | 28953486.26 | 72245032.52 | 55374541.90 | 32427789.58 | 30197773.09 | 10598702.63 | 10062185.30 | 19139100.91 | 9087250.32 | 16518122.31 | 11596209.87 | 1.82 | 0.000 | 1.116 |
| pos | Com_1344 | (2E,4E)-N-(2-methylpropyl)dodeca-2,4-dienamide | C16 H29 N O | 251.22 | 14.28 | 252.23 | 13230245.02 | 54221572.33 | 28802946.47 | 27366631.06 | 90424064.23 | 62014211.94 | 79416710.74 | 178843546.15 | 124436217.38 | 164731188.15 | 88475274.48 | 115010821.23 | -1.44 | 0.008 | 1.290 |
| pos | Com_1535 | N-Acetyl-D-lactosamine | C14 H25 N O11 | 383.14 | 1.32 | 384.15 | 12449895.02 | 10388494.02 | 11019932.09 | 11526203.64 | 11962498.67 | 23201205.49 | 149960776.74 | 119625468.97 | 93207998.33 | 175086927.42 | 93009304.49 | 148873070.89 | -3.28 | 0.000 | 1.713 |
| pos | Com_1537 | 3-Acetyl-11-keto-β-boswellic acid | C32 H48 O5 | 512.35 | 14.34 | 513.36 | 95193456.46 | 77783281.60 | 24218115.67 | 60794361.85 | 50321565.24 | 85658170.31 | 13758977.68 | 7713209.47 | 4637855.72 | 6407564.16 | 5395009.26 | 4318995.42 | 3.22 | 0.000 | 1.518 |
| pos | Com_1626 | Corticosterone | C21 H30 O4 | 346.21 | 12.76 | 347.22 | 27055161.80 | 45016687.29 | 133945201.22 | 55387692.19 | 21391932.91 | 36475383.80 | 9513607.39 | 9401200.79 | 17077085.81 | 9411258.02 | 11532071.97 | 9820431.31 | 2.26 | 0.002 | 1.193 |
| pos | Com_1696 | D-threo-Isocitric acid | C6 H8 O7 | 192.03 | 2.09 | 193.03 | 33592093.12 | 96174931.25 | 56149753.80 | 114766388.88 | 30740706.59 | 70251840.74 | 23268540.81 | 22347577.67 | 23692191.79 | 32681373.73 | 16795981.22 | 13381758.43 | 1.60 | 0.004 | 1.096 |
| pos | Com_1727 | 2'-Deoxyadenosine | C10 H13 N5 O3 | 251.10 | 3.02 | 252.11 | 40177710.16 | 43210630.22 | 35181377.01 | 50648331.67 | 37730695.24 | 49832532.81 | 4070506.29 | 3727764.60 | 8929090.66 | 2828421.60 | 2628318.43 | 3176430.39 | 3.34 | 0.000 | 1.458 |
| pos | Com_1761 | Lysopa 18:0 | C21 H43 O7 P | 438.28 | 11.64 | 439.28 | 16940859.02 | 31324533.29 | 64873911.46 | 45352651.55 | 29704511.43 | 34640327.97 | 116872855.92 | 94574350.13 | 127555950.15 | 124156935.42 | 102493555.02 | 97347377.14 | -1.57 | 0.001 | 1.523 |
| pos | Com_1785 | Hexanoylcarnitine | C13 H25 N O4 | 259.18 | 7.96 | 260.19 | 5274728.58 | 5782770.66 | 5124686.36 | 8709447.13 | 6656385.15 | 5345641.41 | 25485198.82 | 10278959.89 | 88859209.51 | 37372197.31 | 154856237.99 | 155505033.97 | -3.68 | 0.004 | 1.313 |
| pos | Com_1811 | 12,13-EODE | C18 H32 O3 | 296.23 | 13.05 | 297.24 | 7232968.24 | 48140370.75 | 18815667.52 | 19654622.47 | 33991090.18 | 50015135.76 | 107114898.02 | 83148538.22 | 47904219.64 | 75213734.29 | 44126954.36 | 77188912.76 | -1.29 | 0.016 | 1.064 |
| pos | Com_1835 | Pregnenolone | C21 H32 O2 | 316.24 | 14.03 | 317.25 | 78680303.05 | 60724873.70 | 35801323.85 | 67110829.40 | 25070616.18 | 34716282.62 | 8911255.75 | 6761082.45 | 12002608.06 | 6162187.58 | 4353869.64 | 3514356.63 | 2.86 | 0.000 | 1.441 |
| pos | Com_1839 | (+-)-11-hydroxy-delta9-THC-d2 | C21 H27 [2]H3 O3 | 333.24 | 14.35 | 334.24 | 66608180.69 | 94885306.46 | 89966745.78 | 104350481.85 | 100641421.67 | 116478704.46 | 43407185.98 | 33656983.49 | 56521477.68 | 43967713.15 | 18016438.47 | 30645089.20 | 1.34 | 0.001 | 1.083 |
| pos | Com_1875 | LLK | C18 H36 N4 O4 | 372.27 | 7.86 | 373.28 | 76948436.94 | 32891412.13 | 47300447.19 | 69827821.54 | 50801608.57 | 35162159.44 | 6299195.35 | 6187845.65 | 25139107.81 | 5132687.37 | 12479092.94 | 7569838.80 | 2.32 | 0.000 | 1.363 |
| pos | Com_1962 | EPK | C16 H28 N4 O6 | 186.10 | 1.01 | 187.11 | 40885119.25 | 53482897.05 | 41709194.11 | 51658889.81 | 64516333.03 | 70012541.36 | 81227791.48 | 125435230.34 | 120393128.96 | 132990650.23 | 130841112.90 | 136175278.81 | -1.17 | 0.000 | 1.246 |
| pos | Com_2009 | Mycophenolic acid | C17 H20 O6 | 302.11 | 6.41 | 303.12 | 2688351.37 | 7250291.65 | 2427537.14 | 4724759.29 | 1944559.63 | 5896996.72 | 132712769.43 | 128035950.13 | 102522322.46 | 73111510.04 | 71399232.81 | 98198090.76 | -4.60 | 0.000 | 1.233 |
| pos | Com_2022 | β-Nicotinamide mononucleotide | C11 H15 N2 O8 P | 334.06 | 1.36 | 335.06 | 40511522.87 | 49712224.77 | 104183025.68 | 47761612.34 | 40559032.47 | 47727510.83 | 107886170.06 | 118591415.15 | 90309591.10 | 114248373.55 | 137885488.06 | 120433778.27 | -1.06 | 0.002 | 1.479 |
| pos | Com_2110 | 3-[(4-hydroxyphenyl)methyl]-octahydropyrrolo[1,2-a]pyrazine-1,4-dione | C14 H16 N2 O3 | 260.12 | 8.35 | 261.12 | 28681714.64 | 30457574.15 | 41511736.37 | 42333802.38 | 46735528.45 | 46125233.66 | 118748774.81 | 89691664.47 | 100881443.76 | 122744421.49 | 87516380.09 | 94004720.99 | -1.38 | 0.000 | 1.311 |
| pos | Com_2224 | Linoleoyl ethanolamide | C20 H37 N O2 | 323.28 | 14.16 | 324.29 | 8843458.71 | 30229184.47 | 24342767.40 | 24955167.88 | 34867270.65 | 52146884.04 | 66850762.87 | 113345892.77 | 46863464.10 | 74274903.19 | 43768490.42 | 62029676.71 | -1.21 | 0.011 | 1.381 |
| pos | Com_2237 | ILK | C18 H36 N4 O4 | 372.27 | 7.10 | 373.28 | 42984902.73 | 22044323.01 | 53899466.19 | 40333760.52 | 32712364.18 | 26837011.91 | 2849624.75 | 2009506.74 | 8337779.78 | 3128353.65 | 6399755.80 | 3448593.36 | 3.06 | 0.000 | 1.245 |
| pos | Com_2312 | PC (2:0/16:2) | C26 H48 N O8 P | 533.31 | 13.22 | 534.32 | 1018518.41 | 1229112.19 | 1809557.37 | 1110244.44 | 1804127.17 | 1096365.11 | 9862769.53 | 8512428.24 | 34486848.38 | 23484398.07 | 5702906.89 | 5375673.13 | -3.44 | 0.001 | 1.266 |
| pos | Com_2349 | Androsterone | C19 H30 O2 | 290.22 | 13.37 | 291.23 | 51901642.19 | 55296935.48 | 40414749.67 | 77186374.93 | 32164106.64 | 38286018.73 | 22350112.76 | 13077561.12 | 31020569.07 | 16860456.76 | 14935585.71 | 17336346.09 | 1.35 | 0.000 | 1.275 |
| pos | Com_2503 | (5-L-Glutamyl)-L-Amino Acid | C8 H14 N2 O5 | 218.09 | 1.37 | 219.10 | 18227866.14 | 19995884.10 | 19543188.62 | 21118714.46 | 24481360.10 | 22595827.75 | 75019649.30 | 66367160.77 | 59405513.14 | 61926413.69 | 65821741.80 | 64008202.26 | -1.64 | 0.000 | 1.462 |
| pos | Com_2573 | Octadeca-11E,13E,15Z-trienoic acid | C18 H30 O2 | 278.22 | 13.05 | 279.23 | 5835539.67 | 33055926.10 | 12853351.63 | 13689273.27 | 21533561.80 | 31815278.82 | 73490138.05 | 55741210.82 | 29830001.82 | 49928348.95 | 26710199.09 | 46990390.87 | -1.25 | 0.014 | 1.066 |
| pos | Com_2577 | Propionylcarnitine | C10 H19 N O4 | 217.13 | 2.45 | 218.14 | 6333397.03 | 11861637.01 | 41707501.55 | 25762794.33 | 24603264.06 | 13524312.06 | 30767863.02 | 38533298.47 | 91948463.96 | 28869014.71 | 58618098.77 | 50050610.07 | -1.27 | 0.017 | 1.063 |
| pos | Com_2579 | 3-Methylcrotonylglycine | C7 H11 N O3 | 157.07 | 1.27 | 158.08 | 25330518.22 | 29019498.17 | 10686228.73 | 25289642.01 | 36784072.84 | 41312385.04 | 93701304.27 | 89629059.47 | 61885832.39 | 37206854.68 | 67559849.72 | 62887914.15 | -1.29 | 0.003 | 1.360 |
| pos | Com_2676 | Glycerophospho-N-palmitoyl ethanolamine | C21 H44 N O7 P | 453.29 | 14.74 | 454.29 | 481225.97 | 1161068.41 | 924134.55 | 748943.77 | 1169576.50 | 1144399.43 | 2151456.47 | 39782397.06 | 1127936.81 | 49817951.46 | 8112227.88 | 2639058.13 | -4.20 | 0.025 | 1.015 |
| pos | Com_2748 | DRH | C16 H26 N8 O6 | 213.10 | 7.76 | 214.11 | 6100541.78 | 3218479.93 | 2835894.32 | 5355182.34 | 9171819.59 | 4244780.29 | 66689750.51 | 34008456.48 | 38805899.24 | 89640092.96 | 18970295.81 | 39251153.39 | -3.22 | 0.000 | 1.411 |
| pos | Com_2816 | Allantoic acid | C4 H8 N4 O4 | 176.05 | 1.31 | 177.06 | 1469151.76 | 1666805.77 | 7098421.35 | 2050912.53 | 2271468.80 | 2111062.96 | 91167547.60 | 59575730.84 | 57350593.32 | 83290597.87 | 18865081.46 | 50672898.39 | -4.44 | 0.000 | 1.752 |
| pos | Com_2914 | UR-144 N-(2-hydroxypentyl) metabolite | C21 H29 N O2 | 327.22 | 7.78 | 328.22 | 18462823.05 | 14414698.07 | 22977769.23 | 25870319.47 | 65424731.66 | 18087484.62 | 4227748.43 | 4524559.17 | 13826435.36 | 3522899.94 | 7588876.56 | 4978005.74 | 2.10 | 0.001 | 1.490 |
| pos | Com_2999 | 15-OxoEDE | C20 H34 O3 | 322.25 | 14.09 | 323.26 | 43987957.41 | 14688800.95 | 12455194.18 | 7362244.23 | 33436648.40 | 41868682.90 | 2548567.71 | 6578924.12 | 3079166.09 | 2306714.53 | 22319571.84 | 6048576.44 | 1.84 | 0.012 | 1.341 |
| pos | Com_3069 | 11-Deoxy prostaglandin F1α | C20 H36 O4 | 362.24 | 13.94 | 363.25 | 42880483.19 | 32641332.21 | 27599147.73 | 16831275.39 | 33699434.32 | 39614438.22 | 13691732.44 | 5754027.84 | 8657085.63 | 7082643.91 | 4806372.06 | 5489810.95 | 2.09 | 0.000 | 1.752 |
| pos | Com_3138 | PC (5:0/13:1) | C26 H50 N O8 P | 535.33 | 13.21 | 536.33 | 937829.20 | 1573980.56 | 2135444.83 | 2451507.86 | 1514010.37 | 1156892.35 | 5479088.42 | 5732960.93 | 24443739.38 | 7853638.99 | 5637818.14 | 3147454.74 | -2.42 | 0.002 | 1.041 |
| pos | Com_3219 | 2-Aminopimelic acid | C7 H13 N O4 | 175.08 | 2.57 | 176.09 | 29421623.00 | 17135540.75 | 15060591.22 | 18312116.84 | 19595012.65 | 19982078.41 | 78663654.81 | 50146984.89 | 59713647.09 | 64429629.78 | 36147067.15 | 36488338.66 | -1.45 | 0.000 | 1.630 |
| pos | Com_3224 | 4,7-dimethylpyrazolo[5,1-c][1,2,4]triazine-3-carbonitrile | C8 H7 N5 | 173.07 | 1.61 | 174.08 | 35063909.18 | 57378062.00 | 5719353.48 | 53529887.43 | 41787552.29 | 66135118.89 | 7787518.91 | 8450699.14 | 8955939.04 | 7531485.53 | 7575846.48 | 8342911.12 | 2.42 | 0.011 | 1.482 |
| pos | Com_3265 | PC (14:1e/3:0) | C25 H50 N O7 P | 507.33 | 14.54 | 508.34 | 761114.81 | 1299368.45 | 2231125.10 | 1297575.22 | 787842.80 | 922907.88 | 1628872.44 | 46401639.23 | 7327850.85 | 49369556.04 | 7083441.97 | 6919965.50 | -4.02 | 0.007 | 1.106 |
| pos | Com_3286 | (+/-)11(12)-EET | C20 H32 O3 | 302.22 | 13.99 | 303.23 | 31194544.58 | 25951638.23 | 16970965.96 | 30188897.11 | 10285253.30 | 12002677.36 | 4700657.16 | 3244978.96 | 10785810.06 | 2624253.23 | 2964703.18 | 3765485.17 | 2.17 | 0.000 | 1.164 |
| pos | Com_3371 | 3-Methylthiopropylamine | C4 H11 N S | 105.06 | 1.85 | 106.07 | 27952646.59 | 38744824.54 | 25125400.87 | 50684008.36 | 42657653.82 | 53740851.93 | 4219498.43 | 6775894.20 | 5177930.63 | 2981009.35 | 19585274.18 | 11521142.19 | 2.25 | 0.001 | 1.657 |
| pos | Com_3417 | Cytidine 5'-diphosphocholine | C14 H26 N4 O11 P2 | 488.11 | 1.28 | 489.11 | 16050515.93 | 35757145.87 | 55703894.22 | 31984793.85 | 32088201.74 | 34074540.79 | 6497806.66 | 10972061.97 | 4427262.63 | 10856190.69 | 14094166.33 | 5389930.65 | 1.98 | 0.000 | 1.480 |
| pos | Com_3429 | VLK | C17 H34 N4 O4 | 358.26 | 7.05 | 359.27 | 37222220.79 | 21189803.01 | 26588961.05 | 39721282.84 | 27598134.92 | 21892151.69 | 9234063.77 | 9435314.64 | 22207241.24 | 8065884.95 | 13826908.84 | 10347650.09 | 1.25 | 0.001 | 1.238 |
| pos | Com_3643 | L-Glutathione (reduced) | C10 H17 N3 O6 S | 307.08 | 1.55 | 308.09 | 466722.35 | 6287613.77 | 7156656.44 | 6523381.25 | 8463208.77 | 4964573.43 | 66319347.45 | 52713579.63 | 56211895.79 | 58802978.88 | 34913616.73 | 60625464.19 | -3.28 | 0.002 | 1.242 |
| pos | Com_3859 | Cortisone | C21 H28 O5 | 360.19 | 10.62 | 361.20 | 1826526.52 | 3703654.81 | 5691221.67 | 4645982.61 | 4370233.82 | 5535486.00 | 10566224.41 | 7678867.20 | 14960619.50 | 15149376.20 | 6880570.44 | 13718001.77 | -1.42 | 0.001 | 1.137 |
| pos | Com_3860 | Ofloxacin impurity E | C17 H18 F N3 O4 | 347.13 | 1.24 | 348.14 | 3896714.95 | 8442887.77 | 3730076.62 | 10687435.92 | 4535789.34 | 8921147.93 | 48472967.46 | 58084319.42 | 53814747.02 | 18047314.57 | 7381382.90 | 16899685.70 | -2.33 | 0.006 | 1.216 |
| pos | Com_4166 | 7-methyl-3-nitroimidazo[1,2-a]pyridine | C8 H7 N3 O2 | 177.05 | 1.09 | 178.06 | 3551374.64 | 3212835.36 | 7997620.37 | 4539020.37 | 5386854.07 | 4863756.11 | 45068955.58 | 30870238.93 | 23001817.66 | 53341210.51 | 15243214.76 | 19687698.96 | -2.66 | 0.000 | 1.553 |
| pos | Com_4183 | Tetrahydrocortisone | C21 H32 O5 | 346.21 | 13.01 | 347.22 | 11799117.93 | 18735164.97 | 20533623.11 | 24731555.72 | 9595028.09 | 11065973.45 | 7022011.16 | 3930713.95 | 9442474.13 | 4091467.68 | 5614323.69 | 6211324.67 | 1.41 | 0.001 | 1.042 |
| pos | Com_4187 | S-(5-Adenosy)-L-Homocysteine | C14 H20 N6 O5 S | 384.12 | 1.36 | 385.13 | 1338478.68 | 3919484.29 | 7213216.85 | 3700164.06 | 9196582.60 | 8987311.95 | 30314745.21 | 32671834.41 | 50616458.00 | 21113064.17 | 28357501.15 | 30424192.35 | -2.49 | 0.001 | 1.127 |
| pos | Com_4192 | Pilocarpine | C11 H16 N2 O2 | 208.12 | 2.51 | 209.13 | 15510831.16 | 17947903.25 | 20087726.67 | 22603199.82 | 24861997.71 | 25201677.06 | 44467396.30 | 52319907.58 | 48966773.39 | 51713733.51 | 44100487.61 | 49972916.23 | -1.21 | 0.000 | 1.304 |
| pos | Com_4210 | β-Cortolone | C21 H34 O5 | 348.23 | 11.48 | 349.24 | 9440792.21 | 15667847.97 | 23071312.49 | 22241511.16 | 17104666.34 | 15787722.69 | 55264517.31 | 43054462.86 | 46201350.10 | 51531401.13 | 44897708.60 | 44879082.15 | -1.47 | 0.000 | 1.398 |
| pos | Com_4246 | MAG (18:2) | C21 H38 O4 | 354.28 | 14.33 | 355.28 | 23173922.68 | 24374728.29 | 36338516.14 | 27573030.95 | 22601743.96 | 26055543.90 | 5671209.02 | 3411754.51 | 11906756.13 | 5238206.44 | 7649093.65 | 5323329.48 | 2.03 | 0.000 | 1.235 |
| pos | Com_4273 | N-Methyllysine | C7 H16 N2 O2 | 160.12 | 1.05 | 161.13 | 15953672.63 | 36349670.70 | 23682312.72 | 37401437.00 | 25851010.36 | 32494403.06 | 5042554.62 | 7816475.35 | 2947337.89 | 4914387.69 | 8600403.13 | 5400567.32 | 2.31 | 0.000 | 1.535 |
| pos | Com_4303 | 2-{[methyl(2,3,4,5,6-pentahydroxyhexyl)amino]methylidene}malononitrile | C11 H17 N3 O5 | 271.12 | 2.37 | 272.12 | 3769203.21 | 4133589.11 | 4674658.08 | 5643058.19 | 20004153.11 | 6574085.81 | 26611639.38 | 28506808.41 | 30186106.54 | 26131944.68 | 56057149.86 | 35699079.34 | -2.18 | 0.000 | 1.224 |
| pos | Com_4320 | 3,14-dihydro-15-keto-tetranor Prostaglandin E2 | C16 H26 O5 | 320.16 | 13.55 | 321.17 | 9515491.23 | 13310793.97 | 36517004.38 | 19721993.72 | 13974940.51 | 17981174.59 | 844335.96 | 850171.39 | 4548987.23 | 867309.14 | 616284.57 | 655276.57 | 3.73 | 0.000 | 1.353 |
| pos | Com_4341 | 3-(propan-2-yl)-octahydropyrrolo[1,2-a]pyrazine-1,4-dione | C10 H16 N2 O2 | 196.12 | 7.66 | 197.13 | 10993482.76 | 14114722.71 | 19279337.81 | 18660762.63 | 17925515.45 | 20793503.35 | 48309312.19 | 41361926.75 | 44493802.59 | 50870132.26 | 40523293.71 | 43185263.81 | -1.40 | 0.000 | 1.261 |
| pos | Com_4417 | methyl 9-(2,4-dimethoxyanilino)-9-oxononanoate | C18 H27 N O5 | 337.19 | 11.66 | 338.20 | 27105981.77 | 9578108.18 | 6219193.37 | 17787902.11 | 26096871.81 | 20183458.30 | 3741372.29 | 1654183.55 | 1669496.61 | 4633046.37 | 2246392.87 | 1347222.72 | 2.81 | 0.000 | 1.431 |
| pos | Com_4444 | Ethyl chrysanthemumate | C12 H20 O2 | 196.15 | 14.38 | 197.15 | 23893144.03 | 1203584.75 | 20945892.55 | 24100977.33 | 18070843.31 | 40869635.54 | 546133.51 | 488070.34 | 6954445.77 | 378485.77 | 404373.62 | 409331.33 | 3.81 | 0.001 | 1.388 |
| pos | Com_4509 | LPC 17:2 | C25 H48 N O7 P | 505.32 | 14.21 | 506.32 | 2805871.03 | 6014298.73 | 4171264.04 | 5043402.54 | 2444154.13 | 2981173.81 | 2430362.63 | 26299548.46 | 10329022.96 | 33398059.34 | 10814475.16 | 8459667.54 | -1.97 | 0.032 | 1.011 |
| pos | Com_4564 | FLK | C21 H34 N4 O4 | 203.13 | 8.37 | 204.14 | 23718577.92 | 13697342.97 | 24834362.94 | 23964328.29 | 18101476.22 | 15549666.62 | 4953634.87 | 5408608.75 | 11856857.74 | 4963315.66 | 6379923.88 | 4673750.80 | 1.65 | 0.000 | 1.320 |
| pos | Com_4973 | PE (4:0/5:0) | C14 H28 N O8 P | 369.15 | 9.20 | 370.16 | 1204046.66 | 13953597.11 | 3645478.91 | 5732540.68 | 5190518.50 | 7926401.65 | 25377856.79 | 25060772.44 | 15780296.74 | 42797925.77 | 11397749.87 | 11390164.78 | -1.81 | 0.008 | 1.218 |
| pos | Com_4989 | Dl-3-Hydroxy-kynurenine | C10 H12 N2 O4 | 224.08 | 1.19 | 225.09 | 1093264.38 | 2547069.43 | 1719658.57 | 2112914.39 | 1234499.45 | 1164681.85 | 15493635.54 | 18708668.63 | 25648268.44 | 17353999.69 | 33833312.36 | 44649867.73 | -3.98 | 0.000 | 1.825 |
| pos | Com_5005 | PE (5:0/13:1) | C23 H44 N O8 P | 493.28 | 13.56 | 494.29 | 1483600.53 | 785800.15 | 1066896.48 | 1427943.22 | 1140334.03 | 776662.88 | 12831563.87 | 28859564.09 | 30172893.40 | 22859994.43 | 13738034.67 | 12371903.82 | -4.18 | 0.000 | 1.544 |
| pos | Com_5021 | α-Aspartylphenylalanine | C13 H16 N2 O5 | 280.11 | 6.94 | 281.11 | 21165995.13 | 11772960.62 | 27955632.72 | 5499432.59 | 13400704.11 | 12091718.05 | 2054271.56 | 2295808.01 | 5664803.18 | 1718657.47 | 2889708.25 | 2271903.63 | 2.44 | 0.000 | 1.313 |
| pos | Com_5056 | Gly-Tyr | C11 H14 N2 O4 | 238.09 | 2.57 | 239.10 | 6373664.16 | 9225955.27 | 8433882.84 | 11101046.23 | 23362000.18 | 13997610.30 | 34318994.36 | 41494215.34 | 28279559.81 | 32366199.68 | 24135605.34 | 43231762.03 | -1.49 | 0.001 | 1.162 |
| pos | Com_5070 | gamma-Glutamylglutamic acid | C10 H16 N2 O7 | 276.10 | 2.01 | 277.10 | 1580623.23 | 11487860.42 | 9507704.24 | 12308286.84 | 11137355.64 | 10508773.26 | 33828638.19 | 37372717.50 | 19606344.02 | 39963559.18 | 40849072.62 | 43765247.26 | -1.93 | 0.005 | 1.218 |
| pos | Com_5109 | NAD+ | C21 H27 N7 O14 P2 | 663.11 | 1.69 | 664.12 | 1679640.09 | 1427801.60 | 3440049.51 | 1069656.06 | 3106764.19 | 2322596.28 | 4318790.72 | 7566663.50 | 6532943.81 | 10788496.36 | 11052185.03 | 5675351.26 | -1.82 | 0.000 | 1.021 |
| pos | Com_5182 | Deoxyadenosine | C10 H13 N5 O3 | 251.10 | 1.38 | 252.11 | 2368991.61 | 5772345.09 | 4517873.99 | 4920137.85 | 5405945.06 | 4612336.97 | 12641277.70 | 13879958.13 | 14782195.76 | 10766245.08 | 12728034.73 | 11501545.77 | -1.47 | 0.000 | 1.195 |
| pos | Com_5300 | 8-Hydroxyquinoline | C9 H7 N O | 145.05 | 7.24 | 146.06 | 1791516.04 | 3444101.49 | 6730666.54 | 5580530.37 | 3659473.16 | 2485244.64 | 27860322.04 | 16151077.15 | 25257757.43 | 25233485.19 | 11506934.98 | 12365852.60 | -2.32 | 0.000 | 1.387 |
| pos | Com_5448 | Testosterone | C19 H28 O2 | 288.21 | 14.15 | 289.22 | 16212588.88 | 16107596.20 | 6901102.48 | 19619990.24 | 15304431.59 | 12533932.06 | 6845759.87 | 4520069.01 | 9099938.93 | 4517163.78 | 4906248.82 | 5238781.61 | 1.30 | 0.001 | 1.033 |
| pos | Com_5508 | 2-acetamido-3-(4-methoxyphenyl)propanoic acid | C12 H15 N O4 | 237.10 | 6.93 | 238.11 | 5026594.49 | 12262987.06 | 4494444.82 | 10749013.48 | 8060363.31 | 13888931.83 | 32366364.80 | 29408232.52 | 35630065.78 | 20359288.45 | 15155868.85 | 19301043.13 | -1.48 | 0.001 | 1.135 |
| pos | Com_5597 | 1-Phenyl-3-methyl-5-pyrazolone | C10 H10 N2 O | 174.08 | 3.61 | 175.09 | 2919520.92 | 3450265.75 | 3254649.00 | 3214235.77 | 5945268.60 | 4992573.10 | 38419290.40 | 26449957.12 | 25716966.06 | 34996890.11 | 11023563.92 | 19020664.75 | -2.71 | 0.000 | 1.655 |
| pos | Com_5695 | AKK | C15 H31 N5 O4 | 345.24 | 0.90 | 173.63 | 19433808.17 | 13471216.73 | 22209554.80 | 22506330.96 | 23255877.63 | 20646194.15 | 2724185.11 | 2469778.32 | 4265067.61 | 3749495.77 | 3620688.77 | 2837090.70 | 2.63 | 0.000 | 1.803 |
| pos | Com_5729 | Creatine | C4 H9 N3 O2 | 131.07 | 1.28 | 132.08 | 328291.78 | 529275.23 | 19719424.06 | 433392.38 | 1770756.19 | 664524.82 | 21727633.25 | 17158792.73 | 21941221.42 | 19066735.10 | 18491311.95 | 15365415.72 | -2.28 | 0.006 | 1.272 |
| pos | Com_5767 | Artemisinin | C15 H22 O5 | 282.14 | 11.58 | 283.15 | 18674867.01 | 18364351.42 | 9005757.99 | 25303827.03 | 4267375.37 | 6350431.82 | 2870954.75 | 1900859.85 | 5540220.89 | 2309686.63 | 1702966.29 | 2234970.97 | 2.31 | 0.002 | 1.119 |
| pos | Com_5883 | Isoquinoline | C9 H7 N | 129.06 | 5.78 | 130.07 | 2918921.37 | 4585067.48 | 3523461.01 | 5246337.43 | 3943492.30 | 4566928.54 | 14588561.60 | 15524822.54 | 32548849.53 | 11837223.68 | 7271144.95 | 10806087.58 | -1.90 | 0.001 | 1.419 |
| pos | Com_5905 | TLK | C16 H32 N4 O5 | 360.24 | 3.34 | 361.24 | 14356844.60 | 6828685.69 | 15433036.37 | 24445996.95 | 11904951.92 | 9631332.65 | 1856731.70 | 1647022.64 | 4445064.32 | 1850034.81 | 2631661.37 | 1996113.23 | 2.52 | 0.000 | 1.395 |
| pos | Com_5952 | EKK | C17 H33 N5 O6 | 403.24 | 0.91 | 202.63 | 16564515.34 | 16578676.29 | 21675982.83 | 19791227.81 | 15289019.08 | 20353369.16 | 2870242.52 | 2455225.51 | 2789330.71 | 2899043.88 | 2661383.37 | 3068671.04 | 2.72 | 0.000 | 1.669 |
| pos | Com_6004 | 7-Ketocholesterol | C27 H44 O2 | 400.33 | 14.39 | 401.34 | 18035640.08 | 8315670.41 | 5475260.39 | 5973085.07 | 7911501.61 | 10006881.71 | 6049423.94 | 3346107.04 | 2227877.01 | 4397687.63 | 2855649.99 | 1603512.11 | 1.44 | 0.003 | 1.219 |
| pos | Com_6058 | L-Palmitoylcarnitine | C23 H45 N O4 | 399.33 | 13.91 | 400.34 | 13844142.89 | 6095810.82 | 15129944.48 | 8300633.63 | 5331516.96 | 3496335.87 | 490467.32 | 286683.89 | 1483976.58 | 439555.87 | 767282.99 | 665671.83 | 3.66 | 0.000 | 1.301 |
| pos | Com_6217 | GLK | C14 H28 N4 O4 | 316.21 | 3.34 | 317.22 | 16270976.40 | 11532085.05 | 12111989.90 | 19990806.13 | 16564103.10 | 11754162.77 | 1709949.38 | 2627985.50 | 5340442.33 | 2759522.85 | 2966366.68 | 2383293.62 | 2.31 | 0.000 | 1.668 |
| pos | Com_6232 | L-Phenylalanine | C9 H11 N O2 | 165.08 | 6.59 | 166.09 | 8912657.29 | 6904051.26 | 11710659.21 | 10093799.19 | 8064528.32 | 8713217.54 | 3926111.50 | 3690781.00 | 4880126.17 | 3969506.31 | 4876584.91 | 3826564.77 | 1.11 | 0.000 | 1.171 |
| pos | Com_6254 | Lysops 22:5 | C28 H46 N O9 P | 571.29 | 12.04 | 572.30 | 291938.90 | 4396463.54 | 4955811.05 | 10457346.89 | 10396044.75 | 2537464.44 | 27251031.78 | 12954179.69 | 15312070.25 | 16511828.72 | 34421667.59 | 27730881.95 | -2.02 | 0.020 | 1.066 |
| pos | Com_6515 | RLH | C18 H32 N8 O4 | 212.13 | 1.37 | 213.13 | 10353274.47 | 9571552.81 | 10678319.69 | 14086618.64 | 15795920.29 | 9377687.63 | 3427114.69 | 4525464.90 | 8421787.32 | 3744259.21 | 6380261.68 | 4844090.59 | 1.16 | 0.001 | 1.279 |
| pos | Com_6539 | Arachidonic acid | C20 H32 O2 | 304.24 | 13.95 | 305.25 | 16263592.84 | 9581160.50 | 8103268.96 | 5365784.74 | 9387310.93 | 10785907.04 | 2814294.64 | 1934792.00 | 1010046.05 | 2026809.49 | 1072298.33 | 1960630.68 | 2.46 | 0.000 | 1.845 |
| pos | Com_6600 | 4-methylpyridine-3-sulfonic acid | C6 H7 N O3 S | 173.01 | 2.12 | 174.02 | 7632150.58 | 10383043.61 | 11723905.25 | 12239559.41 | 11762462.11 | 11383690.73 | 26555107.06 | 17896139.31 | 24692305.52 | 29609214.36 | 20387566.41 | 24055315.12 | -1.14 | 0.000 | 1.130 |
| pos | Com_6703 | SLH | C15 H25 N5 O5 | 355.18 | 1.38 | 356.19 | 8989895.77 | 6879483.26 | 11238457.49 | 9826273.39 | 22280925.90 | 10579304.63 | 3599920.95 | 3366394.58 | 4440959.10 | 1997934.31 | 6444201.17 | 4933689.91 | 1.49 | 0.001 | 1.395 |
| pos | Com_6839 | 2-Methylbutyroylcarnitine | C12 H23 N O4 | 245.16 | 11.58 | 246.17 | 6064078.13 | 9576837.73 | 4368452.99 | 18963695.04 | 2481863.96 | 5305719.97 | 2093066.97 | 1814946.01 | 2965483.95 | 1572877.31 | 1951509.45 | 1849547.33 | 1.93 | 0.008 | 1.003 |
| pos | Com_6862 | Norharman | C11 H8 N2 | 168.07 | 7.65 | 169.08 | 7346993.47 | 14456997.51 | 11472075.71 | 14764095.04 | 21835014.66 | 18606365.63 | 1689772.69 | 1822800.59 | 3022553.67 | 1445663.36 | 1468471.29 | 1570598.75 | 3.01 | 0.000 | 1.301 |
| pos | Com_6891 | ethyl 2-[(6-oxo-6H-benzo[c]chromen-3-yl)oxy]acetate | C17 H14 O5 | 596.17 | 6.16 | 299.09 | 123356.85 | 537418.72 | 153903.21 | 626055.76 | 815630.59 | 299384.99 | 29525341.11 | 13687840.51 | 10440214.42 | 18598214.30 | 8342730.54 | 25852686.03 | -5.38 | 0.000 | 1.608 |
| pos | Com_7298 | 2'-Deoxyadenosine-5'-monophosphate | C10 H14 N5 O6 P | 331.07 | 1.97 | 332.07 | 4521836.62 | 4378862.84 | 4062494.19 | 6887165.82 | 5130463.84 | 5714336.75 | 1574318.15 | 2188865.57 | 2364126.49 | 1474047.76 | 1191571.91 | 1343905.80 | 1.60 | 0.000 | 1.011 |
| pos | Com_7399 | 7-alpha-carboxy-17-alpha-carboxyethylandrostan lactone phenyl ester | C28 H38 O5 | 454.27 | 10.48 | 455.28 | 2368594.10 | 3259272.30 | 4784164.51 | 3571760.96 | 3145794.15 | 3393407.42 | 13371644.52 | 11902811.88 | 16136643.60 | 14801437.25 | 13401272.53 | 14310065.77 | -2.03 | 0.000 | 1.464 |
| pos | Com_7503 | cis-7-Hexadecenoic Acid | C16 H30 O2 | 254.22 | 13.83 | 255.23 | 1998374.89 | 4791172.49 | 3450821.92 | 3055023.57 | 3844334.57 | 3557633.84 | 13684831.74 | 16538423.48 | 13208087.27 | 11431629.06 | 14575077.26 | 26191336.48 | -2.21 | 0.000 | 1.739 |
| pos | Com_7633 | 7-Ketodeoxycholic acid | C24 H38 O5 | 406.27 | 14.24 | 407.28 | 4286263.73 | 6348653.92 | 2913122.34 | 2045096.32 | 5678523.56 | 8069946.69 | 15702363.55 | 18088909.48 | 11947116.76 | 17946856.41 | 12537711.49 | 14999673.66 | -1.64 | 0.001 | 1.413 |
| pos | Com_7677 | QLK | C17 H33 N5 O5 | 387.25 | 1.39 | 388.25 | 11993749.33 | 7996408.32 | 11908954.68 | 12343737.83 | 5876687.18 | 10275919.30 | 991722.21 | 2060240.79 | 4630550.75 | 1083910.84 | 2317989.04 | 2551258.31 | 2.15 | 0.000 | 1.452 |
| pos | Com_7740 | 3-(3,4,5-trimethoxyphenyl)propanoic acid | C12 H16 O5 | 222.09 | 10.82 | 223.09 | 2945557.05 | 2503392.32 | 3945252.61 | 4002033.31 | 3800536.53 | 2576561.00 | 10378064.43 | 8560902.98 | 16260820.52 | 10082016.29 | 9183368.40 | 2710281.35 | -1.53 | 0.009 | 1.040 |
| pos | Com_7771 | Sodium Dehydrocholate | C24 H33 Na O5 | 424.22 | 12.86 | 425.23 | 3076638.49 | 10800734.38 | 9389659.81 | 12159573.60 | 9611242.99 | 9385637.66 | 4145942.65 | 2964686.10 | 5457620.71 | 2190338.82 | 2415284.69 | 1527461.65 | 1.54 | 0.003 | 1.259 |
| pos | Com_7775 | N1-(2-amino-2-oxoethyl)-2-(isopropylthio)acetamide | C7 H14 N2 O2 S | 230.07 | 8.45 | 231.08 | 3868203.60 | 5151266.83 | 7678256.59 | 6523659.33 | 5228138.09 | 5159999.61 | 22278677.56 | 17641265.93 | 11481412.68 | 12963871.87 | 12804876.50 | 12796109.88 | -1.42 | 0.000 | 1.522 |
| pos | Com_7789 | Pantothenic acid | C9 H17 N O5 | 219.11 | 6.31 | 220.12 | 4877238.13 | 3729698.93 | 8805632.36 | 3467454.41 | 2762260.77 | 3209355.87 | 9945180.27 | 13033709.33 | 17011736.36 | 9975926.07 | 13794499.14 | 23936276.88 | -1.71 | 0.000 | 1.385 |
| pos | Com_7814 | 3b,7b-Dihydroxy-5-androsten-17-one | C19 H28 O3 | 304.20 | 12.95 | 305.21 | 11160449.46 | 12177261.64 | 10261149.95 | 17112689.68 | 10327069.25 | 11902915.67 | 6199710.59 | 3777680.37 | 7170826.69 | 5322816.95 | 4543137.86 | 4575659.31 | 1.21 | 0.000 | 1.306 |
| pos | Com_7985 | Dinophysistoxin-2 | C44 H68 O13 | 786.45 | 7.38 | 394.23 | 3586241.77 | 3928682.06 | 3416726.96 | 3520642.55 | 7470348.28 | 6903333.66 | 18981551.77 | 22835432.34 | 11303513.91 | 18224148.18 | 13382914.98 | 9907194.04 | -1.72 | 0.000 | 1.227 |
| pos | Com_8184 | L-Kynurenine | C10 H12 N2 O3 | 208.08 | 4.70 | 209.09 | 1992550.60 | 4023092.65 | 5438177.18 | 4551069.55 | 3779254.35 | 3682794.84 | 12619588.56 | 12380082.70 | 21376999.65 | 9226257.12 | 5248098.68 | 7822655.68 | -1.55 | 0.002 | 1.348 |
| pos | Com_8190 | JWH-018 N-(3-methylbutyl) isomer | C24 H23 N O | 341.18 | 10.52 | 342.19 | 8618620.90 | 6873796.86 | 8028861.35 | 15631421.84 | 9283972.93 | 6883247.94 | 5334762.43 | 2077520.27 | 2467957.23 | 1822814.42 | 1795684.41 | 1507178.03 | 1.88 | 0.000 | 1.218 |
| pos | Com_8250 | Lysopc 17:0 | C25 H52 N O7 P | 509.35 | 15.58 | 510.36 | 3065870.39 | 11385301.46 | 8546554.37 | 6772140.85 | 11914345.85 | 15302460.63 | 2768265.52 | 1716801.43 | 1665039.47 | 1990528.98 | 2102789.37 | 1988961.36 | 2.22 | 0.001 | 1.308 |
| pos | Com_8267 | UDP | C9 H14 N2 O12 P2 | 404.00 | 1.66 | 405.01 | 1085662.37 | 2277152.67 | 1164304.41 | 1489218.24 | 5398114.67 | 6329105.32 | 11995759.60 | 11189604.68 | 9583654.09 | 22105558.35 | 6642508.80 | 6543655.18 | -1.94 | 0.003 | 1.224 |
| pos | Com_8380 | Kinetin | C10 H9 N5 O | 215.08 | 1.29 | 233.11 | 1962140.93 | 2070267.94 | 2205851.66 | 2080876.40 | 1488054.26 | 1951501.44 | 8236928.60 | 14999126.11 | 7523655.50 | 2239482.11 | 11615506.04 | 2528726.41 | -2.00 | 0.014 | 1.040 |
| pos | Com_8398 | NPK | C15 H27 N5 O5 | 357.20 | 6.71 | 358.21 | 11237181.90 | 6808285.67 | 10477093.59 | 11641168.90 | 12176832.67 | 10699427.88 | 2877970.29 | 3461662.27 | 4118284.85 | 2332370.11 | 3070900.48 | 2764692.74 | 1.76 | 0.000 | 1.476 |
| pos | Com_8607 | 3-hydroxy-2-(3-nitro-4-piperidinobenzyl)propanenitrile | C15 H19 N3 O3 | 311.13 | 12.87 | 312.13 | 1339725.92 | 7900393.05 | 16906405.69 | 9773217.42 | 4883942.55 | 7648603.95 | 328522.39 | 722277.69 | 258722.77 | 744014.77 | 993398.66 | 559810.09 | 3.75 | 0.000 | 1.504 |
| pos | Com_8659 | (+)-alpha-Lipoic acid | C8 H14 O2 S2 | 206.04 | 5.47 | 207.05 | 4390183.54 | 5030456.15 | 2594070.97 | 4033854.55 | 6048075.16 | 7094952.83 | 21303052.43 | 15342464.97 | 16881881.15 | 20726941.51 | 16463898.86 | 18259777.86 | -1.90 | 0.000 | 1.346 |
| pos | Com_8764 | AKB48 N-(4-hydroxypentyl) metabolite | C23 H31 N3 O2 | 381.24 | 7.43 | 382.24 | 8635664.34 | 8251990.00 | 10550207.22 | 9377398.55 | 5117918.91 | 4606351.28 | 1650443.86 | 1060735.77 | 2900040.48 | 735471.50 | 2974668.75 | 1566682.18 | 2.10 | 0.000 | 1.267 |
| pos | Com_8827 | INK | C16 H31 N5 O5 | 188.12 | 3.46 | 189.12 | 9657205.05 | 5866768.84 | 6426844.90 | 11027186.59 | 9469144.17 | 8517894.09 | 2960465.48 | 2307288.71 | 9642498.51 | 1997249.56 | 4634697.37 | 2379875.28 | 1.09 | 0.012 | 1.118 |
| pos | Com_8883 | 2-(14,15-Epoxyeicosatrienoyl) glycerol | C23 H38 O5 | 394.27 | 13.85 | 395.28 | 5016135.77 | 13613148.15 | 9690625.36 | 13502795.82 | 6302504.60 | 11327298.42 | 7747026.72 | 4617466.39 | 4702049.83 | 4660687.76 | 3643437.21 | 4016150.57 | 1.02 | 0.009 | 1.279 |
| pos | Com_8971 | EMK | C16 H30 N4 O6 S | 388.18 | 7.03 | 389.19 | 10629935.15 | 6078713.38 | 8740761.20 | 9359038.58 | 6984846.47 | 6483165.87 | 2616315.57 | 1598513.10 | 5871698.43 | 1510409.08 | 2693743.49 | 1596642.40 | 1.60 | 0.001 | 1.004 |
| pos | Com_8984 | 6-Hydroxymelatonin | C13 H16 N2 O3 | 248.11 | 8.01 | 249.12 | 5235763.34 | 3662883.44 | 6588492.53 | 6012675.49 | 8208528.59 | 8650308.82 | 20130519.35 | 15959474.94 | 17041660.62 | 19741481.13 | 17535726.24 | 16827639.59 | -1.48 | 0.000 | 1.327 |
| pos | Com_8985 | Adrenic acid | C22 H36 O2 | 332.27 | 15.01 | 333.28 | 10736746.49 | 15488264.25 | 5689942.10 | 11725309.74 | 7191066.60 | 13156023.72 | 2052901.66 | 1570306.08 | 1024147.24 | 2176256.03 | 1534871.71 | 2014412.26 | 2.62 | 0.000 | 1.528 |
| pos | Com_9018 | RLK | C18 H37 N7 O4 | 415.29 | 1.39 | 416.30 | 10688914.74 | 7286782.34 | 8109771.62 | 9912034.12 | 4575295.14 | 7712810.70 | 1962797.73 | 1247758.75 | 2402057.12 | 1878659.63 | 2564376.87 | 2019136.53 | 2.00 | 0.000 | 1.555 |
| pos | Com_9039 | gamma-Glutamyltyrosine | C14 H18 N2 O6 | 310.12 | 5.93 | 311.12 | 164015.57 | 209513.77 | 150409.90 | 177530.14 | 367243.95 | 186191.92 | 9673119.97 | 9168061.34 | 571531.04 | 16069382.97 | 2641102.86 | 5068757.32 | -5.11 | 0.001 | 1.360 |
| pos | Com_9160 | VNK | C15 H29 N5 O5 | 359.22 | 1.25 | 360.22 | 5123863.24 | 10239134.72 | 8753297.30 | 13868434.82 | 11767637.48 | 11475050.51 | 2157704.84 | 5684813.89 | 7563510.19 | 2953027.74 | 4860724.46 | 3953424.20 | 1.17 | 0.005 | 1.351 |
| pos | Com_9234 | L-Argininosuccinate | C10 H18 N4 O6 | 290.12 | 1.36 | 291.13 | 3036683.13 | 6389938.77 | 14927240.72 | 9722954.70 | 3023350.63 | 6128963.92 | 13141888.92 | 16871105.94 | 12581416.11 | 18311665.32 | 11798595.58 | 13851704.69 | -1.00 | 0.021 | 1.158 |
| pos | Com_9295 | Bisphenol A | C15 H16 O2 | 228.12 | 13.30 | 229.12 | 4320791.43 | 1183923.82 | 1154949.01 | 1156827.99 | 2119743.76 | 490587.27 | 147486.27 | 123677.81 | 158880.40 | 126357.43 | 136423.91 | 172296.28 | 3.59 | 0.000 | 1.202 |
| pos | Com_9470 | N-[(4-hydroxy-3-methoxyphenyl)methyl]-8-methylnonanamide | C18 H29 N O3 | 307.21 | 13.68 | 308.22 | 2315510.52 | 2760894.75 | 3097869.42 | 3055252.17 | 3504491.89 | 3002194.55 | 19431232.69 | 7479689.97 | 7497064.46 | 6341684.18 | 6892885.56 | 9701114.22 | -1.69 | 0.001 | 1.449 |
| pos | Com_9774 | LPE 17:2 | C22 H42 N O7 P | 463.27 | 14.19 | 464.28 | 552559.52 | 302960.72 | 320679.90 | 312817.94 | 317587.92 | 312573.63 | 362910.06 | 12964701.78 | 2537137.20 | 8045651.77 | 5269500.61 | 2563169.18 | -3.90 | 0.006 | 1.156 |
| pos | Com_9860 | 2-hydroxy-3,6-diphenylcyclohexyl acetate | C20 H22 O3 | 292.14 | 9.64 | 293.15 | 1160766.04 | 2097129.65 | 1845272.45 | 2041322.17 | 1926980.95 | 3849390.92 | 13030533.26 | 12381584.24 | 13760933.59 | 17560474.41 | 10673364.36 | 10327411.76 | -2.59 | 0.000 | 1.456 |
| pos | Com_9875 | 4-Hexyloxyaniline | C12 H19 N O | 193.15 | 11.19 | 194.15 | 932817.33 | 1260107.04 | 1027081.22 | 1415009.94 | 803765.19 | 993218.87 | 186321.19 | 127172.69 | 180062.37 | 140243.83 | 236325.35 | 129431.98 | 2.69 | 0.000 | 1.025 |
| pos | Com_9882 | Estrone | C18 H22 O2 | 270.16 | 14.37 | 271.17 | 2518531.73 | 6481343.79 | 7295129.04 | 9216168.18 | 2406057.60 | 6580903.97 | 1564693.62 | 1074182.22 | 3063606.19 | 1193475.92 | 1381573.51 | 1642717.31 | 1.80 | 0.002 | 1.179 |
| pos | Com_10110 | Lysopc 18:3 | C26 H48 N O7 P | 517.31 | 11.99 | 518.32 | 1533057.29 | 892911.44 | 270457.96 | 654209.73 | 2726832.06 | 931996.74 | 17814813.86 | 7512574.00 | 4089438.51 | 11282824.13 | 4002351.98 | 2428635.13 | -2.75 | 0.001 | 1.492 |
| pos | Com_10195 | 5'-S-Methyl-5'-thioadenosine | C11 H15 N5 O3 S | 297.09 | 6.82 | 298.10 | 4709057.15 | 8838214.11 | 13562728.54 | 8879679.25 | 4428937.52 | 5059102.53 | 1535915.24 | 1428394.48 | 249914.50 | 4799121.01 | 3612420.17 | 681718.66 | 1.89 | 0.013 | 1.472 |
| pos | Com_10314 | Etiocholanolone | C19 H30 O2 | 272.21 | 14.25 | 273.22 | 8981017.91 | 7194260.46 | 4647223.67 | 6784033.30 | 3739270.92 | 5154372.82 | 1686324.55 | 1208913.20 | 1583888.47 | 1619495.30 | 995675.18 | 939658.28 | 2.18 | 0.000 | 1.409 |
| pos | Com_10349 | PC (14:1e/2:0) | C24 H48 N O7 P | 493.32 | 14.40 | 494.32 | 293431.86 | 300386.69 | 390038.73 | 341064.37 | 268322.78 | 305496.52 | 8241632.53 | 9500117.68 | 2165741.67 | 16424906.81 | 1716886.91 | 1094331.40 | -4.37 | 0.002 | 1.476 |
| pos | Com_10411 | 7-Ketolithocholic acid | C24 H38 O4 | 390.28 | 13.85 | 391.28 | 5139291.02 | 11187280.20 | 7432357.26 | 10791894.60 | 6153976.93 | 6937644.30 | 4629190.34 | 3559008.03 | 4664697.82 | 3933868.31 | 3517655.31 | 3351183.49 | 1.01 | 0.002 | 1.364 |
| pos | Com_10492 | Sebacic acid | C10 H18 O4 | 202.12 | 9.31 | 203.13 | 2169796.17 | 2292738.22 | 3487393.19 | 2749966.92 | 2351886.31 | 2487321.18 | 8223959.13 | 6309090.60 | 15437936.30 | 5529156.06 | 4246153.24 | 11416342.04 | -1.72 | 0.002 | 1.276 |
| pos | Com_10574 | 4-(tert-butyl)phenyl 3,5-dimethylisoxazole-4-carboxylate | C16 H19 N O3 | 273.13 | 2.76 | 274.14 | 906329.06 | 2377382.70 | 2737591.34 | 2226958.13 | 3205588.30 | 2817760.18 | 11264380.66 | 15820160.01 | 12891643.10 | 15962764.51 | 12314985.50 | 14249824.72 | -2.53 | 0.000 | 1.397 |
| pos | Com_10691 | Psoralidin | C20 H16 O5 | 336.10 | 1.31 | 337.11 | 7323240.74 | 9861187.38 | 7653447.08 | 6714821.77 | 10623719.02 | 12373939.10 | 1542269.39 | 1636837.36 | 1331070.11 | 882000.92 | 3976942.89 | 3903636.60 | 2.04 | 0.001 | 1.553 |
| pos | Com_11197 | FPK | C20 H30 N4 O4 | 390.22 | 6.76 | 391.23 | 3879823.90 | 5113853.58 | 4797810.67 | 6537247.59 | 8459580.66 | 8091112.51 | 1528239.33 | 1535055.85 | 4609823.99 | 1086744.75 | 1808606.42 | 1560860.98 | 1.60 | 0.001 | 1.446 |
| pos | Com_11359 | N-{[5-(tert-butyl)-2-thienyl]carbonyl}-N'-(6-methyl-2-pyridyl)urea | C16 H19 N3 O2 S | 317.12 | 3.83 | 318.13 | 3573587.30 | 3344443.66 | 3278853.15 | 5131757.81 | 5070589.08 | 4892087.20 | 11951410.81 | 11015084.49 | 12534182.40 | 14480959.91 | 10611311.46 | 11201057.00 | -1.51 | 0.000 | 1.001 |
| pos | Com_11360 | N1-(2,3-dihydro-1,4-benzodioxin-6-yl)acetamide | C10 H11 N O3 | 193.07 | 8.17 | 194.08 | 4822387.15 | 7751736.14 | 3453387.91 | 4540252.21 | 5395520.59 | 7504873.21 | 13825141.45 | 12341707.16 | 11172729.04 | 11084712.49 | 10311403.22 | 15159017.59 | -1.14 | 0.001 | 1.112 |
| pos | Com_11406 | 4-methyl-5-oxo-2-pentyl-2,5-dihydrofuran-3-carboxylic acid | C11 H16 O4 | 212.10 | 8.54 | 213.11 | 1506313.16 | 1491546.47 | 2077738.93 | 1724111.42 | 2179877.56 | 1960306.28 | 6377204.28 | 4732632.76 | 4699015.08 | 5187193.71 | 6277837.84 | 4128981.96 | -1.52 | 0.000 | 1.354 |
| pos | Com_11670 | Methyltestosterone | C20 H30 O2 | 302.22 | 13.34 | 303.23 | 7563840.08 | 6040197.36 | 2223356.05 | 6318871.53 | 2473267.24 | 3612415.51 | 1273209.22 | 1352175.85 | 1832901.84 | 1394399.55 | 1819866.03 | 1030614.52 | 1.70 | 0.002 | 1.441 |
| pos | Com_11699 | VMK | C16 H32 N4 O4 S | 358.20 | 6.90 | 359.21 | 7540082.33 | 3334321.09 | 6414730.27 | 5749358.12 | 5555189.64 | 4269880.06 | 681097.64 | 1071612.20 | 2264249.26 | 929723.35 | 1453709.91 | 1039044.98 | 2.14 | 0.000 | 1.400 |
| pos | Com_11702 | 10-Nitrolinoleate | C18 H31 N O4 | 307.21 | 10.26 | 308.22 | 195825.50 | 201203.32 | 284659.46 | 277340.30 | 169879.60 | 147153.97 | 10457740.27 | 4995432.78 | 13252587.07 | 4580388.64 | 4240658.91 | 8271862.88 | -5.17 | 0.000 | 1.550 |
| pos | Com_11887 | Eicosapentaenoic acid ethyl ester | C22 H34 O2 | 330.26 | 14.16 | 331.26 | 7385216.44 | 3179218.88 | 1762166.26 | 3929653.67 | 1873633.86 | 2576930.46 | 1474283.64 | 931041.05 | 422809.43 | 1064309.59 | 694355.22 | 425755.01 | 2.05 | 0.001 | 1.346 |
| pos | Com_11931 | 3,4-dihydro-2H,6H-[1,3]thiazino[2,3-b]quinazolin-6-one | C11 H10 N2 O S | 240.03 | 1.52 | 241.04 | 2434063.77 | 2322776.79 | 1948320.35 | 2136477.44 | 4132325.25 | 3279173.52 | 14204815.34 | 10838455.33 | 9675691.59 | 12640341.86 | 6772091.75 | 7874106.47 | -1.93 | 0.000 | 1.535 |
| pos | Com_11958 | NNK | C14 H26 N6 O6 | 374.19 | 1.18 | 375.20 | 6312571.50 | 2682702.54 | 4846275.94 | 6470354.05 | 4692012.93 | 6346134.39 | 923443.43 | 875785.44 | 4585163.72 | 2399553.95 | 1079102.18 | 1200500.21 | 1.50 | 0.005 | 1.085 |
| pos | Com_12047 | 2-(2-thienyl)-4H-chromen-4-one | C13 H8 O2 S | 228.02 | 5.48 | 229.03 | 2709956.71 | 3033731.57 | 1533151.48 | 2263793.19 | 3720623.61 | 4799321.69 | 13916495.50 | 10282574.46 | 11175666.63 | 13336342.80 | 11334651.85 | 11858986.13 | -1.99 | 0.000 | 1.300 |
| pos | Com_12185 | Capric acid | C10 H20 O2 | 172.15 | 10.41 | 173.15 | 486040.04 | 1161734.81 | 3166562.32 | 1695172.32 | 1644549.21 | 1375746.76 | 322191.27 | 300862.59 | 284569.82 | 276277.88 | 261312.35 | 244783.12 | 2.50 | 0.001 | 1.008 |
| pos | Com_12192 | Mupirocin | C26 H44 O9 | 482.28 | 15.20 | 483.29 | 4884511.21 | 7856506.75 | 5815048.80 | 6780005.03 | 10291232.31 | 9093042.11 | 1048666.69 | 1690692.32 | 4056145.87 | 539998.02 | 3009839.78 | 733883.03 | 2.01 | 0.003 | 1.503 |
| pos | Com_12303 | Taurolithocholic acid sodium salt | C26 H44 N Na O5 S | 505.28 | 9.00 | 506.29 | 4977250.07 | 5315241.91 | 7084802.70 | 4740859.63 | 9542423.05 | 7088826.61 | 816945.84 | 888714.94 | 1382784.77 | 493201.70 | 1203511.05 | 472309.81 | 2.88 | 0.000 | 1.505 |
| pos | Com_12533 | (5S)-5-hydroxy-1,7-diphenylheptan-3-one | C19 H22 O2 | 264.15 | 14.56 | 265.16 | 5058958.15 | 7784118.99 | 8986895.49 | 8323984.99 | 5830893.75 | 5866575.47 | 3677308.96 | 2339834.59 | 5293427.75 | 2197553.67 | 3160369.00 | 2896850.61 | 1.10 | 0.001 | 1.074 |
| pos | Com_12731 | GMK | C13 H26 N4 O4 S | 334.17 | 1.36 | 335.18 | 406737.51 | 1372356.97 | 359440.48 | 2050388.62 | 768413.22 | 1071162.50 | 10069306.06 | 12247279.32 | 8316630.30 | 4040382.20 | 2608613.39 | 5000852.68 | -2.81 | 0.000 | 1.363 |
| pos | Com_12813 | 3'-Hydroxystanozolol | C21 H32 N2 O2 | 344.24 | 3.34 | 345.25 | 4873488.68 | 3298217.99 | 5934155.99 | 8741950.92 | 3711542.64 | 3763593.56 | 662762.37 | 727093.99 | 1758729.54 | 580175.37 | 816725.88 | 545008.72 | 2.57 | 0.000 | 1.399 |
| pos | Com_12827 | Limonin | C26 H30 O8 | 487.23 | 6.41 | 488.24 | 3747607.78 | 7577482.08 | 4155067.60 | 7703342.23 | 6559855.61 | 5653940.74 | 3094108.23 | 2773956.01 | 1767971.11 | 2829537.10 | 1787867.77 | 2733028.03 | 1.24 | 0.000 | 1.214 |
| pos | Com_13004 | 6 β-Hydroxycortisol | C21 H30 O6 | 720.39 | 10.36 | 361.20 | 1056272.12 | 2547829.03 | 3034371.89 | 2371964.35 | 2889069.80 | 2674317.26 | 8826950.43 | 7003814.77 | 11489120.35 | 9101914.50 | 9579008.92 | 7793084.79 | -1.88 | 0.000 | 1.589 |
| pos | Com_13016 | Milbemycin A3 oxime | C31 H43 N O7 | 541.30 | 13.61 | 542.30 | 155434.07 | 1206218.82 | 1365233.08 | 1507982.09 | 3615733.92 | 1412489.01 | 11029343.76 | 8915647.03 | 7880404.87 | 10623076.26 | 10177087.40 | 12555303.74 | -2.72 | 0.003 | 1.219 |
| pos | Com_13111 | 1-ethyl 4-(2-oxo-1,2-diphenylethyl) succinate | C20 H20 O5 | 362.11 | 1.07 | 363.12 | 2013463.85 | 2474546.12 | 1008929.34 | 1447607.93 | 2003488.25 | 3296328.88 | 6791826.70 | 7243432.84 | 6834183.24 | 7836061.24 | 7904025.12 | 7503460.53 | -1.85 | 0.000 | 1.544 |
| pos | Com_13255 | PLH | C17 H27 N5 O4 | 365.21 | 5.65 | 366.21 | 3926683.99 | 3357927.66 | 5279016.26 | 5914243.20 | 4610979.84 | 3427441.39 | 1561436.00 | 1346597.61 | 3553016.57 | 1647146.52 | 2062888.13 | 1477958.99 | 1.19 | 0.001 | 1.213 |
| pos | Com_13345 | Trenbolone acetate | C20 H24 O3 | 312.17 | 7.71 | 313.18 | 3357562.75 | 3959909.58 | 3961974.60 | 4840635.12 | 5549785.23 | 5322239.90 | 353294.91 | 423178.95 | 704749.93 | 374337.32 | 593661.63 | 421354.20 | 3.23 | 0.000 | 1.630 |
| pos | Com_13376 | PC (3:0/18:5) | C29 H48 N O8 P | 569.31 | 11.07 | 570.32 | 489216.37 | 2617659.13 | 963422.41 | 2670089.90 | 2005166.54 | 925594.18 | 8295734.15 | 9276692.26 | 4966692.26 | 7182976.79 | 8749882.16 | 11924484.38 | -2.38 | 0.001 | 1.217 |
| pos | Com_13488 | ARH | C15 H26 N8 O4 | 382.21 | 0.95 | 192.11 | 5824497.75 | 5145462.80 | 4119157.53 | 6819012.34 | 8261458.85 | 6538732.99 | 1619219.56 | 1539449.17 | 2302055.23 | 1721010.16 | 2707982.29 | 2019369.86 | 1.62 | 0.000 | 1.689 |
| pos | Com_13649 | 2-Hydroxy-6-Aminopurine | C5 H5 N5 O | 151.05 | 1.37 | 152.06 | 813838.37 | 982971.08 | 769202.32 | 845570.48 | 886435.92 | 1420344.51 | 5812695.53 | 11114179.80 | 1408029.29 | 4365696.98 | 3181935.90 | 7133886.39 | -2.53 | 0.002 | 1.509 |
| pos | Com_13691 | IPK | C17 H32 N4 O4 | 178.12 | 5.73 | 179.13 | 5688006.96 | 3515204.39 | 5698401.46 | 7293561.36 | 5550963.36 | 2918991.25 | 2280540.92 | 1911285.14 | 4358218.24 | 1668543.66 | 1955307.53 | 1623133.45 | 1.15 | 0.003 | 1.158 |
| pos | Com_13914 | Taurodeoxycholic Acid | C26 H45 N O6 S | 481.28 | 10.39 | 482.29 | 1332263.28 | 1270298.40 | 1386543.05 | 1598377.68 | 1842390.81 | 1881237.28 | 7639454.39 | 8652671.41 | 10442579.67 | 7070980.22 | 4924474.30 | 5373936.60 | -2.24 | 0.000 | 1.354 |
| pos | Com_14182 | 4-cyclopropyl-6-methoxy-1,3,5-triazin-2-amine | C7 H10 N4 O | 166.09 | 1.36 | 167.09 | 1140012.70 | 2168223.07 | 2584602.71 | 2760318.93 | 6263976.37 | 2611330.05 | 11141021.46 | 9393912.49 | 10145818.87 | 9751105.31 | 3761121.26 | 4910304.29 | -1.49 | 0.004 | 1.083 |
| pos | Com_14253 | Eucalyptol | C10 H18 O | 136.13 | 14.38 | 137.13 | 5736718.00 | 4339754.25 | 3109570.24 | 5335999.57 | 4374651.35 | 3288019.56 | 1565637.61 | 1591028.75 | 2435304.61 | 1422566.18 | 1208392.08 | 1008364.26 | 1.50 | 0.000 | 1.331 |
| pos | Com_14316 | 2-(1H-benzo[d]imidazol-2-yl)-3-(4-toluidino)acrylonitrile | C17 H14 N4 | 274.12 | 6.41 | 275.12 | 463064.95 | 1004204.97 | 544086.45 | 750128.84 | 492250.99 | 789863.95 | 7366118.89 | 10369775.97 | 6990382.29 | 5756727.91 | 5309474.67 | 7063270.14 | -3.41 | 0.000 | 1.480 |
| pos | Com_14623 | 14(15)-EET ethanolamide | C22 H37 N O3 | 385.26 | 11.53 | 386.26 | 1065869.33 | 1632152.72 | 1793965.51 | 2139333.93 | 3126482.12 | 1669451.78 | 7830563.19 | 7767155.54 | 9726753.23 | 9540297.11 | 9690794.42 | 8645187.10 | -2.22 | 0.000 | 1.397 |
| pos | Com_14747 | Debromohymenialdisine | C11 H11 N5 O2 | 267.07 | 6.25 | 268.08 | 207820.56 | 457355.32 | 244823.18 | 574843.98 | 288377.64 | 281564.31 | 10554597.34 | 4833709.92 | 6515770.07 | 4187498.82 | 2750594.53 | 7718245.13 | -4.15 | 0.000 | 1.438 |
| pos | Com_15205 | 4-Butylresorcinol | C10 H14 O2 | 166.10 | 13.85 | 167.11 | 2459702.87 | 2899034.38 | 2975293.16 | 3603038.35 | 2732513.85 | 2580701.48 | 8377202.39 | 9525341.07 | 6527381.28 | 5789088.46 | 3326502.14 | 6740564.97 | -1.22 | 0.002 | 1.694 |
| pos | Com_15235 | SMK | C14 H28 N4 O5 S | 364.18 | 6.54 | 365.19 | 207658.17 | 594947.37 | 279645.38 | 309584.71 | 374939.97 | 546353.42 | 6662129.99 | 9498923.17 | 7221597.70 | 3389811.24 | 3966664.26 | 5242461.35 | -3.96 | 0.000 | 1.413 |
| pos | Com_15340 | 4-Guanidinobutanoic acid | C5 H11 N3 O2 | 145.09 | 1.80 | 146.09 | 503929.19 | 1149672.21 | 760860.45 | 574969.38 | 1232458.34 | 1889919.74 | 3392708.97 | 4108267.79 | 3631346.43 | 3821392.70 | 5978888.12 | 5450478.90 | -2.11 | 0.000 | 1.129 |
| pos | Com_15439 | IKK | C18 H37 N5 O4 | 193.64 | 1.37 | 194.65 | 5133674.62 | 3122999.82 | 5523231.86 | 5222666.73 | 4168483.31 | 3063250.32 | 1345628.34 | 1799550.67 | 2558707.37 | 1440428.76 | 2059768.79 | 1663608.35 | 1.27 | 0.000 | 1.470 |
| pos | Com_15451 | (2,6-dimethylmorpholino)(1-methyl-5-nitro-1H-pyrazol-4-yl)methanone | C11 H16 N4 O4 | 250.11 | 1.36 | 539.17 | 2148974.19 | 3267483.57 | 2116593.22 | 2322047.95 | 3576009.89 | 4853309.79 | 5794484.03 | 9333533.65 | 5397072.01 | 5532506.82 | 6062709.47 | 6617313.95 | -1.08 | 0.001 | 1.171 |
| pos | Com_15508 | GKK | C14 H29 N5 O4 | 165.61 | 0.90 | 166.62 | 4049939.34 | 1712397.03 | 4557195.06 | 6119892.79 | 7355993.65 | 4388655.85 | 1219665.80 | 1846972.35 | 2656809.70 | 1571191.32 | 1882438.82 | 2384816.98 | 1.29 | 0.008 | 1.252 |
| pos | Com_15645 | Glycolithocholic acid | C26 H43 N O4 | 433.32 | 14.45 | 434.33 | 2551960.95 | 4921558.38 | 4641042.44 | 4571361.38 | 4739400.38 | 4374334.90 | 1135637.01 | 1085755.22 | 1227581.93 | 907146.64 | 1391851.05 | 1453187.13 | 1.84 | 0.000 | 1.131 |
| pos | Com_15695 | 7-(2-hydroxypropan-2-yl)-1,4a-dimethyl-decahydronaphthalen-1-ol | C15 H28 O2 | 222.20 | 13.50 | 223.21 | 736594.00 | 1344396.33 | 1892158.87 | 1341680.52 | 1145877.27 | 1064562.28 | 4493048.63 | 5485464.61 | 8224878.03 | 3419686.84 | 3581178.73 | 7229919.75 | -2.11 | 0.000 | 1.529 |
| pos | Com_15856 | Quinoline | C9 H7 N | 129.06 | 10.51 | 130.07 | 1377022.83 | 1388703.09 | 2211077.86 | 1968142.63 | 1483162.45 | 1784636.54 | 9122032.82 | 7904063.84 | 8687254.65 | 8647632.69 | 2388787.16 | 3553413.05 | -1.98 | 0.002 | 1.424 |
| pos | Com_16055 | 4-benzyl-1-[(3,4-dimethoxyphenyl)sulfonyl]piperidine | C20 H25 N O4 S | 375.15 | 1.90 | 376.15 | 237995.97 | 2129407.66 | 1514278.49 | 1761247.71 | 1568777.09 | 1473219.78 | 5938804.31 | 7602385.99 | 8507013.04 | 4679427.63 | 4970905.04 | 6711793.06 | -2.14 | 0.003 | 1.171 |
| pos | Com_16097 | Asp-Phe | C13 H16 N2 O5 | 280.11 | 8.35 | 281.11 | 2338901.22 | 2824415.90 | 3382413.12 | 3256885.53 | 4103404.63 | 4342697.84 | 8585309.01 | 6464687.18 | 8427876.35 | 8865595.35 | 6540451.91 | 6556389.50 | -1.17 | 0.000 | 1.169 |
| pos | Com_16384 | 3-{(Z)-[3-(2-thienyl)-1H-pyrazol-4-yl]methylidene}-1H-indol-2-one | C16 H11 N3 O S | 293.06 | 6.63 | 294.07 | 156585.88 | 263152.92 | 140069.90 | 179167.76 | 255149.29 | 231131.05 | 4120925.34 | 3557355.70 | 8262767.09 | 2477407.58 | 1523054.60 | 1743018.26 | -4.15 | 0.000 | 1.474 |
| pos | Com_16428 | Prostaglandin B2 | C20 H30 O4 | 334.21 | 13.15 | 686.46 | 1325649.67 | 6860525.78 | 5755275.00 | 4471177.23 | 3007296.20 | 5852239.89 | 2755800.34 | 2003583.17 | 1781726.05 | 1683426.70 | 681827.01 | 1913669.25 | 1.33 | 0.021 | 1.091 |
| pos | Com_16522 | (9cis)-Retinal | C20 H28 O | 284.21 | 12.76 | 285.22 | 1815764.77 | 4002623.23 | 4479986.20 | 4260248.22 | 2233857.19 | 2375029.05 | 1526068.56 | 1358874.61 | 891448.15 | 1181035.08 | 1495882.10 | 1045868.28 | 1.35 | 0.001 | 1.138 |
| pos | Com_16607 | Deoxycytidine | C9 H13 N3 O4 | 227.09 | 1.26 | 228.10 | 1362071.21 | 2838486.92 | 1585724.49 | 2218638.07 | 2525807.24 | 2588386.37 | 6203160.99 | 5616055.45 | 8130444.21 | 6742732.29 | 4369919.06 | 3316443.78 | -1.39 | 0.000 | 1.215 |
| pos | Com_16717 | (+/-)17(18)-EpETE methyl ester | C21 H32 O3 | 349.26 | 11.65 | 350.27 | 2498892.69 | 2313532.83 | 3980429.19 | 2318862.15 | 2893207.29 | 1957327.67 | 594669.02 | 585606.72 | 1029986.20 | 1029075.04 | 926647.36 | 772490.42 | 1.69 | 0.000 | 1.137 |
| pos | Com_16736 | Pantetheine | C11 H22 N2 O4 S | 278.13 | 1.20 | 279.14 | 741860.11 | 964262.32 | 381893.14 | 687024.76 | 1117436.74 | 1430955.07 | 6997083.77 | 7242279.92 | 4553074.43 | 8383309.35 | 4099693.88 | 5858046.48 | -2.80 | 0.000 | 1.539 |
| pos | Com_16822 | QKK | C17 H34 N6 O5 | 402.26 | 0.90 | 202.14 | 4514282.76 | 2262916.98 | 3334400.91 | 4266687.90 | 3359353.82 | 2911190.56 | 628749.62 | 722325.48 | 1037672.66 | 680475.53 | 1184506.39 | 896764.50 | 2.00 | 0.000 | 1.765 |
| pos | Com_17042 | Hydrocortisone | C21 H30 O5 | 362.21 | 10.29 | 363.22 | 961402.44 | 1427673.62 | 2459665.18 | 2003124.99 | 2113810.99 | 1885098.88 | 6339008.35 | 5404658.43 | 7779625.14 | 6025098.33 | 5786122.91 | 5828551.55 | -1.78 | 0.000 | 1.653 |
| pos | Com_17154 | Taurochenodeoxycholic acid | C26 H45 N O6 S | 481.28 | 10.64 | 482.29 | 1018829.89 | 1003733.84 | 935940.68 | 1126671.57 | 851555.98 | 1064423.18 | 3783224.14 | 2816890.07 | 7698612.50 | 2883165.06 | 2055377.26 | 2715766.09 | -1.87 | 0.001 | 1.332 |
| pos | Com_17202 | GPK | C13 H24 N4 O4 | 150.09 | 1.15 | 301.19 | 2916272.47 | 2291827.33 | 2717990.19 | 4008142.46 | 5075915.46 | 6259163.77 | 1203877.51 | 1169866.21 | 1210068.68 | 1322877.83 | 1470763.33 | 1579870.04 | 1.55 | 0.001 | 1.431 |
| pos | Com_17272 | 4-methyl-2-oxo-1,2-dihydroquinoline-3-carbonitrile | C11 H8 N2 O | 184.06 | 9.31 | 185.07 | 832489.76 | 1491108.02 | 2388310.34 | 1851682.63 | 1581072.99 | 1732368.47 | 4253401.10 | 4755101.51 | 4057570.10 | 4137937.18 | 4106703.15 | 4708386.58 | -1.40 | 0.001 | 1.279 |
| pos | Com_17787 | RNK | C16 H32 N8 O5 | 416.25 | 0.92 | 209.13 | 4144679.53 | 2374601.44 | 3184911.45 | 4579523.79 | 3441746.43 | 4239865.22 | 1322153.10 | 1535671.84 | 1907070.28 | 1923981.42 | 2244240.86 | 1708157.77 | 1.05 | 0.000 | 1.432 |
| pos | Com_17832 | LNH | C16 H26 N6 O5 | 365.17 | 5.47 | 366.18 | 1702770.62 | 4378113.89 | 2240956.62 | 4570400.16 | 5188671.32 | 4299254.90 | 492901.71 | 432120.42 | 677845.41 | 346690.61 | 570143.35 | 471269.51 | 2.90 | 0.000 | 1.725 |
| pos | Com_17868 | 1,3-dipyridin-3-ylpropane-1,3-dione | C13 H10 N2 O2 | 226.07 | 8.65 | 227.08 | 1584557.12 | 2091144.86 | 3203746.83 | 2020744.90 | 2175794.21 | 2308013.68 | 7966552.63 | 6526646.82 | 5764148.35 | 6734813.36 | 4946091.91 | 5723096.38 | -1.49 | 0.000 | 1.763 |
| pos | Com_17877 | 1-[2-(2,5-dimethyl-1H-pyrrol-1-yl)-4-nitrophenyl]-1H-imidazole | C15 H14 N4 O2 | 282.11 | 8.35 | 283.12 | 1138347.17 | 4014068.56 | 6129562.43 | 5103959.28 | 1843903.38 | 2450498.72 | 344859.19 | 316059.20 | 398309.71 | 107265.65 | 1313918.43 | 718393.86 | 2.69 | 0.001 | 1.402 |
| pos | Com_17896 | RNH | C16 H27 N9 O5 | 212.61 | 0.93 | 213.61 | 2559147.42 | 4055576.68 | 4663706.47 | 4348667.69 | 5924373.89 | 4845667.43 | 1157098.52 | 1786654.08 | 2362809.67 | 2016945.28 | 2696595.58 | 1611602.34 | 1.18 | 0.001 | 1.596 |
| pos | Com_18096 | YLH | C21 H29 N5 O5 | 431.22 | 7.27 | 432.22 | 1935218.61 | 1948289.92 | 1690258.94 | 2041132.74 | 5825988.57 | 3430372.87 | 597335.08 | 655177.95 | 462936.62 | 459937.26 | 621411.50 | 658965.85 | 2.29 | 0.000 | 1.405 |
| pos | Com_18115 | 4-decyl-3-hydroxy-5-oxooxolane-2,3-dicarboxylic acid | C16 H26 O7 | 352.15 | 13.69 | 353.16 | 827160.12 | 2116701.42 | 2117568.48 | 1000665.62 | 2664155.38 | 2731156.32 | 4937321.24 | 4839297.43 | 2437149.61 | 4590202.04 | 4408086.02 | 5280538.75 | -1.21 | 0.006 | 1.009 |
| pos | Com_18211 | Prostaglandin F2α-1-glyceryl ester | C23 H40 O7 | 450.26 | 5.54 | 451.27 | 1998038.04 | 1581157.72 | 3021873.32 | 2860380.56 | 5776037.60 | 3287971.73 | 1330216.74 | 1241973.76 | 2061828.98 | 1556717.53 | 1747507.80 | 1249901.50 | 1.01 | 0.016 | 1.040 |
| pos | Com_18504 | Gatifloxacin | C19 H22 F N3 O4 | 357.15 | 6.32 | 358.16 | 1104065.48 | 1783941.24 | 2179575.82 | 1796673.75 | 1354809.95 | 1655451.57 | 6405146.01 | 5762431.51 | 6886869.18 | 4254882.45 | 3143881.98 | 4500373.26 | -1.65 | 0.000 | 1.310 |
| pos | Com_18771 | diethyl 2-[(4-benzhydrylpiperidino)methylidene]malonate | C26 H31 N O4 | 443.20 | 2.20 | 444.21 | 744021.46 | 1795124.93 | 1770406.19 | 1509290.58 | 1341919.32 | 1325757.99 | 6345045.88 | 6376686.33 | 5884932.17 | 7052406.35 | 1736270.86 | 4295169.37 | -1.90 | 0.001 | 1.480 |
| pos | Com_18792 | CYM-5442 | C23 H27 N3 O4 | 1636.82 | 8.53 | 819.42 | 2718494.11 | 2826282.23 | 2808554.62 | 3781991.34 | 3410763.98 | 2823985.38 | 558529.40 | 686363.53 | 545896.94 | 467815.18 | 188651.28 | 460729.86 | 2.66 | 0.000 | 1.654 |
| pos | Com_19205 | ELK | C17 H32 N4 O6 | 388.23 | 1.73 | 389.24 | 2523359.41 | 1060899.55 | 2611846.58 | 2708021.48 | 1946812.21 | 1379648.05 | 587288.37 | 251804.34 | 759180.18 | 262446.62 | 321101.58 | 316863.38 | 2.29 | 0.000 | 1.417 |
| pos | Com_19409 | Coenzyme Q2 | C19 H26 O4 | 318.18 | 10.64 | 319.19 | 874417.82 | 1423396.53 | 2018073.56 | 2049859.83 | 1366710.33 | 1502598.67 | 5884714.75 | 5210903.43 | 4360454.99 | 5366225.67 | 5629261.46 | 5711250.67 | -1.80 | 0.000 | 1.654 |
| pos | Com_19443 | SKK | C15 H31 N5 O5 | 361.23 | 0.90 | 181.62 | 3635196.98 | 2956788.24 | 3624282.22 | 4232596.53 | 4351748.35 | 3993557.59 | 775495.91 | 693093.25 | 908675.71 | 951920.32 | 808375.95 | 1178051.52 | 2.10 | 0.000 | 1.874 |
| pos | Com_19840 | N-(4-piperidinophenyl)-2-thiophenecarboxamide | C16 H18 N2 O S | 308.09 | 10.97 | 309.10 | 223909.81 | 275782.75 | 198318.84 | 302909.41 | 316361.24 | 370882.38 | 6807115.86 | 4485734.44 | 3135440.80 | 6371135.53 | 2756965.98 | 3722750.12 | -4.01 | 0.000 | 1.534 |
| pos | Com_20340 | (+/-)5(6)-EET Ethanolamide | C22 H37 N O3 | 363.28 | 11.28 | 364.28 | 1549479.82 | 2333470.49 | 1652824.83 | 2692806.18 | 3879597.68 | 3538777.57 | 671384.58 | 449812.28 | 866778.81 | 577104.09 | 917054.44 | 960837.97 | 1.82 | 0.000 | 1.366 |
| pos | Com_20417 | Panthenol | C9 H19 N O4 | 205.13 | 6.39 | 206.14 | 1094480.23 | 1330239.58 | 1321023.51 | 1167575.78 | 2802794.81 | 2389922.22 | 5169023.26 | 3427971.05 | 3119313.87 | 3675867.74 | 2989652.51 | 2777645.96 | -1.07 | 0.003 | 1.262 |
| pos | Com_20540 | 1-benzyl-3-butyl-4-hydroxy-6-phenylpyridin-2(1H)-one | C22 H23 N O2 | 333.17 | 7.63 | 334.18 | 2955008.22 | 1839455.11 | 2397878.33 | 2741854.43 | 2898146.71 | 2479382.77 | 527111.36 | 674771.62 | 942752.78 | 501029.26 | 841319.10 | 607689.80 | 1.90 | 0.000 | 1.680 |
| pos | Com_20622 | VLH | C17 H29 N5 O4 | 367.22 | 2.47 | 368.23 | 831166.92 | 1558477.45 | 1117063.45 | 1615831.72 | 3040942.89 | 1636034.98 | 157247.85 | 285567.11 | 556845.17 | 192371.15 | 161225.26 | 197143.50 | 2.66 | 0.000 | 1.571 |
| pos | Com_20661 | Enalaprilat | C18 H24 N2 O5 | 348.18 | 1.03 | 349.18 | 1247541.14 | 1507176.67 | 1467362.87 | 1415319.85 | 1532836.52 | 937057.03 | 260381.68 | 436636.48 | 542456.41 | 385898.71 | 842846.16 | 280856.02 | 1.56 | 0.001 | 1.296 |
| pos | Com_20757 | trans-Anethole | C10 H12 O | 148.09 | 14.65 | 149.10 | 3290170.62 | 2309827.33 | 1250276.39 | 2655054.55 | 1286259.32 | 1880197.89 | 471159.70 | 331370.60 | 504644.32 | 479426.47 | 536011.63 | 480493.07 | 2.18 | 0.000 | 1.580 |
| pos | Com_20764 | 2-morpholino-1-phenyl-1-ethanol | C12 H17 N O2 | 207.13 | 6.08 | 208.13 | 162719.05 | 182489.16 | 264540.76 | 209974.13 | 224531.00 | 132582.25 | 3721035.85 | 1124392.92 | 928651.02 | 1682462.63 | 782871.50 | 647788.09 | -2.92 | 0.000 | 1.392 |
| pos | Com_20774 | Methylmalonate | C4 H6 O4 | 118.03 | 1.44 | 119.03 | 729684.88 | 1310014.34 | 1024299.32 | 1309079.24 | 1338602.70 | 1153843.57 | 2694001.18 | 3182348.79 | 2220390.27 | 2443783.77 | 6587348.92 | 4258807.86 | -1.64 | 0.000 | 1.522 |
| pos | Com_20810 | ALK | C15 H30 N4 O4 | 330.23 | 7.18 | 331.23 | 1753564.39 | 1290562.96 | 1778345.03 | 2151444.98 | 1386879.63 | 1115324.80 | 328055.96 | 276458.47 | 931004.41 | 324728.35 | 968538.48 | 489836.79 | 1.51 | 0.002 | 1.145 |
| pos | Com_20853 | 1-morpholino-3-(4-nitrophenoxy)propan-2-ol | C13 H18 N2 O5 | 282.12 | 8.59 | 283.13 | 3228698.52 | 2957886.40 | 2687063.73 | 4329797.87 | 3726848.60 | 3724176.25 | 1096951.98 | 1106376.33 | 1251501.41 | 902410.07 | 1194541.49 | 975215.55 | 1.66 | 0.000 | 1.632 |
| pos | Com_21220 | SPK | C14 H26 N4 O5 | 330.19 | 2.00 | 331.20 | 1814328.14 | 4358455.19 | 3432130.96 | 4205020.73 | 1429293.90 | 3514186.26 | 458845.02 | 619143.50 | 889074.59 | 582547.58 | 1139147.05 | 602864.77 | 2.13 | 0.000 | 1.572 |
| pos | Com_21440 | Verrucarol | C15 H22 O4 | 266.15 | 8.16 | 267.16 | 343090.48 | 138286.01 | 118200.90 | 133788.01 | 226422.67 | 148656.67 | 1696932.78 | 881844.19 | 1092908.38 | 1167254.06 | 1135458.59 | 902938.07 | -2.63 | 0.000 | 1.307 |
| pos | Com_21480 | 8-Aminooctanoic acid | C8 H17 N O2 | 159.13 | 1.37 | 160.13 | 1363479.38 | 1732151.68 | 1953793.42 | 1684430.48 | 2436598.84 | 2693510.62 | 4882624.49 | 5651364.55 | 4009631.59 | 5052250.72 | 5654662.39 | 4900962.37 | -1.35 | 0.000 | 1.609 |
| pos | Com_21602 | MNK | C15 H29 N5 O5 S | 373.17 | 5.73 | 374.18 | 2162921.42 | 1206100.35 | 2260112.55 | 2616375.58 | 1858771.84 | 1318723.12 | 177236.31 | 307390.14 | 775470.05 | 254675.62 | 247773.48 | 202661.81 | 2.54 | 0.000 | 1.385 |
| pos | Com_22150 | PNK | C15 H27 N5 O5 | 339.19 | 1.83 | 340.20 | 1468351.76 | 1023667.02 | 1663349.65 | 1832581.72 | 1730807.70 | 1113429.85 | 268425.02 | 261166.39 | 785177.36 | 320630.74 | 329918.28 | 344472.78 | 1.94 | 0.000 | 1.174 |
| pos | Com_22216 | 4-[4-chloro-2-nitro-5-(1H-pyrrol-1-yl)phenyl]morpholine | C14 H14 Cl N3 O3 | 307.07 | 9.03 | 308.08 | 216861.16 | 298135.90 | 263713.82 | 268312.69 | 290718.83 | 304445.48 | 5681761.50 | 3835227.91 | 3978735.76 | 2763070.31 | 1400579.51 | 1956144.94 | -3.58 | 0.000 | 1.687 |
| pos | Com_22392 | All trans-Retinal | C20 H28 O | 284.21 | 13.35 | 285.22 | 2898072.08 | 2416742.05 | 838093.09 | 2444816.19 | 925672.52 | 1478060.79 | 543541.10 | 576279.62 | 452845.22 | 367357.15 | 613223.57 | 558408.81 | 1.82 | 0.002 | 1.463 |
| pos | Com_22500 | 1-(4-benzylpiperazino)-2-(pyridin-2-ylamino)propan-1-one | C19 H24 N4 O | 302.21 | 9.92 | 303.22 | 802197.14 | 455998.56 | 1130406.82 | 1214094.79 | 1006074.53 | 753397.31 | 3198367.13 | 2839205.84 | 3212927.54 | 2866892.16 | 4532968.75 | 1679680.80 | -1.77 | 0.000 | 1.366 |
| pos | Com_22722 | VPH | C16 H25 N5 O4 | 351.20 | 6.95 | 352.20 | 1025711.03 | 580967.46 | 1144599.31 | 581030.87 | 683344.57 | 577385.87 | 229666.65 | 85579.23 | 690996.44 | 93210.18 | 192752.86 | 145528.02 | 1.68 | 0.005 | 1.046 |
| pos | Com_22723 | PE (2:0/16:2) | C23 H42 N O8 P | 491.27 | 12.27 | 492.27 | 877217.75 | 1593108.41 | 2457103.97 | 1811026.98 | 1364241.20 | 994356.70 | 3992655.46 | 4099899.58 | 2292294.87 | 3918421.56 | 4975838.63 | 3924716.58 | -1.35 | 0.001 | 1.171 |
| pos | Com_22855 | Pantethine | C22 H42 N4 O8 S2 | 554.24 | 8.36 | 555.25 | 1164980.62 | 878862.89 | 1102323.91 | 397367.24 | 1768355.70 | 1239355.11 | 445765.57 | 182828.41 | 215156.55 | 270883.08 | 385461.85 | 231257.41 | 1.92 | 0.001 | 1.105 |
| pos | Com_23082 | HNH | C16 H22 N8 O5 | 184.11 | 10.72 | 407.18 | 241806.98 | 1028777.02 | 237440.04 | 929779.36 | 224575.30 | 687569.82 | 2893634.54 | 2142096.17 | 4835331.35 | 1621316.17 | 1882985.07 | 2298197.83 | -2.23 | 0.001 | 1.222 |
| pos | Com_23310 | Sulfamethazine | C12 H14 N4 O2 S | 278.08 | 6.17 | 279.09 | 154229.24 | 263206.32 | 251082.83 | 178466.75 | 181395.15 | 226005.86 | 5219889.95 | 2179898.01 | 1724189.37 | 3764271.77 | 786156.43 | 1922002.53 | -3.64 | 0.000 | 1.728 |
| pos | Com_23390 | 6β-Oxycodol N-oxide | C18 H23 N O5 | 333.15 | 10.44 | 334.16 | 1328501.95 | 700012.38 | 733286.84 | 1830292.18 | 1508910.26 | 997151.81 | 767966.34 | 446088.08 | 303504.19 | 286263.55 | 267184.12 | 167253.18 | 1.67 | 0.001 | 1.111 |
| pos | Com_23516 | N-Acetylsphingosine | C20 H39 N O3 | 363.28 | 10.55 | 364.28 | 1382140.65 | 1988005.85 | 1584840.43 | 2199025.67 | 3123177.29 | 2506333.05 | 541129.42 | 522899.73 | 410893.48 | 463641.35 | 530120.66 | 406993.19 | 2.15 | 0.000 | 1.655 |
| pos | Com_23975 | 5-(hydroxymethyl)-4-methoxy-2,5-dihydrofuran-2-one | C6 H8 O4 | 166.02 | 3.53 | 167.03 | 1314074.83 | 2868513.57 | 3751114.49 | 3414866.08 | 1689710.96 | 2528522.22 | 473829.99 | 490250.90 | 219678.59 | 426927.12 | 558246.78 | 386420.03 | 2.61 | 0.000 | 1.420 |
| pos | Com_24211 | N-METHYL (-)EPHEDRINE | C11 H17 N O | 179.13 | 11.44 | 180.14 | 729734.20 | 2493609.72 | 1231570.09 | 2518826.79 | 1174450.90 | 1496663.29 | 551788.56 | 454374.65 | 428404.22 | 597951.30 | 575409.38 | 569902.19 | 1.60 | 0.003 | 1.154 |
| pos | Com_24217 | Lapachol | C15 H14 O3 | 264.08 | 2.82 | 265.09 | 2530230.52 | 2044022.63 | 3603079.23 | 3218198.14 | 2624609.44 | 2357665.91 | 364937.34 | 418860.99 | 1118211.50 | 500326.56 | 626429.97 | 482830.98 | 2.22 | 0.000 | 1.496 |
| pos | Com_24414 | Triiodothyronine | C15 H12 I3 N O4 | 650.79 | 1.10 | 651.80 | 892350.30 | 1036352.96 | 1254353.31 | 825510.75 | 1012338.42 | 1326092.53 | 4238209.05 | 2654514.13 | 4390432.00 | 2276248.14 | 1875092.24 | 2119759.07 | -1.47 | 0.000 | 1.335 |
| pos | Com_24435 | Andrographolide | C20 H30 O5 | 350.21 | 11.22 | 351.22 | 638128.85 | 1296038.28 | 2935203.72 | 1763187.19 | 1172863.78 | 1252512.47 | 3104834.35 | 2867578.09 | 4385239.12 | 3996839.69 | 2986477.42 | 3147039.15 | -1.18 | 0.005 | 1.406 |
| pos | Com_24717 | (±)8-HEPE | C20 H30 O3 | 300.21 | 11.62 | 301.22 | 723285.00 | 1627075.14 | 2285525.69 | 1477207.43 | 1206155.99 | 1479794.29 | 3417855.35 | 2790964.63 | 3382823.23 | 2878166.31 | 3652301.62 | 4391467.54 | -1.22 | 0.001 | 1.171 |
| pos | Com_24718 | Tetranor-12(S)-HETE | C16 H26 O3 | 288.17 | 10.94 | 289.18 | 1008915.34 | 1096696.57 | 1739540.39 | 1924956.93 | 1788171.96 | 1271249.67 | 2591425.09 | 2256461.84 | 3654228.15 | 3207920.81 | 4908404.72 | 3743862.35 | -1.21 | 0.000 | 1.508 |
| pos | Com_25290 | KQH | C17 H29 N7 O5 | 205.61 | 0.94 | 206.62 | 2354133.57 | 2686808.70 | 2332355.52 | 2419103.02 | 1722694.77 | 2076217.27 | 488864.17 | 460420.81 | 527406.33 | 382444.16 | 479268.32 | 386198.92 | 2.32 | 0.000 | 1.772 |
| pos | Com_25515 | 4-[2-(4-chlorophenyl)diaz-1-enyl]-2-methyl-6-(piperidinomethyl)phenol | C19 H22 Cl N3 O | 343.15 | 2.13 | 344.16 | 446783.63 | 1266583.80 | 936735.20 | 1501533.43 | 839025.15 | 1280181.98 | 3770780.83 | 3089290.13 | 4075683.07 | 3182519.43 | 2428367.36 | 2906608.43 | -1.63 | 0.001 | 1.110 |
| pos | Com_25785 | beta-Estradiol 17-Acetate | C20 H26 O3 | 314.19 | 11.65 | 315.20 | 668564.59 | 829400.44 | 2211983.97 | 1276438.87 | 789843.89 | 742368.15 | 3001322.21 | 2623866.62 | 4001196.09 | 2719194.25 | 1378623.49 | 2807798.14 | -1.34 | 0.002 | 1.397 |
| pos | Com_25940 | Homo-Gamma-Linolenic Acid (C20:3) | C20 H34 O2 | 306.26 | 13.73 | 307.26 | 2251427.91 | 1160165.92 | 547475.94 | 683051.31 | 860528.72 | 1390291.18 | 451476.12 | 411626.40 | 397269.50 | 547378.04 | 420175.69 | 475469.44 | 1.35 | 0.010 | 1.116 |
| pos | Com_26716 | 3-(2-methylpropyl)-octahydropyrrolo[1,2-a]pyrazine-1,4-dione | C11 H18 N2 O2 | 232.12 | 8.56 | 215.12 | 609519.12 | 1030424.19 | 1415799.14 | 1250699.96 | 878667.92 | 1061754.26 | 3088211.82 | 2865565.64 | 3765082.53 | 3473196.35 | 3301057.10 | 3650482.07 | -1.69 | 0.000 | 1.446 |
| pos | Com_26801 | (1E,4E)-1,5-bis(4-methoxyphenyl)penta-1,4-dien-3-one | C19 H18 O3 | 316.11 | 9.51 | 317.11 | 124395.79 | 357943.24 | 344284.96 | 397751.69 | 380632.87 | 413845.05 | 2858768.74 | 2556412.28 | 2483057.49 | 2299839.99 | 1705679.53 | 2014305.94 | -2.79 | 0.000 | 1.405 |
| pos | Com_27266 | N-(5-acetamidopentyl)acetamide | C9 H18 N2 O2 | 208.12 | 7.78 | 209.13 | 439853.42 | 668896.76 | 1284289.02 | 1174405.11 | 662601.15 | 954566.93 | 2457655.02 | 2560614.25 | 3053525.08 | 2426477.62 | 3723115.33 | 3204087.85 | -1.75 | 0.000 | 1.373 |
| pos | Com_27424 | QMK | C16 H31 N5 O5 S | 810.40 | 5.22 | 406.21 | 589433.27 | 1066304.14 | 2091508.30 | 1539755.80 | 1584558.01 | 1860570.26 | 114036.88 | 126248.26 | 144091.04 | 95499.44 | 170861.78 | 124364.23 | 3.49 | 0.000 | 1.371 |
| pos | Com_27484 | (3S,9aS)-3-benzyl-octahydro-1H-pyrido[1,2-a]pyrazin-1-one | C15 H20 N2 O | 266.14 | 0.98 | 267.15 | 1192392.59 | 1379483.32 | 2203298.86 | 1654104.33 | 854383.81 | 1914297.74 | 427656.76 | 262489.48 | 411020.56 | 235391.95 | 285063.58 | 572333.62 | 2.07 | 0.000 | 1.404 |
| pos | Com_27506 | Desmethylclozapine | C17 H17 Cl N4 | 312.12 | 1.05 | 313.13 | 1584004.60 | 1555240.76 | 1657888.85 | 2425250.60 | 2015523.94 | 1814908.50 | 759778.41 | 661000.65 | 940640.83 | 701986.52 | 779055.97 | 1060531.06 | 1.17 | 0.000 | 1.452 |
| pos | Com_27772 | 2-[(3S)-1-Cyclobutyl-3-pyrrolidinyl]-1H-benzimidazole-5-carbonitrile | C16 H18 N4 | 266.15 | 16.95 | 267.16 | 573373.77 | 1215723.96 | 922511.97 | 964392.41 | 732617.83 | 854111.60 | 603451.28 | 377761.56 | 297571.65 | 295585.36 | 305929.22 | 258720.07 | 1.30 | 0.000 | 1.096 |
| pos | Com_27932 | tetranor-12(R)-HETE | C16 H26 O3 | 248.18 | 14.04 | 249.19 | 1981188.24 | 1609397.60 | 835132.10 | 953049.67 | 699225.82 | 1260848.82 | 305209.01 | 469049.17 | 336225.33 | 249191.91 | 487412.00 | 485643.82 | 1.65 | 0.000 | 1.366 |
| pos | Com_28907 | Cnidioside A | C17 H20 O9 | 385.13 | 7.00 | 386.14 | 305562.05 | 1171356.85 | 781244.55 | 1264937.84 | 984720.67 | 1261025.13 | 154572.85 | 145394.41 | 184619.33 | 194697.76 | 242681.11 | 155882.23 | 2.42 | 0.000 | 1.326 |
| pos | Com_29422 | morpholino(quinolin-6-yl)methanone | C14 H14 N2 O2 | 242.11 | 7.84 | 243.11 | 531068.20 | 628094.21 | 565178.83 | 472388.29 | 682524.42 | 729781.01 | 3017810.07 | 2996924.70 | 1780456.65 | 2885937.66 | 2009682.58 | 2196503.87 | -2.04 | 0.000 | 1.616 |
| pos | Com_29928 | Ala-trp | C14 H17 N3 O3 | 275.13 | 6.51 | 276.13 | 945868.43 | 1518651.78 | 1058722.51 | 2293479.40 | 1105288.67 | 1455770.37 | 606336.15 | 591514.18 | 957525.84 | 612608.85 | 696429.27 | 436430.08 | 1.10 | 0.001 | 1.209 |
| pos | Com_30065 | 1-(4-nitrophenyl)piperidine | C11 H14 N2 O2 | 206.11 | 8.07 | 207.11 | 401582.22 | 658556.64 | 951239.84 | 834773.24 | 714290.20 | 700957.19 | 1887881.07 | 1970905.84 | 2806382.43 | 2398634.64 | 2630932.42 | 3321206.91 | -1.82 | 0.000 | 1.406 |
| pos | Com_30298 | LPC 18:3 | C26 H48 N O7 P | 517.32 | 13.98 | 518.32 | 127238.77 | 404654.13 | 328350.72 | 437063.92 | 365930.09 | 387063.16 | 468100.22 | 1363308.64 | 1604560.71 | 3124395.93 | 1226323.69 | 587667.92 | -2.03 | 0.005 | 1.211 |
| pos | Com_30616 | Amikacin | C22 H43 N5 O13 | 585.28 | 6.29 | 586.28 | 440349.36 | 516310.22 | 714378.46 | 658220.34 | 520918.37 | 802897.62 | 1614987.07 | 2224534.80 | 1636581.26 | 3065094.82 | 2263574.15 | 2071368.13 | -1.82 | 0.000 | 1.591 |
| pos | Com_30875 | ethyl 3-[2-(tert-butyl)-4-(1,3-thiazol-2-yl)phenyl]-2-cyanoacrylate | C19 H20 N2 O2 S | 318.14 | 7.85 | 319.15 | 259179.95 | 240878.86 | 349416.34 | 390877.46 | 502002.86 | 460114.15 | 2062305.43 | 2966031.64 | 2726504.82 | 2651065.31 | 2441913.07 | 3164865.41 | -2.86 | 0.000 | 1.572 |
| pos | Com_30905 | Palmitoylcarnitine | C23 H45 N O4 | 399.33 | 11.41 | 400.34 | 1381785.41 | 643758.18 | 803305.58 | 2179823.35 | 427534.94 | 287032.53 | 297810.48 | 222801.17 | 225140.77 | 230828.82 | 225043.02 | 307586.74 | 1.92 | 0.014 | 1.224 |
| pos | Com_31114 | 1-[(3,5-dimethylisoxazol-4-yl)sulfonyl]piperidine | C10 H16 N2 O3 S | 244.08 | 6.55 | 245.09 | 1218395.58 | 1252082.07 | 757909.36 | 2146838.43 | 784964.01 | 1302449.84 | 478833.93 | 415610.43 | 723378.66 | 340360.20 | 610517.15 | 514625.74 | 1.28 | 0.002 | 1.400 |
| pos | Com_31608 | N-(5-methyl-3-isoxazolyl)-2,3-dihydro-1-benzofuran-5-sulfonamide | C12 H12 N2 O4 S | 280.05 | 3.74 | 281.06 | 1094135.96 | 1132996.69 | 1402436.39 | 1206899.80 | 1347494.50 | 1377128.52 | 2277774.20 | 2534422.30 | 2295890.55 | 2881122.75 | 2618891.62 | 2636904.37 | -1.01 | 0.000 | 1.024 |
| pos | Com_32232 | 3-hydroxy-1,5-diphenylpentan-1-one | C17 H18 O2 | 236.12 | 7.38 | 237.13 | 520922.06 | 297164.09 | 697775.74 | 540130.90 | 273314.69 | 327182.74 | 106905.94 | 96234.08 | 99984.17 | 99970.77 | 102572.04 | 93427.41 | 2.15 | 0.000 | 1.156 |
| pos | Com_32666 | 1-[4-(1-adamantyl)phenoxy]-3-piperidinopropan-2-ol hydrochloride | C24 H35 N O2 | 369.27 | 14.12 | 370.27 | 809766.25 | 712169.56 | 848410.31 | 946668.20 | 591750.31 | 510748.17 | 175343.68 | 210470.17 | 438550.83 | 151533.59 | 278775.55 | 183935.07 | 1.62 | 0.000 | 1.089 |
| pos | Com_33167 | MLK | C17 H34 N4 O4 S | 372.22 | 7.05 | 373.23 | 680249.44 | 1133294.42 | 1052136.65 | 1512138.82 | 485990.74 | 1355161.60 | 412989.26 | 330436.66 | 504087.88 | 278310.02 | 473905.68 | 257964.83 | 1.46 | 0.001 | 1.250 |
| pos | Com_33621 | Deflazacort | C25 H31 N O6 | 441.21 | 8.45 | 442.22 | 173735.01 | 253386.48 | 208360.02 | 769830.98 | 242387.12 | 245544.95 | 981424.89 | 651944.84 | 1117396.03 | 2529068.87 | 397168.78 | 598171.50 | -1.73 | 0.007 | 1.233 |
| pos | Com_33769 | Isoproterenol | C11 H17 N O3 | 211.12 | 5.43 | 212.13 | 641454.44 | 686186.51 | 1212956.36 | 822201.67 | 520569.42 | 459501.18 | 305178.79 | 303614.09 | 442274.43 | 345444.86 | 238719.09 | 302647.30 | 1.16 | 0.001 | 1.122 |
| pos | Com_34147 | Norbuprenorphine | C25 H35 N O4 | 413.25 | 10.25 | 414.26 | 127363.74 | 98557.05 | 94727.67 | 91968.67 | 100604.20 | 106690.70 | 2215560.50 | 1522078.92 | 1357100.23 | 2444612.99 | 1360460.99 | 1262443.71 | -4.04 | 0.000 | 1.786 |
| pos | Com_34219 | 4-(cyclohexylmethyl)-6-(2-thienyl)-2,3-dihydropyridazin-3-one hydrate | C15 H18 N2 O S | 296.09 | 5.84 | 297.10 | 177037.27 | 215212.22 | 338437.58 | 453953.18 | 318431.11 | 320055.43 | 1503667.18 | 1516159.80 | 2004028.26 | 1590389.58 | 2013090.83 | 1891073.29 | -2.53 | 0.000 | 1.450 |
| pos | Com_34973 | (3beta,9xi)-3-(beta-D-Glucopyranosyloxy)-14-hydroxycard-20(22)-enolide | C29 H44 O9 | 518.29 | 11.46 | 519.29 | 193602.03 | 229508.90 | 397635.74 | 309679.34 | 447562.55 | 252524.67 | 1477541.47 | 1353258.67 | 1655389.90 | 2077177.61 | 2027268.72 | 2430638.43 | -2.59 | 0.000 | 1.679 |
| pos | Com_35112 | RKK | C18 H38 N8 O4 | 215.15 | 0.86 | 216.16 | 518529.36 | 699230.04 | 1425458.37 | 1004600.48 | 1540131.46 | 1476996.27 | 222338.16 | 239151.47 | 514269.94 | 293705.44 | 269833.18 | 205851.22 | 1.93 | 0.000 | 1.441 |
| pos | Com_35383 | Fmoc-L-Isoleucine | C21 H23 N O4 | 353.16 | 10.23 | 354.17 | 1229654.23 | 1140771.03 | 105840.34 | 1348263.65 | 233632.40 | 492049.09 | 159430.62 | 117299.49 | 119466.58 | 128867.07 | 147687.48 | 158486.28 | 2.45 | 0.024 | 1.261 |
| pos | Com_35519 | PC (14:1e/6:0) | C28 H56 N O7 P | 567.38 | 12.40 | 568.39 | 761891.88 | 525662.49 | 537037.88 | 590405.85 | 626282.36 | 581722.33 | 1837905.29 | 1197565.70 | 1339128.58 | 2241711.77 | 562501.05 | 684921.11 | -1.12 | 0.028 | 1.208 |
| pos | Com_35672 | N-[1-(4-methoxy-2-oxo-2H-pyran-6-yl)-2-methylbutyl]acetamide | C13 H19 N O4 | 253.13 | 8.01 | 254.14 | 130543.44 | 151693.73 | 150816.23 | 181863.04 | 178388.45 | 195954.08 | 975798.94 | 450929.46 | 468961.36 | 461161.88 | 579501.98 | 537226.42 | -1.81 | 0.000 | 1.243 |
| pos | Com_35829 | 1-(4-methylphenyl)-3,5-diphenylpent-2-ene-1,5-dione | C24 H20 O2 | 322.13 | 8.53 | 323.14 | 104696.27 | 125930.56 | 162526.30 | 158606.09 | 164018.65 | 161871.90 | 1444071.46 | 698290.21 | 984353.07 | 1227579.99 | 704442.74 | 691336.92 | -2.71 | 0.000 | 1.517 |
| pos | Com_35991 | Ala-Ile | C9 H18 N2 O3 | 202.13 | 7.29 | 203.14 | 245795.40 | 378590.61 | 669827.45 | 488484.93 | 396485.43 | 530346.77 | 88555.94 | 68939.34 | 72301.57 | 78831.38 | 152376.57 | 62033.31 | 2.37 | 0.000 | 1.218 |
| pos | Com_36667 | 7-hydroxy-6-nitro-2,3-dihydro-1H,5H-pyrido[3,2,1-ij]quinolin-5-one | C12 H10 N2 O4 | 246.06 | 7.42 | 247.07 | 266693.79 | 610570.58 | 541209.40 | 683765.02 | 293313.58 | 416326.29 | 706502.53 | 691526.17 | 1444732.91 | 825891.63 | 1946591.90 | 2175914.59 | -1.47 | 0.005 | 1.184 |
| pos | Com_37356 | (1E)-5-hydroxy-1,7-diphenylhept-1-en-3-one | C19 H20 O2 | 302.13 | 8.28 | 303.13 | 269720.96 | 260373.39 | 162661.41 | 269618.35 | 287945.02 | 266265.48 | 2090992.40 | 1962200.94 | 1564640.04 | 1100645.69 | 711357.67 | 952035.94 | -2.47 | 0.000 | 1.786 |
| pos | Com_37784 | KKK | C18 H38 N6 O4 | 201.15 | 0.85 | 202.15 | 1051779.46 | 422854.37 | 460854.42 | 669326.32 | 497431.27 | 643235.91 | 164360.91 | 114747.91 | 49174.09 | 153711.47 | 96064.45 | 111835.37 | 2.44 | 0.000 | 1.507 |
| pos | Com_38067 | FRH | C21 H30 N8 O4 | 458.24 | 8.02 | 459.24 | 182617.40 | 125202.44 | 191268.45 | 1368342.74 | 286810.12 | 211468.63 | 65824.42 | 83777.99 | 133329.06 | 52002.28 | 52905.19 | 79926.95 | 2.34 | 0.012 | 1.382 |
| pos | Com_38140 | 2-{[(4,5-dimethoxy-2-nitrophenethyl)imino]methyl}phenol | C17 H18 N2 O5 | 330.12 | 10.28 | 331.13 | 73862.89 | 104679.87 | 100719.92 | 93779.35 | 107129.65 | 113610.93 | 950347.21 | 990802.85 | 1803867.93 | 393139.04 | 217852.03 | 495487.52 | -3.03 | 0.001 | 1.558 |
| pos | Com_38255 | LysoPC 12:1 | C20 H36 N O7 P | 433.22 | 8.77 | 434.23 | 900984.70 | 524264.19 | 824437.83 | 1051061.76 | 428565.17 | 369210.74 | 180257.92 | 277539.94 | 324129.41 | 110663.21 | 190479.19 | 220930.65 | 1.65 | 0.001 | 1.177 |
| pos | Com_38303 | 4-acetyl-4-(ethoxycarbonyl)heptanedioic acid | C12 H18 O7 | 296.08 | 6.61 | 297.09 | 104777.17 | 120265.95 | 130851.14 | 167794.57 | 178758.87 | 161685.12 | 354501.59 | 1845935.96 | 207012.47 | 827351.03 | 586136.47 | 1684698.90 | -2.67 | 0.005 | 1.418 |
| pos | Com_38572 | 3-(4,5-diphenyl-1,3-oxazol-2-yl)propanoic acid | C18 H15 N O3 | 293.11 | 9.03 | 294.12 | 461928.68 | 127021.33 | 187399.11 | 286197.88 | 357777.01 | 149049.27 | 1533042.18 | 938920.09 | 810943.88 | 1130541.50 | 902032.33 | 563047.91 | -1.91 | 0.000 | 1.528 |
| pos | Com_39019 | (2E)-1-(2-hydroxy-3,4,5,6-tetramethoxyphenyl)-3-phenylprop-2-en-1-one | C19 H20 O6 | 688.24 | 9.21 | 345.13 | 71081.24 | 195491.36 | 90087.78 | 149908.78 | 463563.81 | 224250.69 | 745540.81 | 1502806.12 | 238268.79 | 974125.26 | 475291.21 | 750184.72 | -1.97 | 0.004 | 1.194 |
| pos | Com_39089 | 3-(4-nitrophenyl)[1,2,3]triazolo[1,5-a]quinazolin-5-amine | C15 H10 N6 O2 | 612.17 | 2.68 | 307.09 | 214147.89 | 262804.91 | 277619.67 | 318816.89 | 274030.57 | 298540.70 | 1518654.42 | 830329.55 | 680413.39 | 1767367.70 | 376009.60 | 1709521.19 | -2.06 | 0.003 | 1.522 |
| pos | Com_39162 | 13,14-dihydro-15-keto-tetranor Prostaglandin D2 | C16 H26 O5 | 280.16 | 8.72 | 281.17 | 318971.39 | 229049.81 | 434358.79 | 263730.00 | 303531.65 | 124837.84 | 1068342.81 | 654234.50 | 293196.09 | 893712.10 | 1290269.80 | 877302.69 | -1.60 | 0.003 | 1.078 |
| pos | Com_39423 | Spectinomycin | C14 H24 N2 O7 | 364.19 | 10.51 | 365.19 | 349440.76 | 596938.65 | 889865.76 | 908603.93 | 632744.55 | 486506.30 | 1585929.95 | 1486232.92 | 1651828.32 | 1494447.71 | 1857278.82 | 1343888.80 | -1.29 | 0.001 | 1.386 |
| pos | Com_39857 | Lysopc 14:0 | C22 H46 N O7 P | 467.30 | 10.83 | 468.31 | 114536.29 | 240953.79 | 438863.04 | 570579.13 | 406336.75 | 358274.56 | 1165317.51 | 1252083.03 | 1260376.62 | 1269990.77 | 1518612.47 | 1036033.68 | -1.82 | 0.002 | 1.122 |
| pos | Com_40333 | 2-[3-(trifluoromethyl)-1H-pyrazol-1-yl]-4H-chromen-4-one | C13 H7 F3 N2 O2 | 280.05 | 9.79 | 281.06 | 107463.12 | 119914.20 | 115136.83 | 139540.86 | 126271.26 | 132400.90 | 1034976.17 | 930504.26 | 1550616.94 | 769214.84 | 364759.58 | 417744.99 | -2.77 | 0.000 | 1.656 |
| pos | Com_41062 | N-Acetyl-L-histidine | C8 H11 N3 O3 | 197.08 | 1.08 | 198.09 | 359280.24 | 1224822.16 | 691377.36 | 962081.11 | 861982.56 | 1042215.18 | 258751.47 | 302527.59 | 225131.75 | 228075.01 | 338406.58 | 299464.35 | 1.64 | 0.001 | 1.492 |
| pos | Com_41544 | FNK | C19 H29 N5 O5 | 407.22 | 7.90 | 408.23 | 317812.85 | 209250.76 | 1199820.49 | 601922.90 | 337948.27 | 321973.79 | 91839.88 | 78041.85 | 105119.40 | 86668.17 | 103581.16 | 84747.99 | 2.44 | 0.002 | 1.463 |
| pos | Com_41560 | LPH | C17 H27 N5 O4 | 365.21 | 7.49 | 366.21 | 369839.04 | 264550.69 | 252058.85 | 545300.66 | 268204.24 | 243806.52 | 71074.40 | 66450.29 | 70767.07 | 65863.29 | 98914.52 | 68304.63 | 2.14 | 0.000 | 1.320 |
| pos | Com_42014 | 16H-dinaphtho[2,1-d:1,2-g][1,3]dioxocine | C22 H16 O2 | 624.24 | 8.39 | 313.13 | 126816.92 | 369625.43 | 324033.06 | 467044.52 | 511363.75 | 493933.33 | 733435.50 | 782211.53 | 705037.96 | 728377.84 | 1091962.08 | 1424982.87 | -1.25 | 0.006 | 1.139 |
| pos | Com_42420 | 4-(2-methoxyphenyl)-1-(3-phenyl-1,2,4-thiadiazol-5-yl)piperidine | C20 H21 N3 O S | 351.14 | 10.29 | 352.15 | 66639.21 | 138223.44 | 219548.07 | 139119.23 | 118712.07 | 204615.84 | 624775.75 | 799456.54 | 982292.26 | 1146766.87 | 617632.54 | 512583.95 | -2.40 | 0.000 | 1.478 |
| pos | Com_43185 | IPH | C17 H27 N5 O4 | 365.20 | 5.40 | 366.21 | 455670.18 | 402575.40 | 315174.52 | 743927.71 | 675840.93 | 548154.69 | 234529.78 | 113333.96 | 308559.07 | 208400.60 | 85411.59 | 137630.32 | 1.53 | 0.001 | 1.499 |
| pos | Com_43235 | CKK | C15 H31 N5 O4 S | 798.38 | 6.48 | 400.20 | 549338.66 | 363094.68 | 457586.95 | 806272.87 | 910463.48 | 632745.03 | 135839.70 | 130191.06 | 306300.87 | 116726.55 | 441398.44 | 189137.99 | 1.50 | 0.002 | 1.331 |
| pos | Com_44321 | 2-Thio-acetyl MAGE | C21 H42 O3 S | 396.27 | 9.53 | 397.28 | 107141.53 | 115811.54 | 112915.54 | 134514.45 | 135470.35 | 136686.24 | 714243.47 | 1099985.05 | 764840.33 | 927654.32 | 1295627.43 | 1214118.56 | -3.02 | 0.000 | 1.818 |
| pos | Com_44718 | HNK | C16 H27 N7 O5 | 397.21 | 0.92 | 199.61 | 416209.46 | 526471.97 | 541118.79 | 828378.10 | 528926.73 | 838498.07 | 231404.65 | 275997.18 | 341934.78 | 255299.74 | 296982.43 | 374941.72 | 1.05 | 0.001 | 1.544 |
| pos | Com_46075 | LPC 10:0 | C18 H38 N O7 P | 411.24 | 8.93 | 412.24 | 101431.79 | 114963.80 | 161851.55 | 138587.60 | 130378.26 | 132858.51 | 790516.47 | 798593.00 | 709231.84 | 870168.14 | 341975.81 | 956926.40 | -2.52 | 0.000 | 1.601 |
| pos | Com_46227 | N'3-(2-thienylmethylene)-5-(1-hexynyl)-3-pyridinecarbohydrazide | C17 H17 N3 O S | 311.11 | 2.91 | 312.12 | 45632.86 | 213532.35 | 63078.18 | 138521.75 | 70449.14 | 194753.76 | 809584.87 | 961583.64 | 928448.06 | 484438.93 | 372432.12 | 396865.26 | -2.45 | 0.000 | 1.413 |
| pos | Com_47560 | Lysops 22:6 | C28 H44 N O9 P | 569.28 | 9.94 | 570.29 | 176879.71 | 133235.93 | 163455.03 | 168861.55 | 127577.29 | 161564.38 | 555999.31 | 586452.31 | 340557.78 | 455517.13 | 847139.55 | 852127.38 | -1.97 | 0.000 | 1.775 |
| neg | Com_47 | D-(-)-Mannitol | C6 H14 O6 | 182.08 | 1.29 | 181.07 | 377639318.31 | 3394545020.41 | 2675020265.00 | 3133000532.08 | 4166834726.75 | 4451148070.10 | 117850066.86 | 108144195.89 | 103711943.03 | 107587745.03 | 209056859.34 | 119128302.53 | 4.57 | 0.000 | 1.698 |
| neg | Com_154 | Gluconic acid | C6 H12 O7 | 196.06 | 1.22 | 195.05 | 145899277.22 | 741997465.81 | 292084749.43 | 455083430.37 | 548037573.86 | 470836318.73 | 132479958.08 | 185063172.53 | 183931091.20 | 100066791.08 | 177252922.65 | 141158133.04 | 1.53 | 0.007 | 1.534 |
| neg | Com_207 | Adipamide | C6 H12 N2 O2 | 144.09 | 1.34 | 143.08 | 66045643.93 | 55210537.88 | 416395384.45 | 537437897.36 | 332825952.02 | 252108962.26 | 62756061.50 | 68344918.15 | 79170748.24 | 68926542.36 | 82087294.00 | 66992600.46 | 1.95 | 0.046 | 1.213 |
| neg | Com_221 | L-Glutamic acid | C5 H9 N O4 | 147.05 | 1.28 | 128.04 | 107523966.12 | 217540567.20 | 511391328.38 | 334588725.99 | 236684613.27 | 355435526.43 | 735120560.33 | 520552117.79 | 586483147.59 | 722018496.13 | 624598196.41 | 651575316.10 | -1.12 | 0.009 | 1.033 |
| neg | Com_309 | L-Serine | C3 H7 N O3 | 105.04 | 1.25 | 104.04 | 55752473.62 | 127903475.81 | 91670249.01 | 121913510.20 | 138414189.43 | 144982753.26 | 233952665.93 | 232388007.81 | 216117271.12 | 293759812.95 | 349189881.48 | 301256121.05 | -1.26 | 0.001 | 1.303 |
| neg | Com_332 | Corchorifatty acid F | C18 H32 O5 | 328.23 | 11.00 | 327.22 | 4864444.27 | 12151242.06 | 18327871.59 | 14612896.51 | 12293099.93 | 7522757.69 | 80471094.46 | 106694924.59 | 29838679.13 | 75218290.14 | 238460610.25 | 76509830.60 | -3.12 | 0.000 | 1.487 |
| neg | Com_387 | cis,cis-Muconic acid | C6 H6 O4 | 142.03 | 2.14 | 141.02 | 18671943.71 | 94855775.00 | 34311447.91 | 71233661.17 | 35955535.87 | 65689673.32 | 169984376.80 | 248697517.42 | 467649435.01 | 78033909.21 | 139504335.56 | 183778600.90 | -2.01 | 0.003 | 1.168 |
| neg | Com_434 | Stearic acid | C18 H36 O2 | 284.27 | 15.49 | 283.26 | 25785686.63 | 87335898.29 | 85487450.89 | 76414660.93 | 99674197.62 | 109092484.98 | 226741720.04 | 217751340.74 | 133038989.05 | 309282015.70 | 224180222.33 | 204646883.60 | -1.44 | 0.003 | 1.434 |
| neg | Com_444 | Glycerol 3-phosphate | C3 H9 O6 P | 172.01 | 1.18 | 171.01 | 114717000.77 | 172045583.17 | 191308370.71 | 214521496.18 | 136697201.63 | 145431365.72 | 40909599.34 | 40162455.69 | 33834539.94 | 32823845.76 | 83745669.34 | 75333130.11 | 1.67 | 0.000 | 1.939 |
| neg | Com_625 | N7-Methylguanosine | C11 H17 N5 O5 | 299.12 | 1.28 | 298.11 | 25105382.27 | 87874672.63 | 139107584.61 | 49118279.96 | 66859066.80 | 61034809.90 | 146426062.86 | 130072106.52 | 108110056.87 | 150341328.40 | 160782527.87 | 168115707.89 | -1.01 | 0.016 | 1.008 |
| neg | Com_664 | trans-10-Heptadecenoic acid | C17 H32 O2 | 268.24 | 14.90 | 267.23 | 3352140.53 | 39483686.94 | 24053731.39 | 14503215.85 | 28440242.28 | 38197837.52 | 120091458.93 | 111264126.19 | 38192653.95 | 142798296.27 | 70083604.29 | 73860651.25 | -1.91 | 0.009 | 1.376 |
| neg | Com_674 | Mevalonic acid | C6 H12 O4 | 148.07 | 1.37 | 147.07 | 48895951.66 | 104101592.71 | 65242707.35 | 79317465.13 | 104128941.86 | 112601770.40 | 243380899.26 | 152378130.92 | 214114265.70 | 151823503.16 | 162861558.46 | 203044720.79 | -1.13 | 0.001 | 1.125 |
| neg | Com_707 | Tanespimycin | C31 H43 N3 O8 | 621.28 | 9.97 | 620.27 | 64240493.34 | 27994449.11 | 12162564.17 | 31444828.64 | 56364625.30 | 34273500.31 | 6710579.71 | 11915143.27 | 15241630.38 | 10885316.86 | 13835607.92 | 6972639.19 | 1.79 | 0.003 | 1.390 |
| neg | Com_713 | Succinic acid | C4 H6 O4 | 118.03 | 1.34 | 117.02 | 33554171.54 | 120345460.71 | 150584893.84 | 87158682.99 | 111770702.30 | 115240518.77 | 28169605.86 | 29776264.32 | 38139339.00 | 28586292.89 | 55024033.33 | 31614589.52 | 1.55 | 0.004 | 1.472 |
| neg | Com_721 | Jasmonic acid | C12 H18 O3 | 210.13 | 12.64 | 209.12 | 47626951.24 | 90260928.74 | 72749586.77 | 110760912.23 | 42050487.39 | 44409476.52 | 14499033.95 | 16302937.93 | 44268160.72 | 11260362.78 | 9472102.28 | 13299106.72 | 1.90 | 0.001 | 1.159 |
| neg | Com_736 | Prolylglycine | C7 H12 N2 O3 | 172.09 | 1.35 | 171.08 | 31039902.96 | 86236775.31 | 103606931.80 | 133007472.26 | 95692722.22 | 81721627.56 | 35143504.03 | 33658537.33 | 34554887.51 | 29290829.82 | 43489617.51 | 38746858.18 | 1.31 | 0.009 | 1.295 |
| neg | Com_742 | Gluconolactone | C6 H10 O6 | 178.05 | 1.31 | 177.04 | 40078224.05 | 114093437.60 | 19573793.18 | 19081692.91 | 30673081.04 | 120647750.35 | 136482280.73 | 163346316.26 | 208957378.55 | 129678396.83 | 115416604.18 | 156788138.21 | -1.40 | 0.013 | 1.639 |
| neg | Com_743 | α-Lactose | C12 H22 O11 | 388.12 | 1.29 | 387.11 | 3322384.01 | 10433867.85 | 15307735.39 | 9742845.01 | 10640791.22 | 10671591.05 | 36572384.83 | 37583308.85 | 27301543.14 | 33659766.39 | 32403314.49 | 66171330.34 | -1.96 | 0.000 | 1.353 |
| neg | Com_774 | Oxoadipic Acid | C6 H8 O5 | 160.04 | 1.36 | 159.03 | 19044418.77 | 75931244.35 | 45395247.38 | 53647695.09 | 56556410.98 | 69262304.97 | 92809214.84 | 106742559.98 | 197387741.06 | 110681926.04 | 102051169.89 | 131039529.40 | -1.21 | 0.005 | 1.439 |
| neg | Com_913 | Phenylacetaldehyde | C8 H8 O | 120.06 | 6.26 | 119.05 | 2294556.28 | 30080218.58 | 107169020.35 | 58989335.24 | 14313325.21 | 20205041.53 | 1637522.01 | 2076436.67 | 926616.12 | 1628755.40 | 1138893.11 | 1157347.81 | 4.77 | 0.003 | 1.812 |
| neg | Com_927 | 3-Phenyllactic acid | C9 H10 O3 | 166.06 | 6.29 | 165.06 | 2391892.64 | 29869424.99 | 103916061.84 | 56845731.42 | 14526865.46 | 19662781.63 | 1943855.56 | 2399503.14 | 1390655.66 | 2141899.68 | 1640057.52 | 1712248.36 | 4.34 | 0.005 | 1.776 |
| neg | Com_992 | 2,2-Bis(hydroxymethyl)propionic acid | C5 H10 O4 | 134.06 | 1.53 | 133.05 | 40303184.86 | 36132429.66 | 62071994.25 | 52504109.42 | 58198401.54 | 59041722.17 | 14879642.92 | 14439608.28 | 13345868.24 | 15452188.16 | 20912427.05 | 13510063.97 | 1.74 | 0.000 | 1.618 |
| neg | Com_1236 | N-Acetylvaline | C7 H13 N O3 | 159.09 | 1.37 | 158.08 | 21198878.25 | 51300023.39 | 25708520.92 | 53659615.78 | 45969929.18 | 57716917.58 | 99920208.57 | 82696371.65 | 108885934.08 | 99803381.36 | 99415398.62 | 96750223.14 | -1.20 | 0.003 | 1.364 |
| neg | Com_1274 | (+/-)5(6)-DiHET | C20 H34 O4 | 338.25 | 12.98 | 337.24 | 2784316.46 | 11853216.00 | 8059824.14 | 9382464.79 | 11189522.33 | 20617318.18 | 44153598.45 | 42551079.45 | 34510737.05 | 47580790.82 | 34326570.71 | 44562485.41 | -1.95 | 0.002 | 1.396 |
| neg | Com_1308 | Homoarginine | C7 H16 N4 O2 | 188.13 | 1.83 | 187.12 | 6934882.25 | 27046929.13 | 27362684.43 | 29208659.93 | 29028662.83 | 25454835.50 | 72215417.64 | 84920375.87 | 103194849.16 | 94045609.05 | 90611977.23 | 87579025.30 | -1.88 | 0.001 | 1.550 |
| neg | Com_1381 | 5-Hydroxytryptophan | C11 H12 N2 O3 | 220.09 | 5.70 | 219.08 | 6763798.06 | 17429737.48 | 11550043.98 | 17854770.97 | 13802252.76 | 15060038.73 | 46459767.04 | 48535644.48 | 96497311.72 | 38959431.40 | 24614547.81 | 43911420.55 | -1.86 | 0.000 | 1.447 |
| neg | Com_1564 | UDP-N-acetylglucosamine | C17 H27 N3 O17 P2 | 607.08 | 1.23 | 606.08 | 5990539.74 | 7993464.40 | 8081698.20 | 5781042.95 | 22730203.70 | 10973259.06 | 31525858.20 | 38846021.03 | 38830365.60 | 57249377.29 | 39359182.71 | 43367157.99 | -2.02 | 0.000 | 1.427 |
| neg | Com_1597 | Traumatic acid | C12 H20 O4 | 228.14 | 9.46 | 227.13 | 6583614.44 | 14046125.46 | 10365459.58 | 25293777.57 | 11501675.83 | 8828166.17 | 46480547.95 | 45032838.19 | 34443084.25 | 37259790.76 | 27881468.74 | 33698303.92 | -1.55 | 0.001 | 1.570 |
| neg | Com_1604 | 11-Deoxy prostaglandin F2β | C20 H34 O4 | 338.25 | 13.18 | 337.24 | 3389570.69 | 10204987.89 | 8906733.05 | 7443785.16 | 6354573.64 | 8780574.07 | 33444219.58 | 29972735.87 | 21564271.18 | 30563535.29 | 25569940.79 | 29231587.81 | -1.92 | 0.000 | 1.523 |
| neg | Com_1710 | Lipoic acid | C8 H14 O2 S2 | 206.04 | 1.09 | 205.04 | 10235197.97 | 42058733.51 | 21320086.27 | 45368859.80 | 22170489.83 | 41287493.33 | 8790861.46 | 9246932.08 | 8403634.33 | 7717670.92 | 20881464.94 | 7304387.84 | 1.55 | 0.006 | 1.218 |
| neg | Com_1814 | Adipic acid | C6 H10 O4 | 146.06 | 1.85 | 145.05 | 1610417.15 | 3862851.41 | 3233671.83 | 4257586.75 | 5008500.96 | 3452115.08 | 15501712.76 | 8350511.51 | 8604504.79 | 10334879.68 | 12454655.33 | 8130658.68 | -1.56 | 0.000 | 1.166 |
| neg | Com_1839 | Maltotetraose | C24 H42 O21 | 666.22 | 1.37 | 665.22 | 6222603.67 | 24749409.28 | 44407286.21 | 32508822.98 | 22300211.70 | 28768073.93 | 4467459.94 | 6363142.02 | 7396355.54 | 4418966.61 | 9944147.84 | 11672480.92 | 1.84 | 0.006 | 1.628 |
| neg | Com_1879 | Dl-Threitol | C4 H10 O4 | 122.06 | 1.34 | 121.05 | 6455702.82 | 11111045.00 | 42894438.17 | 28291562.36 | 10139420.72 | 25933705.79 | 6497957.88 | 5482562.08 | 5482975.59 | 5117985.73 | 9236655.75 | 6625821.73 | 1.70 | 0.020 | 1.144 |
| neg | Com_1885 | Uridine 5'-diphosphogalactose | C15 H24 N2 O17 P2 | 566.06 | 1.20 | 565.05 | 1528511.38 | 7133759.65 | 2036726.09 | 2858628.75 | 19306068.92 | 21336225.39 | 25666101.86 | 35291799.38 | 27625298.74 | 63338292.94 | 21059426.80 | 29013356.98 | -1.90 | 0.012 | 1.194 |
| neg | Com_1980 | Lactobionic acid | C12 H22 O12 | 358.11 | 1.28 | 393.08 | 2769888.28 | 3795977.43 | 4191165.12 | 7432717.44 | 4439901.98 | 8361452.49 | 25604484.83 | 26906807.25 | 24599967.19 | 59122795.55 | 13790778.89 | 31203953.72 | -2.55 | 0.000 | 1.869 |
| neg | Com_1988 | 3-Methylglutaric acid | C6 H10 O4 | 146.06 | 1.38 | 145.05 | 1030917.97 | 7855631.17 | 2680044.42 | 3521767.57 | 5831556.12 | 5777672.04 | 59704411.54 | 13580875.61 | 3984462.20 | 23204694.86 | 6582988.65 | 8539892.64 | -2.11 | 0.034 | 1.046 |
| neg | Com_2003 | 10-Hydroxydecanoic acid | C10 H20 O3 | 188.14 | 9.87 | 187.13 | 912145.04 | 5073438.80 | 3474753.04 | 3778161.67 | 3794839.14 | 2471115.00 | 29068998.94 | 18488138.38 | 8673708.48 | 17766254.79 | 12313721.06 | 15235626.95 | -2.38 | 0.000 | 1.638 |
| neg | Com_2144 | ethyl 3-[(4-chlorophenethyl)amino]-2-cyanoacrylate | C14 H15 Cl N2 O2 | 278.08 | 8.23 | 277.08 | 1103285.27 | 1832795.80 | 2625413.34 | 2636150.67 | 2377654.22 | 1562566.52 | 54109923.39 | 28227402.96 | 35584858.49 | 51411567.29 | 15400444.05 | 27624370.65 | -4.13 | 0.000 | 1.976 |
| neg | Com_2217 | 4-((5-(4-Nitrophenyl)oxazol-2-yl)amino)benzonitrile | C16 H10 N4 O3 | 612.14 | 1.23 | 305.06 | 14552767.73 | 28721382.49 | 23935953.68 | 30520730.09 | 13077593.89 | 24914501.24 | 4836031.83 | 7119644.89 | 1569733.42 | 6104831.38 | 2819718.88 | 11732933.50 | 1.99 | 0.002 | 1.310 |
| neg | Com_2227 | LPC 18:2 | C26 H50 N O7 P | 565.34 | 14.75 | 564.33 | 52634.49 | 196093.04 | 158925.15 | 159655.30 | 151073.95 | 159033.10 | 983464.43 | 34855411.74 | 3437455.12 | 48739997.31 | 7590011.30 | 2484605.67 | -6.80 | 0.001 | 1.471 |
| neg | Com_2241 | L-Pipecolate | C6 H11 N O2 | 129.08 | 1.37 | 128.07 | 14341513.22 | 12787195.05 | 7476702.70 | 12475530.52 | 12802624.86 | 17925504.11 | 50464974.71 | 31011107.44 | 30572316.59 | 36506356.24 | 30201222.10 | 28049642.72 | -1.41 | 0.000 | 1.633 |
| neg | Com_2355 | 2,3-Dinor-8-epi-prostaglandin F2α | C18 H30 O5 | 326.21 | 9.70 | 325.20 | 2472242.77 | 8603429.74 | 11236168.03 | 11824278.86 | 7398497.86 | 6925052.16 | 17607528.83 | 16452405.72 | 26615577.56 | 17405325.62 | 28416208.65 | 27154530.22 | -1.46 | 0.004 | 1.504 |
| neg | Com_2411 | 8-iso Prostaglandin F2α Ethanolamide | C22 H39 N O5 | 397.28 | 12.10 | 442.28 | 7049638.49 | 9658344.42 | 10142261.75 | 10773142.00 | 26076414.71 | 17193944.85 | 2747694.07 | 3642353.63 | 4717894.93 | 1997375.92 | 4023455.92 | 3275077.34 | 1.99 | 0.000 | 1.385 |
| neg | Com_2542 | Azelaic acid | C9 H16 O4 | 188.11 | 3.73 | 187.10 | 842284.23 | 2566464.99 | 2301669.09 | 2128376.88 | 2962610.94 | 2989893.33 | 36993302.51 | 5305635.47 | 3054482.05 | 5248942.12 | 6119389.14 | 51945200.56 | -2.98 | 0.022 | 1.387 |
| neg | Com_2544 | Ergothioneine | C9 H15 N3 O2 S | 229.09 | 1.37 | 228.08 | 1284837.85 | 5022269.63 | 3300283.45 | 3925413.42 | 4320111.77 | 4953827.34 | 11791126.20 | 10848593.09 | 9003895.34 | 9233286.87 | 14169865.02 | 16124960.19 | -1.64 | 0.001 | 1.115 |
| neg | Com_2650 | 20-Hydroxy-(5Z,8Z,11Z,14Z)-eicosatetraenoic acid | C20 H32 O3 | 320.24 | 14.18 | 319.23 | 7988342.82 | 30151307.05 | 12311154.13 | 23023105.40 | 11925430.41 | 13935791.01 | 8144815.02 | 5726558.77 | 12732396.11 | 5795634.79 | 7383933.37 | 7919749.59 | 1.06 | 0.019 | 1.065 |
| neg | Com_3208 | (+/-)19(20)-DiHDPA | C22 H34 O4 | 362.24 | 14.82 | 361.24 | 300076.81 | 4046357.89 | 2773419.55 | 1748585.18 | 5617079.21 | 7763071.57 | 27207626.59 | 20377636.65 | 7854909.72 | 26548141.53 | 15526045.59 | 13329360.23 | -2.32 | 0.008 | 1.347 |
| neg | Com_3210 | N4-Acetylcytidine | C11 H15 N3 O6 | 285.10 | 1.37 | 284.09 | 578028.23 | 2511604.56 | 1424387.44 | 2029946.75 | 2075777.81 | 2580040.92 | 27524968.03 | 24674764.38 | 30384545.35 | 5665849.77 | 4552822.35 | 8207224.63 | -3.17 | 0.001 | 1.541 |
| neg | Com_3300 | O-Acetyl-L-carnitine | C9 H17 N O4 | 203.12 | 3.75 | 202.11 | 548491.09 | 926079.99 | 879656.48 | 829229.51 | 1678046.59 | 984601.02 | 14623194.02 | 9711715.06 | 14033666.43 | 12064681.55 | 18588422.81 | 20177234.48 | -3.93 | 0.000 | 1.775 |
| neg | Com_3441 | LPS 17:1 | C23 H44 N O9 P | 509.28 | 14.46 | 508.27 | 77543.33 | 338818.89 | 88413.95 | 127881.60 | 1149564.05 | 360376.27 | 3530619.73 | 8597410.33 | 6080256.77 | 10295762.48 | 5378281.59 | 7081655.22 | -4.26 | 0.000 | 1.644 |
| neg | Com_3442 | Nicotinamide adenine dinucleotide | C21 H27 N7 O14 P2 | 663.11 | 2.05 | 662.10 | 511841.89 | 1049696.51 | 2541018.47 | 672399.85 | 2321154.26 | 1552340.98 | 2944672.88 | 6075019.38 | 5600200.18 | 10459052.59 | 11106440.87 | 4633942.00 | -2.24 | 0.001 | 1.211 |
| neg | Com_3504 | 2-Arachidonyl Glycerol ether | C23 H40 O3 | 400.27 | 9.82 | 399.26 | 4830781.38 | 5459181.77 | 9458749.67 | 10480257.16 | 7251384.86 | 4280071.98 | 845251.03 | 711085.80 | 2548669.33 | 1215136.31 | 1528794.60 | 1052900.51 | 2.40 | 0.000 | 1.363 |
| neg | Com_3520 | dAMP | C10 H14 N5 O6 P | 331.07 | 2.44 | 330.06 | 3410085.36 | 6337181.62 | 4891229.29 | 8508336.86 | 7415980.69 | 7179298.23 | 2816669.78 | 2711357.21 | 3107530.08 | 2585260.16 | 1930290.69 | 2252411.45 | 1.29 | 0.001 | 1.127 |
| neg | Com_3594 | Phosphoenolpyruvic acid | C3 H5 O6 P | 167.98 | 1.10 | 166.98 | 883735.92 | 4998855.66 | 2115857.78 | 3725045.53 | 5431987.83 | 7429025.73 | 16458816.25 | 22699662.50 | 7965713.31 | 18940595.31 | 9192251.24 | 9819545.57 | -1.79 | 0.006 | 1.013 |
| neg | Com_3603 | Taurocholic acid sodium salt hydrate | C26 H45 N Na O7 S | 538.28 | 9.58 | 537.28 | 91633.63 | 4975317.56 | 1117780.42 | 3255565.02 | 11748148.05 | 3742517.22 | 17336554.48 | 19744982.40 | 6673359.55 | 18832153.32 | 23014520.28 | 30750717.45 | -2.22 | 0.025 | 1.174 |
| neg | Com_3633 | Indole-3-butyric acid | C12 H13 N O2 | 203.09 | 1.26 | 202.08 | 5707454.28 | 10144509.65 | 10033654.34 | 11766936.88 | 14438086.83 | 13171116.86 | 3649416.00 | 4375025.37 | 6246595.47 | 4444957.98 | 5926197.44 | 5516307.40 | 1.11 | 0.001 | 1.579 |
| neg | Com_3678 | Eicosapentaenoic acid | C20 H30 O2 | 302.22 | 14.23 | 301.22 | 5210526.71 | 18903186.26 | 6259887.19 | 8939857.67 | 9541222.92 | 7583642.31 | 4717554.08 | 2257365.85 | 6945181.11 | 2602974.34 | 2487561.39 | 3198954.54 | 1.35 | 0.005 | 1.092 |
| neg | Com_3799 | (-)-Erythromycin | C37 H67 N O13 | 733.45 | 14.01 | 732.45 | 1630383.85 | 633895.61 | 549346.70 | 832645.34 | 1349990.25 | 416385.66 | 2819866.63 | 2113988.98 | 1252062.59 | 4028911.28 | 8038132.35 | 1585881.61 | -1.87 | 0.007 | 1.088 |
| neg | Com_4006 | Curcumin | C21 H20 O6 | 368.12 | 9.11 | 367.12 | 1323540.10 | 8073009.97 | 2532728.00 | 7492112.65 | 1353624.65 | 2682763.80 | 404621.16 | 350453.24 | 779137.65 | 216739.88 | 266576.79 | 302234.50 | 3.34 | 0.000 | 1.448 |
| neg | Com_4074 | 2-[(3S)-1-(Cyclohexylmethyl)-3-pyrrolidinyl]-5-fluoro-1H-benzimidazole | C18 H24 F N3 | 301.20 | 7.23 | 300.19 | 3593081.61 | 2854846.86 | 4596126.98 | 5023517.95 | 5198454.84 | 3509905.82 | 372799.78 | 440238.15 | 2048451.63 | 562189.75 | 1097190.77 | 1039293.30 | 2.16 | 0.001 | 1.214 |
| neg | Com_4255 | Thymidine 5'-monophosphate | C10 H15 N2 O8 P | 322.06 | 1.82 | 321.05 | 2329251.44 | 4227291.45 | 3177052.38 | 5431516.66 | 3758290.53 | 4553608.92 | 1887297.37 | 1534840.07 | 1639393.33 | 1113862.41 | 1071497.50 | 1340702.94 | 1.45 | 0.000 | 1.245 |
| neg | Com_4268 | LPA 8:0 | C11 H23 O7 P | 298.12 | 11.23 | 297.11 | 371862.80 | 3741739.44 | 1214395.60 | 2399747.43 | 569465.14 | 1039140.16 | 235177.60 | 219754.14 | 332541.14 | 116139.23 | 278525.27 | 213099.60 | 2.74 | 0.004 | 1.188 |
| neg | Com_4365 | PC (17:1/17:1) | C42 H80 N O8 P | 803.58 | 16.26 | 802.57 | 583259.24 | 6814092.76 | 3697116.68 | 3589708.30 | 2307431.32 | 3206787.22 | 9020930.18 | 9052190.70 | 7328957.99 | 10319384.60 | 17297399.93 | 17588026.86 | -1.81 | 0.007 | 1.182 |
| neg | Com_4368 | Phenylpyruvic acid | C9 H8 O3 | 164.05 | 1.97 | 163.04 | 840145.38 | 1657156.18 | 1501613.45 | 1559235.31 | 6397139.50 | 3186333.71 | 10199142.76 | 9548575.54 | 2428269.29 | 11301665.68 | 5901635.15 | 9454927.18 | -1.69 | 0.007 | 1.177 |
| neg | Com_4416 | N1-cyclohexyl-4-(4-fluorobenzyl)-1,4-diazepane-1-carbothioamide | C19 H28 F N3 S | 349.20 | 9.78 | 348.19 | 2215166.52 | 3473457.80 | 5733032.95 | 5239501.12 | 4180414.68 | 3436309.20 | 695840.84 | 687895.58 | 2553628.37 | 797398.72 | 1604260.07 | 1093135.06 | 1.71 | 0.001 | 1.059 |
| neg | Com_4669 | Adenosine 5'-Diphosphate | C10 H15 N5 O10 P2 | 427.03 | 1.35 | 426.02 | 1288370.17 | 4340363.75 | 3523529.62 | 3530875.77 | 5184467.73 | 4919112.43 | 8795287.21 | 11825513.88 | 2911433.61 | 17059091.20 | 9664342.13 | 9037444.47 | -1.38 | 0.017 | 1.137 |
| neg | Com_4838 | 1-(4-fluorophenyl)-2-(4-methoxyphenyl)-4-(2-naphthyl)butane-1,4-dione | C27 H21 F O3 | 412.15 | 4.90 | 411.14 | 4917677.55 | 4236681.02 | 3734193.79 | 7174707.25 | 6555348.11 | 6950208.30 | 2230408.58 | 2125678.12 | 3132078.56 | 1239777.46 | 2787035.71 | 2369434.07 | 1.27 | 0.000 | 1.214 |
| neg | Com_5045 | LPC 17:1 | C25 H50 N O7 P | 567.35 | 14.74 | 566.35 | 47833.11 | 210181.89 | 140725.74 | 232404.90 | 128318.24 | 141739.09 | 189421.98 | 9701084.39 | 604271.45 | 9806680.78 | 470541.39 | 402076.39 | -4.55 | 0.028 | 1.130 |
| neg | Com_5673 | Cyclic ADP-ribose | C15 H21 N5 O13 P2 | 541.06 | 2.05 | 540.05 | 290358.89 | 552099.72 | 1114330.96 | 303916.00 | 1238531.28 | 681237.65 | 1374442.74 | 2730380.93 | 2017724.23 | 4809537.91 | 4513786.74 | 2111811.80 | -2.07 | 0.001 | 1.127 |
| neg | Com_5748 | LPS 17:2 | C23 H42 N O9 P | 507.26 | 14.12 | 506.25 | 45257.75 | 702978.22 | 296168.63 | 91595.25 | 1531187.35 | 970993.53 | 4370642.04 | 5119302.98 | 10025667.47 | 7483127.45 | 1395288.66 | 1095775.66 | -3.02 | 0.007 | 1.387 |
| neg | Com_5820 | 3-benzyl-1-butyl-4-hydroxy-1,2-dihydroquinolin-2-one | C20 H21 N O2 | 307.15 | 6.89 | 306.15 | 1617672.73 | 2404516.41 | 2646077.68 | 3475584.95 | 3259600.32 | 2379227.59 | 279311.29 | 405257.34 | 1144279.13 | 445755.54 | 1020922.41 | 789680.17 | 1.95 | 0.001 | 1.053 |
| neg | Com_5882 | LPC 18:1 | C26 H52 N O7 P | 567.35 | 14.93 | 566.35 | 41440.83 | 150081.32 | 156383.73 | 105491.07 | 102702.80 | 178353.61 | 205190.40 | 5712420.43 | 352441.85 | 4881460.40 | 870434.42 | 573028.55 | -4.10 | 0.009 | 1.080 |
| neg | Com_5905 | FAD | C27 H33 N9 O15 P2 | 785.16 | 7.14 | 784.15 | 1313255.16 | 3785555.61 | 2346824.01 | 2607152.05 | 4338709.07 | 4155419.30 | 8451139.03 | 8843858.53 | 4254992.15 | 12884281.15 | 8544895.66 | 9602319.83 | -1.50 | 0.001 | 1.474 |
| neg | Com_5986 | Anandamide (AEA) | C22 H37 N O2 | 407.30 | 14.17 | 406.30 | 1671283.03 | 4644108.08 | 3665404.29 | 5680127.73 | 3737854.25 | 3709281.01 | 112935.79 | 121408.29 | 1108665.72 | 101132.38 | 771634.37 | 543753.80 | 3.07 | 0.001 | 1.407 |
| neg | Com_6010 | 2-Deoxyuridine | C9 H12 N2 O5 | 228.07 | 1.37 | 227.07 | 869383.46 | 2333959.22 | 1785551.31 | 2604248.75 | 3058179.82 | 2899236.03 | 5741774.31 | 4018002.68 | 5866657.34 | 5638509.66 | 5333583.50 | 5654363.97 | -1.25 | 0.004 | 1.151 |
| neg | Com_6047 | 3-[4-methyl-1-(2-methylpropanoyl)-3-oxocyclohexyl]butanoic acid | C15 H24 O4 | 304.15 | 8.48 | 303.14 | 2271117.03 | 5272136.04 | 4131628.43 | 5196755.48 | 7792011.52 | 5569578.55 | 2180729.56 | 2445656.61 | 2742067.43 | 2331097.44 | 1802365.02 | 3077232.89 | 1.05 | 0.008 | 1.345 |
| neg | Com_6110 | Arachidic acid | C20 H40 O2 | 312.30 | 16.01 | 311.30 | 989035.20 | 2980940.99 | 3028982.76 | 2532295.01 | 3183103.49 | 3302621.02 | 5547187.51 | 4798401.45 | 3717751.31 | 6597239.52 | 6061574.47 | 6948734.14 | -1.07 | 0.006 | 1.205 |
| neg | Com_6112 | S-Adenosylhomocysteine | C14 H20 N6 O5 S | 384.12 | 5.37 | 383.11 | 1412214.03 | 8479899.36 | 8288950.45 | 6178555.83 | 3942904.97 | 4475348.15 | 1216048.16 | 1152348.25 | 150123.64 | 3988943.25 | 2839007.67 | 803928.84 | 1.69 | 0.029 | 1.391 |
| neg | Com_6137 | CUMYL-PICA N-pentanoic acid metabolite | C23 H26 N2 O3 | 378.19 | 8.60 | 377.18 | 2782059.38 | 2647029.30 | 3704856.19 | 4334002.45 | 3602316.21 | 2378050.58 | 451354.41 | 580828.30 | 1228554.43 | 501342.78 | 566265.55 | 827759.33 | 2.23 | 0.000 | 1.449 |
| neg | Com_6381 | Prostaglandin E1 Ethanolamide | C22 H39 N O5 | 443.29 | 7.46 | 442.28 | 2643884.38 | 4334046.04 | 6436764.07 | 6206406.48 | 4892044.64 | 4876154.78 | 361435.90 | 567370.93 | 1275650.07 | 397715.39 | 780884.62 | 510032.79 | 2.92 | 0.000 | 1.768 |
| neg | Com_6553 | 5-(tert-butyl)-2-methyl-N-(5-methyl-3-isoxazolyl)-3-furamide | C14 H18 N2 O3 | 262.13 | 6.58 | 261.12 | 3155754.19 | 2199096.05 | 1485025.11 | 3119000.55 | 3593987.92 | 2751838.54 | 594800.49 | 514615.13 | 1297127.90 | 403354.80 | 1092010.54 | 773439.88 | 1.80 | 0.000 | 1.749 |
| neg | Com_6598 | 5-Phosphoribosyl 1-pyrophosphate | C5 H13 O14 P3 | 389.95 | 1.18 | 388.94 | 1601964.22 | 3615183.17 | 4343209.49 | 3549588.86 | 2153004.00 | 2169796.65 | 798633.57 | 949877.16 | 516385.39 | 618996.01 | 1975175.55 | 1441296.71 | 1.47 | 0.003 | 1.636 |
| neg | Com_6608 | Sulfoacetic acid | C2 H4 O5 S | 139.98 | 1.16 | 138.97 | 1502348.61 | 1910785.56 | 1898673.79 | 1814236.86 | 2811402.10 | 2218529.09 | 9476462.44 | 7052844.58 | 10032539.31 | 8926690.16 | 9977328.22 | 13162030.42 | -2.27 | 0.000 | 1.747 |
| neg | Com_6725 | 3-Methylindole | C9 H9 N | 131.07 | 6.29 | 130.07 | 989915.74 | 1755463.32 | 1390008.26 | 1825788.11 | 3722214.33 | 3172937.14 | 5305164.80 | 8142765.19 | 5875235.42 | 8777893.40 | 6575570.29 | 8018901.00 | -1.73 | 0.001 | 1.254 |
| neg | Com_7148 | dTMP | C10 H15 N2 O8 P | 322.06 | 1.36 | 321.05 | 1136955.82 | 2265148.75 | 1371992.98 | 2161292.99 | 1985765.90 | 1975222.31 | 294483.54 | 340321.84 | 300155.02 | 336304.79 | 708188.80 | 378616.56 | 2.21 | 0.000 | 1.566 |
| neg | Com_7352 | PC (2:0/7:0) | C17 H34 N O8 P | 471.22 | 9.29 | 470.22 | 48048.45 | 531756.31 | 326100.71 | 135650.89 | 369223.78 | 327735.68 | 3543907.20 | 2835262.60 | 2575984.26 | 9332194.80 | 1007735.49 | 1375583.06 | -3.57 | 0.000 | 1.752 |
| neg | Com_7882 | Hippuric acid | C9 H9 N O3 | 179.06 | 1.40 | 178.05 | 324060.16 | 1337307.25 | 1080938.19 | 2095103.49 | 875892.53 | 1059667.19 | 1948024.69 | 2758311.85 | 3102635.67 | 1796689.55 | 4510598.75 | 3308238.84 | -1.36 | 0.008 | 1.130 |
| neg | Com_7967 | FMN | C17 H21 N4 O9 P | 456.11 | 7.57 | 455.10 | 894686.30 | 2953183.95 | 2510220.63 | 2545051.93 | 3065893.47 | 2345798.16 | 4061019.79 | 5831587.25 | 7589497.33 | 8205508.88 | 7149464.34 | 6844632.07 | -1.47 | 0.001 | 1.284 |
| neg | Com_8040 | PC (2:0/16:1) | C26 H50 N O8 P | 595.35 | 13.26 | 594.34 | 302798.44 | 191212.40 | 269905.77 | 506196.51 | 152873.34 | 154723.85 | 538648.90 | 735685.74 | 6967291.93 | 899645.61 | 652397.88 | 454783.63 | -2.70 | 0.018 | 1.096 |
| neg | Com_8199 | Glutathione | C10 H17 N3 O6 S | 307.08 | 1.37 | 306.08 | 311551.89 | 1990092.30 | 589895.99 | 1210794.80 | 1438305.20 | 2661170.25 | 4947885.24 | 7573618.34 | 1739413.04 | 3726924.92 | 5730568.31 | 4525893.72 | -1.78 | 0.007 | 1.201 |
| neg | Com_8790 | 15-Deoxy-Δ12,14-prostaglandin D2 | C20 H30 O4 | 316.20 | 9.62 | 315.19 | 1306486.43 | 4063869.62 | 839223.83 | 4337293.98 | 1842752.62 | 4996240.86 | 299139.53 | 274906.22 | 210123.62 | 820990.88 | 395734.75 | 347973.37 | 2.89 | 0.001 | 1.337 |
| neg | Com_8825 | 5,6-Dihydroxyindole-2-Carboxylic Acid | C9 H7 N O4 | 193.04 | 5.57 | 192.03 | 309624.26 | 901119.74 | 1649369.39 | 2099416.83 | 1666490.30 | 1153572.05 | 2210651.71 | 2397557.67 | 4089142.43 | 2472973.53 | 4009206.72 | 4079790.58 | -1.31 | 0.013 | 1.110 |
| neg | Com_8913 | 5-Hydroxytryptophol | C10 H11 N O2 | 177.08 | 0.57 | 176.07 | 636964.56 | 1981082.50 | 1781475.07 | 1588472.26 | 2712394.04 | 2594703.08 | 3945978.56 | 3140358.56 | 4646589.78 | 3492913.95 | 4680271.22 | 3682321.60 | -1.06 | 0.011 | 1.364 |
| neg | Com_9179 | trans-2-Methyl-2-pentenoic Acid | C6 H10 O2 | 114.07 | 2.30 | 113.06 | 337529.91 | 3728899.48 | 1823122.99 | 4034207.06 | 1554112.16 | 1871327.68 | 543219.61 | 525261.76 | 572837.38 | 660394.92 | 555492.18 | 623665.34 | 1.94 | 0.030 | 1.520 |
| neg | Com_9446 | Ouabain | C29 H44 O12 | 584.28 | 7.10 | 583.27 | 539486.23 | 1371322.94 | 1775489.75 | 1288333.88 | 1773426.76 | 1775959.03 | 6306383.50 | 5087379.00 | 4343073.39 | 6207862.60 | 6351682.37 | 4928047.45 | -1.96 | 0.000 | 1.471 |
| neg | Com_9533 | 1-(2,4-dihydroxyphenyl)-2-(3,5-dimethoxyphenyl)propan-1-one | C17 H18 O5 | 302.12 | 9.06 | 301.11 | 467593.68 | 1101267.38 | 273199.34 | 1012428.34 | 366541.96 | 317714.90 | 87214.56 | 86418.59 | 187949.01 | 76368.65 | 87544.17 | 157060.36 | 2.37 | 0.001 | 1.155 |
| neg | Com_9672 | Glyceraldehyde 3 phosphate | C3 H7 O6 P | 170.00 | 1.18 | 168.99 | 1128903.69 | 3456712.98 | 4043329.14 | 3461282.69 | 3804437.08 | 4291243.14 | 496793.48 | 501082.97 | 465435.71 | 622898.95 | 2315292.14 | 581659.76 | 2.02 | 0.001 | 1.929 |
| neg | Com_9850 | [4-(1H-indol-4-yl)piperazino](2-thienyl)methanone | C17 H17 N3 O S | 311.11 | 5.78 | 310.11 | 30666.04 | 100268.37 | 48371.81 | 121184.48 | 68547.46 | 137895.28 | 5870305.89 | 4367528.29 | 3796648.38 | 3434100.45 | 1071412.70 | 2210197.36 | -5.36 | 0.000 | 1.777 |
| neg | Com_10157 | 3-[3-(beta-D-Glucopyranosyloxy)-2-methoxyphenyl]propanoic acid | C16 H22 O9 | 358.12 | 7.55 | 357.12 | 110120.35 | 180072.61 | 171697.01 | 140164.80 | 446379.68 | 209357.42 | 1721405.05 | 905206.41 | 863109.79 | 949305.27 | 1113261.22 | 780367.46 | -2.33 | 0.000 | 1.575 |
| neg | Com_10314 | Agnuside | C22 H26 O11 | 466.15 | 11.55 | 465.15 | 681357.03 | 1788320.30 | 2566538.84 | 1930564.43 | 1224750.29 | 1962850.41 | 127954.53 | 112874.63 | 146841.53 | 131973.27 | 129514.85 | 131497.98 | 3.70 | 0.000 | 1.717 |
| neg | Com_10388 | 12-Epileukotriene B4 | C20 H32 O4 | 372.20 | 7.18 | 371.19 | 447853.31 | 968906.10 | 577503.94 | 1065870.61 | 1458223.90 | 1141018.40 | 4226687.08 | 4587256.24 | 5278654.96 | 2377408.26 | 2168500.56 | 2059337.98 | -1.87 | 0.000 | 1.496 |
| neg | Com_10520 | 3-Nitro-L-Tyrosine | C9 H10 N2 O5 | 226.06 | 1.23 | 225.05 | 356837.78 | 1331808.75 | 932721.76 | 829833.55 | 925694.32 | 798750.77 | 2686535.92 | 1445268.97 | 2301895.57 | 2903120.03 | 2907369.95 | 3069611.71 | -1.57 | 0.001 | 1.433 |
| neg | Com_10591 | N1-{4-methyl-2-[(3,4,5-trimethoxybenzylidene)amino]phenyl}acetamide | C19 H22 N2 O4 | 342.15 | 5.73 | 341.15 | 361074.55 | 446479.10 | 388738.67 | 539628.04 | 790186.23 | 621364.71 | 3769944.41 | 2916657.99 | 3543964.44 | 5119247.23 | 1733455.72 | 1974058.68 | -2.60 | 0.000 | 1.719 |
| neg | Com_10774 | 15(R)-Prostaglandin E2 | C20 H32 O5 | 352.23 | 10.45 | 351.22 | 122376.13 | 945064.85 | 3348497.82 | 1207401.09 | 804217.24 | 1089372.87 | 3888167.33 | 2748559.78 | 3510784.97 | 3006264.71 | 2336019.04 | 3001597.83 | -1.30 | 0.035 | 1.226 |
| neg | Com_10860 | N1-(3-pyridyl)-2,3,4,5,6-pentamethylbenzene-1-sulfonamide | C16 H20 N2 O2 S | 304.13 | 9.41 | 303.12 | 41211.18 | 315357.02 | 166439.26 | 131671.59 | 396072.52 | 103152.75 | 4173958.15 | 4709091.80 | 3020189.15 | 3309454.76 | 2794786.97 | 5185523.10 | -4.33 | 0.000 | 1.980 |
| neg | Com_11275 | Phenylpropiolic acid | C9 H6 O2 | 146.04 | 8.50 | 145.03 | 134587.15 | 467479.72 | 678600.30 | 431441.10 | 401969.94 | 420941.29 | 1568833.43 | 1426504.79 | 4575753.52 | 1510699.61 | 2647911.07 | 3351174.66 | -2.57 | 0.000 | 1.647 |
| neg | Com_11967 | 1a,1b-Dihomo prostaglandin F2α | C22 H38 O5 | 418.24 | 6.38 | 417.24 | 561726.67 | 375211.12 | 615047.95 | 850651.54 | 435535.33 | 215667.98 | 91679.13 | 94265.79 | 103655.24 | 109270.46 | 97372.94 | 102844.09 | 2.35 | 0.000 | 1.265 |
| neg | Com_11991 | 3,4-Dihydroxyphenylpropionic acid | C9 H10 O4 | 182.06 | 6.44 | 181.05 | 148601.27 | 258687.79 | 530479.58 | 345103.43 | 340909.34 | 220980.96 | 747903.08 | 931988.33 | 1339893.31 | 820659.07 | 848552.13 | 1013471.05 | -1.63 | 0.000 | 1.328 |
| neg | Com_12615 | 3-Indoleacrylic acid | C11 H9 N O2 | 187.06 | 6.28 | 186.06 | 380773.98 | 638333.36 | 444061.61 | 584341.15 | 1252409.01 | 870106.01 | 2025289.76 | 2567945.15 | 2016847.67 | 3152287.62 | 1972917.73 | 3121215.50 | -1.83 | 0.000 | 1.329 |
| neg | Com_12621 | 15(R),19(R)-Hydroxy prostaglandin F2α | C20 H34 O6 | 370.24 | 9.73 | 369.23 | 108494.26 | 354368.84 | 401506.94 | 543050.99 | 526066.72 | 265919.43 | 2816606.17 | 2701213.70 | 3725088.13 | 3641370.85 | 3870765.56 | 3113386.83 | -3.18 | 0.000 | 1.620 |
| neg | Com_12635 | Glycochenodeoxycholic acid sodium salt | C26 H43 N O5 Na | 472.30 | 7.39 | 471.29 | 638589.11 | 927486.47 | 875654.90 | 862241.74 | 1323893.14 | 999020.55 | 266496.17 | 293814.61 | 365986.47 | 410260.68 | 471475.88 | 342039.63 | 1.39 | 0.000 | 1.429 |
| neg | Com_12675 | O-Aceyl-L-Serine | C5 H9 N O4 | 147.05 | 6.54 | 146.05 | 144071.91 | 714457.83 | 275083.43 | 460923.94 | 373836.48 | 492831.10 | 3743534.26 | 3297044.56 | 2831711.37 | 2299507.09 | 1414076.11 | 2128729.10 | -2.67 | 0.000 | 1.493 |
| neg | Com_12801 | Citicoline | C14 H26 N4 O11 P2 | 488.11 | 1.14 | 487.10 | 548129.47 | 2144448.66 | 1673283.92 | 2252332.21 | 1682215.49 | 2162071.61 | 746928.88 | 608022.04 | 722634.84 | 668568.11 | 1318156.92 | 919849.04 | 1.07 | 0.026 | 1.439 |
| neg | Com_12926 | Benzyl 6-O-beta-D-glucopyranosyl-beta-D-glucopyranoside | C19 H28 O11 | 432.16 | 14.97 | 431.16 | 408646.25 | 763224.92 | 1977592.57 | 1780318.29 | 1497341.03 | 2406096.59 | 2135574.36 | 2045601.53 | 3317408.12 | 1813069.72 | 4240845.14 | 4169933.46 | -1.00 | 0.037 | 1.106 |
| neg | Com_12948 | Δ17-6-keto prostaglandin F1α | C20 H32 O6 | 368.22 | 9.76 | 367.21 | 96938.95 | 729995.51 | 959113.02 | 1064829.77 | 1096976.15 | 1059230.99 | 2178833.35 | 1769805.05 | 3546598.00 | 1918445.74 | 1862399.04 | 3120427.87 | -1.52 | 0.022 | 1.193 |
| neg | Com_13034 | 2-{1-[2-(4-benzhydrylpiperazino)-2-oxoethyl]cyclopentyl}acetic acid | C26 H32 N2 O3 | 420.24 | 9.65 | 419.23 | 566839.13 | 645004.30 | 834753.58 | 1021709.69 | 790436.52 | 600724.31 | 145548.58 | 130861.05 | 242117.62 | 133510.24 | 228320.14 | 208753.33 | 2.03 | 0.000 | 1.366 |
| neg | Com_13156 | Equol | C15 H14 O3 | 242.09 | 7.72 | 241.09 | 732033.31 | 1538275.95 | 1301548.04 | 1646321.19 | 1635455.50 | 2748383.62 | 94797.56 | 138465.33 | 200616.21 | 169294.11 | 153842.53 | 149790.48 | 3.40 | 0.000 | 2.033 |
| neg | Com_13163 | 11(Z),14(Z)-Eicosadienoic Acid | C20 H36 O2 | 308.27 | 15.29 | 307.26 | 247317.76 | 1061546.78 | 1104255.11 | 824629.02 | 823273.84 | 1400195.04 | 1797358.70 | 2017664.52 | 1074561.62 | 3020487.69 | 2036086.01 | 1479385.47 | -1.06 | 0.023 | 1.033 |
| neg | Com_13180 | Prostaglandin K2 | C20 H30 O5 | 350.21 | 11.83 | 349.20 | 390587.59 | 1714616.12 | 666278.91 | 1446727.81 | 1627287.75 | 1143123.50 | 3006196.89 | 2072690.29 | 1899693.68 | 1524607.61 | 2068898.71 | 4226149.38 | -1.08 | 0.020 | 1.371 |
| neg | Com_13328 | MN-18 N-(5-hydroxypentyl) metabolite | C23 H23 N3 O2 | 373.18 | 6.95 | 372.17 | 636554.79 | 767784.02 | 971724.07 | 1511027.34 | 1073588.94 | 855964.80 | 232361.29 | 161008.96 | 518318.62 | 262840.23 | 286598.81 | 167386.78 | 1.84 | 0.000 | 1.432 |
| neg | Com_13854 | Protectin D1 | C22 H32 O4 | 396.21 | 5.93 | 395.21 | 735250.23 | 798457.95 | 1018283.10 | 1446222.26 | 1395509.47 | 869930.44 | 322756.05 | 245682.56 | 549738.72 | 239143.30 | 667220.01 | 324016.06 | 1.42 | 0.001 | 1.430 |
| neg | Com_14992 | Sorbitan monooleate | C24 H44 O6 | 428.31 | 12.39 | 427.31 | 751172.95 | 242252.25 | 286589.51 | 296486.74 | 293291.24 | 224752.56 | 2035077.90 | 650993.61 | 367358.65 | 752942.66 | 3049990.06 | 346633.90 | -1.78 | 0.044 | 1.205 |
| neg | Com_15007 | 17(S)-HpDHA | C22 H32 O4 | 396.20 | 7.52 | 395.19 | 239454.56 | 300119.55 | 244843.54 | 406763.27 | 658882.17 | 415948.07 | 2668115.34 | 1244420.01 | 1391181.17 | 2106942.25 | 1163716.96 | 1092127.76 | -2.09 | 0.000 | 1.494 |
| neg | Com_15542 | 11-dehydro Thromboxane B2 | C20 H32 O6 | 350.21 | 11.13 | 349.20 | 158614.04 | 319687.83 | 683461.60 | 510422.83 | 338586.06 | 321491.42 | 489943.67 | 494777.23 | 1691359.83 | 385515.63 | 1467950.45 | 1357719.47 | -1.34 | 0.033 | 1.090 |
| neg | Com_16051 | Deoxyinosine | C10 H12 N4 O4 | 252.08 | 5.55 | 251.08 | 138591.73 | 718632.81 | 359393.07 | 547208.27 | 474876.78 | 830343.00 | 2068086.91 | 2164880.16 | 2208842.54 | 1419769.03 | 1130929.34 | 2017986.69 | -1.84 | 0.002 | 1.505 |
| neg | Com_16216 | L-Tryptophan | C11 H12 N2 O2 | 204.09 | 7.68 | 203.08 | 277285.40 | 636278.01 | 772346.07 | 770171.47 | 963381.36 | 876481.20 | 1814881.25 | 1673201.06 | 1829305.45 | 2199465.38 | 1816235.03 | 1989446.93 | -1.40 | 0.002 | 1.357 |
| neg | Com_17154 | (2S)-4-Oxo-2-phenyl-3,4-dihydro-2H-chromen-7-yl beta-D-glucopyranoside | C21 H22 O8 | 402.13 | 7.55 | 401.12 | 54285.93 | 62528.27 | 86405.12 | 84551.54 | 221232.37 | 84491.58 | 1012569.88 | 564526.69 | 414225.84 | 465355.60 | 485482.08 | 426013.48 | -2.50 | 0.000 | 1.743 |
| neg | Com_17558 | N-(2-morpholinophenyl)-2,1,3-benzoxadiazole-4-sulfonamide | C16 H16 N4 O4 S | 360.09 | 9.47 | 359.08 | 117352.47 | 98578.07 | 91115.29 | 106963.04 | 103988.60 | 103513.00 | 230904.30 | 260935.00 | 208103.61 | 213839.03 | 245151.97 | 186842.10 | -1.11 | 0.000 | 1.016 |
| neg | Com_18476 | Cetirizine N-oxide | C21 H25 Cl N2 O4 | 404.15 | 5.57 | 403.14 | 108867.03 | 80390.92 | 71040.07 | 75171.55 | 89019.62 | 98946.18 | 500715.04 | 443125.58 | 427255.51 | 499346.22 | 415036.42 | 452382.69 | -2.39 | 0.000 | 1.759 |
| neg | Com_19274 | L-Homocystine | C8 H16 N2 O4 S2 | 268.05 | 8.15 | 267.04 | 146698.30 | 66013.90 | 58959.93 | 81074.80 | 94260.15 | 76813.00 | 654093.00 | 220947.24 | 294857.71 | 242089.03 | 145931.22 | 108251.69 | -1.67 | 0.008 | 1.079 |
| neg | Com_19330 | Ip7G | C16 H23 N5 O5 | 365.17 | 6.90 | 364.16 | 235874.94 | 452519.46 | 389827.45 | 502551.40 | 874456.81 | 654482.82 | 1118449.26 | 926160.26 | 1245403.87 | 1221181.64 | 1371508.88 | 1235815.45 | -1.19 | 0.003 | 1.216 |
| neg | Com_19388 | Protoporphyrin IX | C34 H34 N4 O4 | 562.26 | 6.89 | 561.25 | 155509.41 | 271942.20 | 136582.78 | 207359.01 | 369143.21 | 257496.97 | 741811.81 | 920684.51 | 1013787.84 | 861748.76 | 904911.03 | 1093735.02 | -1.99 | 0.000 | 1.812 |
| neg | Com_19658 | Dimetghyl 4-Hydroxyisophthalate | C10 H10 O5 | 210.05 | 6.61 | 209.05 | 44862.41 | 149382.33 | 128365.48 | 123087.95 | 282994.64 | 172395.69 | 860407.27 | 879464.86 | 821590.79 | 490407.07 | 1281525.88 | 938390.18 | -2.55 | 0.000 | 1.686 |
| neg | Com_19678 | N-{6-[4-(tert-butyl)phenoxy]-3-pyridinyl}-4-(trifluoromethyl)benzamide | C23 H21 F3 N2 O2 | 414.15 | 9.41 | 413.14 | 35552.08 | 78072.05 | 72501.76 | 79537.66 | 95270.02 | 87340.19 | 849003.59 | 852228.53 | 794222.97 | 692344.03 | 555986.38 | 1497277.41 | -3.55 | 0.000 | 2.030 |
| neg | Com_19942 | 2-cyano-N-(3-oxo-1,3-dihydro-2-benzofuran-5-yl)benzenesulfonamide | C15 H10 N2 O4 S | 360.04 | 8.63 | 359.03 | 178365.28 | 470105.50 | 385276.03 | 354507.92 | 660379.58 | 495731.50 | 834211.00 | 813495.83 | 1134383.16 | 1152660.78 | 1067042.34 | 1020936.01 | -1.24 | 0.003 | 1.083 |

*MW is shorted for molecular weight; FC is abbreviated for fold-change; VIP stands for variable importance in the projection.
