# Supplementary material for: A complex metabolic network and its biomarkers regulate laccase production in white-rot fungus Cerrena unicolor 87613
Source: Microb Cell Fact. 2024 Jun 8;23:167. doi: 10.1186/s12934-024-02443-9 (PMC11162070; doi:10.1186/s12934-024-02443-9)
Supplement: Supplementary file 8 — Supplementary Material 8 [file 12934_2024_2443_MOESM8_ESM.docx]

**Table S2 Collection of differentially expressed genes (DEGs) in the cultures from fructose-cultivated day 6 (FCd-6) versus those from FCd-10 (The raw data are available from the NCBI Sequence Read Archive under the GEO accession number GSE236542).**

| **Gene** | | | | | |  | **FPKM** | | | | | | **Log2FC*** | ***P*-value** | **Gene Description** |
| --- | --- | --- | --- | --- | --- | --- | --- | --- | --- | --- | --- | --- | --- | --- | --- |
| **ID** | **Chr** | **Start** | **End** | **Strand** | **Length** |  | **FCd-6_1** | **FCd-6_2** | **FCd-6_3** | **FCd-10_1** | **FCd-10_2** | **FCd-10_3** |  |  |  |
| A00003 | Contig10 | 11069 | 14786 | - | 2976 |  | 305.05 | 193.69 | 325.07 | 56.08 | 91.60 | 62.49 | 1.97 | 0.000 | - && Q922B2.2 RecName: Full=Aspartate--tRNA ligase, cytoplasmic; AltName: Full=Aspartyl-tRNA synthetase; Short=AspRS && PF01336:OB-fold nucleic acid binding domain\|PF00152:tRNA synthetases class II (D, K and N)\|PF09924:Uncharacterized conserved protein (DUF2156) |
| A00004 | Contig10 | 15619 | 17631 | + | 1425 |  | 44.15 | 37.48 | 51.28 | 15.09 | 24.17 | 19.95 | 1.17 | 0.000 | - && - && PF09130:Domain of unknown function (DUF1932) |
| A00011 | Contig10 | 47905 | 49473 | - | 1569 |  | 4.34 | 7.49 | 3.20 | 2.26 | 2.15 | 2.63 | 1.09 | 0.003 | - && - && - |
| A00015 | Contig10 | 61068 | 63236 | - | 1728 |  | 39.03 | 29.80 | 78.24 | 116.17 | 70.21 | 129.61 | -1.10 | 0.001 | - && Q10177.1 RecName: Full=Manganese transporter pdt1 && PF01566:Natural resistance-associated macrophage protein |
| A00019 | Contig10 | 78811 | 80234 | - | 1086 |  | 14.53 | 13.31 | 9.76 | 6.37 | 7.67 | 3.47 | 1.10 | 0.001 | - && P54072.1 RecName: Full=Uncharacterized transporter YLR152C && PF03547:Membrane transport protein |
| A00026 | Contig10 | 97616 | 100538 | + | 1770 |  | 11.75 | 6.47 | 12.13 | 41.47 | 33.85 | 38.23 | -1.90 | 0.000 | - && Q99385.1 RecName: Full=Vacuolar calcium ion transporter; AltName: Full=High copy number undoes manganese protein 1; AltName: Full=Manganese resistance 1 protein; AltName: Full=Vacuolar Ca(2+)/H(+) exchanger && PF01699:Sodium/calcium exchanger protein |
| A00032 | Contig10 | 122082 | 122895 | - | 636 |  | 27.75 | 49.25 | 37.63 | 95.55 | 97.58 | 99.49 | -1.35 | 0.000 | - && - && - |
| A00041 | Contig10 | 152656 | 153927 | + | 1215 |  | 107.12 | 99.73 | 117.51 | 248.40 | 313.04 | 253.38 | -1.33 | 0.000 | - && P18773.2 RecName: Full=Esterase && PF07859:alpha/beta hydrolase fold |
| A00042 | Contig10 | 154689 | 156831 | + | 1392 |  | 83.80 | 76.08 | 72.12 | 28.28 | 20.51 | 37.89 | 1.42 | 0.000 | - && - && PF11710:G protein-coupled glucose receptor regulating Gpa2 |
| A00052 | Contig10 | 190783 | 191675 | - | 588 |  | 2512.87 | 1831.06 | 2671.05 | 1304.97 | 1268.68 | 695.35 | 1.10 | 0.000 | - && - && - |
| A00055 | Contig10 | 198482 | 200704 | + | 1836 |  | 5651.25 | 6775.66 | 2322.35 | 123.39 | 51.65 | 191.91 | 5.33 | 0.000 | - && - && - |
| A00075 | Contig10 | 304254 | 305074 | + | 660 |  | 5.98 | 3.50 | 1.80 | 5.67 | 5.39 | 12.76 | -1.08 | 0.032 | - && - && - |
| A00076 | Contig10 | 309223 | 310064 | + | 684 |  | 61.70 | 98.33 | 86.41 | 210.78 | 212.13 | 152.17 | -1.22 | 0.000 | - && - && - |
| A00077 | Contig10 | 310486 | 312516 | - | 1389 |  | 3.12 | 4.91 | 3.35 | 16.56 | 24.26 | 7.52 | -2.09 | 0.000 | - && P0CT90.1 RecName: Full=3-O-methyltransferase 2; Short=Mtrase 2 && PF00891:O-methyltransferase |
| A00100 | Contig10 | 393088 | 393885 | + | 516 |  | 20.64 | 21.60 | 9.38 | 5.80 | 6.89 | 6.63 | 1.42 | 0.000 | - && - && - |
| A00109 | Contig10 | 421736 | 423393 | + | 1323 |  | 32.05 | 43.48 | 24.93 | 14.91 | 12.81 | 15.92 | 1.20 | 0.000 | - && Q9C1T1.1 RecName: Full=3-phytase A; AltName: Full=3 phytase A; AltName: Full=Myo-inositol hexakisphosphate phosphohydrolase A; AltName: Full=Myo-inositol-hexaphosphate 3-phosphohydrolase A; Flags: Precursor && PF00328:Histidine phosphatase superfamily (branch 2) |
| A00117 | Contig10 | 461351 | 462583 | + | 1119 |  | 3.70 | 3.23 | 3.84 | 10.95 | 5.52 | 12.63 | -1.43 | 0.000 | - && - && PF09949:Uncharacterized conserved protein (DUF2183) |
| A00122 | Contig10 | 476439 | 477602 | + | 969 |  | 5.19 | 5.70 | 4.24 | 2.51 | 2.32 | 0.72 | 1.45 | 0.002 | - && - && - |
| A00123 | Contig10 | 478325 | 479525 | - | 1017 |  | 168.70 | 157.25 | 112.73 | 56.72 | 26.23 | 51.43 | 1.71 | 0.000 | - && - && - |
| A00135 | Contig10 | 537718 | 538506 | - | 618 |  | 12.76 | 24.20 | 13.89 | 76.25 | 76.49 | 37.21 | -1.90 | 0.000 | - && Q01358.1 RecName: Full=Protein bli-3 && PF16242:Pyridoxamine 5'-phosphate oxidase like |
| A00137 | Contig10 | 541880 | 546350 | - | 2046 |  | 25.69 | 22.28 | 23.35 | 138.78 | 93.52 | 96.98 | -2.21 | 0.000 | - && Q9HTH5.1 RecName: Full=HTH-type transcriptional regulator CdhR; AltName: Full=Carnitine catabolism transcriptional activator && PF01965:DJ-1/PfpI family |
| A00149 | Contig10 | 585098 | 585531 | - | 258 |  | 35.54 | 208.56 | 57.71 | 610.26 | 483.65 | 334.37 | -2.24 | 0.004 | - && - && - |
| A00155 | Contig10 | 612807 | 613266 | + | 342 |  | 45.84 | 25.24 | 66.51 | 18.32 | 17.52 | 6.67 | 1.69 | 0.000 | - && - && - |
| A00170 | Contig10 | 689833 | 697942 | - | 6930 |  | 1.64 | 1.35 | 1.73 | 3.05 | 4.66 | 3.50 | -1.25 | 0.000 | - && - && - |
| A00172 | Contig10 | 701880 | 703240 | - | 1011 |  | 10.14 | 8.24 | 9.49 | 4.90 | 6.39 | 1.65 | 1.11 | 0.007 | - && - && - |
| A00187 | Contig10 | 767085 | 772222 | + | 3594 |  | 74.65 | 57.18 | 64.15 | 36.29 | 35.48 | 19.56 | 1.10 | 0.000 | - && Q9C1W9.1 RecName: Full=DNA ligase 3; AltName: Full=DNA ligase III; AltName: Full=Polydeoxyribonucleotide synthase [ATP] 3 && PF01068:ATP dependent DNA ligase domain\|PF04679:ATP dependent DNA ligase C terminal region\|PF13883:Pyridoxamine 5'-phosphate oxidase\|PF04675:DNA ligase N terminus |
| A00190 | Contig10 | 782147 | 786459 | + | 3261 |  | 38.04 | 37.10 | 39.83 | 74.97 | 67.26 | 93.92 | -1.04 | 0.000 | - && O14134.1 RecName: Full=mRNA export factor elf1 && PF00005:ABC transporter\|PF00385:Chromo (CHRromatin Organisation MOdifier) domain |
| A00194 | Contig10 | 797238 | 800077 | - | 2109 |  | 120.34 | 143.85 | 83.55 | 55.19 | 38.44 | 53.85 | 1.24 | 0.000 | - && Q92222.3 RecName: Full=Endochitinase B; AltName: Full=Chitinase B >G5EAZ3.1 RecName: Full=Endochitinase B; AltName: Full=Chitinase B && PF00734:Fungal cellulose binding domain\|PF00704:Glycosyl hydrolases family 18 |
| A00196 | Contig10 | 803392 | 811400 | + | 4650 |  | 22.29 | 22.26 | 24.50 | 45.08 | 55.82 | 115.48 | -1.65 | 0.000 | - && P22944.2 RecName: Full=Nitrite reductase [NAD(P)H] && PF13806:Rieske-like [2Fe-2S] domain\|PF00009:Elongation factor Tu GTP binding domain\|PF03460:Nitrite/Sulfite reductase ferredoxin-like half domain\|PF01077:Nitrite and sulphite reductase 4Fe-4S domain\|PF04324:BFD-like [2Fe-2S] binding domain\|PF07992:Pyridine nucleotide-disulphide oxidoreductase |
| A00198 | Contig10 | 816527 | 818823 | - | 1557 |  | 7.22 | 6.38 | 7.33 | 62.09 | 87.18 | 91.82 | -3.53 | 0.000 | - && P51687.2 RecName: Full=Sulfite oxidase, mitochondrial; Flags: Precursor && PF03404:Mo-co oxidoreductase dimerisation domain\|PF00174:Oxidoreductase molybdopterin binding domain |
| A00214 | Contig10 | 891009 | 892632 | - | 1209 |  | 9.46 | 11.87 | 11.33 | 34.80 | 40.03 | 29.69 | -1.68 | 0.000 | - && Q06816.2 RecName: Full=Putative epoxide hydrolase; AltName: Full=Epoxide hydratase; Flags: Precursor && PF06441:Epoxide hydrolase N terminus |
| A00219 | Contig10 | 904270 | 905214 | - | 837 |  | 278.49 | 243.59 | 180.95 | 43.12 | 34.78 | 75.91 | 2.19 | 0.000 | - && - && - |
| A00222 | Contig10 | 913296 | 918317 | + | 2937 |  | 35.39 | 23.14 | 32.60 | 9.33 | 8.54 | 4.60 | 2.02 | 0.000 | - && G3Y416.1 RecName: Full=Cytochrome P450 monooxygenase yanC; AltName: Full=Yanuthone D synthesis protein C; Flags: Precursor && PF00067:Cytochrome P450 |
| A00229 | Contig10 | 949934 | 951136 | + | 1026 |  | 22.20 | 49.41 | 14.87 | 10.57 | 3.65 | 10.09 | 1.83 | 0.000 | - && Q9UT59.1 RecName: Full=Putative uncharacterized oxidoreductase C513.07 && PF01370:NAD dependent epimerase/dehydratase family |
| A00245 | Contig10 | 1010381 | 1010863 | - | 483 |  | 13.47 | 14.55 | 14.75 | 9.10 | 1.74 | 4.54 | 1.48 | 0.001 | - && - && - |
| A00261 | Contig10 | 1060800 | 1062019 | - | 1005 |  | 130.39 | 126.76 | 123.53 | 5.95 | 5.03 | 4.80 | 4.59 | 0.000 | - && - && PF00106:short chain dehydrogenase |
| A00272 | Contig10 | 1088000 | 1089452 | - | 1251 |  | 0.63 | 1.28 | 0.66 | 1.57 | 1.27 | 3.72 | -1.35 | 0.022 | - && - && - |
| A00276 | Contig10 | 1098771 | 1105813 | + | 3729 |  | 103.20 | 52.85 | 183.53 | 797.46 | 1219.57 | 2060.01 | -3.59 | 0.000 | - && O13437.1 RecName: Full=Formate dehydrogenase; Short=FDH; AltName: Full=NAD-dependent formate dehydrogenase && PF03124:EXS family\|PF02826:D-isomer specific 2-hydroxyacid dehydrogenase, NAD binding domain\|PF00389:D-isomer specific 2-hydroxyacid dehydrogenase, catalytic domain |
| A00286 | Contig10 | 1144822 | 1148779 | + | 2817 |  | 66.51 | 54.60 | 62.91 | 110.02 | 125.51 | 191.03 | -1.21 | 0.000 | - && Q8SW18.1 RecName: Full=Homeobox protein HD-10; AltName: Full=EcHD-10 && PF00046:Homeobox domain |
| A00311 | Contig10 | 1244084 | 1244518 | + | 315 |  | 39.44 | 215.75 | 48.72 | 1183.09 | 793.46 | 251.02 | -2.87 | 0.003 | - && - && - |
| A00316 | Contig10 | 1272974 | 1274482 | + | 1329 |  | 99.05 | 112.02 | 88.94 | 227.23 | 256.11 | 488.71 | -1.70 | 0.000 | - && Q6C9Y4.1 RecName: Full=Carbamoyl-phosphate synthase arginine-specific small chain; Short=CPS-A; AltName: Full=Arginine-specific carbamoyl-phosphate synthetase, glutamine chain && PF00988:Carbamoyl-phosphate synthase small chain, CPSase domain\|PF00117:Glutamine amidotransferase class-I |
| A00320 | Contig10 | 1292415 | 1293825 | + | 1080 |  | 5.93 | 7.53 | 10.15 | 68.91 | 54.35 | 84.83 | -3.14 | 0.000 | - && - && - |
| A00321 | Contig10 | 1294385 | 1295804 | + | 1011 |  | 29.36 | 18.37 | 46.17 | 157.40 | 200.46 | 125.69 | -2.36 | 0.000 | - && - && - |
| A00324 | Contig10 | 1302026 | 1303583 | + | 1032 |  | 38.03 | 56.91 | 25.49 | 17.94 | 15.78 | 16.16 | 1.27 | 0.000 | - && P58744.2 PUTATIVE PSEUDOGENE: RecName: Full=Putative 2,5-diketo-D-gluconic acid reductase A; Short=2,5-DKG reductase A; Short=2,5-DKGR A; Short=25DKGR-A; AltName: Full=AKR5C && PF00248:Aldo/keto reductase family |
| A00328 | Contig10 | 1313661 | 1314877 | - | 1053 |  | 74.44 | 59.68 | 48.06 | 29.74 | 17.69 | 21.50 | 1.40 | 0.000 | - && - && - |
| A00338 | Contig10 | 1371631 | 1372657 | + | 489 |  | 0.20 | 0.62 | 0.00 | 1.53 | 2.87 | 0.90 | -2.70 | 0.017 | - && - && - |
| A00342 | Contig10 | 1391188 | 1398922 | + | 2991 |  | 2.31 | 1.64 | 1.68 | 9.19 | 18.47 | 11.18 | -2.79 | 0.000 | - && O43112.2 RecName: Full=Phosphoenolpyruvate carboxykinase [ATP] && PF01293:Phosphoenolpyruvate carboxykinase |
| A00347 | Contig11 | 1083906 | 1087092 | - | 879 |  | 1.12 | 1.37 | 0.94 | 2.77 | 4.37 | 1.90 | -1.40 | 0.010 | - && - && - |
| A00350 | Contig11 | 1095875 | 1096324 | + | 321 |  | 49.15 | 27.21 | 48.95 | 3.50 | 3.21 | 3.83 | 3.57 | 0.000 | - && - && - |
| A00358 | Contig11 | 1117533 | 1118479 | - | 774 |  | 36.43 | 50.97 | 32.22 | 10.27 | 15.24 | 14.40 | 1.58 | 0.000 | - && P45207.1 RecName: Full=Stringent starvation protein A homolog && PF13410:Glutathione S-transferase, C-terminal domain\|PF13417:Glutathione S-transferase, N-terminal domain |
| A00360 | Contig11 | 1124100 | 1124651 | - | 552 |  | 0.54 | 1.45 | 0.17 | 1.86 | 3.56 | 2.07 | -1.80 | 0.026 | - && - && - |
| A00365 | Contig11 | 1142716 | 1143714 | - | 888 |  | 170.11 | 149.68 | 184.96 | 628.78 | 415.24 | 891.13 | -1.94 | 0.000 | - && - && PF06687:SUR7/PalI family |
| A00369 | Contig11 | 1170359 | 1171595 | + | 1176 |  | 253.30 | 209.74 | 334.64 | 405.31 | 528.15 | 683.41 | -1.02 | 0.000 | - && - && PF00134:Cyclin, N-terminal domain |
| A00376 | Contig11 | 1203079 | 1205906 | - | 2310 |  | 107.18 | 129.94 | 108.75 | 1076.90 | 593.15 | 681.71 | -2.77 | 0.000 | - && O93967.1 RecName: Full=Phenylalanine ammonia-lyase && PF00221:Aromatic amino acid lyase |
| A00380 | Contig11 | 1227261 | 1231014 | + | 2157 |  | 198.12 | 130.41 | 137.47 | 94.75 | 86.62 | 45.45 | 1.04 | 0.000 | - && - && - |
| A00386 | Contig11 | 1251867 | 1252699 | - | 546 |  | 24.92 | 18.02 | 24.09 | 55.65 | 49.38 | 70.08 | -1.39 | 0.000 | - && - && - |
| A00390 | Contig11 | 1262630 | 1264857 | + | 1998 |  | 109.66 | 96.97 | 115.22 | 210.34 | 220.11 | 308.60 | -1.20 | 0.000 | - && Q5B0C0.1 RecName: Full=Heat shock 70 kDa protein; Flags: Precursor && PF00012:Hsp70 protein |
| A00392 | Contig11 | 1276626 | 1279347 | + | 1761 |  | 20.83 | 18.41 | 22.93 | 33.71 | 47.84 | 73.26 | -1.32 | 0.000 | - && - && - |
| A00397 | Contig11 | 1292436 | 1294770 | - | 1635 |  | 2.89 | 2.64 | 2.79 | 1.14 | 2.06 | 0.38 | 1.22 | 0.015 | - && Q9V4U9.1 RecName: Full=Probable cytochrome P450 6a13; AltName: Full=CYPVIA13 && PF00067:Cytochrome P450 |
| A00399 | Contig11 | 1302065 | 1304381 | + | 1605 |  | 2.09 | 1.88 | 1.82 | 3.67 | 5.54 | 5.52 | -1.35 | 0.000 | - && Q27519.1 RecName: Full=Putative cytochrome P450 CYP13A7 && PF00067:Cytochrome P450 |
| A00416 | Contig11 | 1359825 | 1362501 | + | 1800 |  | 6.52 | 5.02 | 13.60 | 4.73 | 2.34 | 2.29 | 1.43 | 0.000 | - && D4B093.1 RecName: Full=Secreted protein ARB_01864; Flags: Precursor && - |
| A00420 | Contig11 | 1372473 | 1374865 | - | 1779 |  | 2.99 | 2.09 | 2.41 | 0.89 | 0.16 | 0.69 | 2.11 | 0.000 | - && Q9Y758.1 RecName: Full=Cytochrome P450 52A13; AltName: Full=Alkane hydroxylase 2; AltName: Full=Alkane-inducible p450alk 2; AltName: Full=DH-ALK2 [Debaryomyces hansenii] && PF00067:Cytochrome P450 |
| A00421 | Contig11 | 1378151 | 1380386 | - | 1506 |  | 1.05 | 1.13 | 1.58 | 51.71 | 68.87 | 34.67 | -5.37 | 0.000 | - && P09437.2 RecName: Full=Cytochrome b2, mitochondrial; AltName: Full=L-lactate dehydrogenase [Cytochrome]; AltName: Full=L-lactate ferricytochrome C oxidoreductase; Short=L-LCR; Flags: Precursor && PF01070:FMN-dependent dehydrogenase\|PF00173:Cytochrome b5-like Heme/Steroid binding domain |
| A00422 | Contig11 | 1382652 | 1387210 | + | 2454 |  | 4.94 | 8.14 | 5.84 | 78.48 | 92.39 | 44.74 | -3.51 | 0.000 | - && P09437.2 RecName: Full=Cytochrome b2, mitochondrial; AltName: Full=L-lactate dehydrogenase [Cytochrome]; AltName: Full=L-lactate ferricytochrome C oxidoreductase; Short=L-LCR; Flags: Precursor && PF01070:FMN-dependent dehydrogenase\|PF13714:Phosphoenolpyruvate phosphomutase\|PF00173:Cytochrome b5-like Heme/Steroid binding domain |
| A00433 | Contig11 | 36506 | 38275 | - | 1314 |  | 4.65 | 4.20 | 5.14 | 1.78 | 1.50 | 2.40 | 1.30 | 0.000 | - && - && PF13519:von Willebrand factor type A domain |
| A00435 | Contig11 | 41810 | 42507 | - | 639 |  | 0.77 | 1.57 | 0.57 | 2.93 | 3.52 | 1.92 | -1.52 | 0.020 | - && - && - |
| A00440 | Contig11 | 54101 | 55305 | - | 1035 |  | 0.57 | 1.55 | 1.06 | 3.70 | 3.62 | 3.56 | -1.77 | 0.000 | - && - && - |
| A00443 | Contig11 | 58818 | 62940 | - | 3102 |  | 16.27 | 20.74 | 17.20 | 55.04 | 52.24 | 53.89 | -1.57 | 0.000 | - && A2QAN3.1 RecName: Full=Probable beta-galactosidase A; AltName: Full=Lactase A; Flags: Precursor && PF10435:Beta-galactosidase, domain 2\|PF01301:Glycosyl hydrolases family 35\|PF13363:Beta-galactosidase, domain 3\|PF13364:Beta-galactosidase jelly roll domain |
| A00444 | Contig11 | 64117 | 66657 | + | 1617 |  | 4.76 | 2.98 | 3.95 | 60.94 | 84.70 | 53.84 | -4.09 | 0.000 | - && Q9US44.1 RecName: Full=Uncharacterized transporter C1002.16c && PF07690:Major Facilitator Superfamily |
| A00448 | Contig11 | 72027 | 73013 | + | 693 |  | 5.12 | 6.66 | 4.75 | 11.33 | 15.40 | 8.61 | -1.10 | 0.001 | - && - && - |
| A00469 | Contig11 | 157421 | 158476 | - | 1056 |  | 5.88 | 6.46 | 6.83 | 3.19 | 2.66 | 1.58 | 1.37 | 0.000 | - && - && - |
| A00473 | Contig11 | 175952 | 178151 | - | 1764 |  | 120.63 | 113.71 | 113.51 | 49.77 | 46.81 | 59.55 | 1.16 | 0.000 | - && Q9M0X9.1 RecName: Full=4-coumarate--CoA ligase-like 7; AltName: Full=4-coumarate--CoA ligase isoform 6; Short=At4CL6 && PF13193:AMP-binding enzyme C-terminal domain\|PF00501:AMP-binding enzyme |
| A00483 | Contig11 | 212636 | 214700 | + | 1596 |  | 11.12 | 16.35 | 13.85 | 68.54 | 72.55 | 88.14 | -2.47 | 0.000 | - && Q92035.2 RecName: Full=Acetylcholinesterase; Short=AChE; Flags: Precursor && PF00135:Carboxylesterase family |
| A00494 | Contig11 | 256766 | 258280 | + | 945 |  | 105.28 | 109.52 | 69.50 | 11.38 | 9.51 | 6.04 | 3.40 | 0.000 | - && P14065.1 RecName: Full=Glycerol 2-dehydrogenase (NADP(+)); AltName: Full=Galactose-inducible crystallin-like protein 1 && PF00248:Aldo/keto reductase family |
| A00497 | Contig11 | 268119 | 269784 | + | 570 |  | 139.60 | 139.31 | 118.43 | 291.15 | 312.53 | 293.14 | -1.17 | 0.000 | - && Q7SGY6.1 RecName: Full=Succinate dehydrogenase [ubiquinone] cytochrome b small subunit, mitochondrial; Short=CybS; AltName: Full=Succinate-ubiquinone reductase membrane anchor subunit; Flags: Precursor && PF05328:CybS, succinate dehydrogenase cytochrome B small subunit |
| A00506 | Contig11 | 299176 | 299810 | - | 582 |  | 78.27 | 98.84 | 68.90 | 32.77 | 17.69 | 17.79 | 1.85 | 0.000 | - && - && PF11807:Domain of unknown function (DUF3328) |
| A00507 | Contig11 | 301501 | 302809 | + | 1071 |  | 811.84 | 618.45 | 967.66 | 296.29 | 233.81 | 128.89 | 1.86 | 0.000 | - && - && - |
| A00508 | Contig11 | 303642 | 312736 | + | 6231 |  | 80.75 | 81.15 | 97.71 | 36.66 | 26.85 | 16.83 | 1.69 | 0.000 | - && Q5F364.1 RecName: Full=Multidrug resistance-associated protein 1; AltName: Full=ATP-binding cassette sub-family C member 1; AltName: Full=Leukotriene C(4) transporter; Short=LTC4 transporter && PF00326:Prolyl oligopeptidase family\|PF00005:ABC transporter |
| A00514 | Contig11 | 339490 | 343064 | - | 2820 |  | 0.17 | 1.46 | 0.71 | 0.36 | 0.33 | 0.12 | 1.52 | 0.040 | - && - && PF05699:hAT family C-terminal dimerisation region |
| A00523 | Contig11 | 375551 | 378038 | + | 765 |  | 2.84 | 6.56 | 7.05 | 35.57 | 24.11 | 56.67 | -2.82 | 0.000 | - && - && - |
| A00529 | Contig11 | 395477 | 397923 | + | 2214 |  | 4.99 | 7.89 | 5.94 | 16.81 | 16.79 | 16.93 | -1.42 | 0.000 | - && - && - |
| A00532 | Contig11 | 405597 | 408126 | - | 1782 |  | 13.56 | 18.98 | 18.61 | 86.62 | 104.01 | 111.45 | -2.56 | 0.000 | - && P22998.1 RecName: Full=Alpha-amylase; AltName: Full=1,4-alpha-D-glucan glucanohydrolase; Flags: Precursor && PF00128:Alpha amylase, catalytic domain\|PF02806:Alpha amylase, C-terminal all-beta domain\|PF00686:Starch binding domain |
| A00533 | Contig11 | 409490 | 411638 | - | 1536 |  | 11.68 | 32.68 | 17.78 | 1754.02 | 2515.47 | 3278.55 | -6.92 | 0.000 | - && P55306.1 RecName: Full=Catalase && PF00199:Catalase\|PF06628:Catalase-related immune-responsive |
| A00535 | Contig11 | 417282 | 419115 | - | 1362 |  | 17.59 | 16.07 | 17.64 | 6.52 | 7.08 | 5.22 | 1.45 | 0.000 | - && P32470.2 RecName: Full=Chitinase 1; Flags: Precursor && PF00704:Glycosyl hydrolases family 18 |
| A00536 | Contig11 | 420214 | 421289 | - | 696 |  | 1.13 | 0.58 | 1.57 | 2.01 | 3.23 | 2.65 | -1.26 | 0.040 | - && - && - |
| A00537 | Contig11 | 422436 | 424585 | - | 1599 |  | 3.45 | 2.20 | 5.71 | 109.05 | 50.99 | 173.49 | -4.88 | 0.000 | - && Q873X9.1 RecName: Full=Endochitinase B1; AltName: Full=Chitinase B1; Flags: Precursor >E9QRF2.1 RecName: Full=Endochitinase B1; AltName: Full=Chitinase B1; Flags: Precursor && PF00704:Glycosyl hydrolases family 18\|PF02839:Carbohydrate binding domain |
| A00541 | Contig11 | 440441 | 441808 | + | 765 |  | 187.80 | 226.37 | 180.19 | 438.27 | 465.73 | 569.69 | -1.31 | 0.000 | - && Q70KF8.1 RecName: Full=Succinate dehydrogenase [ubiquinone] iron-sulfur subunit, mitochondrial; AltName: Full=Iron-sulfur subunit of complex II; Short=Ip; Flags: Precursor && PF13085:2Fe-2S iron-sulfur cluster binding domain |
| A00542 | Contig11 | 444782 | 447415 | + | 1821 |  | 59.67 | 150.33 | 48.11 | 543.77 | 402.56 | 1672.47 | -3.34 | 0.000 | - && P09794.1 RecName: Full=Alpha-amylase; AltName: Full=1,4-alpha-D-glucan glucanohydrolase; Flags: Precursor && PF00128:Alpha amylase, catalytic domain\|PF02806:Alpha amylase, C-terminal all-beta domain\|PF00686:Starch binding domain |
| A00553 | Contig11 | 488586 | 489487 | + | 573 |  | 1685.37 | 1417.70 | 1418.39 | 635.38 | 569.67 | 635.60 | 1.30 | 0.000 | - && - && - |
| A00554 | Contig11 | 490759 | 491573 | + | 498 |  | 226.71 | 295.72 | 34.67 | 8.07 | 4.51 | 3.88 | 5.08 | 0.000 | - && - && - |
| A00555 | Contig11 | 492181 | 494273 | - | 1764 |  | 113.81 | 88.04 | 72.03 | 36.52 | 24.68 | 24.43 | 1.68 | 0.000 | - && B1Z034.1 RecName: Full=Oxygen-dependent choline dehydrogenase; Short=CDH; Short=CHD; AltName: Full=Betaine aldehyde dehydrogenase; Short=BADH && PF05199:GMC oxidoreductase\|PF00732:GMC oxidoreductase |
| A00560 | Contig11 | 509736 | 511231 | - | 1386 |  | 17.43 | 22.02 | 20.96 | 125.34 | 102.46 | 170.58 | -2.72 | 0.000 | - && - && PF03372:Endonuclease/Exonuclease/phosphatase family\|PF01419:Jacalin-like lectin domain |
| A00567 | Contig11 | 532597 | 534032 | - | 1125 |  | 5.96 | 4.82 | 4.55 | 10.64 | 22.97 | 5.93 | -1.37 | 0.001 | - && Q4WZ70.1 RecName: Full=Chanoclavine-I aldehyde reductase easA; AltName: Full=Ergot alkaloid synthesis protein A; AltName: Full=Old yellow enzyme 3 homolog && PF00724:NADH:flavin oxidoreductase / NADH oxidase family |
| A00576 | Contig11 | 586265 | 587966 | + | 1101 |  | 2.33 | 1.73 | 1.33 | 7.39 | 8.16 | 11.64 | -2.34 | 0.000 | - && - && PF11901:Protein of unknown function (DUF3421)\|PF00092:von Willebrand factor type A domain |
| A00580 | Contig11 | 593393 | 594886 | + | 1494 |  | 34.65 | 32.72 | 35.16 | 61.14 | 71.49 | 73.43 | -1.01 | 0.000 | - && - && - |
| A00583 | Contig11 | 598399 | 601299 | - | 2025 |  | 45.38 | 30.39 | 40.55 | 17.50 | 17.61 | 18.90 | 1.11 | 0.000 | - && Q7XBS0.1 RecName: Full=Urea-proton symporter DUR3; Short=OsDUR3; AltName: Full=High-affinity urea active transporter DUR3 && PF00474:Sodium:solute symporter family |
| A00584 | Contig11 | 602225 | 603203 | + | 864 |  | 80.34 | 97.48 | 93.78 | 26.40 | 25.89 | 26.31 | 1.79 | 0.000 | - && Q09686.1 RecName: Full=Putative glutamine amidotransferase-like protein C13C5.04 && PF00117:Glutamine amidotransferase class-I |
| A00593 | Contig11 | 635111 | 636490 | + | 1380 |  | 3.86 | 2.47 | 3.38 | 2.03 | 1.70 | 0.95 | 1.05 | 0.012 | - && - && PF01728:FtsJ-like methyltransferase |
| A00595 | Contig11 | 639301 | 647480 | - | 1791 |  | 666.00 | 426.33 | 742.89 | 121.27 | 283.71 | 206.68 | 1.59 | 0.000 | - && - && - |
| A00600 | Contig11 | 664260 | 665435 | - | 1176 |  | 0.17 | 0.00 | 0.54 | 2.23 | 2.15 | 0.52 | -2.78 | 0.002 | - && - && PF01728:FtsJ-like methyltransferase |
| A00609 | Contig11 | 689905 | 690841 | + | 756 |  | 54.26 | 59.75 | 57.76 | 151.13 | 145.37 | 144.06 | -1.36 | 0.000 | - && - && - |
| A00613 | Contig11 | 712590 | 715074 | - | 1617 |  | 7.01 | 7.45 | 4.69 | 1.27 | 1.45 | 0.76 | 2.46 | 0.000 | - && P32071.1 RecName: Full=Cycloheximide resistance protein && PF07690:Major Facilitator Superfamily |
| A00626 | Contig11 | 791399 | 793056 | - | 1008 |  | 6.55 | 8.66 | 8.16 | 3.34 | 2.60 | 2.52 | 1.47 | 0.000 | - && - && - |
| A00632 | Contig11 | 813675 | 817058 | + | 2160 |  | 21.59 | 23.56 | 25.97 | 77.65 | 84.12 | 55.46 | -1.61 | 0.000 | - && Q9VA70.1 RecName: Full=Neutral ceramidase; Short=N-CDase; Short=NCDase; AltName: Full=Neutral N-acylsphingosine amidohydrolase; AltName: Full=Neutral acylsphingosine deacylase; AltName: Full=Slug-a-bed protein; Flags: Precursor && PF17048:Neutral/alkaline non-lysosomal ceramidase, C-terminal\|PF04734:Neutral/alkaline non-lysosomal ceramidase, N-terminal |
| A00644 | Contig11 | 854845 | 856810 | - | 1689 |  | 25.69 | 40.18 | 21.15 | 93.99 | 165.66 | 58.71 | -1.87 | 0.000 | - && Q01772.1 RecName: Full=Aldehyde oxidase GLOX; AltName: Full=Glyoxal oxidase; Short=GLOX; Flags: Precursor && PF09118:Domain of unknown function (DUF1929)\|PF07250:Glyoxal oxidase N-terminus |
| A00650 | Contig11 | 870313 | 876163 | + | 4182 |  | 3.94 | 3.89 | 2.51 | 39.55 | 36.62 | 43.21 | -3.53 | 0.000 | - && Q9FJR0.2 RecName: Full=Regulator of nonsense transcripts 1 homolog; AltName: Full=ATP-dependent helicase UPF1 && PF13087:AAA domain\|PF00026:Eukaryotic aspartyl protease\|PF13604:AAA domain |
| A00657 | Contig11 | 901979 | 902697 | + | 546 |  | 6.86 | 8.09 | 16.73 | 18.32 | 45.09 | 12.05 | -1.25 | 0.008 | - && - && PF07366:SnoaL-like polyketide cyclase |
| A00658 | Contig11 | 902848 | 903423 | - | 576 |  | 32.53 | 36.43 | 46.31 | 23.21 | 16.74 | 14.32 | 1.09 | 0.000 | - && D4GWH7.1 RecName: Full=Uncharacterized isochorismatase family protein HVO_2328 && PF00857:Isochorismatase family |
| A00660 | Contig11 | 906739 | 907782 | + | 720 |  | 2.19 | 1.25 | 1.40 | 5.32 | 7.93 | 0.98 | -1.56 | 0.018 | - && - && - |
| A00661 | Contig11 | 909580 | 910985 | - | 1071 |  | 22.00 | 29.34 | 17.06 | 4.36 | 3.67 | 4.75 | 2.42 | 0.000 | - && Q6ZXC1.2 RecName: Full=Probable inactive dehydrogenase EasA; AltName: Full=Ergot alkaloid biosynthesis protein A [Claviceps purpurea] && PF00724:NADH:flavin oxidoreductase / NADH oxidase family |
| A00662 | Contig11 | 915254 | 916400 | + | 900 |  | 70.78 | 72.95 | 45.07 | 15.27 | 11.86 | 13.16 | 2.23 | 0.000 | - && - && PF01040:UbiA prenyltransferase family |
| A00664 | Contig11 | 926969 | 928113 | - | 924 |  | 7.58 | 8.58 | 5.54 | 2.33 | 3.65 | 3.42 | 1.21 | 0.001 | - && - && PF01040:UbiA prenyltransferase family |
| A00679 | Contig11 | 990550 | 992655 | - | 1302 |  | 130.33 | 150.04 | 132.46 | 15.37 | 11.22 | 81.42 | 1.93 | 0.019 | - && O34714.1 RecName: Full=Oxalate decarboxylase OxdC && PF00190:Cupin |
| A00681 | Contig11 | 996288 | 998891 | - | 2016 |  | 7.92 | 6.87 | 8.93 | 66.83 | 24.42 | 83.84 | -2.88 | 0.000 | - && Q7SFB0.1 RecName: Full=Beta-glucuronidase; Short=GlcAase; AltName: Full=Beta-D-glucuronoside glucuronosohydrolase; Flags: Precursor && PF16862:Glycosyl hydrolase family 79 C-terminal beta domain |
| A00691 | Contig11 | 1038940 | 1040648 | - | 1008 |  | 55.66 | 40.33 | 63.71 | 18.37 | 17.27 | 14.97 | 1.66 | 0.000 | - && - && - |
| A00692 | Contig11 | 1041143 | 1043426 | + | 1743 |  | 117.72 | 78.44 | 166.71 | 29.66 | 30.77 | 24.47 | 2.10 | 0.000 | - && Q0R4L2.1 RecName: Full=Pyranose dehydrogenase 3; Short=PDH 3; AltName: Full=Pyranose:quinone oxidoreductase 3; Flags: Precursor && PF00732:GMC oxidoreductase\|PF05199:GMC oxidoreductase |
| A00694 | Contig11 | 1045318 | 1045807 | + | 342 |  | 16.72 | 8.22 | 20.30 | 41.28 | 58.30 | 26.94 | -1.48 | 0.000 | - && - && PF03795:YCII-related domain |
| A00699 | Contig11 | 1065012 | 1067449 | - | 1089 |  | 50.61 | 27.29 | 45.05 | 3.35 | 6.53 | 1.37 | 3.45 | 0.000 | - && O59726.1 RecName: Full=Vacuolar membrane amino acid uptake transporter fnx2 && PF07690:Major Facilitator Superfamily |
| A00704 | Contig11 | 1079413 | 1083546 | + | 3264 |  | 6.19 | 5.63 | 5.26 | 34.55 | 12.48 | 50.84 | -2.52 | 0.000 | - && Q01896.1 RecName: Full=Sodium transport ATPase 2 && PF00702:haloacid dehalogenase-like hydrolase\|PF00122:E1-E2 ATPase\|PF00690:Cation transporter/ATPase, N-terminus\|PF00689:Cation transporting ATPase, C-terminus |
| A00706 | Contig12 | 35168 | 36257 | + | 768 |  | 4.75 | 5.10 | 3.33 | 23.01 | 14.50 | 22.17 | -2.18 | 0.000 | - && - && PF04616:Glycosyl hydrolases family 43 |
| A00709 | Contig12 | 398353 | 400619 | + | 1533 |  | 10.48 | 12.64 | 9.42 | 2.26 | 1.89 | 1.66 | 2.49 | 0.000 | - && Q99056.2 RecName: Full=Laccase-5; AltName: Full=Benzenediol:oxygen oxidoreductase 5; AltName: Full=Diphenol oxidase 5; AltName: Full=Urishiol oxidase 5; Flags: Precursor && PF00394:Multicopper oxidase\|PF07731:Multicopper oxidase\|PF07732:Multicopper oxidase |
| A00710 | Contig12 | 403464 | 407227 | + | 2448 |  | 28.80 | 31.45 | 17.09 | 8.71 | 7.92 | 7.06 | 1.71 | 0.000 | - && Q99044.1 RecName: Full=Laccase-1; AltName: Full=Benzenediol:oxygen oxidoreductase 1; AltName: Full=Diphenol oxidase 1; AltName: Full=Urishiol oxidase 1; Flags: Precursor && PF07732:Multicopper oxidase\|PF07731:Multicopper oxidase\|PF00394:Multicopper oxidase |
| A00713 | Contig12 | 415549 | 417306 | - | 954 |  | 1.65 | 2.32 | 0.67 | 15.09 | 14.13 | 36.15 | -3.82 | 0.000 | - && O14405.1 RecName: Full=Endoglucanase-4; AltName: Full=Cellulase IV; AltName: Full=Cellulase-61A; Short=Cel61A; AltName: Full=Endo-1,4-beta-glucanase IV; Short=EGIV; AltName: Full=Endoglucanase IV; AltName: Full=Endoglucanase-61A; Flags: Precursor && PF00734:Fungal cellulose binding domain\|PF03443:Glycosyl hydrolase family 61 |
| A00716 | Contig12 | 425111 | 427074 | - | 1674 |  | 0.82 | 0.96 | 0.71 | 0.22 | 0.17 | 0.00 | 2.67 | 0.002 | - && Q01772.1 RecName: Full=Aldehyde oxidase GLOX; AltName: Full=Glyoxal oxidase; Short=GLOX; Flags: Precursor && PF09118:Domain of unknown function (DUF1929)\|PF07250:Glyoxal oxidase N-terminus |
| A00740 | Contig12 | 504833 | 506944 | + | 1548 |  | 8.28 | 8.17 | 5.31 | 4.29 | 3.57 | 2.49 | 1.07 | 0.001 | - && B8NHY4.1 RecName: Full=O-methylsterigmatocystin oxidoreductase; Short=OMST oxidoreductase; AltName: Full=Aflatoxin B synthase; AltName: Full=Aflatoxin biosynthesis protein Q; AltName: Full=Cytochrome P450 64 >P0CT93.1 RecName: Full=O-methylsterigmatocystin oxidoreductase; Short=OMST oxidoreductase; AltName: Full=Aflatoxin B synthase; AltName: Full=Aflatoxin biosynthesis protein Q; AltName: Full=Cytochrome P450 64 && PF00067:Cytochrome P450 |
| A00741 | Contig12 | 508139 | 509287 | + | 1149 |  | 0.51 | 0.52 | 0.56 | 4.07 | 4.24 | 4.35 | -2.99 | 0.000 | - && - && PF03732:Retrotransposon gag protein |
| A00742 | Contig12 | 509365 | 513273 | + | 3909 |  | 0.13 | 0.13 | 0.05 | 0.29 | 0.29 | 0.31 | -1.56 | 0.040 | - && P0CT34.1 RecName: Full=Transposon Tf2-1 polyprotein; AltName: Full=Retrotransposable element Tf2 155 kDa protein >P0CT35.1 RecName: Full=Transposon Tf2-2 polyprotein; AltName: Full=Retrotransposable element Tf2 155 kDa protein >P0CT37.1 RecName: Full=Transposon Tf2-4 polyprotein; AltName: Full=Retrotransposable element Tf2 155 kDa protein >P0CT40.1 RecName: Full=Transposon Tf2-9 polyprotein; AltName: Full=Retrotransposable element Tf2 155 kDa protein && PF00665:Integrase core domain\|PF00078:Reverse transcriptase (RNA-dependent DNA polymerase) |
| A00755 | Contig12 | 544244 | 546411 | + | 1803 |  | 2.13 | 2.00 | 2.33 | 11.46 | 3.89 | 26.58 | -2.70 | 0.000 | - && A1C4M2.1 RecName: Full=Extracellular metalloproteinase mep; AltName: Full=Elastinolytic metalloproteinase mep; AltName: Full=Fungalysin mep; Flags: Precursor && PF02128:Fungalysin metallopeptidase (M36)\|PF07504:Fungalysin/Thermolysin Propeptide Motif |
| A00774 | Contig12 | 605133 | 606291 | + | 894 |  | 6.18 | 6.74 | 2.04 | 1.78 | 1.05 | 1.67 | 1.73 | 0.001 | - && - && - |
| A00779 | Contig12 | 626778 | 631875 | - | 2226 |  | 29.59 | 37.52 | 16.91 | 4.20 | 4.25 | 10.21 | 2.17 | 0.000 | - && Q01752.1 RecName: Full=Aryl-alcohol dehydrogenase [NADP(+)]; Short=AAD && PF00248:Aldo/keto reductase family |
| A00789 | Contig12 | 666401 | 667049 | - | 378 |  | 512.06 | 325.33 | 588.70 | 37.10 | 16.59 | 48.29 | 3.81 | 0.000 | - && - && PF16850:Peptidase inhibitor I66 |
| A00795 | Contig12 | 689993 | 690633 | - | 462 |  | 9.82 | 3.48 | 18.78 | 1.21 | 5.27 | 2.85 | 1.78 | 0.004 | - && - && PF16850:Peptidase inhibitor I66 |
| A00797 | Contig12 | 695656 | 696126 | + | 471 |  | 28.05 | 36.87 | 32.20 | 75.43 | 166.35 | 63.54 | -1.65 | 0.000 | - && - && - |
| A00799 | Contig12 | 726716 | 728501 | + | 1731 |  | 48.42 | 52.31 | 66.07 | 107.76 | 148.72 | 108.19 | -1.13 | 0.000 | - && - && - |
| A00801 | Contig12 | 737038 | 737679 | + | 465 |  | 3.60 | 9.07 | 15.52 | 3.82 | 3.42 | 2.26 | 1.57 | 0.003 | - && - && PF16850:Peptidase inhibitor I66 |
| A00811 | Contig12 | 786103 | 786712 | + | 435 |  | 7.03 | 10.85 | 9.03 | 20.85 | 24.53 | 29.25 | -1.47 | 0.000 | - && - && PF16850:Peptidase inhibitor I66 |
| A00814 | Contig12 | 790765 | 793213 | + | 1626 |  | 263.24 | 229.05 | 253.60 | 26.45 | 43.47 | 25.96 | 2.96 | 0.000 | - && Q7Z9I0.2 RecName: Full=Uncharacterized MFS-type transporter SPBC409.08 && PF07690:Major Facilitator Superfamily |
| A00827 | Contig12 | 832046 | 832977 | - | 558 |  | 633.14 | 1017.20 | 92.99 | 13.24 | 10.40 | 6.92 | 5.83 | 0.000 | - && - && - |
| A00842 | Contig12 | 883771 | 884703 | - | 486 |  | 472.32 | 402.59 | 519.72 | 61.18 | 35.25 | 15.17 | 3.64 | 0.000 | - && - && - |
| A00857 | Contig12 | 925611 | 926657 | - | 753 |  | 51.46 | 40.26 | 49.98 | 9.06 | 15.04 | 5.94 | 2.24 | 0.000 | - && - && PF01738:Dienelactone hydrolase family |
| A00858 | Contig12 | 927181 | 927869 | - | 573 |  | 104.11 | 141.38 | 135.51 | 7.67 | 5.88 | 9.65 | 4.04 | 0.000 | - && - && PF01738:Dienelactone hydrolase family |
| A00870 | Contig12 | 968248 | 971232 | + | 1926 |  | 6.55 | 7.77 | 6.21 | 25.97 | 32.18 | 22.51 | -1.97 | 0.000 | - && - && PF07944:Beta-L-arabinofuranosidase, GH127 |
| A00882 | Contig12 | 1009109 | 1010649 | + | 1308 |  | 1.96 | 2.15 | 3.00 | 19.01 | 17.75 | 5.90 | -2.58 | 0.000 | - && - && - |
| A00914 | Contig12 | 151091 | 159670 | + | 2760 |  | 5.57 | 11.89 | 7.81 | 2.41 | 1.83 | 2.10 | 2.00 | 0.000 | - && - && - |
| A00923 | Contig12 | 187492 | 192583 | + | 2955 |  | 11.21 | 10.91 | 7.20 | 3.54 | 4.18 | 3.12 | 1.44 | 0.000 | - && A1CFL6.2 RecName: Full=Cytochrome P450 monooxygenase patI; AltName: Full=Patulin synthesis protein I; AltName: Full=m-hydroxybenzyl alcohol hydroxylase; Flags: Precursor && PF09994:Uncharacterized alpha/beta hydrolase domain (DUF2235)\|PF00067:Cytochrome P450 |
| A00940 | Contig12 | 18711 | 19618 | + | 627 |  | 8.96 | 8.49 | 6.99 | 5.37 | 3.73 | 2.38 | 1.09 | 0.006 | - && - && - |
| A00942 | Contig12 | 266385 | 268527 | + | 1656 |  | 19.65 | 19.10 | 17.87 | 6.10 | 5.20 | 3.60 | 1.93 | 0.000 | - && Q9C512.1 RecName: Full=Mannosyl-oligosaccharide 1,2-alpha-mannosidase MNS1; Short=AtMANIb; AltName: Full=Alpha-mannosidase IB && PF01532:Glycosyl hydrolase family 47 |
| A00951 | Contig12 | 20002 | 23175 | - | 3102 |  | 34.17 | 25.18 | 36.13 | 7.81 | 11.08 | 5.69 | 1.96 | 0.000 | - && - && - |
| A00964 | Contig12 | 340716 | 341448 | + | 501 |  | 124.58 | 91.97 | 102.11 | 47.59 | 32.32 | 37.84 | 1.44 | 0.000 | - && - && - |
| A00979 | Contig12 | 374917 | 377494 | + | 1650 |  | 2.87 | 2.56 | 3.38 | 10.09 | 13.67 | 5.11 | -1.71 | 0.000 | - && Q9SE50.2 RecName: Full=Beta-D-glucopyranosyl abscisate beta-glucosidase; AltName: Full=Beta-glucosidase 1; Short=AtBG1; AltName: Full=Beta-glucosidase 18; Short=AtBGLU18; AltName: Full=Beta-glucosidase homolog 1; Flags: Precursor && PF00232:Glycosyl hydrolase family 1 |
| A00980 | Contig12 | 378065 | 380611 | + | 1728 |  | 15.69 | 11.91 | 17.13 | 47.56 | 69.56 | 46.47 | -1.87 | 0.000 | - && Q9C525.1 RecName: Full=Beta-glucosidase 21; Short=AtBGLU21; AltName: Full=Protein PHOSPHATE STARVATION-RESPONSE 3.2; Flags: Precursor && PF00232:Glycosyl hydrolase family 1 |
| A00981 | Contig12 | 381102 | 382688 | - | 1188 |  | 4.07 | 4.56 | 2.92 | 11.18 | 15.52 | 9.60 | -1.65 | 0.000 | - && - && - |
| A00982 | Contig12 | 383504 | 384904 | - | 999 |  | 2.76 | 5.02 | 4.39 | 9.73 | 14.62 | 12.65 | -1.60 | 0.000 | - && - && - |
| A00998 | Contig13 | 65327 | 67789 | - | 1251 |  | 12.30 | 12.20 | 13.65 | 24.59 | 19.61 | 32.83 | -1.01 | 0.000 | - && P37967.2 RecName: Full=Para-nitrobenzyl esterase; AltName: Full=Intracellular esterase B; AltName: Full=PNB carboxy-esterase; Short=PNBCE && PF00135:Carboxylesterase family |
| A00999 | Contig13 | 72443 | 75431 | + | 2724 |  | 155.90 | 140.15 | 185.72 | 397.05 | 205.26 | 444.01 | -1.12 | 0.000 | - && P0CS63.1 RecName: Full=pH-response transcription factor pacC/RIM101 >P0CS62.1 RecName: Full=pH-response transcription factor pacC/RIM101 && - |
| A01007 | Contig13 | 105990 | 109685 | - | 3141 |  | 169.55 | 106.27 | 137.36 | 28.78 | 64.34 | 15.53 | 1.93 | 0.002 | - && - && - |
| A01013 | Contig13 | 127755 | 131915 | + | 2817 |  | 1.37 | 1.28 | 1.56 | 12.91 | 12.86 | 7.88 | -3.00 | 0.000 | - && P79066.1 RecName: Full=Lipase 1; AltName: Full=Lipase I; AltName: Full=TFL I; Flags: Precursor && PF00135:Carboxylesterase family |
| A01014 | Contig13 | 132249 | 134894 | - | 2181 |  | 10.76 | 12.66 | 11.43 | 32.49 | 20.52 | 18.67 | -1.04 | 0.000 | - && P18773.2 RecName: Full=Esterase && PF07859:alpha/beta hydrolase fold |
| A01017 | Contig13 | 139819 | 140259 | - | 441 |  | 5.37 | 8.65 | 5.59 | 1.48 | 0.85 | 0.80 | 2.65 | 0.000 | - && - && - |
| A01018 | Contig13 | 140723 | 146768 | - | 3609 |  | 102.13 | 88.04 | 103.27 | 32.46 | 27.21 | 23.25 | 1.82 | 0.000 | - && Q9P7U2.1 RecName: Full=Putative aryl-alcohol dehydrogenase C977.14c && PF09994:Uncharacterized alpha/beta hydrolase domain (DUF2235)\|PF00248:Aldo/keto reductase family |
| A01023 | Contig13 | 163047 | 163898 | - | 678 |  | 2.62 | 11.10 | 3.50 | 3.17 | 2.35 | 2.33 | 1.13 | 0.041 | - && - && - |
| A01055 | Contig13 | 282097 | 284344 | + | 1665 |  | 180.92 | 155.56 | 185.11 | 34.82 | 62.35 | 51.81 | 1.81 | 0.000 | - && Q6BZP5.1 RecName: Full=2-methylisocitrate lyase, mitochondrial; Flags: Precursor && PF00463:Isocitrate lyase family |
| A01068 | Contig13 | 322605 | 327920 | + | 4488 |  | 267.69 | 287.61 | 235.27 | 63.06 | 48.12 | 68.97 | 2.13 | 0.000 | - && P41820.1 RecName: Full=Brefeldin A resistance protein && PF01061:ABC-2 type transporter\|PF06422:CDR ABC transporter\|PF00005:ABC transporter |
| A01088 | Contig13 | 398990 | 399452 | + | 351 |  | 64.33 | 71.50 | 43.46 | 140.64 | 325.10 | 280.03 | -2.06 | 0.000 | - && P04158.2 RecName: Full=Fruiting body protein SC1; AltName: Full=Hydrophobin SC1; Flags: Precursor && PF01185:Fungal hydrophobin |
| A01089 | Contig13 | 400640 | 401104 | + | 351 |  | 88.21 | 134.99 | 108.79 | 4173.48 | 4826.14 | 2830.53 | -5.16 | 0.000 | - && P04158.2 RecName: Full=Fruiting body protein SC1; AltName: Full=Hydrophobin SC1; Flags: Precursor && PF01185:Fungal hydrophobin |
| A01090 | Contig13 | 402450 | 402918 | + | 351 |  | 0.84 | 5.43 | 2.86 | 86.30 | 188.55 | 59.51 | -5.19 | 0.000 | - && P16934.1 RecName: Full=Fruiting body protein SC4; AltName: Full=Hydrophobin SC4; Flags: Precursor && PF01185:Fungal hydrophobin |
| A01091 | Contig13 | 404330 | 404805 | + | 354 |  | 28.41 | 28.93 | 19.10 | 116.21 | 151.79 | 59.25 | -2.10 | 0.000 | - && P04158.2 RecName: Full=Fruiting body protein SC1; AltName: Full=Hydrophobin SC1; Flags: Precursor && PF01185:Fungal hydrophobin |
| A01111 | Contig13 | 500813 | 502570 | + | 1479 |  | 5.80 | 4.82 | 5.31 | 2.47 | 2.91 | 1.90 | 1.13 | 0.000 | - && - && - |
| A01114 | Contig13 | 507224 | 509092 | - | 1794 |  | 0.33 | 0.73 | 1.22 | 2.19 | 2.24 | 2.15 | -1.53 | 0.002 | - && - && - |
| A01116 | Contig13 | 513413 | 513914 | + | 324 |  | 573.66 | 822.01 | 310.42 | 132.16 | 67.61 | 89.93 | 2.56 | 0.000 | - && - && - |
| A01118 | Contig13 | 518069 | 522482 | - | 4176 |  | 18.13 | 16.20 | 22.38 | 51.90 | 55.68 | 39.78 | -1.38 | 0.000 | - && - && PF00621:RhoGEF domain |
| A01121 | Contig13 | 536571 | 538043 | + | 1473 |  | 5.82 | 5.72 | 7.13 | 2.22 | 2.35 | 1.73 | 1.57 | 0.000 | - && Q6NT99.1 RecName: Full=Dual specificity protein phosphatase 23; AltName: Full=Low molecular mass dual specificity phosphatase 3; Short=LDP-3 && - |
| A01124 | Contig13 | 543221 | 546980 | - | 1986 |  | 248.00 | 236.36 | 210.99 | 88.08 | 71.13 | 89.22 | 1.48 | 0.000 | - && - && - |
| A01137 | Contig13 | 602131 | 604160 | + | 1485 |  | 8.23 | 8.45 | 5.04 | 1.32 | 2.52 | 0.77 | 2.24 | 0.000 | - && Q701P2.1 RecName: Full=Ent-kaurene oxidase; AltName: Full=Cytochrome P450 503A1; AltName: Full=Cytochrome P450-4 [Fusarium proliferatum] && PF00067:Cytochrome P450 |
| A01145 | Contig13 | 636862 | 639344 | + | 2319 |  | 89.04 | 85.67 | 95.96 | 56.77 | 32.86 | 40.34 | 1.06 | 0.000 | - && - && - |
| A01156 | Contig13 | 672032 | 673864 | - | 933 |  | 37.62 | 55.09 | 37.40 | 140.39 | 57.59 | 83.24 | -1.11 | 0.000 | - && - && - |
| A01160 | Contig13 | 693179 | 694473 | + | 1044 |  | 13.98 | 11.15 | 18.20 | 35.20 | 27.71 | 34.80 | -1.17 | 0.000 | - && P81054.2 RecName: Full=Peptidyl-Lys metalloendopeptidase; Short=MEP; AltName: Full=GfMEP; Flags: Precursor && PF14521:Lysine-specific metallo-endopeptidase |
| A01165 | Contig13 | 705923 | 711220 | + | 2424 |  | 19.40 | 21.20 | 18.35 | 166.36 | 104.54 | 219.47 | -3.06 | 0.000 | - && Q99385.1 RecName: Full=Vacuolar calcium ion transporter; AltName: Full=High copy number undoes manganese protein 1; AltName: Full=Manganese resistance 1 protein; AltName: Full=Vacuolar Ca(2+)/H(+) exchanger && PF01699:Sodium/calcium exchanger protein\|PF01494:FAD binding domain |
| A01169 | Contig13 | 722905 | 724807 | + | 1719 |  | 0.92 | 1.40 | 1.17 | 1.85 | 3.65 | 1.99 | -1.10 | 0.012 | - && - && - |
| A01170 | Contig13 | 728854 | 729910 | + | 900 |  | 16.43 | 18.52 | 14.01 | 43.01 | 51.69 | 34.81 | -1.40 | 0.000 | - && Q8RWL6.1 RecName: Full=Serine/threonine-protein kinase STY17; AltName: Full=Serine/threonine/tyrosine-protein kinase 17 && PF00069:Protein kinase domain |
| A01181 | Contig13 | 761084 | 764008 | - | 2697 |  | 2.16 | 2.20 | 1.86 | 0.83 | 0.69 | 0.72 | 1.47 | 0.000 | - && - && - |
| A01194 | Contig13 | 833942 | 835121 | - | 1119 |  | 14.01 | 14.17 | 13.55 | 5.43 | 3.93 | 6.51 | 1.39 | 0.000 | - && - && PF01636:Phosphotransferase enzyme family |
| A01207 | Contig13 | 887895 | 891035 | - | 2166 |  | 8.97 | 11.86 | 7.21 | 5.01 | 2.46 | 3.89 | 1.30 | 0.000 | - && Q54QI2.1 RecName: Full=Serine/threonine-protein kinase DDB_G0283821 && PF00069:Protein kinase domain |
| A01210 | Contig13 | 909738 | 911709 | - | 1551 |  | 0.06 | 0.32 | 0.06 | 1.33 | 0.42 | 0.17 | -2.10 | 0.048 | - && - && - |
| A01218 | Contig13 | 940781 | 942074 | - | 1008 |  | 427.77 | 440.69 | 328.15 | 188.85 | 162.52 | 153.58 | 1.24 | 0.000 | - && Q9IA79.1 RecName: Full=Probable Bax inhibitor 1; Short=BI-1; AltName: Full=Testis-enhanced gene transcript protein homolog; AltName: Full=Transmembrane BAX inhibitor motif-containing protein 6 && PF01027:Inhibitor of apoptosis-promoting Bax1 |
| A01223 | Contig13 | 953370 | 955202 | - | 1353 |  | 112.96 | 128.14 | 100.33 | 27.78 | 22.62 | 42.87 | 1.87 | 0.000 | - && - && - |
| A01224 | Contig13 | 956774 | 959083 | + | 1551 |  | 4.51 | 3.88 | 1.65 | 14.47 | 13.16 | 11.09 | -1.95 | 0.000 | - && P18631.1 RecName: Full=Low-affinity glucose transporter; AltName: Full=Hexose transporter 1 && PF00083:Sugar (and other) transporter |
| A01230 | Contig13 | 977853 | 979864 | - | 1572 |  | 6.71 | 3.51 | 6.10 | 3.39 | 1.25 | 1.28 | 1.46 | 0.001 | - && O00061.1 RecName: Full=Cytochrome P450 67; AltName: Full=CYPLXVII; AltName: Full=Planta-induced rust protein 16, partial [Uromyces viciae-fabae] && PF00067:Cytochrome P450 |
| A01232 | Contig13 | 983617 | 987173 | - | 903 |  | 1.64 | 1.67 | 1.01 | 0.72 | 0.31 | 0.29 | 1.70 | 0.023 | - && - && - |
| A01240 | Contig13 | 1013822 | 1016121 | - | 2043 |  | 90.74 | 62.16 | 127.70 | 45.21 | 49.99 | 25.60 | 1.22 | 0.000 | - && Q6MG06.1 RecName: Full=Guanine nucleotide-binding protein-like 1 && PF01926:50S ribosome-binding GTPase |
| A01243 | Contig13 | 1023565 | 1027596 | - | 2469 |  | 3.59 | 4.39 | 5.92 | 2.04 | 1.71 | 1.95 | 1.29 | 0.000 | - && P0CG48.3 RecName: Full=Polyubiquitin-C; Contains: RecName: Full=Ubiquitin; Flags: Precursor && PF00240:Ubiquitin family |
| A01247 | Contig14 | 2350 | 4393 | + | 1701 |  | 89.62 | 120.40 | 115.57 | 498.09 | 1156.62 | 744.74 | -2.88 | 0.000 | - && O43112.2 RecName: Full=Phosphoenolpyruvate carboxykinase [ATP] && PF01293:Phosphoenolpyruvate carboxykinase |
| A01252 | Contig14 | 23348 | 24211 | - | 702 |  | 2.53 | 0.86 | 2.21 | 6.53 | 2.93 | 4.13 | -1.28 | 0.017 | - && - && - |
| A01256 | Contig14 | 35157 | 36872 | + | 1377 |  | 12.89 | 12.83 | 6.83 | 3.94 | 2.79 | 3.89 | 1.62 | 0.000 | - && Q54H55.1 RecName: Full=FAD-linked oxidoreductase DDB_G0289697 && PF08031:Berberine and berberine like\|PF01565:FAD binding domain |
| A01286 | Contig14 | 158669 | 163358 | - | 2796 |  | 85.87 | 90.59 | 101.38 | 285.04 | 234.49 | 256.59 | -1.48 | 0.000 | - && P0CM93.1 RecName: Full=Protein PNS1 >P0CM92.1 RecName: Full=Protein PNS1 && PF02020:eIF4-gamma/eIF5/eIF2-epsilon\|PF04515:Plasma-membrane choline transporter |
| A01290 | Contig14 | 173247 | 177548 | + | 3753 |  | 12.56 | 16.29 | 12.88 | 25.76 | 23.75 | 37.86 | -1.07 | 0.000 | - && Q12754.1 RecName: Full=Ribosomal RNA-processing protein 12 && PF08161:NUC173 domain |
| A01293 | Contig14 | 193312 | 195320 | + | 1341 |  | 4.12 | 4.04 | 3.47 | 10.04 | 10.61 | 11.78 | -1.48 | 0.000 | - && A1C8U0.1 RecName: Full=Mannan endo-1,4-beta-mannosidase F; AltName: Full=Endo-beta-1,4-mannanase F; Flags: Precursor && PF00150:Cellulase (glycosyl hydrolase family 5)\|PF00734:Fungal cellulose binding domain |
| A01294 | Contig14 | 196076 | 197301 | + | 777 |  | 104.31 | 133.33 | 71.60 | 25.99 | 20.12 | 15.81 | 2.32 | 0.000 | - && Q48436.2 RecName: Full=Diacetyl reductase [(S)-acetoin forming]; AltName: Full=Acetoin(diacetyl) reductase; Short=AR; AltName: Full=Meso-2,3-butanediol dehydrogenase && PF00106:short chain dehydrogenase |
| A01296 | Contig14 | 201027 | 202266 | - | 774 |  | 7.26 | 4.93 | 6.85 | 15.70 | 16.45 | 22.79 | -1.53 | 0.000 | - && - && - |
| A01306 | Contig14 | 239570 | 240689 | - | 963 |  | 16.49 | 16.68 | 13.66 | 28.16 | 47.44 | 56.14 | -1.49 | 0.000 | - && Q3JRV4.1 RecName: Full=Probable metallo-hydrolase BURPS1710b_2304 && PF00753:Metallo-beta-lactamase superfamily |
| A01312 | Contig14 | 253019 | 253962 | + | 819 |  | 14.69 | 25.86 | 7.14 | 8.79 | 6.97 | 7.61 | 1.03 | 0.010 | - && - && - |
| A01313 | Contig14 | 254911 | 256583 | - | 1371 |  | 343.92 | 317.57 | 176.64 | 60.90 | 52.03 | 117.72 | 1.86 | 0.000 | - && - && - |
| A01315 | Contig14 | 260264 | 261691 | + | 1029 |  | 5.56 | 5.46 | 2.66 | 11.27 | 11.10 | 9.72 | -1.23 | 0.000 | - && Q9HEZ1.1 RecName: Full=Endo-1,4-beta-xylanase A; Short=Xylanase A; AltName: Full=1,4-beta-D-xylan xylanohydrolase A; Flags: Precursor && PF00331:Glycosyl hydrolase family 10 |
| A01317 | Contig14 | 270727 | 273266 | + | 1611 |  | 14.63 | 15.58 | 15.08 | 6.50 | 5.29 | 7.30 | 1.25 | 0.000 | - && P40386.1 RecName: Full=Probable thiamine biosynthetic bifunctional enzyme; Includes: RecName: Full=Thiamine-phosphate synthase; Short=TP synthase; Short=TPS; AltName: Full=Thiamine-phosphate pyrophosphorylase; Short=TMP pyrophosphorylase; Short=TMP-PPase; Includes: RecName: Full=Hydroxyethylthiazole kinase; AltName: Full=4-methyl-5-beta-hydroxyethylthiazole kinase; Short=TH kinase; Short=THZ kinase && PF02581:Thiamine monophosphate synthase/TENI\|PF02110:Hydroxyethylthiazole kinase family |
| A01332 | Contig14 | 314973 | 319075 | - | 3306 |  | 29.44 | 35.04 | 51.70 | 82.44 | 95.11 | 71.57 | -1.10 | 0.000 | - && Q04336.1 RecName: Full=Uncharacterized protein YMR196W && PF03200:Glycosyl hydrolase family 63 C-terminal domain |
| A01336 | Contig14 | 325814 | 326985 | - | 1071 |  | 51.37 | 48.46 | 69.77 | 258.66 | 321.56 | 209.85 | -2.22 | 0.000 | - && - && - |
| A01376 | Contig14 | 530632 | 533773 | - | 2907 |  | 11.43 | 12.85 | 10.94 | 23.51 | 20.35 | 26.69 | -1.00 | 0.000 | - && Q4P3W3.1 RecName: Full=ATP-dependent RNA helicase DBP10 && PF08147:DBP10CT (NUC160) domain\|PF00271:Helicase conserved C-terminal domain\|PF00270:DEAD/DEAH box helicase |
| A01385 | Contig14 | 570644 | 573682 | - | 1128 |  | 661.64 | 740.81 | 679.45 | 383.18 | 258.92 | 318.36 | 1.12 | 0.000 | - && - && - |
| A01389 | Contig14 | 585524 | 587570 | + | 1263 |  | 32.63 | 29.49 | 31.25 | 87.28 | 72.56 | 64.06 | -1.26 | 0.000 | - && - && PF04145:Ctr copper transporter family |
| A01401 | Contig14 | 631263 | 632375 | + | 819 |  | 244.40 | 347.74 | 332.38 | 960.19 | 937.36 | 753.29 | -1.52 | 0.000 | - && - && - |
| A01404 | Contig14 | 644671 | 646676 | + | 1341 |  | 4.71 | 2.84 | 3.61 | 22.59 | 89.14 | 5.96 | -3.40 | 0.001 | - && P0CT90.1 RecName: Full=3-O-methyltransferase 2; Short=Mtrase 2 && PF00891:O-methyltransferase |
| A01410 | Contig14 | 662605 | 666113 | + | 2307 |  | 6.62 | 6.61 | 7.84 | 256.05 | 278.44 | 286.14 | -5.28 | 0.000 | - && D4B0V1.1 RecName: Full=Probable glucan endo-1,3-beta-glucosidase ARB_02077; AltName: Full=(1->3)-beta-glucan endohydrolase ARB_02077; Short=(1->3)-beta-glucanase ARB_02077; Flags: Precursor && PF12708:Pectate lyase superfamily protein |
| A01416 | Contig14 | 690919 | 697693 | + | 2058 |  | 136.12 | 92.49 | 181.46 | 57.61 | 79.46 | 56.20 | 1.09 | 0.000 | - && O14283.2 RecName: Full=Transcription factor prr1; AltName: Full=Pombe response regulator 1 && PF00072:Response regulator receiver domain\|PF03595:Voltage-dependent anion channel |
| A01424 | Contig14 | 736380 | 737048 | + | 669 |  | 74.28 | 79.38 | 55.57 | 15.79 | 11.05 | 28.33 | 1.92 | 0.000 | - && - && PF13460:NAD(P)H-binding |
| A01434 | Contig14 | 769710 | 771450 | + | 1035 |  | 9.91 | 7.86 | 11.30 | 2.44 | 1.36 | 0.68 | 2.70 | 0.000 | - && Q9P7U2.1 RecName: Full=Putative aryl-alcohol dehydrogenase C977.14c && PF00248:Aldo/keto reductase family |
| A01438 | Contig14 | 780111 | 781205 | - | 918 |  | 1.61 | 3.50 | 2.19 | 0.81 | 0.61 | 1.72 | 1.21 | 0.040 | - && O74628.1 RecName: Full=Uncharacterized oxidoreductase C162.03 && PF00106:short chain dehydrogenase |
| A01439 | Contig14 | 781789 | 782897 | - | 924 |  | 20.17 | 20.75 | 14.43 | 9.11 | 3.95 | 3.42 | 1.75 | 0.000 | - && O74628.1 RecName: Full=Uncharacterized oxidoreductase C162.03 && PF00106:short chain dehydrogenase |
| A01441 | Contig14 | 788913 | 789773 | + | 696 |  | 20.54 | 21.49 | 27.43 | 79.26 | 114.86 | 92.55 | -2.05 | 0.000 | - && - && - |
| A01466 | Contig14 | 888462 | 889092 | - | 570 |  | 28.02 | 38.57 | 31.89 | 118.43 | 153.55 | 418.62 | -2.81 | 0.000 | - && - && - |
| A01467 | Contig14 | 890667 | 891294 | + | 567 |  | 27.13 | 35.06 | 42.53 | 392.95 | 673.76 | 1673.29 | -4.71 | 0.000 | - && - && PF01338:Bacillus thuringiensis toxin |
| A01475 | Contig14 | 920849 | 921728 | + | 681 |  | 50.24 | 43.63 | 64.39 | 144.43 | 257.60 | 92.66 | -1.64 | 0.000 | - && - && PF02627:Carboxymuconolactone decarboxylase family |
| A01479 | Contig14 | 930293 | 930810 | + | 462 |  | 26.04 | 28.47 | 28.87 | 110.29 | 90.98 | 74.84 | -1.73 | 0.000 | - && - && PF16850:Peptidase inhibitor I66 |
| A01482 | Contig14 | 944907 | 945329 | + | 300 |  | 165.32 | 111.43 | 114.19 | 7.17 | 7.18 | 9.36 | 4.04 | 0.000 | - && - && - |
| A01503 | Contig15 | 393968 | 395456 | - | 942 |  | 0.52 | 1.17 | 0.39 | 1.99 | 2.38 | 1.02 | -1.37 | 0.043 | - && - && - |
| A01504 | Contig15 | 398204 | 399014 | + | 591 |  | 103.78 | 121.79 | 61.52 | 18.51 | 36.91 | 40.09 | 1.59 | 0.000 | - && - && - |
| A01510 | Contig15 | 424705 | 430233 | - | 2295 |  | 4.04 | 3.72 | 2.71 | 6.64 | 17.70 | 12.31 | -1.81 | 0.000 | - && Q99046.1 RecName: Full=Laccase-2; AltName: Full=Benzenediol:oxygen oxidoreductase 2; AltName: Full=Diphenol oxidase 2; AltName: Full=Urishiol oxidase 2; Flags: Precursor && PF00394:Multicopper oxidase\|PF07731:Multicopper oxidase\|PF07732:Multicopper oxidase |
| A01511 | Contig15 | 432829 | 435205 | - | 1560 |  | 54.61 | 72.59 | 35.72 | 254.00 | 175.82 | 253.99 | -2.07 | 0.000 | - && Q9H845.1 RecName: Full=Acyl-CoA dehydrogenase family member 9, mitochondrial; Short=ACAD-9; Flags: Precursor && PF00441:Acyl-CoA dehydrogenase, C-terminal domain\|PF02770:Acyl-CoA dehydrogenase, middle domain\|PF02771:Acyl-CoA dehydrogenase, N-terminal domain\|PF00173:Cytochrome b5-like Heme/Steroid binding domain |
| A01513 | Contig15 | 435603 | 437539 | - | 1821 |  | 0.32 | 0.77 | 1.10 | 2.62 | 1.59 | 1.88 | -1.47 | 0.004 | - && - && - |
| A01521 | Contig15 | 465885 | 467525 | - | 1566 |  | 3.15 | 1.79 | 2.45 | 1.01 | 0.48 | 1.40 | 1.35 | 0.005 | - && - && - |
| A01522 | Contig15 | 468762 | 469501 | + | 687 |  | 2.01 | 3.07 | 1.86 | 0.27 | 0.14 | 0.38 | 3.13 | 0.000 | - && A8NE23.1 RecName: Full=Alpha-muurolene synthase; AltName: Full=Gamma-muurolene synthase; AltName: Full=Germacrene-A synthase && PF03936:Terpene synthase family, metal binding domain |
| A01531 | Contig15 | 507256 | 513641 | - | 4248 |  | 24.00 | 19.05 | 20.39 | 4.20 | 4.01 | 3.06 | 2.49 | 0.000 | - && Q5F364.1 RecName: Full=Multidrug resistance-associated protein 1; AltName: Full=ATP-binding cassette sub-family C member 1; AltName: Full=Leukotriene C(4) transporter; Short=LTC4 transporter && PF00005:ABC transporter\|PF00664:ABC transporter transmembrane region |
| A01532 | Contig15 | 515411 | 516610 | - | 1056 |  | 52.29 | 56.85 | 31.57 | 11.24 | 10.19 | 17.37 | 1.86 | 0.000 | - && A8NE23.1 RecName: Full=Alpha-muurolene synthase; AltName: Full=Gamma-muurolene synthase; AltName: Full=Germacrene-A synthase && PF03936:Terpene synthase family, metal binding domain |
| A01539 | Contig15 | 534842 | 537567 | + | 2304 |  | 1.93 | 1.44 | 1.59 | 0.32 | 1.02 | 0.95 | 1.11 | 0.017 | - && P32644.1 RecName: Full=Putative ATP-dependent RNA helicase ECM32; AltName: Full=DNA helicase B; Short=Hcs B; AltName: Full=DNA helicase III; AltName: Full=Extracellular mutant protein 32; AltName: Full=Helicase 1; Short=scHelI; AltName: Full=Modulator of translation termination protein 1 && PF13087:AAA domain\|PF13604:AAA domain |
| A01540 | Contig15 | 537723 | 540511 | + | 2406 |  | 21.80 | 24.12 | 23.88 | 10.38 | 6.89 | 9.85 | 1.36 | 0.000 | - && O76512.1 RecName: Full=Regulator of nonsense transcripts 1; AltName: Full=ATP-dependent helicase smg-2; AltName: Full=Nonsense mRNA reducing factor 1; AltName: Full=Up-frameshift suppressor 1 homolog && PF13086:AAA domain\|PF13087:AAA domain |
| A01558 | Contig15 | 592589 | 595834 | + | 3246 |  | 5.01 | 5.81 | 7.09 | 16.30 | 16.35 | 16.71 | -1.46 | 0.000 | - && - && - |
| A01565 | Contig15 | 621422 | 627289 | + | 3414 |  | 5.00 | 4.62 | 4.47 | 44.86 | 65.37 | 30.62 | -3.32 | 0.000 | - && B8NPA4.1 RecName: Full=Probable feruloyl esterase B-1; AltName: Full=Ferulic acid esterase B-1; Short=FAEB-1; Flags: Precursor && PF07519:Tannase and feruloyl esterase\|PF00450:Serine carboxypeptidase |
| A01572 | Contig15 | 643583 | 644801 | - | 768 |  | 97.06 | 90.98 | 92.30 | 51.74 | 54.48 | 33.71 | 1.00 | 0.000 | - && - && PF13637:Ankyrin repeats (many copies)\|PF12796:Ankyrin repeats (3 copies) |
| A01580 | Contig15 | 664734 | 666530 | - | 1083 |  | 6.65 | 11.12 | 7.42 | 16.23 | 22.04 | 18.56 | -1.17 | 0.000 | - && P56404.1 RecName: Full=Aquaporin-8; Short=AQP-8 && PF00230:Major intrinsic protein |
| A01584 | Contig15 | 676335 | 680377 | + | 1110 |  | 72.58 | 65.93 | 74.56 | 399.17 | 510.22 | 530.50 | -2.76 | 0.000 | - && B0DN41.1 RecName: Full=Cyanate hydratase; Short=Cyanase; AltName: Full=Cyanate hydrolase; AltName: Full=Cyanate lyase && PF05347:Complex 1 protein (LYR family)\|PF02560:Cyanate lyase C-terminal domain |
| A01585 | Contig15 | 680596 | 682322 | - | 1323 |  | 8.20 | 10.70 | 9.18 | 53.50 | 58.52 | 63.22 | -2.64 | 0.000 | - && P75791.1 RecName: Full=Uncharacterized protein YbiU && PF07350:Protein of unknown function (DUF1479) |
| A01591 | Contig15 | 693529 | 695779 | - | 1428 |  | 2.55 | 2.11 | 3.13 | 38.89 | 39.00 | 30.17 | -3.79 | 0.000 | - && Q00667.1 RecName: Full=Homogentisate 1,2-dioxygenase; AltName: Full=Homogentisate oxygenase; AltName: Full=Homogentisic acid oxidase; AltName: Full=Homogentisicase && PF04209:homogentisate 1,2-dioxygenase |
| A01603 | Contig15 | 730330 | 732011 | - | 1227 |  | 23.71 | 21.60 | 16.68 | 5.18 | 4.04 | 9.73 | 1.71 | 0.000 | - && - && - |
| A01639 | Contig15 | 106991 | 107699 | + | 531 |  | 2.60 | 4.35 | 1.72 | 8.80 | 11.46 | 7.44 | -1.68 | 0.000 | - && - && - |
| A01646 | Contig15 | 126052 | 128219 | + | 1551 |  | 3.43 | 4.53 | 4.71 | 11.45 | 46.29 | 5.55 | -2.32 | 0.008 | - && Q99046.1 RecName: Full=Laccase-2; AltName: Full=Benzenediol:oxygen oxidoreductase 2; AltName: Full=Diphenol oxidase 2; AltName: Full=Urishiol oxidase 2; Flags: Precursor && PF00394:Multicopper oxidase\|PF07732:Multicopper oxidase\|PF07731:Multicopper oxidase |
| A01649 | Contig15 | 141039 | 145284 | + | 3048 |  | 3.36 | 4.45 | 4.59 | 8.31 | 11.15 | 6.33 | -1.06 | 0.000 | - && - && - |
| A01650 | Contig15 | 146781 | 148950 | + | 1422 |  | 8.18 | 8.75 | 23.32 | 141.69 | 69.91 | 108.68 | -2.99 | 0.000 | - && P29026.1 RecName: Full=Chitinase 1; Flags: Precursor && PF02839:Carbohydrate binding domain\|PF00704:Glycosyl hydrolases family 18 |
| A01653 | Contig15 | 153716 | 154700 | - | 858 |  | 4.60 | 5.15 | 5.75 | 2.18 | 1.53 | 3.17 | 1.17 | 0.004 | - && P37959.2 RecName: Full=Uncharacterized oxidoreductase YusZ; AltName: Full=ORFA && PF00106:short chain dehydrogenase |
| A01658 | Contig15 | 166396 | 168919 | + | 1776 |  | 79.56 | 97.84 | 83.79 | 162.09 | 213.73 | 215.79 | -1.18 | 0.000 | - && O59921.1 RecName: Full=Alpha,alpha-trehalose-phosphate synthase [UDP-forming]; AltName: Full=Trehalose-6-phosphate synthase; AltName: Full=UDP-glucose-glucosephosphate glucosyltransferase && PF00982:Glycosyltransferase family 20 |
| A01659 | Contig15 | 173053 | 173627 | - | 444 |  | 54.85 | 87.73 | 66.04 | 150.56 | 164.24 | 112.66 | -1.03 | 0.000 | - && - && - |
| A01667 | Contig15 | 184867 | 186828 | - | 1467 |  | 5.51 | 7.94 | 8.22 | 23.26 | 22.46 | 22.79 | -1.66 | 0.000 | - && - && PF14587:O-Glycosyl hydrolase family 30 |
| A01680 | Contig15 | 227561 | 228816 | - | 1020 |  | 0.58 | 0.69 | 0.63 | 2.66 | 1.74 | 3.44 | -2.05 | 0.000 | - && - && - |
| A01681 | Contig15 | 229700 | 230398 | + | 699 |  | 154.46 | 158.27 | 213.80 | 584.65 | 774.86 | 560.95 | -1.87 | 0.000 | - && - && - |
| A01699 | Contig15 | 285529 | 288136 | + | 1593 |  | 125.10 | 116.58 | 128.05 | 69.26 | 64.70 | 46.77 | 1.03 | 0.000 | - && Q99385.1 RecName: Full=Vacuolar calcium ion transporter; AltName: Full=High copy number undoes manganese protein 1; AltName: Full=Manganese resistance 1 protein; AltName: Full=Vacuolar Ca(2+)/H(+) exchanger && PF01699:Sodium/calcium exchanger protein |
| A01704 | Contig15 | 303203 | 304438 | - | 1077 |  | 127.99 | 175.05 | 45.63 | 23.09 | 23.55 | 32.02 | 2.15 | 0.000 | - && - && PF00107:Zinc-binding dehydrogenase |
| A01721 | Contig16 | 9943 | 11888 | - | 1452 |  | 303.35 | 296.12 | 338.35 | 579.91 | 665.85 | 892.94 | -1.19 | 0.000 | - && P51044.1 RecName: Full=Citrate synthase, mitochondrial; Flags: Precursor && PF00285:Citrate synthase |
| A01753 | Contig16 | 154206 | 154847 | - | 453 |  | 19.59 | 25.71 | 13.71 | 14.86 | 4.55 | 3.87 | 1.34 | 0.005 | - && - && - |
| A01756 | Contig16 | 161790 | 163902 | - | 1140 |  | 5.45 | 2.73 | 10.42 | 13.29 | 16.26 | 14.24 | -1.24 | 0.001 | - && Q50228.1 RecName: Full=Formamidase; AltName: Full=Formamide amidohydrolase && PF03069:Acetamidase/Formamidase family |
| A01764 | Contig16 | 192231 | 192859 | - | 453 |  | 206.78 | 109.47 | 226.46 | 14.45 | 38.02 | 10.07 | 3.12 | 0.000 | - && - && PF16850:Peptidase inhibitor I66 |
| A01765 | Contig16 | 198199 | 198855 | + | 453 |  | 129.08 | 109.03 | 123.01 | 16.72 | 9.30 | 11.62 | 3.26 | 0.000 | - && - && - |
| A01790 | Contig16 | 298099 | 303623 | - | 3372 |  | 527.93 | 609.65 | 261.42 | 109.00 | 57.24 | 106.71 | 2.36 | 0.000 | - && P53134.1 RecName: Full=Putative oligopeptide transporter YGL114W && PF03169:OPT oligopeptide transporter protein |
| A01792 | Contig16 | 307477 | 309295 | + | 1182 |  | 6.84 | 8.32 | 7.03 | 28.00 | 27.80 | 25.17 | -1.87 | 0.000 | - && Q1HFS8.1 RecName: Full=Endo-beta-1,4-glucanase B; Short=Endoglucanase B; AltName: Full=Carboxymethylcellulase B; AltName: Full=Cellulase B; Flags: Precursor && PF00734:Fungal cellulose binding domain\|PF00150:Cellulase (glycosyl hydrolase family 5) |
| A01803 | Contig16 | 363861 | 364688 | + | 828 |  | 6.91 | 5.70 | 6.51 | 3.50 | 3.96 | 1.80 | 1.05 | 0.007 | - && - && PF01753:MYND finger |
| A01804 | Contig16 | 370162 | 375840 | - | 2652 |  | 30.71 | 34.79 | 28.25 | 12.44 | 13.80 | 14.30 | 1.21 | 0.000 | - && O74790.1 RecName: Full=Monothiol glutaredoxin-4 && PF00462:Glutaredoxin\|PF03731:Ku70/Ku80 N-terminal alpha/beta domain\|PF03730:Ku70/Ku80 C-terminal arm\|PF00085:Thioredoxin\|PF02735:Ku70/Ku80 beta-barrel domain |
| A01815 | Contig16 | 414248 | 416527 | - | 2217 |  | 0.00 | 0.00 | 0.21 | 0.17 | 0.76 | 0.36 | -2.64 | 0.029 | - && - && PF03221:Tc5 transposase DNA-binding domain\|PF03184:DDE superfamily endonuclease |
| A01816 | Contig16 | 417715 | 419272 | + | 1272 |  | 9.69 | 12.55 | 10.20 | 32.49 | 32.38 | 80.17 | -2.16 | 0.000 | - && - && PF00642:Zinc finger C-x8-C-x5-C-x3-H type (and similar) |
| A01819 | Contig16 | 433309 | 435375 | - | 1776 |  | 62.68 | 80.49 | 86.41 | 263.75 | 200.50 | 182.58 | -1.49 | 0.000 | - && - && - |
| A01826 | Contig16 | 457627 | 460152 | - | 1746 |  | 70.65 | 90.90 | 73.35 | 214.46 | 117.68 | 179.09 | -1.12 | 0.000 | - && - && PF16862:Glycosyl hydrolase family 79 C-terminal beta domain |
| A01840 | Contig16 | 517097 | 518748 | + | 1533 |  | 4.89 | 7.86 | 5.66 | 10.98 | 13.31 | 17.17 | -1.17 | 0.000 | - && - && - |
| A01842 | Contig16 | 521604 | 525807 | - | 2661 |  | 22.94 | 28.48 | 26.12 | 57.69 | 53.89 | 64.18 | -1.18 | 0.000 | - && - && PF06966:Protein of unknown function (DUF1295) |
| A01858 | Contig16 | 600011 | 602468 | - | 1665 |  | 12.67 | 23.76 | 6.20 | 33.52 | 18.27 | 43.06 | -1.15 | 0.005 | - && Q00019.1 RecName: Full=Rhamnogalacturonate lyase A; AltName: Full=Rhamnogalacturonan lyase; Flags: Precursor && PF09284:Rhamnogalacturonan lyase B, N-terminal\|PF14683:Polysaccharide lyase family 4, domain III\|PF14686:Polysaccharide lyase family 4, domain II |
| A01861 | Contig16 | 616631 | 618312 | + | 1128 |  | 17.05 | 14.60 | 18.87 | 8.79 | 10.71 | 3.73 | 1.12 | 0.001 | - && D4AMB9.1 RecName: Full=Probable aldose 1-epimerase ARB_05372; AltName: Full=Galactose mutarotase; Flags: Precursor && PF01263:Aldose 1-epimerase |
| A01866 | Contig16 | 629214 | 634362 | + | 3294 |  | 97.53 | 76.80 | 65.75 | 19.87 | 7.33 | 9.91 | 2.69 | 0.000 | - && B8NPT0.1 RecName: Full=Probable feruloyl esterase B-2; AltName: Full=Ferulic acid esterase B-2; Short=FAEB-2; Flags: Precursor && PF07519:Tannase and feruloyl esterase |
| A01869 | Contig16 | 647168 | 649767 | + | 1827 |  | 12.41 | 18.96 | 11.60 | 3.28 | 6.40 | 6.63 | 1.40 | 0.000 | - && Q99385.1 RecName: Full=Vacuolar calcium ion transporter; AltName: Full=High copy number undoes manganese protein 1; AltName: Full=Manganese resistance 1 protein; AltName: Full=Vacuolar Ca(2+)/H(+) exchanger && PF01699:Sodium/calcium exchanger protein |
| A01871 | Contig16 | 654205 | 655874 | - | 1323 |  | 29.14 | 39.84 | 21.61 | 106.85 | 125.03 | 119.80 | -1.96 | 0.000 | - && Q01682.1 RecName: Full=Thiamine-repressible acid phosphatase pho4; Flags: Precursor && PF00328:Histidine phosphatase superfamily (branch 2) |
| A01872 | Contig16 | 656921 | 657774 | - | 618 |  | 18235.49 | 23987.09 | 12735.45 | 2167.35 | 1484.59 | 1493.60 | 3.42 | 0.000 | - && - && - |
| A01882 | Contig16 | 687627 | 690187 | - | 1692 |  | 3.32 | 3.56 | 3.94 | 15.03 | 11.40 | 10.84 | -1.78 | 0.000 | - && P46333.3 RecName: Full=Probable metabolite transport protein CsbC && PF00083:Sugar (and other) transporter |
| A01886 | Contig16 | 701399 | 703519 | - | 1551 |  | 10.43 | 9.26 | 13.90 | 44.31 | 57.16 | 27.61 | -1.94 | 0.000 | - && - && PF16862:Glycosyl hydrolase family 79 C-terminal beta domain |
| A01889 | Contig16 | 724402 | 726898 | - | 1647 |  | 12.93 | 25.97 | 7.49 | 70.73 | 87.93 | 45.82 | -2.14 | 0.000 | - && Q10499.3 RecName: Full=Apoptosis-inducing factor 1 && PF07992:Pyridine nucleotide-disulphide oxidoreductase\|PF00355:Rieske [2Fe-2S] domain\|PF14759:Reductase C-terminal |
| A01892 | Contig16 | 731739 | 737960 | + | 3129 |  | 67.59 | 69.94 | 58.27 | 19.09 | 12.95 | 22.13 | 1.85 | 0.000 | - && - && - |
| A01895 | Contig16 | 744790 | 745469 | - | 555 |  | 15.46 | 17.36 | 7.08 | 14.32 | 35.59 | 70.52 | -1.59 | 0.001 | - && - && - |
| A01912 | Contig16 | 808320 | 810001 | + | 1227 |  | 15.43 | 17.34 | 17.20 | 5.11 | 4.20 | 7.37 | 1.58 | 0.000 | - && - && - |
| A01915 | Contig16 | 824139 | 825086 | - | 753 |  | 4.85 | 9.33 | 1.58 | 1.49 | 0.87 | 1.28 | 2.11 | 0.001 | - && - && PF13430:Domain of unknown function (DUF4112) |
| A01923 | Contig17 | 29949 | 30474 | - | 465 |  | 37.32 | 36.27 | 21.22 | 73.19 | 72.07 | 69.07 | -1.18 | 0.000 | - && - && - |
| A01934 | Contig17 | 78138 | 83440 | - | 2883 |  | 26.99 | 28.66 | 16.44 | 10.22 | 11.75 | 10.20 | 1.16 | 0.000 | - && Q9URX1.1 RecName: Full=UNC93-like protein C922.05c && PF05978:Ion channel regulatory protein UNC-93\|PF07855:Protein of unknown function (DUF1649) |
| A01940 | Contig17 | 103770 | 104873 | + | 873 |  | 6.33 | 6.32 | 5.86 | 2.89 | 3.86 | 2.31 | 1.03 | 0.004 | - && - && - |
| A01945 | Contig17 | 120658 | 122088 | - | 1260 |  | 0.31 | 0.32 | 0.58 | 2.67 | 1.63 | 1.46 | -2.25 | 0.000 | - && - && - |
| A01951 | Contig17 | 140562 | 141599 | - | 1038 |  | 6.36 | 4.06 | 5.81 | 1.17 | 1.62 | 1.44 | 1.94 | 0.000 | - && - && - |
| A01954 | Contig17 | 151041 | 152573 | + | 1032 |  | 3.25 | 6.52 | 2.74 | 39.68 | 62.13 | 33.42 | -3.43 | 0.000 | - && B0Y9G4.1 RecName: Full=Probable endo-beta-1,4-glucanase D; Short=Endoglucanase D; AltName: Full=Carboxymethylcellulase D; AltName: Full=Cellulase D; Flags: Precursor && PF03443:Glycosyl hydrolase family 61\|PF00734:Fungal cellulose binding domain |
| A01955 | Contig17 | 152899 | 154756 | - | 1353 |  | 4.08 | 1.93 | 4.19 | 9.88 | 8.65 | 5.51 | -1.24 | 0.001 | - && - && PF14737:Domain of unknown function (DUF4470) |
| A01961 | Contig17 | 169915 | 170437 | + | 294 |  | 8.38 | 4.10 | 2.80 | 0.64 | 0.64 | 1.49 | 2.47 | 0.003 | - && - && - |
| A01966 | Contig17 | 185827 | 187091 | - | 762 |  | 1.16 | 3.82 | 4.20 | 7.85 | 7.25 | 4.26 | -1.08 | 0.025 | - && Q54DD7.1 RecName: Full=Protein unc-50 homolog && PF05216:UNC-50 family |
| A01979 | Contig17 | 253085 | 256602 | - | 2823 |  | 0.42 | 0.92 | 0.45 | 0.07 | 0.20 | 0.25 | 1.81 | 0.011 | - && - && - |
| A02001 | Contig17 | 343642 | 345175 | - | 1032 |  | 5.83 | 6.91 | 3.45 | 10.24 | 20.50 | 13.01 | -1.43 | 0.000 | - && B0Y9G4.1 RecName: Full=Probable endo-beta-1,4-glucanase D; Short=Endoglucanase D; AltName: Full=Carboxymethylcellulase D; AltName: Full=Cellulase D; Flags: Precursor && PF00734:Fungal cellulose binding domain\|PF03443:Glycosyl hydrolase family 61 |
| A02004 | Contig17 | 354792 | 355673 | + | 882 |  | 6.04 | 2.73 | 4.14 | 2.54 | 2.23 | 1.09 | 1.14 | 0.017 | - && - && - |
| A02009 | Contig17 | 371339 | 372769 | + | 1260 |  | 0.63 | 0.16 | 0.14 | 0.89 | 0.89 | 1.53 | -1.83 | 0.020 | - && - && - |
| A02010 | Contig17 | 375056 | 377939 | + | 2400 |  | 14.59 | 10.67 | 17.58 | 7.05 | 8.07 | 5.67 | 1.04 | 0.000 | - && Q4WMP0.1 RecName: Full=Probable glucan 1,3-beta-glucosidase D; AltName: Full=Exo-1,3-beta-glucanase D >B0Y7W2.1 RecName: Full=Probable glucan 1,3-beta-glucosidase D; AltName: Full=Exo-1,3-beta-glucanase D && PF00150:Cellulase (glycosyl hydrolase family 5) |
| A02021 | Contig17 | 427739 | 429900 | + | 1803 |  | 4.87 | 3.79 | 3.80 | 51.60 | 38.52 | 59.67 | -3.59 | 0.000 | - && A1DBW0.1 RecName: Full=Extracellular metalloproteinase MEP; AltName: Full=Elastinolytic metalloproteinase MEP; AltName: Full=Fungalysin MEP; Flags: Precursor && PF07504:Fungalysin/Thermolysin Propeptide Motif\|PF02128:Fungalysin metallopeptidase (M36) |
| A02023 | Contig17 | 438444 | 440733 | + | 1638 |  | 10.17 | 11.64 | 9.48 | 3.08 | 3.60 | 3.00 | 1.69 | 0.000 | - && P87035.1 RecName: Full=Guanine nucleotide-binding protein alpha-4 subunit && PF00503:G-protein alpha subunit |
| A02024 | Contig17 | 441027 | 442419 | - | 1278 |  | 0.93 | 1.34 | 0.71 | 2.71 | 1.61 | 2.68 | -1.23 | 0.013 | - && - && PF01753:MYND finger |
| A02030 | Contig17 | 462096 | 463234 | - | 1071 |  | 22.00 | 23.81 | 22.35 | 35.79 | 41.95 | 64.32 | -1.06 | 0.000 | - && - && PF05859:Mis12 protein |
| A02032 | Contig17 | 469781 | 471051 | - | 1209 |  | 5.46 | 4.07 | 5.67 | 14.31 | 20.13 | 7.48 | -1.46 | 0.000 | - && - && - |
| A02037 | Contig17 | 479810 | 485133 | + | 1752 |  | 44.07 | 35.24 | 36.39 | 22.15 | 18.11 | 17.03 | 1.01 | 0.000 | - && Q10087.1 RecName: Full=Uncharacterized amino-acid permease C11D3.08c && PF13520:Amino acid permease\|PF03226:Yippee zinc-binding/DNA-binding /Mis18, centromere assembly |
| A02040 | Contig17 | 494121 | 495471 | + | 1272 |  | 197.83 | 161.71 | 290.42 | 308.93 | 493.52 | 677.85 | -1.19 | 0.000 | - && - && - |
| A02075 | Contig17 | 663349 | 666615 | - | 2781 |  | 2.59 | 2.89 | 3.32 | 1.04 | 0.77 | 0.73 | 1.79 | 0.000 | - && - && - |
| A02084 | Contig17 | 703257 | 703835 | - | 579 |  | 0.00 | 0.17 | 0.00 | 0.81 | 2.59 | 1.97 | -4.95 | 0.001 | - && - && - |
| A02086 | Contig18 | 2701 | 4448 | - | 1617 |  | 0.12 | 0.93 | 0.34 | 1.39 | 1.16 | 1.47 | -1.53 | 0.020 | - && - && PF00046:Homeobox domain |
| A02089 | Contig18 | 16283 | 19948 | + | 2184 |  | 2.35 | 2.16 | 3.10 | 1.37 | 1.33 | 0.92 | 1.07 | 0.003 | - && O13728.1 RecName: Full=Alpha-actinin-like protein 1 && PF00307:Calponin homology (CH) domain\|PF08726:Ca2+ insensitive EF hand\|PF00241:Cofilin/tropomyosin-type actin-binding protein |
| A02099 | Contig18 | 69839 | 71700 | - | 1803 |  | 151.21 | 124.83 | 136.64 | 38.06 | 41.07 | 48.04 | 1.70 | 0.000 | - && - && - |
| A02106 | Contig18 | 97719 | 108474 | + | 6120 |  | 19.04 | 15.29 | 19.63 | 43.80 | 55.39 | 37.17 | -1.34 | 0.000 | - && D4ATR3.1 RecName: Full=Uncharacterized secreted glycosidase ARB_07629; Flags: Precursor && PF07971:Glycosyl hydrolase family 92 |
| A02110 | Contig18 | 117445 | 118160 | + | 606 |  | 54.02 | 47.21 | 44.02 | 11.26 | 12.20 | 15.50 | 1.90 | 0.000 | - && - && PF00786:P21-Rho-binding domain\|PF00169:PH domain |
| A02112 | Contig18 | 120289 | 121887 | - | 1551 |  | 41.32 | 34.17 | 42.23 | 10.73 | 14.18 | 8.43 | 1.82 | 0.000 | - && - && PF00890:FAD binding domain |
| A02116 | Contig18 | 133826 | 142029 | + | 6042 |  | 16.24 | 18.28 | 24.51 | 66.18 | 43.41 | 32.96 | -1.27 | 0.000 | - && Q09706.1 RecName: Full=Phospholipase D1; Short=PLD 1; AltName: Full=Choline phosphatase 1; AltName: Full=Phosphatidylcholine-hydrolyzing phospholipase D1 && PF00787:PX domain\|PF13091:PLD-like domain\|PF01267:F-actin capping protein alpha subunit |
| A02121 | Contig18 | 158360 | 163935 | - | 2958 |  | 18.97 | 21.99 | 24.98 | 43.84 | 40.32 | 51.92 | -1.05 | 0.000 | - && Q8JGT5.1 RecName: Full=SUMO-activating enzyme subunit 1; AltName: Full=SUMO-activating enzyme E1 N subunit; AltName: Full=Ubiquitin-like 1-activating enzyme E1A && PF00899:ThiF family\|PF04082:Fungal specific transcription factor domain |
| A02124 | Contig18 | 173967 | 176261 | + | 1623 |  | 6.56 | 5.94 | 6.36 | 26.84 | 56.29 | 17.95 | -2.42 | 0.000 | - && Q0CVU1.2 RecName: Full=Probable rhamnogalacturonate lyase A; Flags: Precursor && PF14686:Polysaccharide lyase family 4, domain II\|PF14683:Polysaccharide lyase family 4, domain III\|PF09284:Rhamnogalacturonan lyase B, N-terminal |
| A02126 | Contig18 | 181865 | 185897 | + | 2574 |  | 403.91 | 431.66 | 415.94 | 1910.34 | 2753.98 | 3094.19 | -2.63 | 0.000 | - && A8NR45.1 RecName: Full=Isocitrate lyase; Short=ICL; Short=Isocitrase; Short=Isocitratase; AltName: Full=Methylisocitrate lyase; Short=MICA; AltName: Full=Threo-D(S)-isocitrate glyoxylate-lyase && PF00463:Isocitrate lyase family\|PF00953:Glycosyl transferase family 4 |
| A02139 | Contig18 | 267369 | 269987 | + | 1983 |  | 12.13 | 10.88 | 10.55 | 21.74 | 21.29 | 24.65 | -1.01 | 0.000 | - && - && PF04000:Sas10/Utp3/C1D family\|PF09368:Sas10 C-terminal domain |
| A02140 | Contig18 | 270670 | 274135 | - | 1956 |  | 90.64 | 81.55 | 93.17 | 53.49 | 34.94 | 28.80 | 1.18 | 0.000 | - && - && - |
| A02146 | Contig18 | 292633 | 293145 | + | 393 |  | 1468.26 | 1895.37 | 1006.24 | 443.69 | 657.65 | 1033.68 | 1.03 | 0.001 | - && - && - |
| A02154 | Contig18 | 325227 | 326196 | + | 852 |  | 11.57 | 29.69 | 10.51 | 2.41 | 6.48 | 4.43 | 1.96 | 0.000 | - && - && - |
| A02166 | Contig18 | 356865 | 358601 | + | 1359 |  | 392.16 | 286.91 | 342.41 | 5.64 | 6.27 | 4.97 | 5.92 | 0.000 | - && P54387.1 RecName: Full=NADP-specific glutamate dehydrogenase; Short=NADP-GDH; AltName: Full=NADP-dependent glutamate dehydrogenase && PF02812:Glu/Leu/Phe/Val dehydrogenase, dimerisation domain\|PF00208:Glutamate/Leucine/Phenylalanine/Valine dehydrogenase |
| A02174 | Contig18 | 388993 | 394665 | + | 3228 |  | 45.73 | 58.44 | 36.25 | 21.40 | 10.44 | 21.72 | 1.39 | 0.000 | - && Q9US41.1 RecName: Full=Uracil-regulated protein 1 && PF00925:GTP cyclohydrolase II\|PF12471:GTP cyclohydrolase N terminal |
| A02178 | Contig18 | 403348 | 403750 | - | 288 |  | 22.94 | 34.86 | 7.93 | 0.97 | 2.60 | 1.22 | 3.78 | 0.000 | - && - && - |
| A02179 | Contig18 | 404167 | 404575 | - | 291 |  | 206.69 | 284.61 | 61.53 | 21.53 | 12.55 | 20.81 | 3.33 | 0.000 | - && - && - |
| A02188 | Contig18 | 438587 | 439000 | - | 414 |  | 8.57 | 9.46 | 6.62 | 3.16 | 0.00 | 3.18 | 1.96 | 0.003 | - && - && - |
| A02190 | Contig18 | 444862 | 445395 | - | 534 |  | 4.62 | 6.39 | 4.79 | 15.41 | 12.10 | 15.45 | -1.44 | 0.000 | - && - && - |
| A02191 | Contig18 | 448168 | 452653 | - | 2736 |  | 24.22 | 20.84 | 27.65 | 63.46 | 57.04 | 77.01 | -1.44 | 0.000 | - && O14123.1 RecName: Full=Probable Na(+)/H(+) antiporter C3A11.09 && PF00999:Sodium/hydrogen exchanger family |
| A02195 | Contig18 | 461002 | 461985 | + | 867 |  | 9.10 | 6.83 | 8.85 | 15.85 | 36.82 | 11.24 | -1.37 | 0.001 | - && - && PF12146:Serine aminopeptidase, S33 |
| A02210 | Contig18 | 519573 | 525628 | - | 3153 |  | 50.16 | 62.82 | 57.31 | 8.48 | 15.47 | 4.12 | 2.60 | 0.000 | - && G5EDB9.1 RecName: Full=Rap guanine nucleotide exchange factor; Short=RA-GEF; AltName: Full=PDZ-domain-containing exchange factor && PF00617:RasGEF domain\|PF00618:RasGEF N-terminal motif |
| A02217 | Contig18 | 553202 | 556813 | + | 1614 |  | 51.50 | 50.19 | 43.01 | 185.08 | 223.82 | 149.85 | -1.95 | 0.000 | - && A8NSD1.2 RecName: Full=Thiamine thiazole synthase; AltName: Full=Thiazole biosynthetic enzyme && PF01946:Thi4 family |
| A02232 | Contig18 | 604741 | 608209 | - | 2793 |  | 0.78 | 0.32 | 0.23 | 0.30 | 0.03 | 0.03 | 1.86 | 0.047 | - && - && - |
| A02242 | Contig18 | 653662 | 655820 | + | 1203 |  | 1.39 | 2.92 | 1.82 | 0.47 | 0.54 | 0.51 | 2.01 | 0.001 | - && - && - |
| A02249 | Contig19 | 26445 | 28167 | + | 921 |  | 5.25 | 8.07 | 5.55 | 3.15 | 3.46 | 1.33 | 1.25 | 0.003 | - && - && PF00069:Protein kinase domain |
| A02256 | Contig19 | 61031 | 62956 | + | 1455 |  | 68.65 | 57.82 | 77.35 | 32.19 | 26.25 | 43.31 | 1.00 | 0.000 | - && - && - |
| A02262 | Contig19 | 83832 | 87336 | + | 3174 |  | 0.06 | 0.16 | 0.20 | 0.03 | 0.00 | 0.00 | 3.84 | 0.024 | - && - && - |
| A02263 | Contig19 | 91410 | 92781 | - | 990 |  | 8.57 | 9.63 | 9.97 | 32.11 | 32.15 | 42.55 | -1.92 | 0.000 | - && Q9USW3.1 RecName: Full=Probable glycosidase C21B10.07 && - |
| A02269 | Contig19 | 126335 | 127061 | - | 606 |  | 61.18 | 80.51 | 55.17 | 33.02 | 32.13 | 25.78 | 1.11 | 0.000 | - && P36088.1 RecName: Full=Free methionine-R-sulfoxide reductase; Short=fRMsr; AltName: Full=GAF domain-containing protein YKL069W && - |
| A02271 | Contig19 | 131831 | 132996 | + | 900 |  | 97.18 | 81.87 | 98.86 | 37.50 | 38.48 | 25.16 | 1.46 | 0.000 | - && - && PF09729:Gti1/Pac2 family |
| A02273 | Contig19 | 138400 | 139392 | - | 993 |  | 167.12 | 138.30 | 142.22 | 69.96 | 70.70 | 78.92 | 1.03 | 0.000 | - && - && PF07716:Basic region leucine zipper |
| A02289 | Contig19 | 194451 | 195052 | + | 423 |  | 72.26 | 68.11 | 63.49 | 34.26 | 33.64 | 22.61 | 1.17 | 0.000 | - && - && PF13380:CoA binding domain |
| A02296 | Contig19 | 227698 | 229841 | - | 1461 |  | 239.52 | 198.58 | 252.60 | 823.16 | 487.40 | 1054.54 | -1.78 | 0.000 | - && Q05744.1 RecName: Full=Cathepsin D; Contains: RecName: Full=Cathepsin D light chain; Contains: RecName: Full=Cathepsin D heavy chain; Flags: Precursor && PF00026:Eukaryotic aspartyl protease |
| A02298 | Contig19 | 235640 | 238861 | - | 1338 |  | 8.25 | 6.98 | 15.57 | 4.26 | 4.76 | 1.90 | 1.50 | 0.000 | - && - && - |
| A02306 | Contig19 | 267729 | 269399 | + | 966 |  | 1.74 | 2.81 | 2.08 | 9.58 | 22.58 | 6.54 | -2.55 | 0.000 | - && Q5AVN4.1 RecName: Full=Pectate lyase A; Flags: Precursor && PF00544:Pectate lyase |
| A02311 | Contig19 | 289520 | 292723 | + | 2529 |  | 37.78 | 30.13 | 42.04 | 18.93 | 16.43 | 19.19 | 1.01 | 0.000 | - && Q96JB2.3 RecName: Full=Conserved oligomeric Golgi complex subunit 3; Short=COG complex subunit 3; AltName: Full=Component of oligomeric Golgi complex 3; AltName: Full=Vesicle-docking protein SEC34 homolog; AltName: Full=p94 && PF04136:Sec34-like family |
| A02316 | Contig19 | 309828 | 311265 | - | 1095 |  | 51.15 | 41.26 | 52.06 | 20.15 | 31.46 | 16.43 | 1.09 | 0.000 | - && Q76NT9.1 RecName: Full=1-aminocyclopropane-1-carboxylate oxidase; Short=ACC oxidase; Short=Ddaco; AltName: Full=Ethylene-forming enzyme; Short=EFE >A6BM06.1 RecName: Full=1-aminocyclopropane-1-carboxylate oxidase; Short=ACC oxidase; Short=Dmaco; AltName: Full=Ethylene-forming enzyme; Short=EFE && PF03171:2OG-Fe(II) oxygenase superfamily\|PF14226:non-haem dioxygenase in morphine synthesis N-terminal |
| A02330 | Contig19 | 375884 | 376735 | - | 462 |  | 535.70 | 1006.05 | 257.44 | 7.49 | 10.33 | 2.66 | 6.46 | 0.000 | - && - && - |
| A02331 | Contig19 | 379273 | 379686 | - | 255 |  | 5.03 | 4.72 | 5.02 | 29.70 | 34.87 | 8.60 | -2.31 | 0.000 | - && - && - |
| A02363 | Contig19 | 488205 | 497600 | - | 5229 |  | 20.76 | 12.81 | 23.57 | 6.47 | 6.23 | 2.99 | 1.86 | 0.000 | - && P28829.1 RecName: Full=Protein kinase byr2; AltName: Full=MAPK kinase kinase; Short=MAPKKK; AltName: Full=Protein kinase ste8 && PF00271:Helicase conserved C-terminal domain\|PF00069:Protein kinase domain\|PF05729:NACHT domain\|PF00270:DEAD/DEAH box helicase |
| A02369 | Contig19 | 515035 | 517339 | - | 1665 |  | 0.83 | 1.51 | 1.26 | 2.36 | 5.17 | 18.92 | -2.88 | 0.001 | - && P06026.2 RecName: Full=Rhizopuspepsin; Flags: Precursor && PF00026:Eukaryotic aspartyl protease |
| A02384 | Contig19 | 571694 | 575015 | - | 2736 |  | 2.67 | 4.18 | 4.34 | 47.12 | 65.90 | 54.27 | -3.90 | 0.000 | - && Q0CI48.2 RecName: Full=Beta-mannosidase A; AltName: Full=Mannanase A; Short=Mannase A; Flags: Precursor && - |
| A02385 | Contig19 | 576058 | 584321 | + | 4848 |  | 20.38 | 21.54 | 17.15 | 130.27 | 148.51 | 128.43 | -2.79 | 0.000 | - && - && - |
| A02389 | Contig19 | 601174 | 602777 | + | 1053 |  | 15.54 | 17.35 | 10.32 | 63.04 | 36.27 | 68.67 | -1.96 | 0.000 | - && - && - |
| A02397 | Contig1 | 3705031 | 3706701 | - | 951 |  | 4.98 | 1.79 | 4.32 | 1.08 | 0.79 | 0.92 | 1.99 | 0.000 | - && P24504.1 RecName: Full=Nuclease PA3; AltName: Full=Deoxyribonuclease PA3; AltName: Full=Endonuclease PA3 && PF02265:S1/P1 Nuclease |
| A02402 | Contig1 | 3720905 | 3721892 | - | 798 |  | 17.55 | 13.71 | 12.48 | 127.59 | 147.92 | 114.81 | -3.16 | 0.000 | - && Q5BG78.1 RecName: Full=Xyloglucan-specific endo-beta-1,4-glucanase A; AltName: Full=Xyloglucanase A; AltName: Full=Xyloglucanendohydrolase A; Flags: Precursor && PF01670:Glycosyl hydrolase family 12 |
| A02407 | Contig1 | 3729571 | 3732868 | - | 1572 |  | 318.45 | 294.20 | 280.56 | 138.16 | 125.77 | 137.00 | 1.16 | 0.000 | - && P25451.1 RecName: Full=Proteasome subunit beta type-3; AltName: Full=Macropain subunit PUP3; AltName: Full=Multicatalytic endopeptidase complex subunit PUP3; AltName: Full=Proteasome component PUP3 && PF00227:Proteasome subunit |
| A02408 | Contig1 | 3733761 | 3734624 | - | 741 |  | 33.13 | 36.85 | 56.71 | 9.97 | 8.34 | 6.04 | 2.38 | 0.000 | - && - && PF13417:Glutathione S-transferase, N-terminal domain |
| A02424 | Contig1 | 3789608 | 3796782 | - | 3894 |  | 19.37 | 14.75 | 20.29 | 10.20 | 7.91 | 7.57 | 1.08 | 0.000 | - && Q8N0N3.1 RecName: Full=Beta-1,3-glucan-binding protein; Short=GBP; Flags: Precursor && PF00722:Glycosyl hydrolases family 16 |
| A02425 | Contig1 | 3798924 | 3807790 | + | 3864 |  | 14.70 | 13.30 | 15.34 | 5.86 | 8.79 | 5.22 | 1.12 | 0.000 | - && - && PF13632:Glycosyl transferase family group 2 |
| A02429 | Contig1 | 3813487 | 3817024 | - | 2598 |  | 28.65 | 30.14 | 28.55 | 111.78 | 103.05 | 122.96 | -1.95 | 0.000 | - && A2R797.1 RecName: Full=Probable alpha-fucosidase A; AltName: Full=Alpha-L-fucoside fucohydrolase A; Flags: Precursor && PF14498:Glycosyl hydrolase family 65, N-terminal domain |
| A02449 | Contig1 | 3888482 | 3891525 | - | 1473 |  | 0.67 | 4.63 | 1.61 | 7.93 | 10.49 | 3.34 | -1.65 | 0.003 | - && - && - |
| A02450 | Contig1 | 3892044 | 3892780 | - | 525 |  | 54.09 | 65.20 | 44.54 | 15.14 | 22.11 | 15.04 | 1.65 | 0.000 | - && Q2UB88.1 RecName: Full=3-hydroxyanthranilate 3,4-dioxygenase 2; AltName: Full=3-hydroxyanthranilate oxygenase 2; Short=3-HAO-2; AltName: Full=3-hydroxyanthranilic acid dioxygenase 2; Short=HAD-2; AltName: Full=Biosynthesis of nicotinic acid protein 1-2 && PF06052:3-hydroxyanthranilic acid dioxygenase |
| A02469 | Contig1 | 3951231 | 3952993 | - | 1707 |  | 5.31 | 6.53 | 4.12 | 21.85 | 42.72 | 55.16 | -2.91 | 0.000 | - && - && PF05199:GMC oxidoreductase |
| A02472 | Contig1 | 3958726 | 3960068 | - | 1107 |  | 163.80 | 163.96 | 64.70 | 14.10 | 10.06 | 9.28 | 3.55 | 0.000 | - && - && - |
| A02475 | Contig1 | 3964897 | 3966823 | - | 1005 |  | 6.38 | 6.79 | 4.18 | 3.81 | 1.58 | 2.18 | 1.19 | 0.004 | - && - && - |
| A02498 | Contig1 | 4037269 | 4038964 | - | 1065 |  | 1960.86 | 2140.58 | 1613.16 | 519.10 | 903.76 | 102.18 | 1.91 | 0.018 | - && Q96TS6.1 RecName: Full=Manganese peroxidase 3; Short=MnP3; AltName: Full=Manganese peroxidase isozyme 3; Flags: Precursor && PF11895:Domain of unknown function (DUF3415)\|PF00141:Peroxidase |
| A02502 | Contig1 | 4059032 | 4060829 | - | 1746 |  | 37.33 | 64.11 | 27.05 | 17.78 | 18.66 | 12.52 | 1.39 | 0.000 | - && - && PF05199:GMC oxidoreductase |
| A02505 | Contig1 | 4062472 | 4064010 | + | 1422 |  | 46.53 | 113.87 | 27.17 | 8.61 | 10.01 | 7.22 | 2.86 | 0.000 | - && O94562.1 RecName: Full=Uncharacterized aminotransferase C1771.03c && PF00202:Aminotransferase class-III |
| A02506 | Contig1 | 4066957 | 4067982 | + | 852 |  | 43.75 | 58.56 | 47.18 | 6.69 | 4.72 | 6.28 | 3.08 | 0.000 | - && O74628.1 RecName: Full=Uncharacterized oxidoreductase C162.03 && PF00106:short chain dehydrogenase |
| A02517 | Contig1 | 4096528 | 4097372 | - | 666 |  | 101.71 | 112.30 | 206.43 | 40.01 | 35.28 | 64.57 | 1.59 | 0.000 | - && - && - |
| A02539 | Contig1 | 4217854 | 4218636 | + | 783 |  | 2.14 | 0.77 | 0.82 | 0.24 | 0.24 | 0.45 | 2.01 | 0.029 | - && - && - |
| A02550 | Contig1 | 4276908 | 4279615 | + | 2538 |  | 0.35 | 0.75 | 0.76 | 0.85 | 1.22 | 2.04 | -1.14 | 0.024 | - && - && - |
| A02554 | Contig1 | 4295189 | 4297468 | + | 2217 |  | 0.04 | 0.23 | 0.00 | 0.38 | 0.51 | 0.32 | -2.15 | 0.037 | - && - && PF03221:Tc5 transposase DNA-binding domain\|PF03184:DDE superfamily endonuclease |
| A02557 | Contig1 | 4308542 | 4308963 | + | 369 |  | 619.68 | 443.72 | 705.55 | 46.11 | 89.30 | 31.63 | 3.40 | 0.000 | - && - && PF11779:Protein of unknown function (DUF3317) |
| A02564 | Contig1 | 4340382 | 4341523 | + | 819 |  | 393.57 | 513.10 | 198.43 | 122.72 | 116.13 | 105.22 | 1.68 | 0.000 | - && - && PF00106:short chain dehydrogenase |
| A02566 | Contig1 | 4345316 | 4346486 | + | 774 |  | 3.06 | 4.54 | 6.37 | 57.14 | 51.04 | 23.02 | -3.23 | 0.000 | - && - && PF00644:Poly(ADP-ribose) polymerase catalytic domain |
| A02570 | Contig1 | 507449 | 511833 | - | 2469 |  | 8.11 | 9.84 | 4.74 | 18.82 | 19.94 | 16.31 | -1.28 | 0.000 | - && F4HUK6.1 RecName: Full=Probable acyl-activating enzyme 1, peroxisomal; AltName: Full=AMP-binding protein 1; Short=AtAMPBP1 && PF13193:AMP-binding enzyme C-terminal domain\|PF00501:AMP-binding enzyme |
| A02585 | Contig1 | 4393139 | 4396122 | - | 2499 |  | 14.40 | 29.33 | 18.68 | 58.07 | 58.59 | 25.60 | -1.19 | 0.000 | - && - && PF14613:Protein of unknown function (DUF4449) |
| A02588 | Contig1 | 4416389 | 4418639 | + | 1611 |  | 52.70 | 34.40 | 55.29 | 15.90 | 21.73 | 13.73 | 1.47 | 0.000 | - && D7PHY8.1 RecName: Full=Efflux pump vrtL; AltName: Full=Viridicatumtoxin synthesis protein L && PF07690:Major Facilitator Superfamily |
| A02595 | Contig1 | 4432157 | 4433455 | + | 819 |  | 239.71 | 196.24 | 260.33 | 67.01 | 50.29 | 40.93 | 2.14 | 0.000 | - && Q552M5.2 RecName: Full=Ras guanine nucleotide exchange factor Y; AltName: Full=RasGEF domain-containing protein Y && PF00617:RasGEF domain |
| A02598 | Contig1 | 4440595 | 4441959 | + | 1296 |  | 428.65 | 440.36 | 363.57 | 123.80 | 100.18 | 141.05 | 1.76 | 0.000 | - && - && - |
| A02609 | Contig1 | 4482361 | 4486527 | - | 3303 |  | 81.92 | 68.99 | 110.49 | 12.34 | 13.97 | 8.85 | 2.89 | 0.000 | - && W7N2C1.1 RecName: Full=Non-canonical non-ribosomal peptide synthetase FUB8; AltName: Full=Fusaric acid biosynthesis protein 8 && PF07993:Male sterility protein\|PF00501:AMP-binding enzyme |
| A02610 | Contig1 | 4489576 | 4491121 | - | 1221 |  | 3.63 | 4.44 | 4.34 | 61.26 | 88.24 | 59.73 | -4.08 | 0.000 | - && - && PF08450:SMP-30/Gluconolaconase/LRE-like region |
| A02613 | Contig1 | 4516057 | 4539038 | - | 8094 |  | 59.61 | 56.09 | 60.81 | 26.43 | 22.14 | 22.54 | 1.31 | 0.000 | - && Q3L245.1 RecName: Full=Pyranose dehydrogenase 1; Short=PDH 1; AltName: Full=Pyranose:quinone oxidoreductase 1; Flags: Precursor && PF00501:AMP-binding enzyme\|PF05199:GMC oxidoreductase\|PF07993:Male sterility protein\|PF00732:GMC oxidoreductase |
| A02617 | Contig1 | 4551649 | 4554082 | - | 1794 |  | 774.42 | 681.85 | 807.94 | 150.72 | 126.22 | 208.00 | 2.22 | 0.000 | - && Q92341.1 RecName: Full=Siderophore iron transporter 3 && PF07690:Major Facilitator Superfamily |
| A02621 | Contig1 | 4573391 | 4578470 | - | 3144 |  | 147.75 | 148.32 | 179.71 | 316.32 | 475.97 | 468.63 | -1.41 | 0.000 | - && P22292.3 RecName: Full=Mitochondrial 2-oxoglutarate/malate carrier protein; Short=OGCP; AltName: Full=Solute carrier family 25 member 11 && PF04082:Fungal specific transcription factor domain\|PF00153:Mitochondrial carrier protein |
| A02622 | Contig1 | 4583033 | 4584436 | + | 1116 |  | 12.81 | 13.94 | 16.53 | 30.16 | 38.08 | 38.06 | -1.30 | 0.000 | - && P77258.1 RecName: Full=N-ethylmaleimide reductase; AltName: Full=N-ethylmaleimide reducing enzyme && PF00724:NADH:flavin oxidoreductase / NADH oxidase family |
| A02624 | Contig1 | 4594727 | 4596063 | + | 1071 |  | 11.14 | 18.18 | 7.51 | 5.06 | 4.02 | 7.05 | 1.19 | 0.001 | - && Q6Z965.1 RecName: Full=12-oxophytodienoate reductase 7; AltName: Full=12-oxophytodienoate-10,11-reductase 7; Short=OPDA-reductase 7; Short=OsOPR7 && PF00724:NADH:flavin oxidoreductase / NADH oxidase family |
| A02634 | Contig1 | 4624523 | 4625427 | - | 525 |  | 2008.12 | 2114.27 | 1820.38 | 877.08 | 756.73 | 981.73 | 1.18 | 0.000 | - && P56578.1 RecName: Full=Putative peroxiredoxin; AltName: Full=MF2; AltName: Full=Thioredoxin reductase; AltName: Allergen=Mal f 3 && PF08534:Redoxin |
| A02646 | Contig1 | 543068 | 543699 | - | 579 |  | 5.28 | 5.20 | 5.84 | 3.55 | 2.43 | 1.52 | 1.12 | 0.016 | - && - && - |
| A02687 | Contig1 | 693806 | 698055 | - | 2583 |  | 54.36 | 53.98 | 53.93 | 20.05 | 19.82 | 22.87 | 1.37 | 0.000 | - && A8NVW1.2 RecName: Full=Mitochondrial outer membrane protein IML2 && PF10300:Protein of unknown function (DUF3808)\|PF01121:Dephospho-CoA kinase |
| A02689 | Contig1 | 701281 | 701863 | + | 444 |  | 26.21 | 24.64 | 30.04 | 66.12 | 120.18 | 69.57 | -1.66 | 0.000 | - && - && - |
| A02716 | Contig1 | 789575 | 790371 | + | 657 |  | 45.02 | 46.60 | 44.91 | 14.09 | 17.95 | 14.69 | 1.55 | 0.000 | - && Q12040.1 RecName: Full=Broad-specificity phosphatase YOR283W && PF00300:Histidine phosphatase superfamily (branch 1) |
| A02719 | Contig1 | 798039 | 799792 | + | 1287 |  | 23.14 | 21.37 | 18.31 | 11.19 | 13.17 | 6.82 | 1.01 | 0.000 | - && P23262.4 RecName: Full=Salicylate hydroxylase; AltName: Full=Salicylate 1-monooxygenase && PF01494:FAD binding domain |
| A02722 | Contig1 | 810942 | 813760 | + | 1746 |  | 73.87 | 64.34 | 50.02 | 296.66 | 358.20 | 345.25 | -2.41 | 0.000 | - && - && - |
| A02724 | Contig1 | 822767 | 824091 | + | 1023 |  | 0.67 | 2.36 | 1.52 | 0.18 | 0.73 | 0.00 | 2.31 | 0.011 | - && - && - |
| A02730 | Contig1 | 830556 | 832529 | + | 1773 |  | 13.63 | 15.23 | 9.89 | 34.80 | 30.15 | 40.34 | -1.44 | 0.000 | - && Q9P785.1 RecName: Full=LisH domain-containing protein C1711.05 && PF05022:SRP40, C-terminal domain |
| A02734 | Contig1 | 843119 | 844539 | + | 1131 |  | 3.57 | 5.24 | 4.85 | 10.17 | 10.01 | 9.85 | -1.14 | 0.000 | - && Q9V3I5.1 RecName: Full=Chromosomal serine/threonine-protein kinase JIL-1 && PF00069:Protein kinase domain |
| A02745 | Contig1 | 875039 | 876266 | + | 1041 |  | 3.22 | 2.41 | 1.93 | 80.74 | 62.86 | 85.48 | -4.92 | 0.000 | - && Q4WBW4.1 RecName: Full=Probable acetylxylan esterase A; Flags: Precursor && PF10503:Esterase PHB depolymerase |
| A02747 | Contig1 | 878370 | 879505 | + | 1080 |  | 48.11 | 49.64 | 31.80 | 7.27 | 5.20 | 4.79 | 2.91 | 0.000 | - && - && PF13508:Acetyltransferase (GNAT) domain |
| A02748 | Contig1 | 880141 | 881266 | + | 1071 |  | 16.02 | 13.12 | 8.79 | 3.14 | 4.20 | 2.95 | 1.88 | 0.000 | - && - && PF13508:Acetyltransferase (GNAT) domain |
| A02750 | Contig1 | 881485 | 883156 | - | 1617 |  | 2.32 | 3.10 | 1.47 | 3.70 | 2.49 | 13.03 | -1.48 | 0.005 | - && P13860.1 RecName: Full=Exoglucanase 1; AltName: Full=1,4-beta-cellobiohydrolase; AltName: Full=Exocellobiohydrolase I; AltName: Full=Exoglucanase I; Flags: Precursor && PF00840:Glycosyl hydrolase family 7\|PF00734:Fungal cellulose binding domain |
| A02762 | Contig1 | 928955 | 930279 | + | 1194 |  | 4.38 | 2.52 | 2.52 | 7.44 | 6.04 | 9.41 | -1.28 | 0.000 | - && - && - |
| A02764 | Contig1 | 937298 | 940156 | - | 1614 |  | 65.49 | 74.14 | 65.65 | 26.13 | 19.78 | 19.03 | 1.66 | 0.000 | - && O13752.1 RecName: Full=Uncharacterized TLC domain-containing protein C17A2.02c && PF03798:TLC domain |
| A02768 | Contig1 | 947906 | 949035 | - | 903 |  | 22.49 | 23.68 | 19.02 | 42.14 | 41.16 | 60.26 | -1.14 | 0.000 | - && Q08235.2 RecName: Full=Ribosome biogenesis protein BRX1 && PF04427:Brix domain |
| A02771 | Contig1 | 104525 | 107373 | - | 2619 |  | 3.99 | 4.25 | 4.64 | 1.39 | 2.47 | 2.21 | 1.09 | 0.000 | - && - && - |
| A02778 | Contig1 | 976589 | 984133 | - | 4818 |  | 83.40 | 72.51 | 82.97 | 30.00 | 36.60 | 46.81 | 1.07 | 0.000 | - && Q1ECW7.1 RecName: Full=Solute carrier family 25 member 47-A; AltName: Full=Hepatocellular carcinoma down-regulated mitochondrial carrier homolog A && PF07690:Major Facilitator Superfamily\|PF00153:Mitochondrial carrier protein |
| A02785 | Contig1 | 1005352 | 1006762 | - | 1233 |  | 2188.30 | 2617.99 | 969.88 | 284.20 | 257.30 | 538.86 | 2.42 | 0.000 | - && - && - |
| A02789 | Contig1 | 1016905 | 1017951 | - | 1047 |  | 72.14 | 86.10 | 67.88 | 16.97 | 22.26 | 13.91 | 2.09 | 0.000 | - && - && PF08445:FR47-like protein |
| A02796 | Contig1 | 1031802 | 1033683 | + | 1530 |  | 135.66 | 121.91 | 79.53 | 50.29 | 40.81 | 69.12 | 1.07 | 0.000 | - && Q86AW9.1 RecName: Full=Guanine deaminase; Short=Guanase; Short=Guanine aminase; AltName: Full=Guanine aminohydrolase; Short=GAH && PF01979:Amidohydrolase family |
| A02799 | Contig1 | 1041763 | 1042325 | - | 459 |  | 31.79 | 34.12 | 21.49 | 183.53 | 200.07 | 167.30 | -2.66 | 0.000 | - && - && - |
| A02802 | Contig1 | 1049902 | 1051152 | + | 1065 |  | 14.91 | 15.74 | 13.04 | 5.79 | 9.14 | 5.77 | 1.08 | 0.000 | - && P53332.1 RecName: Full=Phosphopantetheine adenylyltransferase; Short=PPAT; AltName: Full=Coenzyme A biosynthesis protein 4; AltName: Full=Dephospho-CoA pyrophosphorylase; AltName: Full=Pantetheine-phosphate adenylyltransferase && PF01467:Cytidylyltransferase-like |
| A02809 | Contig1 | 1073528 | 1075184 | - | 1086 |  | 9.17 | 14.88 | 10.01 | 5.42 | 4.91 | 3.64 | 1.29 | 0.000 | - && A0A0H4LJX8.1 RecName: Full=Secondary metabolism regulator laeA; AltName: Full=Methyltransferase laeA; AltName: Full=Velvet complex subunit laeA && PF13489:Methyltransferase domain |
| A02821 | Contig1 | 1099865 | 1103560 | - | 2682 |  | 39.34 | 69.32 | 64.75 | 298.27 | 284.36 | 361.21 | -2.44 | 0.000 | - && Q4WGT3.1 RecName: Full=Probable beta-glucosidase L; AltName: Full=Beta-D-glucoside glucohydrolase L; AltName: Full=Cellobiase L; AltName: Full=Gentiobiase L; Flags: Precursor && PF14310:Fibronectin type III-like domain\|PF00933:Glycosyl hydrolase family 3 N terminal domain\|PF01915:Glycosyl hydrolase family 3 C-terminal domain |
| A02839 | Contig1 | 1137386 | 1139112 | + | 924 |  | 308.51 | 365.48 | 356.40 | 655.18 | 1387.34 | 789.16 | -1.46 | 0.000 | - && P14766.2 RecName: Full=Fructose-1,6-bisphosphatase, cytosolic; Short=FBPase; AltName: Full=D-fructose-1,6-bisphosphate 1-phosphohydrolase && PF00316:Fructose-1-6-bisphosphatase |
| A02845 | Contig1 | 1151857 | 1154764 | + | 873 |  | 20.78 | 16.79 | 35.05 | 10.17 | 5.25 | 9.15 | 1.56 | 0.000 | - && - && - |
| A02849 | Contig1 | 143148 | 145474 | + | 1677 |  | 2.41 | 1.92 | 2.72 | 6.24 | 4.97 | 5.86 | -1.28 | 0.000 | - && Q0CVX4.2 RecName: Full=Probable alpha-galactosidase D; AltName: Full=Melibiase D; Flags: Precursor && PF16499:Alpha galactosidase A |
| A02860 | Contig1 | 146419 | 147952 | + | 1176 |  | 6.96 | 7.00 | 3.88 | 1.11 | 1.11 | 1.94 | 2.10 | 0.000 | - && P79381.1 RecName: Full=Epoxide hydrolase 1; AltName: Full=Epoxide hydratase; AltName: Full=Microsomal epoxide hydrolase && PF06441:Epoxide hydrolase N terminus |
| A02865 | Contig1 | 1208979 | 1216119 | + | 6378 |  | 42.25 | 44.42 | 40.52 | 78.41 | 102.34 | 95.62 | -1.12 | 0.000 | - && Q9C102.1 RecName: Full=Putative glutamate synthase [NADPH]; AltName: Full=NADPH-GOGAT && PF04898:Glutamate synthase central domain\|PF00310:Glutamine amidotransferases class-II\|PF07992:Pyridine nucleotide-disulphide oxidoreductase\|PF01493:GXGXG motif\|PF14691:Dihydroprymidine dehydrogenase domain II, 4Fe-4S cluster\|PF01645:Conserved region in glutamate synthase |
| A02866 | Contig1 | 1217276 | 1221147 | + | 1716 |  | 24.25 | 26.74 | 18.90 | 12.42 | 11.78 | 8.54 | 1.09 | 0.000 | - && - && - |
| A02892 | Contig1 | 1300473 | 1301393 | - | 720 |  | 46.97 | 49.22 | 63.69 | 17.14 | 29.25 | 24.26 | 1.18 | 0.000 | - && - && PF04930:FUN14 family |
| A02909 | Contig1 | 1354929 | 1356018 | - | 918 |  | 2.79 | 3.39 | 2.69 | 5.50 | 9.08 | 10.23 | -1.48 | 0.000 | - && O74631.1 RecName: Full=Protein FDD123; AltName: Full=CvHSP30/1 && PF01036:Bacteriorhodopsin-like protein |
| A02913 | Contig1 | 1370472 | 1371852 | + | 1008 |  | 53.31 | 58.96 | 51.57 | 149.43 | 132.06 | 143.48 | -1.38 | 0.000 | - && - && - |
| A02917 | Contig1 | 1376685 | 1377641 | + | 711 |  | 1062.03 | 1260.57 | 1185.49 | 15.65 | 14.35 | 9.38 | 6.48 | 0.000 | - && Q00719.1 RecName: Full=O-methyltransferase MdmC && PF01596:O-methyltransferase |
| A02924 | Contig1 | 1398981 | 1401253 | - | 1773 |  | 22.63 | 25.54 | 15.61 | 10.92 | 7.66 | 12.08 | 1.06 | 0.000 | - && Q6IRE4.1 RecName: Full=Tumor susceptibility gene 101 protein; AltName: Full=ESCRT-I complex subunit TSG101 && PF05743:UEV domain\|PF09454:Vps23 core domain |
| A02925 | Contig1 | 1402731 | 1404634 | + | 1698 |  | 182.11 | 199.59 | 133.58 | 75.33 | 46.20 | 77.73 | 1.37 | 0.000 | - && - && PF01285:TEA/ATTS domain family |
| A02929 | Contig1 | 1411742 | 1413283 | - | 1152 |  | 4.71 | 4.10 | 4.12 | 2.03 | 2.93 | 0.84 | 1.16 | 0.009 | - && - && - |
| A02948 | Contig1 | 17289 | 18459 | - | 855 |  | 1.27 | 1.41 | 1.60 | 2.52 | 3.50 | 3.80 | -1.20 | 0.013 | - && - && - |
| A02957 | Contig1 | 1506070 | 1507430 | + | 981 |  | 7.14 | 4.81 | 9.68 | 27.64 | 20.33 | 12.43 | -1.48 | 0.000 | - && - && - |
| A02961 | Contig1 | 1512936 | 1516081 | + | 2004 |  | 217.48 | 227.78 | 241.05 | 76.37 | 92.96 | 120.08 | 1.25 | 0.000 | - && Q9HE13.1 RecName: Full=Uncharacterized MFS-type transporter C1399.02 && PF07690:Major Facilitator Superfamily |
| A02991 | Contig1 | 1611046 | 1612670 | + | 1080 |  | 43.09 | 53.54 | 46.10 | 188.72 | 156.97 | 225.82 | -2.00 | 0.000 | - && Q8NEB5.2 RecName: Full=Phospholipid phosphatase 5; AltName: Full=Phosphatidic acid phosphatase type 2 domain-containing protein 1B && PF01569:PAP2 superfamily |
| A03000 | Contig1 | 1628844 | 1630581 | - | 1389 |  | 6.46 | 7.44 | 8.02 | 15.21 | 16.17 | 15.04 | -1.08 | 0.000 | - && - && PF13926:Domain of unknown function (DUF4211) |
| A03009 | Contig1 | 1671805 | 1672827 | + | 825 |  | 265.09 | 285.35 | 257.44 | 120.35 | 126.40 | 68.19 | 1.36 | 0.000 | - && Q9UTA9.1 RecName: Full=Uncharacterized methyltransferase C25B8.09 && PF08241:Methyltransferase domain |
| A03010 | Contig1 | 1673024 | 1674619 | - | 1221 |  | 167.65 | 171.42 | 75.41 | 7.20 | 4.75 | 9.13 | 4.30 | 0.000 | - && - && - |
| A03012 | Contig1 | 1677429 | 1678585 | - | 831 |  | 1.66 | 3.74 | 0.66 | 0.34 | 0.23 | 0.74 | 2.22 | 0.008 | - && O05220.1 RecName: Full=Uncharacterized protein YwrF && PF01613:Flavin reductase like domain |
| A03016 | Contig1 | 1684057 | 1686109 | + | 1872 |  | 3.32 | 6.38 | 2.78 | 1.50 | 1.05 | 0.80 | 1.90 | 0.000 | - && - && - |
| A03023 | Contig1 | 1704860 | 1706215 | + | 1227 |  | 90.41 | 103.17 | 89.04 | 45.57 | 42.72 | 49.49 | 1.04 | 0.000 | - && P91856.1 RecName: Full=Probable phosphoserine aminotransferase; Short=PSAT; AltName: Full=Phosphohydroxythreonine aminotransferase && PF00266:Aminotransferase class-V |
| A03039 | Contig1 | 1749045 | 1750104 | - | 873 |  | 54.10 | 78.08 | 49.60 | 14.46 | 21.77 | 12.97 | 1.89 | 0.000 | - && O13848.1 RecName: Full=NAD/NADP-dependent indole-3-acetaldehyde reductase; AltName: Full=AKR3C2 && PF00248:Aldo/keto reductase family |
| A03045 | Contig1 | 1766843 | 1768723 | + | 1881 |  | 1.21 | 2.29 | 1.17 | 2.63 | 2.59 | 4.29 | -1.03 | 0.011 | - && - && - |
| A03058 | Contig1 | 1810561 | 1812492 | - | 1650 |  | 201.51 | 163.24 | 213.26 | 35.25 | 29.22 | 51.49 | 2.32 | 0.000 | - && Q51548.2 RecName: Full=L-ornithine N(5)-monooxygenase; AltName: Full=L-ornithine N(5)-hydroxylase; Short=Ornithine hydroxylase; AltName: Full=L-ornithine N(5)-oxygenase; AltName: Full=Pyoverdin biosynthesis protein A && PF13434:L-lysine 6-monooxygenase (NADPH-requiring) |
| A03061 | Contig1 | 1813538 | 1821609 | + | 7365 |  | 52.00 | 45.14 | 50.65 | 21.21 | 22.19 | 21.97 | 1.18 | 0.000 | - && Q4WR82.1 RecName: Full=Nonribosomal peptide synthetase 2; AltName: Full=Siderophore peptide synthase C && PF00550:Phosphopantetheine attachment site\|PF00668:Condensation domain\|PF00501:AMP-binding enzyme |
| A03069 | Contig1 | 1849368 | 1851589 | + | 1803 |  | 93.74 | 94.82 | 61.10 | 21.99 | 18.74 | 27.94 | 1.86 | 0.000 | - && P78753.3 RecName: Full=Probable asparagine synthetase [glutamine-hydrolyzing]; AltName: Full=Glutamine-dependent asparagine synthetase && PF13537:Glutamine amidotransferase domain\|PF00733:Asparagine synthase |
| A03087 | Contig1 | 1908047 | 1910365 | + | 1395 |  | 238.77 | 235.75 | 210.40 | 520.49 | 522.74 | 527.31 | -1.20 | 0.000 | - && Q50I20.1 RecName: Full=2-methylcitrate synthase, mitochondrial; Short=Methylcitrate synthase; AltName: Full=(2S,3S)-2-methylcitrate synthase; AltName: Full=Citrate synthase 1; Flags: Precursor >B0YD89.1 RecName: Full=2-methylcitrate synthase, mitochondrial; Short=Methylcitrate synthase; AltName: Full=(2S,3S)-2-methylcitrate synthase; AltName: Full=Citrate synthase 2; Flags: Precursor && PF00285:Citrate synthase |
| A03088 | Contig1 | 1911136 | 1912823 | - | 1032 |  | 3.34 | 6.23 | 3.89 | 2.90 | 2.36 | 1.19 | 1.06 | 0.016 | - && O14295.1 RecName: Full=Pyridoxal reductase; Short=PL reductase; Short=PL-red && PF00248:Aldo/keto reductase family |
| A03096 | Contig1 | 1928758 | 1930936 | + | 1347 |  | 40.85 | 39.57 | 37.91 | 292.43 | 303.56 | 245.16 | -2.83 | 0.000 | - && - && PF04616:Glycosyl hydrolases family 43 |
| A03140 | Contig1 | 2047956 | 2048897 | - | 822 |  | 116.60 | 190.27 | 135.36 | 913.35 | 784.98 | 1355.45 | -2.79 | 0.000 | - && - && - |
| A03148 | Contig1 | 271621 | 272321 | + | 579 |  | 876.53 | 605.62 | 780.81 | 237.86 | 198.38 | 177.18 | 1.88 | 0.000 | - && - && - |
| A03159 | Contig1 | 273744 | 274430 | + | 582 |  | 26.94 | 21.04 | 15.22 | 4.66 | 6.76 | 2.26 | 2.21 | 0.000 | - && - && - |
| A03170 | Contig1 | 21975 | 24390 | + | 2145 |  | 0.18 | 0.33 | 0.43 | 1.18 | 0.52 | 0.74 | -1.38 | 0.035 | - && Q84LM4.1 RecName: Full=Acylamino-acid-releasing enzyme; Short=AARE; AltName: Full=Oxidized protein hydrolase; Short=OPH && PF00326:Prolyl oligopeptidase family |
| A03172 | Contig1 | 2133583 | 2134716 | + | 903 |  | 22.82 | 21.90 | 24.58 | 44.42 | 44.99 | 54.81 | -1.06 | 0.000 | - && G0S1B3.2 RecName: Full=Protein CMS1 && PF14617:U3-containing 90S pre-ribosomal complex subunit |
| A03180 | Contig1 | 2152999 | 2154012 | - | 720 |  | 7.40 | 12.41 | 6.60 | 70.77 | 346.49 | 16.21 | -4.04 | 0.000 | - && - && - |
| A03182 | Contig1 | 279841 | 281201 | + | 1053 |  | 551.54 | 624.45 | 514.00 | 127.86 | 100.72 | 132.35 | 2.23 | 0.000 | - && Q9P7F4.1 RecName: Full=Zinc-type alcohol dehydrogenase-like protein C2E1P3.01 && PF00107:Zinc-binding dehydrogenase\|PF08240:Alcohol dehydrogenase GroES-like domain |
| A03185 | Contig1 | 2169299 | 2170514 | - | 1101 |  | 1.88 | 1.64 | 1.08 | 0.51 | 0.43 | 0.48 | 1.70 | 0.008 | - && - && - |
| A03189 | Contig1 | 2175149 | 2177331 | - | 1275 |  | 62.95 | 47.56 | 49.94 | 10.93 | 9.25 | 8.26 | 2.50 | 0.000 | - && A0A0B5EMG9.1 RecName: Full=Efflux pump FUBT; AltName: Full=Fusaric acid biosynthesis protein T; AltName: Full=Fusaric acid transporter && PF07690:Major Facilitator Superfamily |
| A03193 | Contig1 | 281859 | 282968 | - | 1056 |  | 183.11 | 190.03 | 109.60 | 55.25 | 21.63 | 70.14 | 1.72 | 0.000 | - && - && PF14604:Variant SH3 domain |
| A03196 | Contig1 | 2190088 | 2190365 | - | 228 |  | 122.39 | 117.12 | 64.51 | 50.03 | 30.79 | 62.35 | 1.09 | 0.001 | - && - && - |
| A03202 | Contig1 | 2222356 | 2227376 | + | 3018 |  | 19.57 | 35.59 | 13.59 | 7.68 | 7.66 | 8.61 | 1.52 | 0.000 | - && Q9URY8.1 RecName: Full=Probable sulfate permease C869.05c && PF01740:STAS domain\|PF00916:Sulfate permease family\|PF01709:Transcriptional regulator |
| A03209 | Contig1 | 2240429 | 2243188 | - | 2094 |  | 23.03 | 20.23 | 21.07 | 8.89 | 13.01 | 7.17 | 1.15 | 0.000 | - && O34313.1 RecName: Full=Trifunctional nucleotide phosphoesterase protein YfkN; Includes: RecName: Full=2',3'-cyclic-nucleotide 2'-phosphodiesterase/3'-nucleotidase; Includes: RecName: Full=5'-nucleotidase; Flags: Precursor && PF02872:5'-nucleotidase, C-terminal domain |
| A03210 | Contig1 | 2243927 | 2246191 | + | 1605 |  | 55.17 | 45.16 | 44.91 | 12.99 | 14.87 | 12.74 | 1.84 | 0.000 | - && Q12627.2 RecName: Full=D-lactate dehydrogenase [cytochrome], mitochondrial; AltName: Full=D-lactate ferricytochrome C oxidoreductase; Short=D-LCR; Flags: Precursor && PF01565:FAD binding domain\|PF02913:FAD linked oxidases, C-terminal domain |
| A03221 | Contig1 | 2270005 | 2272092 | + | 1311 |  | 107.18 | 76.88 | 134.90 | 277.06 | 195.29 | 180.27 | -1.03 | 0.000 | - && Q503M4.1 RecName: Full=Monocarboxylate transporter 12-B; Short=MCT 12-B; AltName: Full=Solute carrier family 16 member 12-B && PF07690:Major Facilitator Superfamily |
| A03233 | Contig1 | 2301275 | 2303513 | - | 1956 |  | 2.12 | 2.67 | 0.56 | 5.93 | 3.69 | 6.82 | -1.62 | 0.000 | - && - && - |
| A03240 | Contig1 | 2318005 | 2318508 | + | 504 |  | 1123.37 | 1266.60 | 879.97 | 442.62 | 383.36 | 629.11 | 1.17 | 0.000 | - && - && - |
| A03251 | Contig1 | 2347440 | 2349012 | - | 1032 |  | 4.68 | 6.13 | 6.46 | 18.39 | 14.51 | 11.65 | -1.37 | 0.000 | - && P30887.2 RecName: Full=Acid phosphatase; Flags: Precursor && PF01975:Survival protein SurE |
| A03255 | Contig1 | 2363760 | 2365973 | + | 1230 |  | 7.86 | 8.81 | 8.69 | 1.52 | 3.27 | 3.35 | 1.64 | 0.000 | - && O64517.1 RecName: Full=Metacaspase-4; Short=AtMC4; AltName: Full=Metacaspase 2d; Short=AtMCP2d; AltName: Full=Metacaspase-7; Contains: RecName: Full=Metacaspase-4 subunit p20; Contains: RecName: Full=Metacaspase-4 subunit p10 && PF00656:Caspase domain |
| A03263 | Contig1 | 2385314 | 2388067 | + | 1752 |  | 584.86 | 563.71 | 50.58 | 21.88 | 24.58 | 21.49 | 4.14 | 0.000 | - && Q2KJG8.1 RecName: Full=[3-methyl-2-oxobutanoate dehydrogenase [lipoamide]] kinase, mitochondrial; AltName: Full=Branched-chain alpha-ketoacid dehydrogenase kinase; Short=BCKD-kinase; Short=BCKDHKIN; Flags: Precursor && PF02518:Histidine kinase-, DNA gyrase B-, and HSP90-like ATPase\|PF10436:Mitochondrial branched-chain alpha-ketoacid dehydrogenase kinase |
| A03279 | Contig1 | 2440609 | 2448893 | + | 5883 |  | 298.16 | 193.98 | 497.32 | 89.32 | 136.97 | 61.71 | 1.78 | 0.000 | - && O94123.1 RecName: Full=Phosphoglycerate kinase && PF06201:PITH domain\|PF00162:Phosphoglycerate kinase\|PF08238:Sel1 repeat |
| A03281 | Contig1 | 24613 | 28056 | - | 2730 |  | 6.32 | 5.88 | 14.05 | 21.99 | 10.01 | 24.95 | -1.12 | 0.003 | - && - && PF04082:Fungal specific transcription factor domain |
| A03283 | Contig1 | 2451470 | 2453022 | + | 1389 |  | 92.78 | 88.17 | 58.20 | 26.39 | 34.44 | 39.36 | 1.26 | 0.000 | - && - && - |
| A03298 | Contig1 | 2562818 | 2564208 | + | 1206 |  | 17.74 | 20.06 | 12.35 | 3.72 | 3.88 | 3.27 | 2.20 | 0.000 | - && - && PF00781:Diacylglycerol kinase catalytic domain |
| A03303 | Contig1 | 2576978 | 2578164 | + | 1047 |  | 17.52 | 14.19 | 19.72 | 38.58 | 44.17 | 37.13 | -1.22 | 0.000 | - && - && - |
| A03309 | Contig1 | 2593799 | 2595170 | + | 1071 |  | 5.80 | 6.28 | 4.95 | 2.44 | 3.50 | 2.38 | 1.03 | 0.003 | - && Q6ZXC1.2 RecName: Full=Probable inactive dehydrogenase EasA; AltName: Full=Ergot alkaloid biosynthesis protein A [Claviceps purpurea] && PF00724:NADH:flavin oxidoreductase / NADH oxidase family |
| A03312 | Contig1 | 2607495 | 2609595 | - | 1035 |  | 72.50 | 49.66 | 149.43 | 178.23 | 246.10 | 128.71 | -1.03 | 0.004 | - && - && - |
| A03314 | Contig1 | 2613887 | 2614303 | - | 309 |  | 13.08 | 7.80 | 12.42 | 5.45 | 3.33 | 5.11 | 1.26 | 0.006 | - && - && - |
| A03321 | Contig1 | 2622427 | 2623607 | - | 738 |  | 91.39 | 106.24 | 84.67 | 187.88 | 210.43 | 223.20 | -1.14 | 0.000 | - && P40915.1 RecName: Full=NADH-ubiquinone oxidoreductase 24 kDa subunit, mitochondrial; Flags: Precursor && PF01257:Thioredoxin-like [2Fe-2S] ferredoxin |
| A03323 | Contig1 | 2629669 | 2630652 | - | 678 |  | 40.87 | 39.83 | 40.29 | 103.70 | 162.64 | 47.89 | -1.38 | 0.000 | - && Q9Y7Q2.1 RecName: Full=Glutathione S-transferase 1; AltName: Full=GST-I && PF02798:Glutathione S-transferase, N-terminal domain\|PF00043:Glutathione S-transferase, C-terminal domain |
| A03333 | Contig1 | 2654026 | 2656177 | - | 1668 |  | 7.86 | 9.27 | 8.21 | 4.88 | 3.70 | 2.95 | 1.14 | 0.000 | - && - && PF13489:Methyltransferase domain |
| A03339 | Contig1 | 2681365 | 2682694 | - | 507 |  | 8.75 | 9.50 | 10.45 | 3.32 | 3.88 | 1.04 | 1.80 | 0.000 | - && - && - |
| A03343 | Contig1 | 2691563 | 2692573 | - | 783 |  | 30.10 | 28.98 | 19.95 | 10.99 | 12.19 | 8.97 | 1.30 | 0.000 | - && Q503L4.2 RecName: Full=Isoamyl acetate-hydrolyzing esterase 1 homolog && PF00657:GDSL-like Lipase/Acylhydrolase |
| A03357 | Contig1 | 2740098 | 2743590 | - | 2325 |  | 31.77 | 32.86 | 32.65 | 75.72 | 52.46 | 89.38 | -1.16 | 0.000 | - && P53326.1 RecName: Full=Uncharacterized protein YGR266W && - |
| A03364 | Contig1 | 2758195 | 2759044 | + | 675 |  | 96.41 | 108.42 | 104.34 | 247.66 | 227.30 | 202.04 | -1.13 | 0.000 | - && - && PF08229:ER membrane protein SH3 |
| A03365 | Contig1 | 2759817 | 2764208 | + | 2031 |  | 139.00 | 122.33 | 113.07 | 31.99 | 37.93 | 22.04 | 2.03 | 0.000 | - && - && PF13649:Methyltransferase domain\|PF04191:Phospholipid methyltransferase |
| A03369 | Contig1 | 2777504 | 2786066 | + | 3429 |  | 12.80 | 9.95 | 12.07 | 4.64 | 5.30 | 4.45 | 1.28 | 0.000 | - && Q10088.1 RecName: Full=Putative agmatinase 1; AltName: Full=Agmatine ureohydrolase 1; Short=AUH 1; Flags: Precursor && PF00491:Arginase family |
| A03373 | Contig1 | 2794255 | 2795013 | + | 759 |  | 0.39 | 0.79 | 0.60 | 3.45 | 1.11 | 0.92 | -1.62 | 0.042 | - && - && - |
| A03378 | Contig1 | 2821077 | 2824701 | - | 2907 |  | 27.17 | 22.52 | 31.14 | 8.75 | 11.85 | 6.73 | 1.56 | 0.000 | - && A0A0D2YG01.1 RecName: Full=Non-canonical non-ribosomal peptide synthetase FUB8; AltName: Full=Fusaric acid biosynthesis protein 8 && PF07993:Male sterility protein\|PF00501:AMP-binding enzyme |
| A03385 | Contig1 | 2841940 | 2843052 | - | 882 |  | 8.38 | 8.76 | 6.32 | 27.67 | 24.20 | 72.14 | -2.40 | 0.000 | - && - && - |
| A03386 | Contig1 | 2844196 | 2845862 | + | 1443 |  | 3.28 | 4.31 | 3.67 | 8.75 | 21.73 | 32.11 | -2.47 | 0.000 | - && - && - |
| A03388 | Contig1 | 2859386 | 2860021 | - | 447 |  | 0.88 | 4.27 | 2.04 | 4.18 | 6.70 | 14.72 | -1.83 | 0.003 | - && - && PF16850:Peptidase inhibitor I66 |
| A03394 | Contig1 | 2876810 | 2878368 | + | 1263 |  | 575.15 | 385.42 | 573.49 | 40.27 | 44.32 | 30.02 | 3.74 | 0.000 | - && O60020.1 RecName: Full=Aspartic protease; Flags: Precursor && PF00026:Eukaryotic aspartyl protease |
| A03397 | Contig1 | 2884183 | 2886664 | - | 1920 |  | 103.89 | 125.43 | 96.58 | 200.33 | 237.44 | 340.80 | -1.26 | 0.000 | - && Q9UTJ7.1 RecName: Full=Probable succinate dehydrogenase [ubiquinone] flavoprotein subunit, mitochondrial; AltName: Full=Flavoprotein subunit of complex II; Short=FP; Flags: Precursor && PF00890:FAD binding domain\|PF02910:Fumarate reductase flavoprotein C-term |
| A03404 | Contig1 | 333217 | 348530 | - | 11007 |  | 638.53 | 340.50 | 955.12 | 159.76 | 289.54 | 80.18 | 1.87 | 0.003 | - && - && PF12359:Protein of unknown function (DUF3645)\|PF12340:Protein of unknown function (DUF3638) |
| A03417 | Contig1 | 2956637 | 2966618 | - | 2391 |  | 2.68 | 4.03 | 3.40 | 1.17 | 0.20 | 0.73 | 2.27 | 0.000 | - && Q9D187.1 RecName: Full=Mitotic spindle-associated MMXD complex subunit MIP18; AltName: Full=Protein FAM96B && PF03732:Retrotransposon gag protein |
| A03421 | Contig1 | 2982778 | 2986819 | - | 2055 |  | 111.94 | 94.18 | 104.51 | 52.73 | 64.00 | 38.35 | 1.00 | 0.000 | - && - && PF12697:Alpha/beta hydrolase family |
| A03426 | Contig1 | 351900 | 353551 | - | 1395 |  | 86.37 | 93.62 | 66.53 | 29.82 | 27.78 | 47.06 | 1.24 | 0.000 | - && - && PF00134:Cyclin, N-terminal domain |
| A03451 | Contig1 | 3100784 | 3103460 | + | 1800 |  | 8.22 | 5.58 | 5.94 | 1.77 | 1.40 | 1.85 | 1.97 | 0.000 | - && D4B093.1 RecName: Full=Secreted protein ARB_01864; Flags: Precursor && - |
| A03458 | Contig1 | 3117498 | 3119890 | - | 1779 |  | 1.22 | 2.37 | 3.54 | 1.26 | 0.89 | 0.15 | 1.63 | 0.007 | - && Q9Y758.1 RecName: Full=Cytochrome P450 52A13; AltName: Full=Alkane hydroxylase 2; AltName: Full=Alkane-inducible p450alk 2; AltName: Full=DH-ALK2 [Debaryomyces hansenii] && PF00067:Cytochrome P450 |
| A03460 | Contig1 | 3123174 | 3125409 | - | 1506 |  | 0.59 | 0.73 | 0.36 | 33.52 | 45.62 | 16.67 | -5.83 | 0.000 | - && P09437.2 RecName: Full=Cytochrome b2, mitochondrial; AltName: Full=L-lactate dehydrogenase [Cytochrome]; AltName: Full=L-lactate ferricytochrome C oxidoreductase; Short=L-LCR; Flags: Precursor && PF01070:FMN-dependent dehydrogenase\|PF00173:Cytochrome b5-like Heme/Steroid binding domain |
| A03461 | Contig1 | 3127675 | 3132234 | + | 2454 |  | 5.95 | 11.74 | 5.99 | 53.42 | 59.51 | 33.58 | -2.63 | 0.000 | - && P09437.2 RecName: Full=Cytochrome b2, mitochondrial; AltName: Full=L-lactate dehydrogenase [Cytochrome]; AltName: Full=L-lactate ferricytochrome C oxidoreductase; Short=L-LCR; Flags: Precursor && PF01070:FMN-dependent dehydrogenase\|PF00173:Cytochrome b5-like Heme/Steroid binding domain\|PF13714:Phosphoenolpyruvate phosphomutase |
| A03465 | Contig1 | 3145967 | 3147553 | - | 1023 |  | 4.05 | 4.71 | 2.32 | 2.10 | 1.56 | 0.86 | 1.30 | 0.007 | - && - && - |
| A03487 | Contig1 | 3222993 | 3226449 | + | 2451 |  | 10.78 | 11.10 | 8.91 | 29.94 | 23.37 | 33.51 | -1.50 | 0.000 | - && P10901.1 RecName: Full=Alpha-L-fucosidase; AltName: Full=Alpha-L-fucoside fucohydrolase; Flags: Precursor && PF01120:Alpha-L-fucosidase\|PF16757:Alpha-L-fucosidase C-terminal domain |
| A03489 | Contig1 | 3229472 | 3230347 | + | 639 |  | 72.37 | 110.29 | 27.30 | 7.61 | 3.66 | 7.83 | 3.46 | 0.000 | - && O59827.1 RecName: Full=Glutathione S-transferase 2; AltName: Full=GST-II && PF00043:Glutathione S-transferase, C-terminal domain\|PF02798:Glutathione S-transferase, N-terminal domain |
| A03490 | Contig1 | 3231286 | 3232041 | + | 756 |  | 7.56 | 7.97 | 4.23 | 1.11 | 2.72 | 2.55 | 1.63 | 0.000 | - && - && - |
| A03503 | Contig1 | 32344 | 33907 | + | 1005 |  | 0.10 | 0.70 | 1.00 | 2.98 | 5.59 | 2.36 | -2.60 | 0.000 | - && - && PF13472:GDSL-like Lipase/Acylhydrolase family |
| A03515 | Contig1 | 385550 | 387401 | + | 1338 |  | 5.90 | 6.60 | 4.85 | 3.00 | 2.94 | 2.43 | 1.05 | 0.001 | - && B4S3I3.1 RecName: Full=Probable endonuclease 4; AltName: Full=Endodeoxyribonuclease IV; AltName: Full=Endonuclease IV && PF01261:Xylose isomerase-like TIM barrel |
| A03518 | Contig1 | 3362237 | 3363199 | + | 963 |  | 29.49 | 35.34 | 31.02 | 72.91 | 65.03 | 75.64 | -1.16 | 0.000 | - && - && - |
| A03523 | Contig1 | 3378406 | 3379023 | - | 381 |  | 396.22 | 497.20 | 591.50 | 2452.24 | 2990.86 | 1680.31 | -2.26 | 0.000 | - && - && - |
| A03532 | Contig1 | 3416459 | 3418104 | - | 1407 |  | 1.96 | 0.64 | 3.12 | 0.93 | 0.53 | 0.37 | 1.64 | 0.011 | - && - && - |
| A03535 | Contig1 | 3423153 | 3424910 | - | 1068 |  | 18.56 | 17.86 | 20.01 | 30.46 | 52.94 | 41.83 | -1.15 | 0.000 | - && G2QJ27.1 RecName: Full=Acetylesterase; AltName: Full=Carbohydrate esterase family 16 protein; Flags: Precursor && PF00734:Fungal cellulose binding domain |
| A03544 | Contig1 | 3456754 | 3459033 | + | 2217 |  | 0.18 | 0.32 | 0.08 | 0.93 | 0.59 | 0.55 | -1.84 | 0.011 | - && - && PF03221:Tc5 transposase DNA-binding domain\|PF03184:DDE superfamily endonuclease |
| A03546 | Contig1 | 3473753 | 3475784 | - | 1440 |  | 4.04 | 3.56 | 3.24 | 6.95 | 8.52 | 6.22 | -1.00 | 0.000 | - && - && PF04616:Glycosyl hydrolases family 43 |
| A03560 | Contig1 | 3532673 | 3533200 | + | 528 |  | 6.16 | 6.08 | 9.69 | 16.65 | 15.25 | 18.12 | -1.19 | 0.000 | - && - && - |
| A03566 | Contig1 | 3545809 | 3549049 | + | 1995 |  | 113.23 | 146.13 | 100.74 | 423.29 | 601.22 | 520.31 | -2.10 | 0.000 | - && P33303.2 RecName: Full=Succinate/fumarate mitochondrial transporter; AltName: Full=Regulator of acetyl-CoA synthase activity && PF00153:Mitochondrial carrier protein |
| A03576 | Contig1 | 3584684 | 3585080 | - | 219 |  | 107.16 | 108.64 | 115.54 | 234.81 | 261.60 | 171.51 | -1.01 | 0.000 | - && - && - |
| A03581 | Contig1 | 414558 | 415837 | + | 1041 |  | 5.97 | 24.69 | 6.05 | 73.65 | 90.73 | 44.68 | -2.51 | 0.001 | - && - && PF11911:Protein of unknown function (DUF3429) |
| A03583 | Contig1 | 3600051 | 3600546 | + | 369 |  | 1.60 | 2.18 | 2.72 | 14.70 | 15.98 | 11.42 | -2.69 | 0.000 | - && - && - |
| A03585 | Contig1 | 3607667 | 3609377 | + | 1533 |  | 264.10 | 169.54 | 238.18 | 51.78 | 72.60 | 57.59 | 1.88 | 0.000 | - && - && - |
| A03593 | Contig1 | 3623202 | 3624556 | + | 1044 |  | 5.19 | 4.23 | 3.24 | 48.45 | 81.15 | 75.07 | -4.01 | 0.000 | - && - && PF10282:Lactonase, 7-bladed beta-propeller |
| A03594 | Contig1 | 3626133 | 3627403 | + | 1071 |  | 218.75 | 242.21 | 272.26 | 474.72 | 586.05 | 568.59 | -1.15 | 0.000 | - && - && PF10282:Lactonase, 7-bladed beta-propeller |
| A03602 | Contig1 | 3652967 | 3653481 | - | 405 |  | 480.84 | 734.69 | 874.03 | 4010.39 | 5076.92 | 3222.58 | -2.56 | 0.000 | - && P04158.2 RecName: Full=Fruiting body protein SC1; AltName: Full=Hydrophobin SC1; Flags: Precursor && PF01185:Fungal hydrophobin |
| A03604 | Contig1 | 3655497 | 3655958 | + | 345 |  | 584.76 | 743.16 | 1095.93 | 4509.75 | 5735.20 | 3544.68 | -2.51 | 0.000 | - && P04158.2 RecName: Full=Fruiting body protein SC1; AltName: Full=Hydrophobin SC1; Flags: Precursor && PF01185:Fungal hydrophobin |
| A03607 | Contig1 | 3665617 | 3668174 | + | 807 |  | 1375.68 | 2066.23 | 2467.80 | 4457.21 | 5915.32 | 4260.26 | -1.31 | 0.000 | - && P16933.2 RecName: Full=Fruiting body protein SC3; AltName: Full=Hydrophobin SC3; Flags: Precursor && PF01185:Fungal hydrophobin |
| A03610 | Contig1 | 3676253 | 3677780 | - | 1212 |  | 5.37 | 6.38 | 4.45 | 19.21 | 16.37 | 20.13 | -1.78 | 0.000 | - && - && PF07470:Glycosyl Hydrolase Family 88 |
| A03625 | Contig20 | 398372 | 399457 | - | 1086 |  | 3.99 | 3.61 | 1.93 | 0.86 | 0.43 | 0.65 | 2.30 | 0.000 | - && - && - |
| A03627 | Contig20 | 403208 | 408484 | + | 3510 |  | 6.32 | 4.23 | 5.07 | 2.64 | 2.37 | 2.70 | 1.02 | 0.000 | - && - && - |
| A03643 | Contig20 | 457347 | 459833 | - | 2022 |  | 1.17 | 0.94 | 0.81 | 0.23 | 0.19 | 0.35 | 1.94 | 0.002 | - && - && - |
| A03646 | Contig20 | 465900 | 467620 | + | 1368 |  | 143.15 | 133.63 | 148.38 | 49.89 | 49.54 | 83.97 | 1.21 | 0.000 | - && Q6URB0.1 RecName: Full=Cytochrome c peroxidase, mitochondrial; Short=CCP; Flags: Precursor && PF00141:Peroxidase |
| A03649 | Contig20 | 470635 | 473309 | + | 2301 |  | 5.79 | 5.37 | 2.74 | 0.69 | 0.49 | 0.50 | 3.05 | 0.000 | - && - && - |
| A03652 | Contig20 | 481760 | 485857 | + | 2322 |  | 2.93 | 3.07 | 3.15 | 0.89 | 0.56 | 1.21 | 1.78 | 0.000 | - && - && - |
| A03659 | Contig20 | 39107 | 40386 | + | 1104 |  | 15.09 | 14.19 | 12.99 | 36.50 | 30.36 | 37.60 | -1.31 | 0.000 | - && - && PF03876:SHS2 domain found in N terminus of Rpb7p/Rpc25p/MJ0397 |
| A03665 | Contig20 | 56411 | 58048 | + | 1434 |  | 53.29 | 50.47 | 43.19 | 15.00 | 9.79 | 14.26 | 1.91 | 0.000 | - && - && PF00067:Cytochrome P450 |
| A03673 | Contig20 | 74465 | 75346 | + | 882 |  | 5.81 | 6.03 | 7.56 | 13.57 | 15.60 | 11.24 | -1.06 | 0.000 | - && - && - |
| A03679 | Contig20 | 89997 | 92113 | + | 1542 |  | 30.12 | 24.74 | 23.58 | 9.34 | 9.53 | 9.79 | 1.45 | 0.000 | - && F8WKW1.1 RecName: Full=7-deoxyloganetin glucosyltransferase; AltName: Full=Genipin glucosyltransferase; AltName: Full=UDP-glucose glucosyltransferase 2; Short=GjUGT2; AltName: Full=UDP-glycosyltransferase 85A24 && PF00201:UDP-glucoronosyl and UDP-glucosyl transferase |
| A03683 | Contig20 | 103277 | 107888 | - | 2772 |  | 87.18 | 123.10 | 87.13 | 1326.55 | 3086.24 | 2079.77 | -4.45 | 0.000 | - && P28344.3 RecName: Full=Malate synthase, glyoxysomal && PF01274:Malate synthase\|PF04420:CHD5-like protein |
| A03688 | Contig20 | 11750 | 12654 | - | 738 |  | 1.20 | 1.36 | 1.49 | 0.51 | 0.25 | 0.36 | 1.86 | 0.026 | - && - && - |
| A03690 | Contig20 | 120714 | 121651 | + | 813 |  | 4.85 | 3.21 | 3.37 | 1.27 | 0.69 | 1.08 | 1.91 | 0.000 | - && - && - |
| A03711 | Contig20 | 201985 | 202640 | + | 387 |  | 2.04 | 2.33 | 3.78 | 1.21 | 0.73 | 0.68 | 1.64 | 0.040 | - && - && PF16850:Peptidase inhibitor I66 |
| A03712 | Contig20 | 202742 | 204880 | - | 1743 |  | 5.54 | 10.71 | 6.50 | 124.71 | 170.14 | 166.66 | -4.34 | 0.000 | - && D8Q9M3.1 RecName: Full=Glucoamylase ARB_02327-1; AltName: Full=1,4-alpha-D-glucan glucohydrolase; AltName: Full=Glucan 1,4-alpha-glucosidase; AltName: Allergen=Sch c 1; Flags: Precursor && PF00686:Starch binding domain\|PF00723:Glycosyl hydrolases family 15 |
| A03720 | Contig20 | 256022 | 259864 | + | 1650 |  | 3.05 | 3.22 | 1.72 | 5.33 | 7.89 | 8.67 | -1.45 | 0.000 | - && Q92275.2 RecName: Full=Pheromone B alpha 1 receptor [Schizophyllum commune] && PF02076:Pheromone A receptor |
| A03726 | Contig20 | 274720 | 276317 | - | 1086 |  | 90.16 | 123.13 | 71.67 | 21.44 | 32.15 | 33.37 | 1.71 | 0.000 | - && P50867.2 RecName: Full=Cysteine synthase 1; Short=CS 1; AltName: Full=O-acetylserine (thiol)-lyase 1; Short=OAS-TL 1; AltName: Full=O-acetylserine sulfhydrylase 1; Flags: Precursor && PF00291:Pyridoxal-phosphate dependent enzyme |
| A03731 | Contig20 | 286763 | 287552 | - | 501 |  | 68.29 | 78.15 | 22.61 | 23.89 | 17.19 | 39.76 | 1.06 | 0.009 | - && - && - |
| A03739 | Contig20 | 306009 | 310285 | - | 2811 |  | 54.69 | 99.78 | 36.49 | 5.89 | 4.63 | 9.55 | 3.25 | 0.000 | - && P37959.2 RecName: Full=Uncharacterized oxidoreductase YusZ; AltName: Full=ORFA && PF00106:short chain dehydrogenase\|PF00656:Caspase domain |
| A03741 | Contig20 | 313821 | 316757 | + | 2046 |  | 130.84 | 132.48 | 144.17 | 58.13 | 57.92 | 62.80 | 1.19 | 0.000 | - && Q09768.1 RecName: Full=Glutamate--cysteine ligase; AltName: Full=Gamma-ECS; Short=GCS; AltName: Full=Gamma-glutamylcysteine synthetase && PF03074:Glutamate-cysteine ligase |
| A03745 | Contig20 | 323324 | 324374 | - | 945 |  | 11.16 | 8.92 | 8.51 | 20.68 | 18.42 | 21.36 | -1.08 | 0.000 | - && - && - |
| A03746 | Contig20 | 325139 | 325909 | + | 543 |  | 38.32 | 49.73 | 50.30 | 202.83 | 97.23 | 122.83 | -1.61 | 0.000 | - && - && - |
| A03747 | Contig20 | 327102 | 328011 | + | 855 |  | 63.54 | 49.31 | 77.03 | 23.51 | 54.52 | 12.62 | 1.07 | 0.008 | - && - && - |
| A03752 | Contig20 | 354246 | 356134 | - | 1665 |  | 21.14 | 22.19 | 22.77 | 9.55 | 8.77 | 8.33 | 1.31 | 0.000 | - && A0A0B5EMG9.1 RecName: Full=Efflux pump FUBT; AltName: Full=Fusaric acid biosynthesis protein T; AltName: Full=Fusaric acid transporter && PF07690:Major Facilitator Superfamily |
| A03769 | Contig21 | 93343 | 95804 | - | 2238 |  | 0.53 | 0.09 | 0.29 | 0.96 | 1.00 | 0.82 | -1.62 | 0.011 | - && - && - |
| A03773 | Contig21 | 125304 | 127761 | - | 2232 |  | 5.17 | 3.64 | 5.77 | 10.26 | 11.32 | 9.04 | -1.07 | 0.000 | - && - && - |
| A03793 | Contig21 | 243606 | 245449 | + | 1581 |  | 4.68 | 0.95 | 4.85 | 1.42 | 1.72 | 1.00 | 1.34 | 0.012 | - && P37906.2 RecName: Full=Gamma-glutamylputrescine oxidoreductase; Short=Gamma-Glu-Put oxidase; Short=Gamma-glutamylputrescine oxidase && PF01266:FAD dependent oxidoreductase |
| A03794 | Contig21 | 245756 | 247624 | + | 1521 |  | 4.28 | 4.09 | 3.54 | 1.35 | 1.72 | 1.56 | 1.36 | 0.000 | - && - && PF01266:FAD dependent oxidoreductase |
| A03801 | Contig21 | 282871 | 284924 | - | 1665 |  | 1.78 | 2.17 | 1.48 | 12.24 | 2.81 | 7.85 | -2.08 | 0.000 | - && Q00024.1 RecName: Full=Polyphenol oxidase 1; Short=PPO1; Short=Phenolase 1; AltName: Full=Cresolase 1; AltName: Full=Tyrosinase 1; Flags: Precursor && PF00264:Common central domain of tyrosinase |
| A03818 | Contig21 | 339963 | 340686 | + | 591 |  | 13.18 | 17.33 | 10.20 | 9.65 | 7.13 | 3.27 | 1.02 | 0.011 | - && - && - |
| A03819 | Contig21 | 341135 | 343843 | - | 2601 |  | 56.83 | 41.72 | 70.98 | 24.69 | 25.12 | 27.23 | 1.14 | 0.000 | - && P40566.1 RecName: Full=Uncharacterized glycosyl hydrolase YIR007W && PF00150:Cellulase (glycosyl hydrolase family 5) |
| A03820 | Contig21 | 344629 | 345432 | + | 699 |  | 14.67 | 8.33 | 13.07 | 3.88 | 7.50 | 4.52 | 1.18 | 0.001 | - && - && - |
| A03821 | Contig21 | 345672 | 347913 | - | 2058 |  | 5.94 | 7.61 | 8.43 | 20.26 | 15.01 | 15.78 | -1.22 | 0.000 | - && - && - |
| A03832 | Contig21 | 377457 | 378700 | + | 1071 |  | 102.19 | 138.63 | 58.68 | 9.78 | 8.65 | 11.80 | 3.31 | 0.000 | - && - && - |
| A03833 | Contig21 | 378946 | 379866 | - | 819 |  | 1.69 | 2.33 | 2.12 | 6.51 | 6.52 | 3.00 | -1.39 | 0.002 | - && - && PF00651:BTB/POZ domain |
| A03837 | Contig21 | 389717 | 390386 | - | 480 |  | 714.26 | 349.48 | 725.47 | 236.27 | 180.79 | 95.62 | 1.80 | 0.000 | - && - && PF10467:Peptidase inhibitor clitocypin |
| A03883 | Contig22 | 127268 | 128531 | - | 885 |  | 29.86 | 37.89 | 25.81 | 10.88 | 14.28 | 15.27 | 1.21 | 0.000 | - && P23641.1 RecName: Full=Mitochondrial phosphate carrier protein; AltName: Full=Mitochondrial import receptor; AltName: Full=Phosphate transport protein; Short=PTP; AltName: Full=mPic 1; AltName: Full=p32; Contains: RecName: Full=Mitochondrial phosphate carrier protein, N-terminally processed && PF00153:Mitochondrial carrier protein |
| A03885 | Contig22 | 135380 | 140228 | - | 2262 |  | 10.42 | 10.21 | 9.73 | 21.95 | 24.33 | 21.80 | -1.17 | 0.000 | - && A1C4H2.1 RecName: Full=Probable endo-beta-1,4-glucanase D; Short=Endoglucanase D; AltName: Full=Carboxymethylcellulase D; AltName: Full=Cellulase D; Flags: Precursor && PF00734:Fungal cellulose binding domain\|PF03443:Glycosyl hydrolase family 61\|PF07200:Modifier of rudimentary (Mod(r)) protein |
| A03898 | Contig22 | 171116 | 175788 | - | 2727 |  | 1349.82 | 644.37 | 1643.83 | 443.48 | 785.41 | 272.83 | 1.28 | 0.001 | - && Q9P778.1 RecName: Full=Dipeptidyl-peptidase 5; AltName: Full=Dipeptidyl-peptidase V; Short=DPP V; Short=DppV; Flags: Precursor && PF00326:Prolyl oligopeptidase family |
| A03902 | Contig22 | 181628 | 183428 | - | 1563 |  | 4.29 | 2.76 | 3.27 | 0.66 | 0.36 | 0.62 | 2.66 | 0.000 | - && Q0CJ61.1 RecName: Full=Efflux pump atB; AltName: Full=Terreic acid biosynthesis cluster protein B && PF07690:Major Facilitator Superfamily |
| A03903 | Contig22 | 184927 | 186406 | - | 912 |  | 59.68 | 54.16 | 81.63 | 31.78 | 42.08 | 23.00 | 1.01 | 0.000 | - && P06632.3 RecName: Full=2,5-diketo-D-gluconic acid reductase A; Short=2,5-DKG reductase A; Short=2,5-DKGR A; Short=25DKGR-A; AltName: Full=AKR5C && PF00248:Aldo/keto reductase family |
| A03904 | Contig22 | 187139 | 188664 | + | 1359 |  | 84.53 | 80.52 | 87.59 | 37.70 | 30.72 | 33.71 | 1.31 | 0.000 | - && - && - |
| A03911 | Contig22 | 222156 | 227899 | + | 3729 |  | 290.31 | 240.08 | 359.89 | 25.35 | 19.10 | 17.18 | 3.85 | 0.000 | - && P17576.1 RecName: Full=Polyporopepsin; AltName: Full=Aspartic proteinase && PF08170:POPLD (NUC188) domain\|PF06978:Ribonucleases P/MRP protein subunit POP1\|PF00026:Eukaryotic aspartyl protease |
| A03912 | Contig22 | 228744 | 230575 | + | 1608 |  | 83.82 | 58.37 | 67.43 | 9.54 | 6.75 | 5.95 | 3.24 | 0.000 | - && A0A0D2YFZ8.1 RecName: Full=Efflux pump FUB11; AltName: Full=Fusaric acid biosynthesis protein 11 && PF07690:Major Facilitator Superfamily |
| A03913 | Contig22 | 232220 | 233137 | + | 840 |  | 3.52 | 2.87 | 2.17 | 0.78 | 0.89 | 1.99 | 1.23 | 0.026 | - && - && - |
| A03917 | Contig22 | 243650 | 246539 | + | 1806 |  | 25.17 | 25.07 | 41.12 | 125.49 | 99.73 | 141.21 | -2.00 | 0.000 | - && - && PF02055:O-Glycosyl hydrolase family 30 |
| A03929 | Contig22 | 285077 | 288585 | + | 2901 |  | 31.81 | 35.57 | 30.64 | 21.21 | 14.71 | 12.46 | 1.02 | 0.000 | - && Q9P7I1.2 RecName: Full=K(+)/H(+) antiporter 1 && PF00999:Sodium/hydrogen exchanger family |
| A03930 | Contig22 | 290626 | 292119 | - | 1269 |  | 23.78 | 23.81 | 20.95 | 46.05 | 46.33 | 56.85 | -1.12 | 0.000 | - && P36049.1 RecName: Full=rRNA-processing protein EBP2; AltName: Full=EBNA1-binding protein homolog && PF05890:Eukaryotic rRNA processing protein EBP2 |
| A03934 | Contig22 | 301486 | 302269 | - | 729 |  | 2.30 | 2.89 | 2.63 | 8.46 | 10.91 | 2.53 | -1.49 | 0.004 | - && - && PF04140:Isoprenylcysteine carboxyl methyltransferase (ICMT) family |
| A03937 | Contig22 | 317160 | 322844 | + | 2835 |  | 14.89 | 13.81 | 19.30 | 8.51 | 7.83 | 6.10 | 1.10 | 0.000 | - && Q9C101.1 RecName: Full=Uncharacterized MFS-type transporter PB1E7.08c && PF00083:Sugar (and other) transporter\|PF07690:Major Facilitator Superfamily |
| A03939 | Contig22 | 329526 | 330808 | + | 969 |  | 116.21 | 152.71 | 140.47 | 298.82 | 290.40 | 403.02 | -1.28 | 0.000 | - && - && PF04479:RTA1 like protein |
| A03941 | Contig22 | 336113 | 337423 | + | 993 |  | 44.98 | 61.77 | 63.20 | 24.95 | 14.89 | 27.49 | 1.34 | 0.000 | - && P40113.1 RecName: Full=Protein RTM1 && PF04479:RTA1 like protein |
| A03947 | Contig22 | 360134 | 366112 | + | 3087 |  | 56.70 | 45.50 | 66.32 | 18.69 | 22.02 | 17.51 | 1.53 | 0.000 | - && Q01772.1 RecName: Full=Aldehyde oxidase GLOX; AltName: Full=Glyoxal oxidase; Short=GLOX; Flags: Precursor && PF00155:Aminotransferase class I and II\|PF07250:Glyoxal oxidase N-terminus\|PF09118:Domain of unknown function (DUF1929) |
| A03948 | Contig22 | 367104 | 370348 | + | 2157 |  | 8.96 | 7.21 | 9.40 | 43.48 | 76.51 | 121.12 | -3.24 | 0.000 | - && - && - |
| A03949 | Contig22 | 370824 | 372779 | - | 1671 |  | 4.31 | 3.06 | 3.99 | 69.38 | 196.30 | 23.37 | -4.67 | 0.000 | - && Q01772.1 RecName: Full=Aldehyde oxidase GLOX; AltName: Full=Glyoxal oxidase; Short=GLOX; Flags: Precursor && PF09118:Domain of unknown function (DUF1929)\|PF07250:Glyoxal oxidase N-terminus |
| A03952 | Contig22 | 377447 | 379035 | - | 1200 |  | 5.01 | 6.53 | 5.18 | 16.44 | 17.86 | 15.07 | -1.56 | 0.000 | - && Q10088.1 RecName: Full=Putative agmatinase 1; AltName: Full=Agmatine ureohydrolase 1; Short=AUH 1; Flags: Precursor && PF00491:Arginase family |
| A03954 | Contig22 | 384252 | 385966 | + | 972 |  | 916.95 | 1083.10 | 895.83 | 286.16 | 156.88 | 310.13 | 1.94 | 0.000 | - && Q9P7U2.1 RecName: Full=Putative aryl-alcohol dehydrogenase C977.14c && PF00248:Aldo/keto reductase family |
| A03955 | Contig22 | 386170 | 392078 | - | 3930 |  | 96.09 | 117.99 | 75.54 | 25.12 | 33.75 | 29.74 | 1.71 | 0.000 | - && - && PF12697:Alpha/beta hydrolase family |
| A03956 | Contig22 | 392743 | 393992 | - | 1194 |  | 58.14 | 33.21 | 89.82 | 224.26 | 402.20 | 139.58 | -2.08 | 0.000 | - && - && PF12937:F-box-like |
| A03958 | Contig22 | 398776 | 403310 | - | 2085 |  | 27.81 | 20.99 | 15.51 | 5.96 | 5.61 | 6.36 | 1.84 | 0.000 | - && O94491.1 RecName: Full=Uncharacterized transporter C417.10 && PF07690:Major Facilitator Superfamily\|PF04191:Phospholipid methyltransferase |
| A03960 | Contig22 | 408897 | 411701 | + | 2748 |  | 0.11 | 0.15 | 0.13 | 1.05 | 0.89 | 0.73 | -2.79 | 0.000 | - && - && - |
| A03971 | Contig23 | 17645 | 19379 | + | 936 |  | 26.97 | 27.89 | 29.96 | 83.21 | 76.01 | 85.32 | -1.53 | 0.000 | - && P94522.3 RecName: Full=Extracellular endo-alpha-(1->5)-L-arabinanase 1; Short=ABN; AltName: Full=Endo-1,5-alpha-L-arabinanase; Flags: Precursor && PF04616:Glycosyl hydrolases family 43 |
| A03985 | Contig23 | 121341 | 124577 | + | 2784 |  | 15.34 | 15.90 | 14.11 | 7.59 | 8.24 | 5.74 | 1.07 | 0.000 | - && - && - |
| A04004 | Contig23 | 186147 | 187286 | + | 816 |  | 7.13 | 14.15 | 7.84 | 42.28 | 75.71 | 30.97 | -2.36 | 0.000 | - && - && PF00106:short chain dehydrogenase |
| A04008 | Contig23 | 195879 | 197361 | - | 1419 |  | 10.70 | 13.65 | 14.94 | 161.43 | 205.76 | 148.80 | -3.72 | 0.000 | - && C5FP82.1 RecName: Full=Probable zinc metalloprotease MCYG_04217; Flags: Precursor && PF04389:Peptidase family M28 |
| A04009 | Contig23 | 198474 | 200257 | + | 1452 |  | 10.53 | 10.44 | 13.02 | 83.06 | 126.23 | 75.25 | -3.07 | 0.000 | - && Q0QWS4.1 RecName: Full=L-galactonate dehydratase && PF13378:Enolase C-terminal domain-like\|PF02746:Mandelate racemase / muconate lactonizing enzyme, N-terminal domain |
| A04010 | Contig23 | 200391 | 201482 | - | 918 |  | 4.30 | 3.72 | 3.58 | 9.17 | 18.66 | 12.14 | -1.79 | 0.000 | - && A7J2C6.1 RecName: Full=Acetylesterase; AltName: Full=Carbohydrate esterase family 16 protein; Flags: Precursor && PF00657:GDSL-like Lipase/Acylhydrolase |
| A04015 | Contig23 | 209601 | 210719 | - | 933 |  | 9.93 | 10.01 | 10.77 | 24.65 | 27.99 | 18.91 | -1.22 | 0.000 | - && A7J2C6.1 RecName: Full=Acetylesterase; AltName: Full=Carbohydrate esterase family 16 protein; Flags: Precursor && PF00657:GDSL-like Lipase/Acylhydrolase |
| A04029 | Contig23 | 293641 | 296204 | + | 1938 |  | 0.51 | 0.83 | 0.28 | 2.80 | 3.43 | 0.82 | -2.12 | 0.000 | - && - && - |
| A04030 | Contig23 | 300592 | 310391 | - | 2628 |  | 12.57 | 12.76 | 22.18 | 40.31 | 49.37 | 32.02 | -1.36 | 0.000 | - && Q0CEF3.1 RecName: Full=Probable beta-glucosidase L; AltName: Full=Beta-D-glucoside glucohydrolase L; AltName: Full=Cellobiase L; AltName: Full=Gentiobiase L; Flags: Precursor && PF14310:Fibronectin type III-like domain\|PF01915:Glycosyl hydrolase family 3 C-terminal domain\|PF00933:Glycosyl hydrolase family 3 N terminal domain |
| A04055 | Contig24 | 11279 | 12173 | - | 714 |  | 0.83 | 1.83 | 0.64 | 11.13 | 12.85 | 7.37 | -3.25 | 0.000 | - && - && - |
| A04057 | Contig24 | 16523 | 17432 | - | 681 |  | 1.74 | 4.42 | 1.61 | 0.69 | 0.82 | 0.52 | 1.94 | 0.006 | - && P78820.2 RecName: Full=Acetyl-CoA carboxylase; Short=ACC; AltName: Full=Cell untimely torn protein 6; Includes: RecName: Full=Biotin carboxylase && - |
| A04063 | Contig24 | 45910 | 46728 | - | 711 |  | 15.53 | 9.46 | 9.89 | 5.65 | 3.16 | 6.67 | 1.17 | 0.001 | - && - && - |
| A04087 | Contig24 | 142224 | 144184 | - | 1209 |  | 56.36 | 31.39 | 36.57 | 7.50 | 4.26 | 6.10 | 2.80 | 0.000 | - && O14134.1 RecName: Full=mRNA export factor elf1 && PF00005:ABC transporter |
| A04088 | Contig24 | 145159 | 145886 | + | 627 |  | 343.93 | 257.78 | 327.81 | 125.56 | 122.87 | 81.74 | 1.49 | 0.000 | - && - && - |
| A04090 | Contig24 | 153211 | 159029 | + | 3240 |  | 1.95 | 1.12 | 1.80 | 8.11 | 6.65 | 5.77 | -2.08 | 0.000 | - && - && - |
| A04091 | Contig24 | 162738 | 164055 | - | 999 |  | 0.39 | 0.60 | 0.27 | 2.43 | 1.31 | 2.72 | -2.35 | 0.000 | - && - && - |
| A04092 | Contig24 | 164828 | 168175 | + | 1995 |  | 4.40 | 4.13 | 5.45 | 20.01 | 19.66 | 17.24 | -2.03 | 0.000 | - && P36616.2 RecName: Full=Protein kinase dsk1; AltName: Full=Dis1-suppressing protein kinase && PF00069:Protein kinase domain |
| A04107 | Contig24 | 234113 | 235604 | + | 1062 |  | 13.18 | 11.63 | 11.78 | 5.37 | 5.64 | 2.73 | 1.41 | 0.000 | - && Q09170.2 RecName: Full=Serine/threonine-protein kinase cds1; AltName: Full=Checkpoint kinase cds1 && PF00069:Protein kinase domain |
| A04112 | Contig24 | 254785 | 256604 | + | 1383 |  | 0.36 | 0.58 | 0.53 | 1.76 | 1.42 | 2.41 | -1.93 | 0.001 | - && - && - |
| A04119 | Contig24 | 272825 | 275140 | - | 1689 |  | 1.23 | 0.65 | 0.65 | 2.27 | 1.33 | 1.71 | -1.07 | 0.029 | - && - && - |
| A04148 | Contig25 | 9475 | 11383 | + | 1422 |  | 47.64 | 42.71 | 33.66 | 8.22 | 12.84 | 15.12 | 1.78 | 0.000 | - && - && - |
| A04153 | Contig25 | 24808 | 26346 | + | 1308 |  | 2.94 | 3.68 | 3.14 | 18.01 | 19.54 | 6.58 | -2.18 | 0.000 | - && - && - |
| A04187 | Contig25 | 165376 | 166779 | - | 1095 |  | 58.80 | 55.28 | 71.49 | 23.22 | 20.52 | 24.20 | 1.45 | 0.000 | - && - && PF13343:Bacterial extracellular solute-binding protein |
| A04188 | Contig25 | 168221 | 170052 | - | 1773 |  | 1576.37 | 911.31 | 2001.86 | 486.67 | 1087.37 | 606.04 | 1.04 | 0.001 | - && - && - |
| A04199 | Contig25 | 200891 | 202803 | - | 1563 |  | 74.57 | 77.59 | 94.86 | 184.12 | 231.00 | 209.60 | -1.34 | 0.000 | - && - && - |
| A04210 | Contig25 | 234484 | 237010 | + | 1653 |  | 4.12 | 5.10 | 4.20 | 0.74 | 2.72 | 1.86 | 1.34 | 0.001 | - && O18635.1 RecName: Full=Cytochrome P450 CYP12A2; AltName: Full=CYPXIIA2 && PF00067:Cytochrome P450 |
| A04214 | Contig25 | 256249 | 257499 | - | 696 |  | 25.08 | 21.06 | 37.27 | 6.58 | 11.03 | 7.19 | 1.75 | 0.000 | - && - && - |
| A04216 | Contig25 | 263648 | 265515 | - | 1479 |  | 8.87 | 9.71 | 7.54 | 18.40 | 28.74 | 22.67 | -1.42 | 0.000 | - && O69763.1 RecName: Full=Vanillin dehydrogenase && PF00171:Aldehyde dehydrogenase family |
| A04241 | Contig25 | 367002 | 369474 | - | 1785 |  | 0.77 | 0.84 | 0.77 | 1.57 | 1.05 | 3.54 | -1.37 | 0.010 | - && P50276.1 RecName: Full=High-affinity methionine permease && PF13520:Amino acid permease |
| A04243 | Contig25 | 377515 | 378815 | + | 840 |  | 3.29 | 5.26 | 6.96 | 10.46 | 21.06 | 11.28 | -1.47 | 0.000 | - && F7J1C8.1 RecName: Full=Galactan endo-beta-1,3-galactanase; AltName: Full=Endo-beta-1,3-galactanase; Short=FvEN3GAL; Flags: Precursor && - |
| A04246 | Contig26 | 2567 | 4160 | + | 1425 |  | 3.39 | 2.47 | 2.37 | 0.92 | 0.92 | 0.43 | 1.86 | 0.000 | - && - && - |
| A04247 | Contig26 | 5262 | 5795 | - | 534 |  | 47.64 | 36.28 | 58.33 | 22.76 | 17.71 | 11.67 | 1.45 | 0.000 | - && - && PF07883:Cupin domain |
| A04250 | Contig26 | 32871 | 41431 | + | 2136 |  | 29.87 | 21.01 | 42.34 | 11.16 | 12.10 | 8.46 | 1.56 | 0.000 | - && Q9C0V8.1 RecName: Full=Uncharacterized transporter PB10D8.01 && PF07690:Major Facilitator Superfamily |
| A04255 | Contig26 | 56569 | 59850 | - | 2367 |  | 69.44 | 75.11 | 63.33 | 94.21 | 129.60 | 199.25 | -1.03 | 0.000 | - && O13966.2 RecName: Full=Aconitate hydratase, mitochondrial; Short=Aconitase; AltName: Full=Citrate hydro-lyase; Flags: Precursor && PF00330:Aconitase family (aconitate hydratase)\|PF00694:Aconitase C-terminal domain |
| A04259 | Contig26 | 72615 | 75722 | + | 1893 |  | 1.61 | 0.53 | 1.11 | 0.40 | 0.45 | 0.32 | 1.48 | 0.016 | - && - && PF00076:RNA recognition motif. (a.k.a. RRM, RBD, or RNP domain) |
| A04275 | Contig26 | 209442 | 210420 | + | 618 |  | 14.36 | 11.53 | 9.90 | 4.39 | 3.94 | 1.70 | 1.84 | 0.000 | - && - && - |
| A04280 | Contig26 | 229023 | 230150 | - | 720 |  | 21.91 | 24.40 | 16.24 | 7.01 | 7.41 | 9.39 | 1.39 | 0.000 | - && - && PF00106:short chain dehydrogenase |
| A04281 | Contig26 | 231183 | 234031 | + | 1293 |  | 7.09 | 5.28 | 5.30 | 2.60 | 2.90 | 3.05 | 1.05 | 0.001 | - && - && PF00328:Histidine phosphatase superfamily (branch 2) |
| A04282 | Contig26 | 234447 | 236147 | - | 1521 |  | 602.31 | 387.43 | 841.67 | 120.05 | 183.04 | 62.78 | 2.32 | 0.000 | - && - && - |
| A04288 | Contig26 | 263151 | 264948 | - | 1590 |  | 990.93 | 1069.92 | 2197.56 | 234.27 | 443.74 | 442.16 | 1.93 | 0.000 | - && - && PF01823:MAC/Perforin domain |
| A04318 | Contig27 | 31805 | 33303 | - | 1224 |  | 6.12 | 5.66 | 8.73 | 2.44 | 2.68 | 2.51 | 1.43 | 0.000 | - && - && - |
| A04330 | Contig27 | 74770 | 75418 | + | 519 |  | 118.93 | 182.01 | 40.66 | 9.91 | 6.85 | 3.72 | 4.06 | 0.000 | - && - && - |
| A04331 | Contig27 | 75943 | 80511 | + | 2178 |  | 10.05 | 9.68 | 6.67 | 24.68 | 18.57 | 20.51 | -1.27 | 0.000 | - && Q09653.3 RecName: Full=Putative cytochrome P450 CYP13A10 && PF00067:Cytochrome P450 |
| A04336 | Contig27 | 102668 | 106349 | - | 2949 |  | 2.21 | 2.42 | 1.30 | 13.95 | 19.78 | 8.60 | -2.84 | 0.000 | - && P29064.1 RecName: Full=Alpha-glucosidase; AltName: Full=Maltase; Contains: RecName: Full=Alpha-glucosidase subunit 1; Contains: RecName: Full=Alpha-glucosidase subunit 2; Flags: Precursor && PF16863:N-terminal barrel of NtMGAM and CtMGAM, maltase-glucoamylase\|PF01055:Glycosyl hydrolases family 31 |
| A04359 | Contig27 | 213878 | 216687 | - | 1794 |  | 6.65 | 4.92 | 10.49 | 143.42 | 130.29 | 123.18 | -4.17 | 0.000 | - && J9VQZ4.2 RecName: Full=Laccase-2; AltName: Full=Diphenol oxidase 2; Flags: Precursor && PF07732:Multicopper oxidase\|PF07731:Multicopper oxidase\|PF00394:Multicopper oxidase |
| A04360 | Contig27 | 218358 | 220113 | + | 1380 |  | 4.29 | 3.20 | 5.36 | 46.95 | 55.15 | 37.97 | -3.45 | 0.000 | - && Q8X176.1 RecName: Full=Acid phosphatase; Flags: Precursor && PF04185:Phosphoesterase family |
| A04367 | Contig27 | 242408 | 243168 | - | 588 |  | 134.66 | 151.09 | 316.31 | 440.61 | 319.52 | 702.52 | -1.28 | 0.000 | - && - && - |
| A04371 | Contig27 | 261110 | 262310 | - | 1041 |  | 8.24 | 5.40 | 5.18 | 2.87 | 3.24 | 2.11 | 1.20 | 0.001 | - && - && - |
| A04373 | Contig27 | 266805 | 267620 | - | 642 |  | 579.64 | 589.51 | 531.02 | 212.19 | 107.32 | 223.22 | 1.65 | 0.000 | - && P42769.1 RecName: Full=Glutathione S-transferase PM239X14; AltName: Full=GST class-phi && PF00043:Glutathione S-transferase, C-terminal domain\|PF02798:Glutathione S-transferase, N-terminal domain |
| A04374 | Contig27 | 268477 | 274230 | - | 4035 |  | 0.49 | 0.60 | 0.48 | 0.19 | 0.12 | 0.20 | 1.65 | 0.004 | - && - && PF04937:Protein of unknown function (DUF 659) |
| A04379 | Contig27 | 286583 | 288778 | + | 939 |  | 2175.99 | 1841.63 | 3390.93 | 534.29 | 822.65 | 317.39 | 2.15 | 0.000 | - && P42769.1 RecName: Full=Glutathione S-transferase PM239X14; AltName: Full=GST class-phi && PF00043:Glutathione S-transferase, C-terminal domain\|PF02798:Glutathione S-transferase, N-terminal domain |
| A04382 | Contig27 | 309898 | 310714 | + | 624 |  | 66.68 | 76.42 | 69.10 | 237.04 | 275.13 | 457.36 | -2.19 | 0.000 | - && P61517.1 RecName: Full=Carbonic anhydrase 2; AltName: Full=Carbonate dehydratase 2 >P61518.1 RecName: Full=Carbonic anhydrase 2; AltName: Full=Carbonate dehydratase 2 && PF00484:Carbonic anhydrase |
| A04383 | Contig27 | 313948 | 320326 | + | 5004 |  | 41.81 | 38.48 | 43.37 | 17.75 | 18.95 | 18.66 | 1.16 | 0.000 | - && P32386.2 RecName: Full=ATP-dependent bile acid permease && PF00005:ABC transporter\|PF00664:ABC transporter transmembrane region |
| A04400 | Contig28 | 47621 | 48955 | + | 831 |  | 31.44 | 39.74 | 28.80 | 15.41 | 12.62 | 21.33 | 1.02 | 0.000 | - && - && - |
| A04402 | Contig28 | 54884 | 56392 | - | 1206 |  | 15.13 | 11.82 | 18.18 | 2.02 | 1.24 | 1.09 | 3.38 | 0.000 | - && Q6F6Y2.1 RecName: Full=FAD-dependent urate hydroxylase; AltName: Full=Flavoprotein urate hydroxylase && PF01494:FAD binding domain |
| A04421 | Contig28 | 109532 | 111361 | - | 1539 |  | 19.93 | 14.22 | 14.54 | 7.96 | 6.57 | 5.25 | 1.30 | 0.000 | - && A0A0D2YFZ8.1 RecName: Full=Efflux pump FUB11; AltName: Full=Fusaric acid biosynthesis protein 11 && PF07690:Major Facilitator Superfamily |
| A04426 | Contig28 | 131558 | 132864 | - | 1074 |  | 122.93 | 134.51 | 54.35 | 28.03 | 17.69 | 28.11 | 2.08 | 0.000 | - && Q6ZXC1.2 RecName: Full=Probable inactive dehydrogenase EasA; AltName: Full=Ergot alkaloid biosynthesis protein A [Claviceps purpurea] && PF00724:NADH:flavin oxidoreductase / NADH oxidase family |
| A04441 | Contig28 | 225448 | 227277 | + | 1539 |  | 10.25 | 8.41 | 12.29 | 4.80 | 3.41 | 1.82 | 1.63 | 0.000 | - && A0A0D2YFZ8.1 RecName: Full=Efflux pump FUB11; AltName: Full=Fusaric acid biosynthesis protein 11 && PF07690:Major Facilitator Superfamily |
| A04442 | Contig28 | 228772 | 230155 | + | 1041 |  | 12.41 | 5.98 | 12.99 | 2.69 | 2.70 | 3.62 | 1.80 | 0.000 | - && Q6ZXC1.2 RecName: Full=Probable inactive dehydrogenase EasA; AltName: Full=Ergot alkaloid biosynthesis protein A [Claviceps purpurea] && PF00724:NADH:flavin oxidoreductase / NADH oxidase family |
| A04455 | Contig28 | 271290 | 272140 | - | 603 |  | 5.72 | 5.16 | 3.03 | 1.09 | 2.64 | 1.46 | 1.43 | 0.008 | - && P36639.3 RecName: Full=7,8-dihydro-8-oxoguanine triphosphatase; AltName: Full=2-hydroxy-dATP diphosphatase; AltName: Full=8-oxo-dGTPase; AltName: Full=Nucleoside diphosphate-linked moiety X motif 1; Short=Nudix motif 1; Flags: Precursor && PF00293:NUDIX domain |
| A04473 | Contig29 | 39170 | 40138 | - | 969 |  | 7.02 | 6.01 | 5.47 | 2.80 | 2.42 | 0.82 | 1.62 | 0.000 | - && - && - |
| A04482 | Contig29 | 77238 | 77707 | - | 342 |  | 6.05 | 13.50 | 8.01 | 31.17 | 21.90 | 15.91 | -1.32 | 0.001 | - && P52748.1 RecName: Full=Hydrophobin-1; Flags: Precursor [Pisolithus tinctorius] && PF01185:Fungal hydrophobin |
| A04486 | Contig29 | 87143 | 88719 | + | 1266 |  | 53.12 | 39.41 | 39.11 | 12.19 | 17.67 | 8.94 | 1.76 | 0.000 | - && P42687.1 RecName: Full=Tyrosine-protein kinase SPK-1 [Girardia tigrina] && PF00069:Protein kinase domain |
| A04488 | Contig29 | 95233 | 95676 | + | 327 |  | 3.32 | 1.53 | 2.51 | 5.72 | 4.58 | 8.59 | -1.36 | 0.022 | - && - && PF01185:Fungal hydrophobin |
| A04492 | Contig29 | 109090 | 114297 | + | 3405 |  | 96.55 | 51.77 | 105.84 | 18.64 | 23.37 | 20.72 | 2.02 | 0.000 | - && P48777.2 RecName: Full=Purine permease && PF00850:Histone deacetylase domain\|PF00860:Permease family |
| A04496 | Contig29 | 121368 | 122006 | - | 639 |  | 1.23 | 2.83 | 2.14 | 4.24 | 4.54 | 3.85 | -1.02 | 0.038 | - && - && - |
| A04497 | Contig29 | 124281 | 126391 | + | 1635 |  | 186.23 | 138.70 | 206.11 | 376.33 | 310.15 | 442.39 | -1.09 | 0.000 | - && - && PF05577:Serine carboxypeptidase S28 |
| A04499 | Contig29 | 129403 | 132326 | - | 1773 |  | 34.93 | 31.99 | 24.52 | 13.66 | 6.81 | 11.73 | 1.51 | 0.000 | - && P13006.1 RecName: Full=Glucose oxidase; AltName: Full=Beta-D-glucose:oxygen 1-oxido-reductase; AltName: Full=Glucose oxyhydrase; Short=GOD; Flags: Precursor && PF00732:GMC oxidoreductase\|PF05199:GMC oxidoreductase |
| A04500 | Contig29 | 135153 | 137257 | + | 1683 |  | 10.84 | 14.61 | 7.49 | 3.44 | 4.51 | 4.95 | 1.35 | 0.000 | - && Q12732.2 RecName: Full=Averantin hydroxylase; AltName: Full=Aflatoxin biosynthesis protein G; AltName: Full=Cytochrome P450 60A1 [Aspergillus parasiticus] && PF00067:Cytochrome P450 |
| A04508 | Contig29 | 172319 | 174874 | + | 1821 |  | 35.58 | 37.82 | 47.41 | 122.35 | 175.91 | 97.88 | -1.71 | 0.000 | - && Q70J59.1 RecName: Full=Tripeptidyl-peptidase sed2; AltName: Full=Sedolisin-B; Flags: Precursor && PF09286:Pro-kumamolisin, activation domain |
| A04513 | Contig29 | 186628 | 190060 | + | 1968 |  | 1.25 | 1.48 | 1.72 | 9.12 | 1.57 | 1.25 | -1.42 | 0.020 | - && Q3TQI7.2 RecName: Full=Uncharacterized protein C9orf78 homolog && PF07052:Hepatocellular carcinoma-associated antigen 59 |
| A04537 | Contig29 | 285830 | 288144 | - | 1980 |  | 0.30 | 0.35 | 0.32 | 1.09 | 1.18 | 1.20 | -1.83 | 0.001 | - && - && - |
| A04541 | Contig29 | 36876 | 38367 | + | 312 |  | 38.24 | 49.87 | 33.08 | 80.91 | 94.81 | 69.48 | -1.02 | 0.000 | - && P04158.2 RecName: Full=Fruiting body protein SC1; AltName: Full=Hydrophobin SC1; Flags: Precursor && PF01185:Fungal hydrophobin |
| A04545 | Contig2 | 3400048 | 3400630 | + | 474 |  | 3190.45 | 2548.46 | 3848.44 | 1632.03 | 1757.86 | 764.09 | 1.21 | 0.000 | - && - && - |
| A04546 | Contig2 | 3400904 | 3402334 | + | 1431 |  | 149.59 | 130.06 | 148.80 | 44.49 | 38.99 | 38.27 | 1.82 | 0.000 | - && - && PF00651:BTB/POZ domain |
| A04553 | Contig2 | 3421176 | 3424846 | - | 1908 |  | 1.96 | 3.16 | 2.15 | 7.60 | 6.38 | 5.15 | -1.39 | 0.000 | - && P52289.2 RecName: Full=Repressible acid phosphatase; Flags: Precursor && PF00328:Histidine phosphatase superfamily (branch 2) |
| A04559 | Contig2 | 3446254 | 3450346 | - | 2058 |  | 11.12 | 9.22 | 5.95 | 28.89 | 23.47 | 10.28 | -1.25 | 0.000 | - && O94753.1 RecName: Full=Versatile peroxidase VPL2; AltName: Full=Versatile liquid phase peroxidase 2; Flags: Precursor && PF11895:Domain of unknown function (DUF3415)\|PF00141:Peroxidase |
| A04561 | Contig2 | 3459354 | 3460102 | - | 414 |  | 6.43 | 5.58 | 6.40 | 2.03 | 2.49 | 1.48 | 1.62 | 0.002 | - && P78594.1 RecName: Full=Cytosine deaminase; AltName: Full=Cytosine aminohydrolase && - |
| A04573 | Contig2 | 3497814 | 3499105 | + | 1065 |  | 168.69 | 133.95 | 156.20 | 22.83 | 30.15 | 21.84 | 2.62 | 0.000 | - && P81054.2 RecName: Full=Peptidyl-Lys metalloendopeptidase; Short=MEP; AltName: Full=GfMEP; Flags: Precursor && PF14521:Lysine-specific metallo-endopeptidase |
| A04578 | Contig2 | 3512089 | 3513218 | - | 681 |  | 89.63 | 128.25 | 127.70 | 172.58 | 259.25 | 294.72 | -1.07 | 0.000 | - && P25613.1 RecName: Full=Accumulation of dyads protein 2; AltName: Full=Ammonia transport outward protein 1 && PF01184:GPR1/FUN34/yaaH family |
| A04580 | Contig2 | 3518180 | 3520071 | + | 1512 |  | 740.24 | 1247.75 | 607.91 | 1840.67 | 2123.94 | 3294.55 | -1.48 | 0.000 | - && O74187.1 RecName: Full=Aldehyde dehydrogenase; Short=ALDDH; Short=ALDH && PF00171:Aldehyde dehydrogenase family |
| A04585 | Contig2 | 3537799 | 3539563 | - | 1161 |  | 44.42 | 48.85 | 43.67 | 28.02 | 21.29 | 18.37 | 1.02 | 0.000 | - && - && PF01048:Phosphorylase superfamily |
| A04589 | Contig2 | 3558142 | 3559105 | - | 798 |  | 151.36 | 111.08 | 129.70 | 24.14 | 19.24 | 11.44 | 2.84 | 0.000 | - && - && - |
| A04590 | Contig2 | 3559910 | 3562122 | + | 891 |  | 3884.47 | 3065.61 | 2537.59 | 273.46 | 238.60 | 100.27 | 3.95 | 0.000 | - && - && - |
| A04599 | Contig2 | 333232 | 338973 | - | 3696 |  | 1.63 | 2.36 | 1.95 | 4.78 | 7.52 | 5.25 | -1.56 | 0.000 | - && D4ATR3.1 RecName: Full=Uncharacterized secreted glycosidase ARB_07629; Flags: Precursor && PF07971:Glycosyl hydrolase family 92\|PF07690:Major Facilitator Superfamily |
| A04605 | Contig2 | 3597963 | 3598673 | - | 558 |  | 1.77 | 2.52 | 1.96 | 5.70 | 4.03 | 5.03 | -1.24 | 0.011 | - && - && - |
| A04612 | Contig2 | 3613205 | 3614850 | - | 1371 |  | 4.39 | 5.56 | 7.33 | 13.09 | 12.29 | 22.85 | -1.48 | 0.000 | - && - && PF01823:MAC/Perforin domain |
| A04613 | Contig2 | 3616030 | 3617834 | + | 1338 |  | 7.59 | 17.18 | 15.36 | 16.91 | 37.71 | 67.82 | -1.61 | 0.000 | - && - && PF01823:MAC/Perforin domain |
| A04617 | Contig2 | 348018 | 350197 | + | 1719 |  | 42.91 | 50.05 | 39.06 | 13.65 | 16.66 | 8.17 | 1.78 | 0.000 | - && P40375.1 RecName: Full=NAD-dependent malic enzyme; Short=NAD-ME && PF00390:Malic enzyme, N-terminal domain\|PF03949:Malic enzyme, NAD binding domain |
| A04635 | Contig2 | 405681 | 407339 | - | 1557 |  | 4.24 | 6.64 | 4.40 | 13.63 | 12.20 | 10.60 | -1.25 | 0.000 | - && - && - |
| A04639 | Contig2 | 422319 | 423059 | - | 741 |  | 209.85 | 213.92 | 205.63 | 132.74 | 89.19 | 85.03 | 1.04 | 0.000 | - && - && - |
| A04649 | Contig2 | 436660 | 437669 | + | 741 |  | 15.84 | 15.72 | 12.20 | 19.94 | 79.34 | 20.73 | -1.46 | 0.001 | - && - && - |
| A04655 | Contig2 | 444722 | 446861 | + | 1989 |  | 0.64 | 0.71 | 1.33 | 3.90 | 4.33 | 2.47 | -2.00 | 0.000 | - && P49008.2 RecName: Full=Beta-hexosaminidase; AltName: Full=Beta-GlcNAcase; AltName: Full=Beta-N-acetylhexosaminidase; Short=Beta-NAHase; AltName: Full=N-acetyl-beta-glucosaminidase; Flags: Precursor && PF02838:Glycosyl hydrolase family 20, domain 2\|PF00728:Glycosyl hydrolase family 20, catalytic domain |
| A04657 | Contig2 | 449067 | 451437 | + | 2268 |  | 64.52 | 78.74 | 63.28 | 33.56 | 17.01 | 41.36 | 1.17 | 0.000 | - && Q9FNA3.1 RecName: Full=Alpha-N-acetylglucosaminidase; AltName: Full=N-acetyl-glucosaminidase; Short=AtNAGLU; AltName: Full=Protein CYCLOPS 1; Flags: Precursor && PF05089:Alpha-N-acetylglucosaminidase (NAGLU) tim-barrel domain\|PF12972:Alpha-N-acetylglucosaminidase (NAGLU) C-terminal domain\|PF12971:Alpha-N-acetylglucosaminidase (NAGLU) N-terminal domain |
| A04662 | Contig2 | 39020 | 41100 | - | 1401 |  | 12.74 | 8.31 | 6.46 | 4.27 | 3.01 | 5.20 | 1.14 | 0.001 | - && O69687.4 RecName: Full=Probable fatty acid methyltransferase Rv3720; AltName: Full=S-adenosylmethionine-dependent methyltransferase Rv3720; Short=AdoMet-MT; Short=SAM-MT && PF02353:Mycolic acid cyclopropane synthetase |
| A04681 | Contig2 | 548154 | 549655 | - | 1284 |  | 127.71 | 86.63 | 135.60 | 258.79 | 273.39 | 361.01 | -1.35 | 0.000 | - && P23572.1 RecName: Full=Cyclin-dependent kinase 1; Short=CDK1; AltName: Full=Cell division control protein 2 homolog; AltName: Full=Cell division protein kinase 1; AltName: Full=p34 protein kinase && PF00069:Protein kinase domain |
| A04683 | Contig2 | 552279 | 554472 | + | 1656 |  | 129.21 | 119.79 | 158.98 | 313.52 | 283.88 | 271.60 | -1.09 | 0.000 | - && Q10177.1 RecName: Full=Manganese transporter pdt1 && PF01566:Natural resistance-associated macrophage protein |
| A04702 | Contig2 | 618088 | 621142 | - | 2697 |  | 7.86 | 11.50 | 8.26 | 23.23 | 20.27 | 27.59 | -1.36 | 0.000 | - && O60161.1 RecName: Full=U3 small nucleolar RNA-associated protein 4; Short=U3 snoRNA-associated protein 4; AltName: Full=U3 protein 4 required for transcription && - |
| A04713 | Contig2 | 650542 | 651361 | - | 645 |  | 169.23 | 212.61 | 96.31 | 80.45 | 74.02 | 50.61 | 1.22 | 0.000 | - && - && - |
| A04730 | Contig2 | 695936 | 697838 | + | 1470 |  | 984.29 | 1174.61 | 833.08 | 367.56 | 430.16 | 376.82 | 1.35 | 0.000 | - && O13287.1 RecName: Full=6-phosphogluconate dehydrogenase, decarboxylating && PF00393:6-phosphogluconate dehydrogenase, C-terminal domain\|PF03446:NAD binding domain of 6-phosphogluconate dehydrogenase |
| A04735 | Contig2 | 708064 | 708780 | + | 609 |  | 32.87 | 33.46 | 34.80 | 67.40 | 68.09 | 71.19 | -1.03 | 0.000 | - && - && - |
| A04759 | Contig2 | 784768 | 786781 | + | 1419 |  | 90.82 | 86.81 | 90.77 | 163.21 | 221.92 | 240.83 | -1.22 | 0.000 | - && P55250.1 RecName: Full=Fumarate hydratase, mitochondrial; Short=Fumarase; Flags: Precursor && PF10415:Fumarase C C-terminus\|PF00206:Lyase |
| A04766 | Contig2 | 806601 | 808654 | + | 1083 |  | 355.08 | 380.88 | 283.66 | 173.70 | 105.54 | 174.06 | 1.17 | 0.000 | - && Q00859.1 RecName: Full=Mitogen-activated protein kinase; AltName: Full=FsMAPK && PF00069:Protein kinase domain |
| A04769 | Contig2 | 814737 | 815992 | - | 762 |  | 8.41 | 6.59 | 17.50 | 3.56 | 5.04 | 3.46 | 1.43 | 0.001 | - && - && - |
| A04817 | Contig2 | 89487 | 91787 | - | 2043 |  | 100.44 | 66.68 | 158.91 | 56.66 | 71.39 | 34.58 | 1.00 | 0.002 | - && P36916.4 RecName: Full=Guanine nucleotide-binding protein-like 1; AltName: Full=GTP-binding protein MMR1 && PF01926:50S ribosome-binding GTPase |
| A04824 | Contig2 | 976000 | 979238 | - | 2526 |  | 4.06 | 4.01 | 3.80 | 16.29 | 27.02 | 17.54 | -2.36 | 0.000 | - && Q7Z9M8.1 RecName: Full=Xyloglucanase; Short=XG; AltName: Full=Cel74a; Flags: Precursor && PF00734:Fungal cellulose binding domain |
| A04838 | Contig2 | 1021661 | 1024589 | + | 2814 |  | 91.45 | 80.55 | 102.52 | 27.74 | 46.47 | 31.16 | 1.38 | 0.000 | - && - && - |
| A04842 | Contig2 | 1029072 | 1030501 | + | 828 |  | 90.98 | 80.38 | 96.31 | 265.02 | 321.53 | 187.49 | -1.53 | 0.000 | - && Q07505.1 RecName: Full=Putative carboxymethylenebutenolidase; AltName: Full=Dienelactone hydrolase; Short=DLH && PF01738:Dienelactone hydrolase family |
| A04846 | Contig2 | 1037062 | 1038513 | + | 1059 |  | 47.39 | 62.85 | 54.34 | 24.63 | 30.41 | 22.79 | 1.08 | 0.000 | - && Q6GNT9.1 RecName: Full=Protein MEMO1; AltName: Full=Mediator of ErbB2-driven cell motility 1; Short=Memo-1 && PF01875:Memo-like protein |
| A04851 | Contig2 | 99269 | 104291 | - | 2757 |  | 6.29 | 8.59 | 9.51 | 3.80 | 3.84 | 3.79 | 1.09 | 0.000 | - && P0CG48.3 RecName: Full=Polyubiquitin-C; Contains: RecName: Full=Ubiquitin; Flags: Precursor && PF00240:Ubiquitin family |
| A04855 | Contig2 | 1053460 | 1061324 | - | 5103 |  | 7.96 | 7.38 | 10.36 | 17.20 | 25.37 | 16.13 | -1.19 | 0.000 | - && - && PF02278:Polysaccharide lyase family 8, super-sandwich domain\|PF08124:Polysaccharide lyase family 8, N terminal alpha-helical domain\|PF02884:Polysaccharide lyase family 8, C-terminal beta-sandwich domain |
| A04856 | Contig2 | 1062062 | 1064856 | - | 2412 |  | 26.86 | 25.76 | 55.11 | 83.46 | 80.65 | 69.53 | -1.12 | 0.000 | - && - && PF02884:Polysaccharide lyase family 8, C-terminal beta-sandwich domain\|PF02278:Polysaccharide lyase family 8, super-sandwich domain\|PF08124:Polysaccharide lyase family 8, N terminal alpha-helical domain |
| A04861 | Contig2 | 1076742 | 1080004 | + | 1761 |  | 130.52 | 118.40 | 137.57 | 36.47 | 49.81 | 45.65 | 1.55 | 0.000 | - && Q9JK81.1 RecName: Full=UPF0160 protein MYG1, mitochondrial; AltName: Full=Protein Gamm1; Flags: Precursor && PF03690:Uncharacterised protein family (UPF0160)\|PF01975:Survival protein SurE |
| A04878 | Contig2 | 1117294 | 1118350 | + | 888 |  | 27.32 | 34.82 | 43.82 | 96.76 | 124.29 | 92.21 | -1.56 | 0.000 | - && Q03161.1 RecName: Full=Glucose-6-phosphate 1-epimerase; AltName: Full=D-hexose-6-phosphate mutarotase && PF01263:Aldose 1-epimerase |
| A04891 | Contig2 | 1160139 | 1160905 | - | 468 |  | 737.20 | 670.54 | 784.87 | 2034.93 | 3458.18 | 1232.37 | -1.62 | 0.000 | - && - && - |
| A04897 | Contig2 | 1185581 | 1188649 | - | 2706 |  | 2775.50 | 1978.75 | 2202.05 | 906.25 | 622.34 | 608.41 | 1.70 | 0.000 | - && O94641.1 RecName: Full=Heat shock protein 104; AltName: Full=Protein aggregation-remodeling factor hsp104 && PF07724:AAA domain (Cdc48 subfamily)\|PF00004:ATPase family associated with various cellular activities (AAA)\|PF10431:C-terminal, D2-small domain, of ClpB protein\|PF02861:Clp amino terminal domain, pathogenicity island component |
| A04901 | Contig2 | 1201601 | 1203673 | + | 1725 |  | 35.44 | 57.91 | 12.44 | 1.52 | 0.81 | 2.54 | 4.44 | 0.000 | - && D4AS41.2 RecName: Full=Uncharacterized FAD-linked oxidoreductase ARB_02478; Flags: Precursor && PF08031:Berberine and berberine like\|PF01565:FAD binding domain |
| A04909 | Contig2 | 1222515 | 1224501 | - | 1446 |  | 32.94 | 20.27 | 32.91 | 8.34 | 5.50 | 7.22 | 2.03 | 0.000 | - && - && - |
| A04910 | Contig2 | 1226096 | 1227643 | + | 543 |  | 13.44 | 10.91 | 16.65 | 2.24 | 4.48 | 1.45 | 2.33 | 0.000 | - && - && - |
| A04926 | Contig2 | 1281451 | 1282514 | + | 768 |  | 8.09 | 8.63 | 10.47 | 26.17 | 31.81 | 17.14 | -1.47 | 0.000 | - && A3LZU7.2 RecName: Full=L-rhamnose-1-dehydrogenase && PF00106:short chain dehydrogenase |
| A04931 | Contig2 | 1293320 | 1293940 | + | 621 |  | 95.90 | 74.04 | 69.73 | 34.33 | 37.99 | 19.64 | 1.38 | 0.000 | - && Q9USJ6.1 RecName: Full=NAD(P)H-dependent FMN reductase C4B3.06c; Short=FMN reductase C4B3.06c; AltName: Full=Azoreductase C4B3.06c; AltName: Full=FMN reductase [NAD(P)H] && PF03358:NADPH-dependent FMN reductase |
| A04985 | Contig2 | 1440072 | 1440857 | + | 786 |  | 17.94 | 19.41 | 21.27 | 59.95 | 50.14 | 50.13 | -1.45 | 0.000 | - && - && - |
| A05003 | Contig2 | 1499990 | 1501136 | - | 933 |  | 14.48 | 19.37 | 18.02 | 62.03 | 60.50 | 63.02 | -1.84 | 0.000 | - && D4ARV2.1 RecName: Full=Altered inheritance of mitochondria protein 6 homolog ARB_06966; Flags: Precursor && - |
| A05029 | Contig2 | 1552767 | 1554033 | - | 1026 |  | 6.25 | 6.65 | 5.79 | 33.26 | 54.74 | 27.20 | -2.62 | 0.000 | - && - && - |
| A05030 | Contig2 | 1555479 | 1556739 | + | 1092 |  | 26.55 | 33.83 | 25.68 | 168.75 | 231.80 | 161.94 | -2.71 | 0.000 | - && - && - |
| A05033 | Contig2 | 1563969 | 1565057 | + | 810 |  | 46.50 | 99.15 | 77.82 | 146.82 | 235.41 | 101.08 | -1.11 | 0.001 | - && P39577.2 RecName: Full=Uncharacterized oxidoreductase DltE && PF00106:short chain dehydrogenase |
| A05042 | Contig2 | 1584895 | 1586607 | + | 1608 |  | 83.64 | 36.08 | 71.64 | 14.19 | 7.63 | 8.62 | 2.65 | 0.000 | - && - && PF00501:AMP-binding enzyme |
| A05047 | Contig2 | 1602450 | 1604315 | - | 1683 |  | 118.52 | 112.50 | 108.56 | 49.28 | 47.44 | 50.94 | 1.20 | 0.000 | - && - && PF10297:Minimal binding motif of Hap4 for binding to Hap2/3/5 |
| A05048 | Contig2 | 1606061 | 1608113 | + | 1113 |  | 47.13 | 46.00 | 48.67 | 25.37 | 24.98 | 19.16 | 1.03 | 0.000 | - && P25346.1 RecName: Full=Probable metabolite transport protein GIT1 && PF00083:Sugar (and other) transporter |
| A05050 | Contig2 | 9921 | 11717 | - | 1317 |  | 67.98 | 78.28 | 57.50 | 13.70 | 10.80 | 22.99 | 2.10 | 0.000 | - && - && - |
| A05078 | Contig2 | 1690611 | 1691282 | + | 495 |  | 569.91 | 577.59 | 572.27 | 1348.80 | 911.33 | 1400.24 | -1.09 | 0.000 | - && - && - |
| A05094 | Contig2 | 1732004 | 1735289 | + | 2370 |  | 267.48 | 192.90 | 162.19 | 106.24 | 56.68 | 89.28 | 1.30 | 0.000 | - && O74402.1 RecName: Full=Heat shock protein 78, mitochondrial; Flags: Precursor && PF10431:C-terminal, D2-small domain, of ClpB protein\|PF00004:ATPase family associated with various cellular activities (AAA)\|PF07724:AAA domain (Cdc48 subfamily) |
| A05136 | Contig2 | 1862655 | 1863273 | + | 372 |  | 529.33 | 253.67 | 601.14 | 147.03 | 67.94 | 98.37 | 2.14 | 0.000 | - && P81762.1 RecName: Full=Guanyl-specific ribonuclease Po1; Short=RNase Po1 && - |
| A05138 | Contig2 | 1864626 | 1869740 | + | 4446 |  | 9.07 | 12.08 | 8.38 | 18.38 | 15.81 | 29.88 | -1.12 | 0.000 | - && O74835.1 RecName: Full=rRNA biogenesis protein rrp5; AltName: Full=Ribosomal RNA-processing protein 5; AltName: Full=U3 small nucleolar RNA-associated protein rrp5; Short=U3 snoRNA-associated protein rrp5 && PF00575:S1 RNA binding domain |
| A05161 | Contig2 | 13325 | 15634 | + | 1551 |  | 4.13 | 10.68 | 5.12 | 13.50 | 16.90 | 9.62 | -1.01 | 0.005 | - && P18631.1 RecName: Full=Low-affinity glucose transporter; AltName: Full=Hexose transporter 1 && PF00083:Sugar (and other) transporter |
| A05162 | Contig2 | 185776 | 187285 | - | 972 |  | 74.66 | 72.09 | 61.37 | 32.51 | 32.07 | 27.27 | 1.18 | 0.000 | - && - && - |
| A05165 | Contig2 | 1929259 | 1930311 | - | 1053 |  | 15.08 | 13.44 | 21.60 | 84.00 | 86.32 | 62.34 | -2.21 | 0.000 | - && P39462.1 RecName: Full=NAD-dependent alcohol dehydrogenase && PF08240:Alcohol dehydrogenase GroES-like domain\|PF00107:Zinc-binding dehydrogenase |
| A05168 | Contig2 | 1942974 | 1943770 | - | 564 |  | 3.85 | 2.49 | 1.13 | 7.13 | 5.98 | 5.76 | -1.34 | 0.007 | - && - && - |
| A05169 | Contig2 | 1945295 | 1946981 | + | 1239 |  | 553.50 | 444.09 | 682.58 | 62.56 | 100.49 | 40.02 | 3.05 | 0.000 | - && P17576.1 RecName: Full=Polyporopepsin; AltName: Full=Aspartic proteinase && PF00026:Eukaryotic aspartyl protease |
| A05170 | Contig2 | 1948205 | 1949932 | + | 1257 |  | 112.72 | 118.28 | 169.55 | 409.76 | 404.75 | 240.38 | -1.40 | 0.000 | - && P17576.1 RecName: Full=Polyporopepsin; AltName: Full=Aspartic proteinase && PF00026:Eukaryotic aspartyl protease |
| A05175 | Contig2 | 1958476 | 1960038 | + | 732 |  | 50.92 | 48.00 | 40.93 | 32.70 | 14.83 | 19.06 | 1.07 | 0.000 | - && - && PF13840:ACT domain |
| A05198 | Contig2 | 2022322 | 2023086 | + | 714 |  | 20.58 | 18.98 | 33.26 | 66.13 | 94.79 | 90.59 | -1.79 | 0.000 | - && - && - |
| A05208 | Contig2 | 2043236 | 2044040 | - | 675 |  | 11.98 | 9.52 | 8.26 | 21.47 | 29.68 | 17.03 | -1.20 | 0.000 | - && - && - |
| A05221 | Contig2 | 2074762 | 2075127 | + | 366 |  | 11.85 | 37.30 | 19.97 | 65.14 | 49.36 | 29.01 | -1.05 | 0.009 | - && - && - |
| A05237 | Contig2 | 2123817 | 2125718 | - | 1680 |  | 30.17 | 33.28 | 26.70 | 59.38 | 56.11 | 94.92 | -1.22 | 0.000 | - && Q4P6N0.2 RecName: Full=ATP-dependent RNA helicase HAS1 && PF13959:Domain of unknown function (DUF4217)\|PF00270:DEAD/DEAH box helicase\|PF00271:Helicase conserved C-terminal domain |
| A05242 | Contig2 | 2131225 | 2132990 | + | 1251 |  | 184.67 | 171.33 | 150.94 | 415.61 | 655.57 | 546.54 | -1.67 | 0.000 | - && P53587.2 RecName: Full=Succinate--CoA ligase [GDP-forming] subunit beta, hydrogenosomal; AltName: Full=Succinyl-CoA synthetase beta chain; Short=SCS-beta; Flags: Precursor && PF00549:CoA-ligase\|PF08442:ATP-grasp domain |
| A05249 | Contig2 | 2157065 | 2161183 | + | 3006 |  | 6.79 | 8.38 | 8.42 | 22.02 | 18.28 | 12.70 | -1.17 | 0.000 | - && Q03516.1 RecName: Full=Uncharacterized protein RSN1; AltName: Full=Rescuer of SRO7 at high Nacl protein 1 && PF02714:Calcium-dependent channel, 7TM region, putative phosphate\|PF14703:Cytosolic domain of 10TM putative phosphate transporter\|PF13967:Late exocytosis, associated with Golgi transport\|PF12621:Extracellular tail, of 10TM putative phosphate transporter |
| A05262 | Contig2 | 2194358 | 2195283 | + | 807 |  | 35.43 | 42.54 | 34.52 | 163.24 | 86.53 | 76.88 | -1.54 | 0.000 | - && - && - |
| A05267 | Contig2 | 2217496 | 2225141 | - | 5682 |  | 26.26 | 27.76 | 36.69 | 11.73 | 15.16 | 13.92 | 1.15 | 0.000 | - && Q6NRP2.1 RecName: Full=Proteasome activator complex subunit 4; AltName: Full=Proteasome activator PA200 && PF16507:Proteasome-substrate-size regulator, mid region\|PF11919:Domain of unknown function (DUF3437) |
| A05288 | Contig2 | 2289834 | 2290488 | + | 543 |  | 273.29 | 244.59 | 243.94 | 600.23 | 916.80 | 835.89 | -1.63 | 0.000 | - && P35200.1 RecName: Full=Protein UPS2, mitochondrial; AltName: Full=Altered inheritance rate of mitochondrion protein 30; AltName: Full=Genetic interactor of prohibitins protein 1; AltName: Full=Unprocessed MGM1 protein 2 && PF04707:PRELI-like family |
| A05296 | Contig2 | 2320279 | 2325904 | + | 4401 |  | 24.56 | 32.80 | 40.62 | 69.81 | 57.41 | 70.01 | -1.01 | 0.000 | - && Q9UT00.1 RecName: Full=Uncharacterized protein PYUK71.03c && PF00168:C2 domain |
| A05297 | Contig2 | 2326155 | 2328903 | - | 2184 |  | 23.43 | 25.28 | 24.43 | 49.49 | 43.72 | 60.39 | -1.07 | 0.000 | - && Q4PC17.1 RecName: Full=Multiple RNA-binding domain-containing protein 1 && PF00076:RNA recognition motif. (a.k.a. RRM, RBD, or RNP domain)\|PF14259:RNA recognition motif (a.k.a. RRM, RBD, or RNP domain) |
| A05302 | Contig2 | 2345381 | 2346697 | - | 1140 |  | 89.43 | 126.10 | 59.62 | 9.92 | 7.06 | 14.55 | 3.13 | 0.000 | - && Q12358.1 RecName: Full=Alpha-ketoglutarate-dependent sulfonate dioxygenase && PF02668:Taurine catabolism dioxygenase TauD, TfdA family |
| A05324 | Contig2 | 2427199 | 2428197 | + | 843 |  | 262.71 | 292.83 | 210.01 | 38.37 | 29.09 | 47.47 | 2.74 | 0.000 | - && - && - |
| A05326 | Contig2 | 2430667 | 2431386 | - | 468 |  | 175.93 | 241.53 | 77.88 | 45.55 | 43.60 | 30.38 | 2.05 | 0.000 | - && - && - |
| A05327 | Contig2 | 2431537 | 2432433 | - | 897 |  | 97.94 | 119.86 | 61.00 | 19.91 | 19.41 | 22.60 | 2.17 | 0.000 | - && - && PF12937:F-box-like |
| A05336 | Contig2 | 2465059 | 2466532 | - | 1233 |  | 689.74 | 650.69 | 453.79 | 237.72 | 187.22 | 199.93 | 1.52 | 0.000 | - && - && - |
| A05344 | Contig2 | 2487989 | 2488651 | - | 663 |  | 9.37 | 13.17 | 9.51 | 22.00 | 27.96 | 18.93 | -1.10 | 0.000 | - && - && PF13460:NAD(P)H-binding |
| A05351 | Contig2 | 2501878 | 2502539 | + | 414 |  | 13.81 | 38.55 | 17.43 | 106.37 | 79.14 | 34.34 | -1.66 | 0.000 | - && - && - |
| A05353 | Contig2 | 2509687 | 2512245 | - | 2379 |  | 18.03 | 20.34 | 18.70 | 36.55 | 30.89 | 48.62 | -1.02 | 0.000 | - && Q04177.1 RecName: Full=U3 small nucleolar RNA-associated protein 5; Short=U3 snoRNA-associated protein 5; AltName: Full=U three protein 5; AltName: Full=U3 protein 5 required for transcription; AltName: Full=t-UTP5 && PF04003:Dip2/Utp12 Family |
| A05357 | Contig2 | 2518437 | 2520549 | + | 1419 |  | 31.27 | 31.84 | 21.12 | 15.62 | 13.19 | 11.75 | 1.05 | 0.000 | - && O74852.1 RecName: Full=Uncharacterized MFS-type transporter C18.02 && PF07690:Major Facilitator Superfamily |
| A05360 | Contig2 | 2534762 | 2540526 | + | 3615 |  | 46.21 | 41.07 | 35.15 | 8.38 | 8.36 | 7.87 | 2.31 | 0.000 | - && Q6FTN2.1 RecName: Full=Mitochondrial dicarboxylate transporter; AltName: Full=Dicarboxylate carrier 1 && PF00153:Mitochondrial carrier protein |
| A05393 | Contig2 | 2679240 | 2680533 | + | 1026 |  | 5.77 | 7.04 | 7.03 | 44.47 | 54.38 | 52.69 | -2.93 | 0.000 | - && P83692.1 RecName: Full=Arabinogalactan endo-beta-1,4-galactanase; AltName: Full=Endo-1,4-beta-galactanase; Short=Galactanase && PF07745:Glycosyl hydrolase family 53 |
| A05396 | Contig2 | 2682800 | 2683404 | + | 486 |  | 30.64 | 42.55 | 15.04 | 3.66 | 5.39 | 3.07 | 2.86 | 0.000 | - && - && - |
| A05406 | Contig2 | 266474 | 267347 | - | 714 |  | 70.02 | 146.93 | 123.33 | 530.73 | 596.01 | 212.51 | -1.98 | 0.000 | - && - && - |
| A05423 | Contig2 | 2769134 | 2771187 | - | 1815 |  | 14.94 | 15.10 | 12.88 | 27.82 | 26.72 | 34.43 | -1.05 | 0.000 | - && Q9FMU5.1 RecName: Full=U3 small nucleolar RNA-associated protein 18 homolog && - |
| A05427 | Contig2 | 2783125 | 2785229 | - | 1989 |  | 73.57 | 96.86 | 66.50 | 180.97 | 160.86 | 192.33 | -1.17 | 0.000 | - && Q9FKF6.1 RecName: Full=Cyclin-U4-3; Short=CycU4;3; AltName: Full=Cyclin-P4.2; Short=CycP4;2 && PF08613:Cyclin |
| A05439 | Contig2 | 275407 | 276990 | - | 1233 |  | 23.51 | 34.93 | 20.97 | 35.87 | 51.17 | 109.89 | -1.31 | 0.000 | - && B8N8Q9.1 RecName: Full=NADPH dehydrogenase afvA; AltName: Full=Aflavarin synthesis protein A && PF00724:NADH:flavin oxidoreductase / NADH oxidase family |
| A05440 | Contig2 | 2824165 | 2826609 | + | 1416 |  | 19.92 | 20.84 | 21.61 | 69.73 | 58.51 | 69.79 | -1.67 | 0.000 | - && Q9USJ3.1 RecName: Full=Uncharacterized protein C4B3.03c; Flags: Precursor && PF01595:Domain of unknown function DUF21 |
| A05450 | Contig2 | 277899 | 279052 | + | 924 |  | 2.77 | 4.02 | 4.94 | 8.09 | 13.37 | 6.84 | -1.27 | 0.001 | - && Q8K274.2 RecName: Full=Ketosamine-3-kinase; AltName: Full=Fructosamine-3-kinase-related protein && PF03881:Fructosamine kinase |
| A05451 | Contig2 | 2870433 | 2871153 | + | 537 |  | 1287.90 | 1106.89 | 1095.35 | 528.59 | 373.92 | 168.65 | 1.70 | 0.000 | - && - && PF01476:LysM domain |
| A05454 | Contig2 | 2874796 | 2876269 | + | 1092 |  | 127.95 | 113.26 | 210.05 | 739.06 | 1013.77 | 2093.43 | -3.09 | 0.000 | - && B6Q5I3.1 RecName: Full=Nitrilase && PF00795:Carbon-nitrogen hydrolase |
| A05459 | Contig2 | 2891340 | 2891930 | - | 540 |  | 20.82 | 18.03 | 23.01 | 5.89 | 15.60 | 7.80 | 1.08 | 0.003 | - && - && PF01476:LysM domain |
| A05460 | Contig2 | 2892866 | 2893708 | - | 612 |  | 627.55 | 381.70 | 624.52 | 204.56 | 325.34 | 184.69 | 1.19 | 0.000 | - && - && - |
| A05468 | Contig2 | 2912462 | 2913580 | - | 696 |  | 54.54 | 45.15 | 51.84 | 128.69 | 118.36 | 158.24 | -1.42 | 0.000 | - && - && - |
| A05470 | Contig2 | 2919078 | 2923504 | - | 3618 |  | 134.22 | 90.23 | 192.80 | 42.90 | 55.34 | 41.33 | 1.58 | 0.000 | - && Q86VP6.2 RecName: Full=Cullin-associated NEDD8-dissociated protein 1; AltName: Full=Cullin-associated and neddylation-dissociated protein 1; AltName: Full=TBP-interacting protein of 120 kDa A; Short=TBP-interacting protein 120A; AltName: Full=p120 CAND1 >A7MBJ5.1 RecName: Full=Cullin-associated NEDD8-dissociated protein 1; AltName: Full=Cullin-associated and neddylation-dissociated protein 1 && PF08623:TATA-binding protein interacting (TIP20) |
| A05471 | Contig2 | 2924534 | 2926714 | + | 1776 |  | 91.94 | 54.04 | 144.28 | 29.95 | 49.34 | 24.51 | 1.48 | 0.000 | - && Q6NKX1.1 RecName: Full=Proline dehydrogenase 2, mitochondrial; AltName: Full=Osmotic stress-induced proline dehydrogenase; AltName: Full=Proline oxidase; Flags: Precursor && PF01619:Proline dehydrogenase |
| A05473 | Contig2 | 2927857 | 2929868 | + | 1611 |  | 154.61 | 141.02 | 119.36 | 61.11 | 27.25 | 41.13 | 1.68 | 0.000 | - && Q6NKX1.1 RecName: Full=Proline dehydrogenase 2, mitochondrial; AltName: Full=Osmotic stress-induced proline dehydrogenase; AltName: Full=Proline oxidase; Flags: Precursor && PF01619:Proline dehydrogenase |
| A05479 | Contig2 | 2954863 | 2957581 | + | 1584 |  | 58.27 | 71.30 | 57.32 | 24.20 | 20.51 | 14.85 | 1.65 | 0.000 | - && P08158.1 RecName: Full=Acetamidase && PF01425:Amidase |
| A05480 | Contig2 | 2957808 | 2961442 | - | 2346 |  | 31.10 | 26.53 | 21.84 | 12.47 | 11.33 | 12.16 | 1.14 | 0.000 | - && - && PF01266:FAD dependent oxidoreductase |
| A05486 | Contig2 | 2986443 | 2988029 | - | 1278 |  | 2847.45 | 2903.72 | 2806.04 | 910.23 | 1232.54 | 726.24 | 1.58 | 0.000 | - && - && - |
| A05514 | Contig2 | 3079202 | 3080048 | + | 618 |  | 15.64 | 12.67 | 17.44 | 6.35 | 5.91 | 7.67 | 1.20 | 0.000 | - && - && - |
| A05529 | Contig2 | 3136644 | 3137645 | - | 951 |  | 4.98 | 7.07 | 3.17 | 11.01 | 13.09 | 12.83 | -1.28 | 0.000 | - && - && - |
| A05544 | Contig2 | 3203156 | 3204231 | - | 822 |  | 123.19 | 162.18 | 54.68 | 4.78 | 5.92 | 3.52 | 4.58 | 0.000 | - && - && - |
| A05545 | Contig2 | 3205134 | 3208899 | - | 1428 |  | 480.17 | 674.53 | 319.15 | 28.02 | 17.83 | 16.47 | 4.56 | 0.000 | - && C3K630.1 RecName: Full=L-amino acid amidase && PF11807:Domain of unknown function (DUF3328)\|PF12697:Alpha/beta hydrolase family |
| A05546 | Contig2 | 3209517 | 3210154 | + | 588 |  | 56.68 | 50.02 | 44.28 | 9.22 | 8.12 | 4.63 | 2.78 | 0.000 | - && - && PF11807:Domain of unknown function (DUF3328) |
| A05547 | Contig2 | 3210269 | 3211332 | - | 900 |  | 88.74 | 87.78 | 94.39 | 31.17 | 32.35 | 24.08 | 1.63 | 0.000 | - && - && - |
| A05548 | Contig2 | 3211361 | 3211597 | - | 237 |  | 89.45 | 102.08 | 87.11 | 22.49 | 29.62 | 26.29 | 1.83 | 0.000 | - && - && - |
| A05549 | Contig2 | 3213942 | 3216865 | - | 1809 |  | 12.81 | 11.99 | 11.97 | 3.57 | 8.59 | 4.71 | 1.12 | 0.000 | - && C3K630.1 RecName: Full=L-amino acid amidase && PF00561:alpha/beta hydrolase fold |
| A05556 | Contig2 | 3240980 | 3241928 | + | 885 |  | 9.92 | 9.98 | 8.77 | 3.80 | 4.02 | 4.26 | 1.25 | 0.000 | - && C3K630.1 RecName: Full=L-amino acid amidase && PF00561:alpha/beta hydrolase fold |
| A05560 | Contig2 | 3251565 | 3252783 | - | 996 |  | 386.79 | 404.27 | 334.40 | 191.12 | 165.42 | 77.63 | 1.37 | 0.000 | - && - && - |
| A05570 | Contig2 | 3281749 | 3286331 | + | 2091 |  | 678.53 | 357.05 | 192.05 | 11.27 | 11.82 | 8.48 | 5.28 | 0.000 | - && Q921Q3.3 RecName: Full=Chitobiosyldiphosphodolichol beta-mannosyltransferase; AltName: Full=Asparagine-linked glycosylation protein 1 homolog; AltName: Full=Beta-1,4-mannosyltransferase; AltName: Full=GDP-Man:GlcNAc2-PP-dolichol mannosyltransferase; AltName: Full=GDP-mannose-dolichol diphosphochitobiose mannosyltransferase && PF13579:Glycosyl transferase 4-like domain |
| A05598 | Contig2 | 3372154 | 3377877 | + | 3213 |  | 10.74 | 26.09 | 12.05 | 90.50 | 78.66 | 58.75 | -2.22 | 0.000 | - && O50017.1 RecName: Full=Poly [ADP-ribose] polymerase 2; Short=PARP-2; AltName: Full=NAD(+) ADP-ribosyltransferase 2; Short=ADPRT-2; AltName: Full=Poly[ADP-ribose] synthase 2 && PF02877:Poly(ADP-ribose) polymerase, regulatory domain\|PF00644:Poly(ADP-ribose) polymerase catalytic domain\|PF02469:Fasciclin domain\|PF05406:WGR domain |
| A05599 | Contig2 | 3378509 | 3379342 | + | 834 |  | 5.20 | 4.21 | 4.27 | 10.54 | 9.88 | 8.73 | -1.09 | 0.000 | - && - && - |
| A05601 | Contig2 | 3382021 | 3385523 | - | 1857 |  | 12.11 | 7.95 | 11.12 | 4.23 | 4.03 | 4.21 | 1.32 | 0.000 | - && - && PF00651:BTB/POZ domain |
| A05602 | Contig2 | 3385858 | 3386432 | - | 456 |  | 7203.86 | 4372.83 | 7271.12 | 1694.20 | 2084.88 | 613.54 | 2.10 | 0.000 | - && - && - |
| A05634 | Contig30 | 48407 | 50879 | - | 1785 |  | 0.94 | 1.12 | 0.87 | 2.04 | 2.73 | 1.62 | -1.12 | 0.009 | - && P50276.1 RecName: Full=High-affinity methionine permease && PF13520:Amino acid permease |
| A05648 | Contig30 | 131656 | 134440 | + | 1908 |  | 1.96 | 2.05 | 2.39 | 14.55 | 8.98 | 14.40 | -2.56 | 0.000 | - && P09401.2 RecName: Full=Streptomycin-6-phosphate phosphatase; Flags: Precursor && PF00245:Alkaline phosphatase |
| A05667 | Contig31 | 87523 | 89959 | - | 2211 |  | 6.15 | 6.90 | 9.54 | 15.98 | 15.24 | 14.57 | -1.02 | 0.000 | - && - && - |
| A05680 | Contig31 | 158633 | 160959 | - | 1677 |  | 1.41 | 0.96 | 1.53 | 5.07 | 6.36 | 6.07 | -2.17 | 0.000 | - && Q0CVX4.2 RecName: Full=Probable alpha-galactosidase D; AltName: Full=Melibiase D; Flags: Precursor && PF16499:Alpha galactosidase A |
| A05686 | Contig31 | 194984 | 197516 | + | 2289 |  | 2.54 | 1.05 | 1.56 | 0.65 | 0.78 | 0.84 | 1.18 | 0.010 | - && - && - |
| A05691 | Contig31 | 220780 | 223467 | - | 2457 |  | 11.56 | 9.93 | 10.52 | 5.29 | 4.65 | 5.00 | 1.10 | 0.000 | - && - && - |
| A05708 | Contig32 | 40707 | 41360 | - | 654 |  | 4.22 | 3.68 | 5.03 | 1.29 | 2.00 | 2.42 | 1.18 | 0.014 | - && - && - |
| A05720 | Contig32 | 72805 | 75450 | - | 663 |  | 0.00 | 0.45 | 0.00 | 1.27 | 0.71 | 0.79 | -2.61 | 0.047 | - && Q9S3M0.1 RecName: Full=Cytidine deaminase; Short=CDA; AltName: Full=Cytidine aminohydrolase && PF00383:Cytidine and deoxycytidylate deaminase zinc-binding region |
| A05725 | Contig32 | 89950 | 90527 | - | 405 |  | 731.12 | 617.95 | 524.42 | 69.49 | 53.16 | 61.32 | 3.35 | 0.000 | - && P86242.2 RecName: Full=Papain inhibitor; Short=SPI; Flags: Precursor && PF03330:Rare lipoprotein A (RlpA)-like double-psi beta-barrel |
| A05731 | Contig32 | 116000 | 116449 | + | 399 |  | 238.72 | 208.32 | 387.84 | 84.83 | 112.61 | 71.70 | 1.63 | 0.000 | - && - && - |
| A05732 | Contig32 | 116523 | 117329 | + | 807 |  | 163.36 | 124.40 | 224.13 | 59.43 | 90.01 | 42.63 | 1.41 | 0.000 | - && - && - |
| A05751 | Contig32 | 220884 | 224622 | + | 2760 |  | 27.08 | 29.32 | 24.00 | 79.34 | 85.10 | 98.09 | -1.71 | 0.000 | - && O13923.1 RecName: Full=Coronin-like protein crn1 && PF08953:Domain of unknown function (DUF1899)\|PF16300:Type of WD40 repeat\|PF01400:Astacin (Peptidase family M12A) |
| A05755 | Contig32 | 246642 | 247389 | + | 633 |  | 2.18 | 1.74 | 5.77 | 1.33 | 1.48 | 0.69 | 1.47 | 0.025 | - && - && - |
| A05764 | Contig33 | 29863 | 32176 | - | 2100 |  | 2.72 | 2.53 | 1.35 | 7.92 | 8.65 | 6.90 | -1.83 | 0.000 | - && - && - |
| A05776 | Contig33 | 85550 | 92047 | + | 2916 |  | 4.90 | 4.65 | 4.61 | 12.34 | 27.51 | 12.37 | -1.88 | 0.000 | - && B8NHY4.1 RecName: Full=O-methylsterigmatocystin oxidoreductase; Short=OMST oxidoreductase; AltName: Full=Aflatoxin B synthase; AltName: Full=Aflatoxin biosynthesis protein Q; AltName: Full=Cytochrome P450 64 >P0CT93.1 RecName: Full=O-methylsterigmatocystin oxidoreductase; Short=OMST oxidoreductase; AltName: Full=Aflatoxin B synthase; AltName: Full=Aflatoxin biosynthesis protein Q; AltName: Full=Cytochrome P450 64 && PF00067:Cytochrome P450 |
| A05792 | Contig33 | 135148 | 136605 | + | 1170 |  | 7.50 | 7.64 | 9.45 | 22.61 | 20.56 | 23.18 | -1.43 | 0.000 | - && Q54L00.1 RecName: Full=Probable LIM domain-containing serine/threonine-protein kinase DDB_G0287001 && PF07714:Protein tyrosine kinase |
| A05801 | Contig33 | 164843 | 166489 | + | 1191 |  | 9.69 | 9.61 | 9.36 | 6.12 | 4.40 | 3.09 | 1.07 | 0.000 | - && - && PF00069:Protein kinase domain |
| A05823 | Contig34 | 16857 | 17699 | + | 843 |  | 12.52 | 14.77 | 18.20 | 37.15 | 60.07 | 48.82 | -1.68 | 0.000 | - && - && - |
| A05834 | Contig34 | 56475 | 57149 | + | 492 |  | 15.83 | 19.38 | 7.80 | 8.17 | 5.71 | 6.24 | 1.10 | 0.005 | - && - && - |
| A05851 | Contig34 | 110462 | 111478 | + | 831 |  | 0.12 | 0.12 | 0.00 | 0.90 | 0.34 | 0.63 | -2.97 | 0.040 | - && Q9UUA7.1 RecName: Full=UPF0590 protein C409.17c && PF08588:Protein of unknown function (DUF1769) |
| A05903 | Contig35 | 74749 | 78932 | - | 1008 |  | 14.97 | 11.45 | 9.79 | 2.97 | 4.27 | 2.44 | 1.90 | 0.000 | - && Q9D187.1 RecName: Full=Mitotic spindle-associated MMXD complex subunit MIP18; AltName: Full=Protein FAM96B && - |
| A05907 | Contig35 | 94677 | 98688 | - | 2001 |  | 23.65 | 23.78 | 19.36 | 82.70 | 76.35 | 83.55 | -1.86 | 0.000 | - && - && PF12697:Alpha/beta hydrolase family |
| A05922 | Contig35 | 159243 | 160934 | + | 1206 |  | 1.96 | 1.75 | 2.58 | 5.97 | 7.68 | 6.33 | -1.67 | 0.000 | - && Q8E372.1 RecName: Full=Unsaturated chondroitin disaccharide hydrolase; AltName: Full=Unsaturated glucuronyl hydrolase; Short=SagUGL && PF07470:Glycosyl Hydrolase Family 88 |
| A05926 | Contig35 | 178263 | 181613 | - | 2682 |  | 18.75 | 15.01 | 24.08 | 45.18 | 31.06 | 46.79 | -1.09 | 0.000 | - && - && - |
| A05936 | Contig36 | 47120 | 48845 | + | 1446 |  | 2.52 | 3.26 | 4.04 | 5.56 | 8.42 | 7.95 | -1.16 | 0.000 | - && Q566I3.2 RecName: Full=Hypoxia up-regulated protein 1; Flags: Precursor && PF00012:Hsp70 protein |
| A05949 | Contig36 | 144772 | 147765 | + | 2562 |  | 20.40 | 24.33 | 20.72 | 48.83 | 38.36 | 59.33 | -1.16 | 0.000 | - && Q566I3.2 RecName: Full=Hypoxia up-regulated protein 1; Flags: Precursor && PF00012:Hsp70 protein |
| A05955 | Contig36 | 169332 | 170510 | - | 1023 |  | 2.99 | 3.93 | 2.50 | 5.76 | 8.33 | 10.04 | -1.36 | 0.000 | - && - && - |
| A05957 | Contig37 | 2860 | 6487 | + | 2466 |  | 0.28 | 0.77 | 0.33 | 0.11 | 0.08 | 0.04 | 2.62 | 0.005 | - && - && - |
| A05968 | Contig37 | 33648 | 37410 | + | 2673 |  | 32.28 | 33.01 | 27.75 | 12.94 | 11.31 | 11.26 | 1.39 | 0.000 | - && P0CS15.1 RecName: Full=DNA damage-inducible protein 1 >P0CS14.1 RecName: Full=DNA damage-inducible protein 1 && PF09668:Aspartyl protease |
| A05970 | Contig37 | 39733 | 41366 | + | 1578 |  | 0.75 | 1.02 | 0.69 | 3.20 | 1.07 | 2.78 | -1.52 | 0.004 | - && P13860.1 RecName: Full=Exoglucanase 1; AltName: Full=1,4-beta-cellobiohydrolase; AltName: Full=Exocellobiohydrolase I; AltName: Full=Exoglucanase I; Flags: Precursor && PF00734:Fungal cellulose binding domain\|PF00840:Glycosyl hydrolase family 7 |
| A05971 | Contig37 | 41698 | 42779 | - | 855 |  | 1.85 | 1.29 | 1.28 | 0.44 | 0.77 | 0.41 | 1.45 | 0.041 | - && - && PF11807:Domain of unknown function (DUF3328) |
| A05995 | Contig37 | 131511 | 134944 | - | 1968 |  | 1.10 | 6.07 | 1.58 | 1.24 | 1.38 | 1.03 | 1.27 | 0.024 | - && Q3TQI7.2 RecName: Full=Uncharacterized protein C9orf78 homolog && PF07052:Hepatocellular carcinoma-associated antigen 59 |
| A06016 | Contig39 | 91073 | 92532 | - | 978 |  | 9.78 | 5.75 | 4.02 | 12.43 | 13.30 | 14.90 | -1.06 | 0.001 | - && Q9UTE6.1 RecName: Full=Protein mak16 && PF04874:Mak16 protein C-terminal region\|PF01778:Ribosomal L28e protein family |
| A06027 | Contig3 | 373510 | 375843 | - | 1584 |  | 0.56 | 1.77 | 1.44 | 6.61 | 2.48 | 2.83 | -1.66 | 0.001 | - && A2QQV6.1 RecName: Full=Probable quinate permease; AltName: Full=Quinate transporter && PF00083:Sugar (and other) transporter |
| A06028 | Contig3 | 376608 | 381494 | - | 2652 |  | 8.22 | 10.56 | 7.10 | 57.64 | 66.33 | 48.41 | -2.74 | 0.000 | - && O74923.1 RecName: Full=Uncharacterized transporter C757.13 && PF07859:alpha/beta hydrolase fold\|PF07690:Major Facilitator Superfamily |
| A06035 | Contig3 | 407914 | 408941 | + | 858 |  | 9.42 | 10.76 | 5.32 | 27.90 | 28.58 | 23.12 | -1.64 | 0.000 | - && - && PF01370:NAD dependent epimerase/dehydratase family |
| A06041 | Contig3 | 424191 | 430127 | - | 3900 |  | 45.56 | 36.04 | 54.18 | 127.27 | 126.28 | 120.57 | -1.46 | 0.000 | - && - && - |
| A06043 | Contig3 | 438247 | 439542 | - | 1161 |  | 2.29 | 2.94 | 1.73 | 1.29 | 1.05 | 0.23 | 1.44 | 0.014 | - && - && PF05368:NmrA-like family |
| A06054 | Contig3 | 467799 | 469183 | - | 1035 |  | 37.35 | 82.25 | 32.48 | 9.94 | 7.15 | 21.45 | 1.98 | 0.000 | - && - && PF05368:NmrA-like family |
| A06061 | Contig3 | 504177 | 505545 | + | 1158 |  | 1220.87 | 866.30 | 725.43 | 475.63 | 317.05 | 335.65 | 1.32 | 0.000 | - && Q9D832.1 RecName: Full=DnaJ homolog subfamily B member 4 && PF00226:DnaJ domain\|PF01556:DnaJ C terminal domain |
| A06074 | Contig3 | 564337 | 570338 | + | 2460 |  | 119.41 | 87.62 | 101.38 | 42.91 | 29.61 | 45.73 | 1.38 | 0.000 | - && - && PF11951:Fungal specific transcription factor domain |
| A06079 | Contig3 | 586790 | 588193 | - | 1047 |  | 6.12 | 3.16 | 4.80 | 22.06 | 65.54 | 7.46 | -2.75 | 0.002 | - && O60206.1 RecName: Full=Endo-1,4-beta-xylanase; Short=Xylanase; Flags: Precursor && PF00331:Glycosyl hydrolase family 10 |
| A06081 | Contig3 | 589943 | 591333 | + | 1044 |  | 5.19 | 9.13 | 6.21 | 16.48 | 15.42 | 12.44 | -1.11 | 0.000 | - && - && PF05368:NmrA-like family |
| A06088 | Contig3 | 613454 | 616702 | + | 2511 |  | 48.42 | 30.14 | 55.33 | 17.35 | 15.02 | 13.81 | 1.54 | 0.000 | - && Q9TTK6.3 RecName: Full=Membrane primary amine oxidase; AltName: Full=Copper amine oxidase; AltName: Full=Semicarbazide-sensitive amine oxidase; Short=SSAO; AltName: Full=Vascular adhesion protein 1; Short=VAP-1 && PF01179:Copper amine oxidase, enzyme domain\|PF09248:Domain of unknown function (DUF1965) |
| A06094 | Contig3 | 644794 | 645344 | + | 501 |  | 1.77 | 1.80 | 1.64 | 3.36 | 4.67 | 3.68 | -1.17 | 0.035 | - && - && - |
| A06103 | Contig3 | 679430 | 680877 | + | 1083 |  | 0.82 | 0.83 | 2.02 | 3.63 | 4.32 | 2.76 | -1.54 | 0.002 | - && - && - |
| A06115 | Contig3 | 716112 | 722989 | - | 2913 |  | 153.54 | 116.41 | 144.35 | 75.36 | 62.09 | 65.68 | 1.03 | 0.000 | - && O42783.2 RecName: Full=Neutral trehalase; AltName: Full=Alpha,alpha-trehalase; AltName: Full=Alpha,alpha-trehalose glucohydrolase && PF07492:Neutral trehalase Ca2+ binding domain\|PF01204:Trehalase |
| A06132 | Contig3 | 767045 | 768651 | - | 1296 |  | 329.21 | 217.35 | 489.67 | 80.29 | 147.35 | 86.40 | 1.72 | 0.000 | - && Q8K157.1 RecName: Full=Aldose 1-epimerase; AltName: Full=Galactose mutarotase && PF01263:Aldose 1-epimerase |
| A06133 | Contig3 | 769100 | 771195 | + | 1683 |  | 337.11 | 151.39 | 692.53 | 42.16 | 123.37 | 30.92 | 2.59 | 0.003 | - && Q9SEE5.2 RecName: Full=Galactokinase; AltName: Full=Galactose kinase && PF00288:GHMP kinases N terminal domain\|PF08544:GHMP kinases C terminal\|PF10509:Galactokinase galactose-binding signature |
| A06137 | Contig3 | 775907 | 777184 | + | 1056 |  | 117.93 | 158.38 | 83.56 | 11.95 | 12.41 | 11.55 | 3.32 | 0.000 | - && Q84V25.1 RecName: Full=2-methylene-furan-3-one reductase; AltName: Full=Enone oxidoreductase; Short=FaEO; AltName: Full=Quinone oxidoreductase; Short=FaQR && PF00107:Zinc-binding dehydrogenase\|PF08240:Alcohol dehydrogenase GroES-like domain |
| A06138 | Contig3 | 777465 | 779665 | + | 1362 |  | 30.77 | 27.93 | 23.88 | 96.04 | 128.73 | 84.09 | -1.90 | 0.000 | - && G3XMC6.1 RecName: Full=Dehydrogenase azaJ; AltName: Full=Azaphilone biosynthesis cluster protein azaJ && PF08240:Alcohol dehydrogenase GroES-like domain\|PF00107:Zinc-binding dehydrogenase |
| A06141 | Contig3 | 785210 | 786395 | - | 630 |  | 4.07 | 9.40 | 6.96 | 12.47 | 14.86 | 18.81 | -1.17 | 0.001 | - && - && PF07106:Tat binding protein 1(TBP-1)-interacting protein (TBPIP) |
| A06142 | Contig3 | 786896 | 788265 | + | 1020 |  | 22.14 | 26.47 | 23.55 | 40.42 | 38.27 | 66.59 | -1.01 | 0.000 | - && P38702.1 RecName: Full=Mitochondrial carrier protein LEU5 && PF00153:Mitochondrial carrier protein |
| A06145 | Contig3 | 797340 | 798656 | + | 1104 |  | 57.43 | 37.01 | 62.31 | 24.98 | 20.43 | 19.55 | 1.27 | 0.000 | - && - && - |
| A06150 | Contig3 | 811937 | 812535 | - | 477 |  | 78.76 | 67.14 | 116.25 | 42.73 | 36.70 | 35.51 | 1.19 | 0.000 | - && - && PF12680:SnoaL-like domain |
| A06151 | Contig3 | 813540 | 814142 | - | 480 |  | 15.20 | 12.55 | 17.70 | 52.59 | 37.25 | 41.50 | -1.53 | 0.000 | - && - && - |
| A06153 | Contig3 | 816123 | 816719 | - | 477 |  | 274.52 | 305.16 | 304.50 | 84.09 | 67.51 | 83.53 | 1.91 | 0.000 | - && - && - |
| A06162 | Contig3 | 834397 | 835797 | - | 1128 |  | 27.71 | 27.50 | 28.02 | 14.75 | 11.78 | 14.63 | 1.02 | 0.000 | - && P0CL92.1 RecName: Full=Iron-sulfur clusters transporter ATM1, mitochondrial; Flags: Precursor && PF00664:ABC transporter transmembrane region |
| A06163 | Contig3 | 837534 | 839055 | - | 867 |  | 140.57 | 147.98 | 119.27 | 66.64 | 58.09 | 53.95 | 1.19 | 0.000 | - && - && - |
| A06164 | Contig3 | 840981 | 842975 | + | 1152 |  | 247.62 | 229.36 | 277.54 | 130.99 | 113.76 | 73.82 | 1.24 | 0.000 | - && - && PF01753:MYND finger |
| A06181 | Contig3 | 891784 | 893896 | + | 1554 |  | 46.89 | 41.47 | 27.51 | 8.42 | 4.52 | 3.67 | 2.80 | 0.000 | - && Q9LMF0.1 RecName: Full=UDP-glycosyltransferase 85A5 && PF00201:UDP-glucoronosyl and UDP-glucosyl transferase |
| A06182 | Contig3 | 894675 | 897009 | + | 1641 |  | 117.47 | 114.21 | 81.05 | 38.29 | 15.69 | 46.31 | 1.64 | 0.000 | - && O82382.1 RecName: Full=UDP-glycosyltransferase 71C2 && PF00201:UDP-glucoronosyl and UDP-glucosyl transferase |
| A06210 | Contig3 | 995632 | 1000654 | - | 2748 |  | 3.48 | 4.49 | 5.65 | 25.08 | 27.32 | 26.28 | -2.53 | 0.000 | - && - && PF01926:50S ribosome-binding GTPase\|PF01823:MAC/Perforin domain |
| A06214 | Contig3 | 1003923 | 1004732 | - | 633 |  | 21.50 | 24.74 | 29.73 | 12.70 | 16.71 | 7.63 | 1.04 | 0.001 | - && - && - |
| A06221 | Contig3 | 1020812 | 1022541 | - | 1080 |  | 50.49 | 60.42 | 45.84 | 14.46 | 11.27 | 7.31 | 2.25 | 0.000 | - && O59826.1 RecName: Full=Putative voltage-gated potassium channel subunit beta; AltName: Full=K(+) channel subunit beta && PF00248:Aldo/keto reductase family |
| A06223 | Contig3 | 1030033 | 1030766 | - | 447 |  | 317.43 | 275.79 | 281.20 | 937.05 | 1687.71 | 2248.35 | -2.48 | 0.000 | - && P00048.2 RecName: Full=Cytochrome c && PF00034:Cytochrome c |
| A06229 | Contig3 | 1045181 | 1046938 | + | 1314 |  | 109.78 | 108.56 | 96.77 | 34.22 | 43.46 | 25.65 | 1.61 | 0.000 | - && A7XRY8.1 RecName: Full=Aminotransferase tdiD; AltName: Full=Terrequinone biosynthesis protein D && PF00155:Aminotransferase class I and II |
| A06230 | Contig3 | 1048406 | 1049690 | - | 1062 |  | 13.93 | 12.95 | 16.17 | 3.26 | 3.17 | 1.82 | 2.38 | 0.000 | - && O94038.1 RecName: Full=Alcohol dehydrogenase 2 && PF08240:Alcohol dehydrogenase GroES-like domain\|PF00107:Zinc-binding dehydrogenase |
| A06236 | Contig3 | 1064877 | 1065436 | + | 501 |  | 45.46 | 39.07 | 39.75 | 22.02 | 14.20 | 18.57 | 1.18 | 0.000 | - && - && PF07714:Protein tyrosine kinase |
| A06279 | Contig3 | 1178774 | 1180295 | - | 1230 |  | 5.13 | 6.69 | 10.99 | 4.03 | 4.95 | 2.35 | 1.01 | 0.008 | - && - && - |
| A06281 | Contig3 | 1181411 | 1182904 | - | 1203 |  | 75.82 | 70.76 | 110.41 | 39.48 | 27.31 | 25.97 | 1.47 | 0.000 | - && - && - |
| A06295 | Contig3 | 1237383 | 1238062 | - | 465 |  | 4.24 | 2.59 | 2.16 | 0.80 | 0.60 | 0.94 | 1.93 | 0.009 | - && - && - |
| A06315 | Contig3 | 1324240 | 1326429 | - | 1629 |  | 48.54 | 37.78 | 58.88 | 192.44 | 206.93 | 202.83 | -2.05 | 0.000 | - && Q2U256.1 RecName: Full=Peptidyl-prolyl cis-trans isomerase-like 4; Short=PPIase; AltName: Full=Rotamase && PF00076:RNA recognition motif. (a.k.a. RRM, RBD, or RNP domain)\|PF00651:BTB/POZ domain |
| A06324 | Contig3 | 150644 | 151560 | + | 807 |  | 3.91 | 1.49 | 3.85 | 8.46 | 5.92 | 5.44 | -1.10 | 0.011 | - && P0CU01.1 RecName: Full=Uncharacterized oxidoreductase SPBC1348.09 >P0CU00.1 RecName: Full=Uncharacterized oxidoreductase SPAC977.08 && PF00106:short chain dehydrogenase |
| A06327 | Contig3 | 1354836 | 1355282 | + | 306 |  | 8.38 | 8.53 | 61.50 | 1951.19 | 3081.19 | 620.91 | -6.17 | 0.000 | - && P16934.1 RecName: Full=Fruiting body protein SC4; AltName: Full=Hydrophobin SC4; Flags: Precursor && PF01185:Fungal hydrophobin |
| A06328 | Contig3 | 1357228 | 1357688 | + | 348 |  | 37.40 | 93.75 | 114.45 | 2113.87 | 1966.09 | 1208.70 | -4.43 | 0.000 | - && P16933.2 RecName: Full=Fruiting body protein SC3; AltName: Full=Hydrophobin SC3; Flags: Precursor && PF01185:Fungal hydrophobin |
| A06330 | Contig3 | 1360115 | 1362185 | + | 1785 |  | 17.57 | 15.69 | 19.19 | 37.82 | 28.79 | 50.54 | -1.16 | 0.000 | - && - && - |
| A06334 | Contig3 | 1371650 | 1373069 | - | 1131 |  | 20.05 | 25.03 | 23.26 | 59.77 | 69.36 | 29.95 | -1.22 | 0.000 | - && Q01752.1 RecName: Full=Aryl-alcohol dehydrogenase [NADP(+)]; Short=AAD && PF00248:Aldo/keto reductase family |
| A06335 | Contig3 | 151955 | 153941 | - | 1656 |  | 2.02 | 2.36 | 6.12 | 1.52 | 1.02 | 1.01 | 1.57 | 0.001 | - && - && PF13489:Methyltransferase domain |
| A06336 | Contig3 | 1374178 | 1375934 | + | 1434 |  | 46.69 | 51.94 | 57.21 | 222.00 | 237.55 | 203.48 | -2.09 | 0.000 | - && O00093.2 RecName: Full=3-phytase B; AltName: Full=3 phytase B; AltName: Full=Myo-inositol hexakisphosphate phosphohydrolase B; AltName: Full=Myo-inositol-hexaphosphate 3-phosphohydrolase B; Flags: Precursor && PF00328:Histidine phosphatase superfamily (branch 2) |
| A06345 | Contig3 | 1404531 | 1407859 | + | 2679 |  | 48.07 | 42.87 | 65.84 | 79.01 | 140.96 | 149.90 | -1.24 | 0.000 | - && - && PF04082:Fungal specific transcription factor domain\|PF00172:Fungal Zn(2)-Cys(6) binuclear cluster domain |
| A06350 | Contig3 | 1414571 | 1415952 | + | 1077 |  | 110.50 | 141.31 | 130.96 | 20.05 | 21.73 | 61.68 | 1.89 | 0.000 | - && Q4WZ70.1 RecName: Full=Chanoclavine-I aldehyde reductase easA; AltName: Full=Ergot alkaloid synthesis protein A; AltName: Full=Old yellow enzyme 3 homolog && PF00724:NADH:flavin oxidoreductase / NADH oxidase family |
| A06361 | Contig3 | 1442743 | 1444875 | - | 1695 |  | 10.18 | 11.49 | 19.89 | 71.27 | 46.11 | 84.19 | -2.28 | 0.000 | - && Q873X9.1 RecName: Full=Endochitinase B1; AltName: Full=Chitinase B1; Flags: Precursor >E9QRF2.1 RecName: Full=Endochitinase B1; AltName: Full=Chitinase B1; Flags: Precursor && PF00787:PX domain\|PF00704:Glycosyl hydrolases family 18 |
| A06369 | Contig3 | 161289 | 162788 | + | 1500 |  | 42.14 | 42.36 | 42.69 | 11.41 | 6.99 | 7.37 | 2.30 | 0.000 | - && - && PF06985:Heterokaryon incompatibility protein (HET) |
| A06375 | Contig3 | 1481344 | 1482190 | - | 609 |  | 13.28 | 12.20 | 14.85 | 32.70 | 27.36 | 26.66 | -1.10 | 0.000 | - && - && - |
| A06377 | Contig3 | 1484861 | 1485637 | - | 558 |  | 265.59 | 203.30 | 239.18 | 112.93 | 81.36 | 136.83 | 1.10 | 0.000 | - && - && - |
| A06378 | Contig3 | 1486294 | 1488451 | - | 1587 |  | 9.51 | 7.72 | 8.98 | 128.02 | 88.42 | 148.86 | -3.80 | 0.000 | - && Q92339.1 RecName: Full=High-affinity gluconate transporter ght3; AltName: Full=Hexose transporter 3 && PF00083:Sugar (and other) transporter |
| A06380 | Contig3 | 163149 | 166259 | - | 2451 |  | 157.58 | 113.49 | 289.96 | 62.71 | 99.15 | 60.37 | 1.34 | 0.000 | - && O42938.1 RecName: Full=ATP-dependent 6-phosphofructokinase; Short=ATP-PFK; Short=Phosphofructokinase; AltName: Full=Phosphohexokinase && PF00365:Phosphofructokinase |
| A06390 | Contig3 | 1519832 | 1521003 | - | 984 |  | 3.01 | 4.59 | 4.46 | 7.03 | 11.13 | 5.98 | -1.00 | 0.005 | - && - && - |
| A06397 | Contig3 | 1532453 | 1534111 | + | 1395 |  | 769.95 | 721.93 | 928.30 | 357.96 | 309.48 | 336.57 | 1.27 | 0.000 | - && P82476.3 RecName: Full=Chitin deacetylase; Flags: Precursor && PF01522:Polysaccharide deacetylase |
| A06399 | Contig3 | 1538339 | 1539921 | + | 1332 |  | 317.94 | 289.11 | 257.25 | 32.99 | 21.08 | 27.41 | 3.41 | 0.000 | - && P82476.3 RecName: Full=Chitin deacetylase; Flags: Precursor && PF01522:Polysaccharide deacetylase |
| A06402 | Contig3 | 172139 | 173599 | + | 933 |  | 231.24 | 412.20 | 150.88 | 35.07 | 19.06 | 22.76 | 3.37 | 0.000 | - && O13780.1 RecName: Full=Uncharacterized protein C17G6.02c && PF04479:RTA1 like protein |
| A06403 | Contig3 | 1546776 | 1547433 | + | 594 |  | 13.45 | 12.51 | 9.23 | 25.81 | 26.00 | 24.23 | -1.11 | 0.000 | - && - && - |
| A06412 | Contig3 | 1583227 | 1583720 | - | 321 |  | 14936.97 | 13418.24 | 11435.79 | 1063.99 | 1586.71 | 398.33 | 3.71 | 0.000 | - && - && PF12585:Protein of unknown function (DUF3759) |
| A06413 | Contig3 | 174094 | 175414 | + | 1155 |  | 12.29 | 22.08 | 20.25 | 45.98 | 45.14 | 32.29 | -1.18 | 0.000 | - && - && - |
| A06418 | Contig3 | 1599481 | 1600985 | + | 1137 |  | 121.32 | 104.10 | 111.92 | 17.52 | 20.50 | 10.81 | 2.79 | 0.000 | - && P38891.1 RecName: Full=Branched-chain-amino-acid aminotransferase, mitochondrial; Short=BCAT; AltName: Full=Protein ECA39; AltName: Full=Protein TWT1; Flags: Precursor && PF01063:Amino-transferase class IV |
| A06427 | Contig3 | 1635992 | 1637852 | + | 1581 |  | 20.02 | 19.49 | 20.92 | 37.37 | 35.70 | 58.23 | -1.12 | 0.000 | - && O94365.1 RecName: Full=U3 small nucleolar RNA-associated protein 15; Short=U3 snoRNA-associated protein 15; AltName: Full=U3 protein 15 required for transcription && PF09384:UTP15 C terminal |
| A06463 | Contig3 | 1735816 | 1737048 | - | 1065 |  | 11.20 | 14.52 | 15.10 | 24.84 | 28.39 | 31.72 | -1.06 | 0.000 | - && P53239.1 RecName: Full=Mitochondrial inner membrane protein COX18; AltName: Full=Cytochrome c oxidase assembly protein 18; Flags: Precursor && PF02096:60Kd inner membrane protein |
| A06485 | Contig3 | 1792426 | 1796963 | - | 2721 |  | 9.31 | 11.07 | 9.17 | 47.73 | 73.18 | 57.25 | -2.59 | 0.000 | - && Q2U4L7.2 RecName: Full=Glutaminase A; Flags: Precursor && PF08760:Domain of unknown function (DUF1793)\|PF00215:Orotidine 5'-phosphate decarboxylase / HUMPS family\|PF16334:Domain of unknown function (DUF4964)\|PF16335:Domain of unknown function (DUF4965) |
| A06493 | Contig3 | 1815569 | 1818043 | - | 2025 |  | 38.27 | 36.44 | 37.17 | 17.27 | 21.91 | 16.55 | 1.01 | 0.000 | - && Q9USQ7.1 RecName: Full=Diphthine--ammonia ligase; AltName: Full=Diphthamide synthase; AltName: Full=Diphthamide synthetase && PF01042:Endoribonuclease L-PSP\|PF01902:Diphthamide synthase |
| A06494 | Contig3 | 1818253 | 1821976 | + | 3039 |  | 148.34 | 147.76 | 120.60 | 57.41 | 51.72 | 57.18 | 1.33 | 0.000 | - && O94609.1 RecName: Full=Ubiquitin-activating enzyme E1 1; AltName: Full=Poly(A)+ RNA transport protein 3 && PF00899:ThiF family\|PF10585:Ubiquitin-activating enzyme active site\|PF09358:Ubiquitin fold domain\|PF16190:Ubiquitin-activating enzyme E1 FCCH domain\|PF16191:Ubiquitin-activating enzyme E1 four-helix bundle |
| A06506 | Contig3 | 1853026 | 1853697 | + | 606 |  | 48.81 | 58.81 | 43.72 | 102.14 | 110.60 | 93.84 | -1.02 | 0.000 | - && - && - |
| A06508 | Contig3 | 1857890 | 1859210 | - | 1080 |  | 43.37 | 39.60 | 47.20 | 24.67 | 16.04 | 14.22 | 1.24 | 0.000 | - && Q6FLQ5.1 RecName: Full=Altered inheritance of mitochondria protein 32 && PF06999:Sucrase/ferredoxin-like |
| A06517 | Contig3 | 1885787 | 1887411 | - | 1173 |  | 6.72 | 7.45 | 7.09 | 16.18 | 12.69 | 21.25 | -1.24 | 0.000 | - && D4AMB9.1 RecName: Full=Probable aldose 1-epimerase ARB_05372; AltName: Full=Galactose mutarotase; Flags: Precursor && PF01263:Aldose 1-epimerase |
| A06525 | Contig3 | 1921208 | 1923836 | + | 1356 |  | 204.04 | 346.62 | 163.03 | 73.50 | 66.96 | 75.59 | 1.72 | 0.000 | - && Q9US44.1 RecName: Full=Uncharacterized transporter C1002.16c && PF07690:Major Facilitator Superfamily |
| A06528 | Contig3 | 1935645 | 1943972 | - | 5187 |  | 36.10 | 35.19 | 39.84 | 13.45 | 17.27 | 13.67 | 1.32 | 0.000 | - && Q5HL56.1 RecName: Full=Copper-exporting P-type ATPase A; AltName: Full=Cu(+)-exporting ATPase >Q8CN02.1 RecName: Full=Copper-exporting P-type ATPase A; AltName: Full=Cu(+)-exporting ATPase && PF00501:AMP-binding enzyme\|PF00122:E1-E2 ATPase\|PF13193:AMP-binding enzyme C-terminal domain\|PF00403:Heavy-metal-associated domain\|PF00702:haloacid dehalogenase-like hydrolase |
| A06532 | Contig3 | 1951423 | 1957852 | - | 2805 |  | 34.73 | 32.03 | 33.45 | 11.60 | 9.24 | 9.35 | 1.73 | 0.000 | - && Q12473.1 RecName: Full=Ferric reductase transmembrane component 6; AltName: Full=Ferric-chelate reductase 6; Flags: Precursor && PF08030:Ferric reductase NAD binding domain\|PF08022:FAD-binding domain\|PF01794:Ferric reductase like transmembrane component |
| A06552 | Contig3 | 2010023 | 2012189 | - | 1500 |  | 3.42 | 3.01 | 4.69 | 1.37 | 1.93 | 1.70 | 1.15 | 0.002 | - && G3Y416.1 RecName: Full=Cytochrome P450 monooxygenase yanC; AltName: Full=Yanuthone D synthesis protein C; Flags: Precursor && PF00067:Cytochrome P450 |
| A06553 | Contig3 | 2012953 | 2015146 | - | 1560 |  | 2.84 | 3.02 | 1.93 | 1.56 | 0.96 | 1.13 | 1.10 | 0.008 | - && A1DA63.1 RecName: Full=Fumitremorgin C synthase; AltName: Full=Fumitremorgin biosynthesis protein E && PF00067:Cytochrome P450 |
| A06565 | Contig3 | 2056589 | 2058446 | + | 1455 |  | 2.64 | 3.24 | 3.26 | 56.74 | 76.37 | 60.07 | -4.40 | 0.000 | - && O43000.2 RecName: Full=Pantothenate transporter liz1 && PF07690:Major Facilitator Superfamily |
| A06568 | Contig3 | 233219 | 234850 | - | 1134 |  | 24.43 | 15.49 | 28.11 | 6.02 | 8.17 | 4.26 | 1.88 | 0.000 | - && - && - |
| A06577 | Contig3 | 2078752 | 2081192 | + | 1536 |  | 9.44 | 9.67 | 6.48 | 5.11 | 4.33 | 1.89 | 1.18 | 0.001 | - && P42656.2 RecName: Full=DNA damage checkpoint protein rad24 && PF00244:14-3-3 protein\|PF03443:Glycosyl hydrolase family 61 |
| A06580 | Contig3 | 235654 | 237510 | - | 996 |  | 35.44 | 25.00 | 50.81 | 19.71 | 23.31 | 9.08 | 1.09 | 0.002 | - && - && - |
| A06590 | Contig3 | 2106901 | 2107727 | + | 555 |  | 126.50 | 123.18 | 107.64 | 40.43 | 44.87 | 34.63 | 1.58 | 0.000 | - && Q9X0Y1.1 RecName: Full=Phosphorylated carbohydrates phosphatase TM_1254 && PF13419:Haloacid dehalogenase-like hydrolase |
| A06591 | Contig3 | 240238 | 243391 | + | 2448 |  | 4388.20 | 2081.58 | 5289.18 | 1593.36 | 2162.71 | 824.35 | 1.36 | 0.000 | - && O74770.1 RecName: Full=Probable phosphoketolase && PF03894:D-xylulose 5-phosphate/D-fructose 6-phosphate phosphoketolase\|PF09363:XFP C-terminal domain\|PF09364:XFP N-terminal domain |
| A06600 | Contig3 | 2132821 | 2133948 | - | 762 |  | 43.61 | 33.07 | 29.25 | 13.13 | 8.97 | 10.13 | 1.72 | 0.000 | - && - && - |
| A06605 | Contig3 | 2141809 | 2144000 | + | 1860 |  | 15.69 | 13.12 | 24.36 | 5.73 | 9.76 | 6.79 | 1.25 | 0.000 | - && - && - |
| A06634 | Contig3 | 2231424 | 2233010 | - | 1134 |  | 633.62 | 512.56 | 657.90 | 288.57 | 318.72 | 276.90 | 1.03 | 0.000 | - && A5PJJ7.1 RecName: Full=Short-chain dehydrogenase/reductase family 16C member 6 && PF00106:short chain dehydrogenase |
| A06640 | Contig3 | 2247133 | 2249148 | - | 1503 |  | 99.19 | 38.27 | 87.10 | 25.57 | 37.12 | 19.09 | 1.46 | 0.000 | - && - && - |
| A06657 | Contig3 | 264135 | 264663 | + | 342 |  | 4.90 | 12.62 | 6.68 | 29.25 | 94.43 | 27.46 | -2.64 | 0.000 | - && - && - |
| A06660 | Contig3 | 2316546 | 2318717 | + | 1605 |  | 91.05 | 86.19 | 91.92 | 38.33 | 37.68 | 33.46 | 1.30 | 0.000 | - && O14192.1 RecName: Full=Aromatic amino acid aminotransferase C56E4.03 && PF00155:Aminotransferase class I and II |
| A06704 | Contig3 | 2455390 | 2460959 | - | 2187 |  | 193.01 | 159.14 | 180.57 | 81.57 | 56.03 | 49.16 | 1.51 | 0.000 | - && P56859.3 RecName: Full=Phosphoadenosine phosphosulfate reductase; AltName: Full=3'-phosphoadenylylsulfate reductase; AltName: Full=PAPS reductase, thioredoxin dependent; AltName: Full=PAdoPS reductase && PF07714:Protein tyrosine kinase\|PF00035:Double-stranded RNA binding motif\|PF01507:Phosphoadenosine phosphosulfate reductase family |
| A06716 | Contig3 | 2484269 | 2485115 | + | 618 |  | 147.43 | 170.40 | 194.08 | 69.59 | 48.77 | 103.66 | 1.21 | 0.000 | - && - && PF13462:Thioredoxin |
| A06722 | Contig3 | 2498446 | 2499210 | - | 663 |  | 4.31 | 3.63 | 2.89 | 0.71 | 0.00 | 0.40 | 3.30 | 0.000 | - && - && - |
| A06725 | Contig3 | 2504604 | 2506204 | - | 1038 |  | 19.95 | 22.92 | 18.22 | 12.43 | 7.30 | 10.31 | 1.02 | 0.000 | - && P9WMU2.1 RecName: Full=Probable glycerophosphoryl diester phosphodiesterase 1; Short=Glycerophosphodiester phosphodiesterase 1 >P9WMU3.1 RecName: Full=Probable glycerophosphoryl diester phosphodiesterase 1; Short=Glycerophosphodiester phosphodiesterase 1 && PF03009:Glycerophosphoryl diester phosphodiesterase family |
| A06733 | Contig3 | 2522626 | 2526394 | + | 2676 |  | 70.49 | 31.40 | 56.67 | 26.06 | 20.85 | 23.25 | 1.18 | 0.000 | - && P0CS15.1 RecName: Full=DNA damage-inducible protein 1 >P0CS14.1 RecName: Full=DNA damage-inducible protein 1 && PF09668:Aspartyl protease |
| A06736 | Contig3 | 2528731 | 2530364 | + | 1578 |  | 0.19 | 0.32 | 0.35 | 1.78 | 1.72 | 2.95 | -2.92 | 0.000 | - && P13860.1 RecName: Full=Exoglucanase 1; AltName: Full=1,4-beta-cellobiohydrolase; AltName: Full=Exocellobiohydrolase I; AltName: Full=Exoglucanase I; Flags: Precursor && PF00734:Fungal cellulose binding domain\|PF00840:Glycosyl hydrolase family 7 |
| A06738 | Contig3 | 2534089 | 2536742 | + | 2391 |  | 0.08 | 0.13 | 0.27 | 1.13 | 0.27 | 0.29 | -1.84 | 0.035 | - && - && - |
| A06757 | Contig3 | 323381 | 324623 | + | 1050 |  | 915.32 | 641.24 | 743.67 | 282.36 | 342.79 | 210.79 | 1.46 | 0.000 | - && O75845.2 RecName: Full=Lathosterol oxidase; AltName: Full=C-5 sterol desaturase; AltName: Full=Delta(7)-sterol 5-desaturase; AltName: Full=Delta(7)-sterol C5(6)-desaturase; AltName: Full=Lathosterol 5-desaturase; AltName: Full=Sterol-C5-desaturase && PF04116:Fatty acid hydroxylase superfamily |
| A06760 | Contig3 | 333433 | 338041 | - | 3228 |  | 76.00 | 71.50 | 61.24 | 26.76 | 33.18 | 37.87 | 1.09 | 0.000 | - && Q1K8G0.1 RecName: Full=Cystathionine beta-lyase && PF00288:GHMP kinases N terminal domain\|PF08544:GHMP kinases C terminal\|PF01053:Cys/Met metabolism PLP-dependent enzyme |
| A06761 | Contig3 | 341540 | 342652 | + | 987 |  | 0.60 | 0.71 | 0.74 | 0.00 | 0.09 | 0.00 | 4.44 | 0.004 | - && A8NU13.1 RecName: Full=Linoleate 10R-lipoxygenase COP4; AltName: Full=(+)-sativene synthase; AltName: Full=Beta-copaene synthase; AltName: Full=Beta-cubebene synthase; AltName: Full=Sesquiterpene synthase COP4 && PF03936:Terpene synthase family, metal binding domain |
| A06762 | Contig3 | 342947 | 344086 | - | 1020 |  | 7.64 | 5.61 | 5.82 | 1.56 | 1.47 | 0.52 | 2.43 | 0.000 | - && A8NU13.1 RecName: Full=Linoleate 10R-lipoxygenase COP4; AltName: Full=(+)-sativene synthase; AltName: Full=Beta-copaene synthase; AltName: Full=Beta-cubebene synthase; AltName: Full=Sesquiterpene synthase COP4 && PF03936:Terpene synthase family, metal binding domain |
| A06773 | Contig3 | 369254 | 369713 | - | 411 |  | 0.24 | 0.24 | 0.44 | 2.50 | 1.82 | 1.71 | -2.70 | 0.012 | - && - && - |
| A06775 | Contig40 | 4898 | 6301 | - | 1290 |  | 3.82 | 6.07 | 3.04 | 14.57 | 18.50 | 21.29 | -2.07 | 0.000 | - && - && PF13472:GDSL-like Lipase/Acylhydrolase family |
| A06783 | Contig40 | 47787 | 51046 | + | 2640 |  | 2.69 | 3.54 | 4.88 | 1.98 | 1.63 | 1.36 | 1.16 | 0.000 | - && - && PF05699:hAT family C-terminal dimerisation region |
| A06785 | Contig40 | 56225 | 57523 | + | 1239 |  | 0.80 | 0.73 | 0.66 | 0.00 | 0.00 | 0.14 | 3.95 | 0.003 | - && - && - |
| A06787 | Contig40 | 59806 | 63544 | - | 2760 |  | 27.90 | 29.57 | 25.02 | 62.40 | 72.07 | 78.06 | -1.37 | 0.000 | - && O13923.1 RecName: Full=Coronin-like protein crn1 && PF08953:Domain of unknown function (DUF1899)\|PF01400:Astacin (Peptidase family M12A)\|PF16300:Type of WD40 repeat |
| A06790 | Contig40 | 70868 | 76912 | + | 3711 |  | 1.25 | 1.43 | 0.81 | 0.35 | 0.55 | 0.73 | 1.09 | 0.011 | - && - && - |
| A06807 | Contig41 | 64599 | 67690 | + | 2211 |  | 17.30 | 24.29 | 22.85 | 61.78 | 87.43 | 52.16 | -1.64 | 0.000 | - && Q0CEF3.1 RecName: Full=Probable beta-glucosidase L; AltName: Full=Beta-D-glucoside glucohydrolase L; AltName: Full=Cellobiase L; AltName: Full=Gentiobiase L; Flags: Precursor && PF01915:Glycosyl hydrolase family 3 C-terminal domain\|PF00933:Glycosyl hydrolase family 3 N terminal domain\|PF14310:Fibronectin type III-like domain |
| A06836 | Contig42 | 97403 | 101598 | - | 2874 |  | 14.96 | 25.57 | 14.02 | 4.72 | 1.86 | 3.79 | 2.40 | 0.000 | - && Q01117.2 RecName: Full=Alpha-amylase 1; AltName: Full=1,4-alpha-D-glucan glucanohydrolase 1; Flags: Precursor && PF00106:short chain dehydrogenase\|PF00128:Alpha amylase, catalytic domain\|PF09260:Domain of unknown function (DUF1966) |
| A06837 | Contig42 | 102103 | 103947 | - | 1470 |  | 5.10 | 8.13 | 3.23 | 1.27 | 1.02 | 1.37 | 2.17 | 0.000 | - && Q01117.2 RecName: Full=Alpha-amylase 1; AltName: Full=1,4-alpha-D-glucan glucanohydrolase 1; Flags: Precursor && PF00128:Alpha amylase, catalytic domain\|PF09260:Domain of unknown function (DUF1966) |
| A06839 | Contig42 | 109129 | 110845 | - | 1029 |  | 33.25 | 28.10 | 27.52 | 8.27 | 5.55 | 2.98 | 2.40 | 0.000 | - && - && - |
| A06840 | Contig42 | 111358 | 113647 | + | 1743 |  | 7.69 | 9.22 | 6.71 | 1.66 | 1.13 | 2.32 | 2.21 | 0.000 | - && Q0R4L2.1 RecName: Full=Pyranose dehydrogenase 3; Short=PDH 3; AltName: Full=Pyranose:quinone oxidoreductase 3; Flags: Precursor && PF00732:GMC oxidoreductase\|PF05199:GMC oxidoreductase |
| A06854 | Contig43 | 51403 | 53151 | - | 1641 |  | 3.79 | 2.02 | 1.39 | 7.01 | 6.96 | 5.40 | -1.43 | 0.000 | - && B0XQS8.1 RecName: Full=Probable quinate permease; AltName: Full=Quinate transporter && PF00083:Sugar (and other) transporter |
| A06862 | Contig43 | 83800 | 84495 | - | 696 |  | 2.55 | 2.16 | 1.05 | 0.27 | 0.54 | 0.38 | 2.28 | 0.005 | - && - && PF00561:alpha/beta hydrolase fold |
| A06863 | Contig43 | 94230 | 95319 | + | 912 |  | 40.54 | 53.39 | 39.56 | 180.02 | 205.70 | 293.78 | -2.35 | 0.000 | - && - && - |
| A06864 | Contig43 | 102352 | 105668 | - | 1290 |  | 13.53 | 11.44 | 12.96 | 3.41 | 3.63 | 3.20 | 1.89 | 0.000 | - && - && - |
| A06871 | Contig44 | 25145 | 26079 | + | 669 |  | 0.00 | 0.00 | 0.14 | 1.68 | 0.98 | 0.13 | -4.35 | 0.012 | - && - && PF07883:Cupin domain |
| A06872 | Contig44 | 27184 | 29442 | + | 1596 |  | 30.03 | 19.31 | 36.06 | 9.55 | 9.27 | 8.41 | 1.65 | 0.000 | - && Q9C0V8.1 RecName: Full=Uncharacterized transporter PB10D8.01 && PF07690:Major Facilitator Superfamily |
| A06874 | Contig44 | 32959 | 33899 | - | 876 |  | 9.12 | 5.39 | 6.88 | 18.46 | 19.56 | 11.92 | -1.22 | 0.000 | - && - && PF12171:Zinc-finger double-stranded RNA-binding |
| A06878 | Contig44 | 50822 | 54103 | - | 2367 |  | 74.15 | 75.66 | 63.45 | 100.41 | 161.12 | 223.68 | -1.19 | 0.000 | - && O13966.2 RecName: Full=Aconitate hydratase, mitochondrial; Short=Aconitase; AltName: Full=Citrate hydro-lyase; Flags: Precursor && PF00330:Aconitase family (aconitate hydratase)\|PF00694:Aconitase C-terminal domain |
| A06927 | Contig47 | 7863 | 8440 | + | 405 |  | 307.01 | 304.63 | 295.03 | 46.40 | 32.13 | 47.24 | 2.85 | 0.000 | - && P86242.2 RecName: Full=Papain inhibitor; Short=SPI; Flags: Precursor && PF03330:Rare lipoprotein A (RlpA)-like double-psi beta-barrel |
| A06937 | Contig47 | 50400 | 52088 | + | 1299 |  | 23.15 | 28.36 | 23.21 | 8.57 | 8.86 | 7.30 | 1.60 | 0.000 | - && - && - |
| A06963 | Contig49 | 38577 | 40397 | - | 1359 |  | 1.02 | 1.48 | 1.48 | 0.62 | 0.07 | 0.06 | 2.40 | 0.003 | - && Q09170.2 RecName: Full=Serine/threonine-protein kinase cds1; AltName: Full=Checkpoint kinase cds1 && PF00498:FHA domain\|PF00069:Protein kinase domain |
| A06976 | Contig4 | 937743 | 939307 | - | 1155 |  | 80.93 | 58.76 | 98.55 | 202.78 | 261.78 | 201.27 | -1.48 | 0.000 | - && - && - |
| A06979 | Contig4 | 945038 | 946629 | - | 1086 |  | 62.65 | 57.96 | 57.37 | 21.44 | 23.01 | 33.45 | 1.19 | 0.000 | - && O14104.1 RecName: Full=Homoisocitrate dehydrogenase; Short=HICDH && PF00180:Isocitrate/isopropylmalate dehydrogenase |
| A06983 | Contig4 | 951773 | 953912 | - | 2013 |  | 180.50 | 140.53 | 152.61 | 339.33 | 311.62 | 449.17 | -1.22 | 0.000 | - && Q92403.1 RecName: Full=5-aminolevulinate synthase, mitochondrial; AltName: Full=5-aminolevulinic acid synthase; AltName: Full=Delta-ALA synthase; AltName: Full=Delta-aminolevulinate synthase; Flags: Precursor && PF00155:Aminotransferase class I and II |
| A06996 | Contig4 | 1001018 | 1002613 | - | 1143 |  | 30.45 | 21.17 | 17.10 | 8.02 | 9.99 | 8.68 | 1.36 | 0.000 | - && Q09839.1 RecName: Full=Uncharacterized protein C16C9.01c && PF00294:pfkB family carbohydrate kinase |
| A07003 | Contig4 | 1022244 | 1024002 | + | 1377 |  | 4.80 | 5.39 | 9.82 | 14.94 | 24.00 | 11.22 | -1.33 | 0.000 | - && P43485.2 RecName: Full=Mitomycin radical oxidase && PF01565:FAD binding domain |
| A07010 | Contig4 | 1047697 | 1049443 | - | 1290 |  | 7.11 | 3.89 | 4.67 | 0.80 | 1.16 | 1.90 | 2.02 | 0.000 | - && B8NHY4.1 RecName: Full=O-methylsterigmatocystin oxidoreductase; Short=OMST oxidoreductase; AltName: Full=Aflatoxin B synthase; AltName: Full=Aflatoxin biosynthesis protein Q; AltName: Full=Cytochrome P450 64 >P0CT93.1 RecName: Full=O-methylsterigmatocystin oxidoreductase; Short=OMST oxidoreductase; AltName: Full=Aflatoxin B synthase; AltName: Full=Aflatoxin biosynthesis protein Q; AltName: Full=Cytochrome P450 64 && PF00067:Cytochrome P450 |
| A07013 | Contig4 | 1054331 | 1059812 | - | 3348 |  | 7.13 | 9.03 | 7.56 | 20.05 | 28.30 | 26.76 | -1.66 | 0.000 | - && A8NWR2.2 RecName: Full=Ribosome biogenesis protein ERB1; AltName: Full=Eukaryotic ribosome biogenesis protein 1 && PF08145:BOP1NT (NUC169) domain |
| A07026 | Contig4 | 1103935 | 1106082 | - | 1533 |  | 1.93 | 2.95 | 2.20 | 0.55 | 0.61 | 0.29 | 2.29 | 0.000 | - && - && - |
| A07030 | Contig4 | 1121116 | 1123941 | + | 1680 |  | 20.42 | 40.45 | 16.42 | 8.57 | 6.07 | 11.91 | 1.54 | 0.000 | - && Q09752.2 RecName: Full=Multidrug resistance protein fnx1 && PF07690:Major Facilitator Superfamily |
| A07037 | Contig4 | 1141076 | 1142345 | + | 852 |  | 187.60 | 203.96 | 175.52 | 54.76 | 63.40 | 31.00 | 1.93 | 0.000 | - && Q0TVF9.2 RecName: Full=Dol-P-Man:Man(5)GlcNAc(2)-PP-Dol alpha-1,3-mannosyltransferase; AltName: Full=Asparagine-linked glycosylation protein 6; AltName: Full=Dol-P-Man-dependent alpha(1-3)-mannosyltransferase; AltName: Full=Dolichyl-P-Man:Man(5)GlcNAc(2)-PP-dolichyl mannosyltransferase && - |
| A07038 | Contig4 | 1142716 | 1143556 | - | 549 |  | 14.73 | 16.27 | 13.81 | 50.58 | 78.26 | 47.48 | -1.98 | 0.000 | - && - && - |
| A07048 | Contig4 | 1168581 | 1174249 | - | 4083 |  | 9.39 | 7.94 | 6.35 | 2.63 | 2.73 | 3.03 | 1.50 | 0.000 | - && O94491.1 RecName: Full=Uncharacterized transporter C417.10 && PF07690:Major Facilitator Superfamily |
| A07073 | Contig4 | 1233432 | 1236153 | - | 2274 |  | 102.25 | 71.83 | 126.50 | 33.71 | 54.09 | 47.12 | 1.16 | 0.000 | - && P55251.1 RecName: Full=3-isopropylmalate dehydratase; AltName: Full=Alpha-IPM isomerase; Short=IPMI; AltName: Full=Isopropylmalate isomerase && PF00330:Aconitase family (aconitate hydratase)\|PF00694:Aconitase C-terminal domain |
| A07074 | Contig4 | 1237454 | 1239836 | + | 1887 |  | 37.68 | 53.09 | 80.36 | 261.66 | 231.32 | 154.87 | -1.92 | 0.000 | - && O94547.1 RecName: Full=Serine/threonine-protein kinase srk1; AltName: Full=Sty1-regulated kinase 1 && PF00069:Protein kinase domain |
| A07087 | Contig4 | 1273861 | 1275504 | - | 1476 |  | 87.71 | 82.64 | 95.62 | 209.35 | 336.39 | 203.58 | -1.49 | 0.000 | - && P40086.1 RecName: Full=Cytochrome c oxidase assembly protein COX15 && PF02628:Cytochrome oxidase assembly protein |
| A07094 | Contig4 | 1287861 | 1289218 | - | 1134 |  | 320.68 | 369.77 | 267.93 | 784.74 | 647.43 | 726.06 | -1.17 | 0.000 | - && - && PF13668:Ferritin-like domain |
| A07130 | Contig4 | 1399425 | 1400576 | + | 918 |  | 95.81 | 102.68 | 106.57 | 191.07 | 289.70 | 185.94 | -1.13 | 0.000 | - && A8P7Y3.1 RecName: Full=S-methyl-5'-thioadenosine phosphorylase; AltName: Full=5'-methylthioadenosine phosphorylase; Short=MTA phosphorylase; Short=MTAP; Short=MTAPase && PF01048:Phosphorylase superfamily |
| A07141 | Contig4 | 1473842 | 1478038 | - | 2778 |  | 2493.61 | 1711.59 | 2631.78 | 147.01 | 207.74 | 136.57 | 3.80 | 0.000 | - && O74969.1 RecName: Full=High-affinity glucose transporter ght2; AltName: Full=Hexose transporter 2 && PF08059:SEP domain\|PF00789:UBX domain\|PF00083:Sugar (and other) transporter |
| A07143 | Contig4 | 1484998 | 1487350 | - | 1971 |  | 0.95 | 1.63 | 1.53 | 7.68 | 2.61 | 1.02 | -1.46 | 0.011 | - && - && PF13632:Glycosyl transferase family group 2 |
| A07144 | Contig4 | 1488671 | 1494935 | - | 3423 |  | 11.09 | 7.54 | 26.53 | 3.71 | 4.38 | 1.82 | 2.19 | 0.001 | - && - && PF13632:Glycosyl transferase family group 2 |
| A07145 | Contig4 | 1496670 | 1498641 | + | 1728 |  | 711.68 | 411.89 | 891.14 | 359.21 | 363.77 | 208.99 | 1.11 | 0.000 | - && Q8N0N3.1 RecName: Full=Beta-1,3-glucan-binding protein; Short=GBP; Flags: Precursor && PF00722:Glycosyl hydrolases family 16 |
| A07147 | Contig4 | 1503334 | 1506764 | + | 2637 |  | 423.88 | 201.35 | 478.02 | 104.13 | 106.53 | 58.27 | 2.04 | 0.000 | - && - && PF13632:Glycosyl transferase family group 2 |
| A07154 | Contig4 | 1529523 | 1535403 | + | 3147 |  | 479.45 | 276.38 | 337.91 | 40.08 | 54.67 | 27.33 | 3.16 | 0.000 | - && D4AUF1.1 RecName: Full=WSC domain-containing protein ARB_07867; Flags: Precursor && PF01822:WSC domain\|PF07250:Glyoxal oxidase N-terminus\|PF09118:Domain of unknown function (DUF1929) |
| A07155 | Contig4 | 1535760 | 1537794 | + | 1596 |  | 199.24 | 183.16 | 240.97 | 96.25 | 98.01 | 81.00 | 1.18 | 0.000 | - && - && PF00498:FHA domain |
| A07185 | Contig4 | 1636237 | 1639173 | + | 2067 |  | 1.19 | 1.55 | 1.50 | 1000.68 | 1056.93 | 677.23 | -9.33 | 0.000 | - && P04842.2 RecName: Full=Alcohol oxidase 1; Short=AO 1; Short=AOX 1; AltName: Full=Methanol oxidase 1; Short=MOX 1 >F2QY27.1 RecName: Full=Alcohol oxidase 1; Short=AO 1; Short=AOX 1; AltName: Full=Methanol oxidase 1; Short=MOX 1 && PF00732:GMC oxidoreductase\|PF05199:GMC oxidoreductase |
| A07208 | Contig4 | 1734920 | 1735819 | + | 732 |  | 11.18 | 16.87 | 13.98 | 39.34 | 34.14 | 22.06 | -1.18 | 0.000 | - && - && PF03188:Eukaryotic cytochrome b561 |
| A07213 | Contig4 | 1746682 | 1747544 | + | 639 |  | 563.84 | 506.81 | 511.36 | 122.76 | 122.62 | 152.03 | 1.99 | 0.000 | - && Q9V3P0.1 RecName: Full=Peroxiredoxin 1; AltName: Full=Cytosolic thioredoxin peroxidase; Short=DPx-4783; Short=DmTPx-1; AltName: Full=Thioredoxin peroxidase && PF00578:AhpC/TSA family\|PF10417:C-terminal domain of 1-Cys peroxiredoxin |
| A07223 | Contig4 | 1788616 | 1789538 | + | 627 |  | 128.01 | 65.32 | 208.92 | 386.06 | 345.18 | 309.18 | -1.37 | 0.000 | - && D4AK18.2 RecName: Full=Uncharacterized secreted protein ARB_06907; Flags: Precursor && PF07510:Protein of unknown function (DUF1524) |
| A07224 | Contig4 | 1790340 | 1792628 | - | 1782 |  | 1512.63 | 1262.18 | 1158.22 | 332.74 | 281.25 | 503.75 | 1.82 | 0.000 | - && P30608.1 RecName: Full=Cytochrome P450 52A6; AltName: Full=Alkane-inducible P450-ALK3; AltName: Full=CYPLIIA6 && PF00067:Cytochrome P450 |
| A07225 | Contig4 | 1794504 | 1797396 | + | 2304 |  | 66.25 | 66.84 | 53.88 | 32.34 | 26.49 | 29.37 | 1.08 | 0.000 | - && Q10094.1 RecName: Full=Uncharacterized protein C11D3.15 && PF01968:Hydantoinase/oxoprolinase\|PF05378:Hydantoinase/oxoprolinase N-terminal region |
| A07228 | Contig4 | 1809495 | 1810966 | + | 1008 |  | 10.76 | 8.17 | 4.26 | 0.83 | 0.56 | 0.52 | 3.60 | 0.000 | - && - && - |
| A07231 | Contig4 | 1813590 | 1815185 | - | 1437 |  | 5.49 | 5.87 | 5.34 | 17.24 | 24.10 | 10.75 | -1.64 | 0.000 | - && A1CRV0.2 RecName: Full=Probable glucan 1,3-beta-glucosidase A; AltName: Full=Exo-1,3-beta-glucanase 1; AltName: Full=Exo-1,3-beta-glucanase A; Flags: Precursor && PF00150:Cellulase (glycosyl hydrolase family 5) |
| A07235 | Contig4 | 1822621 | 1823267 | - | 510 |  | 1.55 | 3.54 | 1.25 | 0.18 | 0.73 | 0.52 | 2.15 | 0.018 | - && - && - |
| A07244 | Contig4 | 1852077 | 1852981 | - | 543 |  | 36.14 | 42.71 | 48.79 | 123.11 | 164.81 | 88.08 | -1.56 | 0.000 | - && O14313.2 RecName: Full=Putative peroxiredoxin pmp20; AltName: Full=Peroxisomal membrane protein pmp20; AltName: Full=Thioredoxin reductase && PF08534:Redoxin |
| A07247 | Contig4 | 1863642 | 1864454 | - | 612 |  | 31.10 | 31.33 | 28.96 | 10.08 | 9.79 | 4.30 | 1.92 | 0.000 | - && - && - |
| A07249 | Contig4 | 1868766 | 1870293 | - | 1056 |  | 81.89 | 88.03 | 111.07 | 140.51 | 172.68 | 288.62 | -1.10 | 0.000 | - && Q9DC28.2 RecName: Full=Casein kinase I isoform delta; Short=CKI-delta; Short=CKId; AltName: Full=Tau-protein kinase CSNK1D >Q06486.2 RecName: Full=Casein kinase I isoform delta; Short=CKI-delta; AltName: Full=Tau-protein kinase CSNK1D && PF00069:Protein kinase domain |
| A07250 | Contig4 | 1870822 | 1873217 | + | 2163 |  | 63.18 | 63.82 | 72.18 | 170.31 | 235.61 | 341.82 | -1.91 | 0.000 | - && Q6NVE9.1 RecName: Full=Protein phosphatase PTC7 homolog; AltName: Full=T-cell activation protein phosphatase 2C; Short=TA-PP2C && - |
| A07251 | Contig4 | 1875626 | 1876437 | + | 642 |  | 9.52 | 8.76 | 14.80 | 3.35 | 2.04 | 0.55 | 2.48 | 0.000 | - && - && - |
| A07254 | Contig4 | 1886802 | 1888878 | - | 1392 |  | 4.53 | 6.56 | 4.72 | 0.67 | 0.61 | 0.69 | 3.01 | 0.000 | - && Q9C450.1 RecName: Full=Cytochrome P450 monooxygenase paxQ; AltName: Full=Paxilline synthesis protein Q && PF00067:Cytochrome P450 |
| A07271 | Contig4 | 1946179 | 1946771 | + | 423 |  | 995.82 | 1649.88 | 1145.00 | 3140.40 | 3179.43 | 2400.19 | -1.20 | 0.000 | - && - && PF14200:Ricin-type beta-trefoil lectin domain-like |
| A07272 | Contig4 | 1946986 | 1947754 | + | 705 |  | 58.18 | 64.93 | 30.58 | 14.72 | 10.76 | 18.30 | 1.81 | 0.000 | - && P40586.1 RecName: Full=Uncharacterized protein YIR042C && PF13302:Acetyltransferase (GNAT) domain |
| A07276 | Contig4 | 1955313 | 1956304 | + | 810 |  | 17.53 | 12.52 | 29.66 | 59.10 | 70.03 | 39.55 | -1.50 | 0.000 | - && - && - |
| A07278 | Contig4 | 1958436 | 1960983 | - | 1734 |  | 29.06 | 20.44 | 45.46 | 94.52 | 125.08 | 46.97 | -1.49 | 0.000 | - && Q9SR37.1 RecName: Full=Beta-glucosidase 23; Short=AtBGLU23; AltName: Full=Protein PHOSPHATE STARVATION-RESPONSE 3.1; Flags: Precursor && PF00232:Glycosyl hydrolase family 1 |
| A07284 | Contig4 | 1980250 | 1989559 | - | 4374 |  | 27.23 | 35.87 | 14.89 | 8.42 | 7.94 | 6.30 | 1.78 | 0.000 | - && Q9ZW95.1 RecName: Full=Cytokinin hydroxylase; AltName: Full=Cytochrome P450 35A2 && PF00067:Cytochrome P450 |
| A07290 | Contig4 | 2012013 | 2014520 | + | 2199 |  | 4.93 | 8.67 | 9.18 | 126.45 | 187.01 | 123.20 | -4.26 | 0.000 | - && Q4WFI6.1 RecName: Full=Probable exo-1,4-beta-xylosidase bxlB; AltName: Full=1,4-beta-D-xylan xylohydrolase bxlB; AltName: Full=Beta-xylosidase bxlB; AltName: Full=Xylobiase bxlB; Flags: Precursor >B0Y0I4.1 RecName: Full=Probable exo-1,4-beta-xylosidase bxlB; AltName: Full=1,4-beta-D-xylan xylohydrolase bxlB; AltName: Full=Beta-xylosidase bxlB; AltName: Full=Xylobiase bxlB; Flags: Precursor && PF01915:Glycosyl hydrolase family 3 C-terminal domain\|PF00933:Glycosyl hydrolase family 3 N terminal domain\|PF14310:Fibronectin type III-like domain |
| A07291 | Contig4 | 2015151 | 2018246 | + | 2994 |  | 24.77 | 25.01 | 36.12 | 149.42 | 114.84 | 104.70 | -2.10 | 0.000 | - && - && - |
| A07293 | Contig4 | 2020831 | 2021699 | - | 696 |  | 54.83 | 60.58 | 56.70 | 213.19 | 107.73 | 185.23 | -1.56 | 0.000 | - && - && - |
| A07305 | Contig4 | 2056835 | 2058408 | - | 1395 |  | 13.29 | 9.57 | 13.29 | 4.62 | 4.23 | 4.78 | 1.41 | 0.000 | - && O34703.1 RecName: Full=Uncharacterized ATPase YjoB && PF00004:ATPase family associated with various cellular activities (AAA) |
| A07311 | Contig4 | 2082354 | 2085407 | - | 2712 |  | 15.82 | 15.99 | 9.80 | 6.62 | 4.94 | 6.28 | 1.22 | 0.000 | - && - && - |
| A07315 | Contig4 | 2097843 | 2101278 | - | 3081 |  | 82.60 | 64.22 | 85.54 | 29.68 | 28.71 | 30.31 | 1.39 | 0.000 | - && Q6DCX2.1 RecName: Full=Protein argonaute-2; Short=Argonaute2; AltName: Full=Argonaute RISC catalytic component 2; AltName: Full=Eukaryotic translation initiation factor 2C 2; Short=eIF-2C 2; Short=eIF2C 2; AltName: Full=Protein slicer && PF08699:Argonaute linker 1 domain\|PF16486:N-terminal domain of argonaute\|PF16488:Argonaute linker 2 domain\|PF02170:PAZ domain\|PF02171:Piwi domain |
| A07316 | Contig4 | 2101798 | 2105360 | - | 1887 |  | 1307.91 | 1311.48 | 1014.00 | 330.93 | 227.35 | 563.15 | 1.70 | 0.000 | - && P47154.1 RecName: Full=CAAX prenyl protease 1; AltName: Full=A-factor-converting enzyme; AltName: Full=Prenyl protein-specific endoprotease 1; Short=PPSEP 1 && PF01435:Peptidase family M48\|PF16491:CAAX prenyl protease N-terminal, five membrane helices |
| A07322 | Contig4 | 2121859 | 2130108 | + | 5169 |  | 31.51 | 32.67 | 22.07 | 13.26 | 9.76 | 13.19 | 1.25 | 0.000 | - && B0BK71.1 RecName: Full=Dye-decolorizing peroxidase msp1; AltName: Full=Peroxidase 1; Short=MsP1; Flags: Precursor && PF00067:Cytochrome P450\|PF00026:Eukaryotic aspartyl protease |
| A07323 | Contig4 | 2130552 | 2133186 | - | 2367 |  | 112.06 | 115.23 | 99.11 | 240.99 | 240.14 | 490.37 | -1.57 | 0.000 | - && - && PF05199:GMC oxidoreductase |
| A07324 | Contig4 | 2134459 | 2138879 | + | 3315 |  | 52.86 | 38.73 | 30.97 | 77.76 | 91.07 | 204.32 | -1.61 | 0.000 | - && B0BK72.1 RecName: Full=Peroxidase 2; Short=MsP2; Flags: Precursor && PF13519:von Willebrand factor type A domain |
| A07325 | Contig4 | 2139367 | 2144463 | + | 2256 |  | 26.49 | 23.67 | 29.76 | 69.46 | 56.47 | 55.51 | -1.18 | 0.000 | - && A2QQV6.1 RecName: Full=Probable quinate permease; AltName: Full=Quinate transporter && PF00083:Sugar (and other) transporter\|PF13869:Nucleotide hydrolase |
| A07335 | Contig4 | 2184973 | 2185698 | + | 726 |  | 2.85 | 3.87 | 2.14 | 0.90 | 0.77 | 1.93 | 1.30 | 0.027 | - && - && - |
| A07345 | Contig4 | 2216314 | 2216938 | - | 525 |  | 27.98 | 41.69 | 25.23 | 8.01 | 13.37 | 7.69 | 1.71 | 0.000 | - && - && - |
| A07354 | Contig4 | 2264435 | 2268161 | - | 1701 |  | 31.07 | 23.08 | 35.07 | 5.94 | 10.57 | 5.88 | 1.99 | 0.000 | - && G3Y416.1 RecName: Full=Cytochrome P450 monooxygenase yanC; AltName: Full=Yanuthone D synthesis protein C; Flags: Precursor && PF00067:Cytochrome P450 |
| A07357 | Contig4 | 2272191 | 2274330 | + | 1638 |  | 15.65 | 11.22 | 16.45 | 5.88 | 7.14 | 6.16 | 1.18 | 0.000 | - && Q9P6J5.1 RecName: Full=Uncharacterized permease C1683.05 && PF02133:Permease for cytosine/purines, uracil, thiamine, allantoin |
| A07358 | Contig4 | 2275965 | 2277775 | + | 936 |  | 14.22 | 14.16 | 9.95 | 4.49 | 3.30 | 3.56 | 1.75 | 0.000 | - && - && - |
| A07361 | Contig4 | 2285859 | 2292364 | + | 2970 |  | 36.22 | 86.23 | 17.01 | 10.58 | 12.89 | 23.34 | 1.57 | 0.030 | - && O74628.1 RecName: Full=Uncharacterized oxidoreductase C162.03 && PF02798:Glutathione S-transferase, N-terminal domain\|PF00106:short chain dehydrogenase |
| A07367 | Contig4 | 2310581 | 2317146 | + | 1878 |  | 45.99 | 60.51 | 25.44 | 10.55 | 8.08 | 11.17 | 2.15 | 0.000 | - && Q9LMF0.1 RecName: Full=UDP-glycosyltransferase 85A5 && PF00201:UDP-glucoronosyl and UDP-glucosyl transferase |
| A07368 | Contig4 | 2317894 | 2319441 | - | 789 |  | 10.12 | 7.89 | 8.68 | 2.73 | 1.66 | 2.56 | 1.94 | 0.000 | - && - && - |
| A07369 | Contig4 | 2321240 | 2323642 | + | 1653 |  | 37.34 | 31.82 | 36.86 | 13.69 | 18.07 | 13.86 | 1.22 | 0.000 | - && Q94AX4.1 RecName: Full=D-lactate dehydrogenase [cytochrome], mitochondrial; Short=AtD-LDH; AltName: Full=D-lactate ferricytochrome C oxidoreductase; AltName: Full=Glycolate deshydrogenase; Flags: Precursor && PF02913:FAD linked oxidases, C-terminal domain\|PF01565:FAD binding domain |
| A07382 | Contig4 | 2358092 | 2359686 | + | 1356 |  | 14.69 | 13.18 | 12.73 | 4.48 | 7.66 | 6.67 | 1.11 | 0.000 | - && - && PF01266:FAD dependent oxidoreductase |
| A07399 | Contig4 | 2407644 | 2410925 | + | 3171 |  | 15.77 | 15.48 | 16.13 | 5.22 | 8.06 | 5.78 | 1.31 | 0.000 | - && O59751.1 RecName: Full=Kinesin-like protein 6 && PF00225:Kinesin motor domain |
| A07405 | Contig4 | 2423426 | 2423983 | + | 558 |  | 3890.75 | 2434.68 | 4269.37 | 1230.02 | 1640.87 | 694.99 | 1.57 | 0.000 | - && - && - |
| A07423 | Contig4 | 2492077 | 2492772 | + | 696 |  | 13.60 | 16.44 | 14.96 | 37.34 | 44.79 | 28.62 | -1.30 | 0.000 | - && Q10058.1 RecName: Full=Putative oxidoreductase C1F5.03c && PF01266:FAD dependent oxidoreductase |
| A07426 | Contig4 | 2506898 | 2510206 | + | 2205 |  | 8.59 | 7.51 | 9.40 | 3.90 | 5.31 | 3.42 | 1.01 | 0.000 | - && Q12083.1 RecName: Full=DNA mismatch repair protein MLH3; AltName: Full=MutL protein homolog 3 && PF08676:MutL C terminal dimerisation domain\|PF13589:Histidine kinase-, DNA gyrase B-, and HSP90-like ATPase |
| A07430 | Contig4 | 2518607 | 2519982 | + | 1152 |  | 257.55 | 254.98 | 214.58 | 58.84 | 73.54 | 45.40 | 2.03 | 0.000 | - && O59930.1 RecName: Full=3-isopropylmalate dehydrogenase; Short=3-IPM-DH; Short=IMDH; AltName: Full=Beta-IPM dehydrogenase && PF00180:Isocitrate/isopropylmalate dehydrogenase |
| A07433 | Contig4 | 2524586 | 2526043 | - | 1458 |  | 1425.42 | 1380.17 | 1116.31 | 571.56 | 617.33 | 611.90 | 1.12 | 0.000 | - && P50139.2 RecName: Full=GTP cyclohydrolase-2; AltName: Full=GTP cyclohydrolase II && PF00925:GTP cyclohydrolase II |
| A07438 | Contig4 | 2544641 | 2546661 | + | 1455 |  | 3.80 | 6.49 | 2.13 | 1.16 | 1.80 | 0.60 | 1.80 | 0.000 | - && - && - |
| A07451 | Contig4 | 19838 | 25853 | + | 2964 |  | 36.66 | 52.36 | 34.18 | 121.66 | 141.11 | 95.99 | -1.54 | 0.000 | - && P80576.2 RecName: Full=Phospho-2-dehydro-3-deoxyheptonate aldolase; AltName: Full=3-deoxy-D-arabino-heptulosonate 7-phosphate synthase; AltName: Full=DAHP synthase; AltName: Full=Phospho-2-keto-3-deoxyheptonate aldolase && PF00083:Sugar (and other) transporter\|PF01474:Class-II DAHP synthetase family |
| A07453 | Contig4 | 29731 | 31151 | + | 1011 |  | 3.22 | 2.58 | 1.63 | 1.29 | 0.74 | 1.48 | 1.08 | 0.039 | - && Q5NUF4.1 RecName: Full=2-hydroxyisoflavanone dehydratase; AltName: Full=Carboxylesterase HIDM && PF07859:alpha/beta hydrolase fold |
| A07460 | Contig4 | 62033 | 63780 | + | 1698 |  | 3.72 | 4.08 | 4.95 | 9.09 | 7.72 | 9.66 | -1.05 | 0.000 | - && - && - |
| A07462 | Contig4 | 69901 | 71920 | - | 1446 |  | 44.32 | 50.06 | 58.69 | 108.17 | 139.96 | 85.88 | -1.13 | 0.000 | - && Q9C450.1 RecName: Full=Cytochrome P450 monooxygenase paxQ; AltName: Full=Paxilline synthesis protein Q && PF00067:Cytochrome P450 |
| A07467 | Contig4 | 85808 | 87831 | + | 1497 |  | 43.21 | 54.79 | 53.88 | 113.54 | 114.93 | 96.20 | -1.10 | 0.000 | - && Q701P2.1 RecName: Full=Ent-kaurene oxidase; AltName: Full=Cytochrome P450 503A1; AltName: Full=Cytochrome P450-4 [Fusarium proliferatum] && PF00067:Cytochrome P450 |
| A07474 | Contig4 | 134841 | 135931 | - | 936 |  | 15.17 | 11.91 | 18.06 | 40.36 | 41.80 | 26.63 | -1.27 | 0.000 | - && - && PF07859:alpha/beta hydrolase fold |
| A07481 | Contig4 | 171981 | 175142 | - | 3162 |  | 0.16 | 0.29 | 0.46 | 0.74 | 1.33 | 0.47 | -1.49 | 0.014 | - && P10394.1 RecName: Full=Retrovirus-related Pol polyprotein from transposon 412; Includes: RecName: Full=Protease; Includes: RecName: Full=Reverse transcriptase; Includes: RecName: Full=Endonuclease && PF00665:Integrase core domain |
| A07483 | Contig4 | 176021 | 178324 | - | 2304 |  | 0.09 | 0.13 | 0.08 | 0.61 | 0.49 | 0.27 | -2.20 | 0.014 | - && - && - |
| A07484 | Contig4 | 179957 | 180948 | + | 804 |  | 16.31 | 20.85 | 22.84 | 20.23 | 60.08 | 82.96 | -1.44 | 0.000 | - && - && - |
| A07485 | Contig4 | 181508 | 182493 | - | 618 |  | 2177.72 | 2271.89 | 2230.83 | 969.45 | 1126.66 | 827.32 | 1.19 | 0.000 | - && Q92429.1 RecName: Full=Superoxide dismutase [Mn], mitochondrial; AltName: Full=Mn-SOD; Flags: Precursor && PF02777:Iron/manganese superoxide dismutases, C-terminal domain\|PF00081:Iron/manganese superoxide dismutases, alpha-hairpin domain |
| A07497 | Contig4 | 237337 | 238599 | - | 1035 |  | 28.39 | 35.50 | 33.01 | 12.29 | 12.48 | 13.40 | 1.34 | 0.000 | - && - && - |
| A07499 | Contig4 | 241332 | 242137 | - | 633 |  | 7.94 | 11.10 | 10.25 | 63.51 | 64.33 | 43.12 | -2.55 | 0.000 | - && - && - |
| A07503 | Contig4 | 259510 | 260160 | + | 372 |  | 6.63 | 6.21 | 9.82 | 1.76 | 6.04 | 3.07 | 1.06 | 0.045 | - && - && - |
| A07514 | Contig4 | 301571 | 302984 | - | 1305 |  | 14.51 | 27.15 | 12.53 | 105.75 | 36.80 | 149.96 | -2.43 | 0.000 | - && - && - |
| A07525 | Contig4 | 340143 | 341957 | - | 1500 |  | 306.33 | 280.75 | 309.92 | 136.57 | 86.37 | 147.96 | 1.27 | 0.000 | - && - && - |
| A07528 | Contig4 | 351243 | 352915 | - | 1380 |  | 9.57 | 13.46 | 9.93 | 29.13 | 22.39 | 20.60 | -1.13 | 0.000 | - && - && - |
| A07532 | Contig4 | 370706 | 371891 | - | 924 |  | 0.53 | 0.33 | 0.89 | 2.02 | 1.42 | 1.42 | -1.48 | 0.032 | - && - && - |
| A07533 | Contig4 | 374319 | 375708 | + | 999 |  | 30.50 | 94.46 | 11.34 | 6.27 | 6.65 | 8.08 | 2.70 | 0.001 | - && P42327.1 RecName: Full=Alcohol dehydrogenase; Short=ADH && PF00107:Zinc-binding dehydrogenase\|PF08240:Alcohol dehydrogenase GroES-like domain |
| A07536 | Contig4 | 381396 | 382079 | + | 573 |  | 20.99 | 26.10 | 20.88 | 40.30 | 105.54 | 26.80 | -1.34 | 0.001 | - && P52748.1 RecName: Full=Hydrophobin-1; Flags: Precursor [Pisolithus tinctorius] && PF01185:Fungal hydrophobin |
| A07547 | Contig4 | 418084 | 428466 | - | 5970 |  | 19.42 | 17.10 | 20.01 | 7.31 | 7.23 | 5.72 | 1.48 | 0.000 | - && Q9P7V2.1 RecName: Full=ATP-binding cassette transporter abc4; Short=ABC transporter abc4; AltName: Full=ATP-energized glutathione S-conjugate pump abc4; AltName: Full=Glutathione S-conjugate-transporting ATPase abc4 && PF00664:ABC transporter transmembrane region\|PF00005:ABC transporter\|PF00067:Cytochrome P450 |
| A07557 | Contig4 | 462853 | 464091 | - | 996 |  | 933.86 | 652.62 | 879.65 | 256.74 | 488.63 | 331.65 | 1.20 | 0.000 | - && Q9UUH4.1 RecName: Full=Methylsterol monooxygenase; AltName: Full=C-4 methylsterol oxidase && PF04116:Fatty acid hydroxylase superfamily |
| A07571 | Contig4 | 526626 | 533593 | + | 4464 |  | 71.30 | 84.58 | 62.78 | 10.03 | 11.41 | 10.64 | 2.77 | 0.000 | - && P50125.2 RecName: Full=Homocysteine synthase; AltName: Full=O-acetylhomoserine sulfhydrylase; Short=OAH SHL; Short=OAH sulfhydrylase && PF01053:Cys/Met metabolism PLP-dependent enzyme |
| A07572 | Contig4 | 534094 | 538882 | + | 3675 |  | 13.07 | 15.90 | 17.65 | 143.64 | 231.57 | 92.10 | -3.33 | 0.000 | - && Q6UEF1.1 RecName: Full=Oxidoreductase AflY; AltName: Full=Aflatoxin biosynthesis protein Y && PF03221:Tc5 transposase DNA-binding domain\|PF14027:Protein of unknown function (DUF4243)\|PF03184:DDE superfamily endonuclease |
| A07585 | Contig4 | 571235 | 572969 | - | 1359 |  | 40.92 | 43.80 | 40.33 | 145.64 | 125.23 | 168.28 | -1.81 | 0.000 | - && - && PF07470:Glycosyl Hydrolase Family 88 |
| A07600 | Contig4 | 626907 | 628391 | - | 1266 |  | 418.71 | 302.67 | 344.69 | 73.85 | 103.89 | 56.63 | 2.19 | 0.000 | - && Q9C167.2 RecName: Full=Ribonucleoside-diphosphate reductase small chain; AltName: Full=Ribonucleotide reductase small subunit && PF00268:Ribonucleotide reductase, small chain |
| A07601 | Contig4 | 629392 | 630740 | + | 1155 |  | 166.64 | 95.35 | 163.48 | 43.96 | 46.36 | 50.00 | 1.60 | 0.000 | - && Q9C167.2 RecName: Full=Ribonucleoside-diphosphate reductase small chain; AltName: Full=Ribonucleotide reductase small subunit && PF00268:Ribonucleotide reductase, small chain |
| A07605 | Contig4 | 637131 | 638850 | + | 1143 |  | 3.71 | 3.60 | 5.59 | 1.96 | 2.54 | 1.23 | 1.17 | 0.004 | - && Q701P2.1 RecName: Full=Ent-kaurene oxidase; AltName: Full=Cytochrome P450 503A1; AltName: Full=Cytochrome P450-4 [Fusarium proliferatum] && PF00067:Cytochrome P450 |
| A07627 | Contig4 | 706122 | 710784 | - | 3561 |  | 0.94 | 0.87 | 0.85 | 3.33 | 0.81 | 3.97 | -1.61 | 0.001 | - && Q4LAL9.1 RecName: Full=Cathepsin D; Flags: Precursor && PF00026:Eukaryotic aspartyl protease |
| A07641 | Contig4 | 758533 | 760723 | - | 1947 |  | 618.97 | 562.27 | 541.11 | 118.66 | 112.79 | 177.18 | 2.08 | 0.000 | - && O59714.1 RecName: Full=Uncharacterized membrane protein C3B8.06 && PF10348:Domain of unknown function (DUF2427)\|PF10355:Protein of unknown function (Ytp1) |
| A07646 | Contig4 | 774396 | 776264 | + | 1443 |  | 1.57 | 0.49 | 0.76 | 5.05 | 5.51 | 2.74 | -2.24 | 0.000 | - && - && PF12846:AAA-like domain |
| A07649 | Contig4 | 782653 | 784236 | - | 1170 |  | 234.20 | 328.79 | 200.89 | 63.05 | 114.25 | 107.79 | 1.42 | 0.000 | - && O13991.3 RecName: Full=Uncharacterized oxidoreductase C26H5.09c && PF02894:Oxidoreductase family, C-terminal alpha/beta domain\|PF01408:Oxidoreductase family, NAD-binding Rossmann fold |
| A07651 | Contig4 | 787440 | 790704 | - | 3153 |  | 27.68 | 41.96 | 24.42 | 9.49 | 8.43 | 15.98 | 1.47 | 0.000 | - && P39692.2 RecName: Full=Sulfite reductase [NADPH] flavoprotein component && PF00667:FAD binding domain\|PF00175:Oxidoreductase NAD-binding domain |
| A07659 | Contig4 | 812204 | 813614 | - | 1179 |  | 3.51 | 2.13 | 3.18 | 1.27 | 1.19 | 0.60 | 1.53 | 0.002 | - && - && - |
| A07660 | Contig4 | 814310 | 814797 | - | 384 |  | 24.39 | 16.73 | 17.13 | 1.70 | 4.14 | 4.11 | 2.55 | 0.000 | - && - && - |
| A07661 | Contig4 | 816037 | 817145 | + | 891 |  | 33.09 | 24.34 | 16.71 | 9.97 | 7.77 | 12.21 | 1.31 | 0.000 | - && Q0IIS3.1 RecName: Full=Epoxide hydrolase 3; AltName: Full=Abhydrolase domain-containing protein 9 && PF00561:alpha/beta hydrolase fold |
| A07667 | Contig4 | 824828 | 826614 | - | 1263 |  | 193.61 | 194.02 | 221.47 | 938.66 | 1292.39 | 1461.95 | -2.60 | 0.000 | - && Q06099.1 RecName: Full=S-(hydroxymethyl)glutathione dehydrogenase; AltName: Full=Glutathione-dependent formaldehyde dehydrogenase; Short=FALDH; Short=FDH; Short=FLD; Short=GSH-FDH && PF08240:Alcohol dehydrogenase GroES-like domain\|PF00107:Zinc-binding dehydrogenase |
| A07682 | Contig4 | 878486 | 881017 | - | 1644 |  | 13.97 | 18.01 | 11.61 | 57.50 | 54.09 | 66.51 | -2.03 | 0.000 | - && Q07904.1 RecName: Full=Thiamine pathway transporter THI73 && PF07690:Major Facilitator Superfamily |
| A07688 | Contig4 | 893470 | 897309 | - | 1215 |  | 331.84 | 493.27 | 249.69 | 108.04 | 128.67 | 191.05 | 1.33 | 0.000 | - && Q8NJR5.2 RecName: Full=Protein URE2 && PF00043:Glutathione S-transferase, C-terminal domain\|PF02798:Glutathione S-transferase, N-terminal domain |
| A07692 | Contig4 | 907001 | 908405 | + | 951 |  | 154.80 | 157.39 | 161.76 | 87.01 | 77.57 | 61.92 | 1.07 | 0.000 | - && Q9X248.1 RecName: Full=3-oxoacyl-[acyl-carrier-protein] reductase FabG; AltName: Full=3-ketoacyl-acyl carrier protein reductase; AltName: Full=Beta-Ketoacyl-acyl carrier protein reductase; AltName: Full=Beta-ketoacyl-ACP reductase && PF00106:short chain dehydrogenase |
| A07713 | Contig51 | 31589 | 36112 | - | 3450 |  | 1.29 | 0.32 | 0.71 | 2.28 | 1.60 | 0.92 | -1.05 | 0.038 | - && Q4P0P3.2 RecName: Full=Mediator of RNA polymerase II transcription subunit 14; AltName: Full=Mediator complex subunit 14 && PF08638:Mediator complex subunit MED14 |
| A07715 | Contig51 | 42244 | 46534 | - | 1956 |  | 7.96 | 18.53 | 8.31 | 34.27 | 75.76 | 48.72 | -2.19 | 0.000 | - && O43112.2 RecName: Full=Phosphoenolpyruvate carboxykinase [ATP] && PF01293:Phosphoenolpyruvate carboxykinase |
| A07719 | Contig52 | 7686 | 10511 | - | 1659 |  | 48.50 | 28.74 | 29.35 | 14.15 | 21.67 | 14.23 | 1.09 | 0.000 | - && Q09752.2 RecName: Full=Multidrug resistance protein fnx1 && PF07690:Major Facilitator Superfamily |
| A07723 | Contig52 | 25607 | 30848 | + | 2325 |  | 0.85 | 3.32 | 4.09 | 1.25 | 1.89 | 0.75 | 1.08 | 0.037 | - && - && PF00644:Poly(ADP-ribose) polymerase catalytic domain |
| A07734 | Contig54 | 5792 | 10154 | - | 1023 |  | 5.11 | 4.71 | 5.00 | 1.65 | 2.47 | 2.57 | 1.15 | 0.002 | - && P21575.2 RecName: Full=Dynamin-1; AltName: Full=B-dynamin; AltName: Full=D100; AltName: Full=Dynamin, brain && - |
| A07741 | Contig5 | 361393 | 364415 | - | 2211 |  | 6.15 | 6.58 | 5.62 | 42.33 | 60.37 | 29.77 | -2.85 | 0.000 | - && A2QTU5.1 RecName: Full=Alpha-xylosidase A; Flags: Precursor && PF01055:Glycosyl hydrolases family 31 |
| A07743 | Contig5 | 367794 | 373249 | + | 3915 |  | 50.98 | 39.33 | 46.60 | 17.53 | 14.90 | 16.12 | 1.50 | 0.000 | - && Q99046.1 RecName: Full=Laccase-2; AltName: Full=Benzenediol:oxygen oxidoreductase 2; AltName: Full=Diphenol oxidase 2; AltName: Full=Urishiol oxidase 2; Flags: Precursor && PF07731:Multicopper oxidase\|PF00394:Multicopper oxidase\|PF07732:Multicopper oxidase\|PF08487:Vault protein inter-alpha-trypsin domain\|PF13768:von Willebrand factor type A domain |
| A07744 | Contig5 | 373633 | 377232 | + | 3321 |  | 2.82 | 1.72 | 5.17 | 0.51 | 0.56 | 0.50 | 2.63 | 0.000 | - && A6X935.2 RecName: Full=Inter alpha-trypsin inhibitor, heavy chain 4; Short=ITI heavy chain H4; Short=ITI-HC4; Short=Inter-alpha-inhibitor heavy chain 4; Flags: Precursor && PF13768:von Willebrand factor type A domain |
| A07748 | Contig5 | 387116 | 387540 | - | 288 |  | 13.35 | 34.16 | 20.62 | 241.53 | 110.51 | 124.32 | -2.81 | 0.000 | - && - && - |
| A07749 | Contig5 | 388011 | 400177 | - | 3306 |  | 18.13 | 20.74 | 19.51 | 51.53 | 35.90 | 43.08 | -1.16 | 0.000 | - && P83534.2 RecName: Full=Bifunctional ribokinase/ribose-5-phosphate isomerase A; Includes: RecName: Full=Ribokinase; Includes: RecName: Full=Ribose-5-phosphate isomerase A; AltName: Full=Phosphoriboisomerase A; Short=PRI && PF00294:pfkB family carbohydrate kinase\|PF05426:Alginate lyase |
| A07751 | Contig5 | 34703 | 36385 | + | 1344 |  | 0.66 | 0.90 | 0.48 | 3.20 | 6.97 | 1.37 | -2.50 | 0.000 | - && O34371.1 RecName: Full=Putative oxidoreductase YteT; Flags: Precursor && PF02894:Oxidoreductase family, C-terminal alpha/beta domain\|PF01408:Oxidoreductase family, NAD-binding Rossmann fold |
| A07756 | Contig5 | 431338 | 433526 | - | 1551 |  | 2986.57 | 2728.47 | 2430.87 | 35.69 | 79.85 | 12.50 | 5.99 | 0.000 | - && Q12718.1 RecName: Full=Laccase-2; AltName: Full=Benzenediol:oxygen oxidoreductase 2; AltName: Full=Diphenol oxidase 2; AltName: Full=Laccase I; AltName: Full=Urishiol oxidase 2; Flags: Precursor && PF00394:Multicopper oxidase\|PF07731:Multicopper oxidase\|PF07732:Multicopper oxidase |
| A07770 | Contig5 | 477206 | 481888 | + | 3333 |  | 7.99 | 10.27 | 8.96 | 24.63 | 28.90 | 23.88 | -1.51 | 0.000 | - && P37967.2 RecName: Full=Para-nitrobenzyl esterase; AltName: Full=Intracellular esterase B; AltName: Full=PNB carboxy-esterase; Short=PNBCE && PF00135:Carboxylesterase family |
| A07771 | Contig5 | 482129 | 485479 | + | 1557 |  | 58.71 | 53.00 | 50.87 | 8.53 | 11.66 | 6.88 | 2.59 | 0.000 | - && P47173.1 RecName: Full=Uncharacterized protein YJR142W && PF00293:NUDIX domain |
| A07775 | Contig5 | 495850 | 497401 | + | 1146 |  | 8.95 | 11.13 | 7.01 | 2.37 | 2.86 | 2.22 | 1.86 | 0.000 | - && Q01752.1 RecName: Full=Aryl-alcohol dehydrogenase [NADP(+)]; Short=AAD && PF00248:Aldo/keto reductase family |
| A07778 | Contig5 | 510015 | 510614 | - | 600 |  | 17.09 | 12.38 | 17.20 | 9.04 | 5.46 | 4.97 | 1.26 | 0.000 | - && - && - |
| A07784 | Contig5 | 49390 | 50981 | - | 1041 |  | 5.11 | 3.18 | 2.81 | 45.18 | 91.81 | 58.25 | -4.14 | 0.000 | - && - && PF03171:2OG-Fe(II) oxygenase superfamily\|PF14226:non-haem dioxygenase in morphine synthesis N-terminal |
| A07788 | Contig5 | 554194 | 555006 | - | 762 |  | 17.60 | 27.80 | 16.54 | 4.17 | 3.19 | 5.30 | 2.29 | 0.000 | - && - && PF13417:Glutathione S-transferase, N-terminal domain |
| A07791 | Contig5 | 564944 | 566814 | + | 1503 |  | 18.43 | 18.70 | 14.04 | 35.64 | 32.82 | 49.11 | -1.20 | 0.000 | - && Q7ZYQ6.1 RecName: Full=DDB1- and CUL4-associated factor 13; AltName: Full=WD repeat and SOF domain-containing protein 1 && PF04158:Sof1-like domain\|PF00400:WD domain, G-beta repeat |
| A07792 | Contig5 | 567249 | 568340 | + | 891 |  | 14.94 | 23.55 | 15.99 | 31.38 | 48.85 | 48.36 | -1.24 | 0.000 | - && P04963.3 RecName: Full=Chloroperoxidase; AltName: Full=Chloride peroxidase; Short=CPO; Flags: Precursor && PF01328:Peroxidase, family 2 |
| A07810 | Contig5 | 614070 | 616481 | - | 1899 |  | 42.11 | 43.08 | 28.72 | 12.90 | 9.81 | 16.96 | 1.52 | 0.000 | - && P65822.1 RecName: Full=Putative hydrolase Mb2247c; Flags: Precursor >P9WHR4.1 RecName: Full=Carboxylesterase B; Flags: Precursor >P9WHR5.1 RecName: Full=Carboxylesterase B; Flags: Precursor && PF00561:alpha/beta hydrolase fold |
| A07814 | Contig5 | 630398 | 631248 | - | 657 |  | 1.95 | 2.14 | 1.25 | 0.57 | 0.28 | 0.94 | 1.58 | 0.041 | - && - && - |
| A07821 | Contig5 | 656972 | 657505 | + | 480 |  | 7.40 | 6.27 | 8.94 | 3.51 | 3.32 | 0.73 | 1.58 | 0.003 | - && - && - |
| A07830 | Contig5 | 687861 | 688819 | - | 735 |  | 9.39 | 7.65 | 10.32 | 124.92 | 76.16 | 127.40 | -3.59 | 0.000 | - && A0A0A7EQR3.1 RecName: Full=4-O-methyl-glucuronoyl methylesterase; AltName: Full=Glucuronoyl esterase; Short=GE; Flags: Precursor && PF00734:Fungal cellulose binding domain |
| A07831 | Contig5 | 689561 | 691162 | + | 1440 |  | 14.31 | 12.41 | 12.94 | 112.32 | 74.30 | 156.62 | -3.11 | 0.000 | - && A0A0A7EQR3.1 RecName: Full=4-O-methyl-glucuronoyl methylesterase; AltName: Full=Glucuronoyl esterase; Short=GE; Flags: Precursor && PF00734:Fungal cellulose binding domain |
| A07832 | Contig5 | 693882 | 695406 | + | 1239 |  | 261.27 | 180.12 | 229.00 | 103.76 | 78.35 | 110.99 | 1.19 | 0.000 | - && Q68EH8.1 RecName: Full=FAD synthase; AltName: Full=FAD pyrophosphorylase; AltName: Full=FMN adenylyltransferase; AltName: Full=Flavin adenine dinucleotide synthase; Includes: RecName: Full=Molybdenum cofactor biosynthesis protein-like region; Includes: RecName: Full=FAD synthase region && PF01507:Phosphoadenosine phosphosulfate reductase family |
| A07833 | Contig5 | 696151 | 697017 | - | 867 |  | 12.74 | 11.00 | 5.90 | 2.80 | 3.46 | 3.14 | 1.66 | 0.000 | - && - && - |
| A07837 | Contig5 | 709340 | 711510 | - | 1500 |  | 2.10 | 1.81 | 1.28 | 24.43 | 34.82 | 16.26 | -3.86 | 0.000 | - && G3Y416.1 RecName: Full=Cytochrome P450 monooxygenase yanC; AltName: Full=Yanuthone D synthesis protein C; Flags: Precursor && PF00067:Cytochrome P450 |
| A07847 | Contig5 | 742833 | 749367 | - | 4749 |  | 3.59 | 4.59 | 6.08 | 20.22 | 17.78 | 12.42 | -1.82 | 0.000 | - && Q9USG8.1 RecName: Full=Meiotically up-regulated gene 190 protein && PF00168:C2 domain |
| A07876 | Contig5 | 848475 | 849996 | + | 1452 |  | 6.38 | 5.39 | 5.47 | 2.64 | 3.09 | 1.51 | 1.25 | 0.000 | - && - && - |
| A07877 | Contig5 | 850914 | 851566 | + | 459 |  | 10.53 | 31.71 | 22.29 | 34.63 | 90.76 | 115.29 | -1.90 | 0.000 | - && - && PF16850:Peptidase inhibitor I66 |
| A07888 | Contig5 | 896130 | 899108 | + | 1821 |  | 3.90 | 3.31 | 4.82 | 10.99 | 15.06 | 10.60 | -1.61 | 0.000 | - && Q12119.1 RecName: Full=Purine-cytosine permease FCY22; Short=PCP FCY22; AltName: Full=Cytosine/purine transport protein FCY22; AltName: Full=Fluorocytosine resistance protein 22 && PF02133:Permease for cytosine/purines, uracil, thiamine, allantoin |
| A07899 | Contig5 | 931041 | 935060 | - | 939 |  | 1.58 | 1.50 | 1.65 | 96.18 | 66.59 | 92.43 | -5.76 | 0.000 | - && - && - |
| A07902 | Contig5 | 941020 | 943462 | - | 1575 |  | 102.67 | 277.20 | 69.66 | 34.31 | 58.19 | 67.76 | 1.49 | 0.026 | - && Q27519.1 RecName: Full=Putative cytochrome P450 CYP13A7 && PF00067:Cytochrome P450 |
| A07913 | Contig5 | 969747 | 972433 | - | 2121 |  | 61.64 | 150.65 | 27.00 | 13.67 | 10.02 | 14.85 | 2.63 | 0.000 | - && J9R393.2 RecName: Full=Microbial Terpene synthase-like protein 13; Short=SmMTPSL13 && PF03936:Terpene synthase family, metal binding domain |
| A07914 | Contig5 | 977475 | 980030 | - | 1950 |  | 159.43 | 308.84 | 97.58 | 35.58 | 27.84 | 51.44 | 2.30 | 0.000 | - && Q47944.1 RecName: Full=L-sorbose 1-dehydrogenase; Short=SDH && PF05199:GMC oxidoreductase\|PF00732:GMC oxidoreductase |
| A07917 | Contig5 | 94329 | 95520 | - | 1020 |  | 54.23 | 84.44 | 21.14 | 15.58 | 10.19 | 21.68 | 1.75 | 0.000 | - && Q4WZ70.1 RecName: Full=Chanoclavine-I aldehyde reductase easA; AltName: Full=Ergot alkaloid synthesis protein A; AltName: Full=Old yellow enzyme 3 homolog && PF00724:NADH:flavin oxidoreductase / NADH oxidase family |
| A07919 | Contig5 | 995407 | 998041 | - | 1524 |  | 98.93 | 157.24 | 69.29 | 37.24 | 37.47 | 60.69 | 1.27 | 0.000 | - && G3Y416.1 RecName: Full=Cytochrome P450 monooxygenase yanC; AltName: Full=Yanuthone D synthesis protein C; Flags: Precursor && PF00067:Cytochrome P450 |
| A07920 | Contig5 | 1000265 | 1001712 | + | 1161 |  | 57.24 | 111.28 | 47.92 | 31.49 | 34.99 | 36.58 | 1.07 | 0.000 | - && P38256.1 RecName: Full=Uncharacterized protein YBR096W && PF13279:Thioesterase-like superfamily |
| A07924 | Contig5 | 1015440 | 1016588 | - | 981 |  | 25.63 | 19.44 | 13.41 | 1.05 | 0.95 | 2.86 | 3.59 | 0.000 | - && - && PF06330:Trichodiene synthase (TRI5) |
| A07925 | Contig5 | 1020565 | 1021714 | - | 981 |  | 43.82 | 40.11 | 49.82 | 19.25 | 9.35 | 14.22 | 1.64 | 0.000 | - && - && PF06330:Trichodiene synthase (TRI5) |
| A07933 | Contig5 | 1051336 | 1052428 | - | 969 |  | 47.32 | 55.32 | 21.12 | 78.15 | 136.12 | 111.12 | -1.39 | 0.000 | - && P47818.1 RecName: Full=Protein CCC1; AltName: Full=Cross-complementer of CSG1 protein 1 && PF01988:VIT family |
| A07940 | Contig5 | 1075368 | 1077754 | + | 1620 |  | 45.22 | 44.62 | 39.42 | 81.55 | 151.57 | 105.58 | -1.39 | 0.000 | - && Q10286.1 RecName: Full=Myo-inositol transporter 1 && PF00083:Sugar (and other) transporter |
| A07941 | Contig5 | 1078117 | 1079956 | - | 1332 |  | 23.47 | 22.16 | 18.11 | 7.93 | 9.21 | 7.51 | 1.37 | 0.000 | - && Q80UY1.1 RecName: Full=Carnosine N-methyltransferase && PF07942:N2227-like protein |
| A07949 | Contig5 | 1117584 | 1119430 | - | 1101 |  | 1.52 | 1.19 | 1.08 | 16.47 | 20.15 | 9.09 | -3.59 | 0.000 | - && O74213.1 RecName: Full=Endopolygalacturonase I; AltName: Full=Pectinase 1; AltName: Full=Polygalacturonase I; Short=PG-I; Flags: Precursor && PF00295:Glycosyl hydrolases family 28 |
| A07956 | Contig5 | 1149201 | 1150380 | + | 852 |  | 46.76 | 63.86 | 31.74 | 25.13 | 21.21 | 19.06 | 1.12 | 0.000 | - && - && - |
| A07967 | Contig5 | 1175323 | 1182569 | + | 5643 |  | 45.66 | 44.28 | 26.34 | 7.22 | 6.64 | 6.64 | 2.50 | 0.000 | - && P41820.1 RecName: Full=Brefeldin A resistance protein && PF00856:SET domain\|PF00005:ABC transporter\|PF14510:ABC-transporter extracellular N-terminal\|PF01061:ABC-2 type transporter\|PF06422:CDR ABC transporter |
| A07971 | Contig5 | 1200883 | 1205352 | + | 4257 |  | 15.47 | 16.06 | 10.51 | 6.81 | 5.06 | 6.62 | 1.19 | 0.000 | - && - && - |
| A07985 | Contig5 | 1237996 | 1239961 | - | 1326 |  | 3.05 | 2.20 | 2.96 | 4.37 | 12.35 | 2.05 | -1.19 | 0.023 | - && Q9HDX2.1 RecName: Full=Uncharacterized lactate 2-monooxygenase PB1A11.03 && PF01070:FMN-dependent dehydrogenase |
| A07987 | Contig5 | 1245303 | 1246603 | + | 966 |  | 7.76 | 4.78 | 5.39 | 16.84 | 18.90 | 14.17 | -1.48 | 0.000 | - && Q01752.1 RecName: Full=Aryl-alcohol dehydrogenase [NADP(+)]; Short=AAD && PF00248:Aldo/keto reductase family |
| A07989 | Contig5 | 1250646 | 1252427 | - | 1461 |  | 4.05 | 4.19 | 2.25 | 1.09 | 2.82 | 1.02 | 1.09 | 0.020 | - && O13716.2 RecName: Full=Glucan endo-1,3-alpha-glucosidase agn1; AltName: Full=Endo-1,3-alpha-glucanase agn1; Flags: Precursor && PF03659:Glycosyl hydrolase family 71 |
| A07995 | Contig5 | 126583 | 130628 | - | 2088 |  | 9.78 | 7.50 | 10.54 | 17.51 | 23.00 | 27.99 | -1.30 | 0.000 | - && P17576.1 RecName: Full=Polyporopepsin; AltName: Full=Aspartic proteinase && PF00026:Eukaryotic aspartyl protease |
| A07996 | Contig5 | 1268226 | 1269805 | - | 1152 |  | 1126.32 | 1144.70 | 1133.15 | 159.80 | 109.37 | 127.07 | 3.10 | 0.000 | - && Q01752.1 RecName: Full=Aryl-alcohol dehydrogenase [NADP(+)]; Short=AAD && PF00248:Aldo/keto reductase family |
| A08004 | Contig5 | 1293427 | 1294646 | - | 888 |  | 11.21 | 11.53 | 6.89 | 5.16 | 3.69 | 4.05 | 1.20 | 0.000 | - && - && PF01612:3'-5' exonuclease |
| A08006 | Contig5 | 132404 | 134201 | + | 1242 |  | 3.89 | 5.90 | 3.75 | 23.41 | 40.17 | 14.84 | -2.53 | 0.000 | - && P17576.1 RecName: Full=Polyporopepsin; AltName: Full=Aspartic proteinase && PF00026:Eukaryotic aspartyl protease |
| A08017 | Contig5 | 134410 | 136238 | - | 1539 |  | 172.35 | 131.05 | 62.44 | 19.50 | 17.46 | 20.98 | 2.66 | 0.000 | - && - && PF13391:HNH endonuclease |
| A08030 | Contig5 | 1377991 | 1379842 | - | 1797 |  | 195.40 | 137.31 | 208.52 | 94.80 | 89.03 | 81.56 | 1.03 | 0.000 | - && - && - |
| A08032 | Contig5 | 1387990 | 1390303 | - | 1644 |  | 85.89 | 82.13 | 76.57 | 34.07 | 42.82 | 26.74 | 1.24 | 0.000 | - && Q9VS79.2 RecName: Full=Cytochrome P450 4d8; AltName: Full=CYPIVD8 && PF00067:Cytochrome P450 |
| A08033 | Contig5 | 1392710 | 1394305 | - | 1596 |  | 79.27 | 66.04 | 61.07 | 17.46 | 11.73 | 18.81 | 2.10 | 0.000 | - && - && - |
| A08046 | Contig5 | 1451128 | 1453055 | - | 1809 |  | 2.56 | 2.05 | 2.37 | 4.19 | 5.33 | 5.68 | -1.12 | 0.000 | - && - && - |
| A08055 | Contig5 | 1545854 | 1546892 | - | 861 |  | 106.50 | 92.58 | 100.79 | 435.12 | 569.27 | 452.15 | -2.28 | 0.000 | - && - && PF06912:Protein of unknown function (DUF1275) |
| A08062 | Contig5 | 1574620 | 1577501 | - | 1899 |  | 444.57 | 553.43 | 411.05 | 67.75 | 73.55 | 70.11 | 2.74 | 0.000 | - && Q4P7G1.1 RecName: Full=Eukaryotic translation initiation factor 3 subunit G; Short=eIF3g; AltName: Full=Eukaryotic translation initiation factor 3 RNA-binding subunit; Short=eIF-3 RNA-binding subunit; AltName: Full=Translation initiation factor eIF3 p33 subunit homolog; Short=eIF3 p33 homolog && PF00076:RNA recognition motif. (a.k.a. RRM, RBD, or RNP domain)\|PF01370:NAD dependent epimerase/dehydratase family\|PF12353:Eukaryotic translation initiation factor 3 subunit G |
| A08064 | Contig5 | 1579125 | 1580614 | - | 1350 |  | 345.62 | 473.53 | 236.22 | 88.44 | 71.21 | 169.93 | 1.68 | 0.000 | - && Q19QT7.1 RecName: Full=Cystathionine gamma-lyase; AltName: Full=Cysteine-protein sulfhydrase; AltName: Full=Gamma-cystathionase && PF01053:Cys/Met metabolism PLP-dependent enzyme |
| A08077 | Contig5 | 1645837 | 1646908 | + | 837 |  | 33.69 | 33.22 | 32.31 | 135.50 | 222.79 | 96.46 | -2.20 | 0.000 | - && A6THU5.1 RecName: Full=4-hydroxy-2-oxo-heptane-1,7-dioate aldolase; AltName: Full=2,4-dihydroxyhept-2-ene-1,7-dioic acid aldolase; Short=HHED aldolase; AltName: Full=4-hydroxy-2-ketoheptane-1,7-dioate aldolase; Short=HKHD aldolase && PF03328:HpcH/HpaI aldolase/citrate lyase family |
| A08082 | Contig5 | 1656818 | 1658931 | + | 1473 |  | 361.48 | 249.78 | 375.45 | 47.86 | 39.21 | 46.05 | 2.89 | 0.000 | - && Q9C0V1.1 RecName: Full=Ammonium transporter 1 && PF00909:Ammonium Transporter Family |
| A08098 | Contig5 | 1699485 | 1704630 | - | 3006 |  | 9.91 | 12.29 | 10.18 | 84.01 | 79.81 | 70.77 | -2.86 | 0.000 | - && Q9I3H5.1 RecName: Full=Baeyer-Villiger monooxygenase; Short=BVMO && PF13738:Pyridine nucleotide-disulphide oxidoreductase |
| A08120 | Contig5 | 1795594 | 1797042 | - | 1449 |  | 17.83 | 15.17 | 28.43 | 49.81 | 49.62 | 33.01 | -1.11 | 0.000 | - && - && - |
| A08136 | Contig5 | 1843694 | 1845247 | - | 735 |  | 15.56 | 17.35 | 16.41 | 51.52 | 36.30 | 41.19 | -1.39 | 0.000 | - && - && - |
| A08137 | Contig5 | 1846686 | 1848091 | - | 921 |  | 1.82 | 1.53 | 2.48 | 0.41 | 0.41 | 0.29 | 2.41 | 0.001 | - && - && - |
| A08151 | Contig5 | 1887938 | 1889737 | + | 1326 |  | 17.77 | 15.97 | 15.64 | 7.12 | 4.31 | 4.43 | 1.64 | 0.000 | - && - && - |
| A08163 | Contig5 | 1936443 | 1938470 | + | 1653 |  | 27.50 | 21.92 | 24.04 | 9.39 | 9.91 | 10.35 | 1.31 | 0.000 | - && - && PF08568:Uncharacterised protein family, YAP/Alf4/glomulin |
| A08165 | Contig5 | 1947603 | 1949100 | + | 1329 |  | 62.62 | 61.34 | 70.39 | 26.10 | 20.22 | 28.66 | 1.37 | 0.000 | - && - && PF13523:Acetyltransferase (GNAT) domain |
| A08166 | Contig5 | 1950027 | 1951400 | + | 1023 |  | 18.22 | 18.74 | 19.38 | 10.42 | 8.42 | 5.40 | 1.22 | 0.000 | - && P43903.2 RecName: Full=Quinone oxidoreductase; AltName: Full=NADPH:quinone reductase && PF00107:Zinc-binding dehydrogenase |
| A08171 | Contig5 | 1963696 | 1966575 | - | 2049 |  | 84.70 | 72.07 | 76.46 | 38.15 | 36.69 | 29.30 | 1.16 | 0.000 | - && - && PF01636:Phosphotransferase enzyme family |
| A08172 | Contig5 | 185561 | 186523 | - | 963 |  | 3.69 | 5.11 | 3.98 | 12.43 | 18.37 | 11.48 | -1.73 | 0.000 | - && - && - |
| A08176 | Contig5 | 1978224 | 1980325 | - | 1794 |  | 20.23 | 27.59 | 26.33 | 60.66 | 44.72 | 64.18 | -1.19 | 0.000 | - && Q8T9S7.1 RecName: Full=Phosphatidylinositol 3,4,5-trisphosphate 3-phosphatase and dual-specificity protein phosphatase PTEN; AltName: Full=Pten 3-phosphoinositide phosphatase alpha && - |
| A08188 | Contig5 | 2025278 | 2028607 | - | 1995 |  | 10.23 | 9.36 | 8.79 | 20.29 | 31.30 | 13.28 | -1.19 | 0.000 | - && Q0CTV2.1 RecName: Full=Probable alpha-L-arabinofuranosidase A; Short=ABF A; Short=Arabinosidase A; Flags: Precursor && PF06964:Alpha-L-arabinofuranosidase C-terminus |
| A08191 | Contig5 | 2041889 | 2043311 | + | 1134 |  | 9.13 | 11.69 | 8.46 | 104.05 | 54.89 | 124.91 | -3.28 | 0.000 | - && A2R2G1.1 RecName: Full=Leucine aminopeptidase 1; AltName: Full=Leucyl aminopeptidase 1; Short=LAP1; Flags: Precursor && PF04389:Peptidase family M28 |
| A08196 | Contig5 | 2055502 | 2056808 | - | 1074 |  | 15.52 | 15.52 | 13.61 | 224.25 | 287.02 | 68.07 | -3.70 | 0.000 | - && Q9UR19.1 RecName: Full=Versatile peroxidase VPL1; AltName: Full=Versatile liquid phase peroxidase 1; Flags: Precursor && PF11895:Domain of unknown function (DUF3415)\|PF00141:Peroxidase |
| A08202 | Contig5 | 2081144 | 2086170 | - | 2340 |  | 3.62 | 3.17 | 3.28 | 47.27 | 37.84 | 41.52 | -3.65 | 0.000 | - && Q0CTV2.1 RecName: Full=Probable alpha-L-arabinofuranosidase A; Short=ABF A; Short=Arabinosidase A; Flags: Precursor && PF06964:Alpha-L-arabinofuranosidase C-terminus |
| A08206 | Contig5 | 191140 | 192222 | + | 915 |  | 25.54 | 37.08 | 19.17 | 143.26 | 60.36 | 62.05 | -1.70 | 0.000 | - && - && PF13668:Ferritin-like domain |
| A08214 | Contig5 | 2121044 | 2122935 | + | 1818 |  | 1.57 | 1.77 | 1.51 | 3.45 | 3.76 | 3.96 | -1.20 | 0.000 | - && - && PF00271:Helicase conserved C-terminal domain\|PF00270:DEAD/DEAH box helicase |
| A08217 | Contig5 | 193884 | 195420 | - | 1299 |  | 1479.88 | 1722.36 | 782.49 | 109.62 | 72.14 | 239.36 | 3.24 | 0.000 | - && - && - |
| A08226 | Contig5 | 2170559 | 2171802 | + | 1119 |  | 8.90 | 12.02 | 10.37 | 4.60 | 4.77 | 5.49 | 1.07 | 0.000 | - && - && - |
| A08238 | Contig5 | 2203028 | 2205493 | - | 2346 |  | 71.70 | 70.69 | 74.49 | 129.72 | 143.53 | 209.37 | -1.15 | 0.000 | - && Q9FNM7.2 RecName: Full=DEAD-box ATP-dependent RNA helicase 26 && PF00270:DEAD/DEAH box helicase\|PF00271:Helicase conserved C-terminal domain |
| A08240 | Contig5 | 2210117 | 2212629 | + | 1632 |  | 7.79 | 7.44 | 3.92 | 4.30 | 2.01 | 2.80 | 1.07 | 0.004 | - && Q4WAZ6.1 RecName: Full=Multifunctional cytochrome P450 monooxygenase af510; AltName: Full=Fumagillin bioynthesis cluster P450 monooxygenase; Short=Fma-P450 && PF00067:Cytochrome P450 |
| A08244 | Contig5 | 2221745 | 2223765 | + | 1530 |  | 19.27 | 21.13 | 19.17 | 8.98 | 8.75 | 9.52 | 1.13 | 0.000 | - && Q9Y7T0.2 RecName: Full=Uncharacterized J domain-containing protein C63.03 && PF00226:DnaJ domain |
| A08247 | Contig5 | 2245828 | 2248233 | - | 1884 |  | 350.03 | 291.04 | 379.90 | 66.20 | 46.16 | 62.42 | 2.55 | 0.000 | - && Q09920.1 RecName: Full=Iron transport multicopper oxidase fio1; Flags: Precursor && PF07732:Multicopper oxidase\|PF07731:Multicopper oxidase\|PF00394:Multicopper oxidase |
| A08248 | Contig5 | 2249384 | 2250885 | + | 1158 |  | 1041.97 | 903.41 | 1213.97 | 183.52 | 160.71 | 199.24 | 2.54 | 0.000 | - && P40088.1 RecName: Full=Plasma membrane iron permease && PF03239:Iron permease FTR1 family |
| A08252 | Contig5 | 2266210 | 2268447 | - | 1998 |  | 92.73 | 77.28 | 104.29 | 176.09 | 232.76 | 141.43 | -1.00 | 0.000 | - && P37967.2 RecName: Full=Para-nitrobenzyl esterase; AltName: Full=Intracellular esterase B; AltName: Full=PNB carboxy-esterase; Short=PNBCE && PF00135:Carboxylesterase family |
| A08256 | Contig5 | 2279224 | 2287080 | - | 2286 |  | 3.28 | 2.63 | 3.56 | 6.58 | 7.08 | 6.22 | -1.07 | 0.000 | - && - && - |
| A08261 | Contig5 | 207323 | 209633 | - | 1935 |  | 21.40 | 20.03 | 27.43 | 83.01 | 85.24 | 77.51 | -1.84 | 0.000 | - && O74771.2 RecName: Full=Protein dip1 && PF09431:Protein of unknown function (DUF2013) |
| A08266 | Contig5 | 2317183 | 2319628 | - | 1680 |  | 3.76 | 4.00 | 3.21 | 1.34 | 1.89 | 1.78 | 1.13 | 0.001 | - && - && PF03663:Glycosyl hydrolase family 76 |
| A08283 | Contig5 | 213470 | 214937 | + | 1344 |  | 2.05 | 2.39 | 1.43 | 4.17 | 5.22 | 5.55 | -1.35 | 0.000 | - && - && PF01753:MYND finger |
| A08284 | Contig5 | 2380462 | 2382001 | + | 1332 |  | 5.18 | 5.80 | 2.06 | 1.97 | 1.19 | 1.91 | 1.36 | 0.002 | - && - && - |
| A08285 | Contig5 | 2383513 | 2385395 | - | 1431 |  | 2.34 | 4.42 | 4.21 | 6.34 | 5.95 | 10.73 | -1.07 | 0.002 | - && P30838.3 RecName: Full=Aldehyde dehydrogenase, dimeric NADP-preferring; AltName: Full=ALDHIII; AltName: Full=Aldehyde dehydrogenase 3; AltName: Full=Aldehyde dehydrogenase family 3 member A1 && PF00171:Aldehyde dehydrogenase family |
| A08286 | Contig5 | 2387649 | 2389640 | + | 1476 |  | 102.41 | 84.13 | 115.80 | 39.08 | 45.41 | 32.52 | 1.37 | 0.000 | - && P34946.1 RecName: Full=Carboxypeptidase S1 && PF00450:Serine carboxypeptidase |
| A08291 | Contig5 | 2405903 | 2407821 | + | 1674 |  | 62.91 | 68.30 | 63.74 | 149.40 | 121.12 | 157.27 | -1.13 | 0.000 | - && O94512.1 RecName: Full=Oxysterol-binding protein-like protein 1 && PF01237:Oxysterol-binding protein |
| A08300 | Contig5 | 2434996 | 2436373 | + | 1266 |  | 6.54 | 7.77 | 7.14 | 26.88 | 71.58 | 29.81 | -2.58 | 0.000 | - && K5XDZ6.1 RecName: Full=4-O-methyl-glucuronoyl methylesterase; AltName: Full=Glucuronoyl esterase; Short=GCE; Short=GE; Flags: Precursor && - |
| A08302 | Contig5 | 2439823 | 2440604 | + | 573 |  | 19.62 | 18.57 | 17.86 | 112.42 | 111.58 | 83.32 | -2.46 | 0.000 | - && - && - |
| A08306 | Contig5 | 220785 | 223754 | - | 1737 |  | 15.50 | 15.26 | 21.83 | 37.79 | 66.18 | 37.84 | -1.43 | 0.000 | - && P55306.1 RecName: Full=Catalase && PF06628:Catalase-related immune-responsive\|PF00199:Catalase |
| A08310 | Contig5 | 2456691 | 2459237 | - | 1827 |  | 100.38 | 131.71 | 69.35 | 330.08 | 262.39 | 247.86 | -1.48 | 0.000 | - && Q6QWR1.1 RecName: Full=Pyranose 2-oxidase; Short=P2O; Short=P2Ox; Short=POD; Short=POx; Short=PROD; Short=Pyranose oxidase; AltName: Full=FAD-oxidoreductase; AltName: Full=Glucose 2-oxidase; AltName: Full=Pyranose:oxygen 2-oxidoreductase; Flags: Precursor && PF05199:GMC oxidoreductase |
| A08311 | Contig5 | 2461733 | 2464940 | + | 2634 |  | 62.89 | 98.67 | 66.52 | 287.37 | 199.59 | 289.93 | -1.77 | 0.000 | - && P84193.3 RecName: Full=Aldos-2-ulose dehydratase; AltName: Full=D-arabino-hex-2-ulose dehydratase; AltName: Full=Pyranosone dehydratase && - |
| A08319 | Contig5 | 2493339 | 2498861 | + | 3942 |  | 7.08 | 9.27 | 7.93 | 4.10 | 4.82 | 2.69 | 1.06 | 0.000 | - && - && PF01753:MYND finger |
| A08329 | Contig5 | 2541921 | 2545468 | + | 2280 |  | 456.60 | 684.27 | 70.64 | 8.98 | 10.31 | 9.55 | 5.39 | 0.000 | - && - && - |
| A08340 | Contig5 | 19659 | 24893 | - | 2976 |  | 20.24 | 22.20 | 20.29 | 46.87 | 62.06 | 118.04 | -1.86 | 0.000 | - && - && - |
| A08349 | Contig5 | 279455 | 281826 | - | 1731 |  | 2.28 | 2.55 | 3.17 | 1.08 | 1.46 | 0.20 | 1.54 | 0.004 | - && Q70GH4.1 RecName: Full=Tripeptidyl-peptidase sed3; AltName: Full=Sedolisin-C; Flags: Precursor && PF09286:Pro-kumamolisin, activation domain |
| A08351 | Contig5 | 25081 | 26725 | - | 1344 |  | 15.19 | 19.20 | 16.24 | 30.26 | 32.11 | 40.29 | -1.02 | 0.000 | - && B0DWM8.1 RecName: Full=Ribosome biogenesis protein YTM1 && PF08154:NLE (NUC135) domain |
| A08353 | Contig5 | 286789 | 289219 | - | 1788 |  | 5.13 | 8.08 | 9.86 | 25.57 | 20.99 | 14.53 | -1.40 | 0.000 | - && Q10988.1 RecName: Full=UV-damage endonuclease; Short=UVDE && PF03851:UV-endonuclease UvdE |
| A08360 | Contig5 | 324236 | 325485 | + | 1089 |  | 1.54 | 1.84 | 0.59 | 5.84 | 5.33 | 3.38 | -1.87 | 0.000 | - && - && - |
| A08364 | Contig5 | 334954 | 337063 | - | 1437 |  | 19.14 | 31.37 | 14.75 | 7.22 | 6.84 | 14.66 | 1.18 | 0.001 | - && - && PF09365:Conserved hypothetical protein (DUF2461) |
| A08365 | Contig5 | 338847 | 341051 | + | 1581 |  | 3368.47 | 3955.14 | 1596.16 | 621.06 | 117.12 | 431.85 | 2.93 | 0.000 | - && Q99046.1 RecName: Full=Laccase-2; AltName: Full=Benzenediol:oxygen oxidoreductase 2; AltName: Full=Diphenol oxidase 2; AltName: Full=Urishiol oxidase 2; Flags: Precursor && PF07731:Multicopper oxidase\|PF07732:Multicopper oxidase\|PF00394:Multicopper oxidase |
| A08368 | Contig5 | 343809 | 344366 | + | 495 |  | 1542.79 | 1606.21 | 1265.06 | 726.43 | 442.33 | 465.03 | 1.43 | 0.000 | - && - && - |
| A08370 | Contig5 | 350076 | 353132 | + | 1998 |  | 407.79 | 365.33 | 421.13 | 450.45 | 794.19 | 1250.54 | -1.06 | 0.000 | - && A8PDE3.1 RecName: Full=Acetyl-coenzyme A synthetase; AltName: Full=Acetate--CoA ligase; AltName: Full=Acyl-activating enzyme && PF16177:Acetyl-coenzyme A synthetase N-terminus\|PF00501:AMP-binding enzyme\|PF13193:AMP-binding enzyme C-terminal domain |
| A08397 | Contig6 | 666204 | 668226 | - | 1965 |  | 1416.97 | 1054.25 | 888.95 | 418.71 | 186.03 | 293.91 | 1.90 | 0.000 | - && - && PF02179:BAG domain |
| A08398 | Contig6 | 671749 | 674190 | + | 1647 |  | 10.30 | 10.67 | 7.77 | 171.89 | 104.01 | 151.65 | -3.90 | 0.000 | - && P32946.1 RecName: Full=Lipase 2; Flags: Precursor && PF00135:Carboxylesterase family |
| A08399 | Contig6 | 687032 | 689147 | + | 1017 |  | 13072.41 | 12417.04 | 17049.43 | 4088.99 | 6396.53 | 2533.09 | 1.71 | 0.000 | - && Q01982.1 RecName: Full=Glyceraldehyde-3-phosphate dehydrogenase; Short=GAPDH && PF00044:Glyceraldehyde 3-phosphate dehydrogenase, NAD binding domain\|PF02800:Glyceraldehyde 3-phosphate dehydrogenase, C-terminal domain |
| A08410 | Contig6 | 733106 | 735296 | + | 1614 |  | 101.66 | 106.98 | 108.39 | 11.99 | 15.78 | 16.26 | 2.85 | 0.000 | - && Q12718.1 RecName: Full=Laccase-2; AltName: Full=Benzenediol:oxygen oxidoreductase 2; AltName: Full=Diphenol oxidase 2; AltName: Full=Laccase I; AltName: Full=Urishiol oxidase 2; Flags: Precursor && PF07731:Multicopper oxidase\|PF00394:Multicopper oxidase\|PF07732:Multicopper oxidase |
| A08417 | Contig6 | 755854 | 758517 | - | 1809 |  | 5.23 | 5.38 | 5.86 | 19.95 | 13.25 | 15.43 | -1.56 | 0.000 | - && Q00922.1 RecName: Full=Alcohol oxidase; Short=AO; Short=AOX; AltName: Full=Methanol oxidase; Short=MOX && PF00732:GMC oxidoreductase\|PF05199:GMC oxidoreductase |
| A08419 | Contig6 | 759553 | 760340 | - | 669 |  | 6.78 | 6.60 | 5.87 | 10.48 | 13.85 | 24.92 | -1.36 | 0.000 | - && O94255.2 RecName: Full=Carbonic anhydrase; AltName: Full=Carbonate dehydratase && PF00484:Carbonic anhydrase |
| A08427 | Contig6 | 789803 | 791611 | + | 1401 |  | 7.88 | 21.14 | 12.85 | 62.13 | 67.82 | 39.28 | -2.02 | 0.000 | - && - && PF10281:Putative stress-responsive nuclear envelope protein |
| A08440 | Contig6 | 62234 | 64651 | - | 2145 |  | 0.60 | 0.09 | 0.30 | 0.61 | 0.61 | 1.39 | -1.40 | 0.048 | - && Q84LM4.1 RecName: Full=Acylamino-acid-releasing enzyme; Short=AARE; AltName: Full=Oxidized protein hydrolase; Short=OPH && PF00326:Prolyl oligopeptidase family |
| A08449 | Contig6 | 868453 | 869739 | - | 1287 |  | 0.92 | 1.17 | 1.42 | 2.32 | 2.69 | 5.52 | -1.59 | 0.001 | - && - && - |
| A08458 | Contig6 | 892987 | 894099 | + | 1113 |  | 6.56 | 6.40 | 8.70 | 28.90 | 18.34 | 19.32 | -1.62 | 0.000 | - && - && - |
| A08467 | Contig6 | 921262 | 923937 | - | 1974 |  | 29.22 | 22.02 | 22.72 | 13.31 | 11.86 | 7.11 | 1.20 | 0.000 | - && Q68AP4.1 RecName: Full=N-substituted formamide deformylase; Flags: Precursor && PF07969:Amidohydrolase family |
| A08469 | Contig6 | 928440 | 930718 | + | 1797 |  | 8.83 | 10.73 | 12.91 | 76.59 | 81.32 | 79.55 | -2.87 | 0.000 | - && Q3L245.1 RecName: Full=Pyranose dehydrogenase 1; Short=PDH 1; AltName: Full=Pyranose:quinone oxidoreductase 1; Flags: Precursor && PF00732:GMC oxidoreductase\|PF05199:GMC oxidoreductase |
| A08470 | Contig6 | 931177 | 936342 | - | 3216 |  | 12.88 | 9.90 | 11.73 | 27.65 | 34.00 | 20.25 | -1.25 | 0.000 | - && P22533.2 RecName: Full=Beta-mannanase/endoglucanase A; Includes: RecName: Full=Mannan endo-1,4-beta-mannosidase A; AltName: Full=Beta-mannanase; AltName: Full=Endo-1,4-mannanase; Includes: RecName: Full=Endo-1,4-beta-glucanase; AltName: Full=Cellulase; Flags: Precursor && PF04479:RTA1 like protein\|PF12891:Glycoside hydrolase family 44 |
| A08474 | Contig6 | 944917 | 946157 | + | 951 |  | 1.56 | 2.22 | 1.92 | 4.13 | 5.51 | 2.31 | -1.07 | 0.021 | - && - && - |
| A08475 | Contig6 | 954514 | 957497 | - | 2193 |  | 18.39 | 17.30 | 21.24 | 54.61 | 52.97 | 70.23 | -1.64 | 0.000 | - && - && PF00641:Zn-finger in Ran binding protein and others |
| A08478 | Contig6 | 972926 | 973309 | + | 384 |  | 1.03 | 1.57 | 1.19 | 8.28 | 5.85 | 4.34 | -2.29 | 0.000 | - && - && - |
| A08479 | Contig6 | 975662 | 977463 | - | 1143 |  | 11.99 | 16.60 | 16.46 | 26.42 | 33.50 | 39.54 | -1.14 | 0.000 | - && - && PF08190:pre-RNA processing PIH1/Nop17 |
| A08480 | Contig6 | 977970 | 980030 | + | 1215 |  | 34.90 | 43.21 | 32.18 | 84.03 | 73.73 | 123.22 | -1.35 | 0.000 | - && Q9UT09.1 RecName: Full=Phospho-2-dehydro-3-deoxyheptonate aldolase, tyrosine-inhibited; AltName: Full=3-deoxy-D-arabino-heptulosonate 7-phosphate synthase; AltName: Full=DAHP synthase; AltName: Full=Phospho-2-keto-3-deoxyheptonate aldolase && PF00793:DAHP synthetase I family |
| A08519 | Contig6 | 1206748 | 1207951 | - | 924 |  | 7.04 | 4.89 | 8.01 | 53.32 | 82.16 | 50.24 | -3.22 | 0.000 | - && P30887.2 RecName: Full=Acid phosphatase; Flags: Precursor && PF01975:Survival protein SurE |
| A08521 | Contig6 | 1211721 | 1212931 | - | 855 |  | 30.79 | 25.83 | 35.58 | 119.30 | 95.69 | 114.45 | -1.84 | 0.000 | - && B2VA83.1 RecName: Full=5'-nucleotidase SurE; AltName: Full=Nucleoside 5'-monophosphate phosphohydrolase && PF01975:Survival protein SurE |
| A08533 | Contig6 | 1259843 | 1262243 | + | 1641 |  | 5.89 | 5.87 | 4.51 | 14.64 | 21.39 | 7.49 | -1.42 | 0.000 | - && P11707.2 RecName: Full=Cytochrome P450 3A6; AltName: Full=CYPIIIA6; AltName: Full=Cytochrome P450-3C && PF00067:Cytochrome P450 |
| A08536 | Contig6 | 1266858 | 1267410 | + | 435 |  | 9.52 | 7.85 | 9.24 | 20.20 | 43.25 | 12.71 | -1.52 | 0.000 | - && A0A097ZPE4.1 RecName: Full=Cytochrome P450 monooxygenase andK; AltName: Full=Anditomin synthesis protein K; Flags: Precursor && PF00067:Cytochrome P450 |
| A08545 | Contig6 | 1296298 | 1296733 | - | 264 |  | 58.27 | 44.11 | 47.75 | 19.83 | 19.86 | 20.28 | 1.32 | 0.000 | - && O14280.2 RecName: Full=Uncharacterized bolA-like protein C8C9.11 && - |
| A08552 | Contig6 | 1310214 | 1310811 | + | 474 |  | 8760.69 | 12208.60 | 7251.32 | 5675.04 | 2727.74 | 3470.91 | 1.25 | 0.000 | - && O14368.1 RecName: Full=Heat shock protein 16; AltName: Full=16 kDa heat shock protein && PF00011:Hsp20/alpha crystallin family |
| A08556 | Contig6 | 1322690 | 1323646 | + | 798 |  | 16.68 | 17.23 | 9.62 | 54.71 | 80.00 | 37.06 | -1.98 | 0.000 | - && - && PF00775:Dioxygenase |
| A08559 | Contig6 | 1327615 | 1329302 | + | 1632 |  | 2.48 | 3.08 | 3.25 | 15.87 | 16.81 | 15.86 | -2.46 | 0.000 | - && - && PF00651:BTB/POZ domain |
| A08562 | Contig6 | 103791 | 106373 | + | 1941 |  | 45.06 | 28.14 | 60.52 | 17.10 | 28.60 | 11.26 | 1.23 | 0.000 | - && A2QEQ6.1 RecName: Full=Beta-glucuronidase; Short=GlcAase; AltName: Full=Beta-D-glucuronoside glucuronosohydrolase; Flags: Precursor && PF16862:Glycosyl hydrolase family 79 C-terminal beta domain |
| A08565 | Contig6 | 1342319 | 1345150 | + | 1467 |  | 8.67 | 9.72 | 5.54 | 3.57 | 1.85 | 3.65 | 1.40 | 0.000 | - && - && PF13520:Amino acid permease |
| A08566 | Contig6 | 1345368 | 1347185 | - | 1368 |  | 21.91 | 36.62 | 7.81 | 3.55 | 3.08 | 6.67 | 2.32 | 0.000 | - && - && PF07690:Major Facilitator Superfamily |
| A08574 | Contig6 | 1377754 | 1380010 | + | 1773 |  | 21.41 | 21.69 | 16.23 | 10.76 | 6.44 | 7.28 | 1.28 | 0.000 | - && Q6PFP6.1 RecName: Full=NADPH-dependent diflavin oxidoreductase 1; AltName: Full=NADPH-dependent FMN and FAD-containing oxidoreductase && PF00667:FAD binding domain\|PF00258:Flavodoxin\|PF00175:Oxidoreductase NAD-binding domain |
| A08584 | Contig6 | 109638 | 112533 | - | 2157 |  | 34.42 | 27.51 | 43.32 | 6.72 | 11.59 | 5.66 | 2.14 | 0.000 | - && A2QEQ6.1 RecName: Full=Beta-glucuronidase; Short=GlcAase; AltName: Full=Beta-D-glucuronoside glucuronosohydrolase; Flags: Precursor && PF16862:Glycosyl hydrolase family 79 C-terminal beta domain |
| A08590 | Contig6 | 1410965 | 1412142 | + | 1119 |  | 246.64 | 158.70 | 238.78 | 75.53 | 36.64 | 16.23 | 2.33 | 0.000 | - && - && - |
| A08591 | Contig6 | 1413701 | 1414536 | + | 780 |  | 81.28 | 110.94 | 55.28 | 5.99 | 3.72 | 6.53 | 3.93 | 0.000 | - && - && - |
| A08598 | Contig6 | 1436522 | 1440869 | - | 3894 |  | 28.87 | 27.64 | 27.66 | 58.75 | 49.74 | 65.33 | -1.05 | 0.000 | - && P13433.2 RecName: Full=DNA-directed RNA polymerase, mitochondrial; Flags: Precursor && PF00940:DNA-dependent RNA polymerase\|PF14700:DNA-directed RNA polymerase N-terminal |
| A08606 | Contig6 | 1480996 | 1481624 | + | 513 |  | 5.96 | 13.50 | 4.63 | 50.67 | 41.97 | 24.12 | -2.28 | 0.000 | - && - && - |
| A08608 | Contig6 | 1482168 | 1482859 | + | 567 |  | 116.34 | 63.38 | 92.64 | 25.89 | 42.60 | 12.69 | 1.75 | 0.000 | - && - && - |
| A08618 | Contig6 | 120588 | 122390 | - | 1155 |  | 8.88 | 7.13 | 9.02 | 5.42 | 3.65 | 2.74 | 1.08 | 0.001 | - && - && - |
| A08627 | Contig6 | 1534247 | 1537751 | - | 3174 |  | 0.93 | 0.22 | 0.09 | 0.06 | 0.06 | 0.00 | 3.39 | 0.003 | - && - && - |
| A08648 | Contig6 | 1623375 | 1624099 | + | 618 |  | 574.55 | 590.47 | 402.65 | 140.85 | 137.08 | 353.73 | 1.31 | 0.000 | - && - && - |
| A08657 | Contig6 | 1664199 | 1668412 | + | 3705 |  | 0.24 | 0.41 | 0.54 | 0.08 | 0.13 | 0.19 | 1.60 | 0.023 | - && - && - |
| A08670 | Contig6 | 1714414 | 1723385 | + | 3726 |  | 11.91 | 12.72 | 10.10 | 6.95 | 5.70 | 4.40 | 1.03 | 0.000 | - && Q9V770.1 RecName: Full=Probable cytochrome P450 6a17; AltName: Full=CYPVIA17 && PF00067:Cytochrome P450 |
| A08671 | Contig6 | 1724237 | 1725209 | + | 852 |  | 176.95 | 129.73 | 185.38 | 48.61 | 40.87 | 42.23 | 1.90 | 0.000 | - && - && PF09792:Ubiquitin 3 binding protein But2 C-terminal domain |
| A08672 | Contig6 | 1726544 | 1727591 | - | 924 |  | 174.37 | 133.31 | 176.87 | 88.64 | 82.26 | 69.71 | 1.01 | 0.000 | - && - && PF09792:Ubiquitin 3 binding protein But2 C-terminal domain |
| A08673 | Contig6 | 145309 | 146179 | - | 573 |  | 613.82 | 675.21 | 583.81 | 1359.85 | 1536.97 | 1322.35 | -1.17 | 0.000 | - && - && PF10342:Ser-Thr-rich glycosyl-phosphatidyl-inositol-anchored membrane family |
| A08685 | Contig6 | 1762340 | 1764531 | - | 1500 |  | 532.78 | 377.79 | 466.68 | 104.28 | 132.37 | 102.39 | 2.02 | 0.000 | - && Q9C101.1 RecName: Full=Uncharacterized MFS-type transporter PB1E7.08c && PF00083:Sugar (and other) transporter |
| A08695 | Contig6 | 156803 | 157584 | + | 615 |  | 6.89 | 8.65 | 10.10 | 4.41 | 4.87 | 2.57 | 1.11 | 0.004 | - && - && - |
| A08697 | Contig6 | 1812836 | 1814903 | - | 1959 |  | 10.77 | 13.02 | 13.71 | 70.16 | 29.72 | 129.15 | -2.61 | 0.000 | - && - && - |
| A08702 | Contig6 | 1835359 | 1836544 | - | 1014 |  | 4.47 | 5.05 | 3.15 | 12.26 | 8.40 | 6.66 | -1.11 | 0.001 | - && - && - |
| A08709 | Contig6 | 1855152 | 1856153 | - | 774 |  | 14.91 | 22.18 | 7.32 | 3.50 | 6.05 | 2.72 | 1.86 | 0.000 | - && - && PF11807:Domain of unknown function (DUF3328) |
| A08710 | Contig6 | 1857118 | 1861965 | + | 1494 |  | 9.57 | 12.36 | 5.99 | 3.50 | 3.32 | 3.52 | 1.43 | 0.000 | - && - && PF11807:Domain of unknown function (DUF3328) |
| A08711 | Contig6 | 1863705 | 1864696 | + | 753 |  | 37.97 | 27.73 | 21.23 | 10.18 | 6.59 | 2.45 | 2.18 | 0.000 | - && - && PF11807:Domain of unknown function (DUF3328) |
| A08720 | Contig6 | 1894348 | 1895845 | - | 1224 |  | 5.64 | 2.21 | 5.67 | 1.91 | 1.99 | 2.29 | 1.13 | 0.008 | - && - && - |
| A08751 | Contig6 | 181175 | 182694 | + | 1038 |  | 32.20 | 46.52 | 33.71 | 81.43 | 85.58 | 59.69 | -1.01 | 0.000 | - && O94564.1 RecName: Full=Zinc-type alcohol dehydrogenase-like protein C1773.06c && PF00107:Zinc-binding dehydrogenase\|PF08240:Alcohol dehydrogenase GroES-like domain |
| A08778 | Contig6 | 2084018 | 2085861 | - | 1581 |  | 3.24 | 3.49 | 3.29 | 1.71 | 1.24 | 0.94 | 1.36 | 0.000 | - && - && PF01266:FAD dependent oxidoreductase |
| A08859 | Contig6 | 2327906 | 2335478 | + | 5004 |  | 2.07 | 2.95 | 1.04 | 0.37 | 0.54 | 0.37 | 2.24 | 0.000 | - && Q8I7P9.1 RecName: Full=Retrovirus-related Pol polyprotein from transposon opus; Includes: RecName: Full=Protease; Includes: RecName: Full=Reverse transcriptase; Includes: RecName: Full=Endonuclease && PF00385:Chromo (CHRromatin Organisation MOdifier) domain\|PF00665:Integrase core domain\|PF00078:Reverse transcriptase (RNA-dependent DNA polymerase) |
| A08860 | Contig6 | 2336003 | 2336606 | + | 486 |  | 11.97 | 7.44 | 6.01 | 14.43 | 18.49 | 18.60 | -1.02 | 0.003 | - && - && - |
| A08861 | Contig6 | 2339180 | 2341472 | + | 1572 |  | 1.76 | 1.47 | 1.57 | 8.86 | 11.61 | 3.46 | -2.32 | 0.000 | - && Q09653.3 RecName: Full=Putative cytochrome P450 CYP13A10 && PF00067:Cytochrome P450 |
| A08866 | Contig6 | 2357239 | 2358447 | - | 1002 |  | 3.25 | 2.91 | 3.19 | 5.13 | 6.45 | 7.53 | -1.03 | 0.002 | - && - && - |
| A08877 | Contig6 | 2395373 | 2397519 | - | 1869 |  | 10.39 | 10.69 | 8.26 | 20.76 | 19.78 | 19.67 | -1.04 | 0.000 | - && Q04800.1 RecName: Full=Ferric reductase transmembrane component 1; AltName: Full=Ferric-chelate reductase 1 && PF08030:Ferric reductase NAD binding domain\|PF01794:Ferric reductase like transmembrane component\|PF08022:FAD-binding domain |
| A08879 | Contig6 | 2399457 | 2403091 | + | 2901 |  | 1.26 | 2.80 | 1.76 | 13.86 | 21.36 | 12.49 | -3.03 | 0.000 | - && P29064.1 RecName: Full=Alpha-glucosidase; AltName: Full=Maltase; Contains: RecName: Full=Alpha-glucosidase subunit 1; Contains: RecName: Full=Alpha-glucosidase subunit 2; Flags: Precursor && PF01055:Glycosyl hydrolases family 31\|PF16863:N-terminal barrel of NtMGAM and CtMGAM, maltase-glucoamylase |
| A08888 | Contig6 | 2451400 | 2454193 | - | 1839 |  | 12.22 | 5.95 | 23.74 | 120.14 | 118.65 | 111.95 | -3.06 | 0.000 | - && J9VQZ4.2 RecName: Full=Laccase-2; AltName: Full=Diphenol oxidase 2; Flags: Precursor && PF00394:Multicopper oxidase\|PF07732:Multicopper oxidase\|PF07731:Multicopper oxidase |
| A08889 | Contig6 | 2455671 | 2457617 | + | 1500 |  | 14.72 | 8.37 | 26.37 | 111.26 | 109.40 | 91.09 | -2.66 | 0.000 | - && Q8X176.1 RecName: Full=Acid phosphatase; Flags: Precursor && PF04185:Phosphoesterase family |
| A08905 | Contig6 | 296542 | 298728 | - | 2073 |  | 455.91 | 381.02 | 257.35 | 124.12 | 68.95 | 89.45 | 1.95 | 0.000 | - && - && PF02179:BAG domain |
| A08925 | Contig6 | 408797 | 414479 | + | 1710 |  | 3.11 | 2.47 | 2.46 | 6.45 | 7.72 | 5.95 | -1.32 | 0.000 | - && Q566I3.2 RecName: Full=Hypoxia up-regulated protein 1; Flags: Precursor && PF00012:Hsp70 protein |
| A08926 | Contig6 | 438413 | 441164 | + | 2292 |  | 1.12 | 1.09 | 1.51 | 4.24 | 4.04 | 4.90 | -1.82 | 0.000 | - && Q566I3.2 RecName: Full=Hypoxia up-regulated protein 1; Flags: Precursor && PF00012:Hsp70 protein |
| A08929 | Contig6 | 487129 | 490804 | + | 2448 |  | 3.34 | 3.03 | 3.51 | 7.22 | 7.80 | 6.06 | -1.09 | 0.000 | - && Q566I3.2 RecName: Full=Hypoxia up-regulated protein 1; Flags: Precursor && PF00012:Hsp70 protein |
| A08934 | Contig6 | 536162 | 536611 | - | 399 |  | 0.00 | 0.00 | 0.46 | 2.11 | 0.70 | 3.08 | -3.69 | 0.011 | - && - && - |
| A08978 | Contig7 | 481060 | 485306 | - | 1878 |  | 3.94 | 4.38 | 4.86 | 6.77 | 21.83 | 7.01 | -1.43 | 0.000 | - && - && PF01161:Phosphatidylethanolamine-binding protein\|PF03732:Retrotransposon gag protein |
| A08979 | Contig7 | 486473 | 488091 | + | 1212 |  | 21.32 | 21.04 | 13.42 | 4.86 | 7.03 | 5.21 | 1.71 | 0.000 | - && - && PF06441:Epoxide hydrolase N terminus |
| A08981 | Contig7 | 491692 | 492808 | + | 816 |  | 0.24 | 0.00 | 0.11 | 0.80 | 2.29 | 1.18 | -3.60 | 0.002 | - && Q06816.2 RecName: Full=Putative epoxide hydrolase; AltName: Full=Epoxide hydratase; Flags: Precursor && PF06441:Epoxide hydrolase N terminus |
| A08984 | Contig7 | 516945 | 518078 | - | 1134 |  | 70.08 | 57.81 | 75.08 | 112.95 | 207.69 | 111.36 | -1.09 | 0.000 | - && - && - |
| A08986 | Contig7 | 522316 | 525614 | - | 2403 |  | 29.54 | 23.94 | 22.05 | 4.79 | 5.73 | 5.00 | 2.28 | 0.000 | - && Q06816.2 RecName: Full=Putative epoxide hydrolase; AltName: Full=Epoxide hydratase; Flags: Precursor && PF06441:Epoxide hydrolase N terminus |
| A08991 | Contig7 | 537280 | 544639 | - | 1617 |  | 67.14 | 74.75 | 38.13 | 3.53 | 4.75 | 2.39 | 4.08 | 0.000 | - && Q5N4X9.1 RecName: Full=2-phytyl-1,4-naphtoquinone methyltransferase; AltName: Full=Demethylphylloquinone methyltransferase >Q31P90.1 RecName: Full=2-phytyl-1,4-naphtoquinone methyltransferase; AltName: Full=Demethylphylloquinone methyltransferase && PF06330:Trichodiene synthase (TRI5)\|PF13847:Methyltransferase domain |
| A08992 | Contig7 | 545117 | 551033 | - | 3117 |  | 26.76 | 28.76 | 37.95 | 131.98 | 77.33 | 48.90 | -1.47 | 0.000 | - && O08394.1 RecName: Full=Bifunctional cytochrome P450/NADPH--P450 reductase 1; AltName: Full=CYP102A2; AltName: Full=Fatty acid hydroxylase CypD; AltName: Full=Flavocytochrome P450 102A2; Includes: RecName: Full=Cytochrome P450 102A2; Includes: RecName: Full=NADPH--cytochrome P450 reductase && PF00067:Cytochrome P450\|PF06330:Trichodiene synthase (TRI5) |
| A08993 | Contig7 | 560081 | 561186 | + | 945 |  | 45.08 | 35.69 | 26.58 | 3.46 | 5.55 | 5.29 | 2.91 | 0.000 | - && - && PF06330:Trichodiene synthase (TRI5) |
| A08994 | Contig7 | 561367 | 563625 | - | 1674 |  | 180.42 | 125.46 | 163.71 | 38.82 | 62.46 | 14.94 | 2.01 | 0.000 | - && Q59XM0.1 RecName: Full=MFS antiporter QDR3 && PF07690:Major Facilitator Superfamily |
| A08995 | Contig7 | 564593 | 566125 | - | 1221 |  | 25.28 | 50.48 | 16.68 | 6.59 | 8.82 | 7.55 | 2.01 | 0.000 | - && Q06816.2 RecName: Full=Putative epoxide hydrolase; AltName: Full=Epoxide hydratase; Flags: Precursor && PF06441:Epoxide hydrolase N terminus |
| A09002 | Contig7 | 583002 | 587021 | + | 1584 |  | 24.84 | 43.10 | 10.55 | 5.43 | 5.44 | 5.76 | 2.24 | 0.000 | - && - && PF06330:Trichodiene synthase (TRI5) |
| A09005 | Contig7 | 598820 | 600875 | - | 1857 |  | 24.85 | 25.41 | 21.64 | 53.47 | 46.12 | 68.29 | -1.22 | 0.000 | - && Q9UT32.1 RecName: Full=Putative ribosome biogenesis protein C8F11.04; AltName: Full=U3 snoRNP-associated protein C8F11.04 && PF00687:Ribosomal protein L1p/L10e family |
| A09012 | Contig7 | 628409 | 630637 | + | 1641 |  | 102.99 | 83.57 | 100.31 | 23.07 | 25.44 | 15.56 | 2.16 | 0.000 | - && Q59XM0.1 RecName: Full=MFS antiporter QDR3 && PF07690:Major Facilitator Superfamily |
| A09016 | Contig7 | 639328 | 641111 | - | 1416 |  | 6.62 | 9.85 | 8.45 | 12.55 | 12.49 | 24.98 | -1.01 | 0.001 | - && - && - |
| A09031 | Contig7 | 686924 | 687569 | + | 585 |  | 27.81 | 25.74 | 28.89 | 14.70 | 12.48 | 11.85 | 1.08 | 0.000 | - && - && - |
| A09034 | Contig7 | 690661 | 694351 | - | 3432 |  | 0.32 | 0.41 | 0.40 | 1.06 | 0.68 | 0.69 | -1.11 | 0.022 | - && - && - |
| A09045 | Contig7 | 736677 | 737983 | - | 1077 |  | 45.50 | 23.96 | 49.79 | 9.81 | 6.78 | 9.53 | 2.19 | 0.000 | - && - && - |
| A09048 | Contig7 | 742604 | 744932 | + | 1680 |  | 3.46 | 1.67 | 4.19 | 7.23 | 7.75 | 6.16 | -1.18 | 0.001 | - && Q9SYK0.1 RecName: Full=Beta-hexosaminidase 2; AltName: Full=Beta-GlcNAcase 2; AltName: Full=Beta-N-acetylhexosaminidase 2; AltName: Full=Beta-hexosaminidase 3; Short=AtHEX3; AltName: Full=N-acetyl-beta-glucosaminidase 2; Flags: Precursor && PF00728:Glycosyl hydrolase family 20, catalytic domain\|PF14845:beta-acetyl hexosaminidase like |
| A09054 | Contig7 | 5684 | 12445 | + | 3852 |  | 6.66 | 7.01 | 7.02 | 20.49 | 27.00 | 21.21 | -1.73 | 0.000 | - && Q4V7N3.1 RecName: Full=Serine/threonine/tyrosine-interacting protein A && PF00782:Dual specificity phosphatase, catalytic domain |
| A09058 | Contig7 | 769839 | 770996 | - | 903 |  | 2306.52 | 2169.07 | 2067.56 | 1082.40 | 945.23 | 983.81 | 1.12 | 0.000 | - && - && - |
| A09070 | Contig7 | 809052 | 809541 | + | 432 |  | 23.74 | 22.77 | 15.86 | 8.87 | 14.30 | 7.11 | 1.04 | 0.003 | - && - && - |
| A09071 | Contig7 | 810442 | 811359 | + | 861 |  | 7.10 | 5.71 | 7.43 | 70.04 | 100.03 | 59.12 | -3.50 | 0.000 | - && - && - |
| A09077 | Contig7 | 75745 | 77097 | + | 1086 |  | 369.08 | 361.90 | 333.86 | 155.57 | 164.98 | 166.71 | 1.13 | 0.000 | - && O14230.1 RecName: Full=Farnesyl pyrophosphate synthase; Short=FPP synthase; Short=FPS; AltName: Full=(2E,6E)-farnesyl diphosphate synthase; AltName: Full=Dimethylallyltranstransferase; AltName: Full=Farnesyl diphosphate synthase; AltName: Full=Geranyltranstransferase && PF00348:Polyprenyl synthetase |
| A09079 | Contig7 | 837434 | 841941 | - | 873 |  | 21.23 | 21.50 | 19.67 | 6.64 | 12.87 | 7.34 | 1.22 | 0.000 | - && - && - |
| A09082 | Contig7 | 855474 | 857313 | + | 1227 |  | 56.73 | 42.30 | 46.83 | 23.01 | 12.89 | 10.94 | 1.64 | 0.000 | - && - && - |
| A09084 | Contig7 | 861859 | 862139 | + | 219 |  | 483.11 | 542.74 | 270.71 | 54.22 | 19.66 | 66.92 | 3.20 | 0.000 | - && - && - |
| A09086 | Contig7 | 865130 | 866653 | + | 999 |  | 15.40 | 14.67 | 17.10 | 6.18 | 6.37 | 2.72 | 1.63 | 0.000 | - && Q76NT9.1 RecName: Full=1-aminocyclopropane-1-carboxylate oxidase; Short=ACC oxidase; Short=Ddaco; AltName: Full=Ethylene-forming enzyme; Short=EFE >A6BM06.1 RecName: Full=1-aminocyclopropane-1-carboxylate oxidase; Short=ACC oxidase; Short=Dmaco; AltName: Full=Ethylene-forming enzyme; Short=EFE && PF14226:non-haem dioxygenase in morphine synthesis N-terminal\|PF03171:2OG-Fe(II) oxygenase superfamily |
| A09087 | Contig7 | 866801 | 868957 | - | 1704 |  | 79.74 | 60.80 | 98.53 | 30.84 | 40.93 | 19.42 | 1.39 | 0.000 | - && O48786.1 RecName: Full=Cytochrome P450 734A1; AltName: Full=Protein PHYB ACTIVATION-TAGGED SUPPRESSOR 1 && PF00067:Cytochrome P450 |
| A09090 | Contig7 | 874066 | 876695 | + | 1569 |  | 13.70 | 12.22 | 15.55 | 95.82 | 101.78 | 90.05 | -2.79 | 0.000 | - && Q00017.1 RecName: Full=Rhamnogalacturonan acetylesterase; Short=RGAE; Flags: Precursor && PF00106:short chain dehydrogenase\|PF13472:GDSL-like Lipase/Acylhydrolase family |
| A09092 | Contig7 | 882334 | 885033 | + | 1911 |  | 4.90 | 2.99 | 8.89 | 150.40 | 333.93 | 83.44 | -5.08 | 0.000 | - && Q8NK92.2 RecName: Full=Aorsin; Flags: Precursor && PF09286:Pro-kumamolisin, activation domain\|PF00082:Subtilase family |
| A09093 | Contig7 | 885194 | 886365 | - | 942 |  | 2.30 | 4.58 | 2.33 | 18.76 | 16.60 | 27.48 | -2.77 | 0.000 | - && - && - |
| A09094 | Contig7 | 886733 | 888489 | - | 1536 |  | 23.82 | 32.74 | 26.41 | 11.44 | 14.08 | 12.57 | 1.12 | 0.000 | - && P35669.2 RecName: Full=Glutathione synthetase large chain; Short=GSH synthetase large chain; Short=GSH-S; AltName: Full=Glutathione synthase large chain; AltName: Full=Phytochelatin synthetase && PF03917:Eukaryotic glutathione synthase, ATP binding domain\|PF03199:Eukaryotic glutathione synthase |
| A09099 | Contig7 | 81397 | 87239 | - | 2661 |  | 80.00 | 57.83 | 93.34 | 320.72 | 410.46 | 271.88 | -2.12 | 0.000 | - && - && - |
| A09110 | Contig7 | 88395 | 90814 | + | 2085 |  | 29.46 | 34.86 | 39.30 | 219.91 | 122.43 | 109.56 | -2.12 | 0.000 | - && - && - |
| A09113 | Contig7 | 939619 | 940765 | - | 1086 |  | 67.73 | 63.41 | 57.54 | 28.58 | 25.69 | 35.23 | 1.08 | 0.000 | - && O60122.1 RecName: Full=Anthranilate phosphoribosyltransferase && PF02885:Glycosyl transferase family, helical bundle domain\|PF00591:Glycosyl transferase family, a/b domain |
| A09117 | Contig7 | 954167 | 955213 | + | 1047 |  | 165.00 | 157.44 | 159.93 | 56.35 | 73.94 | 45.93 | 1.45 | 0.000 | - && P36551.3 RecName: Full=Oxygen-dependent coproporphyrinogen-III oxidase, mitochondrial; Short=COX; Short=Coprogen oxidase; Short=Coproporphyrinogenase; Flags: Precursor && PF01218:Coproporphyrinogen III oxidase |
| A09118 | Contig7 | 959084 | 960741 | + | 1359 |  | 94.10 | 110.07 | 86.17 | 30.75 | 14.88 | 22.47 | 2.09 | 0.000 | - && Q08268.1 RecName: Full=Probable transporter MCH4 && PF07690:Major Facilitator Superfamily |
| A09136 | Contig7 | 1005098 | 1006615 | - | 1341 |  | 4.41 | 4.12 | 4.16 | 2.02 | 2.44 | 1.57 | 1.07 | 0.002 | - && Q86AW9.1 RecName: Full=Guanine deaminase; Short=Guanase; Short=Guanine aminase; AltName: Full=Guanine aminohydrolase; Short=GAH && PF01979:Amidohydrolase family |
| A09141 | Contig7 | 1018159 | 1019595 | - | 1164 |  | 21.52 | 21.91 | 18.05 | 6.51 | 8.61 | 6.56 | 1.50 | 0.000 | - && Q07589.2 RecName: Full=Uncharacterized protein YDL144C && PF08546:Ketopantoate reductase PanE/ApbA C terminal\|PF02558:Ketopantoate reductase PanE/ApbA |
| A09144 | Contig7 | 1022522 | 1027691 | + | 3477 |  | 31.76 | 30.72 | 33.81 | 52.60 | 68.49 | 78.55 | -1.05 | 0.000 | - && - && PF00494:Squalene/phytoene synthase |
| A09153 | Contig7 | 1057863 | 1059758 | - | 1080 |  | 30.86 | 25.10 | 33.07 | 320.74 | 419.69 | 891.64 | -4.20 | 0.000 | - && Q8NKE2.2 RecName: Full=Alternative oxidase, mitochondrial; Flags: Precursor && PF01786:Alternative oxidase |
| A09157 | Contig7 | 1068873 | 1072584 | + | 3324 |  | 11.63 | 13.92 | 18.14 | 47.54 | 43.76 | 50.48 | -1.70 | 0.000 | - && O13992.1 RecName: Full=Meiotically up-regulated gene 56 protein && PF15404:Pleckstrin homology domain |
| A09159 | Contig7 | 1080233 | 1081487 | + | 957 |  | 7.01 | 11.22 | 6.20 | 130.82 | 62.70 | 71.99 | -3.44 | 0.000 | - && P0CT91.1 RecName: Full=Lipase A; AltName: Full=Acetylxylan esterase; Flags: Precursor && PF01764:Lipase (class 3) |
| A09165 | Contig7 | 17768 | 19198 | - | 795 |  | 171.78 | 175.02 | 104.22 | 56.10 | 39.80 | 39.08 | 1.74 | 0.000 | - && Q9HDX3.1 RecName: Full=AB hydrolase superfamily protein B1A11.02 && PF07859:alpha/beta hydrolase fold |
| A09167 | Contig7 | 1094387 | 1095982 | - | 1386 |  | 14.73 | 16.08 | 17.47 | 73.06 | 76.46 | 60.53 | -2.12 | 0.000 | - && - && - |
| A09176 | Contig7 | 1127586 | 1128700 | - | 801 |  | 98.23 | 77.83 | 48.13 | 28.48 | 22.79 | 30.13 | 1.46 | 0.000 | - && - && - |
| A09177 | Contig7 | 106704 | 108466 | + | 1371 |  | 4.67 | 5.13 | 5.66 | 2.59 | 3.07 | 1.92 | 1.03 | 0.002 | - && Q53552.1 RecName: Full=Salicylate hydroxylase; AltName: Full=Salicylate 1-monooxygenase && PF01494:FAD binding domain |
| A09180 | Contig7 | 1134398 | 1143053 | + | 4905 |  | 59.81 | 48.46 | 31.06 | 15.86 | 15.12 | 16.59 | 1.55 | 0.000 | - && Q99078.2 RecName: Full=Dual specificity protein kinase FUZ7 && PF00069:Protein kinase domain |
| A09181 | Contig7 | 1143509 | 1146120 | - | 1812 |  | 37.87 | 33.41 | 37.05 | 108.72 | 76.92 | 59.47 | -1.18 | 0.000 | - && P39992.1 RecName: Full=Uncharacterized protein YEL023C && PF09994:Uncharacterized alpha/beta hydrolase domain (DUF2235) |
| A09214 | Contig7 | 1279671 | 1280735 | + | 825 |  | 40.16 | 29.93 | 44.29 | 128.51 | 176.10 | 116.80 | -1.88 | 0.000 | - && P40354.1 RecName: Full=Protein N-terminal amidase; Short=NT-amidase && PF00795:Carbon-nitrogen hydrolase |
| A09219 | Contig7 | 1296881 | 1299229 | - | 873 |  | 14.57 | 18.74 | 13.50 | 44.77 | 27.02 | 23.32 | -1.02 | 0.000 | - && Q5A416.1 RecName: Full=tRNA (adenine(58)-N(1))-methyltransferase catalytic subunit TRM61; AltName: Full=tRNA(m1A58)-methyltransferase subunit TRM61; Short=tRNA(m1A58)MTase subunit TRM61 && PF08704:tRNA methyltransferase complex GCD14 subunit |
| A09222 | Contig7 | 1302787 | 1306506 | + | 3456 |  | 8.62 | 10.69 | 8.96 | 20.94 | 17.50 | 22.19 | -1.10 | 0.000 | - && O94653.2 RecName: Full=Ribosome biogenesis protein bms1 && PF08142:AARP2CN (NUC121) domain\|PF04950:40S ribosome biogenesis protein Tsr1 and BMS1 C-terminal |
| A09226 | Contig7 | 1326751 | 1328579 | + | 1491 |  | 189.73 | 176.94 | 152.56 | 56.31 | 39.11 | 90.41 | 1.48 | 0.000 | - && - && PF00651:BTB/POZ domain |
| A09235 | Contig7 | 1356738 | 1358581 | + | 1182 |  | 104.61 | 89.69 | 74.50 | 24.92 | 33.98 | 36.01 | 1.50 | 0.000 | - && Q9UTK3.1 RecName: Full=Probable nicotinate phosphoribosyltransferase; Short=NAPRTase && PF04095:Nicotinate phosphoribosyltransferase (NAPRTase) family |
| A09249 | Contig7 | 1397591 | 1400441 | - | 1863 |  | 1.96 | 1.08 | 1.86 | 1.10 | 0.45 | 0.71 | 1.11 | 0.025 | - && P32948.1 RecName: Full=Lipase 4; Flags: Precursor && PF00135:Carboxylesterase family |
| A09251 | Contig7 | 1407156 | 1408241 | - | 1029 |  | 661.28 | 554.23 | 762.31 | 296.02 | 247.17 | 263.53 | 1.29 | 0.000 | - && P14791.2 RecName: Full=Heme oxygenase 1; Short=HO-1 && PF01126:Heme oxygenase |
| A09252 | Contig7 | 1409155 | 1410999 | - | 1434 |  | 45.66 | 27.51 | 65.80 | 6.45 | 7.38 | 4.90 | 2.89 | 0.000 | - && A1YPR2.1 RecName: Full=2-epi-5-epi-valiolone synthase; Short=EEVS && PF01761:3-dehydroquinate synthase |
| A09253 | Contig7 | 1411960 | 1412984 | + | 855 |  | 0.92 | 1.88 | 0.43 | 6.89 | 22.66 | 0.62 | -3.22 | 0.013 | - && Q00719.1 RecName: Full=O-methyltransferase MdmC && PF01596:O-methyltransferase |
| A09271 | Contig7 | 1502999 | 1504569 | - | 1212 |  | 5.04 | 5.96 | 6.41 | 0.85 | 2.93 | 0.65 | 1.97 | 0.000 | - && - && - |
| A09273 | Contig7 | 1508325 | 1508821 | - | 303 |  | 67.36 | 163.34 | 126.92 | 299.00 | 368.57 | 278.63 | -1.40 | 0.000 | - && - && - |
| A09275 | Contig7 | 1512883 | 1514233 | - | 1284 |  | 2.61 | 3.28 | 2.13 | 0.80 | 1.24 | 1.85 | 1.05 | 0.022 | - && - && - |
| A09278 | Contig7 | 1514753 | 1516916 | - | 1506 |  | 54.15 | 43.33 | 55.38 | 24.46 | 19.02 | 15.44 | 1.38 | 0.000 | - && A6RUD7.1 RecName: Full=Carboxypeptidase Y homolog A; Flags: Precursor && PF00450:Serine carboxypeptidase |
| A09280 | Contig7 | 1519764 | 1521912 | - | 1491 |  | 17.92 | 20.47 | 18.99 | 6.21 | 11.49 | 9.59 | 1.07 | 0.000 | - && P30574.2 RecName: Full=Carboxypeptidase Y; AltName: Full=Carboxypeptidase YSCY; Flags: Precursor && PF00450:Serine carboxypeptidase |
| A09293 | Contig7 | 1563012 | 1564853 | - | 1731 |  | 10.42 | 9.69 | 8.18 | 4.00 | 2.92 | 3.65 | 1.42 | 0.000 | - && - && - |
| A09307 | Contig7 | 1605128 | 1605830 | - | 594 |  | 12.62 | 31.43 | 12.46 | 8.03 | 8.04 | 3.40 | 1.54 | 0.000 | - && - && - |
| A09311 | Contig7 | 1614250 | 1615044 | + | 738 |  | 2.40 | 4.90 | 3.09 | 0.76 | 0.76 | 0.71 | 2.22 | 0.000 | - && - && PF13417:Glutathione S-transferase, N-terminal domain |
| A09317 | Contig7 | 1628635 | 1630243 | + | 1044 |  | 68.10 | 74.91 | 67.02 | 250.58 | 407.80 | 233.77 | -2.09 | 0.000 | - && P43549.1 RecName: Full=Uncharacterized membrane protein YFL054C && PF00230:Major intrinsic protein |
| A09321 | Contig7 | 146794 | 152132 | - | 1398 |  | 247.35 | 220.74 | 240.85 | 147.47 | 87.85 | 92.91 | 1.11 | 0.000 | - && - && - |
| A09339 | Contig7 | 1679713 | 1682002 | - | 1863 |  | 0.53 | 4.90 | 0.39 | 0.45 | 0.35 | 0.61 | 2.04 | 0.011 | - && Q4P647.1 RecName: Full=FACT complex subunit POB3; AltName: Full=Facilitates chromatin transcription complex subunit POB3 && PF03531:Structure-specific recognition protein (SSRP1)\|PF08512:Histone chaperone Rttp106-like |
| A09343 | Contig7 | 157137 | 158864 | + | 1263 |  | 125.38 | 143.47 | 110.95 | 63.74 | 52.33 | 65.04 | 1.07 | 0.000 | - && Q93ZE9.1 RecName: Full=Phosphatidylinositol/phosphatidylcholine transfer protein SFH3; AltName: Full=Protein SEC FOURTEEN HOMOLOGS 3; Short=AtSFH3 && PF03765:CRAL/TRIO, N-terminal domain\|PF00650:CRAL/TRIO domain |
| A09345 | Contig7 | 1704342 | 1706032 | + | 1563 |  | 0.76 | 0.19 | 0.41 | 1.73 | 2.34 | 0.84 | -1.85 | 0.004 | - && - && - |
| A09354 | Contig7 | 159393 | 164054 | - | 1848 |  | 1.39 | 2.34 | 1.19 | 9.01 | 7.50 | 11.49 | -2.51 | 0.000 | - && Q4WAZ6.1 RecName: Full=Multifunctional cytochrome P450 monooxygenase af510; AltName: Full=Fumagillin bioynthesis cluster P450 monooxygenase; Short=Fma-P450 && PF00067:Cytochrome P450 |
| A09389 | Contig7 | 1868386 | 1868754 | + | 219 |  | 2806.81 | 1016.26 | 4260.90 | 24.33 | 27.78 | 32.46 | 6.58 | 0.000 | - && - && - |
| A09421 | Contig7 | 189088 | 190882 | - | 1521 |  | 5.64 | 11.68 | 4.62 | 35.96 | 34.28 | 22.39 | -2.08 | 0.000 | - && Q6UEF1.1 RecName: Full=Oxidoreductase AflY; AltName: Full=Aflatoxin biosynthesis protein Y && PF14027:Protein of unknown function (DUF4243) |
| A09429 | Contig7 | 2022635 | 2023428 | + | 681 |  | 133.06 | 122.94 | 111.87 | 286.12 | 226.67 | 266.50 | -1.08 | 0.000 | - && Q09791.1 RecName: Full=Uncharacterized protein C13G6.15c && PF04847:Calcipressin |
| A09431 | Contig7 | 2028422 | 2030420 | - | 1419 |  | 5.70 | 6.08 | 4.31 | 11.60 | 15.77 | 8.60 | -1.16 | 0.000 | - && P31049.1 RecName: Full=Probable fatty acid methyltransferase; AltName: Full=ORF3; AltName: Full=S-adenosylmethionine-dependent methyltransferase; Short=AdoMet-MT; Short=SAM-MT && PF02353:Mycolic acid cyclopropane synthetase |
| A09439 | Contig7 | 2064676 | 2066728 | + | 1377 |  | 4.94 | 4.74 | 5.97 | 20.03 | 24.34 | 14.21 | -1.90 | 0.000 | - && Q0CJ57.1 RecName: Full=Cytochrome P450 monooxygenase atE; AltName: Full=Terreic acid biosynthesis cluster protein E && PF00067:Cytochrome P450 |
| A09442 | Contig7 | 2074944 | 2076803 | - | 945 |  | 8.14 | 7.65 | 7.73 | 20.58 | 17.63 | 17.37 | -1.24 | 0.000 | - && - && - |
| A09461 | Contig7 | 2146913 | 2148690 | + | 1332 |  | 6.81 | 8.44 | 9.67 | 36.43 | 34.51 | 38.94 | -2.14 | 0.000 | - && Q2UP32.1 RecName: Full=Putative galacturan 1,4-alpha-galacturonidase A; AltName: Full=Exopolygalacturonase A; AltName: Full=Exorhamnogalacturonase A; AltName: Full=Poly(1,4-alpha-D-galacturonide)galacturonohydrolase A; Flags: Precursor >B8NJB0.2 RecName: Full=Putative galacturan 1,4-alpha-galacturonidase A; AltName: Full=Exopolygalacturonase A; AltName: Full=Exorhamnogalacturonase A; AltName: Full=Poly(1,4-alpha-D-galacturonide)galacturonohydrolase rgxA; Flags: Precursor && PF00295:Glycosyl hydrolases family 28 |
| A09462 | Contig7 | 2149908 | 2151674 | + | 1422 |  | 0.07 | 0.21 | 0.32 | 2.83 | 3.88 | 2.22 | -3.89 | 0.000 | - && Q2UP32.1 RecName: Full=Putative galacturan 1,4-alpha-galacturonidase A; AltName: Full=Exopolygalacturonase A; AltName: Full=Exorhamnogalacturonase A; AltName: Full=Poly(1,4-alpha-D-galacturonide)galacturonohydrolase A; Flags: Precursor >B8NJB0.2 RecName: Full=Putative galacturan 1,4-alpha-galacturonidase A; AltName: Full=Exopolygalacturonase A; AltName: Full=Exorhamnogalacturonase A; AltName: Full=Poly(1,4-alpha-D-galacturonide)galacturonohydrolase rgxA; Flags: Precursor && PF00295:Glycosyl hydrolases family 28 |
| A09466 | Contig7 | 2157475 | 2158463 | + | 864 |  | 18.72 | 22.08 | 9.52 | 10.28 | 5.96 | 8.23 | 1.04 | 0.003 | - && C3K630.1 RecName: Full=L-amino acid amidase && PF00561:alpha/beta hydrolase fold |
| A09486 | Contig7 | 2235254 | 2236740 | - | 1254 |  | 4.17 | 7.69 | 3.72 | 15.06 | 13.36 | 12.53 | -1.40 | 0.000 | - && - && - |
| A09502 | Contig7 | 2276103 | 2278811 | - | 2601 |  | 53.26 | 40.80 | 67.22 | 25.81 | 24.29 | 25.71 | 1.09 | 0.000 | - && P40566.1 RecName: Full=Uncharacterized glycosyl hydrolase YIR007W && PF00150:Cellulase (glycosyl hydrolase family 5) |
| A09523 | Contig7 | 264815 | 265333 | - | 519 |  | 38.00 | 31.92 | 105.61 | 96.74 | 129.86 | 128.34 | -1.02 | 0.007 | - && - && - |
| A09525 | Contig7 | 269102 | 269896 | + | 795 |  | 51.22 | 48.24 | 72.51 | 27.05 | 22.61 | 14.68 | 1.42 | 0.000 | - && - && - |
| A09549 | Contig8 | 1572657 | 1574757 | - | 1578 |  | 118.91 | 77.93 | 146.29 | 215.91 | 296.85 | 283.85 | -1.22 | 0.000 | - && P07921.1 RecName: Full=Lactose permease && PF00083:Sugar (and other) transporter |
| A09569 | Contig8 | 1687205 | 1691188 | - | 2646 |  | 4.99 | 4.51 | 4.56 | 28.73 | 12.91 | 28.46 | -2.32 | 0.000 | - && - && PF07690:Major Facilitator Superfamily |
| A09576 | Contig8 | 1729368 | 1730168 | - | 627 |  | 10.06 | 96.07 | 19.09 | 641.80 | 683.64 | 153.40 | -3.56 | 0.001 | - && - && - |
| A09581 | Contig8 | 1743773 | 1747065 | - | 3234 |  | 12.35 | 12.91 | 15.54 | 27.35 | 48.51 | 42.55 | -1.54 | 0.000 | - && P08092.2 RecName: Full=Negative regulator of sexual conjugation and meiosis && PF00069:Protein kinase domain |
| A09587 | Contig8 | 1782955 | 1784859 | + | 1275 |  | 1.78 | 3.54 | 1.22 | 9.24 | 17.11 | 8.88 | -2.43 | 0.000 | - && Q00359.1 RecName: Full=Exopolygalacturonase; Short=ExoPG; AltName: Full=Galacturan 1,4-alpha-galacturonidase; AltName: Full=Poly(1,4-alpha-D-galacturonide)galacturonohydrolase; Flags: Precursor && PF00295:Glycosyl hydrolases family 28 |
| A09606 | Contig8 | 1866007 | 1869899 | + | 1992 |  | 6.39 | 8.47 | 7.15 | 13.99 | 16.68 | 17.01 | -1.12 | 0.000 | - && - && - |
| A09644 | Contig8 | 30194 | 31615 | + | 1422 |  | 0.62 | 0.92 | 1.54 | 1.78 | 1.91 | 3.39 | -1.20 | 0.018 | - && - && - |
| A09645 | Contig8 | 35398 | 35864 | + | 342 |  | 17.30 | 9.10 | 9.08 | 6.01 | 1.09 | 4.36 | 1.63 | 0.003 | - && - && PF03330:Rare lipoprotein A (RlpA)-like double-psi beta-barrel |
| A09659 | Contig8 | 86116 | 87746 | - | 1524 |  | 63.86 | 41.63 | 73.61 | 25.95 | 32.80 | 13.53 | 1.31 | 0.000 | - && - && PF14441:OTT_1508-like deaminase |
| A09661 | Contig8 | 90321 | 94526 | - | 2970 |  | 14.08 | 26.13 | 15.16 | 2.90 | 1.61 | 1.98 | 3.09 | 0.000 | - && Q01117.2 RecName: Full=Alpha-amylase 1; AltName: Full=1,4-alpha-D-glucan glucanohydrolase 1; Flags: Precursor && PF09260:Domain of unknown function (DUF1966)\|PF00106:short chain dehydrogenase\|PF00128:Alpha amylase, catalytic domain |
| A09662 | Contig8 | 95043 | 96888 | - | 1470 |  | 3.56 | 4.51 | 1.93 | 1.65 | 1.85 | 1.37 | 1.04 | 0.013 | - && Q01117.2 RecName: Full=Alpha-amylase 1; AltName: Full=1,4-alpha-D-glucan glucanohydrolase 1; Flags: Precursor && PF00128:Alpha amylase, catalytic domain\|PF09260:Domain of unknown function (DUF1966) |
| A09664 | Contig8 | 102080 | 103794 | - | 1008 |  | 41.08 | 28.18 | 28.46 | 12.34 | 7.15 | 8.71 | 1.79 | 0.000 | - && - && - |
| A09665 | Contig8 | 104307 | 106598 | + | 1731 |  | 9.00 | 7.71 | 8.02 | 2.27 | 2.65 | 2.38 | 1.76 | 0.000 | - && Q0R4L2.1 RecName: Full=Pyranose dehydrogenase 3; Short=PDH 3; AltName: Full=Pyranose:quinone oxidoreductase 3; Flags: Precursor && PF05199:GMC oxidoreductase\|PF00732:GMC oxidoreductase |
| A09666 | Contig8 | 107570 | 108216 | - | 534 |  | 1.29 | 2.63 | 2.91 | 3.15 | 7.01 | 10.68 | -1.61 | 0.004 | - && Q9H9V4.2 RecName: Full=RING finger protein 122 && PF13639:Ring finger domain |
| A09681 | Contig8 | 176144 | 179445 | - | 2274 |  | 15.13 | 12.98 | 12.09 | 7.24 | 7.45 | 4.90 | 1.04 | 0.000 | - && Q2U4L7.2 RecName: Full=Glutaminase A; Flags: Precursor && PF08760:Domain of unknown function (DUF1793)\|PF16335:Domain of unknown function (DUF4965) |
| A09708 | Contig8 | 267838 | 269747 | + | 1221 |  | 152.06 | 170.36 | 121.28 | 52.30 | 39.64 | 48.66 | 1.66 | 0.000 | - && - && - |
| A09719 | Contig8 | 301646 | 302971 | + | 1032 |  | 861.53 | 927.42 | 483.66 | 318.27 | 231.40 | 247.46 | 1.51 | 0.000 | - && - && PF03036:Perilipin family |
| A09721 | Contig8 | 319501 | 321556 | + | 1686 |  | 0.88 | 1.07 | 0.98 | 0.22 | 0.56 | 0.10 | 1.73 | 0.013 | - && - && - |
| A09723 | Contig8 | 329255 | 329896 | + | 642 |  | 52.22 | 33.15 | 36.00 | 17.62 | 5.10 | 9.71 | 1.90 | 0.000 | - && - && - |
| A09731 | Contig8 | 356797 | 360274 | - | 2937 |  | 0.50 | 0.68 | 0.90 | 0.41 | 0.29 | 0.24 | 1.15 | 0.038 | - && Q9HDW7.1 RecName: Full=Calcium-transporting ATPase 2 && PF00122:E1-E2 ATPase\|PF00690:Cation transporter/ATPase, N-terminus |
| A09743 | Contig8 | 418447 | 420512 | - | 1167 |  | 76.72 | 73.89 | 85.64 | 34.45 | 36.18 | 29.18 | 1.24 | 0.000 | - && A7F075.1 RecName: Full=Metacaspase-1; Flags: Precursor && PF00656:Caspase domain |
| A09744 | Contig8 | 423216 | 423924 | + | 660 |  | 69.62 | 53.54 | 85.12 | 39.66 | 16.45 | 29.78 | 1.28 | 0.000 | - && - && - |
| A09747 | Contig8 | 436458 | 441609 | + | 2475 |  | 49.16 | 52.81 | 43.07 | 21.49 | 24.74 | 24.40 | 1.04 | 0.000 | - && P50276.1 RecName: Full=High-affinity methionine permease && PF13520:Amino acid permease |
| A09748 | Contig8 | 446286 | 447962 | - | 1524 |  | 354.94 | 334.76 | 358.57 | 182.94 | 157.61 | 151.39 | 1.09 | 0.000 | - && - && PF00505:HMG (high mobility group) box |
| A09752 | Contig8 | 463139 | 464150 | + | 897 |  | 3.30 | 2.91 | 3.46 | 13.13 | 7.31 | 7.14 | -1.51 | 0.000 | - && O07575.1 RecName: Full=Uncharacterized oxidoreductase YhdF && PF00106:short chain dehydrogenase |
| A09763 | Contig8 | 501157 | 510447 | + | 3342 |  | 192.99 | 182.81 | 142.49 | 44.31 | 55.35 | 51.89 | 1.77 | 0.000 | - && Q3ZFI7.1 RecName: Full=D-galacturonate reductase; AltName: Full=D-galacturonic acid reductase && PF00248:Aldo/keto reductase family\|PF01822:WSC domain\|PF09362:Domain of unknown function (DUF1996) |
| A09771 | Contig8 | 542870 | 544170 | + | 963 |  | 77.72 | 148.03 | 67.45 | 610.59 | 758.90 | 445.17 | -2.63 | 0.000 | - && Q54GH4.1 RecName: Full=Inositol oxygenase; AltName: Full=Myo-inositol oxygenase; Short=MI oxygenase && PF05153:Myo-inositol oxygenase |
| A09773 | Contig8 | 547489 | 548127 | - | 639 |  | 4.17 | 6.44 | 7.72 | 2.19 | 3.37 | 3.43 | 1.03 | 0.016 | - && - && - |
| A09776 | Contig8 | 560469 | 561877 | + | 957 |  | 23.90 | 42.38 | 18.61 | 6.35 | 3.91 | 5.50 | 2.43 | 0.000 | - && O94524.1 RecName: Full=Glutathione S-transferase omega-like 2; AltName: Full=Glutathione-dependent dehydroascorbate reductase && PF13410:Glutathione S-transferase, C-terminal domain\|PF13409:Glutathione S-transferase, N-terminal domain |
| A09779 | Contig8 | 574343 | 578550 | - | 1749 |  | 187.96 | 179.60 | 183.54 | 43.83 | 40.94 | 43.75 | 2.10 | 0.000 | - && - && - |
| A09783 | Contig8 | 585592 | 589013 | - | 1296 |  | 6.54 | 9.53 | 6.13 | 20.63 | 21.89 | 12.59 | -1.31 | 0.000 | - && Q9FZ42.1 RecName: Full=Glucose and ribitol dehydrogenase homolog 1 && PF00106:short chain dehydrogenase |
| A09785 | Contig8 | 592928 | 595198 | + | 1587 |  | 19.82 | 19.17 | 20.89 | 37.59 | 34.98 | 53.86 | -1.08 | 0.000 | - && P38355.1 RecName: Full=Uncharacterized transporter YBR287W && PF03547:Membrane transport protein |
| A09789 | Contig8 | 613213 | 614313 | - | 846 |  | 27.97 | 20.65 | 21.38 | 10.72 | 12.39 | 11.72 | 1.01 | 0.000 | - && - && PF13472:GDSL-like Lipase/Acylhydrolase family |
| A09791 | Contig8 | 615944 | 617450 | + | 1149 |  | 15.02 | 14.77 | 14.71 | 40.36 | 53.12 | 36.28 | -1.54 | 0.000 | - && C5CZC3.1 RecName: Full=D-galactonate dehydratase; Short=GalD && PF02746:Mandelate racemase / muconate lactonizing enzyme, N-terminal domain\|PF13378:Enolase C-terminal domain-like |
| A09792 | Contig8 | 617849 | 618868 | - | 954 |  | 526.40 | 347.47 | 496.68 | 184.35 | 136.98 | 103.03 | 1.69 | 0.000 | - && - && - |
| A09800 | Contig8 | 646788 | 648945 | - | 1632 |  | 28.16 | 25.84 | 23.34 | 127.98 | 186.13 | 127.12 | -2.51 | 0.000 | - && Q6TU48.1 RecName: Full=NADP-dependent malic enzyme; Short=NADP-ME; AltName: Full=SrfA-induced gene A protein && PF00390:Malic enzyme, N-terminal domain\|PF03949:Malic enzyme, NAD binding domain |
| A09805 | Contig8 | 662596 | 663096 | - | 501 |  | 2.95 | 3.21 | 3.10 | 0.37 | 1.31 | 0.88 | 1.86 | 0.008 | - && - && - |
| A09808 | Contig8 | 668872 | 671691 | + | 1992 |  | 44.10 | 38.96 | 59.71 | 368.21 | 344.32 | 376.76 | -2.93 | 0.000 | - && - && - |
| A09811 | Contig8 | 683407 | 684725 | + | 1146 |  | 2.06 | 1.84 | 1.43 | 37.20 | 48.03 | 29.48 | -4.43 | 0.000 | - && P32816.1 RecName: Full=Glycerol dehydrogenase; Short=GDH; Short=GLDH; Short=GlyDH && PF00465:Iron-containing alcohol dehydrogenase |
| A09820 | Contig8 | 713559 | 714225 | - | 609 |  | 210.32 | 226.33 | 160.65 | 29.63 | 13.83 | 35.16 | 2.93 | 0.000 | - && - && - |
| A09821 | Contig8 | 715680 | 716485 | - | 609 |  | 0.16 | 0.00 | 0.30 | 1.38 | 0.46 | 2.16 | -3.12 | 0.013 | - && - && - |
| A09823 | Contig8 | 718483 | 720006 | - | 1524 |  | 6.02 | 11.33 | 6.65 | 37.48 | 42.20 | 39.50 | -2.31 | 0.000 | - && - && PF07632:Protein of unknown function (DUF1593) |
| A09838 | Contig8 | 802532 | 804045 | + | 1170 |  | 13.65 | 12.61 | 10.46 | 6.47 | 3.76 | 7.95 | 1.01 | 0.001 | - && Q45FA5.1 RecName: Full=Serine/threonine-protein kinase SRPK; Short=PSRPK && PF00069:Protein kinase domain |
| A09842 | Contig8 | 812692 | 813549 | + | 789 |  | 327.05 | 322.41 | 255.52 | 128.10 | 149.02 | 165.06 | 1.03 | 0.000 | - && - && PF13417:Glutathione S-transferase, N-terminal domain |
| A09843 | Contig8 | 814181 | 817592 | - | 2259 |  | 11.79 | 12.31 | 8.69 | 5.42 | 3.32 | 3.22 | 1.45 | 0.000 | - && Q942X8.1 RecName: Full=Probable potassium transporter 2; AltName: Full=OsHAK2 && PF02705:K+ potassium transporter |
| A09846 | Contig8 | 824592 | 826977 | + | 1641 |  | 16.04 | 38.48 | 23.94 | 7.35 | 9.98 | 6.04 | 1.75 | 0.000 | - && Q3MID2.1 RecName: Full=Leukotriene-B(4) omega-hydroxylase 2; AltName: Full=CYPIVF3; AltName: Full=Cytochrome P450 4F3; AltName: Full=Cytochrome P450-LTB-omega; AltName: Full=Leukotriene-B(4) 20-monooxygenase 2 && PF00067:Cytochrome P450 |
| A09850 | Contig8 | 841013 | 842456 | - | 1053 |  | 0.94 | 1.05 | 0.43 | 1.33 | 3.20 | 1.75 | -1.38 | 0.023 | - && U6A629.1 RecName: Full=Alpha-L-arabinofuranosidase A; Short=ABF A; Short=Arabinosidase A; AltName: Full=Arabinoxylan-arabinofuranohydrolase of 70 kDa; Short=AF-70; Flags: Precursor && - |
| A09854 | Contig8 | 863179 | 863962 | - | 726 |  | 2.44 | 4.42 | 3.02 | 5.15 | 5.42 | 9.43 | -1.02 | 0.016 | - && - && - |
| A09856 | Contig8 | 869073 | 870465 | + | 1224 |  | 71.13 | 67.50 | 68.14 | 29.26 | 33.34 | 31.26 | 1.14 | 0.000 | - && O94671.1 RecName: Full=Probable homoserine dehydrogenase; Short=HDH && PF03447:Homoserine dehydrogenase, NAD binding domain\|PF00742:Homoserine dehydrogenase |
| A09857 | Contig8 | 871755 | 874256 | + | 1629 |  | 5.45 | 6.29 | 4.54 | 49.70 | 54.02 | 34.91 | -3.09 | 0.000 | - && Q5BF93.2 RecName: Full=Mannosyl-oligosaccharide alpha-1,2-mannosidase 1B; AltName: Full=Class I alpha-mannosidase 1B; AltName: Full=Man(9)-alpha-mannosidase 1B; Flags: Precursor && PF01532:Glycosyl hydrolase family 47 |
| A09860 | Contig8 | 880475 | 881274 | - | 477 |  | 2.89 | 2.74 | 2.87 | 6.66 | 6.28 | 8.83 | -1.36 | 0.002 | - && P0CT12.1 RecName: Full=Histone H2A >L7HZV6.1 RecName: Full=Histone H2A && - |
| A09868 | Contig8 | 899240 | 901554 | - | 1635 |  | 3.50 | 5.53 | 3.86 | 13.15 | 13.11 | 13.79 | -1.64 | 0.000 | - && Q09653.3 RecName: Full=Putative cytochrome P450 CYP13A10 && PF00067:Cytochrome P450 |
| A09880 | Contig8 | 964908 | 965559 | + | 531 |  | 167.12 | 93.77 | 121.28 | 9.16 | 18.51 | 22.81 | 2.92 | 0.000 | - && - && PF01040:UbiA prenyltransferase family |
| A09881 | Contig8 | 965753 | 966796 | - | 843 |  | 212.06 | 219.83 | 213.58 | 89.50 | 92.50 | 59.96 | 1.42 | 0.000 | - && - && PF14497:Glutathione S-transferase, C-terminal domain |
| A09891 | Contig8 | 995063 | 996021 | + | 705 |  | 5.04 | 8.69 | 14.90 | 287.78 | 337.13 | 1034.06 | -5.86 | 0.000 | - && - && - |
| A09905 | Contig8 | 1056109 | 1058207 | - | 1719 |  | 35.05 | 27.04 | 26.68 | 108.83 | 91.27 | 145.29 | -1.96 | 0.000 | - && - && - |
| A09911 | Contig8 | 1072993 | 1073721 | + | 729 |  | 1.89 | 3.03 | 2.88 | 14.88 | 18.88 | 9.87 | -2.48 | 0.000 | - && - && PF11937:Protein of unknown function (DUF3455) |
| A09920 | Contig8 | 1100017 | 1100580 | + | 564 |  | 3.85 | 5.52 | 3.40 | 1.49 | 1.33 | 2.80 | 1.18 | 0.027 | - && - && - |
| A09923 | Contig8 | 1118201 | 1120461 | - | 1608 |  | 6.56 | 4.93 | 5.79 | 28.20 | 31.20 | 16.37 | -2.13 | 0.000 | - && - && PF07690:Major Facilitator Superfamily |
| A09928 | Contig8 | 1136880 | 1140747 | - | 2871 |  | 42.62 | 52.62 | 34.17 | 8.50 | 8.74 | 7.31 | 2.40 | 0.000 | - && W7N2C1.1 RecName: Full=Non-canonical non-ribosomal peptide synthetase FUB8; AltName: Full=Fusaric acid biosynthesis protein 8 && PF00501:AMP-binding enzyme\|PF07993:Male sterility protein |
| A09930 | Contig8 | 1144892 | 1155897 | + | 7371 |  | 15.77 | 17.77 | 11.40 | 7.15 | 7.00 | 6.45 | 1.13 | 0.000 | - && P9WQE7.1 RecName: Full=Phthiocerol synthesis polyketide synthase type I PpsA; AltName: Full=Beta-ketoacyl-acyl-carrier-protein synthase I && PF08659:KR domain\|PF00109:Beta-ketoacyl synthase, N-terminal domain\|PF00698:Acyl transferase domain\|PF00975:Thioesterase domain\|PF14765:Polyketide synthase dehydratase\|PF02801:Beta-ketoacyl synthase, C-terminal domain |
| A09931 | Contig8 | 1157189 | 1159047 | + | 894 |  | 14.01 | 11.79 | 7.97 | 5.65 | 5.44 | 5.10 | 1.06 | 0.001 | - && - && - |
| A09935 | Contig8 | 1178461 | 1180456 | + | 1464 |  | 4.31 | 7.20 | 5.49 | 17.37 | 17.33 | 11.69 | -1.45 | 0.000 | - && G3Y416.1 RecName: Full=Cytochrome P450 monooxygenase yanC; AltName: Full=Yanuthone D synthesis protein C; Flags: Precursor && PF00067:Cytochrome P450 |
| A09936 | Contig8 | 1180615 | 1181590 | - | 813 |  | 0.36 | 0.00 | 0.11 | 1.96 | 0.35 | 0.76 | -2.68 | 0.026 | - && - && PF01753:MYND finger |
| A09938 | Contig8 | 1184491 | 1186925 | + | 1854 |  | 2.23 | 4.93 | 3.01 | 13.16 | 29.18 | 8.85 | -2.33 | 0.000 | - && Q4WAZ6.1 RecName: Full=Multifunctional cytochrome P450 monooxygenase af510; AltName: Full=Fumagillin bioynthesis cluster P450 monooxygenase; Short=Fma-P450 && PF00067:Cytochrome P450 |
| A09941 | Contig8 | 1196473 | 1200818 | + | 2481 |  | 104.96 | 87.24 | 92.27 | 27.77 | 30.98 | 17.76 | 1.89 | 0.000 | - && Q4WAZ6.1 RecName: Full=Multifunctional cytochrome P450 monooxygenase af510; AltName: Full=Fumagillin bioynthesis cluster P450 monooxygenase; Short=Fma-P450 && PF00067:Cytochrome P450\|PF07859:alpha/beta hydrolase fold |
| A09947 | Contig8 | 1224325 | 1225564 | - | 972 |  | 0.61 | 0.41 | 0.85 | 3.94 | 5.20 | 5.24 | -2.94 | 0.000 | - && - && - |
| A09949 | Contig8 | 1239915 | 1241915 | + | 1437 |  | 1.44 | 2.45 | 1.97 | 5.53 | 8.60 | 3.30 | -1.57 | 0.000 | - && B8NHY4.1 RecName: Full=O-methylsterigmatocystin oxidoreductase; Short=OMST oxidoreductase; AltName: Full=Aflatoxin B synthase; AltName: Full=Aflatoxin biosynthesis protein Q; AltName: Full=Cytochrome P450 64 >P0CT93.1 RecName: Full=O-methylsterigmatocystin oxidoreductase; Short=OMST oxidoreductase; AltName: Full=Aflatoxin B synthase; AltName: Full=Aflatoxin biosynthesis protein Q; AltName: Full=Cytochrome P450 64 && PF00067:Cytochrome P450 |
| A09950 | Contig8 | 1242197 | 1244194 | - | 1497 |  | 1.91 | 1.21 | 1.40 | 6.18 | 13.76 | 3.34 | -2.36 | 0.000 | - && B8NHY4.1 RecName: Full=O-methylsterigmatocystin oxidoreductase; Short=OMST oxidoreductase; AltName: Full=Aflatoxin B synthase; AltName: Full=Aflatoxin biosynthesis protein Q; AltName: Full=Cytochrome P450 64 >P0CT93.1 RecName: Full=O-methylsterigmatocystin oxidoreductase; Short=OMST oxidoreductase; AltName: Full=Aflatoxin B synthase; AltName: Full=Aflatoxin biosynthesis protein Q; AltName: Full=Cytochrome P450 64 && PF00067:Cytochrome P450 |
| A09956 | Contig8 | 1270839 | 1274613 | + | 1713 |  | 5.53 | 7.27 | 5.39 | 3.44 | 2.90 | 1.69 | 1.18 | 0.000 | - && - && - |
| A09959 | Contig8 | 1281753 | 1282665 | - | 804 |  | 12.39 | 25.72 | 8.75 | 6.28 | 2.68 | 6.55 | 1.60 | 0.000 | - && P0CU01.1 RecName: Full=Uncharacterized oxidoreductase SPBC1348.09 >P0CU00.1 RecName: Full=Uncharacterized oxidoreductase SPAC977.08 && PF00106:short chain dehydrogenase |
| A09971 | Contig8 | 1309463 | 1311850 | - | 1629 |  | 14.16 | 12.63 | 16.77 | 32.03 | 47.70 | 28.55 | -1.31 | 0.000 | - && A1CP08.1 RecName: Full=Probable mannosyl-oligosaccharide alpha-1,2-mannosidase 1B; AltName: Full=Class I alpha-mannosidase 1B; AltName: Full=Man(9)-alpha-mannosidase 1B; Flags: Precursor && PF01532:Glycosyl hydrolase family 47 |
| A09982 | Contig8 | 1376835 | 1378397 | - | 1173 |  | 23.03 | 18.06 | 23.83 | 7.57 | 9.26 | 6.28 | 1.49 | 0.000 | - && - && - |
| A10000 | Contig8 | 1455725 | 1460347 | + | 2490 |  | 19.68 | 20.48 | 22.12 | 57.41 | 55.08 | 46.66 | -1.35 | 0.000 | - && D4ANU4.1 RecName: Full=N,O-diacetylmuramidase; AltName: Full=Lysozyme CH; Flags: Precursor && PF01183:Glycosyl hydrolases family 25\|PF07690:Major Facilitator Superfamily |
| A10025 | Contig8 | 1545893 | 1549142 | + | 2091 |  | 0.71 | 0.62 | 0.48 | 0.27 | 0.22 | 0.21 | 1.37 | 0.047 | - && P14868.2 RecName: Full=Aspartate--tRNA ligase, cytoplasmic; AltName: Full=Aspartyl-tRNA synthetase; Short=AspRS; AltName: Full=Cell proliferation-inducing gene 40 protein && PF15902:Sortilin, neurotensin receptor 3,\|PF00152:tRNA synthetases class II (D, K and N) |
| A10031 | Contig8 | 1567298 | 1569628 | + | 1644 |  | 4.74 | 6.78 | 4.61 | 41.86 | 46.81 | 36.46 | -2.96 | 0.000 | - && P11707.2 RecName: Full=Cytochrome P450 3A6; AltName: Full=CYPIIIA6; AltName: Full=Cytochrome P450-3C && PF00067:Cytochrome P450 |
| A10040 | Contig9 | 403343 | 405664 | - | 966 |  | 68.08 | 61.52 | 84.73 | 24.68 | 19.87 | 22.89 | 1.67 | 0.000 | - && - && - |
| A10046 | Contig9 | 417298 | 424167 | + | 4131 |  | 6.52 | 6.76 | 6.35 | 12.49 | 15.05 | 12.47 | -1.03 | 0.000 | - && B0XMP7.2 RecName: Full=Probable beta-galactosidase A; AltName: Full=Lactase A; Flags: Precursor && PF01301:Glycosyl hydrolases family 35\|PF10435:Beta-galactosidase, domain 2\|PF13364:Beta-galactosidase jelly roll domain\|PF13363:Beta-galactosidase, domain 3 |
| A10055 | Contig9 | 62331 | 64234 | + | 1296 |  | 12.55 | 15.18 | 9.87 | 21.86 | 26.87 | 29.59 | -1.06 | 0.000 | - && Q9BRP1.1 RecName: Full=Programmed cell death protein 2-like && PF04194:Programmed cell death protein 2, C-terminal putative domain |
| A10056 | Contig9 | 448008 | 449616 | - | 798 |  | 19.40 | 20.13 | 15.57 | 165.79 | 179.83 | 137.58 | -3.13 | 0.000 | - && A1DBT4.1 RecName: Full=Probable pectinesterase A; AltName: Full=Pectin methylesterase A; Flags: Precursor && PF01095:Pectinesterase |
| A10058 | Contig9 | 454024 | 455138 | + | 1056 |  | 16.43 | 8.37 | 12.98 | 5.22 | 8.07 | 3.82 | 1.14 | 0.001 | - && - && - |
| A10072 | Contig9 | 521164 | 523216 | + | 1653 |  | 47.48 | 37.11 | 87.04 | 126.19 | 198.38 | 94.02 | -1.29 | 0.000 | - && - && - |
| A10073 | Contig9 | 524964 | 525465 | - | 381 |  | 230.59 | 321.45 | 241.44 | 91.04 | 83.29 | 59.43 | 1.76 | 0.000 | - && - && - |
| A10075 | Contig9 | 533838 | 534292 | - | 402 |  | 5.64 | 14.98 | 9.54 | 1.86 | 2.79 | 4.58 | 1.71 | 0.001 | - && - && - |
| A10084 | Contig9 | 564222 | 566521 | + | 2187 |  | 2.61 | 2.11 | 2.38 | 4.79 | 6.21 | 5.42 | -1.21 | 0.000 | - && - && - |
| A10086 | Contig9 | 571044 | 572839 | + | 1632 |  | 1.09 | 1.35 | 0.84 | 2.69 | 2.24 | 2.42 | -1.16 | 0.005 | - && - && - |
| A10087 | Contig9 | 573261 | 575459 | - | 1365 |  | 13.22 | 15.37 | 13.99 | 71.17 | 65.29 | 63.52 | -2.23 | 0.000 | - && Q5B6Q3.1 RecName: Full=Glucan endo-1,6-beta-glucosidase B; AltName: Full=Beta-1,6-glucanase B; AltName: Full=Endo-1,6-beta-D-glucanase B; AltName: Full=Endo-1,6-beta-glucanase B; Flags: Precursor && PF00150:Cellulase (glycosyl hydrolase family 5) |
| A10096 | Contig9 | 598472 | 604852 | - | 2991 |  | 3.07 | 3.66 | 3.42 | 2.19 | 1.69 | 1.00 | 1.06 | 0.001 | - && - && PF04082:Fungal specific transcription factor domain |
| A10114 | Contig9 | 700026 | 702019 | + | 1533 |  | 174.50 | 142.95 | 150.58 | 53.18 | 90.31 | 82.21 | 1.05 | 0.000 | - && Q92459.2 RecName: Full=Squalene synthase; Short=SQS; Short=SS; AltName: Full=FPP:FPP farnesyltransferase; AltName: Full=Farnesyl-diphosphate farnesyltransferase && PF00494:Squalene/phytoene synthase |
| A10121 | Contig9 | 81153 | 87067 | - | 5067 |  | 0.41 | 0.36 | 0.27 | 0.33 | 1.51 | 0.76 | -1.33 | 0.014 | - && P29742.1 RecName: Full=Clathrin heavy chain && PF13838:Clathrin-H-link\|PF09268:Clathrin, heavy-chain linker\|PF00637:Region in Clathrin and VPS\|PF01394:Clathrin propeller repeat |
| A10129 | Contig9 | 752959 | 761325 | + | 5238 |  | 6.70 | 9.03 | 7.78 | 18.26 | 25.61 | 23.47 | -1.52 | 0.000 | - && Q4WY82.2 RecName: Full=Linoleate 10R-lipoxygenase; AltName: Full=Cyclooxygenase-like fatty acid oxygenase; AltName: Full=Fatty acid oxygenase ppoC; AltName: Full=Linoleate 10R-dioxygenase; Short=10R-DOX; AltName: Full=Psi-producing oxygenase C; Short=AfPpoC && PF03098:Animal haem peroxidase\|PF00067:Cytochrome P450 |
| A10134 | Contig9 | 772428 | 774873 | + | 1686 |  | 8.77 | 6.37 | 6.18 | 3.49 | 1.50 | 3.33 | 1.36 | 0.000 | - && - && - |
| A10149 | Contig9 | 820167 | 824282 | - | 2214 |  | 124.61 | 71.01 | 132.44 | 17.31 | 22.79 | 14.15 | 2.60 | 0.000 | - && P36032.2 RecName: Full=Probable transporter MCH2 && PF07690:Major Facilitator Superfamily |
| A10150 | Contig9 | 824643 | 826425 | - | 1461 |  | 138.29 | 63.63 | 164.07 | 17.34 | 16.59 | 5.65 | 3.21 | 0.000 | - && - && PF07690:Major Facilitator Superfamily |
| A10153 | Contig9 | 834214 | 836398 | - | 1605 |  | 2.03 | 3.25 | 2.16 | 1.05 | 0.93 | 1.04 | 1.30 | 0.002 | - && A1CFL5.1 RecName: Full=Cytochrome P450 monooxygenase patH; AltName: Full=Patulin synthesis protein H; AltName: Full=m-cresol hydrolase; Flags: Precursor && PF00067:Cytochrome P450 |
| A10157 | Contig9 | 842382 | 845383 | - | 1971 |  | 51.88 | 41.87 | 46.81 | 19.83 | 11.49 | 17.54 | 1.52 | 0.000 | - && - && PF07690:Major Facilitator Superfamily |
| A10166 | Contig9 | 94284 | 95743 | - | 978 |  | 5.04 | 5.03 | 2.52 | 10.23 | 7.27 | 7.81 | -1.01 | 0.004 | - && Q9UTE6.1 RecName: Full=Protein mak16 && PF01778:Ribosomal L28e protein family\|PF04874:Mak16 protein C-terminal region |
| A10171 | Contig9 | 891556 | 893130 | - | 1410 |  | 547.49 | 436.58 | 256.10 | 115.91 | 130.99 | 309.71 | 1.16 | 0.001 | - && Q5R7Z9.1 RecName: Full=Hydroxymethylglutaryl-CoA synthase, cytoplasmic; Short=HMG-CoA synthase; AltName: Full=3-hydroxy-3-methylglutaryl coenzyme A synthase && PF01154:Hydroxymethylglutaryl-coenzyme A synthase N terminal\|PF08540:Hydroxymethylglutaryl-coenzyme A synthase C terminal |
| A10176 | Contig9 | 907356 | 908201 | - | 846 |  | 113.06 | 124.24 | 99.23 | 218.71 | 180.69 | 280.50 | -1.01 | 0.000 | - && - && - |
| A10184 | Contig9 | 924677 | 926187 | - | 1458 |  | 9.94 | 10.53 | 5.01 | 18.21 | 17.27 | 18.48 | -1.08 | 0.000 | - && - && - |
| A10196 | Contig9 | 969562 | 972750 | + | 2274 |  | 14.53 | 16.07 | 15.18 | 7.15 | 6.46 | 7.29 | 1.13 | 0.000 | - && Q99P66.1 RecName: Full=H(+)/Cl(-) exchange transporter 5; AltName: Full=Chloride channel protein 5; Short=ClC-5; AltName: Full=Chloride transporter ClC-5 && PF00654:Voltage gated chloride channel |
| A10204 | Contig9 | 1000622 | 1001859 | + | 1161 |  | 29.64 | 30.61 | 25.65 | 11.84 | 10.88 | 6.50 | 1.56 | 0.000 | - && - && PF07247:Alcohol acetyltransferase |
| A10215 | Contig9 | 1041431 | 1042455 | + | 903 |  | 10.26 | 18.23 | 12.95 | 33.24 | 37.32 | 19.05 | -1.11 | 0.000 | - && - && PF07264:Etoposide-induced protein 2.4 (EI24) |
| A10216 | Contig9 | 1042835 | 1045690 | + | 1878 |  | 59.80 | 56.93 | 50.15 | 7.77 | 11.66 | 6.12 | 2.71 | 0.000 | - && Q0USX0.1 RecName: Full=Pheromone-processing carboxypeptidase KEX1; AltName: Full=Carboxypeptidase D; Flags: Precursor && PF00450:Serine carboxypeptidase |
| A10220 | Contig9 | 1057171 | 1059081 | + | 1170 |  | 315.11 | 354.45 | 284.59 | 147.20 | 110.49 | 116.64 | 1.35 | 0.000 | - && Q8IYS1.2 RecName: Full=Peptidase M20 domain-containing protein 2; AltName: Full=Aminoacylase-1-like protein 2 && PF01546:Peptidase family M20/M25/M40\|PF07687:Peptidase dimerisation domain |
| A10229 | Contig9 | 1092123 | 1097858 | + | 3120 |  | 802.79 | 542.55 | 1110.07 | 367.99 | 363.37 | 365.72 | 1.16 | 0.000 | - && O74445.2 RecName: Full=Probable 26S protease subunit rpt4 && PF00004:ATPase family associated with various cellular activities (AAA)\|PF00450:Serine carboxypeptidase |
| A10233 | Contig9 | 1100887 | 1102017 | - | 999 |  | 18.56 | 15.48 | 20.85 | 9.36 | 8.34 | 7.64 | 1.11 | 0.000 | - && Q54T76.1 RecName: Full=Probable dual specificity protein phosphatase DDB_G0281963 && PF00782:Dual specificity phosphatase, catalytic domain |
| A10235 | Contig9 | 1109568 | 1111048 | - | 1074 |  | 11.57 | 14.21 | 14.63 | 30.99 | 55.70 | 117.83 | -2.34 | 0.000 | - && Q9SLN8.1 RecName: Full=2-alkenal reductase (NADP(+)-dependent); AltName: Full=Alkenal double bound reductase; AltName: Full=Allylic alcohol dehydrogenase 1; Short=allyl-ADH1; AltName: Full=Flavin-free double bond reductase; Short=NtDBR; AltName: Full=Pulegone reductase; Short=NtRed-1 && PF16884:N-terminal domain of oxidoreductase\|PF00107:Zinc-binding dehydrogenase |
| A10255 | Contig9 | 140990 | 142414 | + | 1092 |  | 73.86 | 71.89 | 48.10 | 11.22 | 13.46 | 10.37 | 2.47 | 0.000 | - && Q01284.1 RecName: Full=Nitronate monooxygenase; AltName: Full=2-nitropropane dioxygenase; Short=2-NPD; AltName: Full=Nitroalkane oxidase; Flags: Precursor && PF03060:Nitronate monooxygenase |
| A10256 | Contig9 | 1206345 | 1207662 | + | 846 |  | 60.72 | 43.91 | 92.75 | 304.14 | 344.68 | 173.96 | -2.06 | 0.000 | - && - && PF01764:Lipase (class 3) |
| A10287 | Contig9 | 1310490 | 1312357 | - | 1584 |  | 10.33 | 11.03 | 12.51 | 4.78 | 2.78 | 3.32 | 1.64 | 0.000 | - && - && PF01266:FAD dependent oxidoreductase |
| A10289 | Contig9 | 1312664 | 1314505 | - | 1581 |  | 1.31 | 0.70 | 2.72 | 0.30 | 0.36 | 0.83 | 1.67 | 0.011 | - && P37906.2 RecName: Full=Gamma-glutamylputrescine oxidoreductase; Short=Gamma-Glu-Put oxidase; Short=Gamma-glutamylputrescine oxidase && PF01266:FAD dependent oxidoreductase |
| A10343 | Contig9 | 192805 | 195104 | - | 846 |  | 745.70 | 880.83 | 294.78 | 218.82 | 104.12 | 188.28 | 1.91 | 0.000 | - && - && - |
| A10345 | Contig9 | 204343 | 205459 | + | 879 |  | 1063.88 | 1969.50 | 262.10 | 68.39 | 38.13 | 41.83 | 4.47 | 0.000 | - && - && - |
| A10348 | Contig9 | 213022 | 213969 | - | 816 |  | 39.88 | 44.41 | 46.91 | 114.35 | 151.31 | 161.54 | -1.70 | 0.000 | - && P06182.1 RecName: Full=Cytochrome c heme lyase; Short=CCHL; AltName: Full=Holocytochrome-c synthase && PF01265:Cytochrome c/c1 heme lyase |
| A10358 | Contig9 | 241500 | 246418 | - | 3285 |  | 12.37 | 12.71 | 11.43 | 38.17 | 51.15 | 38.98 | -1.81 | 0.000 | - && D4AUF4.1 RecName: Full=WSC domain-containing protein ARB_07870; Flags: Precursor && PF00141:Peroxidase |
| novel | Contig24 | 220232 | 222724 | + | 2283 |  | 6.74 | 7.26 | 7.12 | 2.74 | 4.55 | 2.34 | 1.13 | 0.000 | - && - && - |
| novel | Contig1 | 4396761 | 4397368 | + | 520 |  | 3.79 | 3.86 | 4.04 | 1.80 | 1.62 | 0.17 | 1.70 | 0.011 | - && - && - |
| novel | Contig24 | 281556 | 283009 | - | 1214 |  | 0.32 | 0.58 | 0.23 | 1.39 | 1.16 | 1.16 | -1.71 | 0.014 | - && - && - |
| novel | Contig25 | 26360 | 28561 | - | 2011 |  | 9.56 | 8.54 | 7.95 | 4.28 | 2.79 | 1.27 | 1.64 | 0.000 | - && - && - |
| novel | Contig1 | 4411939 | 4414912 | + | 2617 |  | 401.12 | 313.29 | 301.35 | 25.22 | 14.92 | 22.67 | 4.02 | 0.000 | - && O94510.2 RecName: Full=Glucan endo-1,3-alpha-glucosidase agn2; AltName: Full=Endo-1,3-alpha-glucanase agn2 && PF03659:Glycosyl hydrolase family 71 |
| novel | Contig26 | 127014 | 128207 | + | 1194 |  | 0.00 | 0.08 | 0.00 | 0.39 | 0.78 | 0.44 | -4.27 | 0.006 | - && - && - |
| novel | Contig26 | 125886 | 126807 | - | 865 |  | 0.57 | 0.70 | 0.11 | 2.92 | 1.95 | 1.32 | -2.17 | 0.003 | - && - && - |
| novel | Contig27 | 15447 | 16135 | - | 503 |  | 1.57 | 2.99 | 1.09 | 3.53 | 4.84 | 7.68 | -1.51 | 0.008 | - && - && - |
| novel | Contig27 | 111287 | 112365 | - | 1079 |  | 0.55 | 1.12 | 1.52 | 2.95 | 4.34 | 3.01 | -1.69 | 0.001 | - && - && - |
| novel | Contig29 | 271168 | 272710 | + | 1410 |  | 0.28 | 0.21 | 0.32 | 0.99 | 0.46 | 0.93 | -1.55 | 0.048 | - && - && - |
| novel | Contig29 | 250826 | 251603 | - | 672 |  | 4.55 | 6.72 | 6.12 | 1.11 | 0.28 | 0.39 | 3.29 | 0.000 | - && - && - |
| novel | Contig29 | 265289 | 266676 | - | 1279 |  | 0.15 | 0.00 | 0.07 | 0.29 | 0.15 | 1.58 | -3.16 | 0.018 | - && - && - |
| novel | Contig3 | 209765 | 210419 | + | 536 |  | 6.81 | 8.24 | 5.79 | 18.49 | 20.08 | 14.90 | -1.36 | 0.000 | - && - && - |
| novel | Contig3 | 518802 | 520379 | + | 1524 |  | 4.33 | 3.43 | 3.96 | 1.29 | 1.97 | 0.63 | 1.59 | 0.000 | - && - && - |
| novel | Contig3 | 651323 | 656994 | + | 5609 |  | 3.62 | 3.36 | 4.36 | 1.33 | 1.97 | 1.28 | 1.31 | 0.000 | - && - && - |
| novel | Contig3 | 915408 | 916212 | + | 755 |  | 6.66 | 5.72 | 6.17 | 2.11 | 4.22 | 2.67 | 1.04 | 0.008 | - && - && - |
| novel | Contig3 | 403189 | 404647 | - | 1260 |  | 3.13 | 4.70 | 7.47 | 2.15 | 1.93 | 1.39 | 1.48 | 0.000 | - && - && - |
| novel | Contig3 | 562010 | 563909 | - | 1845 |  | 2.03 | 0.76 | 1.34 | 0.41 | 0.51 | 0.67 | 1.39 | 0.013 | - && - && - |
| novel | Contig3 | 804912 | 805983 | - | 1072 |  | 2.02 | 1.50 | 0.85 | 3.05 | 3.58 | 2.87 | -1.12 | 0.015 | - && - && - |
| novel | Contig1 | 408943 | 410002 | - | 995 |  | 14.07 | 5.85 | 19.19 | 62.68 | 162.20 | 96.58 | -3.04 | 0.000 | - && - && PF04140:Isoprenylcysteine carboxyl methyltransferase (ICMT) family |
| novel | Contig1 | 410697 | 411823 | - | 1005 |  | 1.28 | 1.40 | 0.82 | 2.05 | 3.91 | 2.18 | -1.22 | 0.019 | - && - && PF04140:Isoprenylcysteine carboxyl methyltransferase (ICMT) family |
| novel | Contig3 | 1239116 | 1239852 | - | 681 |  | 2.46 | 1.92 | 1.21 | 0.27 | 0.41 | 0.13 | 2.78 | 0.002 | - && - && - |
| novel | Contig3 | 2431229 | 2431991 | - | 622 |  | 2.54 | 2.58 | 3.38 | 0.30 | 0.90 | 1.83 | 1.48 | 0.027 | - && - && - |
| novel | Contig30 | 184529 | 185997 | + | 1179 |  | 1.76 | 1.96 | 2.25 | 0.71 | 0.56 | 0.15 | 2.07 | 0.001 | - && - && - |
| novel | Contig31 | 81440 | 83146 | + | 1707 |  | 0.75 | 0.35 | 0.64 | 1.20 | 1.04 | 1.75 | -1.19 | 0.030 | - && - && - |
| novel | Contig31 | 187239 | 189371 | - | 1978 |  | 2.09 | 0.46 | 0.23 | 0.33 | 0.38 | 0.09 | 1.80 | 0.036 | - && - && - |
| novel | Contig32 | 117811 | 120552 | - | 2178 |  | 23.95 | 17.42 | 27.14 | 6.83 | 8.90 | 6.69 | 1.61 | 0.000 | - && - && - |
| novel | Contig33 | 172699 | 174020 | + | 1141 |  | 1.12 | 3.34 | 2.72 | 0.66 | 1.31 | 0.46 | 1.56 | 0.009 | - && - && - |
| novel | Contig33 | 174388 | 178742 | + | 3967 |  | 3.65 | 3.04 | 2.37 | 1.32 | 1.16 | 0.55 | 1.58 | 0.000 | - && Q55EI8.1 RecName: Full=Probable serine/threonine-protein kinase DDB_G0268876 && PF07714:Protein tyrosine and serine/threonine kinase\|PF00069:Protein kinase domain |
| novel | Contig1 | 551612 | 552427 | - | 751 |  | 1.05 | 1.20 | 0.73 | 2.74 | 3.24 | 1.99 | -1.42 | 0.015 | - && - && - |
| novel | Contig35 | 154087 | 156193 | + | 1890 |  | 0.21 | 0.27 | 0.39 | 1.58 | 0.69 | 1.07 | -1.96 | 0.002 | - && - && - |
| novel | Contig35 | 181787 | 188759 | + | 6768 |  | 0.76 | 0.96 | 0.94 | 2.56 | 1.63 | 1.85 | -1.18 | 0.000 | - && - && - |
| novel | Contig35 | 28068 | 29409 | - | 1073 |  | 4.04 | 3.18 | 3.41 | 2.44 | 0.96 | 1.88 | 1.01 | 0.021 | - && - && PF00172:Fungal Zn(2)-Cys(6) binuclear cluster domain |
| novel | Contig1 | 611230 | 613177 | - | 1703 |  | 14.07 | 12.61 | 9.49 | 119.74 | 61.40 | 92.96 | -2.92 | 0.000 | - && - && - |
| novel | Contig36 | 52128 | 53123 | - | 922 |  | 0.53 | 0.33 | 0.40 | 1.12 | 2.23 | 2.28 | -2.16 | 0.002 | - && - && - |
| novel | Contig39 | 100814 | 101643 | + | 664 |  | 4.75 | 4.38 | 6.33 | 2.25 | 1.13 | 1.45 | 1.68 | 0.000 | - && - && - |
| novel | Contig4 | 557949 | 558437 | + | 437 |  | 2.03 | 0.92 | 1.05 | 7.27 | 12.64 | 2.41 | -2.48 | 0.000 | - && - && - |
| novel | Contig4 | 1494952 | 1496074 | + | 1065 |  | 2.68 | 2.07 | 3.43 | 0.44 | 1.76 | 0.58 | 1.56 | 0.007 | - && - && - |
| novel | Contig4 | 1567842 | 1571199 | + | 2956 |  | 1.70 | 2.28 | 1.45 | 0.76 | 1.05 | 0.59 | 1.18 | 0.002 | - && - && - |
| novel | Contig4 | 1845672 | 1846510 | + | 787 |  | 1.13 | 1.28 | 2.44 | 0.71 | 0.48 | 0.11 | 1.90 | 0.020 | - && - && - |
| novel | Contig4 | 1874589 | 1875573 | + | 910 |  | 1.41 | 1.43 | 2.41 | 0.41 | 0.72 | 0.77 | 1.46 | 0.024 | - && - && - |
| novel | Contig4 | 2062962 | 2063776 | + | 700 |  | 88.88 | 76.73 | 106.23 | 204.49 | 263.71 | 120.61 | -1.12 | 0.000 | - && - && - |
| novel | Contig4 | 2064417 | 2066653 | + | 2106 |  | 6.32 | 6.01 | 4.29 | 2.71 | 1.96 | 1.88 | 1.35 | 0.000 | - && - && - |
| novel | Contig4 | 2066691 | 2070503 | + | 3040 |  | 26.08 | 22.85 | 41.74 | 14.33 | 19.00 | 10.77 | 1.04 | 0.000 | - && P12807.1 RecName: Full=Peroxisomal primary amine oxidase; AltName: Full=Copper amine oxidase; AltName: Full=Methylamine oxidase && PF02728:Copper amine oxidase, N3 domain\|PF01179:Copper amine oxidase, enzyme domain |
| novel | Contig4 | 91692 | 92811 | - | 1010 |  | 14.55 | 15.51 | 12.12 | 4.91 | 3.89 | 2.09 | 1.95 | 0.000 | - && - && - |
| novel | Contig4 | 1923807 | 1926012 | - | 2087 |  | 2.36 | 2.31 | 3.02 | 5.91 | 6.37 | 5.89 | -1.24 | 0.000 | - && - && - |
| novel | Contig4 | 1970149 | 1971661 | - | 1329 |  | 8.61 | 7.02 | 8.25 | 2.46 | 4.37 | 4.75 | 1.04 | 0.001 | - && - && - |
| novel | Contig1 | 1324153 | 1325101 | - | 712 |  | 719.86 | 599.79 | 656.77 | 1973.00 | 1281.10 | 1110.29 | -1.14 | 0.000 | - && Q4HXT6.2 RecName: Full=Plasma membrane proteolipid 3 && PF01679:Proteolipid membrane potential modulator |
| novel | Contig4 | 2434550 | 2437052 | - | 1901 |  | 155.61 | 194.81 | 146.52 | 62.17 | 72.14 | 71.92 | 1.27 | 0.000 | - && P41764.2 RecName: Full=Glucose-6-phosphate 1-dehydrogenase; Short=G6PD && PF00479:Glucose-6-phosphate dehydrogenase, NAD binding domain\|PF02781:Glucose-6-phosphate dehydrogenase, C-terminal domain |
| novel | Contig41 | 111622 | 112978 | - | 1232 |  | 1.44 | 2.36 | 0.96 | 0.46 | 0.00 | 0.28 | 2.69 | 0.001 | - && - && - |
| novel | Contig42 | 75366 | 76801 | + | 1385 |  | 0.21 | 0.58 | 0.33 | 2.09 | 1.55 | 0.70 | -1.95 | 0.005 | - && - && - |
| novel | Contig42 | 81798 | 82522 | - | 392 |  | 2.01 | 1.79 | 1.63 | 6.92 | 12.42 | 4.25 | -2.12 | 0.000 | - && - && - |
| novel | Contig46 | 100308 | 101779 | - | 1181 |  | 0.42 | 0.77 | 0.62 | 0.24 | 0.16 | 0.00 | 2.19 | 0.045 | - && - && - |
| novel | Contig5 | 56110 | 56620 | + | 418 |  | 5.66 | 4.08 | 5.03 | 2.46 | 3.14 | 1.26 | 1.11 | 0.046 | - && - && - |
| novel | Contig5 | 309372 | 313538 | + | 3580 |  | 9.12 | 7.60 | 5.41 | 2.45 | 2.04 | 1.20 | 1.96 | 0.000 | - && - && - |
| novel | Contig5 | 412030 | 412423 | + | 328 |  | 3.61 | 3.98 | 1.39 | 0.86 | 0.57 | 0.27 | 2.41 | 0.013 | - && - && - |
| novel | Contig5 | 810336 | 812505 | + | 1950 |  | 2.93 | 1.96 | 3.42 | 1.10 | 1.30 | 0.50 | 1.52 | 0.000 | - && - && - |
| novel | Contig5 | 1144034 | 1146514 | + | 1902 |  | 7.15 | 6.39 | 4.27 | 6.73 | 29.04 | 21.46 | -1.68 | 0.000 | - && - && PF13673:Acetyltransferase (GNAT) domain |
| novel | Contig5 | 1616615 | 1619092 | + | 1933 |  | 2.91 | 1.14 | 3.88 | 12.14 | 7.60 | 6.17 | -1.71 | 0.000 | - && - && - |
| novel | Contig1 | 2933908 | 2935249 | - | 1073 |  | 3.31 | 4.49 | 1.53 | 1.22 | 1.40 | 1.72 | 1.11 | 0.027 | - && - && PF00172:Fungal Zn(2)-Cys(6) binuclear cluster domain |
| novel | Contig5 | 1868332 | 1868910 | + | 404 |  | 0.24 | 0.25 | 0.00 | 3.24 | 0.93 | 2.82 | -3.83 | 0.004 | - && - && - |
| novel | Contig5 | 211115 | 213152 | - | 1748 |  | 0.73 | 0.11 | 1.10 | 1.60 | 1.66 | 1.46 | -1.28 | 0.035 | - && - && - |
| novel | Contig5 | 442477 | 443211 | - | 497 |  | 1434.80 | 1593.89 | 1445.60 | 675.92 | 418.89 | 514.90 | 1.47 | 0.000 | - && - && - |
| novel | Contig5 | 703458 | 705342 | - | 1605 |  | 39.20 | 27.40 | 54.18 | 16.95 | 18.26 | 9.57 | 1.43 | 0.000 | - && - && - |
| novel | Contig5 | 1018931 | 1019302 | - | 272 |  | 6.16 | 8.49 | 6.38 | 3.09 | 0.34 | 2.26 | 1.88 | 0.006 | - && - && - |
| novel | Contig1 | 3177539 | 3179657 | - | 1815 |  | 0.33 | 1.16 | 1.26 | 2.58 | 2.32 | 1.98 | -1.33 | 0.006 | - && P56502.2 RecName: Full=Pheromone B alpha 3 receptor && PF02076:Pheromone A receptor |
| novel | Contig5 | 1397225 | 1398408 | - | 1066 |  | 4.16 | 3.86 | 2.66 | 1.32 | 0.61 | 0.41 | 2.19 | 0.000 | - && - && - |
| novel | Contig5 | 1573230 | 1573905 | - | 560 |  | 1.58 | 1.43 | 1.96 | 0.50 | 0.67 | 0.00 | 2.09 | 0.028 | - && - && - |
| novel | Contig5 | 1854110 | 1855117 | - | 948 |  | 12.17 | 10.48 | 11.27 | 6.31 | 2.57 | 2.96 | 1.52 | 0.000 | - && B8NM67.1 RecName: Full=Oxidase ustYa; AltName: Full=Ustiloxin B biosynthesis protein Ya && PF11807:Mycotoxin biosynthesis protein UstYa |
| novel | Contig5 | 1922537 | 1923798 | - | 1039 |  | 1.42 | 2.80 | 2.46 | 0.09 | 0.09 | 0.34 | 3.69 | 0.000 | - && - && - |
| novel | Contig5 | 2087553 | 2089237 | - | 1245 |  | 24.63 | 37.25 | 37.86 | 209.37 | 273.16 | 99.32 | -2.54 | 0.000 | - && - && - |
| novel | Contig5 | 2269345 | 2271278 | - | 1638 |  | 13.36 | 15.38 | 20.41 | 30.65 | 33.95 | 37.99 | -1.06 | 0.000 | - && - && - |
| novel | Contig5 | 2288185 | 2288877 | - | 573 |  | 3.10 | 1.93 | 3.99 | 0.16 | 0.82 | 0.31 | 2.81 | 0.000 | - && - && - |
| novel | Contig5 | 2522679 | 2530355 | - | 6782 |  | 40.42 | 52.61 | 25.17 | 15.01 | 15.75 | 17.66 | 1.29 | 0.000 | - && Q91W86.3 RecName: Full=Vacuolar protein sorting-associated protein 11 homolog && PF00637:Region in Clathrin and VPS |
| novel | Contig1 | 3263008 | 3263753 | - | 684 |  | 9.08 | 7.49 | 8.55 | 3.28 | 3.28 | 4.62 | 1.17 | 0.001 | - && - && - |
| novel | Contig50 | 42741 | 43481 | - | 681 |  | 0.14 | 0.15 | 0.27 | 1.51 | 1.92 | 1.16 | -3.04 | 0.003 | - && - && - |
| novel | Contig52 | 1689 | 3624 | - | 1752 |  | 1.74 | 1.09 | 0.99 | 0.48 | 0.43 | 0.45 | 1.49 | 0.007 | - && - && - |
| novel | Contig1 | 3268819 | 3269473 | - | 590 |  | 15.04 | 21.44 | 14.24 | 8.08 | 7.14 | 7.14 | 1.18 | 0.000 | - && - && - |
| novel | Contig6 | 220422 | 222971 | + | 2380 |  | 0.95 | 0.46 | 0.27 | 4.79 | 1.69 | 1.00 | -2.15 | 0.000 | - && - && PF00172:Fungal Zn(2)-Cys(6) binuclear cluster domain |
| novel | Contig6 | 314028 | 314962 | + | 864 |  | 0.57 | 0.70 | 1.59 | 2.27 | 2.82 | 1.93 | -1.30 | 0.032 | - && - && - |
| novel | Contig6 | 317087 | 319844 | + | 2038 |  | 2.56 | 1.43 | 1.17 | 0.41 | 0.05 | 0.04 | 3.36 | 0.000 | - && - && - |
| novel | Contig6 | 946498 | 947825 | + | 1058 |  | 2.61 | 2.47 | 3.11 | 0.71 | 2.12 | 0.75 | 1.19 | 0.024 | - && - && - |
| novel | Contig6 | 1441070 | 1442212 | + | 1091 |  | 1.81 | 1.20 | 1.67 | 3.51 | 3.78 | 2.82 | -1.11 | 0.009 | - && - && - |
| novel | Contig6 | 1610584 | 1612876 | + | 1879 |  | 6.98 | 10.20 | 11.04 | 3.63 | 3.49 | 1.91 | 1.64 | 0.000 | - && - && - |
| novel | Contig6 | 76840 | 80051 | - | 3161 |  | 1.00 | 1.75 | 1.16 | 0.38 | 0.77 | 0.64 | 1.12 | 0.010 | - && - && - |
| novel | Contig6 | 274773 | 286783 | - | 8410 |  | 4.03 | 3.49 | 3.10 | 10.96 | 11.60 | 11.70 | -1.69 | 0.000 | - && - && - |
| novel | Contig6 | 502016 | 503130 | - | 985 |  | 0.20 | 0.51 | 0.65 | 1.33 | 1.52 | 2.14 | -1.88 | 0.008 | - && - && - |
| novel | Contig6 | 974833 | 975612 | - | 503 |  | 0.78 | 0.40 | 1.27 | 2.60 | 4.09 | 2.27 | -1.87 | 0.011 | - && - && - |
| novel | Contig6 | 1360045 | 1360516 | - | 416 |  | 3.08 | 3.86 | 0.88 | 8.09 | 7.20 | 3.59 | -1.27 | 0.033 | - && - && - |
| novel | Contig6 | 1510339 | 1511905 | - | 1219 |  | 98.44 | 77.82 | 76.96 | 6.44 | 6.14 | 2.45 | 4.07 | 0.000 | - && - && - |
| novel | Contig6 | 1527584 | 1528900 | - | 1147 |  | 14.44 | 13.92 | 15.77 | 0.90 | 0.57 | 0.46 | 4.52 | 0.000 | - && - && - |
| novel | Contig6 | 2045848 | 2047446 | - | 1425 |  | 3.46 | 4.51 | 1.73 | 0.20 | 0.26 | 0.37 | 3.55 | 0.000 | - && - && - |
| novel | Contig6 | 2219039 | 2221412 | - | 2148 |  | 3.63 | 3.04 | 2.21 | 1.39 | 1.39 | 1.23 | 1.15 | 0.001 | - && - && - |
| novel | Contig7 | 154391 | 156849 | + | 2129 |  | 12.41 | 12.54 | 9.05 | 5.40 | 4.40 | 5.03 | 1.20 | 0.000 | - && - && - |
| novel | Contig7 | 553503 | 555952 | + | 2276 |  | 0.78 | 1.50 | 0.40 | 3.41 | 1.97 | 1.43 | -1.35 | 0.008 | - && - && - |
| novel | Contig7 | 811773 | 812737 | + | 782 |  | 3.28 | 1.80 | 2.80 | 0.72 | 0.36 | 0.00 | 2.87 | 0.000 | - && - && - |
| novel | Contig7 | 1014586 | 1018108 | + | 3069 |  | 8.13 | 9.29 | 9.08 | 31.87 | 32.82 | 29.54 | -1.83 | 0.000 | - && - && - |
| novel | Contig7 | 1179535 | 1180897 | + | 1107 |  | 17.37 | 18.41 | 18.40 | 7.94 | 10.57 | 6.50 | 1.12 | 0.000 | - && - && - |
| novel | Contig7 | 1195412 | 1197231 | + | 1700 |  | 17.92 | 16.42 | 17.03 | 47.74 | 45.59 | 64.48 | -1.62 | 0.000 | - && - && - |
| novel | Contig7 | 1208286 | 1212546 | + | 4037 |  | 2.49 | 2.41 | 3.10 | 9.66 | 7.91 | 4.24 | -1.45 | 0.000 | - && - && - |
| novel | Contig7 | 1487844 | 1489668 | + | 1764 |  | 0.56 | 0.68 | 0.57 | 1.43 | 3.66 | 1.29 | -1.82 | 0.001 | - && - && - |
| novel | Contig7 | 1492593 | 1494751 | + | 2038 |  | 1.84 | 1.72 | 1.34 | 0.92 | 0.46 | 1.08 | 1.00 | 0.028 | - && - && - |
| novel | Contig7 | 2205252 | 2206019 | + | 674 |  | 4.24 | 2.38 | 3.52 | 1.39 | 1.39 | 0.65 | 1.57 | 0.006 | - && - && - |
| novel | Contig7 | 2244403 | 2247562 | + | 3048 |  | 8.99 | 9.55 | 9.05 | 3.80 | 2.36 | 1.76 | 1.80 | 0.000 | - && - && - |
| novel | Contig7 | 608420 | 611041 | - | 2426 |  | 50.48 | 74.82 | 37.16 | 13.84 | 15.67 | 12.19 | 1.96 | 0.000 | - && - && - |
| novel | Contig1 | 3992286 | 3994182 | - | 1427 |  | 4.21 | 5.21 | 5.95 | 57.85 | 74.85 | 55.78 | -3.62 | 0.000 | - && Q9Y6Z9.1 RecName: Full=Sorbose reductase sou1; AltName: Full=Sorbitol utilization protein sou1 && - |
| novel | Contig8 | 461734 | 462622 | + | 816 |  | 13.29 | 8.24 | 11.19 | 4.47 | 6.77 | 2.15 | 1.29 | 0.001 | - && - && - |
| novel | Contig8 | 721213 | 721898 | + | 686 |  | 1.15 | 0.29 | 1.46 | 0.00 | 0.27 | 0.00 | 3.41 | 0.019 | - && - && - |
| novel | Contig8 | 1412091 | 1413386 | + | 1124 |  | 2.19 | 2.77 | 2.11 | 1.33 | 0.75 | 1.25 | 1.09 | 0.025 | - && - && - |
| novel | Contig8 | 1721920 | 1723590 | + | 1388 |  | 2.98 | 3.04 | 2.04 | 0.67 | 0.40 | 1.26 | 1.78 | 0.000 | - && - && - |
| novel | Contig8 | 441723 | 442257 | - | 422 |  | 7.01 | 8.33 | 7.14 | 1.77 | 5.99 | 3.33 | 1.02 | 0.038 | - && - && - |
| novel | Contig8 | 598553 | 600776 | - | 2002 |  | 15.81 | 17.00 | 8.44 | 7.71 | 3.23 | 5.08 | 1.36 | 0.000 | - && - && - |
| novel | Contig8 | 1021850 | 1022905 | - | 835 |  | 20.19 | 21.88 | 14.33 | 7.95 | 12.56 | 6.62 | 1.06 | 0.001 | - && - && - |
| novel | Contig8 | 1028413 | 1029608 | - | 919 |  | 0.21 | 0.11 | 0.70 | 2.44 | 0.92 | 1.05 | -2.11 | 0.015 | - && - && - |
| novel | Contig8 | 1253533 | 1254408 | - | 814 |  | 0.85 | 2.22 | 3.25 | 5.74 | 2.99 | 5.28 | -1.15 | 0.026 | - && - && - |
| novel | Contig8 | 1425072 | 1427716 | - | 2226 |  | 1.59 | 2.16 | 0.98 | 3.65 | 4.67 | 4.18 | -1.40 | 0.000 | - && - && - |
| novel | Contig8 | 1475115 | 1476155 | - | 989 |  | 2.79 | 3.15 | 3.14 | 0.76 | 0.19 | 0.53 | 2.62 | 0.000 | - && - && - |
| novel | Contig9 | 624655 | 626680 | + | 1897 |  | 4.94 | 5.98 | 4.09 | 2.02 | 2.27 | 1.71 | 1.32 | 0.000 | - && - && - |
| novel | Contig9 | 894739 | 895100 | + | 303 |  | 27.01 | 18.55 | 17.79 | 10.18 | 11.43 | 8.40 | 1.08 | 0.002 | - && - && - |
| novel | Contig9 | 926429 | 927795 | + | 1219 |  | 0.57 | 0.66 | 0.52 | 0.77 | 2.07 | 1.87 | -1.43 | 0.023 | - && - && - |
| novel | Contig9 | 931597 | 933502 | - | 1713 |  | 0.98 | 1.47 | 1.55 | 3.55 | 2.30 | 2.15 | -1.00 | 0.015 | - && - && - |
| novel | Contig1 | 4621591 | 4621998 | - | 347 |  | 0.85 | 0.29 | 0.53 | 4.85 | 3.51 | 1.52 | -2.57 | 0.007 | - && - && - |
| novel | Contig10 | 236855 | 242662 | + | 4836 |  | 10.62 | 9.84 | 10.33 | 4.68 | 3.83 | 4.43 | 1.25 | 0.000 | - && - && - |
| novel | Contig1 | 634460 | 637318 | + | 2305 |  | 2.10 | 2.18 | 2.42 | 6.53 | 7.51 | 7.27 | -1.67 | 0.000 | - && G2QJ27.1 RecName: Full=Acetylesterase; AltName: Full=Carbohydrate esterase family 16 protein; Flags: Precursor && PF00657:GDSL-like Lipase/Acylhydrolase |
| novel | Contig10 | 251332 | 252014 | + | 627 |  | 0.94 | 0.16 | 0.73 | 2.68 | 5.08 | 1.12 | -2.28 | 0.005 | - && - && - |
| novel | Contig10 | 370048 | 370613 | + | 500 |  | 2.76 | 1.81 | 0.55 | 0.00 | 0.75 | 0.00 | 2.77 | 0.025 | - && - && - |
| novel | Contig10 | 459050 | 459719 | + | 670 |  | 1.32 | 0.90 | 1.23 | 0.42 | 0.28 | 0.13 | 2.06 | 0.036 | - && - && - |
| novel | Contig10 | 734332 | 735681 | + | 1121 |  | 4.93 | 5.64 | 4.81 | 1.33 | 1.75 | 1.02 | 1.90 | 0.000 | - && - && - |
| novel | Contig10 | 872397 | 874296 | + | 1564 |  | 2.33 | 4.36 | 2.51 | 26.60 | 21.85 | 29.07 | -3.07 | 0.000 | - && - && PF04616:Glycosyl hydrolases family 43 |
| novel | Contig10 | 1299175 | 1300123 | + | 882 |  | 2.35 | 3.07 | 2.80 | 1.48 | 0.53 | 0.10 | 1.96 | 0.004 | - && - && - |
| novel | Contig10 | 673860 | 674696 | - | 594 |  | 8.13 | 5.75 | 8.92 | 4.25 | 3.47 | 1.03 | 1.38 | 0.004 | - && - && - |
| novel | Contig10 | 1304463 | 1305672 | - | 1011 |  | 1.76 | 1.49 | 1.36 | 0.92 | 0.56 | 0.43 | 1.26 | 0.044 | - && - && - |
| novel | Contig10 | 1356489 | 1358512 | - | 1692 |  | 122.55 | 128.51 | 56.09 | 32.99 | 24.29 | 28.01 | 1.85 | 0.000 | - && - && - |
| novel | Contig10 | 1388079 | 1390185 | - | 1628 |  | 12.72 | 19.30 | 28.45 | 991.07 | 1029.54 | 954.73 | -5.62 | 0.000 | - && D4AK17.1 RecName: Full=PI-PLC X domain-containing protein 1; Flags: Precursor && - |
| novel | Contig11 | 296665 | 299175 | + | 2389 |  | 51.67 | 54.54 | 43.44 | 9.71 | 5.02 | 5.25 | 2.91 | 0.000 | - && - && PF11807:Mycotoxin biosynthesis protein UstYa |
| novel | Contig11 | 974666 | 976039 | + | 1314 |  | 3.15 | 5.65 | 4.59 | 2.42 | 2.21 | 1.54 | 1.12 | 0.003 | - && - && - |
| novel | Contig11 | 1136286 | 1137566 | + | 1165 |  | 181.21 | 223.27 | 108.29 | 25.60 | 16.87 | 37.82 | 2.67 | 0.000 | - && - && - |
| novel | Contig1 | 975675 | 976579 | + | 693 |  | 116.67 | 97.20 | 104.27 | 33.86 | 46.87 | 47.24 | 1.31 | 0.000 | - && - && PF08650:DASH complex subunit Dad4 |
| novel | Contig11 | 331873 | 336525 | - | 4596 |  | 6.93 | 5.92 | 5.80 | 2.62 | 2.97 | 2.50 | 1.20 | 0.000 | - && - && - |
| novel | Contig11 | 636507 | 636874 | - | 302 |  | 2.94 | 3.32 | 3.02 | 0.00 | 0.31 | 0.29 | 3.95 | 0.002 | - && - && - |
| novel | Contig11 | 1113828 | 1114542 | - | 609 |  | 17.00 | 25.22 | 3.15 | 0.31 | 0.61 | 0.58 | 4.92 | 0.000 | - && - && - |
| novel | Contig11 | 1134061 | 1136128 | - | 1764 |  | 5.70 | 3.81 | 3.42 | 1.59 | 1.17 | 2.19 | 1.39 | 0.000 | - && - && - |
| novel | Contig11 | 1165723 | 1167195 | - | 1440 |  | 0.41 | 0.49 | 1.27 | 3.31 | 1.69 | 2.13 | -1.72 | 0.002 | - && - && - |
| novel | Contig11 | 1186327 | 1186846 | - | 406 |  | 4.37 | 1.73 | 1.80 | 89.58 | 97.99 | 65.49 | -5.00 | 0.000 | - && - && - |
| novel | Contig11 | 1187035 | 1187653 | - | 499 |  | 0.99 | 1.41 | 1.28 | 2.81 | 4.69 | 4.40 | -1.69 | 0.005 | - && - && - |
| novel | Contig12 | 12415 | 14769 | + | 1727 |  | 8.28 | 5.70 | 8.57 | 17.22 | 16.64 | 23.38 | -1.34 | 0.000 | - && - && - |
| novel | Contig1 | 75314 | 76365 | + | 940 |  | 1.15 | 1.17 | 1.85 | 4.28 | 5.18 | 1.59 | -1.40 | 0.010 | - && - && - |
| novel | Contig12 | 83183 | 84552 | + | 1191 |  | 0.58 | 1.52 | 1.38 | 0.16 | 0.31 | 0.52 | 1.82 | 0.018 | - && - && - |
| novel | Contig12 | 624104 | 626101 | + | 1711 |  | 4.03 | 4.05 | 1.66 | 1.42 | 0.33 | 0.51 | 2.11 | 0.000 | - && - && - |
| novel | Contig12 | 34275 | 35091 | - | 763 |  | 0.90 | 1.32 | 1.44 | 7.72 | 1.72 | 5.18 | -2.00 | 0.001 | - && - && - |
| novel | Contig12 | 637241 | 639240 | - | 1872 |  | 0.42 | 0.48 | 0.24 | 0.00 | 0.05 | 0.05 | 3.57 | 0.009 | - && - && - |
| novel | Contig12 | 793227 | 795463 | - | 2065 |  | 10.98 | 9.87 | 9.86 | 4.80 | 4.85 | 2.76 | 1.31 | 0.000 | - && - && - |
| novel | Contig12 | 1011067 | 1012256 | - | 1119 |  | 2.11 | 1.88 | 1.39 | 0.50 | 1.09 | 0.16 | 1.63 | 0.014 | - && - && - |
| novel | Contig13 | 61923 | 65284 | + | 3299 |  | 3.71 | 1.89 | 1.63 | 1.11 | 1.25 | 1.17 | 1.04 | 0.005 | - && - && - |
| novel | Contig13 | 660770 | 661501 | + | 661 |  | 0.60 | 1.21 | 0.97 | 0.14 | 0.28 | 0.00 | 2.71 | 0.031 | - && - && - |
| novel | Contig13 | 243888 | 244832 | - | 778 |  | 3.42 | 1.81 | 3.87 | 16.46 | 24.79 | 12.52 | -2.56 | 0.000 | - && - && - |
| novel | Contig13 | 277198 | 279514 | - | 2099 |  | 1.03 | 1.24 | 1.52 | 0.53 | 0.58 | 0.25 | 1.48 | 0.005 | - && - && - |
| novel | Contig13 | 711667 | 714087 | - | 2358 |  | 0.71 | 0.81 | 0.77 | 0.48 | 0.24 | 0.22 | 1.29 | 0.030 | - && - && - |
| novel | Contig14 | 137879 | 139397 | + | 1344 |  | 8.88 | 6.65 | 11.42 | 1.32 | 1.18 | 0.91 | 2.98 | 0.000 | - && - && - |
| novel | Contig14 | 283263 | 285131 | + | 1821 |  | 5.25 | 3.80 | 3.01 | 2.26 | 2.52 | 1.25 | 1.00 | 0.007 | - && - && - |
| novel | Contig14 | 797590 | 799297 | + | 1279 |  | 54.66 | 37.28 | 80.35 | 420.91 | 374.30 | 323.31 | -2.70 | 0.000 | - && Q9LIS2.1 RecName: Full=Glycine-rich RNA-binding protein 4, mitochondrial; Short=AtGR-RBP4; AltName: Full=AtRBG4; AltName: Full=Glycine-rich protein 4; Short=AtGRP4; AltName: Full=Mitochondrial RNA-binding protein 1b; Short=At-mRBP1b; Flags: Precursor && PF00076:RNA recognition motif. (a.k.a. RRM, RBD, or RNP domain) |
| novel | Contig14 | 290734 | 292238 | - | 1342 |  | 1.32 | 1.20 | 0.88 | 3.90 | 2.44 | 1.70 | -1.24 | 0.010 | - && - && - |
| novel | Contig14 | 804164 | 805448 | - | 1285 |  | 1.53 | 1.48 | 2.20 | 0.44 | 1.17 | 0.82 | 1.11 | 0.042 | - && - && - |
| novel | Contig14 | 934633 | 935964 | - | 1058 |  | 1.58 | 1.90 | 1.90 | 5.30 | 8.58 | 15.10 | -2.43 | 0.000 | - && - && - |
| novel | Contig15 | 65261 | 66163 | + | 765 |  | 8.38 | 8.40 | 3.46 | 2.08 | 2.69 | 2.98 | 1.38 | 0.002 | - && - && - |
| novel | Contig15 | 161804 | 162757 | - | 903 |  | 1.64 | 2.22 | 1.62 | 5.90 | 1.97 | 3.98 | -1.11 | 0.026 | - && - && - |
| novel | Contig15 | 175866 | 177583 | - | 1661 |  | 5.82 | 5.20 | 6.71 | 2.03 | 2.82 | 2.27 | 1.32 | 0.000 | - && - && - |
| novel | Contig15 | 276917 | 279076 | - | 1915 |  | 3.30 | 4.93 | 3.48 | 7.96 | 10.71 | 9.21 | -1.25 | 0.000 | - && - && - |
| novel | Contig15 | 475055 | 476987 | - | 1547 |  | 16.25 | 10.84 | 10.87 | 4.23 | 1.27 | 0.85 | 2.58 | 0.000 | - && Q9FPR3.1 RecName: Full=Serine/threonine-protein kinase EDR1; AltName: Full=MAPKK kinase EDR1; AltName: Full=Protein ENHANCED DISEASE RESISTANCE 1; Short=AtEDR1; AltName: Full=Serine/threonine/tyrosine-protein kinase 10 && PF00069:Protein kinase domain |
| novel | Contig1 | 3007901 | 3010651 | + | 2484 |  | 0.48 | 0.44 | 0.66 | 0.72 | 1.32 | 1.31 | -1.08 | 0.031 | - && - && - |
| novel | Contig16 | 426303 | 429046 | + | 2632 |  | 1.24 | 0.99 | 0.87 | 0.75 | 0.46 | 0.30 | 1.04 | 0.038 | - && - && - |
| novel | Contig16 | 718063 | 719411 | + | 1232 |  | 12.65 | 10.43 | 10.01 | 4.55 | 2.36 | 1.21 | 2.03 | 0.000 | - && Q9UT59.1 RecName: Full=Putative uncharacterized oxidoreductase C513.07 && PF01073:3-beta hydroxysteroid dehydrogenase/isomerase family |
| novel | Contig16 | 779506 | 782128 | + | 2221 |  | 2.40 | 2.80 | 1.89 | 8.08 | 4.76 | 6.68 | -1.46 | 0.000 | - && Q8RWL6.1 RecName: Full=Serine/threonine-protein kinase STY17; AltName: Full=Serine/threonine/tyrosine-protein kinase 17 && PF07714:Protein tyrosine and serine/threonine kinase |
| novel | Contig16 | 798628 | 799902 | + | 1169 |  | 12.15 | 8.50 | 15.39 | 5.04 | 7.53 | 2.70 | 1.24 | 0.001 | - && - && - |
| novel | Contig16 | 195615 | 197182 | - | 1454 |  | 0.88 | 1.17 | 1.01 | 0.45 | 0.26 | 0.12 | 1.89 | 0.009 | - && - && - |
| novel | Contig16 | 203165 | 207263 | - | 3759 |  | 7.40 | 8.44 | 7.29 | 3.41 | 3.64 | 3.20 | 1.18 | 0.000 | - && - && - |
| novel | Contig16 | 229303 | 231392 | - | 1737 |  | 4.14 | 2.66 | 2.26 | 0.97 | 1.78 | 0.56 | 1.46 | 0.002 | - && - && - |
| novel | Contig17 | 256912 | 258591 | + | 1338 |  | 1.40 | 2.40 | 0.89 | 4.19 | 2.80 | 3.80 | -1.20 | 0.006 | - && - && - |
| novel | Contig17 | 667479 | 669635 | + | 1915 |  | 6.33 | 5.40 | 4.72 | 0.83 | 1.03 | 1.01 | 2.52 | 0.000 | - && - && - |
| novel | Contig17 | 685490 | 686013 | + | 404 |  | 1061.93 | 1018.05 | 630.86 | 230.73 | 316.98 | 44.31 | 2.20 | 0.004 | - && - && - |
| novel | Contig18 | 310932 | 316888 | + | 4978 |  | 6.56 | 5.32 | 5.12 | 1.90 | 2.41 | 2.06 | 1.42 | 0.000 | - && - && - |
| novel | Contig18 | 500062 | 501133 | + | 987 |  | 1.80 | 1.42 | 1.30 | 0.76 | 0.38 | 0.00 | 1.99 | 0.014 | - && - && - |
| novel | Contig18 | 440003 | 440591 | - | 512 |  | 0.19 | 0.59 | 0.00 | 0.55 | 1.28 | 3.09 | -2.65 | 0.025 | - && - && - |
| novel | Contig19 | 109421 | 109748 | + | 277 |  | 3.56 | 4.35 | 5.28 | 1.35 | 1.35 | 1.27 | 1.73 | 0.019 | - && - && - |
| novel | Contig19 | 172993 | 173599 | + | 546 |  | 1.26 | 1.65 | 2.68 | 0.00 | 0.17 | 0.80 | 2.52 | 0.014 | - && - && - |
| novel | Contig19 | 377823 | 379175 | + | 1299 |  | 49.04 | 66.00 | 44.66 | 10.80 | 14.70 | 3.85 | 2.44 | 0.000 | - && - && - |
| novel | Contig19 | 72896 | 73498 | - | 446 |  | 0.22 | 1.13 | 1.02 | 4.61 | 6.09 | 5.90 | -2.81 | 0.000 | - && - && - |
| novel | Contig19 | 482703 | 483449 | - | 631 |  | 2.81 | 3.18 | 4.20 | 2.07 | 1.04 | 1.39 | 1.18 | 0.030 | - && - && - |
| novel | Contig2 | 674562 | 675968 | + | 1307 |  | 0.53 | 0.61 | 0.70 | 1.43 | 2.44 | 1.21 | -1.46 | 0.012 | - && - && - |
| novel | Contig2 | 1228914 | 1229631 | + | 604 |  | 127.66 | 90.25 | 166.06 | 16.41 | 8.99 | 9.59 | 3.46 | 0.000 | - && - && - |
| novel | Contig1 | 3641549 | 3644129 | + | 2344 |  | 40.26 | 42.70 | 46.34 | 13.16 | 17.33 | 9.92 | 1.68 | 0.000 | - && - && - |
| novel | Contig2 | 2130704 | 2131027 | + | 324 |  | 4.26 | 1.86 | 3.95 | 11.25 | 2.89 | 9.75 | -1.25 | 0.043 | - && - && - |
| novel | Contig2 | 2370564 | 2377054 | + | 3425 |  | 72.43 | 127.38 | 38.89 | 18.64 | 19.79 | 14.89 | 2.16 | 0.000 | - && - && - |
| novel | Contig2 | 2479343 | 2486962 | + | 2422 |  | 17.87 | 17.16 | 16.07 | 38.06 | 43.21 | 39.57 | -1.24 | 0.000 | - && - && - |
| novel | Contig2 | 3185794 | 3187681 | + | 1470 |  | 224.04 | 154.47 | 211.60 | 123.39 | 72.60 | 53.61 | 1.24 | 0.000 | - && - && - |
| novel | Contig2 | 3217290 | 3218464 | + | 1038 |  | 3.80 | 3.68 | 3.26 | 2.25 | 0.90 | 1.44 | 1.22 | 0.006 | - && - && - |
| novel | Contig2 | 3508933 | 3509294 | + | 231 |  | 7.26 | 9.56 | 7.12 | 1.62 | 0.81 | 0.00 | 3.30 | 0.000 | - && - && - |
| novel | Contig2 | 1073961 | 1075527 | - | 1508 |  | 69.38 | 69.17 | 80.63 | 172.98 | 215.15 | 414.35 | -1.87 | 0.000 | - && - && - |
| novel | Contig2 | 2888223 | 2889199 | - | 862 |  | 163.69 | 177.14 | 66.87 | 13.67 | 13.68 | 28.40 | 2.87 | 0.000 | - && - && - |
| novel | Contig2 | 2973474 | 2974998 | - | 1405 |  | 1.54 | 1.64 | 2.15 | 0.20 | 0.93 | 0.19 | 2.01 | 0.002 | - && - && - |
| novel | Contig2 | 3146240 | 3155332 | - | 7496 |  | 5.08 | 5.83 | 5.15 | 17.25 | 12.65 | 11.91 | -1.38 | 0.000 | - && O15050.4 RecName: Full=TPR and ankyrin repeat-containing protein 1; AltName: Full=Lupus brain antigen 1 homolog && PF13361:UvrD-like helicase C-terminal domain |
| novel | Contig1 | 4085436 | 4086709 | + | 1219 |  | 0.97 | 0.66 | 0.75 | 4.30 | 3.61 | 2.30 | -2.10 | 0.000 | - && - && - |
| novel | Contig1 | 4174121 | 4175277 | + | 905 |  | 50.12 | 41.71 | 36.24 | 25.52 | 14.58 | 19.98 | 1.09 | 0.000 | - && - && - |
| novel | Contig1 | 4175763 | 4176623 | + | 394 |  | 4.75 | 8.15 | 7.65 | 1.66 | 4.75 | 3.12 | 1.11 | 0.034 | - && - && - |
| novel | Contig20 | 242926 | 245878 | + | 2901 |  | 3.77 | 2.56 | 1.89 | 0.81 | 0.77 | 0.64 | 1.89 | 0.000 | - && - && - |
| novel | Contig20 | 247413 | 254958 | + | 6375 |  | 2.52 | 2.49 | 2.91 | 6.60 | 9.02 | 3.83 | -1.30 | 0.000 | - && P40288.1 RecName: Full=Glucose 1-dehydrogenase && - |
| novel | Contig20 | 228322 | 232121 | - | 3298 |  | 3.50 | 2.07 | 2.71 | 1.42 | 0.85 | 0.77 | 1.45 | 0.000 | - && - && - |
| novel | Contig23 | 239679 | 241516 | + | 1713 |  | 1.96 | 1.70 | 1.81 | 0.65 | 0.82 | 0.56 | 1.42 | 0.002 | - && - && - |

*FC is abbreviated for fold-change; and “- && - &&” indicates that the marked gene encodes a hypothesis protein.
