# Supplementary material for: A complex metabolic network and its biomarkers regulate laccase production in white-rot fungus Cerrena unicolor 87613
Source: Microb Cell Fact. 2024 Jun 8;23:167. doi: 10.1186/s12934-024-02443-9 (PMC11162070; doi:10.1186/s12934-024-02443-9)
Supplement: Supplementary file 9 — Supplementary Material 9 [file 12934_2024_2443_MOESM9_ESM.docx]

**SUPPLEMENTARY FIGURE LEGENDS**


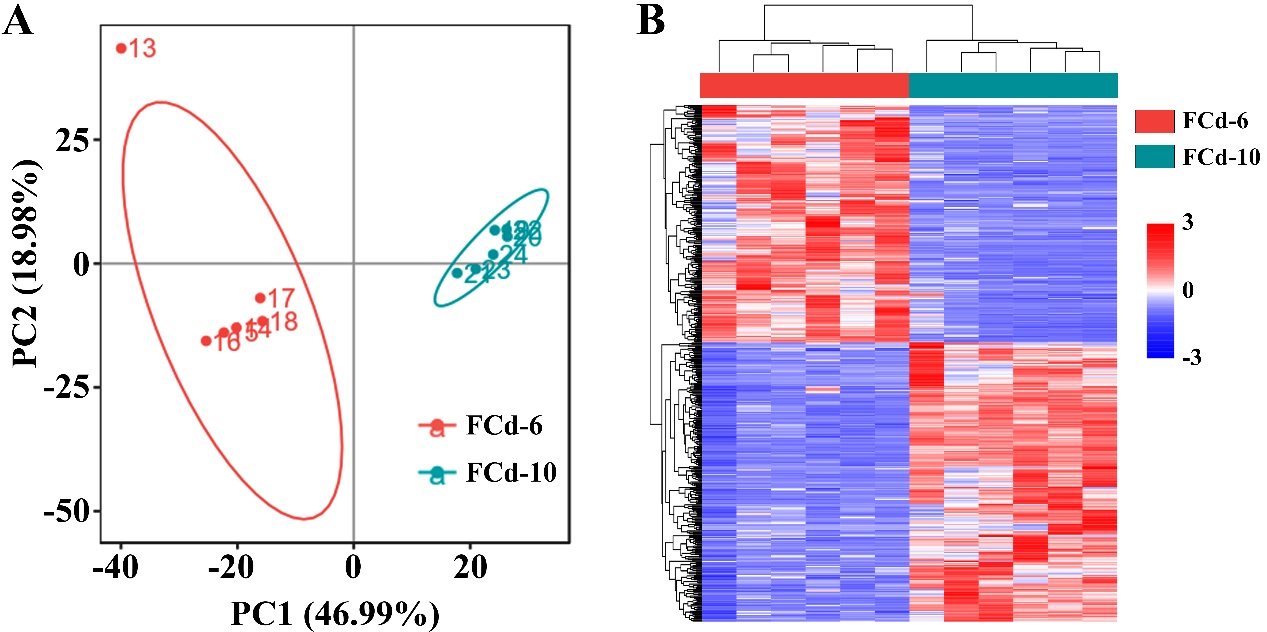


**Fig. S1** Quantity analyses of metabolomic data between FCd-6 and FCd-10 group. **A** Score plot (PC1 vs PC2) of principal component analysis (PCA) of metabolites extracted from FCd-6 compared to those from FCd-10. The orange circle indicated FCd-6 group, whereas the green circle indicated FCd-10 group. B Heat map of all detected metabolites. Red and blue colors indicate increased and decreased levels of metabolites relative to the median metabolite level, respectively (see color scale).


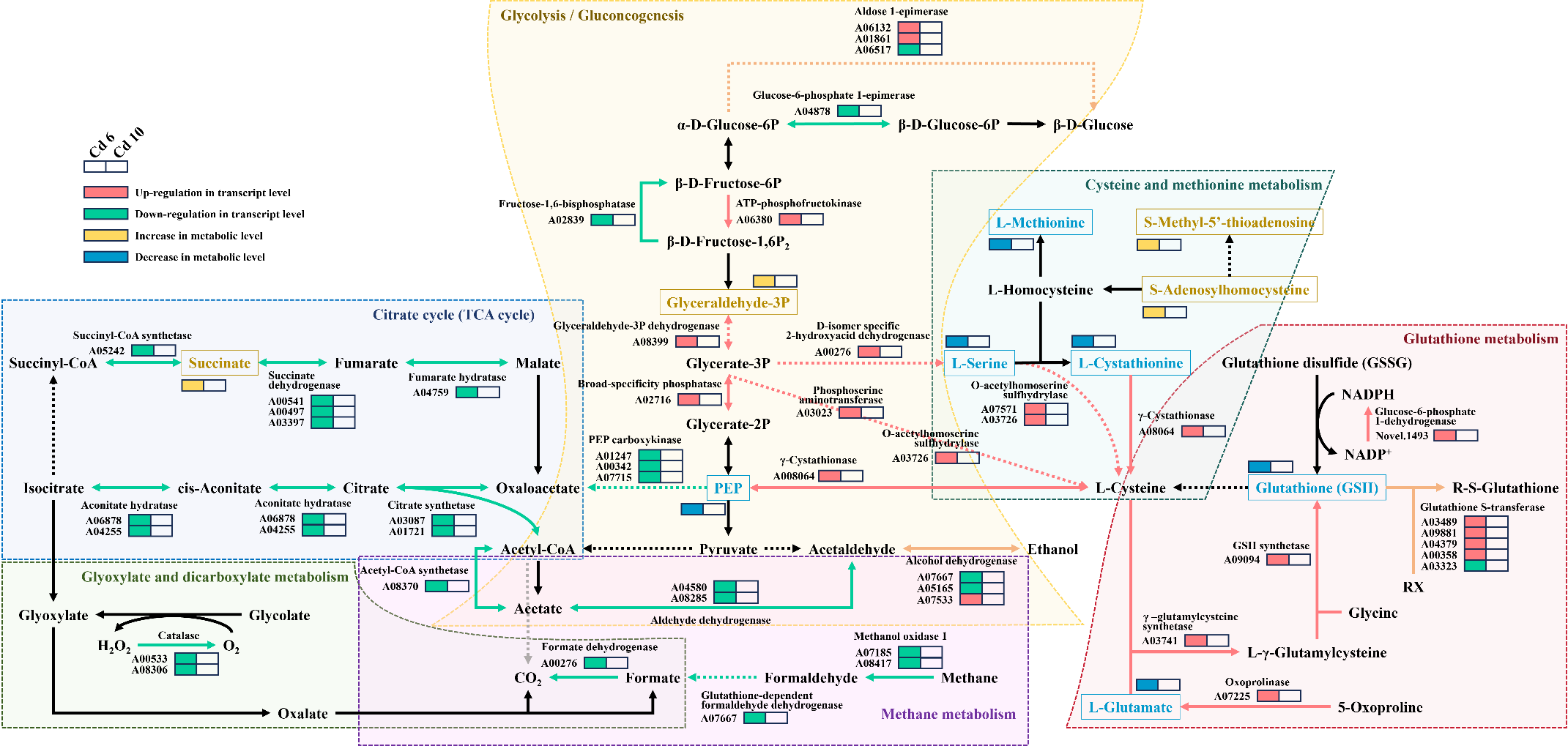


**Fig. S2** The detailed illustration of complicated metabolic networks co-founded by both transcriptomic and metabolomic analysis. The left and right box indicated FCd-6 and FCd-10 samples, respectively. The red box indicated significantly up-regulated pattern of transcription, whereas the green box indicated down-regulated pattern of transcription. The yellow box indicated significantly increased pattern of metabolome, whereas the blue box indicated significantly decreased pattern of metabolome.


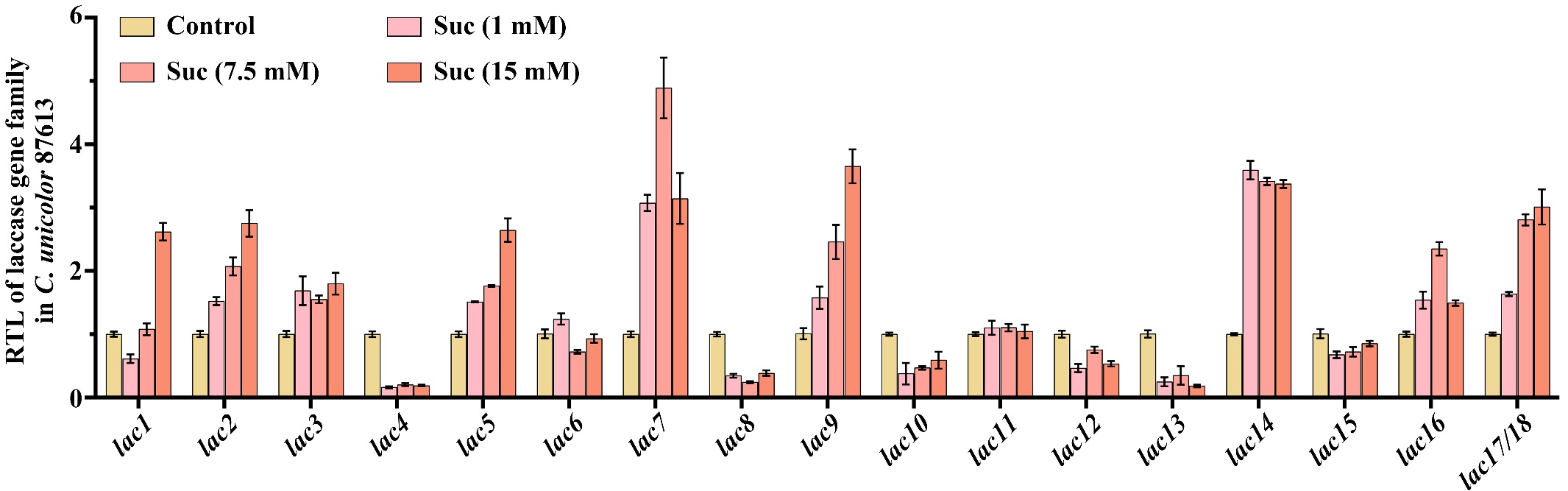


**Fig. S3** Alteration of transcript level of laccase gene family (*lac1* – *18*) in response to 2-day treatment of succinate (with the concentration of 1, 7.5, or 15 mM, respectively) in *C. unicolor* 87613.


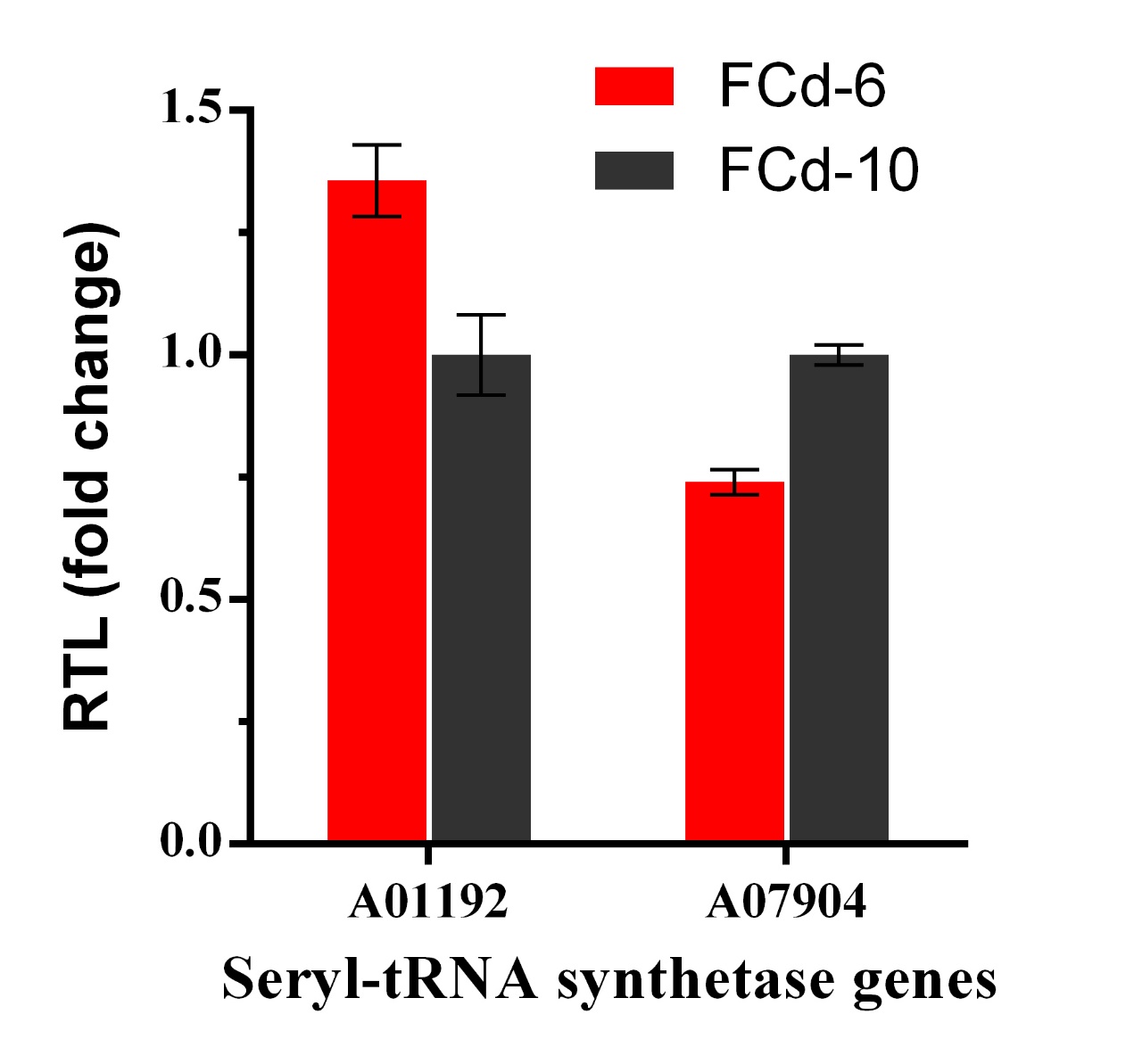


**Fig. S4** Fold changes of two seryl-tRNA synthetase encoding genes in FCd-6 (red boxes) compared to those in FCd-10 (black boxes).


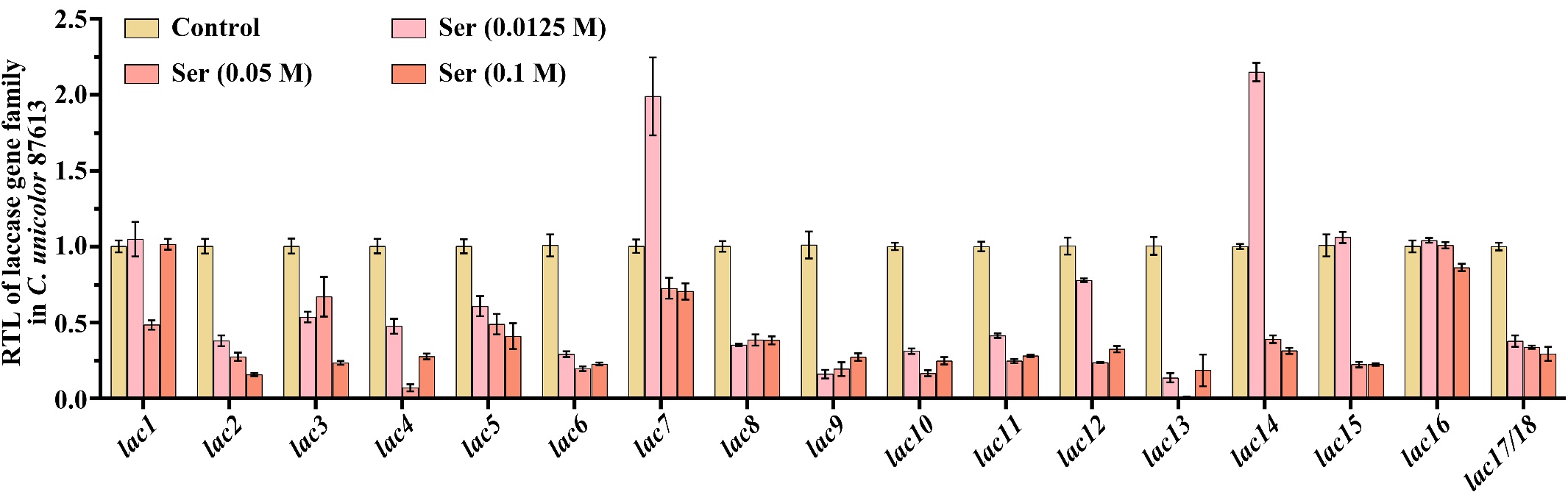


**Fig. S5** Alteration of transcript level of laccase gene family (*lac1* – *18*) in response to 2-day treatment of serine (with the concentration of 0.0125, 0.05, or 0.1 M, respectively) in *C. unicolor* 87613.


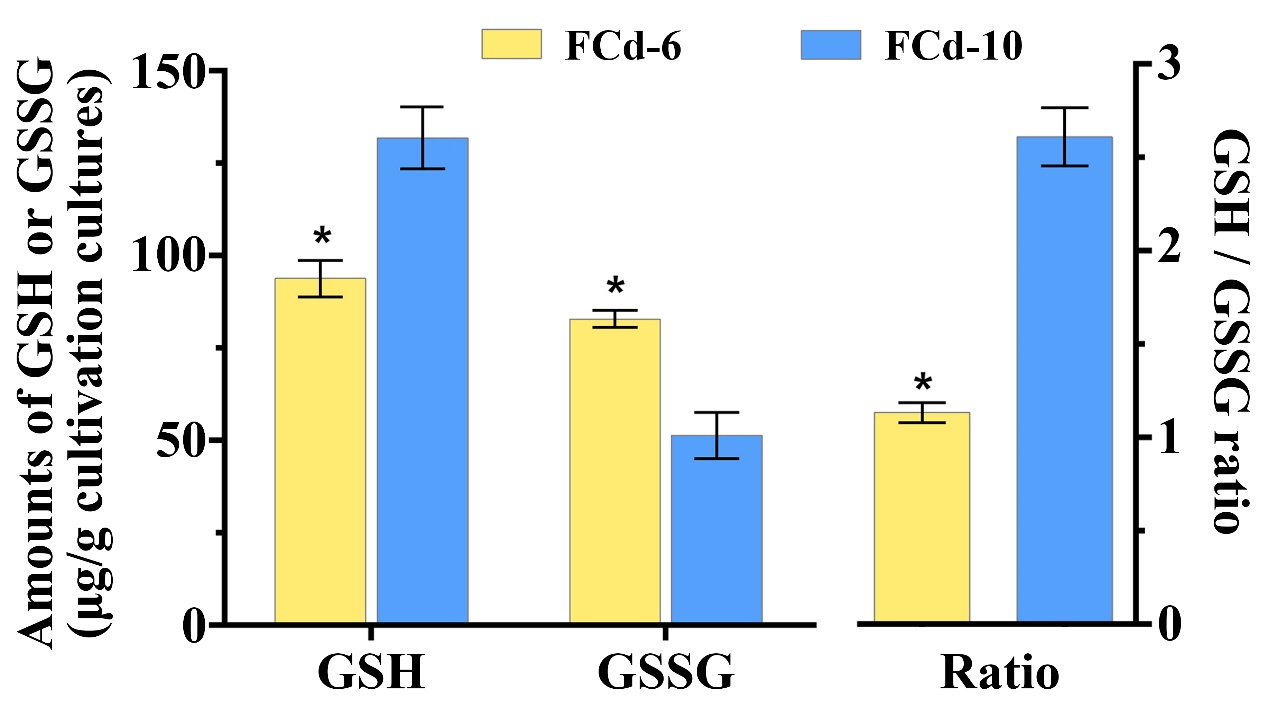


**Fig. S6** The amounts of GSH, GSSG and their ratio in FCd-6 (yellow boxes) compared to those in FCd-10 (blue boxes).
